# An RNA Interference Lethality Screen of the Human Druggable Genome to Identify Molecular Vulnerabilities in Epithelial Ovarian Cancer

**DOI:** 10.1371/journal.pone.0047086

**Published:** 2012-10-09

**Authors:** Geetika Sethi, Harsh B. Pathak, Hong Zhang, Yan Zhou, Margret B. Einarson, Vinod Vathipadiekal, Sumedha Gunewardena, Michael J. Birrer, Andrew K. Godwin

**Affiliations:** 1 Department of Pathology and Laboratory Medicine, University of Kansas Medical Center, Kansas City, Kansas, United States of America; 2 Department of Biochemistry, Drexel University College of Medicine, Philadelphia, Pennsylvania, United States of America; 3 University of Kansas Cancer Center, Kansas City, Kansas, United States of America; 4 Fox Chase Cancer Center, Philadelphia, Pennsylvania, United States of America; 5 Biostatistics and Bioinformatics Facility, Fox Chase Cancer Center, Philadelphia, Pennsylvania, United States of America; 6 Translational Core Facility, Fox Chase Cancer Center, Philadelphia, Pennsylvania, United States of America; 7 Massachusetts General Hospital, Harvard Medical School, Boston, Massachusetts, United States of America; 8 Molecular and Integrative Physiology, University of Kansas Medical Center, Kansas City, Kansas, United States of America; University of Texas Health Science Center at San Antonio, United States of America

## Abstract

Targeted therapies have been used to combat many tumor types; however, few have effectively improved the overall survival in women with epithelial ovarian cancer, begging for a better understanding of this deadly disease and identification of essential drivers of tumorigenesis that can be targeted effectively. Therefore, we used a loss-of-function screening approach to help identify molecular vulnerabilities that may represent key points of therapeutic intervention. We employed an unbiased high-throughput lethality screen using a 24,088 siRNA library targeting over 6,000 druggable genes and studied their effects on growth and/or survival of epithelial ovarian cancer (EOC) cell lines. The top 300 “hits” affecting the viability of A1847 cells were rescreened across additional EOC cell lines and non-tumorigenic, human immortalized ovarian epithelial cell lines. Fifty-three gene candidates were found to exhibit effects in all tumorigenic cell lines tested. Extensive validation of these hits refined the list to four high quality candidates (*HSPA5*, *NDC80*, *NUF2*, and *PTN*). Mechanistic studies show that silencing of three genes leads to increased apoptosis, while *HSPA5* silencing appears to alter cell growth through G1 cell cycle arrest. Furthermore, two independent gene expression studies show that *NDC80*, *NUF2* and *PTN* were significantly aberrantly overexpressed in serous adenocarcinomas. Overall, our functional genomics results integrated with the genomics data provide an important unbiased avenue towards the identification of prospective therapeutic targets for drug discovery, which is an urgent and unmet clinical need for ovarian cancer.

## Introduction

Epithelial ovarian cancer is the second most common gynecological cancer, and one of the deadliest, among women, with an estimated 22,280 new cases and 15,500 deaths for 2012. [1] Among the different types of epithelial ovarian cancer, which includes serous, mucinous, clear cell and endometrial [2], [3], the majority of deaths from ovarian cancer occur in patients with advanced-stage, high-grade serous ovarian cancer. [4] As such, there is an urgent need for new therapeutic approaches to combat this deadly disease.

Development of new therapies, especially in the era of targeted treatments and personalized medicine, is typically driven by understanding the underlying biology, molecular biology and biochemistry of tumor cells and their surrounding microenvironments targeting genetic alterations. [5] This is a common theme in drug discovery and can provide specificity, but cannot generally provide comprehensiveness in targeting. Cancer cells can evolve that lack the targeted genetic alterations or that are resistant and could cause progressive disease. [5] Therefore, it is essential to expand our armament of therapies, but more importantly our concept of important drug targets. The evolutionary nature of cancer implies, contrary to conventional wisdom, that the essential features of any therapy for the consistent cure or control of cancer must be independent of the particular pathways of tumor cell evolution, and independent of any particular genetic or epigenetic alterations. Although the genetic and epigenetic complexity of cancer is nearly unlimited, tumor cell evolution is constrained. [6], [7] A malignant cell will result, if and only if, the alterations cause normal cellular machinery to carry out the processes of proliferation and invasiveness.

Current drug discovery efforts tend to focus on commonly mutated signal transduction pathways, e.g., a series of growth factor receptors and downstream modulators (phosphatases and kinases) that are working in concert to promote growth but are not the central machinery. Therefore, we performed non-biased high-throughput lethality screens (HTS) of small interfering RNAs (siRNAs) to identify genes that are essential for ovarian tumor cell growth and survival. The top hits were extensively validated and their clinical value assessed. Overall, we found *NDC80*, *NUF2* and *PTN* as important molecular vulnerabilities, which represent potentially important therapeutic targets in ovarian cancer.

## Results

### HTS of the Druggable Genome

The primary high-throughput RNAi screen was performed (as depicted in **Figure S1A**) using the Human Druggable Genome Library (Dharmacon) (**Table S1)** consisting of 24,088 siRNAs targeting 6,022 genes using A1847 cells, an epithelial ovarian carcinoma (EOC) cell line, which consistently yielded reproducible transfection data under HTS conditions. Positive and negative control internal reference wells were included on every plate to allow for calculation of the transfection efficiency (see **Supplementary Information S1** for additional details). A1847 cells were transfected using HTS conditions as described in the **Material and Methods** section. The normalized viability scores (defined as the (fluorescence intensity_sample_)/(median fluorescence intensity_reference_)) obtained through the HTS displayed a Gaussian distribution (**Figure S1B**). Following statistical data analysis (see **Supplementary Information S1**), a total of 300 genes representing ∼5% of the genes targeted by this library were selected for inclusion in the next round of screening.

### HTS of a Panel of EOC Cell Lines Using a Subset of the Druggable Genome

**Figure 1:**
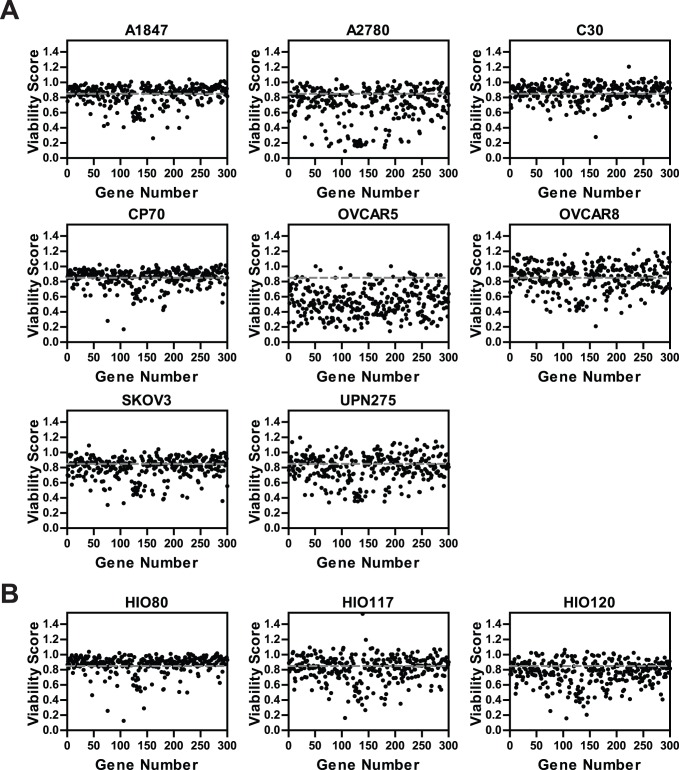
Secondary **screens on a panel of EOC and HIO cell lines. A.** Eight EOC cell lines were reverse transfected with siRNAs targeting 300 genes identified from the primary screening of the A1847 cell line using transfection parameters optimized for each cell line (see **Table S3**). Each circle represents an averaged viability score from technical replicates following silencing of a particular gene. The grey dotted line represents the cut off value for the viability score (0.85) to select hits. **B.** Three HIO cell lines were transfected using parameters optimized for each (see **Table S3**).

**Figure 2:**
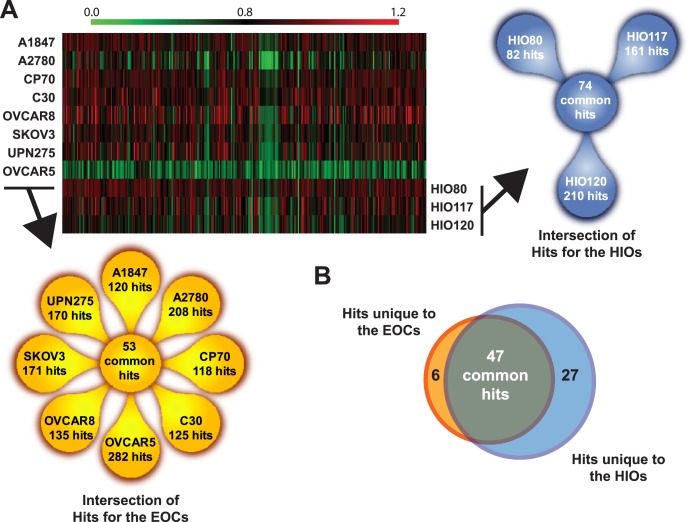
Hits unique to the EOC cell lines. **A.** A heat map representation of the viability scores for eight EOC and three HIO cell lines achieved from secondary screens of the siRNA library targeting 300 genes. All viability score values range between 0.12 and 1.53. Shades of green represent reduced viability (<0.80), shades of red represents increased viability (>0.80), and black represents a viability score of 0.80. The heat map was generated using MultiExperiment Viewer. Floral diagrams in yellow and blue show the number of hits across either the eight EOC or three HIO cell lines, respectively, and the intersection of the hits within each group. **B.** A Venn diagram shows the number of hits in common between the HIOs and EOCs and the number hits unique to each group.

Next, we determined which of the 300 genes identified as hits from the primary screen mutually affected the cell growth and survival across multiple EOC cell lines using an independent siRNA library (**Table S2**). This new focused library was designed using an entirely new pool of four siRNA sequences targeting each gene in order to minimize potential false-positives from the primary screen due to off-target effects. This strategy of using a different set of siRNA pools for the secondary screens has been adopted as a revalidation step in itself. [8] Transfection conditions for seven additional ovarian tumorigenic cell lines (A2780, CP70, C30, OVCAR5, OVCAR8, SKOV3 and UPN275) were then optimized for HTS (**Table S3**). These seven new EOC cell lines and the A1847 cell line were then subjected to HTS using the subset library targeting the 300 genes. A viability score was derived for all 300 siRNA pools in each cell line (shown graphically in **Figure 1A**) which ranged from 0.09–1.20 across the cell lines. “Hits” for each of the eight cell lines were selected based on both statistical significance (false discovery rate (FDR) <0.05) and biological significance (viability score <0.85) (see **Supplementary Information S1** for additional details). A heat map of the hits across each EOC cell line generated using MultiExperiment Viewer [9], [10] and the intersection of the hits among the cell lines are shown schematically in **Figure 2A**. A total of 53 hits were considered significant across all eight EOC cell lines (**Figure 2A** and **Table S4**). The average coefficient of correlation (r) for the technical replicates across all of the cell lines was 0.91±0.03 (**Figure S2A**).

The PANTHER biological classification system [11] showed that “metabolic processes” was the largest category (∼43%) to which these 53 genes belonged (**Figure S3A**). Specifically, when functional characterization of these genes was performed using Ingenuity Pathway Analysis (IPA) software, it was shown that the 53 hits were enriched for genes related to protein synthesis involving ribosomal proteins and elongation factors (**Figure S3B**). Recent reports have provided evidence that in addition to involvement in protein synthesis, ribosomal proteins and elongation factors have a role in cell cycle regulation and survival. [12], [13], [14], [15] Drugs targeting different molecular components involved in protein synthesis machinery are already in clinical trials for various tumor types including breast, colon, and colorectal cancers [16], [17], [18], supporting the translational potential of our hits. Network characterization using IPA software showed that the genes in the network with the highest score exhibited their downstream effects through, ERK1/2 and AKT, key survival genes which have been implicated as mediators of major oncogenic pathways in ovarian cancer (**Figure S3C**) [19], [20], [21], [22].

### HTS of Non-tumorigenic HIO Lines

Next, we determined which of the hits had the greatest effect on the EOC cell lines and little or no effect on the non-tumorigenic human immortalized ovarian surface epithelial (HIO) cell lines. We, therefore, screened the 53 hits for effects on the viability of three HIO cell lines (HIO80, HIO120 and HIO117). Although we were interested in screening only the 53 hits, in order to maintain the same screening format and minimize any technical differences in how the siRNA screens were performed for the HIO cell lines, we again used the custom siRNA library targeting all 300 genes. A viability score was derived following silencing of each gene (**Figure 1B** **)**. The average coefficient of correlation between technical replicates for the three HIO cell lines (**Figure S2B**, average r = 0.90±0.03) was similar to that of the EOC cell lines.

Hits for the HIO cell lines were selected as described above (normalized viability score <0.85 and FDR <5%). A total of 74 hits were identified common to all three HIO cell lines (**Figure 2A**). A Venn diagram shows the intersection of the hits between the EOC and the HIO cell lines in **Figure 2B**. Forty-seven hits were in common between the two groups. However, there were six hits unique to the EOC cell lines (genes which affected the viability of all eight of the EOC cell lines but not all three of the HIO cell lines) which we selected for further validation. We also selected one additional gene, *NUF2*, for further validation studies. *NUF2*, although not a unique hit to the EOC cell lines, displayed the lowest Viability Index score, defined as the ratio of the average normalized viability of the EOC cell lines to the average normalized viability of the HIO cell lines (**Table S4**). These seven genes (*BCAR3*, *HSPA5*, *NAMPT*, *NDC80*, *NUF2*, *PTN*, and, *RPS19*) were further analyzed for potential off-target effects.

### Deconvolution of siRNA Pools

**Figure 3:**
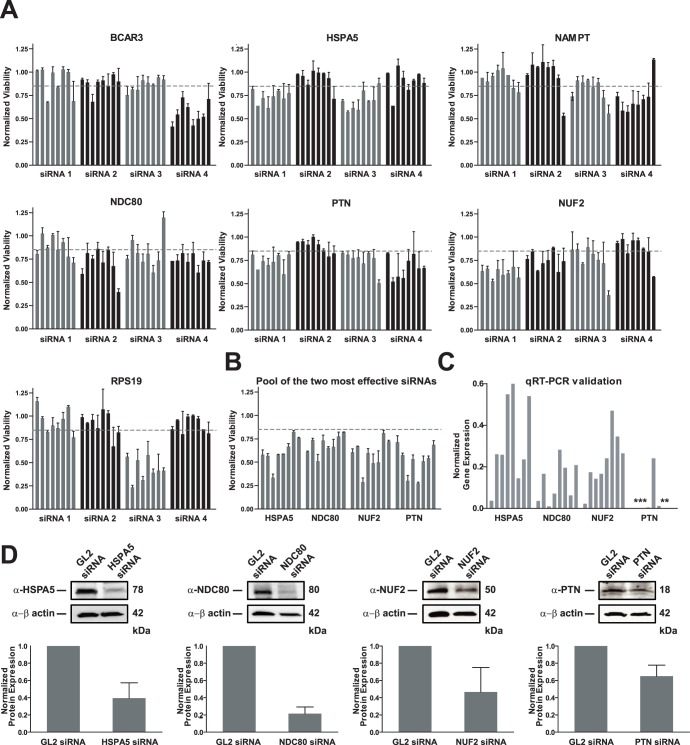
Validation of hits via siRNA deconvolution screening, qRT-PCR and Western blotting. **A.** The seven hits selected for further studies were validated by performing deconvolution screens where individual siRNAs which were initially a part of a pool of four siRNAs in the secondary screens were evaluated for their effects on cell viability. For each hit being validated, the average normalized viability scores (±SD) following gene silencing using each of the four species of siRNAs evaluated in the eight EOC cell lines are shown. Hits were considered on-target if viability scores of less than 0.85 were observed for all eight EOC cell lines for at least two independent siRNA species targeting a gene. The bar graphs represent the eight EOC cell lines in the following order: A1847, A2780, C30, CP70, OVCAR5, OVCAR8, SKOV3, and UPN275. **B.** The two most effective siRNAs targeting each gene were pooled (12.5 nM each siRNA species) and the effect on cell viability was quantified. The bars represent the eight EOC cells lines as described in Panel A. **C.** qRT-PCR was performed on all of the eight EOC cell lines following gene silencing for 72 h using a pool of the two most effective siRNAs identified from the deconvolution studies from panel A for the four hits that were determined to be on target. The asterisk represents *PTN* mRNA levels which are below the level of detection following siRNA treatment (i.e. complete knockdown of mRNA). **D.** Western blot analysis was performed following gene silencing for either 72 h (*HSPA5*, *NDC80*, and *PTN*) or 120 h (*NUF2*) to determine the level of knockdown at the protein level in A1847 cells. Immuno blots were quantified using AlphaView software, version 3.3 (Cell Biosciences).

To rule out off-target effects, we individually evaluated the four siRNAs from the siRNA pools used in the secondary screens targeting the seven genes. The 28 individual siRNAs in the deconvolution screen (**Table S5**) were evaluated in the panel of EOC cell lines. In order to accept any of the seven genes as having, valid on-target effects on the viability of EOC cell lines, we required that at least two out of the four individual siRNAs targeting each gene resulted in a viability score of 0.85 or less with a FDR of less than 5% across all eight of the EOC cell lines. [23], [24] Based on these stringent criteria, four genes, *HSPA5*, *NDC80*, *NUF2*, and *PTN*, were considered to be on-target, validated hits (**Figure 3A** and **Table 1**). Next, we pooled the two most effective siRNA species (highlighted in green, **Table S5**) targeting each of the four genes and quantified the level of reduction in cell viability for each cell line. These optimal siRNA pools resulted in greater than 30% reduction in cell viability across a majority of the EOC cell lines (**Figure 3B**).

**Table 1:** List of hits validated following deconvolution of siRNA pools.

Gene	Alternate Names & Description
*HSPA5*	Heat shock 70 kDa protein 5 (glucose-regulated protein, 78 kDa), GRP78
*NDC80*	Kinetochore protein HEC1, KNTC2
*NUF2*	NUF2, NDC80 kinetochore complex component, homolog (*S. cerevisiae*)
*PTN*	Pleiotrophin (heparin binding growth factor 8, neurite growth-promoting factor 1)

As an additional check on the ability of siRNAs to correctly target their mRNAs, we used the optimal pool of the two most effective siRNAs (**Figure 3B**) and performed quantitative RT-PCR to determine the level of mRNA knockdown following transfection of the pooled siRNAs for each cell line (**Figure 3C**). Messenger RNA levels of *HSPA5*, *NDC80*, *NUF2*, and *PTN* across all the eight EOC cell lines were shown to be reduced by an average of 67%, 87%, 77%, and 96%, respectively. Western blot analysis following transfection of one EOC cell line, A1847, was completed as an additional means to demonstrate specificity of the siRNA pools by evaluating the cellular levels of the respective proteins for the mRNAs being targeted. All four siRNA pools down-regulated the protein levels of their respective mRNA targets by ∼50% or more in this cell line (**Figure 3D**).

### Effects on Apoptosis and Cell Cycle Progression

**Figure 4:**
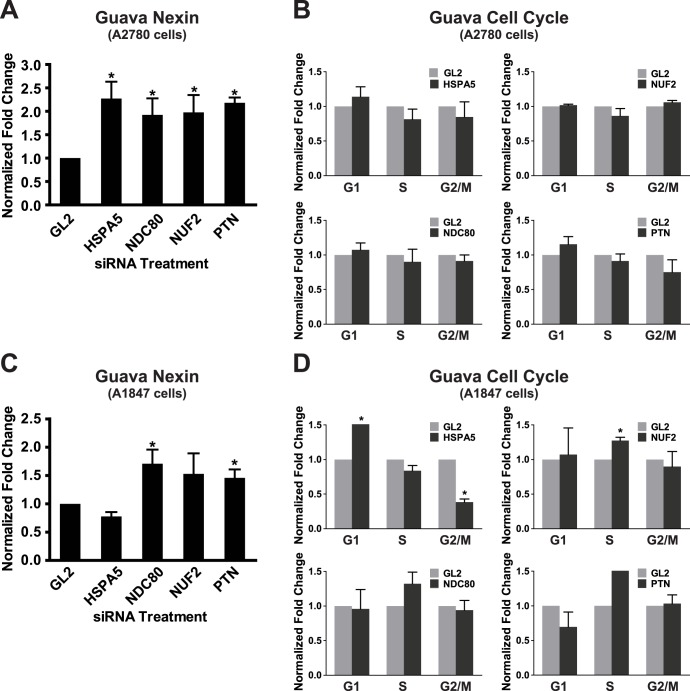
Effect of gene silencing on cell survival and cell cycle progression. A1847 and A2780 cells were transfected with *HSPA5*, *NDC80*, *NUF2*, *PTN* or *GL2* siRNAs. Seventy-two hours post-transfection, cells were harvested and processed for analysis of apoptosis or cell cycle progression. **A.** & **C.** The fraction of apoptotic cells was measured by annexin V staining followed by enumeration by using a Guava flow cytometer (Millipore). The fold-change in apoptotic cells is shown (mean ± SD, n = 3). **B** & **D.** The fraction of cells in each phase of the cell cycle was measured by propidium iodide staining followed by enumeration using the Guava instrument. The fold-change for each cell cycle phase is shown (mean ± SD, n = 3).

Our end-point parameter of cell viability that we used to identify the hits in the HTS studies provides limited information on the mechanism of decreased cell viability/growth induced by gene silencing. We, therefore, investigated the functional effects of targeting each of the four validated hits on apoptosis and cell cycle progression using two EOC cell lines, A1847 and A2780. We transfected these cell lines with a pool of the two most effective siRNA sequences (12.5 nM each siRNA, the same siRNA pool used for the quantitative RT-PCR (qRT-PCR) and Western blots analysis) for each of the four validated hits (*HSPA5, NDC80, NUF2* and *PTN*) and measured the effects on apoptosis and cell cycle progression 72 h following transfection in 96-well plates. In A2780 cells, knockdown of all four genes resulted in an increase in apoptotic cells as measured by positive annexin V staining using a Guava flow cytometer relative to cells transfected with *GL2*-targeting control siRNA (**Figure 4A**). On average, there was a 2.5-fold increase in apoptotic cells. However, in these cells, there was no significant effect on the cell cycle following knockdown of these four hits (**Figure 4B**).

In A1847 cells, knockdown of three genes (*NDC80*, *NUF2* and *PTN*) resulted in a 1.7-fold increase in apoptotic cells relative to cells transfected with *GL2*-targeting control siRNA (**Figure 4C**). Knockdown of *HSPA5* did not result in any significant increase in apoptosis (**Figure 4C**). However, we did observe that its knockdown did result in a 2-fold increase in the population of cells in the G1 phase of the cell cycle and a corresponding 2-fold decrease in the G2/M phase (**Figure 4D**). Knockdown of *NDC80*, *NUF2*, and *PTN* in A1847 cells resulted in a slight increase in the number of cells in the S phase (**Figure 4D**). However, this increase was, on average, less than 1.5-fold and did not appear to be highly statistically significant. Knockdown of these four genes did not have a measurable effect on survival of the non-tumorigenic HIO80 cells (**Figure S4**).

### Assessment of Validated Hits in Clinical Samples

**Figure 5:**
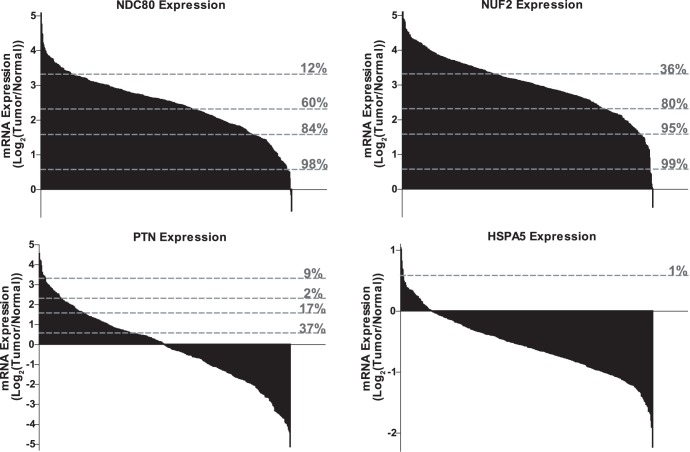
Assessment of gene expression using TCGA ovarian cancer data set. TCGA data set on 494 ovarian serous adenocarcinomas was queried to determine the mRNA expression levels (log_2_(tumor/normal ratio)) of *NDC80*, *NUF2*, *PTN,* and *HSPA5*. These data are shown as bar graphs with the grey dashed lines indicating the percentage of samples with 1.5-fold, 3-fold, 5-fold and 10-fold overexpression as compared to unmatched normal samples in the TCGA data set.

Serous adenocarcinoma is the major subtype of epithelial ovarian cancer. In order to gauge the potential clinical significance of the four validated hits, we surveyed The Cancer Genome Atlas (TCGA) ovarian serous adenocarcinoma database. [25] Gene expression data on the four validated hits from 494 serous adenocarcinomas were obtained from the TCGA portal (http://tcga-data.nci.nih.gov/tcga/tcgaHome2.jsp). Expression of *NDC80*, *NUF2*, and *PTN* is up-regulated by ≥1.5-fold in 98%, 99%, and 37% of the tumor samples, respectively (**Figure 5**); however, only 1% of the samples showed ≥1.5-fold overexpression for *HSPA5* with approximately 87% of the samples showing reduced expression relative to normal (**Figure 5**). None of these genes are mutated in more than 1% of the samples (data not shown). We next analyzed copy number variation (CNV) and DNA methylation for *NDC80*, *NUF2* and *PTN* using the TCGA database. CNV analysis demonstrated low level copy number gains (>1.2-fold) in 12%, 39% and 36% of the samples for *NDC80, NUF2 and PTN,* respectively, and a copy number loss in 41% of the samples for the *HSPA5* gene. There was a weak but statistically significant correlation between gene expression and copy number for the three genes across 494 samples (**Figure S5A**). Moreover, for these three genes we found that, on average, the samples with copy number gain exhibited a 1.6-fold increase in gene expression as compared to samples with no copy number gain (p value <0.0001, <0.0001, and <0.05 for *NDC80*, *NUF2* and *PTN,* respectively (**Figure S5B**). We analyzed whether changes in DNA methylation were also associated with aberrant expression. The promoter regions of *HSPA5*, *NDC80*, *NUF2* and *PTN* are hypomethylated (β<0.25) in ≥94% of the tumor samples. However, we did not find any statistically significant correlation between expression and promoter methylation for any of these genes in TCGA data set (data not shown).

### Validation of Clinical Significance in an Independent Cohort

**Figure 6:**
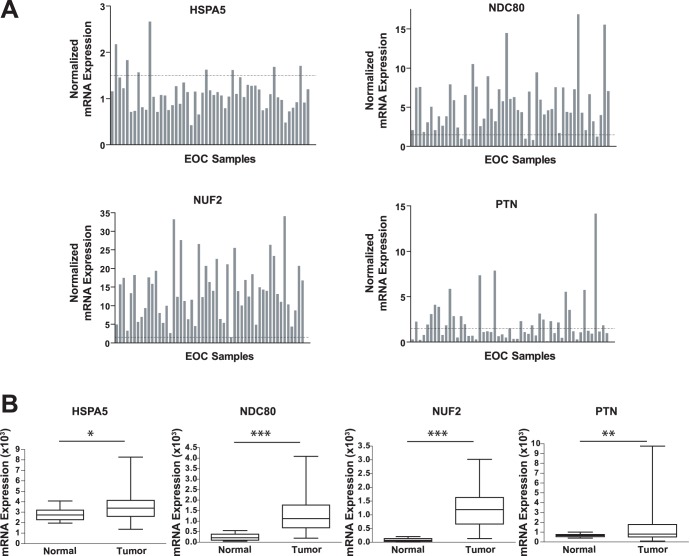
Assessment of clinical significance using an independent cohort. **A.** Shown are the gene expression levels for 53 serous adenocarcinomas normalized to the mean gene expression levels measured in normal ovarian tissue (n = 10). **B.** Shown are the mean gene expression levels across the serous adenocarcinomas for *NDC80*, *NUF2, PTN* and *HSPA5.* A two-tailed t-test indicates that the increase in gene expression in the tumor samples is statistically significant relative to the normal tissue. *** = p<0.0005; ** = p<0.005; * = p<0.05.

To further establish the potential association with pathogenesis of this disease, we examined the expression of the top four validated hits in an independent gene profiling data set of primary ovarian tumor samples. [26] Gene expression profiles of microdissected, late stage, high grade ovarian serous carcinomas (n = 53) and microdissected human ovarian surface epithelial (HOSE) samples (n = 10) were evaluated. Normalized expression levels for all the tumor samples are shown in **Figure 6A**. We found that 48/53 (90%) of the tumor samples were overexpressing *NDC80* by 1.5-fold or greater. Likewise, 53/53 samples (100%) for *NUF2* and 24/53 samples (42%) for *PTN* were found to be overexpressed by ≥1.5-fold. These data correlate well with TCGA data on expression in ovarian tumor samples. *HSPA5* was overexpressed in 8/53 (15%) of the samples. The increase in the average mRNA levels across the tumor samples relative to the normal HOSE samples was statistically significant for all four genes (p<0.005) (**Figure 6B**). We also assessed the prognostic value of these genes by performing Kaplan-Meier survival analysis of the intensity measurements from the microarray data of the four genes with the corresponding clinical data from each patient. Kaplan-Meier survival analysis for *NUF2* suggested that a high level of mRNA expression was related to poor prognosis in these patients (**Figure S6A**). Analysis for the other three genes was not statistically significant. (data not shown). A similar prognostic value of *NUF2* mRNA levels was found from survival analysis of TCGA data (**Figure S6B**).

## Discussion

The high attrition rates of drug development projects for targeted therapies [27], necessitates identification and validation of new druggable molecular targets, with their role in ovarian cancer clearly defined to minimize failure of the drug during the development pipeline. Using an integrated RNAi screening approach to target over 6,000 druggable genes we identified 53 that were required for growth and survival across a panel of EOC cell lines; seven of these were predominantly active in tumorigenic cells and were considered for additional deconvolution and validation studies. Four candidates out of the seven (*HSPA5*, *NDC80*, *NUF2*, and *PTN*) ultimately proved to be valid hits for EOC cells with minimal effects on the non-tumorigenic HIO cells.

The loss-of-function screening studies reported in this paper have provided us with a functional genomic snapshot of novel molecular vulnerabilities in epithelial ovarian cancer outside the realm of commonly targeted molecular signaling pathways. We have studied the four validated targets (*HSPA5*, *NDC80*, *NUF2* and *PTN*), all with a role in growth or survival of EOC cells, using *in vitro* cell-based assays. The results show that ovarian tumorigenic cells, on average, are comparatively more vulnerable to the candidate targets compared with non-tumorigenic cells suggesting a possible therapeutic window of sensitization. All four genes have been previously reported as hits in RNA interference screens. [28], [29], [30] All four targets code for proteins amenable to therapeutic intervention and have been previously reported to participate in cell cycle pathways or survival pathways in other tumor types. [31], [32], [33] Genomics data from the TCGA and the Birrer lab further support the notion that for at least three of the targets (*NDC80*, *NUF2*, *PTN*) there should be selective vulnerability to therapeutics in tumor cells relative to normal cells given the significant up-regulation in serous adenocarcinomas. [34] Currently, the most promising inhibitors targeting these candidates include INH11 [35] which targets the NDC80/NUF2 pathway, the neutralizing anti-PTN antibodies [36] which functionally inhibit the tumor growth promoting activities of PTN, and epigallocatechin gallate which inhibits HSPA5. [37] Additionally, siRNA-based drugs have also proven to be feasible options for *in vivo* therapy [38], [39], [40] providing us with avenues to proceed with preclinical studies to measure the effectiveness of targeting our four hits using orthotopic, xenograft mouse models of ovarian cancer.

*HSPA5* (Table 1) is a gene whose product is a central regulator for endoplasmic reticulum homeostasis which is critical for the survival of eukaryotic cells. [41] HSPA5 is a stress-inducible ER chaperone that is highly induced in a wide range of tumors through factors like hypoxia and acidosis in the microenvironment of poorly perfused tumors. [41] In a previous study, antibodies targeting cell surface HSPA5 induced apoptosis in SKOV3 cells. [42] In the current study, silencing of HSPA5 induced significant apoptosis in A2780 cells and showed a significant cell cycle arrest of A1847 cells in the G1 phase. Gene expression data from TCGA suggest that reduced expression is more common for this gene, which is counter to our screening results. CNV analysis shows that *HSPA5* is lost in 41% of the samples and mutational analysis of TCGA data shows that *HSPA5* is mutated in less than 1% of samples. Given the reduced expression, gene deletion, and lack of mutations in the tumor samples from ovarian cancer patients, additional studies are required in order to gain a better understanding of the mechanism of action and the clinical significance of this hit for ovarian cancer.

Consistent with our screening data, *NDC80* and *NUF2* are overexpressed in nearly 100% of the samples and *PTN* is overexpressed in ∼40% of the samples for two independent cohorts of patient samples. The protein products of *NDC80* (*HEC1*, *KNTC2*) and *NUF2* (*CDCA1*) are part of a mitotic complex involved in kinetochore interactions and the spindle assembly checkpoint in mitosis. [43] Mitosis dysregulation is a common cause in carcinogenesis. [44] In a previous study, siRNA mediated knockdown against *NDC80* and *NUF2* has been shown to cause abnormal mitotic exit and induce apoptosis in colorectal cancer and gastric cancer cell lines. [32] In another study silencing of *NDC80* in an EOC cell line, SKOV3.ip1, suggested that an increase in apoptosis-related cell death. [45] Both *NDC80* and *NUF2* have been shown to be up regulated in brain, liver, and breast cancer. [46] Over-expression of *NDC80* and *NUF2* has been related to poor clinical prognosis in patients with breast cancers and non-small cell lung cancers [43], [47]. Disruption of NDC80 and NUF2 complex formation using a small molecule inhibitor, INH1, has been shown to reduce proliferation in breast cancer cells and reduce tumor growth in a xenograft mouse model. [35] Kinetochore components, particularly NDC80 and NUF2, have been proposed as potential targets for cancer therapeutics. [48] Our study represents the first report on NDC80 and NUF2 as potential drug targets for treatment of ovarian cancer.

*PTN* (pleiotrophin, *HARP*) is another interesting gene identified whose product is a growth factor known to elicit downstream survival signaling pathways through multiple receptors namely ALK, SDC3, SDC1 and PTPRb/z. [49] It has been shown to play a pivotal role in tumorigenesis in pancreatic, brain and breast tumor models. [50] It is involved in cell transformation, growth, survival, migration and angiogenesis. The *PTN* gene is highly expressed during embryogenesis but shows very limited expression in adult tissues, where it is restricted to the brain. [51], [52], [53], [54] We have shown using ELISA assays that PTN levels are significantly elevated in conditioned media of the ovarian cancer cell lines examined (G. Sethi and A.K. Godwin, unpublished data). This makes it an attractive therapeutic target for ovarian cancer as anti-PTN therapeutics are expected to show high efficacy with minimal side effects on non-tumorigenic cells. Our study is the first to show that *PTN* is required for growth and survival of ovarian tumor cells.

It is now well established that both oncogenic and non-oncogenic addictions contribute to the extensively rewired pathways that underlie the malignant phenotype in cancer cells. [55] We have concentrated on genes which have activities across multiple ovarian cancer cell lines representing primarily the serous subtype. Future studies which expand our screening panel to include additional cell lines which represent other EOC subtypes (clear cell, endometrioid, and mucinous) should provide us with subtype related/specific sensitization patterns that can further be explored. In addition, we will need to establish if any or all of the validated targets have oncogenic properties, the efficacy of targeting these candidates *in vivo*, and whether targeting these candidates exhibits “genotype dependent lethality” [55] that exploits the enhanced sensitivity of cancer cells to DNA damage. As we continue to move towards better treatments for ovarian cancer patients, it will be essential to clearly define critical and functional nodes whose perturbation will lead to cancer cell lethality.

## Materials and Methods

### Cell Culture

All cell lines used in this study were obtained or derived while at the Fox Chase Cancer Center (FCCC) (Philadelphia, PA). Details of the origin of the EOC cell lines (A1847, A2780, C30, CP70, OVCAR5, OVCAR8, and SKOV3) have been previously reported [56], [57], [58]; HIO80, HIO117, and HIO120 representing non-tumorigenic human ovarian epithelial cell lines were derived by the Godwin lab and described previously [59], [60], [61], [62]. De-identified human ovarian tissue not required for diagnosis was obtained from the Biosample Repository Core Facility following approval by the Fox Chase Cancer Center (FCCC) Institutional Review Board and written informed consent. The UPN275 EOC cell line was derived and its use was approved under a protocol approved by the FCCC Institutional Review Board. All EOC and HIO cell lines were grown in RPMI 1640 (Invitrogen), supplemented with 10% FBS (Hyclone), 2 mM L-glutamine (Invitrogen), 100 IU/ml penicillin G (Invitrogen), and 100 µg/ml streptomycin (Invitrogen) and insulin 15 IU/ml (Invitrogen). The cell lines were maintained at 37°C in a humidified atmosphere with 5% CO_2_.

### High-throughput Screening

The siRNA library targeting the human druggable genome consisting of 24,088 siRNAs against 6,022 genes (siGENOME: 4 siRNAs/well/gene) was purchased from Dharmacon as a set of pre-validated siRNAs arrayed into seventy-six 96-well plates (**Table S1**). A custom library targeting 300 genes identified from the primary screen on the A1847 cell line was used for the secondary screens across the panel of tumorigenic and non-tumorigenic cell lines. This library was purchased from Qiagen as a set of 1,200 siRNAs (4 siRNAs/well/gene) arrayed into five 96-well plates (**Table S2**). siRNAs for the deconvolution screens and validation experiments were purchased from Qiagen. Positive and negative control siRNAs, *PLK1* and *GL2*, respectively, and cationic lipid transfection reagent, DharmaFECT-1, were purchased from Dharmacon. All siRNA transfections were done using the reverse transfection method. [63] Briefly, DharmaFECT-1 was diluted in reduced-serum media (OptiMEM, Invitrogen) and added to the siRNAs arrayed in v-bottom 96-well dilution plates using a bulk reagent microplate dispenser. The concentration of siRNA pools was 50 nM (12.5 nM of each individual siRNA species). The siRNA-lipid complexes were allowed to form for 30 min at room temperature. Each siRNA-lipid complex was then aliquoted equally into two 96-well flat-bottom test plates as technical replicates using a CyBio Vario liquid handler followed by addition of cells in an antibiotic-free medium using a bulk reagent microplate dispenser (∼100 µL final volume per well). Following 72 h of incubation at 37°C, cell viability was determined by using CellTiter-Blue (CTB, Promega). The CTB reagent was diluted 3-fold in phosphate-buffered saline (PBS) prior to its addition to the assay plates (20 µL per well added using a bulk reagent microplate dispenser). Fluorescence intensity was measured by using the Envision (Perkin Elmer) multi-label plate reader 3 h following addition of the CTB reagent. Data were analyzed as described below. Parameters which affect efficiency of transfection such as the dilution factor of the DharmaFECT-1 lipid reagent, the concentration of the siRNA, and the cell seeding density were optimized for each of the eleven cell lines (eight tumorigenic and three non-tumorigenic) used in the studies and are reported in **Table S3**.

### Statistical Analysis for siRNA HTS

A complete description of the statistical analysis performed on the HTS data is provided in the **Supplementary Information S1** section.

### qRT-PCR

Each of the eight cell lines was transfected (in 96-well plates) with the pool of the two most effective siRNAs (25 nM pooled concentration of the two most effective) targeting the genes of interest and the *GL2* negative control siRNA. After 72 h, total RNA was isolated using TRIzol reagent, reverse transcription was performed followed by quantitative PCR as described previously. [64] Quantification of the RT-PCR data is described in the **Supplementary Information S1** section.

### Western Blotting

Cells were transfected with siRNAs (25 nM pooled concentration of the two most effective) in 10 cm plates. Following transfection (72–96 h), cells were harvested and lysates prepared as described previously. [65] Primary antibodies specific for HSPA5 (Abcam, 1∶200), NDC80 (Abcam, 1∶1,000), NUF2 (Abcam, 1∶1,000), PTN (Abcam, 1∶250) and β-actin (Sigma, 1∶1,000) were used for immunoblotting. The blots were quantified by densitometry using the AlphaView software, version 3.3 (Cell Biosciences).

### Apoptosis and Cell Cycle Analysis

Two EOC cell lines (A1847 and A2780) were transfected with *HSPA5*, *NDC80*, *NUF2*, *PTN* and *GL2* siRNA in 96-well plates. After 72 h, cells were trypsinized and processed for apoptosis and cell cycle analysis using the Guava Nexin and Guava Cell Cycle assays, respectively, following the manufacturer’s instructions (Guava Technologies, Millipore).

### Analysis of Genomic Data Sets

The log_2_ ratios for gene expression and for copy number for each of the four genes *HSPA5*, *NDC80*, *NUF2* and *PTN* were downloaded from TCGA portal (http://tcga-data.nci.nih.gov/tcga/tcgaHome2.jsp). Samples showing expression levels of log_2_ tumor/normal ratio ≥0.584 for a particular gene were considered up-regulated. Samples showing a log_2_ tumor/normal ratio >0.3 [66] for CNV were considered to exhibit copy number gain. Beta values from the Illumina Infinium Human27-methylation assay for DNA methylation at CpG islands were also downloaded from the same TCGA portal. A mean beta value was calculated when results from two or more probes were provided for a particular gene per sample. [67] The beta values of <0.25 were considered to be hypomethylated. [68] Gene expression, copy number, and DNA methylation data were downloaded for 494 cases. The NCI dataset for gene expression in 53 ovarian serous adenocarcinoma (advanced stage, high grade) tumor samples and 10 normal human ovarian surface epithelial samples was provided by Dr. Michael Birrer. Gene expression and survival data were used for analysis as described previously [26].

### Bioinformatics and Enrichment Analysis

The PANTHER (*Protein ANalysis THrough Evolutionary Relationships*) classification system [11] was used to classify genes by their biological processes. Genes of interest were uploaded into this web-based application for analysis. Functional analysis and network generation was done using Ingenuity Pathway Analysis (IPA) software (Ingenuity Systems, www.ingenuity.com).

### Statistical Analysis

Descriptive statistics including mean and standard deviation, student’s two-tailed t-tests, and Kaplan-Meier survival analysis were computed using GraphPad Prism 5.0 software. P values <0.05 were considered significant.

## Supporting Information

Figure S1 **High-throughput siRNA screening of the human druggable genome.** **A.** Schematic showing the high-throughput screening procedure used for the primary and subsequent screens. **B.** The viability scores from the primary HTS of the siRNA library targeting 6,022 genes of the human druggable genome using the EOC cell line, A1847, display a Gaussian distribution.
(EPS)

Figure S2 **Correlation of technical replicates. A.** Eight EOC cell lines were reverse transfected with siRNAs targeting 300 genes identified from the primary screening of the A1847 cell line using transfection parameters optimized for each cell line (see **Table S3**). The measured viability from each technical replicate following silencing of a particular gene is graphed for each cell line. Spearman’s coefficient of correlation was calculated for each set of replicates. **B.** Three HIO cell lines were transfected using parameters optimized for each (see **Table S3**). See panel A for description of graphs.
(EPS)

Figure S3 **Bioinformatics analysis.** **A.** The 53 genes identified as hits across all EOC cell lines were grouped by biological process using PANTHER biological classification system. These 53 genes fell into 13 biological processes. **B.** The top 20 functions for the 53 hits as determined by the Ingenuity Pathway Analysis (IPA) software are shown. The 53 hits are enriched for genes related to protein synthesis involving ribosomal proteins and elongation factors. **C.** Analysis of hits using IPA software to perform network characterization resulted in three networks. Shown is the network with the highest score defined as the negative exponent of the p-value calculation. The red nodes represent the hits being queried and the edges connecting the nodes represent the biological relationships that are supported by the IPA knowledge base. In the network shown, genes related to protein synthesis, cell signaling and cell death are centered on well-known survival genes *ERK1/2* and *PI3* kinase complex. The red nodes represent the 53 genes, and the edges connecting the nodes represent the biological relationships that are supported by the IPA knowledge base.
(EPS)

Figure S4 **Effect of gene silencing on survival of non-tumorigenic HIO80 cells.** HIO80 cells were transfected with *HSPA5*, *NDC80*, *NUF2*, *PTN* or GL2 siRNAs. Seventy-two hours post-transfection, cells were harvested and processed for analysis of apoptosis. The fraction of apoptotic cells was measured by annexin V staining followed by enumeration by using a Guava flow cytometer (Millipore). The fold-change in apoptotic cells is shown (mean ± SD, n = 2).
(EPS)

Figure S5 **Correlation of gene expression to CNV.** **A.** Spearman correlation analysis was performed to correlate copy number variation to gene expression for *NDC80*, *NUF2*, and *PTN* across 494 samples. The correlation coefficient is shown for each analysis along with a probability value to measure statistical significance. **B.** Box plots showing differences in gene expression for *NDC80, NUF2* and *PTN* in samples with or without copy number gain. The threshold for copy number gain was set at a ratio >1.2 (log_2_ tumor/normal ratio >0.3. A two-tailed t-test indicates that the increase in gene expression with copy number gain is statistically significant relative to no copy number gain. *** = p<0.0005; ** = p<0.005.
(EPS)

Figure S6 **Survival analysis.** Kaplan-Meier survival analysis was performed for patients with below-median (blue line) or above-median (red line) *NUF2* mRNA expression using (**A**) the data set from the NCI provided by Dr. Michael Birrer and (**B**) TCGA data set.
(EPS)

Table S1 List of 6,022 genes targeted by this siRNA library (Human Druggable Set G-004600, Dharmacon). Each well contains a pool of 4 siRNA duplexes targeting the indicated gene. The siRNA pools are arrayed into seventy-six 96-well plates.
(PDF)

Table S2 List of 300 genes targeted by this custom siRNA library purchased from Qiagen. Each well contains a pool of 4 siRNA duplexes targeting the indicated gene. The siRNA pools are arrayed into five 96-well plates.
(PDF)

Table S3 List of the eight epithelial ovarian cancer (EOC) tumorigenic and the three human immortalized ovarian surface epithelial (HIO) non-tumorigenic cell lines used in this study. The EOC cell lines have been selected to represent epithelial serous histotype, which is the major subtype of ovarian cancer. The transfection conditions were optimized for cell seeding density per well, dilution of the lipid-based transfection reagent, and the final siRNA concentration for each of the cell lines used in the study. The following ranges for each parameter were evaluated during optimization: cell densities (6.5×10^3^–1×10^4^ per well); lipid dilution (1∶250–1∶1000); siRNA concentration (50 nM–100 nM).
(DOC)

Table S4 Average viability scores and viability indices across the eight tumorigenic and three non-tumorigenic cell lines following HTS of the 300 gene custom library. Genes highlighted in yellow were hits unique to the EOC cell lines and selected for further validation. *NUF2* (highlighted in green) had the lowest Viability Index score and was also selected for further validation.
(XLS)

Table S5 List of 28 siRNAs used in the deconvolution screen purchased from Qiagen. Each well contains a single siRNA duplex targeting the indicated gene. The siRNAs are arrayed into a single 96-well plate. The two most effective siRNAs are highlighted in green.
(XLS)

Supplementary Information S1 (DOC)
