# Supplementary material for: An RNA Interference Lethality Screen of the Human Druggable Genome to Identify Molecular Vulnerabilities in Epithelial Ovarian Cancer
Source: PLoS One. 2012 Oct 9;7(10):e47086. doi: 10.1371/journal.pone.0047086 (PMC3467214; doi:10.1371/journal.pone.0047086)
Supplement: Table S1 — List of 6,022 genes targeted by this siRNA library (Human Druggable Set G-004600, Dharmacon). Each well contains a pool of 4 siRNA duplexes targeting the indicated gene. The siRNA pools are arrayed into seventy-six 96-well plates. (PDF) [file pone.0047086.s007.pdf]

## Supplementary Table S1

**List of 6,022 genes targeted by this siRNA library purchased from Dharmacon (Human Druggable Set G-004600).**
**Each well contains a pool of 4 siRNA duplexes targeting the indicated gene. The siRNA pools are arrayed into seventy-six 96-well plates.**

| PLATE | WELL | LOCUS ID | NCBI GENE SYMBOL | mRNA ACCESSION # | RAW FLUORESCENCE READING 1 | RAW FLUORESCENCE READING 2 | NORMALIZED VIABILITY 1 | NORMALIZED VIABILITY 2 | B SCORE 1 | B SCORE 2 |
|---|---|---|---|---|---|---|---|---|---|---|
| 1 | A01 | NA | pos | NA | 58009 | 81723 | 0.35 | 0.42 | 25.97 | 15.97 |
| 1 | A02 | NA | NA | NA | 182620 | 184579 | 1.09 | 0.95 | -1.17 | -2.15 |
| 1 | A03 | 55752 | SEPT11 | NM_018243 | 178408 | 178170 | 1.07 | 0.92 | -3.27 | -0.22 |
| 1 | A04 | 14 | AAMP | NM_001087 | 127939 | 153000 | 0.76 | 0.79 | 7.33 | 2.31 |
| 1 | A05 | 5244 | ABCB4 | NM_000443 | 207697 | 187075 | 1.24 | 0.96 | 1.33 | -0.87 |
| 1 | A06 | 89845 | ABCC10 | NM_033450 | 208945 | 175461 | 1.25 | 0.90 | 0.18 | 0.17 |
| 1 | A07 | 5825 | ABCD3 | NM_002858 | 197160 | 176067 | 1.18 | 0.91 | -0.11 | -0.56 |
| 1 | A08 | 64240 | ABCG5 | NM_022436 | 188650 | 166268 | 1.13 | 0.86 | -2.03 | 0.18 |
| 1 | A09 | 64241 | ABCG8 | NM_022437 | 178579 | 182553 | 1.07 | 0.94 | -0.24 | -2.47 |
| 1 | A10 | 80325 | ABTB1 | NM_172027 | 168561 | 166405 | 1.01 | 0.86 | 1.22 | 0.97 |
| 1 | A11 | 98 | ACYP2 | NM_138448 | 185269 | 171893 | 1.11 | 0.89 | -1.86 | -0.16 |
| 1 | A12 | 119 | ADD2 | NM_001617 | 175355 | 167517 | 1.05 | 0.86 | 0.05 | 1.06 |
| 1 | B01 | NA | neg | NA | 155071 | 201591 | 0.93 | 1.04 | 6.48 | -4.63 |
| 1 | B02 | NA | neg | NA | 191977 | 198941 | 1.15 | 1.03 | -1.56 | -4.16 |
| 1 | B03 | 51517 | NCKIPSD | NM_016453 | 170964 | 181226 | 1.02 | 0.93 | -0.01 | -0.25 |
| 1 | B04 | 176 | AGC1 | NM_001135 | 193887 | 182016 | 1.16 | 0.94 | -5.39 | -2.29 |
| 1 | B05 | 199 | AIF1 | NM_001623 | 221869 | 177767 | 1.32 | 0.92 | -0.11 | 1.28 |
| 1 | B06 | 9447 | AIM2 | NM_004833 | 196549 | 178934 | 1.17 | 0.92 | 4.53 | 0.07 |
| 1 | B07 | 11217 | AKAP2 | NM_007203 | 188774 | 164163 | 1.13 | 0.85 | 3.36 | 2.05 |
| 1 | B08 | 27295 | PDLIM3 | NM_014476 | 186872 | 168946 | 1.12 | 0.87 | 0.01 | 0.22 |
| 1 | B09 | 117583 | ALS2CR19 | NM_152526 | 187067 | 171519 | 1.12 | 0.88 | -0.44 | -0.01 |
| 1 | B10 | 27063 | ANKRD1 | NM_014391 | 166037 | 174708 | 0.99 | 0.90 | 3.41 | 0.02 |
| 1 | B11 | 63926 | ANKRD5 | NM_022096 | 176917 | 187968 | 1.06 | 0.97 | 1.61 | -2.48 |
| 1 | B12 | 11199 | ANXA10 | NM_007193 | 183388 | 177204 | 1.09 | 0.91 | -0.05 | -0.13 |
| 1 | C01 | NA | pos | NA | 59501 | 78460 | 0.36 | 0.40 | 28.03 | 16.68 |
| 1 | C02 | NA | NA | NA | 222255 | 187036 | 1.33 | 0.96 | -7.41 | -2.44 |
| 1 | C03 | 311 | ANXA11 | NM_001157 | 172414 | 172210 | 1.03 | 0.89 | 0.42 | 0.96 |
| 1 | C04 | 312 | ANXA13 | NM_004306 | 175349 | 163474 | 1.05 | 0.84 | -0.61 | 0.60 |
| 1 | C05 | 322 | APBB1 | NM_001164 | 227395 | 183221 | 1.36 | 0.94 | -0.57 | -0.06 |
| 1 | C06 | 10307 | APBB3 | NM_006051 | 226261 | 203978 | 1.35 | 1.05 | -1.20 | -4.71 |
| 1 | C07 | 324 | APC | NM_000038 | 206756 | 167825 | 1.23 | 0.87 | 0.19 | 1.03 |
| 1 | C08 | 10297 | APC2 | NM_005883 | 174203 | 175180 | 1.04 | 0.90 | 3.51 | -1.25 |
| 1 | C09 | 325 | APCS | NM_001639 | 187372 | 170606 | 1.12 | 0.88 | 0.24 | -0.22 |
| 1 | C10 | 22824 | APG-1 | NM_014278 | 190362 | 162471 | 1.14 | 0.84 | -1.14 | 1.80 |
| 1 | C11 | 333 | APLP1 | NM_005166 | 187217 | 181446 | 1.12 | 0.94 | 0.11 | -1.71 |
| 1 | C12 | 80832 | APOL4 | NM_030643 | 187298 | 174000 | 1.12 | 0.90 | -0.16 | 0.06 |
| 1 | D01 | NA | neg | NA | 138246 | 179881 | 0.83 | 0.93 | 11.02 | 1.06 |
| 1 | D02 | NA | neg | NA | 220912 | 190435 | 1.32 | 0.98 | -6.98 | -0.80 |
| 1 | D03 | 23047 | APRIN | NM_015032 | 215984 | 194719 | 1.29 | 1.00 | -8.93 | -0.76 |
| 1 | D04 | 23647 | ARFIP2 | NM_012402 | 171544 | 184245 | 1.02 | 0.95 | 0.35 | -0.82 |
| 1 | D05 | 9828 | ARHGEF17 | NM_014786 | 233896 | 199024 | 1.40 | 1.03 | -1.85 | -0.60 |
| 1 | D06 | 7984 | ARHGEF5 | NM_005435 | 222211 | 189399 | 1.33 | 0.98 | -0.18 | 0.09 |
| 1 | D07 | 58480 | RHOU | NM_021205 | 201288 | 186278 | 1.20 | 0.96 | 1.51 | 0.01 |
| 1 | D08 | 55729 | ATF7IP | NM_018179 | 188237 | 174146 | 1.12 | 0.90 | 0.58 | 1.17 |
| 1 | D09 | 57099 | AVEN | NM_020371 | 194081 | 164502 | 1.16 | 0.85 | -1.09 | 3.09 |
| 1 | D10 | 60468 | BACH2 | NM_021813 | 184804 | 185475 | 1.10 | 0.96 | 0.20 | -0.02 |
| 1 | D11 | 572 | BAD | NM_004322 | 188820 | 176058 | 1.13 | 0.91 | -0.11 | 1.48 |
| 1 | D12 | 9532 | BAG2 | NM_004282 | 186899 | 187204 | 1.12 | 0.97 | 0.06 | -0.03 |
| 1 | E01 | NA | neg | NA | 144472 | 200641 | 0.86 | 1.03 | 9.94 | -2.46 |
| 1 | E02 | NA | neg | NA | 200926 | 185355 | 1.20 | 0.96 | -2.35 | 0.23 |
| 1 | E03 | 9531 | BAG3 | NM_004281 | 168964 | 190703 | 1.01 | 0.98 | 1.58 | 0.08 |
| 1 | E04 | 9529 | BAG5 | NM_004873 | 153272 | 175149 | 0.92 | 0.90 | 4.61 | 0.92 |
| 1 | E05 | 9223 | BAIAP1 | NM_004742 | 207487 | 187018 | 1.24 | 0.96 | 4.18 | 1.65 |
| 1 | E06 | 8938 | BAIAP3 | NM_003933 | 225433 | 191135 | 1.35 | 0.99 | -0.61 | -0.08 |
| 1 | E07 | 10286 | BCAS2 | NM_005872 | 213174 | 188524 | 1.27 | 0.97 | -0.80 | -0.24 |
| 1 | E08 | 53335 | BCL11A | NM_018014 | 194030 | 182149 | 1.16 | 0.94 | -0.40 | -0.10 |
| 1 | E09 | 596 | BCL2 | NM_000633 | 184951 | 167953 | 1.10 | 0.87 | 1.17 | 2.62 |
| 1 | E10 | 255877 | BCL6B | NM_181844 | 192713 | 198355 | 1.15 | 1.02 | -1.24 | -2.15 |
| 1 | E11 | 605 | BCL7A | NM_020993 | 187901 | 173112 | 1.12 | 0.89 | 0.37 | 2.13 |
| 1 | E12 | 9275 | BCL7B | NM_001707 | 191237 | 191252 | 1.14 | 0.99 | -0.61 | -0.61 |
| 1 | F01 | NA | NA | NA | 130675 | 195558 | 0.78 | 1.01 | 10.56 | -2.23 |
| 1 | F02 | NA | pos | NA | 79306 | 76298 | 0.47 | 0.39 | 21.74 | 18.77 |
| 1 | F03 | 617 | BCS1L | NM_004328 | 165789 | 182262 | 0.99 | 0.94 | -0.11 | 0.90 |
| 1 | F04 | 55814 | BDP1 | NM_018429 | 160453 | 170482 | 0.96 | 0.88 | 0.66 | 1.08 |
| 1 | F05 | 79444 | BIRC7 | NM_022161 | 215222 | 182667 | 1.28 | 0.94 | 0.11 | 1.75 |
| 1 | F06 | 664 | BNIP3 | NM_004052 | 209767 | 187344 | 1.25 | 0.97 | 0.42 | -0.07 |
| 1 | F07 | 7862 | BRPF1 | NM_004634 | 199914 | 183449 | 1.19 | 0.95 | -0.30 | -0.01 |
| 1 | F08 | 112939 | BTBD14B | NM_052876 | 178078 | 185841 | 1.06 | 0.96 | 0.69 | -1.42 |
| 1 | F09 | 9478 | CABP1 | NM_004276 | 180975 | 178991 | 1.08 | 0.92 | -0.35 | 0.01 |
| 1 | F10 | 805 | CALM2 | NM_001743 | 182039 | 183034 | 1.09 | 0.94 | -1.30 | -0.11 |
| 1 | F11 | 51806 | CALML5 | NM_017422 | 183128 | 182964 | 1.09 | 0.94 | -0.98 | -0.26 |
| 1 | F12 | 821 | CANX | NM_001746 | 168697 | 154850 | 1.01 | 0.80 | 1.91 | 5.14 |
| 1 | G01 | NA | neg | NA | 139256 | 197436 | 0.83 | 1.02 | 9.80 | -2.08 |
| 1 | G02 | NA | neg | NA | 179936 | 186832 | 1.07 | 0.96 | 0.94 | -0.21 |
| 1 | G03 | 822 | CAPG | NM_001747 | 170354 | 178376 | 1.02 | 0.92 | 0.01 | 2.07 |
| 1 | G04 | 828 | CAPS | NM_004058 | 170212 | 182744 | 1.02 | 0.94 | -0.35 | -0.60 |
| 1 | G05 | 829 | CAPZA1 | NM_006135 | 218889 | 197128 | 1.31 | 1.02 | 0.42 | -0.31 |
| 1 | G06 | 58484 | CARD12 | NM_021209 | 204384 | 187878 | 1.22 | 0.97 | 2.70 | 0.32 |
| 1 | G07 | 64127 | CARD15 | NM_022162 | 205547 | 195325 | 1.23 | 1.01 | -0.41 | -1.62 |
| 1 | G08 | 84674 | CARD6 | NM_032587 | 186362 | 184654 | 1.11 | 0.95 | -0.01 | -0.72 |

| | | | | | | | | | | |
|---|---|---|---|---|---|---|---|---|---|---|
| 1 | G09 | 844 | CASQ1 | NM_001231 | 177766 | 162655 | 1.06 | 0.84 | 1.46 | 3.37 |
| 1 | G10 | 845 | CASQ2 | NM_001232 | 178692 | 175156 | 1.07 | 0.90 | 0.54 | 1.76 |
| 1 | G11 | 11335 | CBX3 | NM_007276 | 188990 | 177653 | 1.13 | 0.92 | -1.14 | 1.15 |
| 1 | G12 | 23468 | CBX5 | NM_012117 | 193309 | 194643 | 1.15 | 1.00 | -2.33 | -1.39 |
| 1 | H01 | NA | NA | NA | 152215 | 210512 | 0.91 | 1.09 | 6.49 | -5.34 |
| 1 | H02 | NA | pos | NA | 75319 | 76832 | 0.45 | 0.40 | 23.23 | 18.20 |
| 1 | H03 | 23466 | CBX6 | NM_014292 | 166806 | 185135 | 1.00 | 0.95 | 0.29 | -0.08 |
| 1 | H04 | 57332 | CBX8 | NM_020649 | 205852 | 188588 | 1.23 | 0.97 | -8.60 | -2.59 |
| 1 | H05 | 10576 | CCT2 | NM_006431 | 222321 | 189564 | 1.33 | 0.98 | -0.82 | 0.06 |
| 1 | H06 | 7203 | CCT3 | NM_005998 | 215888 | 187621 | 1.29 | 0.97 | -0.29 | -0.60 |
| 1 | H07 | 22948 | CCT5 | NM_012073 | 200878 | 175662 | 1.20 | 0.91 | 0.11 | 0.88 |
| 1 | H08 | 908 | CCT6A | NM_001762 | 184930 | 174506 | 1.10 | 0.90 | -0.18 | 0.10 |
| 1 | H09 | 10574 | CCT7 | NM_006429 | 171829 | 179996 | 1.03 | 0.93 | 2.27 | -0.64 |
| 1 | H10 | 977 | CD151 | NM_004357 | 179829 | 179858 | 1.07 | 0.93 | -0.20 | -0.03 |
| 1 | H11 | 933 | CD22 | NM_001771 | 178145 | 177811 | 1.06 | 0.92 | 0.73 | 0.16 |
| 1 | H12 | 947 | CD34 | NM_001773 | 176731 | 181130 | 1.06 | 0.93 | 0.79 | 0.03 |
| 2 | A01 | NA | pos | NA | 70973 | 76856 | 0.37 | 0.37 | 12.43 | 13.56 |
| 2 | A02 | NA | NA | NA | 177073 | 182953 | 0.91 | 0.89 | -0.21 | -0.66 |
| 2 | A03 | 961 | CD47 | NM_001777 | 165976 | 183598 | 0.86 | 0.89 | 1.44 | -0.04 |
| 2 | A04 | 962 | CD48 | NM_001778 | 186708 | 187183 | 0.96 | 0.91 | -1.60 | -1.77 |
| 2 | A05 | 965 | CD58 | NM_001779 | 179846 | 187499 | 0.93 | 0.91 | 0.22 | -0.20 |
| 2 | A06 | 975 | CD81 | NM_004356 | 168439 | 184289 | 0.87 | 0.89 | 1.15 | 0.40 |
| 2 | A07 | 928 | CD9 | NM_001769 | 177149 | 184039 | 0.91 | 0.89 | -0.14 | 0.00 |
| 2 | A08 | 11140 | CDC37 | NM_007065 | 175071 | 174729 | 0.90 | 0.85 | -1.01 | 0.08 |
| 2 | A09 | 10602 | CDC42EP3 | NM_006449 | 175332 | 175444 | 0.90 | 0.85 | 0.03 | -0.23 |
| 2 | A10 | 988 | CDC5L | NM_001253 | 180901 | 169329 | 0.93 | 0.82 | -1.09 | 0.93 |
| 2 | A11 | 990 | CDC6 | NM_001254 | 161131 | 166013 | 0.83 | 0.80 | 1.66 | 0.93 |
| 2 | A12 | 999 | CDH1 | NM_004360 | 173264 | 179801 | 0.89 | 0.87 | -0.03 | 0.00 |
| 2 | B01 | NA | neg | NA | 208475 | 226704 | 1.08 | 1.10 | -3.17 | -6.04 |
| 2 | B02 | NA | neg | NA | 187581 | 196100 | 0.97 | 0.95 | -0.68 | -1.94 |
| 2 | B03 | 1008 | CDH10 | NM_006727 | 187100 | 203927 | 0.97 | 0.99 | -0.30 | -2.28 |
| 2 | B04 | 1009 | CDH11 | NM_001797 | 180728 | 176592 | 0.93 | 0.85 | -0.11 | 0.13 |
| 2 | B05 | 1010 | CDH12 | NM_004061 | 171033 | 187949 | 0.88 | 0.91 | 2.05 | 0.23 |
| 2 | B06 | 1012 | CDH13 | NM_001257 | 172290 | 185742 | 0.89 | 0.90 | 1.47 | 0.69 |
| 2 | B07 | 1013 | CDH15 | NM_004933 | 182628 | 192943 | 0.94 | 0.93 | -0.02 | -0.71 |
| 2 | B08 | 1014 | CDH16 | NM_004062 | 165888 | 173910 | 0.86 | 0.84 | 0.86 | 0.68 |
| 2 | B09 | 1016 | CDH18 | NM_004934 | 186648 | 190179 | 0.96 | 0.92 | -0.54 | -1.72 |
| 2 | B10 | 28513 | CDH19 | NM_021153 | 174598 | 180855 | 0.90 | 0.88 | 0.44 | -0.13 |
| 2 | B11 | 1000 | CDH2 | NM_001792 | 181463 | 172846 | 0.94 | 0.84 | 0.02 | 0.50 |
| 2 | B12 | 28316 | CDH20 | NM_031891 | 180712 | 186872 | 0.93 | 0.90 | -0.14 | -0.46 |
| 2 | C01 | NA | pos | NA | 88818 | 91867 | 0.46 | 0.44 | 11.12 | 12.28 |
| 2 | C02 | NA | NA | NA | 188690 | 193368 | 0.97 | 0.94 | -0.77 | -1.32 |
| 2 | C03 | 64072 | CDH23 | NM_022124 | 170569 | 177298 | 0.88 | 0.86 | 1.71 | 1.54 |
| 2 | C04 | 1002 | CDH4 | NM_001794 | 167929 | 179443 | 0.87 | 0.87 | 1.46 | 0.00 |
| 2 | C05 | 1003 | CDH5 | NM_001795 | NA | NA | NA | NA | NA | NA |
| 2 | C06 | 1004 | CDH6 | NM_004932 | 179741 | 195705 | 0.93 | 0.95 | 0.62 | -0.40 |
| 2 | C07 | 1005 | CDH7 | NM_004361 | 163790 | 173974 | 0.84 | 0.84 | 2.27 | 2.08 |
| 2 | C08 | 1006 | CDH8 | NM_001796 | 174759 | 170832 | 0.90 | 0.83 | -0.15 | 1.34 |
| 2 | C09 | 1007 | CDH9 | NM_016279 | 182454 | 179222 | 0.94 | 0.87 | 0.00 | 0.00 |
| 2 | C10 | 55755 | CDK5RAP2 | NM_018249 | 187353 | 195680 | 0.97 | 0.95 | -1.03 | -1.87 |
| 2 | C11 | 9085 | CDY1 | NM_004680 | 182582 | 189856 | 0.94 | 0.92 | -0.07 | -1.53 |
| 2 | C12 | 9426 | CDY2 | NM_004825 | 180980 | 186617 | 0.93 | 0.90 | -0.13 | -0.18 |
| 2 | D01 | NA | neg | NA | 196364 | 211970 | 1.01 | 1.03 | -1.24 | -3.09 |
| 2 | D02 | NA | neg | NA | 192027 | 195177 | 0.99 | 0.94 | -0.72 | -0.84 |
| 2 | D03 | 1054 | CEBPG | NM_001806 | 204118 | 193867 | 1.05 | 0.94 | -1.83 | 0.04 |
| 2 | D04 | 1058 | CENPA | NM_001809 | 183001 | 171224 | 0.94 | 0.83 | 0.11 | 1.83 |
| 2 | D05 | 1059 | CENPB | NM_001810 | 205685 | 206625 | 1.06 | 1.00 | -1.58 | -1.30 |
| 2 | D06 | 1060 | CENPC1 | NM_001812 | 189687 | 198757 | 0.98 | 0.96 | -0.11 | -0.08 |
| 2 | D07 | 11190 | CEP2 | NM_007186 | 175004 | 194941 | 0.90 | 0.94 | 1.38 | 0.00 |
| 2 | D08 | 1068 | CETN1 | NM_004066 | 175350 | 186858 | 0.90 | 0.90 | 0.23 | -0.08 |
| 2 | D09 | 1070 | CETN3 | NM_004365 | 201657 | 184598 | 1.04 | 0.89 | -1.84 | 0.00 |
| 2 | D10 | 1072 | CFL1 | NM_005507 | 154297 | 187122 | 0.80 | 0.91 | 3.35 | 0.00 |
| 2 | D11 | 8545 | CGGBP1 | NM_003663 | 190829 | 200168 | 0.98 | 0.97 | -0.61 | -2.19 |
| 2 | D12 | 10669 | CGREF1 | NM_006569 | 182733 | 190536 | 0.94 | 0.92 | 0.11 | 0.02 |
| 2 | E01 | NA | neg | NA | 194044 | 234165 | 1.00 | 1.13 | -1.18 | -6.52 |
| 2 | E02 | NA | neg | NA | 186606 | 201137 | 0.96 | 0.97 | -0.30 | -2.10 |
| 2 | E03 | 10036 | CHAF1A | NM_005483 | 179580 | 184556 | 0.93 | 0.89 | 0.87 | 0.83 |
| 2 | E04 | 1113 | CHGA | NM_001275 | 175802 | 181428 | 0.91 | 0.88 | 0.74 | 0.00 |
| 2 | E05 | 1149 | CIDEA | NM_001279 | 171285 | NA | 0.88 | NA | 2.29 | NA |
| 2 | E06 | 27141 | CIDEB | NM_014430 | 194867 | 204668 | 1.01 | 0.99 | -0.95 | -1.33 |
| 2 | E07 | 1153 | CIRBP | NM_001280 | 196806 | 199862 | 1.02 | 0.97 | -1.44 | -1.12 |
| 2 | E08 | 25792 | CIZ1 | NM_012127 | 172961 | 164531 | 0.89 | 0.80 | 0.29 | 2.45 |
| 2 | E09 | 23332 | CLASP1 | NM_015282 | 164565 | NA | 0.85 | NA | 2.36 | NA |
| 2 | E10 | 1047 | CLGN | NM_004362 | 189931 | 183741 | 0.98 | 0.89 | -1.12 | 0.00 |
| 2 | E11 | 63967 | CLSPN | NM_022111 | 186279 | 179824 | 0.96 | 0.87 | -0.29 | 0.08 |
| 2 | E12 | 64084 | CLSTN2 | NM_022131 | 189351 | 190506 | 0.98 | 0.92 | -0.90 | -0.43 |
| 2 | F01 | NA | NA | NA | 203032 | 216699 | 1.05 | 1.05 | -2.19 | -4.94 |
| 2 | F02 | NA | pos | NA | 77608 | 75922 | 0.40 | 0.37 | 12.75 | 13.93 |
| 2 | F03 | 9746 | CLSTN3 | NM_014718 | 197202 | 186712 | 1.02 | 0.90 | -1.16 | -0.21 |
| 2 | F04 | 1211 | CLTA | NM_001833 | 175510 | 175811 | 0.91 | 0.85 | 0.85 | 0.00 |
| 2 | F05 | 1212 | CLTB | NM_001834 | 191066 | 201767 | 0.99 | 0.98 | 0.00 | -1.86 |
| 2 | F06 | 1272 | CNTN1 | NM_001843 | 186513 | 179950 | 0.96 | 0.87 | 0.11 | 1.22 |
| 2 | F07 | 6900 | CNTN2 | NM_005076 | 185144 | 181883 | 0.96 | 0.88 | 0.02 | 0.54 |
| 2 | F08 | 23603 | CORO1C | NM_014325 | 194102 | 188557 | 1.00 | 0.91 | -2.16 | -1.52 |
| 2 | F09 | 7464 | CORO2A | NM_003389 | 184916 | 170431 | 0.95 | 0.83 | 0.00 | 0.69 |
| 2 | F10 | 10391 | CORO2B | NM_006091 | 181129 | 177838 | 0.93 | 0.86 | 0.00 | 0.03 |
| 2 | F11 | 8738 | CRADD | NM_003805 | 184564 | 182303 | 0.95 | 0.88 | -0.02 | -1.00 |
| 2 | F12 | 1388 | CREBL1 | NM_004381 | 182154 | NA | 0.94 | NA | 0.03 | NA |

| | | | | | | | | | | |
|---|---|---|---|---|---|---|---|---|---|---|
| 2 | G01 | NA | neg | NA | 201320 | 219447 | 1.04 | 1.06 | -1.67 | -4.89 |
| 2 | G02 | NA | neg | NA | 193690 | 195998 | 1.00 | 0.95 | -0.76 | -1.74 |
| 2 | G03 | 1393 | CRHBP | NM_001882 | 191306 | 200071 | 0.99 | 0.97 | -0.15 | -1.58 |
| 2 | G04 | 1462 | CSPG2 | NM_004385 | 191092 | 204212 | 0.99 | 0.99 | -0.70 | -3.39 |
| 2 | G05 | 1479 | CSTF3 | NM_001326 | 198574 | 181672 | 1.02 | 0.88 | -0.58 | 1.25 |
| 2 | G06 | 1490 | CTGF | NM_001901 | 201440 | 191576 | 1.04 | 0.93 | -1.35 | 0.08 |
| 2 | G07 | 56998 | CTNNBIP1 | NM_020248 | 198821 | 192289 | 1.03 | 0.93 | -1.30 | -0.44 |
| 2 | G08 | 10659 | CUGBP2 | NM_006561 | 177313 | 194879 | 0.91 | 0.94 | 0.15 | -1.95 |
| 2 | G09 | 1535 | CYBA | NM_000101 | 179094 | 174532 | 0.92 | 0.84 | 1.01 | 0.56 |
| 2 | G10 | 3491 | CYR61 | NM_001554 | 162972 | 169276 | 0.84 | 0.82 | 2.47 | 1.60 |
| 2 | G11 | 7812 | D1S155E | NM_007158 | 175849 | 178568 | 0.91 | 0.86 | 1.33 | -0.08 |
| 2 | G12 | 23500 | DAAM2 | NM_015345 | 172224 | 175315 | 0.89 | 0.85 | 1.52 | 1.27 |
| 2 | H01 | NA | NA | NA | 182493 | 216300 | 0.94 | 1.05 | -0.57 | -4.03 |
| 2 | H02 | NA | pos | NA | 76765 | 87814 | 0.40 | 0.43 | 12.03 | 13.19 |
| 2 | H03 | 1602 | DACH1 | NM_004392 | 179231 | 183849 | 0.92 | 0.89 | 0.15 | 1.03 |
| 2 | H04 | 117154 | DACH2 | NM_053281 | 194780 | 194445 | 1.00 | 0.94 | -2.27 | -1.64 |
| 2 | H05 | 11083 | DATF1 | NM_022105 | 195078 | 192751 | 1.01 | 0.93 | -1.30 | 0.20 |
| 2 | H06 | 1616 | DAXX | NM_001350 | 192660 | 196927 | 0.99 | 0.95 | -1.45 | -0.20 |
| 2 | H07 | 1617 | DAZ | NM_004081 | 174557 | 185709 | 0.90 | 0.90 | 0.45 | 0.88 |
| 2 | H08 | 1643 | DDB2 | NM_000107 | 183935 | 191879 | 0.95 | 0.93 | -1.78 | -1.11 |
| 2 | H09 | 9191 | DEDD | NM_004216 | NA | 188980 | NA | 0.91 | NA | -0.94 |
| 2 | H10 | 148252 | DIRAS1 | NM_145173 | 174194 | 189948 | 0.90 | 0.92 | 0.00 | -0.73 |
| 2 | H11 | 8788 | DLK1 | NM_003836 | 169187 | 177629 | 0.87 | 0.86 | 0.99 | 0.48 |
| 2 | H12 | 28514 | DLL1 | NM_005618 | 162429 | 166804 | 0.84 | 0.81 | 1.55 | 2.84 |
| 3 | A01 | NA | pos | NA | 77213 | 78749 | 0.39 | 0.37 | 18.64 | 11.48 |
| 3 | A02 | NA | NA | NA | 177815 | 178867 | 0.91 | 0.83 | -0.50 | -0.93 |
| 3 | A03 | 8701 | DNAH11 | NM_003777 | 175273 | 161561 | 0.89 | 0.75 | 1.61 | 3.16 |
| 3 | A04 | 1767 | DNAH5 | NM_001369 | 184834 | 180972 | 0.94 | 0.84 | -2.13 | -0.73 |
| 3 | A05 | 83658 | DNCL2A | NM_014183 | 183629 | 187106 | 0.93 | 0.87 | -0.04 | -0.83 |
| 3 | A06 | 83657 | DNCL2B | NM_130897 | 187089 | 184641 | 0.95 | 0.86 | -1.17 | -1.29 |
| 3 | A07 | 1783 | DNCLI2 | NM_006141 | 162878 | 167295 | 0.83 | 0.78 | 1.99 | 0.18 |
| 3 | A08 | 4733 | DRG1 | NM_004147 | 168338 | 168790 | 0.86 | 0.79 | 0.04 | -0.57 |
| 3 | A09 | 1819 | DRG2 | NM_001388 | 171117 | 166350 | 0.87 | 0.77 | -0.09 | 0.11 |
| 3 | A10 | 10620 | ARID3B | NM_006465 | 168123 | 164108 | 0.86 | 0.76 | 1.66 | -0.11 |
| 3 | A11 | 1822 | DRPLA | NM_001940 | 173868 | 167179 | 0.89 | 0.78 | 1.30 | 0.85 |
| 3 | A12 | 1823 | DSC1 | NM_004948 | 181362 | 157955 | 0.92 | 0.74 | -1.61 | 0.96 |
| 3 | B01 | NA | neg | NA | 199872 | 233333 | 1.02 | 1.09 | -4.76 | -7.43 |
| 3 | B02 | NA | neg | NA | 194644 | 199538 | 0.99 | 0.93 | -3.76 | -3.24 |
| 3 | B03 | 1824 | DSC2 | NM_004949 | 192175 | 189894 | 0.98 | 0.88 | -1.66 | -0.11 |
| 3 | B04 | 1825 | DSC3 | NM_001941 | 169921 | 190464 | 0.87 | 0.89 | 0.65 | -1.66 |
| 3 | B05 | 1828 | DSG1 | NM_001942 | 179756 | 195473 | 0.92 | 0.91 | 0.63 | -1.62 |
| 3 | B06 | 1829 | DSG2 | NM_001943 | 174735 | 189630 | 0.89 | 0.88 | 1.12 | -1.66 |
| 3 | B07 | 1830 | DSG3 | NM_001944 | 169811 | 168212 | 0.86 | 0.78 | 0.61 | 0.32 |
| 3 | B08 | 1837 | DTNA | NM_001390 | 171161 | 151578 | 0.87 | 0.71 | -0.56 | 1.81 |
| 3 | B09 | 11100 | HNRPUL1 | NM_007040 | 190163 | 161044 | 0.97 | 0.75 | -3.77 | 1.01 |
| 3 | B10 | 2202 | EFEMP1 | NM_004105 | 178324 | 164330 | 0.91 | 0.76 | -0.35 | 0.11 |
| 3 | B11 | 10278 | EFS | NM_005864 | 180858 | 174324 | 0.92 | 0.81 | -0.09 | 0.21 |
| 3 | B12 | 1959 | EGR2 | NM_000399 | 172043 | 169320 | 0.88 | 0.79 | 0.11 | -0.21 |
| 3 | C01 | NA | pos | NA | 75115 | 88746 | 0.38 | 0.41 | 19.85 | 10.93 |
| 3 | C02 | NA | NA | NA | 177854 | 183528 | 0.91 | 0.85 | 0.30 | -0.82 |
| 3 | C03 | 1978 | EIF4EBP1 | NM_004095 | 166776 | 192598 | 0.85 | 0.90 | 4.04 | 0.00 |
| 3 | C04 | 1979 | EIF4EBP2 | NM_004096 | 163247 | 175232 | 0.83 | 0.82 | 2.79 | 0.67 |
| 3 | C05 | 1994 | ELAVL1 | NM_001419 | 196752 | NA | 1.00 | NA | -1.73 | NA |
| 3 | C06 | 1993 | ELAVL2 | NM_004432 | 185097 | 179266 | 0.94 | 0.83 | 0.02 | 0.06 |
| 3 | C07 | 1995 | ELAVL3 | NM_001420 | 168954 | 182278 | 0.86 | 0.85 | 1.65 | -0.98 |
| 3 | C08 | 9844 | ELMO1 | NM_014800 | 172982 | 159111 | 0.88 | 0.74 | -0.04 | 1.32 |
| 3 | C09 | 8507 | ENC1 | NM_003633 | 190647 | 190172 | 0.97 | 0.89 | -2.99 | -2.15 |
| 3 | C10 | 2022 | ENG | NM_000118 | 191236 | 187976 | 0.97 | 0.87 | -1.93 | -2.38 |
| 3 | C11 | 2060 | EPS15 | NM_001981 | 196238 | 186345 | 1.00 | 0.87 | -2.15 | -0.84 |
| 3 | C12 | 51327 | ERAF | NM_016633 | 125824 | 117598 | 0.64 | 0.55 | 9.77 | 6.64 |
| 3 | D01 | NA | neg | NA | 192438 | 218614 | 0.98 | 1.02 | -3.06 | -4.36 |
| 3 | D02 | NA | neg | NA | 198207 | 201321 | 1.01 | 0.94 | -4.15 | -2.22 |
| 3 | D03 | 26284 | ERAL1 | NM_005702 | 184734 | 180065 | 0.94 | 0.84 | 0.04 | 2.36 |
| 3 | D04 | 51466 | EVL | NM_016337 | 174930 | 184549 | 0.89 | 0.86 | -0.02 | 0.32 |
| 3 | D05 | 2130 | EWSR1 | NM_005243 | 183846 | 183731 | 0.94 | 0.86 | 0.14 | 1.08 |
| 3 | D06 | 2153 | F5 | NM_000130 | 197043 | 177033 | 1.00 | 0.82 | -2.84 | 1.14 |
| 3 | D07 | 2157 | F8 | NM_000132 | 168242 | 180815 | 0.86 | 0.84 | 1.20 | 0.00 |
| 3 | D08 | 2186 | FALZ | NM_004459 | 161983 | 176221 | 0.82 | 0.82 | 1.47 | 0.00 |
| 3 | D09 | 2189 | FANCG | NM_004629 | 165699 | 185913 | 0.84 | 0.87 | 1.17 | -0.82 |
| 3 | D10 | 2220 | FCN2 | NM_004108 | 180449 | 183038 | 0.92 | 0.85 | -0.46 | -0.96 |
| 3 | D11 | 9158 | FIBP | NM_004214 | 187246 | 188538 | 0.95 | 0.88 | -1.02 | -0.31 |
| 3 | D12 | 55156 | FLJ10511 | NM_018120 | 194779 | 191871 | 0.99 | 0.89 | -3.93 | -1.75 |
| 3 | E01 | NA | neg | NA | 201425 | 211118 | 1.03 | 0.98 | -3.67 | -3.15 |
| 3 | E02 | NA | neg | NA | 191053 | 191890 | 0.97 | 0.89 | -1.70 | -0.77 |
| 3 | E03 | 79990 | FLJ21019 | NM_024927 | 187465 | 189530 | 0.95 | 0.88 | 0.62 | 1.47 |
| 3 | E04 | 284306 | ZNF547 | NM_173631 | 185188 | 186144 | 0.94 | 0.87 | -0.87 | 0.40 |
| 3 | E05 | 2317 | FLNB | NM_001457 | 190471 | 186482 | 0.97 | 0.87 | -0.02 | 1.02 |
| 3 | E06 | 2331 | FMOD | NM_002023 | 191055 | 196234 | 0.97 | 0.91 | -0.61 | -0.95 |
| 3 | E07 | 2332 | FMR1 | NM_002024 | 183535 | 190005 | 0.93 | 0.88 | -0.61 | -0.86 |
| 3 | E08 | 2354 | FOSB | NM_006732 | 170308 | 185052 | 0.87 | 0.86 | 0.98 | -0.81 |
| 3 | E09 | 25794 | FSCN2 | NM_012418 | 173181 | 172002 | 0.88 | 0.80 | 0.84 | 1.18 |
| 3 | E10 | 29999 | FSCN3 | NM_020369 | 201393 | 193753 | 1.03 | 0.90 | -3.35 | -2.01 |
| 3 | E11 | 11167 | FSTL1 | NM_007085 | 187320 | 189982 | 0.95 | 0.88 | 0.06 | -0.21 |
| 3 | E12 | 2495 | FTH1 | NM_002032 | 179691 | 178313 | 0.91 | 0.83 | 0.03 | 0.21 |
| 3 | F01 | NA | NA | NA | 190207 | 226735 | 0.97 | 1.06 | -1.73 | -4.90 |
| 3 | F02 | NA | pos | NA | 73582 | 82667 | 0.37 | 0.38 | 20.46 | 12.95 |
| 3 | F03 | 8880 | FUBP1 | NM_003902 | 195109 | 202970 | 0.99 | 0.94 | -1.03 | -0.01 |
| 3 | F04 | 10772 | FUSIP1 | NM_006625 | 179302 | 190559 | 0.91 | 0.89 | 0.05 | 0.04 |

| | | | | | | | | | | |
|---|---|---|---|---|---|---|---|---|---|---|
| 3 | F05 | 8087 | FXR1 | NM_005087 | 186365 | 198200 | 0.95 | 0.92 | 0.57 | -0.24 |
| 3 | F06 | 9513 | FXR2 | NM_004860 | 183360 | 189905 | 0.93 | 0.88 | 0.66 | 0.02 |
| 3 | F07 | 9846 | GAB2 | NM_012296 | 184878 | 174514 | 0.94 | 0.81 | -1.06 | 1.25 |
| 3 | F08 | 2553 | GABPB2 | NM_002041 | 180491 | 179007 | 0.92 | 0.83 | -1.15 | 0.12 |
| 3 | F09 | 8139 | GAN | NM_022041 | 180196 | 184626 | 0.92 | 0.86 | -0.69 | -0.20 |
| 3 | F10 | 9247 | GCM2 | NM_004752 | 178121 | 170636 | 0.91 | 0.79 | 0.88 | 1.04 |
| 3 | F11 | 2669 | GEM | NM_005261 | 182889 | 193045 | 0.93 | 0.90 | 0.71 | -0.40 |
| 3 | F12 | 54810 | GIPC2 | NM_017655 | 179026 | 188460 | 0.91 | 0.88 | -0.03 | -0.87 |
| 3 | G01 | NA | neg | NA | 204131 | 227041 | 1.04 | 1.06 | -3.90 | -4.57 |
| 3 | G02 | NA | neg | NA | 178057 | 224166 | 0.91 | 1.04 | 1.06 | -4.21 |
| 3 | G03 | 2738 | GLI4 | NM_138465 | 195636 | 205830 | 1.00 | 0.96 | -0.66 | 0.00 |
| 3 | G04 | 84662 | GLIS2 | NM_032575 | 181978 | 194167 | 0.93 | 0.90 | 0.02 | -0.04 |
| 3 | G05 | 2814 | GP5 | NM_004488 | 191728 | 188294 | 0.98 | 0.88 | 0.02 | 1.35 |
| 3 | G06 | 2815 | GP9 | NM_000174 | 189442 | 193131 | 0.96 | 0.90 | -0.02 | -0.02 |
| 3 | G07 | 160622 | GRASP | NM_181711 | 185383 | 193497 | 0.94 | 0.90 | -0.68 | -0.74 |
| 3 | G08 | 2926 | GRSF1 | NM_002092 | 174275 | 182955 | 0.89 | 0.85 | 0.51 | 0.00 |
| 3 | G09 | 2934 | GSN | NM_000177 | 178603 | 186904 | 0.91 | 0.87 | 0.09 | -0.11 |
| 3 | G10 | 9260 | PDLIM7 | NM_005451 | 181765 | 169123 | 0.93 | 0.79 | 0.66 | 1.60 |
| 3 | G11 | 84163 | GTF2IRD2 | NM_173537 | 189452 | 181984 | 0.96 | 0.85 | -0.06 | 1.34 |
| 3 | G12 | 2975 | GTF3C1 | NM_001520 | 185677 | 181117 | 0.95 | 0.84 | -0.82 | 0.41 |
| 3 | H01 | NA | NA | NA | 210183 | 220240 | 1.07 | 1.03 | -4.86 | -3.24 |
| 3 | H02 | NA | pos | NA | 75580 | 79041 | 0.38 | 0.37 | 20.75 | 14.25 |
| 3 | H03 | 3005 | H1F0 | NM_005318 | 193416 | 224784 | 0.98 | 1.05 | -0.04 | -1.86 |
| 3 | H04 | 55766 | H2AFJ | NM_018267 | 192374 | 206481 | 0.98 | 0.96 | -1.77 | -1.08 |
| 3 | H05 | 3015 | H2AFZ | NM_002106 | 206333 | 203131 | 1.05 | 0.95 | -2.57 | 0.00 |
| 3 | H06 | 3021 | H3F3B | NM_005324 | 186194 | 193396 | 0.95 | 0.90 | 0.79 | 0.44 |
| 3 | H07 | 9982 | HBP17 | NM_005130 | 192931 | 191479 | 0.98 | 0.89 | -1.93 | 0.00 |
| 3 | H08 | 3280 | HES1 | NM_005524 | 191632 | 195739 | 0.98 | 0.91 | -2.60 | -1.10 |
| 3 | H09 | 57801 | HES4 | NM_021170 | 179176 | 181120 | 0.91 | 0.84 | 0.17 | 1.09 |
| 3 | H10 | 84667 | HES7 | NM_032580 | 184446 | 173515 | 0.94 | 0.81 | 0.35 | 1.54 |
| 3 | H11 | 25994 | HIG1 | NM_014056 | 188714 | 189745 | 0.96 | 0.88 | 0.27 | 0.86 |
| 3 | H12 | 28988 | HIP-55 | NM_014063 | 182027 | 190262 | 0.93 | 0.89 | 0.06 | -0.24 |
| 4 | A01 | NA | pos | NA | 77049 | 76251 | 0.43 | 0.41 | 15.90 | 15.55 |
| 4 | A02 | NA | NA | NA | 174015 | 172070 | 0.96 | 0.92 | -0.89 | -1.21 |
| 4 | A03 | 28988 | HIP-55 | NM_014063 | 165193 | 169701 | 0.91 | 0.91 | 0.40 | -0.85 |
| 4 | A04 | 3006 | HIST1H1C | NM_005319 | 165206 | 163263 | 0.91 | 0.87 | 0.89 | 0.12 |
| 4 | A05 | 8349 | HIST2H2BE | NM_003528 | 178179 | 177407 | 0.98 | 0.95 | -0.36 | -0.11 |
| 4 | A06 | 3097 | HIVEP2 | NM_006734 | 142472 | 170819 | 0.79 | 0.91 | 0.83 | -0.13 |
| 4 | A07 | 10042 | HMG2L1 | NM_005487 | 180427 | 165901 | 1.00 | 0.88 | -1.76 | 0.52 |
| 4 | A08 | 8091 | HMGA2 | NM_003483 | 207308 | 160533 | 1.15 | 0.86 | -4.99 | 0.12 |
| 4 | A09 | 3151 | HMGN2 | NM_005517 | 175086 | 176774 | 0.97 | 0.94 | -3.57 | -2.33 |
| 4 | A10 | 3161 | HMMR | NM_012484 | 154666 | 159120 | 0.85 | 0.85 | 0.28 | 0.13 |
| 4 | A11 | 3184 | HNRPD | NM_002138 | 175374 | 167664 | 0.97 | 0.89 | -0.29 | -0.42 |
| 4 | A12 | 50863 | HNT | NM_016522 | 115266 | 150220 | 0.64 | 0.80 | 2.43 | 2.88 |
| 4 | B01 | NA | neg | NA | 195398 | 209754 | 1.08 | 1.12 | -2.02 | -6.54 |
| 4 | B02 | NA | neg | NA | 185345 | 186145 | 1.02 | 0.99 | -0.27 | -2.41 |
| 4 | B03 | 3241 | HPCAL1 | NM_002149 | 194120 | 181392 | 1.07 | 0.97 | -2.02 | -1.63 |
| 4 | B04 | 9410 | HPRP8BP | NM_004814 | 185673 | 179507 | 1.03 | 0.96 | -0.08 | -1.45 |
| 4 | B05 | 89781 | HPS4 | NM_022081 | 190058 | 185065 | 1.05 | 0.99 | 0.16 | -0.18 |
| 4 | B06 | 11103 | HRB2 | NM_007043 | 151125 | 169748 | 0.84 | 0.91 | 1.91 | 1.33 |
| 4 | B07 | 3304 | HSPA1B | NM_005346 | 186353 | 174634 | 1.03 | 0.93 | -0.20 | 0.26 |
| 4 | B08 | 3305 | HSPA1L | NM_005527 | 195952 | 173384 | 1.08 | 0.92 | -0.44 | -0.86 |
| 4 | B09 | 3306 | HSPA2 | NM_021979 | 168709 | 165780 | 0.93 | 0.88 | 0.12 | 0.86 |
| 4 | B10 | 3308 | HSPA4 | NM_002154 | 181422 | 175860 | 1.00 | 0.94 | -1.77 | -1.53 |
| 4 | B11 | 3309 | HSPA5 | NM_005347 | 164397 | 150859 | 0.91 | 0.80 | 4.19 | 3.79 |
| 4 | B12 | 3310 | HSPA6 | NM_002155 | 125026 | 172737 | 0.69 | 0.92 | 3.32 | 0.21 |
| 4 | C01 | NA | pos | NA | 80183 | 86901 | 0.44 | 0.46 | 17.28 | 16.43 |
| 4 | C02 | NA | NA | NA | 175089 | 169550 | 0.97 | 0.90 | 0.85 | 1.97 |
| 4 | C03 | 3313 | HSPA9B | NM_004134 | 181606 | 180092 | 1.00 | 0.96 | -0.51 | 0.07 |
| 4 | C04 | 27129 | HSPB7 | NM_014424 | 181013 | 172836 | 1.00 | 0.92 | 0.08 | 1.19 |
| 4 | C05 | 3329 | HSPD1 | NM_002156 | 189000 | 192902 | 1.04 | 1.03 | -0.31 | -0.08 |
| 4 | C06 | 10808 | HSPH1 | NM_006644 | 145382 | 189308 | 0.80 | 1.01 | 2.25 | -0.62 |
| 4 | C07 | 29935 | HSU24186 | NM_013347 | 180244 | 185640 | 1.00 | 0.99 | 0.20 | -0.19 |
| 4 | C08 | 55109 | VG5Q | NM_018046 | 191931 | 169126 | 1.06 | 0.90 | -0.40 | 1.36 |
| 4 | C09 | 54739 | HSXIAPAF1 | NM_017523 | 171294 | 186789 | 0.95 | 1.00 | -0.98 | -1.34 |
| 4 | C10 | 10525 | HYOU1 | NM_006389 | 159450 | 176326 | 0.88 | 0.94 | 1.38 | -0.13 |
| 4 | C11 | 3381 | IBSP | NM_004967 | 179821 | 180250 | 0.99 | 0.96 | 0.87 | 0.12 |
| 4 | C12 | 7087 | ICAM5 | NM_003259 | 140652 | 177832 | 0.78 | 0.95 | -0.03 | 0.80 |
| 4 | D01 | NA | neg | NA | 186985 | 187502 | 1.03 | 1.00 | -1.42 | -1.34 |
| 4 | D02 | NA | neg | NA | 180989 | 186780 | 1.00 | 1.00 | -0.39 | -1.21 |
| 4 | D03 | 3430 | IFI35 | NM_005533 | 168228 | 179952 | 0.93 | 0.96 | 1.59 | -0.07 |
| 4 | D04 | 3483 | IGFALS | NM_004970 | 184611 | 172801 | 1.02 | 0.92 | -0.76 | 1.02 |
| 4 | D05 | 3484 | IGFBP1 | NM_000596 | 178627 | 193416 | 0.99 | 1.03 | 1.27 | -0.34 |
| 4 | D06 | 3485 | IGFBP2 | NM_000597 | 161956 | 184058 | 0.90 | 0.98 | -0.83 | 0.13 |
| 4 | D07 | 3487 | IGFBP4 | NM_001552 | 171564 | 188017 | 0.95 | 1.00 | 1.49 | -0.78 |
| 4 | D08 | 3488 | IGFBP5 | NM_000599 | 186898 | 164619 | 1.03 | 0.88 | 0.26 | 1.98 |
| 4 | D09 | 3489 | IGFBP6 | NM_002178 | 158309 | 185081 | 0.87 | 0.99 | 1.05 | -1.21 |
| 4 | D10 | 3490 | IGFBP7 | NM_001553 | 167767 | 172623 | 0.93 | 0.92 | -0.27 | 0.34 |
| 4 | D11 | 10642 | IMP-1 | NM_006546 | 190169 | 175688 | 1.05 | 0.94 | -1.14 | 0.75 |
| 4 | D12 | 10644 | IMP-2 | NM_006548 | 151775 | 182621 | 0.84 | 0.97 | -2.17 | -0.21 |
| 4 | E01 | NA | neg | NA | 169974 | 202044 | 0.94 | 1.08 | 1.01 | -5.92 |
| 4 | E02 | NA | neg | NA | 176678 | 179841 | 0.98 | 0.96 | -0.16 | -2.03 |
| 4 | E03 | 3621 | ING1 | NM_005537 | 177284 | 156021 | 0.98 | 0.83 | -0.49 | 2.08 |
| 4 | E04 | 3622 | ING1L | NM_001564 | 186165 | 180636 | 1.03 | 0.96 | -1.54 | -2.38 |
| 4 | E05 | 54556 | ING3 | NM_019071 | 182662 | 162286 | 1.01 | 0.87 | 0.06 | 3.07 |
| 4 | E06 | 51147 | ING4 | NM_016162 | 163243 | 180417 | 0.90 | 0.96 | -1.57 | -1.27 |
| 4 | E07 | 3642 | INSM1 | NM_002196 | 171847 | 170879 | 0.95 | 0.91 | 0.93 | 0.19 |
| 4 | E08 | 84684 | INSM2 | NM_032594 | 184912 | 181244 | 1.02 | 0.97 | 0.09 | -2.96 |

| | | | | | | | | | | |
|---|---|---|---|---|---|---|---|---|---|---|
| 4 | E09 | 10260 | IRLB | NM_005848 | 162066 | 167863 | 0.90 | 0.90 | -0.12 | -0.23 |
| 4 | E10 | 3685 | ITGAV | NM_002210 | 138507 | 139360 | 0.77 | 0.74 | 4.28 | 4.13 |
| 4 | E11 | 26548 | ITGB1BP2 | NM_012278 | 180677 | 181862 | 1.00 | 0.97 | -0.01 | -2.36 |
| 4 | E12 | 10625 | IVNS1ABP | NM_006469 | 136048 | 168052 | 0.75 | 0.90 | 0.03 | 0.30 |
| 4 | F01 | NA | NA | NA | 187806 | 193802 | 1.04 | 1.03 | -2.19 | -2.34 |
| 4 | F02 | NA | pos | NA | 87028 | 72461 | 0.48 | 0.39 | 15.26 | 18.89 |
| 4 | F03 | 23512 | JJAZ1 | NM_015355 | 173510 | 170935 | 0.96 | 0.91 | 0.05 | 1.61 |
| 4 | F04 | 3720 | JARID2 | NM_004973 | 161569 | 169846 | 0.89 | 0.91 | 2.60 | 1.65 |
| 4 | F05 | 10657 | KHDRBS1 | NM_006559 | 182430 | 171471 | 1.01 | 0.91 | -0.01 | 3.61 |
| 4 | F06 | 8570 | KHSRP | NM_003685 | 131167 | 187569 | 0.72 | 1.00 | 3.87 | -0.38 |
| 4 | F07 | 9813 | KIAA0494 | XM_375726 | 184044 | 189829 | 1.02 | 1.01 | -1.30 | -0.99 |
| 4 | F08 | 9877 | KIAA0663 | XM_378929 | 178234 | 170636 | 0.99 | 0.91 | 1.13 | 1.03 |
| 4 | F09 | 23158 | KIAA0882 | XM_093895 | 163604 | 176414 | 0.90 | 0.94 | -0.49 | 0.41 |
| 4 | F10 | 57554 | KIAA1365 | NM_020794 | 172260 | 181949 | 0.95 | 0.97 | -1.68 | -1.18 |
| 4 | F11 | 85453 | TSPYL5 | NM_033512 | 179920 | 183331 | 0.99 | 0.98 | 0.01 | -0.48 |
| 4 | F12 | 22920 | KIFAP3 | NM_014970 | 143013 | 184779 | 0.79 | 0.99 | -1.28 | -0.48 |
| 4 | G01 | NA | neg | NA | 180730 | 196545 | 1.00 | 1.05 | -0.40 | -4.06 |
| 4 | G02 | NA | neg | NA | 180893 | NA | 1.00 | NA | -0.43 | NA |
| 4 | G03 | 90990 | KIFC2 | NM_145754 | 177390 | 175684 | 0.98 | 0.94 | -0.05 | -0.46 |
| 4 | G04 | 22944 | KIN | NM_012311 | 177276 | 173534 | 0.98 | 0.93 | 0.45 | -0.24 |
| 4 | G05 | 10518 | CIB2 | NM_006383 | 204746 | 181416 | 1.13 | 0.97 | -3.31 | 0.62 |
| 4 | G06 | 117286 | CIB3 | NM_054113 | 201014 | 175116 | 1.11 | 0.93 | -7.65 | 0.55 |
| 4 | G07 | 136259 | KLF14 | NM_138693 | 184827 | 179961 | 1.02 | 0.96 | -0.86 | -0.51 |
| 4 | G08 | 28999 | KLF15 | NM_014079 | 176170 | 177010 | 0.97 | 0.94 | 2.06 | -1.33 |
| 4 | G09 | 83855 | KLF16 | NM_031918 | 156951 | 170316 | 0.87 | 0.91 | 1.23 | 0.23 |
| 4 | G10 | 11133 | KPTN | NM_007059 | 165679 | 162689 | 0.92 | 0.87 | 0.03 | 0.94 |
| 4 | G11 | 3897 | L1CAM | NM_000425 | 168037 | 169739 | 0.93 | 0.91 | 2.64 | 0.65 |
| 4 | G12 | 3899 | LAF4 | NM_002285 | 145526 | 179444 | 0.80 | 0.96 | -1.15 | -0.79 |
| 4 | H01 | NA | NA | NA | 180746 | 201017 | 1.00 | 1.07 | 0.99 | -2.97 |
| 4 | H02 | NA | pos | NA | 59265 | 82380 | 0.33 | 0.44 | 22.02 | 17.79 |
| 4 | H03 | 3902 | LAG3 | NM_002286 | 168774 | 169610 | 0.93 | 0.90 | 2.83 | 2.47 |
| 4 | H04 | 3929 | LBP | NM_004139 | 188901 | 183558 | 1.04 | 0.98 | -0.17 | -0.12 |
| 4 | H05 | 51176 | LEF1 | NM_016269 | 193607 | 195257 | 1.07 | 1.04 | 0.01 | 0.08 |
| 4 | H06 | 85329 | LGALS12 | NM_033101 | 196970 | 182809 | 1.09 | 0.97 | -5.56 | 1.09 |
| 4 | H07 | 3957 | LGALS2 | NM_006498 | 183707 | 176552 | 1.02 | 0.94 | 0.72 | 1.97 |
| 4 | H08 | 3960 | LGALS4 | NM_006149 | 196610 | 180845 | 1.09 | 0.96 | -0.09 | -0.12 |
| 4 | H09 | 3964 | LGALS8 | NM_006499 | 156701 | 180410 | 0.87 | 0.96 | 2.66 | 0.34 |
| 4 | H10 | 10186 | LHFP | NM_005780 | 174047 | 179866 | 0.96 | 0.96 | -0.03 | -0.19 |
| 4 | H11 | 8994 | LIMD1 | NM_014240 | 195659 | 184888 | 1.08 | 0.99 | -0.76 | -0.12 |
| 4 | H12 | 8825 | LIN7A | NM_004664 | 145293 | 188631 | 0.80 | 1.01 | 0.28 | -0.52 |
| 5 | A01 | NA | pos | NA | 95157 | 89681 | 0.47 | 0.45 | 14.85 | 19.76 |
| 5 | A02 | NA | NA | NA | 181962 | 186776 | 0.89 | 0.94 | 1.15 | -0.11 |
| 5 | A03 | 3998 | LMAN1 | NM_005570 | 197207 | 180964 | 0.97 | 0.91 | -0.78 | 1.76 |
| 5 | A04 | 25802 | LMOD1 | NM_012134 | 197092 | 190820 | 0.97 | 0.96 | -1.04 | -0.89 |
| 5 | A05 | 10128 | LRPPRC | NM_133259 | 198311 | 193779 | 0.97 | 0.98 | -0.34 | 0.15 |
| 5 | A06 | 4053 | LTBP2 | NM_000428 | 177999 | 181539 | 0.87 | 0.92 | 1.43 | 1.39 |
| 5 | A07 | 4077 | M17S2 | NM_005899 | 196907 | 170104 | 0.97 | 0.86 | -1.22 | 2.69 |
| 5 | A08 | 22823 | M96 | NM_007358 | 174253 | 191789 | 0.85 | 0.97 | 1.92 | -1.39 |
| 5 | A09 | 23499 | MACF1 | NM_012090 | 172777 | 187536 | 0.85 | 0.95 | 2.68 | -0.14 |
| 5 | A10 | 8174 | MADCAM1 | NM_130760 | 173810 | 180470 | 0.85 | 0.91 | 1.02 | -0.67 |
| 5 | A11 | 4087 | SMAD2 | NM_005901 | 195344 | 188300 | 0.96 | 0.95 | -0.94 | -1.48 |
| 5 | A12 | 9935 | MAFB | NM_005461 | 182874 | 178509 | 0.90 | 0.90 | 0.34 | 1.55 |
| 5 | B01 | NA | neg | NA | 229528 | 219849 | 1.13 | 1.11 | -6.12 | -6.32 |
| 5 | B02 | NA | neg | NA | 196498 | 196152 | 0.96 | 0.99 | -0.90 | -1.47 |
| 5 | B03 | 23764 | MAFF | NM_012323 | 188008 | 192207 | 0.92 | 0.97 | 0.91 | 0.02 |
| 5 | B04 | 22919 | MAPRE1 | NM_012325 | 192029 | 192819 | 0.94 | 0.97 | -0.01 | -0.74 |
| 5 | B05 | 10982 | MAPRE2 | NM_014268 | 204356 | 192747 | 1.00 | 0.97 | -1.05 | 0.92 |
| 5 | B06 | 4150 | MAZ | NM_002383 | 207164 | 201274 | 1.02 | 1.02 | -2.93 | -2.10 |
| 5 | B07 | 53615 | MBD3 | NM_003926 | 202750 | 198498 | 0.99 | 1.00 | -1.90 | -2.56 |
| 5 | B08 | 4153 | MBL2 | NM_000242 | 182339 | 187126 | 0.89 | 0.95 | 0.89 | 0.12 |
| 5 | B09 | 4162 | MCAM | NM_006500 | 191257 | 193082 | 0.94 | 0.98 | 0.01 | -0.72 |
| 5 | B10 | 8888 | MCM3AP | NM_003906 | 172473 | 179799 | 0.85 | 0.91 | 1.47 | 0.02 |
| 5 | B11 | 4210 | MEFV | NM_000243 | 190619 | 183833 | 0.93 | 0.93 | 0.04 | -0.01 |
| 5 | B12 | 4221 | MEN1 | NM_000244 | 189131 | 188693 | 0.93 | 0.95 | -0.40 | 0.02 |
| 5 | C01 | NA | pos | NA | 92748 | 91427 | 0.45 | 0.46 | 15.97 | 22.31 |
| 5 | C02 | NA | NA | NA | 205117 | 198308 | 1.01 | 1.00 | -1.76 | 0.44 |
| 5 | C03 | 4240 | MFGE8 | NM_005928 | 199145 | 203859 | 0.98 | 1.03 | -0.35 | -0.02 |
| 5 | C04 | 9258 | MFHAS1 | NM_004225 | 181492 | 201665 | 0.89 | 1.02 | 2.16 | -0.20 |
| 5 | C05 | 65997 | RASL11B | NM_023940 | 182033 | 215682 | 0.89 | 1.09 | 2.97 | -1.42 |
| 5 | C06 | 4276 | MICA | NM_000247 | 195295 | 205309 | 0.96 | 1.04 | -0.56 | -0.57 |
| 5 | C07 | 4277 | MICB | NM_005931 | 199836 | 197646 | 0.98 | 1.00 | -0.94 | -0.04 |
| 5 | C08 | 57708 | MI-ER1 | NM_020948 | 198012 | 197785 | 0.97 | 1.00 | -1.09 | 0.29 |
| 5 | C09 | 9063 | PIAS2 | NM_004671 | 192284 | 200404 | 0.94 | 1.01 | 0.35 | 0.14 |
| 5 | C10 | 25988 | MIZF | NM_015517 | 192693 | 191334 | 0.95 | 0.97 | -1.22 | 0.01 |
| 5 | C11 | 4288 | MKI67 | NM_002417 | 188578 | 190358 | 0.92 | 0.96 | 0.87 | 1.00 |
| 5 | C12 | 84365 | MKI67IP | NM_032390 | 176391 | 177118 | 0.87 | 0.89 | 2.11 | 4.74 |
| 5 | D01 | NA | neg | NA | 223785 | 189198 | 1.10 | 0.96 | -4.39 | 1.88 |
| 5 | D02 | NA | neg | NA | 191007 | 199652 | 0.94 | 1.01 | 0.78 | -0.26 |
| 5 | D03 | 8195 | MKKS | NM_018848 | 193354 | 200004 | 0.95 | 1.01 | 0.88 | 0.35 |
| 5 | D04 | 57591 | MKL1 | NM_020831 | 192740 | 186652 | 0.95 | 0.94 | 0.70 | 2.45 |
| 5 | D05 | 4291 | MLF1 | NM_022443 | 195103 | 191960 | 0.96 | 0.97 | 1.22 | 3.01 |
| 5 | D06 | 4292 | MLH1 | NM_000249 | 201514 | 213900 | 0.99 | 1.08 | -1.22 | -2.75 |
| 5 | D07 | 27030 | MLH3 | NM_014381 | 198319 | 179474 | 0.97 | 0.91 | -0.38 | 3.26 |
| 5 | D08 | 55904 | MLL5 | NM_018682 | 192801 | 199598 | 0.95 | 1.01 | 0.05 | -0.51 |
| 5 | D09 | 4301 | MLLT4 | NM_005936 | 198798 | 211905 | 0.98 | 1.07 | -0.37 | -2.64 |
| 5 | D10 | 4302 | MLLT6 | NM_005937 | 171834 | 189406 | 0.84 | 0.96 | 2.39 | -0.01 |
| 5 | D11 | 254251 | MLR1 | NM_153686 | 196352 | 193135 | 0.96 | 0.98 | -0.04 | 0.01 |
| 5 | D12 | 166785 | MMAA | NM_172250 | 198434 | 198355 | 0.97 | 1.00 | -1.05 | -0.02 |

| | | | | | | | | | | |
|---|---|---|---|---|---|---|---|---|---|---|
| 5 | E01 | NA | neg | NA | 223374 | 204352 | 1.10 | 1.03 | -5.77 | -3.18 |
| 5 | E02 | NA | neg | NA | 184568 | 189761 | 0.91 | 0.96 | 0.35 | -0.20 |
| 5 | E03 | 4332 | MNDA | NM_002432 | 191359 | 193252 | 0.94 | 0.98 | -0.25 | -0.23 |
| 5 | E04 | 84159 | ARID5B | XM_084482 | 170145 | 169215 | 0.83 | 0.86 | 2.82 | 4.05 |
| 5 | E05 | 57496 | MRTF-B | NM_014048 | 192829 | 195905 | 0.95 | 0.99 | 0.14 | 0.24 |
| 5 | E06 | 10801 | MSF | NM_006640 | 180915 | 188084 | 0.89 | 0.95 | 0.59 | 0.57 |
| 5 | E07 | 4436 | MSH2 | NM_000251 | 184172 | 188903 | 0.90 | 0.95 | 0.40 | -0.63 |
| 5 | E08 | 4438 | MSH4 | NM_002440 | 184837 | 172154 | 0.91 | 0.87 | -0.13 | 3.15 |
| 5 | E09 | 4439 | MSH5 | NM_002441 | 188104 | 198584 | 0.92 | 1.00 | -0.12 | -1.88 |
| 5 | E10 | 2956 | MSH6 | NM_000179 | 194744 | 199368 | 0.96 | 1.01 | -2.67 | -4.02 |
| 5 | E11 | 10232 | MSLN | NM_005823 | 186109 | 179867 | 0.91 | 0.91 | 0.13 | 0.76 |
| 5 | E12 | 23787 | MTCH1 | NM_014341 | 192833 | 192196 | 0.95 | 0.97 | -1.61 | -0.73 |
| 5 | F01 | NA | NA | NA | 207404 | 215566 | 1.02 | 1.09 | -2.79 | -3.38 |
| 5 | F02 | NA | pos | NA | 81159 | 81863 | 0.40 | 0.41 | 17.14 | 23.98 |
| 5 | F03 | 93649 | MYOCD | NM_153604 | 192341 | 202587 | 0.94 | 1.02 | 0.06 | -0.04 |
| 5 | F04 | 4619 | MYH1 | NM_005963 | 194854 | 188625 | 0.96 | 0.95 | -0.62 | 2.18 |
| 5 | F05 | 4629 | MYH11 | NM_002474 | 199704 | 208055 | 0.98 | 1.05 | -0.48 | -0.15 |
| 5 | F06 | 8735 | MYH13 | NM_003802 | 175492 | 193412 | 0.86 | 0.98 | 1.90 | 1.58 |
| 5 | F07 | 4620 | MYH2 | NM_017534 | 177416 | 195906 | 0.87 | 0.99 | 1.93 | 0.04 |
| 5 | F08 | 4624 | MYH6 | NM_002471 | 187288 | 198372 | 0.92 | 1.00 | -0.06 | -0.12 |
| 5 | F09 | 4627 | MYH9 | NM_002473 | 187405 | 180929 | 0.92 | 0.91 | 0.45 | 3.84 |
| 5 | F10 | 10398 | MYL9 | NM_006097 | 176274 | 194430 | 0.86 | 0.98 | 0.70 | -0.90 |
| 5 | F11 | 4651 | MYO10 | NM_012334 | 191086 | 198460 | 0.94 | 1.00 | -0.19 | -0.94 |
| 5 | F12 | 25924 | MYRIP | NM_015460 | 187712 | 195373 | 0.92 | 0.99 | -0.34 | 0.72 |
| 5 | G01 | NA | neg | NA | 207039 | 214823 | 1.02 | 1.09 | -1.79 | -4.74 |
| 5 | G02 | NA | neg | NA | 200703 | 194682 | 0.98 | 0.98 | -0.79 | -0.62 |
| 5 | G03 | 26993 | AKAP8L | NM_014371 | 199079 | 199309 | 0.98 | 1.01 | -0.06 | -0.89 |
| 5 | G04 | 338322 | NALP10 | NM_176821 | 196881 | 190852 | 0.97 | 0.96 | 0.01 | 0.20 |
| 5 | G05 | 126204 | NALP13 | NM_176810 | 203477 | 200829 | 1.00 | 1.01 | -0.14 | -0.19 |
| 5 | G06 | 338323 | NALP14 | NM_176822 | 193850 | 196589 | 0.95 | 0.99 | -0.05 | -0.59 |
| 5 | G07 | 55655 | NALP2 | NM_017852 | 182086 | 197627 | 0.89 | 1.00 | 2.14 | -1.84 |
| 5 | G08 | 4683 | NBS1 | NM_002485 | 182224 | 187172 | 0.89 | 0.95 | 1.68 | 0.65 |
| 5 | G09 | 4684 | NCAM1 | NM_000615 | 196247 | 183500 | 0.96 | 0.93 | -0.01 | 1.79 |
| 5 | G10 | 4685 | NCAM2 | NM_004540 | 196899 | 172723 | 0.97 | 0.87 | -1.61 | 2.02 |
| 5 | G11 | 4686 | NCBP1 | NM_002486 | 191090 | 182203 | 0.94 | 0.92 | 0.75 | 0.87 |
| 5 | G12 | 4692 | NDN | NM_002487 | 186599 | 195430 | 0.92 | 0.99 | 0.77 | -0.81 |
| 5 | H01 | NA | NA | NA | 202715 | 220882 | 0.99 | 1.12 | -0.62 | -4.50 |
| 5 | H02 | NA | pos | NA | 83694 | 91569 | 0.41 | 0.46 | 18.17 | 21.96 |
| 5 | H03 | 4756 | NEO1 | NM_002499 | 196065 | 193867 | 0.96 | 0.98 | 0.90 | 1.71 |
| 5 | H04 | 4792 | NFKBIA | NM_020529 | 212252 | 216297 | 1.04 | 1.09 | -1.93 | -3.52 |
| 5 | H05 | 4807 | NHLH1 | NM_005598 | 199420 | 212381 | 0.98 | 1.07 | 0.99 | -1.07 |
| 5 | H06 | 22795 | NID2 | NM_007361 | 196276 | 197807 | 0.96 | 1.00 | 0.05 | 0.64 |
| 5 | H07 | 4814 | NINJ1 | NM_004148 | 196295 | 186796 | 0.96 | 0.94 | 0.38 | 1.86 |
| 5 | H08 | 8996 | NOL3 | NM_003946 | 196306 | 202685 | 0.96 | 1.02 | -0.05 | -1.04 |
| 5 | H09 | 9221 | NOLC1 | NM_004741 | 201252 | 197564 | 0.99 | 1.00 | -0.31 | 0.40 |
| 5 | H10 | 4857 | NOVA1 | NM_002515 | 194252 | 183691 | 0.95 | 0.93 | -0.70 | 1.25 |
| 5 | H11 | 4858 | NOVA2 | NM_002516 | 201824 | 195575 | 0.99 | 0.99 | -0.46 | -0.39 |
| 5 | H12 | 4868 | NPHS1 | NM_004646 | 188680 | 202206 | 0.93 | 1.02 | 0.93 | -0.71 |
| 6 | A01 | NA | pos | NA | 91974 | 83796 | 0.46 | 0.43 | 18.78 | 20.23 |
| 6 | A02 | NA | NA | NA | 181340 | 194604 | 0.90 | 0.99 | 0.49 | -1.65 |
| 6 | A03 | 4897 | NRCAM | NM_005010 | 190798 | 175522 | 0.95 | 0.90 | -1.81 | 1.93 |
| 6 | A04 | 4900 | NRGN | NM_006176 | 185044 | 196471 | 0.92 | 1.00 | -0.01 | -2.11 |
| 6 | A05 | 4682 | NUBP1 | NM_002484 | 203698 | 187260 | 1.01 | 0.96 | -2.25 | 1.93 |
| 6 | A06 | 10101 | NUBP2 | NM_012225 | 189519 | 193328 | 0.94 | 0.99 | -1.14 | -0.57 |
| 6 | A07 | 4924 | NUCB1 | NM_006184 | 187438 | 185582 | 0.93 | 0.95 | -0.81 | 0.22 |
| 6 | A08 | 4925 | NUCB2 | NM_005013 | 179640 | 186591 | 0.89 | 0.95 | 0.01 | -0.53 |
| 6 | A09 | 10215 | OLIG2 | NM_005806 | 182792 | 190590 | 0.91 | 0.97 | 0.94 | -0.22 |
| 6 | A10 | 23594 | ORC6L | NM_014321 | 181567 | 175277 | 0.90 | 0.90 | 0.22 | 2.07 |
| 6 | A11 | 5007 | OSBP | NM_002556 | 180393 | 189654 | 0.90 | 0.97 | 0.40 | -0.93 |
| 6 | A12 | 114876 | OSBPL1A | NM_018030 | 183311 | 165479 | 0.91 | 0.85 | 0.64 | 5.29 |
| 6 | B01 | NA | neg | NA | 217209 | 214352 | 1.08 | 1.10 | -4.94 | -4.90 |
| 6 | B02 | NA | neg | NA | 193803 | 190016 | 0.97 | 0.97 | -0.15 | -0.10 |
| 6 | B03 | 10631 | POSTN | NM_006475 | 187100 | 182099 | 0.93 | 0.93 | 0.86 | 1.28 |
| 6 | B04 | 26578 | OSTF1 | NM_012383 | 199759 | 190557 | 0.99 | 0.97 | -1.11 | -0.30 |
| 6 | B05 | 29763 | PACSIN3 | NM_016223 | 183774 | 196537 | 0.92 | 1.00 | 3.74 | 0.74 |
| 6 | B06 | 5068 | PAP | NM_002580 | 186611 | 196821 | 0.93 | 1.01 | 1.37 | -0.61 |
| 6 | B07 | 56288 | PARD3 | NM_019619 | 192797 | 184907 | 0.96 | 0.95 | 0.01 | 1.00 |
| 6 | B08 | 55742 | PARVA | NM_018222 | 199679 | 194668 | 0.99 | 0.99 | -2.18 | -1.47 |
| 6 | B09 | 5075 | PAX1 | NM_006192 | 198185 | 192815 | 0.99 | 0.99 | -0.30 | -0.01 |
| 6 | B10 | 5079 | PAX5 | NM_016734 | 193096 | 185110 | 0.96 | 0.95 | -0.22 | 0.78 |
| 6 | B11 | 5083 | PAX9 | NM_006194 | 185137 | 188204 | 0.92 | 0.96 | 1.34 | 0.01 |
| 6 | B12 | 55893 | ZNF395 | NM_018660 | 195779 | 197397 | 0.97 | 1.01 | 0.00 | -0.36 |
| 6 | C01 | NA | pos | NA | 91937 | 87498 | 0.46 | 0.45 | 20.94 | 21.45 |
| 6 | C02 | NA | NA | NA | 198201 | 187119 | 0.99 | 0.96 | -0.82 | 1.78 |
| 6 | C03 | 5094 | PCBP2 | NM_005016 | 189574 | 192660 | 0.94 | 0.98 | 0.58 | 0.50 |
| 6 | C04 | 57060 | PCBP4 | NM_020418 | 195527 | 195237 | 0.97 | 1.00 | -0.01 | 0.08 |
| 6 | C05 | 5097 | PCDH1 | NM_002587 | 215586 | 211781 | 1.07 | 1.08 | -2.54 | -0.96 |
| 6 | C06 | 27328 | PCDH11X | NM_014522 | 193732 | 197713 | 0.96 | 1.01 | 0.15 | 0.52 |
| 6 | C07 | 83259 | PCDH11Y | NM_032971 | 192341 | 195419 | 0.96 | 1.00 | 0.33 | 0.23 |
| 6 | C08 | 51294 | PCDH12 | NM_016580 | 194297 | 191005 | 0.97 | 0.98 | -0.84 | 0.55 |
| 6 | C09 | 65217 | PCDH15 | NM_033056 | 207975 | 211338 | 1.04 | 1.08 | -2.07 | -2.36 |
| 6 | C10 | 8642 | PCDH16 | NM_003737 | 190446 | 206322 | 0.95 | 1.05 | 0.55 | -2.10 |
| 6 | C11 | 5099 | PCDH7 | NM_002589 | 192790 | 206233 | 0.96 | 1.05 | 0.01 | -2.25 |
| 6 | C12 | 5100 | PCDH8 | NM_002590 | 205827 | 202650 | 1.02 | 1.04 | -1.83 | -0.09 |
| 6 | D01 | NA | neg | NA | 206822 | 189590 | 1.03 | 0.97 | -2.88 | 0.29 |
| 6 | D02 | NA | neg | NA | 195903 | 198255 | 0.98 | 1.01 | -0.65 | -1.42 |
| 6 | D03 | 56147 | PCDHA1 | NM_018900 | 197675 | 190060 | 0.98 | 0.97 | -1.38 | 0.00 |
| 6 | D04 | 56139 | PCDHA10 | NM_018901 | 178309 | 191466 | 0.89 | 0.98 | 3.21 | -0.18 |

| | | | | | | | | | | |
|---|---|---|---|---|---|---|---|---|---|---|
| 6 | D05 | 56138 | PCDHA11 | NM_018902 | 194262 | 218242 | 0.97 | 1.12 | 1.53 | -3.24 |
| 6 | D06 | 56137 | PCDHA12 | NM_018903 | 195966 | 192018 | 0.98 | 0.98 | -0.61 | 0.64 |
| 6 | D07 | 56136 | PCDHA13 | NM_018904 | 202730 | 200100 | 1.01 | 1.02 | -2.10 | -1.70 |
| 6 | D08 | 56146 | PCDHA2 | NM_018905 | 185736 | 183216 | 0.92 | 0.94 | 0.61 | 1.09 |
| 6 | D09 | 56145 | PCDHA3 | NM_018906 | 199829 | 202721 | 1.00 | 1.04 | -0.71 | -1.67 |
| 6 | D10 | 56144 | PCDHA4 | NM_018907 | 180127 | 188829 | 0.90 | 0.97 | 2.36 | 0.34 |
| 6 | D11 | 56143 | PCDHA5 | NM_018908 | 179847 | 182176 | 0.90 | 0.93 | 2.36 | 1.50 |
| 6 | D12 | 56142 | PCDHA6 | NM_018909 | 201611 | 197098 | 1.00 | 1.01 | -1.27 | 0.00 |
| 6 | E01 | NA | neg | NA | 207932 | 197350 | 1.04 | 1.01 | -3.95 | -1.48 |
| 6 | E02 | NA | neg | NA | 200477 | 189315 | 1.00 | 0.97 | -2.42 | 0.10 |
| 6 | E03 | 56141 | PCDHA7 | NM_018910 | 186907 | 194590 | 0.93 | 0.99 | -0.01 | -1.13 |
| 6 | E04 | 56140 | PCDHA8 | NM_018911 | 174179 | 186660 | 0.87 | 0.95 | 3.22 | 0.53 |
| 6 | E05 | 9752 | PCDHA9 | NM_014005 | 178329 | 186907 | 0.89 | 0.96 | 3.95 | 2.71 |
| 6 | E06 | 56135 | PCDHAC1 | NM_018898 | 215938 | 209582 | 1.08 | 1.07 | -5.53 | -3.07 |
| 6 | E07 | 56134 | PCDHAC2 | NM_018899 | 187755 | 186990 | 0.93 | 0.96 | 0.13 | 0.65 |
| 6 | E08 | 29930 | PCDHB1 | NM_013340 | 194157 | 187831 | 0.97 | 0.96 | -1.95 | -0.06 |
| 6 | E09 | 56126 | PCDHB10 | NM_018930 | 183827 | 182053 | 0.92 | 0.93 | 1.73 | 2.18 |
| 6 | E10 | 56125 | PCDHB11 | NM_018931 | 198243 | 202533 | 0.99 | 1.04 | -2.18 | -2.60 |
| 6 | E11 | 56124 | PCDHB12 | NM_018932 | 191790 | 188592 | 0.96 | 0.96 | -0.92 | -0.01 |
| 6 | E12 | 56122 | PCDHB14 | NM_018934 | 191332 | 195842 | 0.95 | 1.00 | 0.00 | 0.00 |
| 6 | F01 | NA | NA | NA | 198554 | 193037 | 0.99 | 0.99 | -2.18 | 0.00 |
| 6 | F02 | NA | pos | NA | 75796 | 75482 | 0.38 | 0.39 | 22.95 | 23.20 |
| 6 | F03 | 56121 | PCDHB15 | NM_018935 | 194781 | 192082 | 0.97 | 0.98 | -1.77 | 0.00 |
| 6 | F04 | 56133 | PCDHB2 | NM_018936 | 189130 | 187276 | 0.94 | 0.96 | 0.01 | 1.04 |
| 6 | F05 | 56132 | PCDHB3 | NM_018937 | 196428 | 195738 | 0.98 | 1.00 | 0.09 | 1.59 |
| 6 | F06 | 56131 | PCDHB4 | NM_018938 | 181609 | 198163 | 0.90 | 1.01 | 1.34 | -0.19 |
| 6 | F07 | 26167 | PCDHB5 | NM_015669 | 187681 | 194592 | 0.93 | 0.99 | -0.01 | -0.22 |
| 6 | F08 | 56130 | PCDHB6 | NM_018939 | 179432 | 198830 | 0.89 | 1.02 | 0.91 | -1.61 |
| 6 | F09 | 26025 | PCDHGA12 | NM_003735 | 195777 | 196211 | 0.97 | 1.00 | -0.87 | 0.01 |
| 6 | F10 | 9708 | PCDHGA8 | NM_014004 | 183566 | 194296 | 0.91 | 0.99 | 0.67 | -0.34 |
| 6 | F11 | 8641 | PCDHGB4 | NM_003736 | 192967 | 186654 | 0.96 | 0.95 | -1.32 | 1.00 |
| 6 | F12 | 5098 | PCDHGC3 | NM_002588 | 191160 | 193893 | 0.95 | 0.99 | -0.12 | 1.02 |
| 6 | G01 | NA | neg | NA | 201176 | 203671 | 1.00 | 1.04 | -3.00 | -3.78 |
| 6 | G02 | NA | neg | NA | 184277 | 193981 | 0.92 | 0.99 | 0.46 | -1.87 |
| 6 | G03 | 5118 | PCOLCE | NM_002593 | 184717 | 192028 | 0.92 | 0.98 | 0.01 | -1.67 |
| 6 | G04 | 5134 | PDCD2 | NM_002598 | 177387 | 184433 | 0.88 | 0.94 | 2.13 | -0.08 |
| 6 | G05 | 10016 | PDCD6 | NM_013232 | 195957 | 199058 | 0.98 | 1.02 | -0.09 | -0.74 |
| 6 | G06 | 9124 | PDLIM1 | NM_020992 | 187148 | 185326 | 0.93 | 0.95 | -0.08 | 0.67 |
| 6 | G07 | 5175 | PECAM1 | NM_000442 | 197723 | 194298 | 0.98 | 0.99 | -2.34 | -1.84 |
| 6 | G08 | 54477 | PEPP2 | NM_019012 | 175108 | 181852 | 0.87 | 0.93 | 1.51 | 0.06 |
| 6 | G09 | 5203 | PFDN4 | NM_002623 | 187968 | 183799 | 0.94 | 0.94 | 0.45 | 0.78 |
| 6 | G10 | 5217 | PFN2 | NM_002628 | 189884 | 181431 | 0.95 | 0.93 | -0.90 | 0.51 |
| 6 | G11 | 1911 | PHC1 | NM_004426 | 185213 | 177063 | 0.92 | 0.90 | -0.01 | 1.22 |
| 6 | G12 | 1912 | PHC2 | NM_004427 | 188922 | 191992 | 0.94 | 0.98 | 0.06 | -0.29 |
| 6 | H01 | NA | NA | NA | 209761 | 198366 | 1.04 | 1.01 | -2.76 | -0.84 |
| 6 | H02 | NA | pos | NA | 88443 | 83845 | 0.44 | 0.43 | 22.08 | 21.77 |
| 6 | H03 | 5253 | PHF2 | NM_005392 | 194470 | 209363 | 0.97 | 1.07 | 0.01 | -3.20 |
| 6 | H04 | 84295 | PHF6 | NM_032335 | 209817 | 191084 | 1.04 | 0.98 | -2.51 | 0.50 |
| 6 | H05 | 8301 | PICALM | NM_007166 | 215538 | 211995 | 1.07 | 1.08 | -2.10 | -1.40 |
| 6 | H06 | 54984 | PINX1 | NM_017884 | 196158 | 197402 | 0.98 | 1.01 | 0.08 | 0.19 |
| 6 | H07 | 10635 | PIR51 | NM_006479 | 195297 | 205787 | 0.97 | 1.05 | 0.15 | -2.21 |
| 6 | H08 | 5310 | PKD1 | NM_000296 | 192296 | 179525 | 0.96 | 0.92 | -0.01 | 2.42 |
| 6 | H09 | 3146 | HMGB1 | NM_002128 | 198456 | 191842 | 0.99 | 0.98 | 0.30 | 1.09 |
| 6 | H10 | 51277 | RBJ | NM_016544 | 198535 | 197580 | 0.99 | 1.01 | -0.68 | -0.77 |
| 6 | H11 | 4489 | MT1A | NM_005946 | 197979 | 193781 | 0.99 | 0.99 | -0.63 | -0.19 |
| 6 | H12 | 4502 | MT2A | NM_005953 | 183044 | 191780 | 0.91 | 0.98 | 3.26 | 1.65 |
| 7 | A01 | NA | pos | NA | 91022 | 74730 | 0.44 | 0.37 | 20.40 | 20.89 |
| 7 | A02 | NA | NA | NA | 184757 | 197364 | 0.90 | 0.97 | 1.41 | -1.77 |
| 7 | A03 | 5325 | PLAGL1 | NM_002656 | 194846 | 183569 | 0.94 | 0.90 | -0.09 | 2.77 |
| 7 | A04 | 59338 | PLEKHA1 | NM_021622 | 188748 | 202732 | 0.92 | 1.00 | 0.62 | -2.43 |
| 7 | A05 | 5360 | PLTP | NM_006227 | 199856 | 192440 | 0.97 | 0.95 | -0.81 | 0.52 |
| 7 | A06 | 5378 | PMS1 | NM_000534 | 185354 | 196736 | 0.90 | 0.97 | 0.85 | -0.38 |
| 7 | A07 | 5395 | PMS2 | NM_000535 | 211847 | 200868 | 1.03 | 0.99 | -4.17 | -1.44 |
| 7 | A08 | 10957 | PNRC1 | NM_006813 | 192909 | 185564 | 0.94 | 0.91 | 0.12 | 0.06 |
| 7 | A09 | 25913 | POT1 | NM_015450 | 191002 | 186078 | 0.93 | 0.92 | -0.02 | -0.10 |
| 7 | A10 | 5514 | PPP1R10 | NM_002714 | 179040 | 177080 | 0.87 | 0.87 | 1.90 | 1.52 |
| 7 | A11 | 26051 | PPP1R16B | NM_015568 | 192998 | 187457 | 0.94 | 0.92 | -0.25 | -0.62 |
| 7 | A12 | 55607 | PPP1R9A | XM_371933 | 191634 | 184118 | 0.93 | 0.91 | 0.01 | 0.24 |
| 7 | B01 | NA | neg | NA | 222786 | 225864 | 1.08 | 1.11 | -4.65 | -6.63 |
| 7 | B02 | NA | neg | NA | 191171 | 219679 | 0.93 | 1.08 | 1.76 | -5.49 |
| 7 | B03 | 59335 | PRDM12 | NM_021619 | 201294 | 185553 | 0.98 | 0.91 | 0.24 | 2.81 |
| 7 | B04 | 63978 | PRDM14 | NM_024504 | 201093 | 191742 | 0.98 | 0.94 | -0.24 | 0.00 |
| 7 | B05 | 11107 | PRDM5 | NM_018699 | 204467 | 198108 | 0.99 | 0.97 | -0.11 | -0.12 |
| 7 | B06 | 11105 | PRDM7 | NM_052996 | 187490 | 201264 | 0.91 | 0.99 | 2.06 | -0.81 |
| 7 | B07 | 56979 | PRDM9 | NM_020227 | 203695 | 196561 | 0.99 | 0.97 | -0.88 | -0.23 |
| 7 | B08 | 10113 | PREB | NM_013388 | 204478 | 200008 | 0.99 | 0.98 | -0.58 | -2.21 |
| 7 | B09 | 5551 | PRF1 | NM_005041 | 198375 | 181715 | 0.96 | 0.89 | 0.13 | 1.11 |
| 7 | B10 | 10394 | PRG3 | NM_006093 | 197794 | 182875 | 0.96 | 0.90 | -0.26 | 0.86 |
| 7 | B11 | 5612 | PRKRIR | NM_004705 | 197118 | 179593 | 0.96 | 0.88 | 0.55 | 1.24 |
| 7 | B12 | 8559 | PRPF18 | NM_003675 | 199316 | 187613 | 0.97 | 0.92 | 0.09 | 0.00 |
| 7 | C01 | NA | pos | NA | 92966 | 90918 | 0.45 | 0.45 | 20.40 | 20.24 |
| 7 | C02 | NA | NA | NA | 187219 | 197753 | 0.91 | 0.97 | 1.31 | 0.49 |
| 7 | C03 | 9128 | PRPF4 | NM_004697 | 196292 | 183390 | 0.95 | 0.90 | 0.01 | 5.14 |
| 7 | C04 | 10594 | PRPF8 | NM_006445 | 182587 | 175861 | 0.89 | 0.87 | 2.26 | 4.87 |
| 7 | C05 | 57716 | PRX | NM_181882 | 211533 | 208912 | 1.03 | 1.03 | -2.79 | -0.19 |
| 7 | C06 | 5663 | PSEN1 | NM_000021 | 190992 | 207892 | 0.93 | 1.02 | 0.11 | -0.11 |
| 7 | C07 | 5708 | PSMD2 | NM_002808 | 193238 | 195525 | 0.94 | 0.96 | 0.00 | 1.89 |
| 7 | C08 | 5829 | PXN | NM_002859 | 198395 | 198830 | 0.96 | 0.98 | -0.60 | -0.06 |

| | | | | | | | | | | |
|---|---|---|---|---|---|---|---|---|---|---|
| 7 | C09 | 25837 | RAB26 | XM_352913 | 178843 | 200840 | 0.87 | 0.99 | 2.84 | -0.49 |
| 7 | C10 | 10981 | RAB32 | NM_006834 | 196949 | 201610 | 0.95 | 0.99 | -1.34 | -0.68 |
| 7 | C11 | 142684 | RAB40A | NM_080879 | 193335 | 190863 | 0.94 | 0.94 | 0.07 | 1.08 |
| 7 | C12 | 10966 | RAB40B | NM_006822 | 212630 | 197826 | 1.03 | 0.97 | -3.85 | 0.04 |
| 7 | D01 | NA | neg | NA | 201154 | 200777 | 0.98 | 0.99 | -0.15 | -1.14 |
| 7 | D02 | NA | neg | NA | 206896 | 199811 | 1.00 | 0.98 | -1.32 | -0.97 |
| 7 | D03 | 57799 | RAB40C | NM_021168 | 193415 | 206126 | 0.94 | 1.01 | 1.95 | -0.14 |
| 7 | D04 | 53916 | RAB4B | NM_016154 | 210309 | 184129 | 1.02 | 0.91 | -2.00 | 2.26 |
| 7 | D05 | 27342 | RABGEF1 | NM_014504 | 201195 | 199648 | 0.98 | 0.98 | 0.67 | 0.45 |
| 7 | D06 | 5884 | RAD17 | NM_002873 | 198321 | 196844 | 0.96 | 0.97 | -0.02 | 0.86 |
| 7 | D07 | 5885 | RAD21 | NM_006265 | 202936 | 206319 | 0.98 | 1.02 | -0.61 | -1.18 |
| 7 | D08 | 5886 | RAD23A | NM_005053 | 210548 | 194234 | 1.02 | 0.96 | -1.70 | -0.28 |
| 7 | D09 | 5887 | RAD23B | NM_002874 | 199492 | 198713 | 0.97 | 0.98 | 0.02 | -1.17 |
| 7 | D10 | 10743 | RAI1 | NM_030665 | 192692 | 195688 | 0.93 | 0.96 | 0.89 | -0.66 |
| 7 | D11 | 5898 | RALA | NM_005402 | 204259 | 190223 | 0.99 | 0.94 | -0.78 | 0.13 |
| 7 | D12 | 5899 | RALB | NM_002881 | 193961 | 181584 | 0.94 | 0.89 | 1.29 | 1.97 |
| 7 | E01 | NA | neg | NA | 208143 | 205675 | 1.01 | 1.01 | -4.57 | -1.84 |
| 7 | E02 | NA | neg | NA | 195561 | 197738 | 0.95 | 0.97 | -2.03 | -0.37 |
| 7 | E03 | 84220 | RANBP2L1 | NM_005054 | 181536 | 206502 | 0.88 | 1.02 | 1.35 | 0.00 |
| 7 | E04 | 8498 | RANBP3 | NM_003624 | 185899 | 197518 | 0.90 | 0.97 | -0.06 | 0.00 |
| 7 | E05 | 11186 | RASSF1 | NM_007182 | 209328 | 201894 | 1.01 | 0.99 | -3.98 | 0.25 |
| 7 | E06 | 83593 | RASSF5 | NM_031437 | 191654 | 202060 | 0.93 | 0.99 | -1.67 | 0.11 |
| 7 | E07 | 5934 | RBL2 | NM_005611 | 174002 | 191811 | 0.84 | 0.94 | 2.25 | 1.71 |
| 7 | E08 | 54033 | RBM11 | NM_144770 | 182231 | 210432 | 0.88 | 1.04 | 1.04 | -3.07 |
| 7 | E09 | 64783 | RBM15 | NM_022768 | 168917 | 178463 | 0.82 | 0.88 | 3.21 | 2.78 |
| 7 | E10 | 10181 | RBM5 | NM_005778 | 182022 | 200520 | 0.88 | 0.99 | 0.04 | -1.34 |
| 7 | E11 | 10180 | RBM6 | NM_005777 | 185926 | 203009 | 0.90 | 1.00 | -0.07 | -2.03 |
| 7 | E12 | 5937 | RBMS1 | NM_002897 | 210514 | 194979 | 1.02 | 0.96 | -5.07 | -0.30 |
| 7 | F01 | NA | NA | NA | 211821 | 208537 | 1.03 | 1.03 | -3.72 | -3.17 |
| 7 | F02 | NA | pos | NA | 84984 | 76256 | 0.41 | 0.38 | 21.98 | 21.28 |
| 7 | F03 | 5939 | RBMS2 | NM_002898 | 196182 | 205309 | 0.95 | 1.01 | -0.01 | -0.57 |
| 7 | F04 | 51742 | ARID4B | NM_016374 | 173299 | 198555 | 0.84 | 0.98 | 4.10 | -0.99 |
| 7 | F05 | 28954 | REM1 | NM_014012 | 194166 | 198265 | 0.94 | 0.98 | 0.69 | 0.12 |
| 7 | F06 | 9185 | REPS2 | NM_004726 | 191235 | 193790 | 0.93 | 0.95 | 0.02 | 0.84 |
| 7 | F07 | 5982 | RFC2 | NM_002914 | 189217 | 195459 | 0.92 | 0.96 | 0.77 | 0.23 |
| 7 | F08 | 5984 | RFC4 | NM_002916 | 195861 | 178127 | 0.95 | 0.88 | -0.12 | 2.10 |
| 7 | F09 | 5992 | RFX4 | NM_002920 | 197912 | 183226 | 0.96 | 0.90 | -1.06 | 1.10 |
| 7 | F10 | 9886 | RHOBTB1 | NM_014836 | 186343 | 189561 | 0.90 | 0.93 | 0.77 | -0.12 |
| 7 | F11 | 23221 | RHOBTB2 | NM_015178 | 197344 | 189888 | 0.96 | 0.93 | -0.78 | -0.40 |
| 7 | F12 | 8572 | PDLIM4 | NM_003687 | 197587 | 197836 | 0.96 | 0.97 | -0.84 | -1.63 |
| 7 | G01 | NA | neg | NA | 216530 | 218619 | 1.05 | 1.08 | -5.08 | -4.89 |
| 7 | G02 | NA | neg | NA | 205588 | 199290 | 1.00 | 0.98 | -2.87 | -1.31 |
| 7 | G03 | 6016 | RIT1 | NM_006912 | 205780 | 207957 | 1.00 | 1.02 | -2.37 | -0.92 |
| 7 | G04 | 10921 | RNPS1 | NM_006711 | 191210 | 191430 | 0.93 | 0.94 | 0.06 | 0.47 |
| 7 | G05 | 10073 | RNUT1 | NM_005701 | 187492 | 212373 | 0.91 | 1.05 | 1.63 | -2.34 |
| 7 | G06 | 6121 | RPE65 | NM_000329 | 199935 | 193881 | 0.97 | 0.95 | -2.16 | 0.96 |
| 7 | G07 | 6103 | RPGR | NM_000328 | 190955 | 204871 | 0.93 | 1.01 | 0.00 | -1.36 |
| 7 | G08 | 10670 | RRAGA | NM_006570 | 187518 | 187801 | 0.91 | 0.92 | 1.15 | 0.46 |
| 7 | G09 | 6247 | RS1 | NM_000330 | 207316 | 189433 | 1.01 | 0.93 | -3.38 | 0.10 |
| 7 | G10 | 6249 | RSN | NM_002956 | 194846 | 184744 | 0.94 | 0.91 | -1.37 | 0.92 |
| 7 | G11 | 864 | RUNX3 | NM_004350 | 189123 | 189189 | 0.92 | 0.93 | 0.47 | -0.13 |
| 7 | G12 | 6271 | S100A1 | NM_006271 | 191441 | 194402 | 0.93 | 0.96 | -0.01 | -0.85 |
| 7 | H01 | NA | NA | NA | 213472 | 210202 | 1.04 | 1.03 | -2.57 | -2.85 |
| 7 | H02 | NA | pos | NA | 84638 | 94058 | 0.41 | 0.46 | 23.53 | 18.61 |
| 7 | H03 | 6282 | S100A11 | NM_005620 | 203780 | 205600 | 0.99 | 1.01 | -0.07 | 0.00 |
| 7 | H04 | 6284 | S100A13 | XM_371380 | 216235 | 219989 | 1.05 | 1.08 | -3.12 | -4.33 |
| 7 | H05 | 57402 | S100A14 | NM_020672 | 204344 | 204809 | 0.99 | 1.01 | 0.11 | -0.47 |
| 7 | H06 | 6273 | S100A2 | NM_005978 | 203844 | 204648 | 0.99 | 1.01 | -1.06 | -0.55 |
| 7 | H07 | 6274 | S100A3 | NM_002960 | 200140 | 197283 | 0.97 | 0.97 | 0.03 | 0.52 |
| 7 | H08 | 6275 | S100A4 | NM_002961 | 201485 | 190675 | 0.98 | 0.94 | 0.21 | 0.41 |
| 7 | H09 | 6279 | S100A8 | NM_002964 | 211645 | 198012 | 1.03 | 0.97 | -2.37 | -1.01 |
| 7 | H10 | 6286 | S100P | NM_005980 | 197633 | 191688 | 0.96 | 0.94 | -0.04 | 0.12 |
| 7 | H11 | 6299 | SALL1 | NM_002968 | 190263 | 186956 | 0.92 | 0.92 | 2.13 | 0.77 |
| 7 | H12 | 27164 | SALL3 | NM_171999 | 195466 | 192389 | 0.95 | 0.95 | 1.06 | 0.00 |
| 8 | A01 | NA | pos | NA | 77229 | 93511 | 0.38 | 0.46 | 22.90 | 17.19 |
| 8 | A02 | NA | NA | NA | 203369 | 200679 | 1.01 | 0.98 | -0.94 | -1.30 |
| 8 | A03 | 57167 | SALL4 | NM_020436 | 201626 | 180936 | 1.00 | 0.89 | -0.76 | 2.32 |
| 8 | A04 | 6310 | SCA1 | NM_000332 | 195356 | 200292 | 0.97 | 0.98 | 0.09 | -1.02 |
| 8 | A05 | 6314 | SCA7 | NM_000333 | 207149 | 199890 | 1.02 | 0.98 | 0.15 | -0.02 |
| 8 | A06 | 7857 | SCG2 | NM_003469 | 202867 | 195599 | 1.00 | 0.96 | -0.40 | -0.05 |
| 8 | A07 | 4250 | SCGB2A2 | NM_002411 | 204608 | 192180 | 1.01 | 0.94 | -0.08 | 0.02 |
| 8 | A08 | 9997 | SCO2 | NM_005138 | 191224 | 187336 | 0.95 | 0.92 | 0.36 | 0.97 |
| 8 | A09 | 6342 | SCP2 | NM_002979 | 202856 | 204398 | 1.00 | 1.00 | -0.42 | -1.99 |
| 8 | A10 | 57758 | SCUBE2 | NM_020974 | 196818 | 185325 | 0.97 | 0.91 | 0.37 | 0.98 |
| 8 | A11 | 6385 | SDC4 | NM_002999 | 202099 | 196944 | 1.00 | 0.96 | -1.12 | -0.96 |
| 8 | A12 | 8436 | SDPR | NM_004657 | 185403 | 178032 | 0.92 | 0.87 | 2.34 | 2.64 |
| 8 | B01 | NA | neg | NA | 238153 | 239844 | 1.18 | 1.17 | -8.28 | -7.42 |
| 8 | B02 | NA | neg | NA | 203325 | 201842 | 1.01 | 0.99 | -1.70 | -0.86 |
| 8 | B03 | 9554 | SEC22L1 | NM_004892 | 197826 | 198098 | 0.98 | 0.97 | -0.80 | 0.00 |
| 8 | B04 | 6401 | SELE | NM_000450 | 198353 | 203156 | 0.98 | 0.99 | -1.24 | -0.88 |
| 8 | B05 | 8991 | SELENBP1 | NM_003944 | 212956 | 206262 | 1.05 | 1.01 | -1.71 | -0.48 |
| 8 | B06 | 6403 | SELP | NM_003005 | 196818 | 191810 | 0.97 | 0.94 | -0.02 | 1.24 |
| 8 | B07 | 57190 | SEPN1 | NM_020451 | 199974 | 195998 | 0.99 | 0.96 | 0.03 | 0.00 |
| 8 | B08 | 1731 | SEPT1 | NM_052838 | 196795 | 203179 | 0.97 | 0.99 | -1.45 | -1.13 |
| 8 | B09 | 55964 | SEPT3 | NM_019106 | 196169 | 192934 | 0.97 | 0.94 | 0.09 | 0.63 |
| 8 | B10 | 23157 | SEPT6 | NM_015129 | 189981 | 192727 | 0.94 | 0.94 | 0.90 | 0.34 |
| 8 | B11 | 10291 | SF3A1 | NM_005877 | 178720 | 182495 | 0.88 | 0.89 | 2.54 | 2.17 |
| 8 | B12 | 10946 | SF3A3 | NM_006802 | 191011 | NA | 0.94 | NA | 0.52 | NA |

| | | | | | | | | | | |
|---|---|---|---|---|---|---|---|---|---|---|
| 8 | C01 | NA | pos | NA | 93404 | 92657 | 0.46 | 0.45 | 20.47 | 19.24 |
| 8 | C02 | NA | NA | NA | 204888 | 199165 | 1.01 | 0.97 | -0.60 | 0.86 |
| 8 | C03 | 6421 | SFPQ | NM_005066 | 198228 | 190772 | 0.98 | 0.93 | 0.51 | 2.52 |
| 8 | C04 | 9295 | SFRS11 | NM_004768 | 216487 | 207822 | 1.07 | 1.02 | -3.27 | -0.42 |
| 8 | C05 | 6427 | SFRS2 | NM_003016 | 204652 | 215093 | 1.01 | 1.05 | 1.25 | -0.75 |
| 8 | C06 | 6428 | SFRS3 | NM_003017 | 191297 | 199438 | 0.95 | 0.98 | 2.41 | 1.18 |
| 8 | C07 | 6432 | SFRS7 | NM_006276 | 200789 | 210305 | 0.99 | 1.03 | 1.27 | -1.21 |
| 8 | C08 | 6433 | SFRS8 | NM_152235 | 192614 | 200101 | 0.95 | 0.98 | 0.73 | 0.66 |
| 8 | C09 | 6441 | SFTPD | NM_003019 | 208866 | 211975 | 1.03 | 1.04 | -0.92 | -1.40 |
| 8 | C10 | 8910 | SGCE | NM_003919 | 205964 | 200985 | 1.02 | 0.98 | -0.73 | 0.18 |
| 8 | C11 | 23013 | SHARP | NM_015001 | 208783 | 196260 | 1.03 | 0.96 | -1.75 | 1.06 |
| 8 | C12 | 6462 | SHBG | NM_001040 | 203964 | 205361 | 1.01 | 1.01 | -0.54 | -0.18 |
| 8 | D01 | NA | neg | NA | 202705 | 203671 | 1.00 | 1.00 | -0.11 | -0.11 |
| 8 | D02 | NA | neg | NA | 205127 | 200373 | 1.01 | 0.98 | -0.57 | 0.46 |
| 8 | D03 | 114132 | SIGLEC11 | NM_052884 | 216693 | 205523 | 1.07 | 1.01 | -2.90 | -0.21 |
| 8 | D04 | 89858 | SIGLECL1 | NM_033329 | 188634 | 200653 | 0.93 | 0.98 | 2.07 | 0.62 |
| 8 | D05 | 25942 | SIN3A | NM_015477 | 222441 | 223556 | 1.10 | 1.09 | -2.03 | -2.40 |
| 8 | D06 | 8487 | SIP1 | NM_003616 | 213111 | 215128 | 1.05 | 1.05 | -1.63 | -1.72 |
| 8 | D07 | 7884 | SLBP | NM_006527 | 203934 | 213405 | 1.01 | 1.04 | 0.75 | -1.93 |
| 8 | D08 | 9351 | SLC9A3R2 | NM_004785 | 184331 | 193729 | 0.91 | 0.95 | 2.37 | 1.57 |
| 8 | D09 | 6601 | SMARCC2 | NM_003075 | 208145 | 202735 | 1.03 | 0.99 | -0.71 | 0.00 |
| 8 | D10 | 10051 | SMC4L1 | NM_005496 | 203255 | 200951 | 1.01 | 0.98 | -0.14 | -0.01 |
| 8 | D11 | 6615 | SNAI1 | NM_005985 | 198224 | 201226 | 0.98 | 0.99 | 0.32 | 0.01 |
| 8 | D12 | 6591 | SNAI2 | NM_003068 | 200998 | 202135 | 0.99 | 0.99 | 0.10 | 0.19 |
| 8 | E01 | NA | neg | NA | 199736 | 206669 | 0.99 | 1.01 | -0.89 | -1.54 |
| 8 | E02 | NA | neg | NA | 201582 | 195688 | 1.00 | 0.96 | -1.24 | 0.35 |
| 8 | E03 | 6619 | SNAPC3 | NM_003084 | 191778 | 198968 | 0.95 | 0.97 | 0.47 | 0.00 |
| 8 | E04 | 6638 | SNRPN | NM_003097 | 190152 | 197324 | 0.94 | 0.97 | 0.44 | 0.28 |
| 8 | E05 | 30837 | SOCS7 | NM_014598 | 192277 | 194388 | 0.95 | 0.95 | 2.33 | 1.72 |
| 8 | E06 | 6651 | SON | NM_003103 | 141096 | 157029 | 0.70 | 0.77 | 10.64 | 7.40 |
| 8 | E07 | 10580 | SORBS1 | NM_006434 | 213816 | 192870 | 1.06 | 0.94 | -2.46 | 0.69 |
| 8 | E08 | 6672 | SP100 | NM_003113 | 192123 | 201562 | 0.95 | 0.99 | -0.44 | -0.69 |
| 8 | E09 | 3431 | SP110 | NM_004509 | 179818 | 197488 | 0.89 | 0.97 | 3.30 | 0.00 |
| 8 | E10 | 121340 | SP7 | NM_152860 | 204745 | 204501 | 1.01 | 1.00 | -1.76 | -1.54 |
| 8 | E11 | 6678 | SPARC | NM_003118 | 205569 | 204678 | 1.02 | 1.00 | -2.41 | -1.50 |
| 8 | E12 | 8404 | SPARCL1 | NM_004684 | 207923 | 211595 | 1.03 | 1.04 | -2.55 | -2.36 |
| 8 | F01 | NA | NA | NA | 194314 | 224957 | 0.96 | 1.10 | 0.67 | -4.95 |
| 8 | F02 | NA | pos | NA | 86092 | 80371 | 0.43 | 0.39 | 21.13 | 20.00 |
| 8 | F03 | 6683 | SPG4 | NM_014946 | 192423 | 204807 | 0.95 | 1.00 | 0.88 | -1.26 |
| 8 | F04 | 8406 | SRPX | NM_006307 | 199861 | 199166 | 0.99 | 0.97 | -0.86 | -0.28 |
| 8 | F05 | 6747 | SSR3 | NM_007107 | 181519 | 196705 | 0.90 | 0.96 | 4.90 | 1.07 |
| 8 | F06 | 6748 | SSR4 | NM_006280 | 192165 | 198121 | 0.95 | 0.97 | 1.52 | 0.05 |
| 8 | F07 | 6749 | SSRP1 | NM_003146 | 203808 | 195439 | 1.01 | 0.96 | -0.03 | 0.00 |
| 8 | F08 | 6759 | SSX4 | NM_005636 | 193915 | 195979 | 0.96 | 0.96 | -0.24 | 0.02 |
| 8 | F09 | 6782 | STCH | NM_006948 | 200582 | 194557 | 0.99 | 0.95 | -0.09 | 0.25 |
| 8 | F10 | 6840 | SVIL | NM_003174 | 198136 | 200637 | 0.98 | 0.98 | 0.02 | -1.12 |
| 8 | F11 | 8189 | SYMPK | NM_004819 | 189165 | 199412 | 0.94 | 0.98 | 1.23 | -0.84 |
| 8 | F12 | 10492 | SYNCRIP | NM_006372 | 197832 | 196479 | 0.98 | 0.96 | -0.10 | 0.00 |
| 8 | G01 | NA | neg | NA | 198884 | 204887 | 0.98 | 1.00 | -0.98 | -1.49 |
| 8 | G02 | NA | neg | NA | 190498 | 214701 | 0.94 | 1.05 | 0.60 | -3.19 |
| 8 | G03 | 51385 | ZNF589 | NM_016089 | 195359 | 204176 | 0.97 | 1.00 | -0.47 | -1.16 |
| 8 | G04 | 51616 | TAF9L | NM_015975 | 189716 | 195778 | 0.94 | 0.96 | 0.27 | 0.29 |
| 8 | G05 | 6886 | TAL1 | NM_003189 | 204044 | 199814 | 1.01 | 0.98 | -0.15 | 0.53 |
| 8 | G06 | 6887 | TAL2 | NM_005421 | 197289 | 200241 | 0.98 | 0.98 | -0.24 | -0.32 |
| 8 | G07 | 10010 | TANK | NM_004180 | 206115 | 192831 | 1.02 | 0.94 | -1.26 | 0.44 |
| 8 | G08 | 6895 | TARBP2 | NM_004178 | 187647 | 197423 | 0.93 | 0.97 | 0.15 | -0.24 |
| 8 | G09 | 8887 | TAX1BP1 | NM_006024 | 187220 | 191545 | 0.93 | 0.94 | 1.65 | 0.76 |
| 8 | G10 | 6923 | TCEB2 | NM_007108 | 179262 | 194059 | 0.89 | 0.95 | 2.80 | 0.01 |
| 8 | G11 | 6941 | TCF19 | NM_007109 | 185216 | 194551 | 0.92 | 0.95 | 1.18 | -0.01 |
| 8 | G12 | 6934 | TCF7L2 | NM_030756 | 198497 | 202677 | 0.98 | 0.99 | -1.02 | -1.08 |
| 8 | H01 | NA | NA | NA | 214395 | 224503 | 1.06 | 1.10 | -1.23 | -3.39 |
| 8 | H02 | NA | pos | NA | 84148 | 90667 | 0.42 | 0.44 | 23.39 | 19.70 |
| 8 | H03 | 10732 | TCFL5 | NM_006602 | 193568 | 189886 | 0.96 | 0.93 | 2.56 | 2.80 |
| 8 | H04 | 6950 | TCP1 | NM_030752 | 205864 | 196187 | 1.02 | 0.96 | -0.09 | 1.71 |
| 8 | H05 | 7013 | TERF1 | NM_003218 | 223175 | 211359 | 1.10 | 1.03 | -1.08 | 0.02 |
| 8 | H06 | 7014 | TERF2 | NM_005652 | 210152 | 219897 | 1.04 | 1.08 | 0.02 | -2.23 |
| 8 | H07 | 54386 | TERF2IP | NM_018975 | 201430 | 209297 | 1.00 | 1.02 | 2.32 | -0.91 |
| 8 | H08 | 7018 | TF | NM_001063 | 203463 | 204755 | 1.01 | 1.00 | -0.15 | -0.02 |
| 8 | H09 | 7072 | TIA1 | NM_022037 | 209231 | 206094 | 1.04 | 1.01 | 0.18 | -0.26 |
| 8 | H10 | 8914 | TIMELESS | NM_003920 | 208448 | 208348 | 1.03 | 1.02 | -0.02 | -0.97 |
| 8 | H11 | 25976 | TIPARP | NM_015508 | 207423 | 191075 | 1.03 | 0.94 | -0.32 | 2.07 |
| 8 | H12 | 7123 | TNA | NM_003278 | 192518 | 193367 | 0.95 | 0.95 | 2.80 | 2.02 |
| 9 | A01 | NA | pos | NA | 93687 | 93154 | 0.45 | 0.45 | 17.69 | 17.72 |
| 9 | A02 | NA | NA | NA | 197560 | 182328 | 0.95 | 0.88 | -0.58 | 2.69 |
| 9 | A03 | 3371 | TNC | NM_002160 | 193543 | 206398 | 0.93 | 1.00 | 0.09 | -0.64 |
| 9 | A04 | 7130 | TNFAIP6 | NM_007115 | 192501 | 198776 | 0.92 | 0.96 | -0.11 | -0.12 |
| 9 | A05 | 10318 | TNIP1 | NM_006058 | 195696 | 202587 | 0.94 | 0.98 | 0.90 | 0.92 |
| 9 | A06 | 7134 | TNNC1 | NM_003280 | 180774 | 198905 | 0.87 | 0.96 | 3.44 | 0.08 |
| 9 | A07 | 7135 | TNNI1 | NM_003281 | 206727 | 203753 | 0.99 | 0.99 | -0.57 | -1.14 |
| 9 | A08 | 7148 | TNXB | NM_019105 | 196110 | 199097 | 0.94 | 0.96 | -1.09 | -0.12 |
| 9 | A09 | 7184 | TRA1 | NM_003299 | 195846 | 195575 | 0.94 | 0.95 | -0.23 | 0.28 |
| 9 | A10 | 9322 | TRIP10 | NM_004240 | 186916 | 189913 | 0.90 | 0.92 | 0.22 | 0.32 |
| 9 | A11 | 7216 | TRO | NM_016157 | 196525 | 196177 | 0.94 | 0.95 | -0.14 | -0.12 |
| 9 | A12 | 10024 | TROAP | NM_005480 | 187978 | 192439 | 0.90 | 0.93 | 0.98 | 1.39 |
| 9 | B01 | NA | neg | NA | 222363 | 222908 | 1.07 | 1.08 | -4.37 | -5.06 |
| 9 | B02 | NA | neg | NA | 200377 | 200275 | 0.96 | 0.97 | -0.50 | -1.25 |
| 9 | B03 | 10083 | USH1C | NM_005709 | 200017 | 203332 | 0.96 | 0.98 | -0.47 | -1.04 |
| 9 | B04 | 7412 | VCAM1 | NM_001078 | 196566 | 192392 | 0.94 | 0.93 | -0.25 | 0.05 |

| | | | | | | | | | | |
|---|---|---|---|---|---|---|---|---|---|---|
| 9 | B05 | 7429 | VIL1 | NM_007127 | 210219 | 201926 | 1.01 | 0.98 | -1.09 | 0.12 |
| 9 | B06 | 7430 | VIL2 | NM_003379 | 217589 | 204007 | 1.04 | 0.99 | -2.46 | -1.69 |
| 9 | B07 | 7441 | VPREB1 | NM_007128 | 205202 | 191938 | 0.98 | 0.93 | 0.27 | -0.06 |
| 9 | B08 | 7448 | VTN | NM_000638 | 187944 | 189368 | 0.90 | 0.92 | 0.92 | 0.61 |
| 9 | B09 | 7450 | VWF | NM_000552 | 196744 | 185446 | 0.94 | 0.90 | 0.19 | 1.08 |
| 9 | B10 | 8936 | WASF1 | NM_003931 | 189618 | 179752 | 0.91 | 0.87 | 0.32 | 1.12 |
| 9 | B11 | 10810 | WASF3 | NM_006646 | 190919 | 197051 | 0.92 | 0.95 | 1.42 | -1.18 |
| 9 | B12 | 7456 | WASPIP | NM_003387 | 209961 | 195602 | 1.01 | 0.95 | -2.32 | -0.05 |
| 9 | C01 | NA | pos | NA | 93812 | 92900 | 0.45 | 0.45 | 18.97 | 17.86 |
| 9 | C02 | NA | NA | NA | 194910 | 205245 | 0.93 | 0.99 | 1.18 | -1.07 |
| 9 | C03 | 51085 | WBSCR14 | NM_032951 | 199815 | 203545 | 0.96 | 0.99 | 0.28 | -0.06 |
| 9 | C04 | 64393 | WIG1 | NM_022470 | 188001 | 196743 | 0.90 | 0.95 | 1.98 | 0.33 |
| 9 | C05 | 8840 | WISP1 | NM_003882 | 190578 | 211262 | 0.91 | 1.02 | 3.09 | -0.44 |
| 9 | C06 | 8839 | WISP2 | NM_003881 | 200766 | 199489 | 0.96 | 0.97 | 1.22 | 0.08 |
| 9 | C07 | 7507 | XPA | NM_000380 | 212364 | 201081 | 1.02 | 0.97 | -0.27 | -0.59 |
| 9 | C08 | 7515 | XRCC1 | NM_006297 | 209123 | 213990 | 1.00 | 1.04 | -2.08 | -2.52 |
| 9 | C09 | 10413 | YAP1 | NM_006106 | 193446 | 189065 | 0.93 | 0.92 | 1.49 | 1.48 |
| 9 | C10 | 7531 | YWHAE | NM_006761 | 204519 | 192104 | 0.98 | 0.93 | -1.58 | 0.06 |
| 9 | C11 | 7534 | YWHAZ | NM_003406 | 205102 | 195351 | 0.98 | 0.95 | -0.35 | 0.12 |
| 9 | C12 | 7455 | ZAN | NM_003386 | 211129 | 205119 | 1.01 | 0.99 | -1.80 | -0.64 |
| 9 | D01 | NA | neg | NA | 211947 | 217089 | 1.02 | 1.05 | -2.06 | -2.91 |
| 9 | D02 | NA | neg | NA | 205319 | 203310 | 0.98 | 0.98 | -0.90 | -0.59 |
| 9 | D03 | 58487 | ZF | NM_021212 | 202216 | 196386 | 0.97 | 0.95 | -0.38 | 1.31 |
| 9 | D04 | 7541 | ZFP161 | NM_003409 | 181320 | 206728 | 0.87 | 1.00 | 2.91 | -1.19 |
| 9 | D05 | 55734 | ZFP64 | NM_018197 | 205166 | 191709 | 0.98 | 0.93 | 0.28 | 3.02 |
| 9 | D06 | 23414 | ZFPM2 | NM_012082 | 207310 | 200121 | 0.99 | 0.97 | -0.17 | 0.14 |
| 9 | D07 | 7546 | ZIC2 | NM_007129 | 182990 | 206074 | 0.88 | 1.00 | 4.66 | -1.27 |
| 9 | D08 | 7547 | ZIC3 | NM_003413 | 194913 | 200958 | 0.93 | 0.97 | 0.18 | -0.17 |
| 9 | D09 | 7556 | ZNF10 | NM_015394 | 203212 | 189632 | 0.97 | 0.92 | -0.47 | 1.54 |
| 9 | D10 | 7678 | ZNF124 | NM_003431 | 201058 | 186677 | 0.96 | 0.90 | -1.21 | 1.13 |
| 9 | D11 | 7705 | ZNF146 | NM_007145 | 192924 | 198663 | 0.92 | 0.96 | 1.54 | -0.28 |
| 9 | D12 | 7709 | ZNF151 | NM_003443 | 206809 | 209313 | 0.99 | 1.01 | -1.28 | -1.19 |
| 9 | E01 | NA | neg | NA | 226596 | 202294 | 1.09 | 0.98 | -5.74 | -0.94 |
| 9 | E02 | NA | neg | NA | 194777 | 216699 | 0.93 | 1.05 | -0.15 | -3.37 |
| 9 | E03 | 90338 | ZNF160 | NM_033288 | 190935 | 198957 | 0.92 | 0.96 | 0.50 | 0.35 |
| 9 | E04 | 7750 | ZNF198 | NM_003453 | 190925 | 191769 | 0.92 | 0.93 | 0.11 | 0.80 |
| 9 | E05 | 7752 | ZNF200 | NM_003454 | 191350 | 213776 | 0.92 | 1.03 | 1.61 | -1.23 |
| 9 | E06 | 7570 | ZNF22 | NM_006963 | 214677 | 192250 | 1.03 | 0.93 | -2.57 | 0.94 |
| 9 | E07 | 10472 | ZNF238 | NM_006352 | 212287 | 194078 | 1.02 | 0.94 | -1.60 | 0.22 |
| 9 | E08 | 23598 | ZNF278 | NM_014323 | 208421 | 187796 | 1.00 | 0.91 | -3.30 | 1.52 |
| 9 | E09 | 51711 | ZNF325 | NM_016265 | 178686 | 207839 | 0.86 | 1.01 | 2.74 | -2.05 |
| 9 | E10 | 84671 | ZNF347 | NM_032584 | 189168 | 195210 | 0.91 | 0.94 | -0.22 | -0.84 |
| 9 | E11 | 171017 | ZNF384 | NM_133476 | 202652 | 201120 | 0.97 | 0.97 | -1.27 | -1.22 |
| 9 | E12 | 7594 | ZNF43 | NM_003423 | 186690 | 200368 | 0.89 | 0.97 | 1.15 | -0.21 |
| 9 | F01 | NA | NA | NA | 219157 | 214537 | 1.05 | 1.04 | -2.72 | -2.80 |
| 9 | F02 | NA | pos | NA | 83377 | 84609 | 0.40 | 0.41 | 21.17 | 19.09 |
| 9 | F03 | 51710 | ZNF44 | NM_016264 | 208950 | 201864 | 1.00 | 0.98 | -0.95 | 0.06 |
| 9 | F04 | 10773 | ZNF482 | NM_006626 | 203310 | 199183 | 0.97 | 0.96 | -0.35 | -0.25 |
| 9 | F05 | 199692 | ZNF627 | NM_145295 | 211855 | 208413 | 1.02 | 1.01 | -0.28 | -0.12 |
| 9 | F06 | 7625 | ZNF74 | NM_003426 | 208816 | 199484 | 1.00 | 0.97 | 0.17 | -0.08 |
| 9 | F07 | 7629 | ZNF76 | NM_003427 | 211304 | 196214 | 1.01 | 0.95 | 0.29 | 0.06 |
| 9 | F08 | 7637 | ZNF84 | NM_003428 | 200404 | 187471 | 0.96 | 0.91 | -0.18 | 1.78 |
| 9 | F09 | 10009 | ZBTB33 | NM_006777 | 195795 | 198514 | 0.94 | 0.96 | 1.45 | -0.28 |
| 9 | F10 | 10320 | ZNFN1A1 | NM_006060 | 193934 | 195344 | 0.93 | 0.95 | 0.65 | -0.66 |
| 9 | F11 | 22807 | ZNFN1A2 | NM_016260 | 193152 | 192965 | 0.93 | 0.93 | 2.12 | 0.36 |
| 9 | F12 | 9183 | ZW10 | NM_004724 | 203763 | 192431 | 0.98 | 0.93 | -0.14 | 1.33 |
| 9 | G01 | NA | neg | NA | 218228 | 197549 | 1.05 | 0.96 | -3.11 | -0.93 |
| 9 | G02 | NA | neg | NA | 204261 | 209934 | 0.98 | 1.02 | -0.65 | -3.02 |
| 9 | G03 | 7791 | ZYX | NM_003461 | 200901 | 206819 | 0.96 | 1.00 | -0.09 | -1.77 |
| 9 | G04 | 307 | ANXA4 | NM_001153 | 188255 | 192074 | 0.90 | 0.93 | 1.75 | -0.04 |
| 9 | G05 | 308 | ANXA5 | NM_001154 | 209200 | 192978 | 1.00 | 0.93 | -0.37 | 1.49 |
| 9 | G06 | 317 | APAF1 | NM_001160 | 203672 | 222146 | 0.98 | 1.08 | 0.53 | -4.89 |
| 9 | G07 | 334 | APLP2 | NM_001642 | 213178 | 189241 | 1.02 | 0.92 | -0.60 | 0.25 |
| 9 | G08 | 351 | APP | NM_000484 | 185574 | 191461 | 0.89 | 0.93 | 1.88 | 0.12 |
| 9 | G09 | 396 | ARHGDIA | NM_004309 | 205029 | 198238 | 0.98 | 0.96 | -0.73 | -1.22 |
| 9 | G10 | 9181 | ARHGEF2 | NM_004723 | 187966 | 191893 | 0.90 | 0.93 | 1.15 | -1.07 |
| 9 | G11 | 399 | RHOH | NM_004310 | 209636 | 186738 | 1.00 | 0.90 | -1.34 | 0.41 |
| 9 | G12 | 402 | ARL2 | NM_001667 | 199088 | 194134 | 0.95 | 0.94 | 0.14 | 0.05 |
| 9 | H01 | NA | NA | NA | 216851 | 213029 | 1.04 | 1.03 | -1.49 | -2.31 |
| 9 | H02 | NA | pos | NA | 86511 | 88371 | 0.41 | 0.43 | 21.44 | 18.69 |
| 9 | H03 | 29108 | ASC | NM_013258 | 204662 | 194927 | 0.98 | 0.94 | 0.62 | 1.46 |
| 9 | H04 | 332 | BIRC5 | NM_001168 | 207331 | 198871 | 0.99 | 0.96 | -0.24 | 0.04 |
| 9 | H05 | 1104 | CHC1 | NM_001269 | 233290 | 211685 | 1.12 | 1.02 | -3.23 | -0.44 |
| 9 | H06 | 1102 | CHC1L | NM_001268 | 216888 | 207625 | 1.04 | 1.00 | -0.43 | -1.22 |
| 9 | H07 | 8853 | DDEF2 | NM_003887 | 198797 | 193116 | 0.95 | 0.93 | 3.31 | 0.82 |
| 9 | H08 | 10395 | DLC1 | NM_006094 | 202656 | 204366 | 0.97 | 0.99 | 0.25 | -0.83 |
| 9 | H09 | 10160 | FARP1 | NM_005766 | 209756 | 215286 | 1.01 | 1.04 | -0.19 | -2.87 |
| 9 | H10 | 2245 | FGD1 | NM_004463 | 204588 | 193186 | 0.98 | 0.93 | -0.40 | -0.06 |
| 9 | H11 | 23637 | RABGAP1 | NM_012197 | 209027 | 192111 | 1.00 | 0.93 | 0.14 | 0.74 |
| 9 | H12 | 6606 | SMN1 | NM_000344 | 184922 | 195844 | 0.89 | 0.95 | 4.00 | 0.99 |
| 10 | A01 | NA | pos | NA | 97253 | 86966 | 0.47 | 0.40 | 19.89 | 21.72 |
| 10 | A02 | NA | NA | NA | 198788 | 191323 | 0.96 | 0.88 | -0.91 | 0.84 |
| 10 | A03 | 2646 | GCKR | NM_001486 | 195155 | 191163 | 0.94 | 0.88 | -1.53 | 2.14 |
| 10 | A04 | 9771 | RAPGEF5 | NM_012294 | 192929 | 200488 | 0.93 | 0.92 | -0.04 | -0.34 |
| 10 | A05 | 2889 | RAPGEF1 | NM_005312 | 197138 | 196362 | 0.95 | 0.90 | 0.02 | 0.13 |
| 10 | A06 | 2979 | GUCA1B | NM_002098 | 198456 | 199313 | 0.96 | 0.91 | -0.50 | -0.65 |
| 10 | A07 | 3273 | HRG | NM_000412 | 191570 | 179872 | 0.93 | 0.82 | 0.50 | 3.74 |
| 10 | A08 | 3316 | HSPB2 | NM_001541 | 202545 | 195024 | 0.98 | 0.89 | -1.61 | -0.13 |

| | | | | | | | | | | |
|---|---|---|---|---|---|---|---|---|---|---|
| 10 | A09 | 3320 | HSPCA | NM_005348 | 197575 | 195344 | 0.96 | 0.90 | -1.01 | -1.18 |
| 10 | A10 | 3326 | HSPCB | NM_007355 | 190456 | 192231 | 0.92 | 0.88 | 0.58 | -0.64 |
| 10 | A11 | 3486 | IGFBP3 | NM_000598 | 186940 | 181671 | 0.90 | 0.83 | 2.24 | 1.81 |
| 10 | A12 | 8826 | IQGAP1 | NM_003870 | 187919 | 170547 | 0.91 | 0.78 | 1.59 | 4.90 |
| 10 | B01 | NA | neg | NA | 223789 | 231654 | 1.08 | 1.06 | -5.73 | -7.20 |
| 10 | B02 | NA | neg | NA | 206914 | 206929 | 1.00 | 0.95 | -2.28 | -2.26 |
| 10 | B03 | 10788 | IQGAP2 | NM_006633 | 197700 | 207590 | 0.96 | 0.95 | -1.75 | -1.12 |
| 10 | B04 | 6453 | ITSN1 | NM_003024 | 198523 | 202198 | 0.96 | 0.93 | -0.89 | -0.66 |
| 10 | B05 | 3827 | KNG1 | NM_000893 | 204381 | 200957 | 0.99 | 0.92 | -1.16 | -0.77 |
| 10 | B06 | 91662 | NALP12 | NM_033297 | 201112 | 191215 | 0.97 | 0.88 | -0.74 | 0.99 |
| 10 | B07 | 51735 | RAPGEF6 | NM_016340 | 194374 | 187108 | 0.94 | 0.86 | 0.23 | 2.32 |
| 10 | B08 | 84687 | PPP1R9B | NM_032595 | 196122 | 189051 | 0.95 | 0.87 | 0.00 | 1.08 |
| 10 | B09 | 5523 | PPP2R3A | NM_002718 | 190436 | 190731 | 0.92 | 0.87 | 0.76 | -0.23 |
| 10 | B10 | 5575 | PRKAR1B | NM_002735 | 190110 | 191114 | 0.92 | 0.88 | 0.95 | -0.39 |
| 10 | B11 | 5660 | PSAP | NM_002778 | 199348 | 185594 | 0.96 | 0.85 | 0.00 | 1.05 |
| 10 | B12 | 8437 | RASAL1 | NM_004658 | 190723 | 193962 | 0.92 | 0.89 | 1.32 | 0.24 |
| 10 | C01 | NA | pos | NA | 102646 | 90920 | 0.50 | 0.42 | 19.40 | 21.47 |
| 10 | C02 | NA | NA | NA | 200719 | 201280 | 0.97 | 0.92 | -0.69 | -0.60 |
| 10 | C03 | 10125 | RASGRP1 | NM_005739 | 186734 | 193050 | 0.90 | 0.88 | 0.81 | 2.31 |
| 10 | C04 | 10235 | RASGRP2 | NM_005825 | 189103 | 199072 | 0.91 | 0.91 | 1.36 | 0.50 |
| 10 | C05 | 115727 | RASGRP4 | NM_052949 | 203416 | 211244 | 0.98 | 0.97 | -0.65 | -2.30 |
| 10 | C06 | 5981 | RFC1 | NM_002913 | 198931 | 210239 | 0.96 | 0.96 | 0.02 | -2.29 |
| 10 | C07 | 9610 | RIN1 | NM_004292 | 196081 | 201211 | 0.95 | 0.92 | 0.19 | 0.02 |
| 10 | C08 | 6285 | S100B | NM_006272 | 203473 | 192580 | 0.98 | 0.88 | -1.19 | 0.91 |
| 10 | C09 | 6295 | SAG | NM_000541 | 198325 | 191032 | 0.96 | 0.88 | -0.54 | 0.23 |
| 10 | C10 | 5265 | SERPINA1 | NM_000295 | 212877 | 191886 | 1.03 | 0.88 | -3.40 | -0.02 |
| 10 | C11 | 866 | SERPINA6 | NM_001756 | 196563 | 197018 | 0.95 | 0.90 | 0.88 | -0.71 |
| 10 | C12 | 6906 | SERPINA7 | NM_000354 | 198777 | 199050 | 0.96 | 0.91 | -0.02 | -0.25 |
| 10 | D01 | NA | neg | NA | 221624 | 224091 | 1.07 | 1.03 | -3.80 | -5.04 |
| 10 | D02 | NA | neg | NA | 196031 | 200332 | 0.95 | 0.92 | 1.44 | -0.29 |
| 10 | D03 | 5269 | SERPINB6 | NM_004568 | 197461 | 205170 | 0.95 | 0.94 | -0.21 | 0.01 |
| 10 | D04 | 5272 | SERPINB9 | NM_004155 | 201241 | 193632 | 0.97 | 0.89 | 0.04 | 1.71 |
| 10 | D05 | 462 | SERPINC1 | NM_000488 | 205261 | 201015 | 0.99 | 0.92 | 0.15 | -0.13 |
| 10 | D06 | 3053 | SERPIND1 | NM_000185 | 201199 | 197249 | 0.97 | 0.90 | 0.73 | 0.43 |
| 10 | D07 | 5270 | SERPINE2 | NM_006216 | 205605 | 202040 | 0.99 | 0.93 | -0.59 | -0.02 |
| 10 | D08 | 5345 | SERPINF2 | NM_000934 | 174769 | 189522 | 0.85 | 0.87 | 5.87 | 1.64 |
| 10 | D09 | 871 | SERPINH1 | NM_001235 | 201693 | 204870 | 0.98 | 0.94 | -0.06 | -2.41 |
| 10 | D10 | 6418 | SET | NM_003011 | 204135 | 195083 | 0.99 | 0.89 | -0.43 | -0.54 |
| 10 | D11 | 2810 | SFN | NM_006142 | 210109 | 194580 | 1.02 | 0.89 | -0.72 | -0.10 |
| 10 | D12 | 6447 | SGNE1 | NM_003020 | 201278 | 195992 | 0.97 | 0.90 | 0.64 | 0.48 |
| 10 | E01 | NA | neg | NA | 209441 | 222461 | 1.01 | 1.02 | -3.31 | -4.77 |
| 10 | E02 | NA | neg | NA | 182753 | 198023 | 0.88 | 0.91 | 2.16 | 0.12 |
| 10 | E03 | 6494 | SIPA1 | NM_006747 | 191250 | 199992 | 0.92 | 0.92 | -0.94 | 0.99 |
| 10 | E04 | 6654 | SOS1 | NM_0055633 | 190805 | 200220 | 0.92 | 0.92 | 0.18 | 0.34 |
| 10 | E05 | 56882 | SPEC1 | NM_020239 | 181568 | 193209 | 0.88 | 0.89 | 3.00 | 1.38 |
| 10 | E06 | 7249 | TSC2 | NM_000548 | 194960 | 202501 | 0.94 | 0.93 | 0.01 | -0.67 |
| 10 | E07 | 7410 | VAV2 | NM_003371 | 193009 | 202755 | 0.93 | 0.93 | -0.01 | -0.22 |
| 10 | E08 | 8976 | WASL | NM_003941 | 185816 | 202019 | 0.90 | 0.93 | 1.60 | -0.91 |
| 10 | E09 | 8892 | EIF2B2 | NM_014239 | 190414 | 190861 | 0.92 | 0.87 | 0.25 | 0.34 |
| 10 | E10 | 2909 | GRLF1 | NM_024342 | 200968 | 191045 | 0.97 | 0.88 | -1.79 | 0.22 |
| 10 | E11 | 9232 | PTTG1 | NM_004219 | 208234 | 198926 | 1.01 | 0.91 | -2.34 | -1.02 |
| 10 | E12 | 7291 | TWIST1 | NM_000474 | 211991 | 199510 | 1.02 | 0.91 | -3.56 | -0.27 |
| 10 | F01 | NA | NA | NA | 220034 | 215691 | 1.06 | 0.99 | -3.93 | -3.84 |
| 10 | F02 | NA | pos | NA | 75937 | 80811 | 0.37 | 0.37 | 25.59 | 23.14 |
| 10 | F03 | 7409 | VAV1 | NM_005428 | 193172 | 212193 | 0.93 | 0.97 | 0.21 | -1.87 |
| 10 | F04 | 9839 | ZFHX1B | NM_014795 | 195783 | 193177 | 0.95 | 0.89 | 0.71 | 1.32 |
| 10 | F05 | 26574 | AATF | NM_012138 | 203899 | 193565 | 0.99 | 0.89 | -0.02 | 0.88 |
| 10 | F06 | 29777 | ABT1 | NM_013375 | 202599 | 197067 | 0.98 | 0.90 | -0.01 | -0.01 |
| 10 | F07 | 27125 | AF5Q31 | NM_014423 | 207975 | 215581 | 1.01 | 0.99 | -1.52 | -3.21 |
| 10 | F08 | 11036 | ALF | NM_006872 | 194187 | 194867 | 0.94 | 0.89 | 1.44 | 0.09 |
| 10 | F09 | 257 | ALX3 | NM_006492 | 184243 | 188256 | 0.89 | 0.86 | 3.06 | 0.44 |
| 10 | F10 | 60529 | ALX4 | NM_021926 | 206632 | 189933 | 1.00 | 0.87 | -1.40 | 0.02 |
| 10 | F11 | 170302 | ARX | NM_139058 | 204362 | 204171 | 0.99 | 0.94 | 0.00 | -2.49 |
| 10 | F12 | 429 | ASCL1 | NM_004316 | 203037 | 196683 | 0.98 | 0.90 | -0.17 | -0.13 |
| 10 | G01 | NA | neg | NA | 206735 | 233089 | 1.00 | 1.07 | -2.79 | -7.33 |
| 10 | G02 | NA | neg | NA | 194004 | 213834 | 0.94 | 0.98 | -0.18 | -3.48 |
| 10 | G03 | 55870 | ASH1L | NM_018489 | 182556 | 202858 | 0.88 | 0.93 | 0.80 | -0.01 |
| 10 | G04 | 463 | ATBF1 | NM_006885 | 197854 | 210958 | 0.96 | 0.97 | -1.30 | -2.24 |
| 10 | G05 | 466 | ATF1 | NM_005171 | 191651 | 209400 | 0.93 | 0.96 | 0.90 | -2.29 |
| 10 | G06 | 467 | ATF3 | NM_001674 | 193453 | 196936 | 0.94 | 0.90 | 0.28 | 0.01 |
| 10 | G07 | 468 | ATF4 | NM_001675 | 201995 | 200977 | 0.98 | 0.92 | -1.88 | -0.29 |
| 10 | G08 | 22809 | ATF5 | NM_012068 | 193760 | 196929 | 0.94 | 0.90 | -0.06 | -0.33 |
| 10 | G09 | 22926 | ATF6 | NM_007348 | 191172 | 184683 | 0.92 | 0.85 | 0.06 | 1.14 |
| 10 | G10 | 11016 | ATF7 | NM_006856 | 188734 | 184470 | 0.91 | 0.85 | 0.68 | 1.10 |
| 10 | G11 | 571 | BACH1 | NM_001186 | 201656 | 191178 | 0.98 | 0.88 | -1.03 | 0.10 |
| 10 | G12 | 579 | BAPX1 | NM_001189 | 211332 | 195312 | 1.02 | 0.90 | -3.46 | 0.13 |
| 10 | H01 | NA | NA | NA | 215679 | 226207 | 1.04 | 1.04 | -2.95 | -6.79 |
| 10 | H02 | NA | pos | NA | 85106 | 90077 | 0.41 | 0.41 | 23.80 | 20.44 |
| 10 | H03 | 56033 | BARX1 | NM_021570 | 189547 | 209752 | 0.92 | 0.96 | 1.04 | -2.23 |
| 10 | H04 | 10538 | BATF | NM_006399 | 202962 | 213773 | 0.98 | 0.98 | -0.68 | -3.65 |
| 10 | H05 | 9031 | BAZ1B | NM_023005 | 208520 | 188929 | 1.01 | 0.87 | -0.89 | 0.96 |
| 10 | H06 | 604 | BCL6 | NM_001706 | 210455 | 190483 | 1.02 | 0.87 | -1.53 | 0.46 |
| 10 | H07 | 8553 | BHLHB2 | NM_003670 | 200905 | 194824 | 0.97 | 0.89 | 0.01 | 0.10 |
| 10 | H08 | 646 | BNC1 | NM_001717 | 201625 | 191566 | 0.97 | 0.88 | 0.00 | -0.09 |
| 10 | H09 | 675 | BRCA2 | NM_000059 | 210964 | 189894 | 1.02 | 0.87 | -2.33 | -0.74 |
| 10 | H10 | 10902 | BRD8 | NM_006696 | 198103 | 180727 | 0.96 | 0.83 | 0.43 | 1.01 |
| 10 | H11 | 2972 | BRF1 | NM_001519 | 192214 | 180798 | 0.93 | 0.83 | 2.58 | 1.33 |
| 10 | H12 | 687 | BTEB1 | NM_001206 | 202499 | 198033 | 0.98 | 0.91 | 0.02 | -1.25 |

| | | | | | | | | | | |
|---|---|---|---|---|---|---|---|---|---|---|
| 11 | A01 | NA | pos | NA | 104270 | 78865 | 0.43 | 0.34 | 19.67 | 28.64 |
| 11 | A02 | NA | NA | NA | 279006 | 261969 | 1.16 | 1.13 | -6.21 | -5.09 |
| 11 | A03 | 9774 | BCLAF1 | NM_014739 | 239521 | 230338 | 1.00 | 0.99 | 0.14 | 0.71 |
| 11 | A04 | 690 | BTF3L1 | NM_001208 | 227699 | 243156 | 0.95 | 1.05 | 3.11 | 0.09 |
| 11 | A05 | 694 | BTG1 | NM_001731 | 247807 | 234638 | 1.03 | 1.01 | -1.01 | -0.02 |
| 11 | A06 | 7832 | BTG2 | NM_006763 | 240361 | 232984 | 1.00 | 1.00 | 0.68 | 0.34 |
| 11 | A07 | 811 | CALR | NM_004343 | 249447 | 232293 | 1.04 | 1.00 | -1.32 | -0.73 |
| 11 | A08 | 8092 | CART1 | NM_006982 | 233115 | 228610 | 0.97 | 0.98 | 0.06 | 0.55 |
| 11 | A09 | 862 | CBFA2T1 | NM_004349 | 227749 | 229270 | 0.95 | 0.99 | -0.93 | -0.78 |
| 11 | A10 | 9139 | CBFA2T2 | NM_005093 | 236554 | 236850 | 0.98 | 1.02 | -0.65 | 0.02 |
| 11 | A11 | 863 | CBFA2T3 | NM_005187 | 230241 | 228609 | 0.96 | 0.98 | -0.06 | -1.18 |
| 11 | A12 | 865 | CBFB | NM_001755 | 230835 | 262827 | 0.96 | 1.13 | 0.43 | -3.87 |
| 11 | B01 | NA | neg | NA | 248010 | 268499 | 1.03 | 1.16 | -3.00 | -7.61 |
| 11 | B02 | NA | neg | NA | 247320 | 247777 | 1.03 | 1.07 | -2.90 | -3.80 |
| 11 | B03 | 8535 | CBX4 | NM_003655 | 221719 | 233242 | 0.92 | 1.00 | 1.39 | -1.15 |
| 11 | B04 | 1044 | CDX1 | NM_001804 | 248442 | 244512 | 1.03 | 1.05 | -1.35 | -1.48 |
| 11 | B05 | 1045 | CDX2 | NM_001265 | 232029 | 229363 | 0.96 | 0.99 | -0.06 | -0.37 |
| 11 | B06 | 1046 | CDX4 | NM_005193 | 235570 | 213570 | 0.98 | 0.92 | 0.00 | 2.59 |
| 11 | B07 | 1050 | CEBPA | NM_004364 | 219701 | 214350 | 0.91 | 0.92 | 1.70 | 1.26 |
| 11 | B08 | 1051 | CEBPB | NM_005194 | 224179 | 224024 | 0.93 | 0.96 | 0.00 | 0.08 |
| 11 | B09 | 1112 | CHES1 | NM_005197 | 222443 | 230487 | 0.92 | 0.99 | -1.53 | -2.32 |
| 11 | B10 | 4435 | CITED1 | NM_004143 | 227674 | 219005 | 0.95 | 0.94 | -0.72 | 1.99 |
| 11 | B11 | 10370 | CITED2 | NM_006079 | 196369 | 200956 | 0.82 | 0.87 | 3.56 | 2.59 |
| 11 | B12 | 1161 | CKN1 | NM_000082 | 220235 | 234990 | 0.92 | 1.01 | 0.61 | -0.06 |
| 11 | C01 | NA | pos | NA | 81917 | 79234 | 0.34 | 0.34 | 21.59 | 24.45 |
| 11 | C02 | NA | NA | NA | 222778 | 208060 | 0.93 | 0.90 | 0.73 | 0.71 |
| 11 | C03 | 4849 | CNOT3 | NM_014516 | 211849 | 190762 | 0.88 | 0.82 | 2.85 | 3.87 |
| 11 | C04 | 9337 | CNOT8 | NM_004779 | 248537 | 222095 | 1.03 | 0.96 | -1.37 | -0.15 |
| 11 | C05 | 1316 | COPEB | NM_001300 | 216506 | 201125 | 0.90 | 0.87 | 2.24 | 2.02 |
| 11 | C06 | 1385 | CREB1 | NM_134442 | 238119 | 210719 | 0.99 | 0.91 | -0.38 | 0.31 |
| 11 | C07 | 9586 | CREB5 | NM_004904 | 252020 | 202014 | 1.05 | 0.87 | -3.09 | 0.73 |
| 11 | C08 | 1389 | CREBL2 | NM_001310 | 219941 | 209287 | 0.91 | 0.90 | 0.62 | -0.02 |
| 11 | C09 | 8804 | CREG | NM_003851 | 201238 | 202573 | 0.84 | 0.87 | 1.61 | 0.02 |
| 11 | C10 | 1390 | CREM | NM_001881 | 230165 | 226059 | 0.96 | 0.97 | -1.10 | -2.12 |
| 11 | C11 | 1406 | CRX | NM_000554 | 222166 | 200181 | 0.92 | 0.86 | -0.26 | -0.07 |
| 11 | C12 | 8531 | CSDA | NM_003651 | 222589 | 223954 | 0.93 | 0.96 | 0.26 | -0.84 |
| 11 | D01 | NA | neg | NA | 250830 | 227826 | 1.04 | 0.98 | -3.62 | -2.75 |
| 11 | D02 | NA | neg | NA | 235161 | 235008 | 0.98 | 1.01 | -1.30 | -4.08 |
| 11 | D03 | 30818 | CSEN | NM_013434 | 243694 | 202186 | 1.01 | 0.87 | -2.06 | 1.94 |
| 11 | D04 | 10664 | CTCF | NM_006565 | 221368 | 222047 | 0.92 | 0.96 | 2.46 | 0.03 |
| 11 | D05 | 1523 | CUTL1 | NM_001913 | 235122 | 211751 | 0.98 | 0.91 | -0.71 | 0.24 |
| 11 | D06 | 1628 | DBP | NM_001352 | 222992 | 215057 | 0.93 | 0.93 | 1.67 | -0.31 |
| 11 | D07 | 1649 | DDIT3 | NM_004083 | 227410 | 193752 | 0.95 | 0.83 | 0.36 | 2.42 |
| 11 | D08 | 7913 | DEK | NM_003472 | 230620 | 215703 | 0.96 | 0.93 | -1.15 | -1.02 |
| 11 | D09 | 1746 | DLX2 | NM_004405 | 208512 | 226405 | 0.87 | 0.97 | 0.34 | -4.20 |
| 11 | D10 | 1747 | DLX3 | NM_005220 | 220816 | 215657 | 0.92 | 0.93 | 0.09 | -0.03 |
| 11 | D11 | 1748 | DLX4 | NM_001934 | 232908 | 215042 | 0.97 | 0.93 | -2.04 | -2.64 |
| 11 | D12 | 1749 | DLX5 | NM_005221 | 223665 | 219394 | 0.93 | 0.94 | -0.09 | 0.18 |
| 11 | E01 | NA | neg | NA | 239392 | 243606 | 0.99 | 1.05 | -2.16 | -3.70 |
| 11 | E02 | NA | neg | NA | 239255 | 228085 | 0.99 | 0.98 | -2.14 | -0.84 |
| 11 | E03 | 1750 | DLX6 | XM_376652 | 229105 | 225787 | 0.95 | 0.97 | -0.14 | -0.44 |
| 11 | E04 | 1761 | DMRT1 | NM_021951 | 236903 | 233018 | 0.98 | 1.00 | -0.08 | -0.03 |
| 11 | E05 | 10655 | DMRT2 | NM_181872 | 222616 | 223600 | 0.93 | 0.96 | 0.90 | 0.02 |
| 11 | E06 | 58524 | DMRT3 | NM_021240 | 238673 | 220194 | 0.99 | 0.95 | -0.89 | 0.71 |
| 11 | E07 | 9988 | DMTF1 | NM_021145 | 237807 | 211086 | 0.99 | 0.91 | -1.42 | 1.19 |
| 11 | E08 | 1810 | DR1 | NM_001938 | 221219 | 221140 | 0.92 | 0.95 | 0.00 | -0.06 |
| 11 | E09 | 1820 | ARID3A | NM_005224 | 200822 | 192365 | 0.83 | 0.83 | 1.24 | 4.03 |
| 11 | E10 | 26584 | DUX1 | NM_012146 | 215233 | 226300 | 0.89 | 0.97 | 0.68 | -0.02 |
| 11 | E11 | 26583 | DUX2 | NM_012147 | 217066 | 212867 | 0.90 | 0.92 | 0.06 | -0.27 |
| 11 | E12 | 26582 | DUX3 | NM_012148 | 221428 | 225222 | 0.92 | 0.97 | 0.00 | 1.07 |
| 11 | F01 | NA | NA | NA | 246338 | 282356 | 1.02 | 1.22 | -3.55 | -12.67 |
| 11 | F02 | NA | pos | NA | 114769 | 94417 | 0.48 | 0.41 | 15.93 | 21.95 |
| 11 | F03 | 1869 | E2F1 | NM_005225 | 219253 | 213155 | 0.91 | 0.92 | 0.96 | 0.05 |
| 11 | F04 | 1877 | E4F1 | NM_004424 | 233439 | 238310 | 0.97 | 1.03 | 0.08 | -2.84 |
| 11 | F05 | 8721 | EDF1 | NM_003792 | 225574 | 210526 | 0.94 | 0.91 | 0.10 | 0.60 |
| 11 | F06 | 1958 | EGR1 | NM_001964 | 229619 | 222790 | 0.95 | 0.96 | 0.09 | -1.61 |
| 11 | F07 | 1960 | EGR3 | NM_004430 | 228176 | 226778 | 0.95 | 0.98 | -0.36 | -3.53 |
| 11 | F08 | 1961 | EGR4 | NM_001965 | 227477 | 219940 | 0.95 | 0.95 | -1.29 | -1.68 |
| 11 | F09 | 26298 | EHF | NM_012153 | 209006 | 202080 | 0.87 | 0.87 | -0.34 | 0.41 |
| 11 | F10 | 1997 | ELF1 | NM_172373 | 209741 | 211131 | 0.87 | 0.91 | 1.13 | 0.94 |
| 11 | F11 | 1998 | ELF2 | NM_006874 | 218658 | 200403 | 0.91 | 0.86 | -0.53 | 0.19 |
| 11 | F12 | 1999 | ELF3 | NM_004433 | 219482 | 221224 | 0.91 | 0.95 | -0.07 | -0.03 |
| 11 | G01 | NA | neg | NA | 241871 | 229473 | 1.01 | 0.99 | -2.79 | -2.81 |
| 11 | G02 | NA | neg | NA | 233881 | 227451 | 0.97 | 0.98 | -1.61 | -2.44 |
| 11 | G03 | 2000 | ELF4 | NM_001421 | 243829 | 219281 | 1.01 | 0.94 | -2.58 | -0.96 |
| 11 | G04 | 2001 | ELF5 | NM_001422 | 237532 | 216872 | 0.99 | 0.93 | -0.43 | 1.23 |
| 11 | G05 | 2002 | ELK1 | NM_005229 | 226548 | 217503 | 0.94 | 0.94 | 0.06 | -0.57 |
| 11 | G06 | 2004 | ELK3 | NM_005230 | 238954 | 227115 | 0.99 | 0.98 | -1.19 | -2.29 |
| 11 | G07 | 2005 | ELK4 | NM_001973 | 221975 | 224440 | 0.92 | 0.97 | 0.66 | -2.98 |
| 11 | G08 | 2016 | EMX1 | NM_004097 | 219887 | 211402 | 0.91 | 0.91 | -0.06 | 0.02 |
| 11 | G09 | 2018 | EMX2 | NM_004098 | 204846 | 205025 | 0.85 | 0.88 | 0.38 | -0.02 |
| 11 | G10 | 2019 | EN1 | NM_001426 | 215014 | 197739 | 0.89 | 0.85 | 0.46 | 3.52 |
| 11 | G11 | 2020 | EN2 | NM_001427 | 208297 | 201694 | 0.87 | 0.87 | 1.10 | 0.07 |
| 11 | G12 | 8320 | EOMES | NM_005442 | 220082 | 221517 | 0.91 | 0.95 | -0.06 | 0.03 |
| 11 | H01 | NA | NA | NA | 256779 | 241897 | 1.07 | 1.04 | -4.83 | -2.79 |
| 11 | H02 | NA | pos | NA | 101843 | 79069 | 0.42 | 0.34 | 18.12 | 27.21 |
| 11 | H03 | 2077 | ERF | NM_006494 | 237404 | 226866 | 0.99 | 0.98 | -1.46 | -0.05 |
| 11 | H04 | 2113 | ETS1 | NM_005238 | 230519 | 225880 | 0.96 | 0.97 | 0.78 | 1.88 |

| | | | | | | | | | | |
|---|---|---|---|---|---|---|---|---|---|---|
| 11 | H05 | 2114 | ETS2 | NM_005239 | 240928 | 235494 | 1.00 | 1.01 | -1.90 | -1.58 |
| 11 | H06 | 2117 | ETV3 | NM_005240 | 232025 | 232533 | 0.96 | 1.00 | 0.00 | -0.98 |
| 11 | H07 | 2118 | ETV4 | NM_001986 | 224669 | 225046 | 0.93 | 0.97 | 0.43 | -0.79 |
| 11 | H08 | 2120 | ETV6 | NM_001987 | 215548 | 220671 | 0.90 | 0.95 | 0.75 | 0.62 |
| 11 | H09 | 51513 | ETV7 | NM_016135 | 219110 | 217174 | 0.91 | 0.94 | -1.56 | 0.06 |
| 11 | H10 | 2128 | EVX1 | NM_001989 | 219863 | 231924 | 0.91 | 1.00 | -0.09 | -0.47 |
| 11 | H11 | 55810 | FOXJ2 | NM_018416 | 210184 | 211663 | 0.87 | 0.91 | 0.99 | 0.54 |
| 11 | H12 | 2307 | FKHL18 | NM_004118 | 220795 | 212706 | 0.92 | 0.92 | 0.00 | 3.96 |
| 12 | A01 | NA | pos | NA | 62751 | 76755 | 0.25 | 0.30 | 28.17 | 25.66 |
| 12 | A02 | NA | NA | NA | 296309 | 289633 | 1.17 | 1.14 | -9.80 | -4.44 |
| 12 | A03 | 2353 | FOS | NM_005252 | 259725 | 252050 | 1.02 | 1.00 | -3.00 | 1.46 |
| 12 | A04 | 8061 | FOSL1 | NM_005438 | 276483 | 273501 | 1.09 | 1.08 | -6.02 | -0.98 |
| 12 | A05 | 2355 | FOSL2 | NM_005253 | 250514 | 268744 | 0.99 | 1.06 | -1.95 | -1.00 |
| 12 | A06 | 3169 | FOXA1 | NM_004496 | 232074 | 265850 | 0.92 | 1.05 | 0.34 | -0.83 |
| 12 | A07 | 3170 | FOXA2 | NM_021784 | 244671 | 270010 | 0.96 | 1.07 | -1.13 | -1.90 |
| 12 | A08 | 3171 | FOXA3 | NM_004497 | 219228 | 243005 | 0.86 | 0.96 | 1.96 | 0.87 |
| 12 | A09 | 27023 | FOXB1 | NM_012182 | 225546 | 234447 | 0.89 | 0.93 | 0.30 | 2.13 |
| 12 | A10 | 2296 | FOXC1 | NM_001453 | 220143 | 275629 | 0.87 | 1.09 | 1.48 | -3.20 |
| 12 | A11 | 2303 | FOXC2 | NM_005251 | 227791 | 229935 | 0.90 | 0.91 | 0.20 | 3.18 |
| 12 | A12 | 2297 | FOXD1 | NM_004472 | 242761 | 262351 | 0.96 | 1.04 | -0.20 | 0.83 |
| 12 | B01 | NA | neg | NA | 281450 | 279261 | 1.11 | 1.10 | -9.83 | -8.36 |
| 12 | B02 | NA | neg | NA | 247906 | 235962 | 0.98 | 0.93 | -4.38 | -2.24 |
| 12 | B03 | 2306 | FOXD2 | NM_004474 | 231912 | 224057 | 0.91 | 0.89 | -0.92 | 0.03 |
| 12 | B04 | 27022 | FOXD3 | NM_012183 | 242755 | 238095 | 0.96 | 0.94 | -2.98 | -1.37 |
| 12 | B05 | 2298 | FOXD4 | NM_207305 | 223369 | 223558 | 0.88 | 0.88 | 0.02 | -0.01 |
| 12 | B06 | 2304 | FOXE1 | NM_004473 | 224660 | 224089 | 0.89 | 0.89 | -0.90 | -0.32 |
| 12 | B07 | 2301 | FOXE3 | NM_012186 | 222971 | 215472 | 0.88 | 0.85 | -0.05 | 0.41 |
| 12 | B08 | 2294 | FOXF1 | NM_001451 | 216348 | 210608 | 0.85 | 0.83 | -0.02 | 0.06 |
| 12 | B09 | 2295 | FOXF2 | NM_001452 | 209259 | 212562 | 0.83 | 0.84 | 0.51 | -0.17 |
| 12 | B10 | 2290 | FOXG1B | NM_005249 | 213418 | 204061 | 0.84 | 0.81 | 0.12 | 1.53 |
| 12 | B11 | 8928 | FOXH1 | NM_003923 | 211060 | 224006 | 0.83 | 0.89 | 0.48 | -1.37 |
| 12 | B12 | 2299 | FOXI1 | NM_012188 | 221162 | 230055 | 0.87 | 0.91 | 0.86 | 0.01 |
| 12 | C01 | NA | pos | NA | 82542 | 73336 | 0.33 | 0.29 | 19.36 | 20.21 |
| 12 | C02 | NA | NA | NA | 224788 | 225271 | 0.89 | 0.89 | -3.76 | -1.27 |
| 12 | C03 | 2302 | FOXJ1 | NM_001454 | 206124 | 195638 | 0.81 | 0.77 | 0.12 | 3.50 |
| 12 | C04 | 2300 | FOXL1 | NM_005250 | 202978 | 214679 | 0.80 | 0.85 | 0.34 | 1.40 |
| 12 | C05 | 668 | FOXL2 | NM_023067 | 203718 | 219791 | 0.80 | 0.87 | 0.06 | -0.02 |
| 12 | C06 | 2305 | FOXM1 | NM_021953 | 199202 | 201683 | 0.79 | 0.80 | 0.09 | 2.31 |
| 12 | C07 | 8456 | FOXN1 | NM_003593 | 202766 | 214459 | 0.80 | 0.85 | 0.09 | 0.02 |
| 12 | C08 | 2308 | FOXO1A | NM_002015 | 197294 | 187386 | 0.78 | 0.74 | -0.06 | 2.80 |
| 12 | C09 | 2309 | FOXO3A | NM_001455 | 223989 | 231045 | 0.88 | 0.91 | -5.03 | -3.33 |
| 12 | C10 | 27086 | FOXP1 | NM_032682 | 208956 | 216267 | 0.82 | 0.85 | -2.30 | -0.74 |
| 12 | C11 | 93986 | FOXP2 | NM_014491 | 195874 | 224014 | 0.77 | 0.89 | -0.20 | -1.91 |
| 12 | C12 | 50943 | FOXP3 | NM_014009 | 219824 | 233902 | 0.87 | 0.92 | -2.07 | -1.08 |
| 12 | D01 | NA | neg | NA | 277196 | 270132 | 1.09 | 1.07 | -9.66 | -7.13 |
| 12 | D02 | NA | neg | NA | 227332 | 221657 | 0.90 | 0.88 | -1.55 | -0.28 |
| 12 | D03 | 2551 | GABPA | NM_002040 | 214083 | 226992 | 0.84 | 0.90 | 1.45 | -0.45 |
| 12 | D04 | 2623 | GATA1 | NM_002049 | 222939 | 215539 | 0.88 | 0.85 | -0.28 | 1.76 |
| 12 | D05 | 2624 | GATA2 | NM_032638 | 230780 | 216077 | 0.91 | 0.85 | -1.71 | 0.99 |
| 12 | D06 | 2625 | GATA3 | NM_002051 | 246412 | 222788 | 0.97 | 0.88 | -4.96 | -0.20 |
| 12 | D07 | 2626 | GATA4 | NM_002052 | 201582 | 218074 | 0.79 | 0.86 | 2.90 | -0.02 |
| 12 | D08 | 140628 | GATA5 | NM_080473 | 217092 | 222991 | 0.86 | 0.88 | -0.66 | -1.75 |
| 12 | D09 | 2637 | GBX2 | NM_001485 | 199996 | 210827 | 0.79 | 0.83 | 1.49 | 0.01 |
| 12 | D10 | 8521 | GCM1 | NM_003643 | 217851 | 211845 | 0.86 | 0.84 | -1.12 | 0.36 |
| 12 | D11 | 2672 | GFI1 | NM_005263 | 208826 | 211862 | 0.82 | 0.84 | 0.32 | 0.29 |
| 12 | D12 | 8328 | GFI1B | NM_004188 | 220237 | 230742 | 0.87 | 0.91 | 0.49 | -0.15 |
| 12 | E01 | NA | neg | NA | 284012 | 276089 | 1.12 | 1.09 | -11.13 | -7.77 |
| 12 | E02 | NA | neg | NA | 249070 | 224382 | 0.98 | 0.89 | -5.45 | -0.46 |
| 12 | E03 | 2735 | GLI | NM_005269 | 216149 | 220059 | 0.85 | 0.87 | 0.75 | 0.74 |
| 12 | E04 | 2736 | GLI2 | NM_005270 | 209003 | 212630 | 0.82 | 0.84 | 1.62 | 2.38 |
| 12 | E05 | 2737 | GLI3 | NM_000168 | 217514 | 215501 | 0.86 | 0.85 | 0.08 | 1.27 |
| 12 | E06 | 10691 | GMEB1 | NM_006582 | 229521 | 237722 | 0.90 | 0.94 | -2.58 | -2.10 |
| 12 | E07 | 2928 | GSCL | NM_005315 | 228823 | 224825 | 0.90 | 0.89 | -1.89 | -0.77 |
| 12 | E08 | 2957 | GTF2A1 | NM_015859 | 191729 | 210836 | 0.76 | 0.83 | 3.10 | 0.17 |
| 12 | E09 | 2958 | GTF2A2 | NM_004492 | 187666 | 197555 | 0.74 | 0.78 | 3.13 | 2.09 |
| 12 | E10 | 2969 | GTF2I | NM_001518 | 214016 | 221134 | 0.84 | 0.87 | -0.86 | -0.75 |
| 12 | E11 | 9569 | GTF2IRD1 | NM_005685 | 218409 | 224235 | 0.86 | 0.89 | -1.60 | -1.26 |
| 12 | E12 | 2971 | GTF3A | NM_002097 | 221647 | 232304 | 0.87 | 0.92 | -0.10 | -0.17 |
| 12 | F01 | NA | NA | NA | 236541 | 267123 | 0.93 | 1.06 | -2.73 | -6.85 |
| 12 | F02 | NA | pos | NA | 94643 | 81804 | 0.37 | 0.32 | 20.34 | 19.35 |
| 12 | F03 | 9330 | GTF3C3 | NM_012086 | 225755 | 229057 | 0.89 | 0.91 | -0.12 | -0.88 |
| 12 | F04 | 9421 | HAND1 | NM_004821 | 221449 | 236133 | 0.87 | 0.93 | 0.28 | -1.29 |
| 12 | F05 | 29915 | HCFC2 | NM_013320 | 222342 | 221984 | 0.88 | 0.88 | -0.02 | 0.01 |
| 12 | F06 | 3054 | HCFC1 | NM_005334 | 213062 | 214470 | 0.84 | 0.85 | 0.78 | 0.84 |
| 12 | F07 | 8820 | HESX1 | NM_003865 | 229691 | 213732 | 0.91 | 0.84 | -1.34 | 0.46 |
| 12 | F08 | 23462 | HEY1 | NM_012258 | 214944 | 214169 | 0.85 | 0.85 | 0.02 | -0.65 |
| 12 | F09 | 23493 | HEY2 | NM_012259 | 216968 | 209993 | 0.86 | 0.83 | -0.94 | -0.01 |
| 12 | F10 | 26508 | HEYL | NM_014571 | 212540 | 213851 | 0.84 | 0.85 | 0.07 | -0.06 |
| 12 | F11 | 3087 | HHEX | NM_002729 | 226244 | 206634 | 0.89 | 0.82 | -2.19 | 0.88 |
| 12 | F12 | 3090 | HIC1 | NM_006497 | 222001 | 228577 | 0.88 | 0.90 | 0.53 | 0.01 |
| 12 | G01 | NA | neg | NA | 258166 | 272347 | 1.02 | 1.08 | -6.91 | -7.47 |
| 12 | G02 | NA | neg | NA | 223069 | 226257 | 0.88 | 0.89 | -1.21 | -0.95 |
| 12 | G03 | 7290 | HIRA | NM_003325 | 219306 | 225190 | 0.86 | 0.89 | 0.25 | -0.22 |
| 12 | G04 | 59269 | HIVEP3 | NM_024503 | 198948 | 220877 | 0.78 | 0.87 | 3.27 | 0.98 |
| 12 | G05 | 3104 | HKR3 | NM_005341 | 217722 | 222834 | 0.86 | 0.88 | 0.06 | 0.01 |
| 12 | G06 | 3142 | HLX1 | NM_021958 | 213187 | 212103 | 0.84 | 0.84 | 0.09 | 1.29 |
| 12 | G07 | 3110 | HLXB9 | NM_005515 | 215799 | 228120 | 0.85 | 0.90 | 0.24 | -1.46 |
| 12 | G08 | 10363 | HMG20A | NM_018200 | 216934 | 216310 | 0.86 | 0.85 | -0.98 | -0.83 |

| | | | | | | | | | | |
|---|---|---|---|---|---|---|---|---|---|---|
| 12 | G09 | 10362 | HMG20B | NM_006339 | 208890 | 208952 | 0.82 | 0.83 | -0.30 | 0.25 |
| 12 | G10 | 3198 | HOXA1 | NM_005522 | 209245 | 210790 | 0.83 | 0.83 | -0.07 | 0.49 |
| 12 | G11 | 3206 | HOXA10 | NM_018951 | 210625 | 215725 | 0.83 | 0.85 | -0.32 | -0.29 |
| 12 | G12 | 3207 | HOXA11 | NM_005523 | 226844 | 229554 | 0.89 | 0.91 | -0.93 | -0.01 |
| 12 | H01 | NA | NA | NA | 269068 | 265902 | 1.06 | 1.05 | -6.56 | -4.11 |
| 12 | H02 | NA | pos | NA | 97396 | 85089 | 0.38 | 0.34 | 21.34 | 21.45 |
| 12 | H03 | 3209 | HOXA13 | NM_000522 | 247526 | 241169 | 0.98 | 0.95 | -2.21 | -0.03 |
| 12 | H04 | 3199 | HOXA2 | NM_006735 | 241863 | 267201 | 0.95 | 1.06 | -1.59 | -3.12 |
| 12 | H05 | 3201 | HOXA4 | NM_002141 | 243323 | 244269 | 0.96 | 0.97 | -1.98 | -0.57 |
| 12 | H06 | 3202 | HOXA5 | NM_019102 | 227355 | 237138 | 0.90 | 0.94 | -0.09 | 0.20 |
| 12 | H07 | 3204 | HOXA7 | NM_006896 | 230043 | 228486 | 0.91 | 0.90 | 0.05 | 0.94 |
| 12 | H08 | 3205 | HOXA9 | NM_002142 | 218936 | 228165 | 0.86 | 0.90 | 0.82 | -0.06 |
| 12 | H09 | 3211 | HOXB1 | NM_002144 | 229204 | 229750 | 0.90 | 0.91 | -1.48 | -0.24 |
| 12 | H10 | 10481 | HOXB13 | NM_006361 | 219554 | 231121 | 0.87 | 0.91 | 0.38 | 0.06 |
| 12 | H11 | 3212 | HOXB2 | NM_002145 | 218289 | 221339 | 0.86 | 0.87 | 0.55 | 1.37 |
| 12 | H12 | 3213 | HOXB3 | NM_002146 | 233567 | 246597 | 0.92 | 0.97 | 0.10 | 0.03 |
| 13 | A01 | NA | pos | NA | 74736 | 77640 | 0.31 | 0.33 | 23.97 | 30.76 |
| 13 | A02 | NA | NA | NA | 267032 | 269913 | 1.12 | 1.14 | -4.59 | -6.25 |
| 13 | A03 | 3215 | HOXB5 | NM_002147 | 246407 | 272065 | 1.03 | 1.15 | -0.78 | -6.71 |
| 13 | A04 | 3216 | HOXB6 | NM_018952 | 230515 | 274161 | 0.97 | 1.16 | 0.62 | -5.25 |
| 13 | A05 | 3217 | HOXB7 | NM_004502 | 237054 | 235339 | 0.99 | 1.00 | 0.25 | 0.60 |
| 13 | A06 | 3226 | HOXC10 | NM_017409 | 236967 | 233600 | 0.99 | 0.99 | 0.01 | 0.79 |
| 13 | A07 | 3227 | HOXC11 | NM_014212 | 247854 | 238898 | 1.04 | 1.01 | -1.70 | 0.08 |
| 13 | A08 | 3229 | HOXC13 | NM_017410 | 214734 | 234246 | 0.90 | 0.99 | 3.13 | -0.02 |
| 13 | A09 | 3221 | HOXC4 | NM_014620 | 226628 | 227396 | 0.95 | 0.96 | -0.01 | 0.33 |
| 13 | A10 | 3222 | HOXC5 | NM_018953 | 234167 | 238057 | 0.98 | 1.01 | -0.30 | -1.76 |
| 13 | A11 | 3223 | HOXC6 | NM_004503 | 247827 | 238410 | 1.04 | 1.01 | -3.14 | -0.74 |
| 13 | A12 | 3225 | HOXC9 | NM_006897 | 232769 | 240568 | 0.98 | 1.02 | 1.48 | 0.02 |
| 13 | B01 | NA | neg | NA | 275119 | 270622 | 1.15 | 1.15 | -7.90 | -7.87 |
| 13 | B02 | NA | neg | NA | 232210 | 240012 | 0.97 | 1.02 | -1.53 | -1.98 |
| 13 | B03 | 3236 | HOXD10 | NM_002148 | 233784 | 234286 | 0.98 | 0.99 | -1.01 | -0.93 |
| 13 | B04 | 3237 | HOXD11 | NM_021192 | 215233 | 222480 | 0.90 | 0.94 | 0.78 | 3.20 |
| 13 | B05 | 3238 | HOXD12 | NM_021193 | 224526 | 230719 | 0.94 | 0.98 | 0.00 | 0.00 |
| 13 | B06 | 3239 | HOXD13 | NM_000523 | 234101 | 224533 | 0.98 | 0.95 | -1.67 | 1.04 |
| 13 | B07 | 3233 | HOXD4 | NM_014621 | 223306 | 231348 | 0.94 | 0.98 | -0.17 | 0.05 |
| 13 | B08 | 3234 | HOXD8 | NM_019558 | 228521 | 230255 | 0.96 | 0.98 | -1.02 | -0.74 |
| 13 | B09 | 3235 | HOXD9 | NM_014213 | 204959 | 221388 | 0.86 | 0.94 | 1.09 | 0.00 |
| 13 | B10 | 55806 | HR | NM_005144 | 212726 | 234891 | 0.89 | 1.00 | 0.78 | -2.64 |
| 13 | B11 | 3281 | HSBP1 | NM_001537 | 212265 | 226815 | 0.89 | 0.96 | 0.03 | 0.00 |
| 13 | B12 | 3297 | HSF1 | NM_005526 | 228558 | 222990 | 0.96 | 0.95 | 0.00 | 1.91 |
| 13 | C01 | NA | pos | NA | 66705 | 66486 | 0.28 | 0.28 | 20.56 | 28.88 |
| 13 | C02 | NA | NA | NA | 236728 | 220225 | 0.99 | 0.93 | -4.69 | -0.71 |
| 13 | C03 | 3298 | HSF2 | NM_004506 | 221214 | 206232 | 0.93 | 0.87 | -1.64 | 1.93 |
| 13 | C04 | 3299 | HSF4 | NM_001538 | 213473 | 206490 | 0.90 | 0.88 | -1.46 | 3.74 |
| 13 | C05 | 86614 | HSFY1 | NM_033108 | 202828 | 217516 | 0.85 | 0.92 | 0.73 | 0.00 |
| 13 | C06 | 11319 | HSGT1 | NM_007265 | 206150 | 214059 | 0.86 | 0.91 | -0.01 | 0.52 |
| 13 | C07 | 10553 | HTATIP2 | NM_006410 | 204881 | 223228 | 0.86 | 0.95 | 0.07 | -0.93 |
| 13 | C08 | 3394 | ICSBP1 | NM_002163 | 194858 | 208639 | 0.82 | 0.88 | 1.48 | 0.88 |
| 13 | C09 | 3399 | ID3 | NM_002167 | 195433 | 219236 | 0.82 | 0.93 | 0.01 | -2.13 |
| 13 | C10 | 3400 | ID4 | NM_001546 | 192282 | 207993 | 0.81 | 0.88 | 1.32 | 0.00 |
| 13 | C11 | 3428 | IFI16 | NM_005531 | 213649 | 219365 | 0.90 | 0.93 | -2.67 | -1.10 |
| 13 | C12 | 3607 | FOXK2 | NM_004514 | 229262 | 232496 | 0.96 | 0.99 | -2.60 | -2.46 |
| 13 | D01 | NA | neg | NA | 240232 | 244772 | 1.01 | 1.04 | -3.46 | -6.35 |
| 13 | D02 | NA | neg | NA | 236710 | 224747 | 0.99 | 0.95 | -2.94 | -2.49 |
| 13 | D03 | 3609 | ILF3 | NM_004516 | 221509 | 221651 | 0.93 | 0.94 | 0.07 | -1.95 |
| 13 | D04 | 3651 | IPF1 | NM_000209 | 214977 | 218938 | 0.90 | 0.93 | 0.07 | 0.44 |
| 13 | D05 | 3659 | IRF1 | NM_002198 | 245578 | 208823 | 1.03 | 0.89 | -3.87 | 0.76 |
| 13 | D06 | 3660 | IRF2 | NM_002199 | 214267 | 212074 | 0.90 | 0.90 | 0.53 | -0.01 |
| 13 | D07 | 3661 | IRF3 | NM_001571 | 222461 | 224723 | 0.93 | 0.95 | -0.79 | -2.13 |
| 13 | D08 | 3662 | IRF4 | NM_002460 | 203643 | 206286 | 0.85 | 0.87 | 1.93 | 0.42 |
| 13 | D09 | 3663 | IRF5 | NM_002200 | 212310 | 203414 | 0.89 | 0.86 | -0.74 | 0.00 |
| 13 | D10 | 3664 | IRF6 | NM_006147 | 212671 | 185957 | 0.89 | 0.79 | 0.04 | 3.33 |
| 13 | D11 | 3665 | IRF7 | NM_001572 | 212835 | 218123 | 0.89 | 0.93 | -0.80 | -1.78 |
| 13 | D12 | 50805 | IRX4 | NM_016358 | 223866 | 215087 | 0.94 | 0.91 | -0.05 | -0.02 |
| 13 | E01 | NA | neg | NA | 255769 | 268755 | 1.07 | 1.14 | -4.53 | -8.09 |
| 13 | E02 | NA | neg | NA | 231063 | 225738 | 0.97 | 0.96 | -0.86 | 0.19 |
| 13 | E03 | 10379 | ISGF3G | NM_006084 | 222805 | 221883 | 0.93 | 0.94 | 1.11 | 0.88 |
| 13 | E04 | 64843 | ISL2 | NM_145805 | 251835 | 238400 | 1.06 | 1.01 | -4.16 | -0.44 |
| 13 | E05 | 3725 | JUN | NM_002228 | 232286 | 233346 | 0.97 | 0.99 | -0.65 | -1.08 |
| 13 | E06 | 3726 | JUNB | NM_002229 | 226796 | 242046 | 0.95 | 1.03 | -0.09 | -2.90 |
| 13 | E07 | 3727 | JUND | NM_005354 | 221603 | 219649 | 0.93 | 0.93 | 0.58 | 1.72 |
| 13 | E08 | 10661 | KLF1 | NM_006563 | 235501 | 223373 | 0.99 | 0.95 | -1.56 | 0.01 |
| 13 | E09 | 11278 | KLF12 | NM_007249 | 208052 | 207003 | 0.87 | 0.88 | 1.13 | 2.19 |
| 13 | E10 | 51621 | KLF13 | NM_015995 | 222770 | 218233 | 0.93 | 0.93 | -0.22 | -0.01 |
| 13 | E11 | 10365 | KLF2 | NM_016270 | 211277 | 196834 | 0.89 | 0.83 | 0.67 | 5.20 |
| 13 | E12 | 51274 | KLF3 | NM_016531 | 231289 | 238361 | 0.97 | 1.01 | 0.09 | -1.62 |
| 13 | F01 | NA | NA | NA | 244730 | 238643 | 1.03 | 1.01 | -3.59 | -3.54 |
| 13 | F02 | NA | pos | NA | 82551 | 87907 | 0.35 | 0.37 | 20.50 | 25.47 |
| 13 | F03 | 9314 | KLF4 | NM_004235 | 207006 | 219917 | 0.87 | 0.93 | 2.76 | 0.01 |
| 13 | F04 | 688 | KLF5 | NM_001730 | 233416 | 232327 | 0.98 | 0.99 | -2.12 | -0.52 |
| 13 | F05 | 8609 | KLF7 | NM_003709 | 223197 | 225750 | 0.94 | 0.96 | 0.00 | -0.87 |
| 13 | F06 | 10660 | LBX1 | NM_006562 | 230923 | 238409 | 0.97 | 1.01 | -1.40 | -3.45 |
| 13 | F07 | 8861 | LDB1 | NM_003893 | 220967 | 219759 | 0.93 | 0.93 | -0.02 | 0.45 |
| 13 | F08 | 9079 | LDB2 | NM_001290 | 213941 | 212070 | 0.90 | 0.90 | 0.94 | 0.94 |
| 13 | F09 | 8022 | LHX3 | NM_014564 | 203566 | 212366 | 0.85 | 0.90 | 1.10 | -0.09 |
| 13 | F10 | 89884 | LHX4 | NM_033343 | 221037 | 211687 | 0.93 | 0.90 | -0.66 | 0.00 |
| 13 | F11 | 64211 | LHX5 | NM_022363 | 205023 | 217337 | 0.86 | 0.92 | 0.90 | 0.00 |
| 13 | F12 | 26468 | LHX6 | NM_014368 | 227184 | 220993 | 0.95 | 0.94 | 0.00 | 0.47 |

| | | | | | | | | | | |
|---|---|---|---|---|---|---|---|---|---|---|
| 13 | G01 | NA | neg | NA | 247173 | 225275 | 1.04 | 0.96 | -3.99 | -0.69 |
| 13 | G02 | NA | neg | NA | 225517 | 231580 | 0.95 | 0.98 | -0.77 | -1.90 |
| 13 | G03 | 4009 | LMX1A | NM_177398 | 225802 | 221524 | 0.95 | 0.94 | -0.07 | -0.01 |
| 13 | G04 | 4010 | LMX1B | NM_002316 | 214887 | 219698 | 0.90 | 0.93 | 0.59 | 2.20 |
| 13 | G05 | 11178 | LZTS1 | NM_021020 | 213418 | 215584 | 0.89 | 0.91 | 1.41 | 1.37 |
| 13 | G06 | 4084 | MAD | NM_002357 | 209063 | 221905 | 0.88 | 0.94 | 1.81 | 0.01 |
| 13 | G07 | 4088 | SMAD3 | NM_005902 | 220433 | 223831 | 0.92 | 0.95 | 0.02 | -0.05 |
| 13 | G08 | 4089 | SMAD4 | NM_005359 | 232116 | 218447 | 0.97 | 0.93 | -1.80 | -0.01 |
| 13 | G09 | 4094 | MAF | NM_005360 | 215268 | 214582 | 0.90 | 0.91 | -0.68 | -0.24 |
| 13 | G10 | 4097 | MAFG | NM_002359 | 201358 | 210728 | 0.84 | 0.89 | 2.23 | 0.47 |
| 13 | G11 | 7975 | MAFK | NM_002360 | 211028 | 211789 | 0.88 | 0.90 | -0.03 | 1.35 |
| 13 | G12 | 4152 | MBD1 | NM_002384 | 230463 | 226815 | 0.97 | 0.96 | -0.52 | -0.37 |
| 13 | H01 | NA | NA | NA | 239769 | 250142 | 1.01 | 1.06 | -1.83 | -3.66 |
| 13 | H02 | NA | pos | NA | 81480 | 77749 | 0.34 | 0.33 | 21.68 | 29.53 |
| 13 | H03 | 8932 | MBD2 | NM_003927 | 231563 | 219962 | 0.97 | 0.93 | 0.14 | 2.10 |
| 13 | H04 | 4197 | MDS1 | NM_004991 | 226453 | 242825 | 0.95 | 1.03 | -0.07 | -0.44 |
| 13 | H05 | 4204 | MECP2 | NM_004992 | 231077 | 234058 | 0.97 | 0.99 | -0.15 | -0.37 |
| 13 | H06 | 4205 | MEF2A | NM_005587 | 224109 | 239189 | 0.94 | 1.01 | 0.63 | -1.50 |
| 13 | H07 | 4207 | MEF2B | NM_005919 | 227496 | 234831 | 0.95 | 1.00 | 0.03 | -0.35 |
| 13 | H08 | 4208 | MEF2C | NM_002397 | 233478 | 230649 | 0.98 | 0.98 | -0.94 | -0.54 |
| 13 | H09 | 4209 | MEF2D | NM_005920 | 226888 | 220633 | 0.95 | 0.94 | -1.34 | 0.41 |
| 13 | H10 | 4212 | MEIS2 | NM_020149 | 223764 | 220777 | 0.94 | 0.94 | -0.04 | 0.35 |
| 13 | H11 | 4222 | MEOX1 | NM_004527 | 205631 | 221607 | 0.86 | 0.94 | 1.83 | 1.28 |
| 13 | H12 | 4223 | MEOX2 | NM_005924 | 226922 | 225745 | 0.95 | 0.96 | 1.06 | 1.65 |
| 14 | A01 | NA | pos | NA | 79242 | 69489 | 0.34 | 0.29 | 15.96 | 27.80 |
| 14 | A02 | NA | NA | NA | 256198 | 236706 | 1.09 | 0.99 | -2.29 | 0.88 |
| 14 | A03 | 4286 | MITF | NM_000248 | 227196 | 239089 | 0.97 | 1.00 | 1.55 | 0.60 |
| 14 | A04 | 83881 | MIXL1 | NM_031944 | 247072 | 253410 | 1.05 | 1.06 | -1.28 | -1.38 |
| 14 | A05 | 4297 | MLL | NM_005933 | 238502 | 239869 | 1.02 | 1.00 | 0.02 | 1.13 |
| 14 | A06 | 9757 | MLL4 | NM_014727 | 225382 | 246555 | 0.96 | 1.03 | -0.01 | 0.14 |
| 14 | A07 | 8028 | MLLT10 | NM_004641 | 226435 | 243225 | 0.97 | 1.01 | 0.22 | -0.62 |
| 14 | A08 | 4303 | MLLT7 | NM_005938 | 234502 | 240620 | 1.00 | 1.00 | -0.40 | -0.94 |
| 14 | A09 | 4335 | MNT | NM_020310 | 234028 | 236576 | 1.00 | 0.99 | -0.06 | -0.11 |
| 14 | A10 | 10934 | MORF4 | NM_006792 | 246570 | 243163 | 1.05 | 1.01 | -1.07 | -0.22 |
| 14 | A11 | 9242 | MSC | NM_005098 | 206542 | 225478 | 0.88 | 0.94 | 1.90 | 1.50 |
| 14 | A12 | 4487 | MSX1 | NM_002448 | 235986 | 234441 | 1.01 | 0.98 | 0.00 | 2.08 |
| 14 | B01 | NA | neg | NA | 270216 | 279804 | 1.15 | 1.17 | -4.91 | -7.61 |
| 14 | B02 | NA | neg | NA | 239524 | 243393 | 1.02 | 1.01 | -1.75 | -1.75 |
| 14 | B03 | 4488 | MSX2 | NM_002449 | 231232 | 232492 | 0.99 | 0.97 | -0.05 | 0.12 |
| 14 | B04 | 9219 | MTA2 | NM_004739 | 217953 | 225755 | 0.93 | 0.94 | 0.54 | 1.53 |
| 14 | B05 | 7978 | MTERF | NM_006980 | 239156 | 246612 | 1.02 | 1.03 | -1.23 | -1.50 |
| 14 | B06 | 4520 | MTF1 | NM_005955 | 219169 | 238329 | 0.93 | 0.99 | -0.55 | -0.08 |
| 14 | B07 | 10608 | MXD4 | NM_006454 | 203107 | 203856 | 0.87 | 0.85 | 1.44 | 4.18 |
| 14 | B08 | 4601 | MXI1 | NM_005962 | 227939 | 221956 | 0.97 | 0.93 | -0.90 | 0.52 |
| 14 | B09 | 4602 | MYB | NM_005375 | 211149 | 230545 | 0.90 | 0.96 | 1.12 | -0.68 |
| 14 | B10 | 4603 | MYBL1 | XM_034274 | 217878 | 235245 | 0.93 | 0.98 | 0.70 | -0.49 |
| 14 | B11 | 4605 | MYBL2 | NM_002466 | 222795 | 239252 | 0.95 | 1.00 | -0.96 | -2.26 |
| 14 | B12 | 4609 | MYC | NM_002467 | 223992 | 227688 | 0.96 | 0.95 | 0.06 | 1.62 |
| 14 | C01 | NA | pos | NA | 68833 | 70596 | 0.29 | 0.29 | 15.15 | 24.38 |
| 14 | C02 | NA | NA | NA | 231444 | 242965 | 0.99 | 1.01 | -1.62 | -3.38 |
| 14 | C03 | 4610 | MYCL1 | NM_005376 | 227367 | 228634 | 0.97 | 0.95 | -0.36 | -0.97 |
| 14 | C04 | 4611 | MYCL2 | NM_005377 | 203842 | 207594 | 0.87 | 0.87 | 1.29 | 2.75 |
| 14 | C05 | 4613 | MYCN | NM_005378 | 210254 | 212934 | 0.90 | 0.89 | 1.04 | 2.22 |
| 14 | C06 | 4617 | MYF5 | NM_005593 | 213468 | 209748 | 0.91 | 0.87 | -0.67 | 2.82 |
| 14 | C07 | 4618 | MYF6 | NM_002469 | 206573 | 227398 | 0.88 | 0.95 | 0.37 | -1.32 |
| 14 | C08 | 4654 | MYOD1 | NM_002478 | 207602 | 215749 | 0.89 | 0.90 | 0.48 | -0.19 |
| 14 | C09 | 4656 | MYOG | NM_002479 | 234013 | 215108 | 1.00 | 0.90 | -1.95 | 0.10 |
| 14 | C10 | 4661 | MYT1 | NM_004535 | 202613 | 216898 | 0.86 | 0.90 | 1.57 | 0.76 |
| 14 | C11 | 23040 | MYT1L | XM_039762 | 220965 | 227044 | 0.94 | 0.95 | -1.48 | -2.00 |
| 14 | C12 | 4665 | NAB2 | NM_005967 | 236932 | 240942 | 1.01 | 1.00 | -1.98 | -2.21 |
| 14 | D01 | NA | neg | NA | 226261 | 236899 | 0.96 | 0.99 | -0.83 | -1.28 |
| 14 | D02 | NA | neg | NA | 227856 | 234074 | 0.97 | 0.98 | -0.99 | -0.83 |
| 14 | D03 | 79923 | NANOG | NM_024865 | 244771 | 229275 | 1.04 | 0.96 | -1.89 | 0.05 |
| 14 | D04 | 8031 | NCOA4 | NM_005437 | 217453 | 203238 | 0.93 | 0.85 | 0.15 | 4.57 |
| 14 | D05 | 9611 | NCOR1 | NM_006311 | 223056 | 239514 | 0.95 | 1.00 | -0.02 | -0.94 |
| 14 | D06 | 9612 | NCOR2 | NM_006312 | 201704 | 239864 | 0.86 | 1.00 | 0.81 | -0.91 |
| 14 | D07 | 4760 | NEUROD1 | NM_002500 | 212710 | 227719 | 0.91 | 0.95 | 0.01 | -0.25 |
| 14 | D08 | 10725 | NFAT5 | NM_006599 | 210811 | 220323 | 0.90 | 0.92 | 0.42 | 0.20 |
| 14 | D09 | 4772 | NFATC1 | NM_006162 | 226760 | 197076 | 0.97 | 0.82 | -0.94 | 4.12 |
| 14 | D10 | 4773 | NFATC2 | NM_012340 | 223788 | 220133 | 0.95 | 0.92 | -0.35 | 1.36 |
| 14 | D11 | 4775 | NFATC3 | NM_004555 | 208792 | 222004 | 0.89 | 0.93 | 0.04 | -0.07 |
| 14 | D12 | 4776 | NFATC4 | NM_004554 | 220308 | 236221 | 0.94 | 0.98 | 0.00 | -0.34 |
| 14 | E01 | NA | neg | NA | 260352 | 260196 | 1.11 | 1.08 | -3.62 | -4.85 |
| 14 | E02 | NA | neg | NA | 229489 | 234406 | 0.98 | 0.98 | -0.44 | -0.70 |
| 14 | E03 | 4778 | NFE2 | NM_006163 | 220066 | 231020 | 0.94 | 0.96 | 1.37 | -0.05 |
| 14 | E04 | 4779 | NFE2L1 | NM_003204 | 227328 | 235477 | 0.97 | 0.98 | -0.15 | -0.44 |
| 14 | E05 | 4780 | NFE2L2 | NM_006164 | 198878 | 223726 | 0.85 | 0.93 | 3.20 | 1.78 |
| 14 | E06 | 9603 | NFE2L3 | NM_004289 | 216464 | 234795 | 0.92 | 0.98 | 0.01 | 0.08 |
| 14 | E07 | 4774 | NFIA | NM_005595 | 219814 | 222394 | 0.94 | 0.93 | -0.01 | 0.79 |
| 14 | E08 | 4781 | NFIB | NM_005596 | 230210 | 240544 | 0.98 | 1.00 | -0.87 | -2.88 |
| 14 | E09 | 4782 | NFIC | NM_005597 | 213038 | 220932 | 0.91 | 0.92 | 1.20 | 0.46 |
| 14 | E10 | 4783 | NFIL3 | NM_005384 | 211832 | 231455 | 0.90 | 0.96 | 1.60 | -0.28 |
| 14 | E11 | 4784 | NFIX | NM_002501 | 216580 | 209372 | 0.92 | 0.87 | -0.04 | 2.14 |
| 14 | E12 | 4790 | NFKB1 | NM_003998 | 237019 | 238012 | 1.01 | 0.99 | -1.01 | -0.44 |
| 14 | F01 | NA | NA | NA | 244021 | 250011 | 1.04 | 1.04 | -3.34 | -3.86 |
| 14 | F02 | NA | pos | NA | 93483 | 92437 | 0.40 | 0.39 | 12.19 | 21.51 |
| 14 | F03 | 4791 | NFKB2 | NM_002502 | 220194 | 225674 | 0.94 | 0.94 | -0.04 | 0.16 |
| 14 | F04 | 4796 | NFKBIL2 | NM_013432 | 227416 | 230533 | 0.97 | 0.96 | -1.56 | -0.29 |

| | | | | | | | | | | |
|---|---|---|---|---|---|---|---|---|---|---|
| 14 | F05 | 4800 | NFYA | NM_002505 | 215049 | 226997 | 0.92 | 0.95 | 0.13 | 0.61 |
| 14 | F06 | 4801 | NFYB | NM_006166 | 202506 | 237323 | 0.86 | 0.99 | 0.05 | -0.97 |
| 14 | F07 | 4802 | NFYC | NM_014223 | 213070 | 233860 | 0.91 | 0.98 | -0.71 | -1.71 |
| 14 | F08 | 4821 | NKX2-2 | NM_002509 | 194370 | 208325 | 0.83 | 0.87 | 1.43 | 1.66 |
| 14 | F09 | 159296 | NKX2-3 | NM_145285 | 210482 | 220405 | 0.90 | 0.92 | 0.06 | -0.10 |
| 14 | F10 | 1482 | NKX2-5 | NM_004387 | 216289 | 226913 | 0.92 | 0.95 | -0.26 | -0.20 |
| 14 | F11 | 26257 | NKX2-8 | NM_014360 | 193608 | 218229 | 0.83 | 0.91 | 0.93 | 0.07 |
| 14 | F12 | 4824 | NKX3-1 | NM_006167 | 229563 | 229168 | 0.98 | 0.96 | -1.64 | 0.34 |
| 14 | G01 | NA | neg | NA | 251546 | 242811 | 1.07 | 1.01 | -3.70 | -3.66 |
| 14 | G02 | NA | neg | NA | 224315 | 236532 | 0.96 | 0.99 | -0.90 | -2.65 |
| 14 | G03 | 4825 | NKX6-1 | NM_006168 | 222980 | 233294 | 0.95 | 0.97 | 0.08 | -2.02 |
| 14 | G04 | 84504 | NKX6-2 | NM_177400 | 204118 | 221032 | 0.87 | 0.92 | 1.25 | 0.29 |
| 14 | G05 | 9111 | NMI | NM_004688 | 224184 | 237000 | 0.96 | 0.99 | -0.40 | -1.96 |
| 14 | G06 | 4899 | NRF1 | NM_005011 | 221311 | 225999 | 0.94 | 0.94 | -1.48 | -0.10 |
| 14 | G07 | 8204 | NRIP1 | NM_003489 | 223531 | 215548 | 0.95 | 0.90 | -1.38 | 0.29 |
| 14 | G08 | 4901 | NRL | NM_006177 | 212807 | 222513 | 0.91 | 0.93 | -0.06 | -1.58 |
| 14 | G09 | 4904 | NSEP1 | NM_004559 | 200317 | 201154 | 0.85 | 0.84 | 1.52 | 2.05 |
| 14 | G10 | 3175 | ONECUT1 | NM_004498 | 221915 | 218503 | 0.95 | 0.91 | -0.43 | 0.20 |
| 14 | G11 | 9480 | ONECUT2 | NM_004852 | 197503 | 206281 | 0.84 | 0.86 | 0.94 | 1.04 |
| 14 | G12 | 5013 | OTX1 | NM_014562 | 209513 | 227441 | 0.89 | 0.95 | 0.84 | -0.34 |
| 14 | H01 | NA | NA | NA | 254427 | 251018 | 1.08 | 1.05 | -3.47 | -3.62 |
| 14 | H02 | NA | pos | NA | 79603 | 79164 | 0.34 | 0.33 | 14.56 | 24.06 |
| 14 | H03 | 57459 | P66BETA | NM_020699 | 228573 | 233602 | 0.97 | 0.97 | 0.04 | -0.71 |
| 14 | H04 | 5074 | PAWR | NM_002583 | 233126 | 240356 | 0.99 | 1.00 | -1.21 | -1.46 |
| 14 | H05 | 5077 | PAX3 | NM_000438 | 234496 | 237079 | 1.00 | 0.99 | -0.94 | -0.61 |
| 14 | H06 | 5078 | PAX4 | NM_006193 | 211870 | 226567 | 0.90 | 0.94 | 0.02 | 1.17 |
| 14 | H07 | 5081 | PAX7 | NM_002584 | 215507 | 224223 | 0.92 | 0.93 | -0.02 | 0.25 |
| 14 | H08 | 57326 | PBXIP1 | NM_020524 | 216764 | 220030 | 0.92 | 0.92 | 0.06 | 0.19 |
| 14 | H09 | 10923 | PC4 | NM_006713 | 229806 | 226667 | 0.98 | 0.95 | -0.99 | -0.70 |
| 14 | H10 | 5201 | PFDN1 | NM_002622 | 220396 | 223084 | 0.94 | 0.93 | 0.26 | 0.82 |
| 14 | H11 | 5204 | PFDN5 | NM_002624 | 214855 | 222855 | 0.92 | 0.93 | -0.32 | -0.27 |
| 14 | H12 | 5252 | PHF1 | NM_002636 | 213948 | 217219 | 0.91 | 0.91 | 0.91 | 2.67 |
| 15 | A01 | NA | pos | NA | 87103 | 72491 | 0.33 | 0.31 | 20.37 | 28.47 |
| 15 | A02 | NA | NA | NA | 266880 | 293317 | 1.02 | 1.25 | -2.43 | -6.59 |
| 15 | A03 | 401 | PHOX2A | NM_005169 | 291293 | 289622 | 1.12 | 1.23 | -5.62 | -4.88 |
| 15 | A04 | 8929 | PHOX2B | NM_003924 | 251193 | 292035 | 0.96 | 1.24 | 0.61 | -5.48 |
| 15 | A05 | 10745 | PHTF1 | NM_006608 | 250186 | 262570 | 0.96 | 1.12 | -0.17 | -1.64 |
| 15 | A06 | 10401 | PIAS3 | NM_006099 | 250620 | 241983 | 0.96 | 1.03 | 0.31 | 2.59 |
| 15 | A07 | 51588 | PIAS4 | NM_015897 | 245762 | 252854 | 0.94 | 1.08 | -0.47 | -0.23 |
| 15 | A08 | 8544 | PIR | NM_003662 | 241257 | 263960 | 0.93 | 1.12 | 0.42 | -2.42 |
| 15 | A09 | 5307 | PITX1 | NM_002653 | 239349 | 241663 | 0.92 | 1.03 | -0.28 | 0.23 |
| 15 | A10 | 5308 | PITX2 | NM_000325 | 226493 | 238048 | 0.87 | 1.01 | 2.86 | 0.72 |
| 15 | A11 | 5309 | PITX3 | NM_005029 | 232839 | 238281 | 0.89 | 1.01 | 0.13 | 1.67 |
| 15 | A12 | 5316 | PKNOX1 | NM_004571 | 249507 | 233125 | 0.96 | 0.99 | -0.12 | 3.38 |
| 15 | B01 | NA | neg | NA | 301276 | 282290 | 1.16 | 1.20 | -9.64 | -8.61 |
| 15 | B02 | NA | neg | NA | 253417 | 231440 | 0.97 | 0.99 | -3.57 | -0.54 |
| 15 | B03 | 5324 | PLAG1 | NM_002655 | 208623 | 230439 | 0.80 | 0.98 | 2.01 | 0.75 |
| 15 | B04 | 5326 | PLAGL2 | NM_002657 | 246115 | 233550 | 0.94 | 0.99 | -1.59 | 0.03 |
| 15 | B05 | 10765 | JARID1B | NM_006618 | 218236 | 226249 | 0.84 | 0.96 | 1.03 | 0.36 |
| 15 | B06 | 5449 | POU1F1 | NM_000306 | 233064 | 226644 | 0.89 | 0.96 | -0.32 | 1.25 |
| 15 | B07 | 5450 | POU2AF1 | NM_006235 | 230704 | 232270 | 0.89 | 0.99 | -1.41 | -0.74 |
| 15 | B08 | 5451 | POU2F1 | NM_002697 | 220716 | 225969 | 0.85 | 0.96 | 0.17 | -0.16 |
| 15 | B09 | 5452 | POU2F2 | NM_002698 | 215962 | 223174 | 0.83 | 0.95 | -0.16 | -0.61 |
| 15 | B10 | 25833 | POU2F3 | NM_014352 | 231339 | 224647 | 0.89 | 0.96 | -0.61 | -0.92 |
| 15 | B11 | 5453 | POU3F1 | NM_002699 | 205267 | 217072 | 0.79 | 0.92 | 0.77 | 1.27 |
| 15 | B12 | 5454 | POU3F2 | NM_005604 | 219811 | 230933 | 0.84 | 0.98 | 0.79 | -0.04 |
| 15 | C01 | NA | pos | NA | 79455 | 72904 | 0.30 | 0.31 | 18.73 | 24.95 |
| 15 | C02 | NA | NA | NA | 236032 | 230605 | 0.91 | 0.98 | -1.12 | -0.09 |
| 15 | C03 | 5455 | POU3F3 | NM_006236 | 204631 | 240620 | 0.79 | 1.02 | 2.76 | -0.55 |
| 15 | C04 | 5456 | POU3F4 | NM_000307 | 180064 | 207917 | 0.69 | 0.89 | 7.03 | 4.42 |
| 15 | C05 | 5457 | POU4F1 | NM_006237 | 217013 | 226318 | 0.83 | 0.96 | 1.42 | 0.66 |
| 15 | C06 | 5459 | POU4F3 | NM_002700 | 231253 | 233615 | 0.89 | 0.99 | 0.15 | 0.46 |
| 15 | C07 | 10891 | PPARGC1A | NM_013261 | 222564 | 236512 | 0.85 | 1.01 | -0.13 | -1.09 |
| 15 | C08 | 639 | PRDM1 | NM_001198 | 236829 | 213804 | 0.91 | 0.91 | -1.63 | 2.09 |
| 15 | C09 | 63976 | PRDM16 | NM_022114 | 215274 | 222756 | 0.83 | 0.95 | 0.16 | -0.23 |
| 15 | C10 | 7799 | PRDM2 | NM_012231 | 233960 | 223863 | 0.90 | 0.95 | -0.70 | -0.48 |
| 15 | C11 | 11108 | PRDM4 | NM_012406 | 228473 | 225823 | 0.88 | 0.96 | -1.93 | 0.20 |
| 15 | C12 | 5626 | PROP1 | NM_006261 | 229785 | 234963 | 0.88 | 1.00 | -0.23 | -0.36 |
| 15 | D01 | NA | neg | NA | 247744 | 244909 | 0.95 | 1.04 | -3.98 | -3.92 |
| 15 | D02 | NA | neg | NA | 236709 | 230958 | 0.91 | 0.98 | -2.58 | -1.71 |
| 15 | D03 | 5396 | PRRX1 | NM_022716 | 231411 | 225497 | 0.89 | 0.96 | -2.01 | 0.28 |
| 15 | D04 | 51450 | PRRX2 | NM_016307 | 222950 | 222927 | 0.86 | 0.95 | 0.22 | 0.47 |
| 15 | D05 | 5813 | PURA | NM_005859 | 220308 | 225508 | 0.85 | 0.96 | -0.36 | -0.77 |
| 15 | D06 | 5925 | RB1 | NM_000321 | 221019 | 226835 | 0.85 | 0.97 | 0.08 | -0.03 |
| 15 | D07 | 57786 | RBAK | NM_021163 | 187335 | 217195 | 0.72 | 0.92 | 2.96 | 0.41 |
| 15 | D08 | 5926 | ARID4A | NM_023001 | 213653 | 220462 | 0.82 | 0.94 | -0.06 | -0.54 |
| 15 | D09 | 5927 | JARID1A | NM_005056 | 224347 | 202593 | 0.86 | 0.86 | -2.35 | 1.41 |
| 15 | D10 | 5971 | RELB | NM_006509 | 188291 | 216639 | 0.72 | 0.92 | 3.72 | -0.90 |
| 15 | D11 | 473 | RERE | NM_012102 | 208205 | 220333 | 0.80 | 0.94 | -0.73 | -0.50 |
| 15 | D12 | 5978 | REST | NM_005612 | 216205 | 222554 | 0.83 | 0.95 | 0.12 | 0.04 |
| 15 | E01 | NA | neg | NA | 308894 | 249780 | 1.19 | 1.06 | -10.53 | -4.50 |
| 15 | E02 | NA | neg | NA | 240605 | 228842 | 0.92 | 0.97 | -1.88 | -1.18 |
| 15 | E03 | 5989 | RFX1 | NM_002918 | 211679 | 230082 | 0.81 | 0.98 | 1.69 | -0.25 |
| 15 | E04 | 5990 | RFX2 | NM_000635 | 235297 | 239933 | 0.90 | 1.02 | -0.15 | -2.04 |
| 15 | E05 | 5991 | RFX3 | NM_002919 | 225538 | 219691 | 0.87 | 0.94 | 0.17 | 0.34 |
| 15 | E06 | 5993 | RFX5 | NM_000449 | 211599 | 229203 | 0.81 | 0.98 | 2.48 | -0.21 |
| 15 | E07 | 8625 | RFXANK | NM_003721 | 206165 | 219533 | 0.79 | 0.93 | 1.78 | 0.23 |
| 15 | E08 | 5994 | RFXAP | NM_000538 | 230307 | 211462 | 0.88 | 0.90 | -0.97 | 1.08 |

| | | | | | | | | | | |
|---|---|---|---|---|---|---|---|---|---|---|
| 15 | E09 | 6018 | RLF | NM_012421 | 212312 | 196946 | 0.81 | 0.84 | 0.37 | 2.50 |
| 15 | E10 | 6239 | RREB1 | NM_002955 | 233420 | 205782 | 0.90 | 0.88 | -0.80 | 1.01 |
| 15 | E11 | 861 | RUNX1 | NM_001754 | 224638 | 227173 | 0.86 | 0.97 | -1.61 | -1.39 |
| 15 | E12 | 860 | RUNX2 | NM_004348 | 236474 | 235551 | 0.91 | 1.00 | -1.25 | -1.83 |
| 15 | F01 | NA | NA | NA | 287793 | 260382 | 1.10 | 1.11 | -8.15 | -6.29 |
| 15 | F02 | NA | pos | NA | 94723 | 97722 | 0.36 | 0.42 | 16.33 | 19.53 |
| 15 | F03 | 10284 | SAP18 | NM_005870 | 234589 | 225072 | 0.90 | 0.96 | -1.51 | 0.44 |
| 15 | F04 | 8819 | SAP30 | NM_003864 | 233682 | 237686 | 0.90 | 1.01 | -0.24 | -1.78 |
| 15 | F05 | 6322 | SCML1 | NM_006746 | 217200 | 218401 | 0.83 | 0.93 | 0.93 | 0.44 |
| 15 | F06 | 6473 | SHOX | NM_000451 | 229437 | 227060 | 0.88 | 0.97 | -0.08 | 0.03 |
| 15 | F07 | 6474 | SHOX2 | NM_003030 | 216533 | 225802 | 0.83 | 0.96 | 0.17 | -0.87 |
| 15 | F08 | 6495 | SIX1 | NM_005982 | 229060 | 222632 | 0.88 | 0.95 | -1.11 | -0.79 |
| 15 | F09 | 10736 | SIX2 | NM_016932 | 200925 | 205326 | 0.77 | 0.87 | 1.52 | 1.06 |
| 15 | F10 | 6496 | SIX3 | NM_005413 | 232253 | 211625 | 0.89 | 0.90 | -0.95 | -0.02 |
| 15 | F11 | 51804 | SIX4 | NM_017420 | 208997 | 229588 | 0.80 | 0.98 | 0.08 | -1.88 |
| 15 | F12 | 4990 | SIX6 | NM_007374 | 202793 | 222140 | 0.78 | 0.95 | 2.73 | 0.19 |
| 15 | G01 | NA | neg | NA | 267626 | 238326 | 1.03 | 1.01 | -6.22 | -2.52 |
| 15 | G02 | NA | neg | NA | 273477 | 230779 | 1.05 | 0.98 | -6.96 | -1.32 |
| 15 | G03 | 30061 | SLC40A1 | NM_014585 | 234477 | 227940 | 0.90 | 0.97 | -2.12 | 0.25 |
| 15 | G04 | 6603 | SMARCD2 | NM_003077 | 227294 | 217172 | 0.87 | 0.92 | -0.05 | 1.74 |
| 15 | G05 | 6604 | SMARCD3 | NM_003078 | 227447 | 225063 | 0.87 | 0.96 | -0.99 | -0.34 |
| 15 | G06 | 6605 | SMARCE1 | NM_003079 | 226261 | 231082 | 0.87 | 0.98 | -0.30 | -0.34 |
| 15 | G07 | 8289 | ARID1A | NM_018450 | 211866 | 215626 | 0.81 | 0.92 | 0.13 | 1.02 |
| 15 | G08 | 6618 | SNAPC2 | NM_003083 | 214941 | 218321 | 0.83 | 0.93 | 0.06 | 0.16 |
| 15 | G09 | 55509 | SNFT | NM_018664 | 206493 | 220371 | 0.79 | 0.94 | 0.19 | -1.05 |
| 15 | G10 | 22938 | SKIIP | NM_012245 | 192821 | 190394 | 0.74 | 0.81 | 3.43 | 3.62 |
| 15 | G11 | 6663 | SOX10 | NM_006941 | 202530 | 220692 | 0.78 | 0.94 | 0.27 | -0.20 |
| 15 | G12 | 6665 | SOX15 | NM_006942 | 223613 | 242285 | 0.86 | 1.03 | -0.54 | -2.73 |
| 15 | H01 | NA | NA | NA | 270045 | 251635 | 1.04 | 1.07 | -5.71 | -3.01 |
| 15 | H02 | NA | pos | NA | 90052 | 92434 | 0.35 | 0.39 | 17.12 | 22.27 |
| 15 | H03 | 54345 | SOX18 | NM_018419 | 212393 | 280874 | 0.82 | 1.20 | 1.51 | -6.52 |
| 15 | H04 | 6657 | SOX2 | NM_003106 | 232940 | 238601 | 0.89 | 1.02 | 0.05 | -0.03 |
| 15 | H05 | 6659 | SOX4 | NM_003107 | 233027 | 239718 | 0.89 | 1.02 | -0.87 | -1.04 |
| 15 | H06 | 30812 | SOX8 | NM_014587 | 236879 | 240319 | 0.91 | 1.02 | -0.83 | -0.18 |
| 15 | H07 | 6662 | SOX9 | NM_000346 | 226006 | 223278 | 0.87 | 0.95 | -0.84 | 1.43 |
| 15 | H08 | 6667 | SP1 | NM_138473 | 205368 | 223465 | 0.79 | 0.95 | 2.09 | 0.97 |
| 15 | H09 | 11262 | SP140 | NM_007237 | 221830 | 229983 | 0.85 | 0.98 | -0.94 | -0.96 |
| 15 | H10 | 6670 | SP3 | NM_003111 | 221534 | 223358 | 0.85 | 0.95 | 0.61 | 0.02 |
| 15 | H11 | 6671 | SP4 | NM_003112 | 211764 | 214271 | 0.81 | 0.91 | -0.08 | 2.45 |
| 15 | H12 | 25803 | SPDEF | NM_012391 | 201818 | 225033 | 0.77 | 0.96 | 3.05 | 1.63 |
| 16 | A01 | NA | pos | NA | 83126 | 75226 | 0.36 | 0.29 | 21.30 | 30.34 |
| 16 | A02 | NA | NA | NA | 287422 | 276169 | 1.23 | 1.07 | -5.62 | -4.62 |
| 16 | A03 | 6688 | SPI1 | NM_003120 | 284929 | 249387 | 1.22 | 0.97 | -5.52 | 0.78 |
| 16 | A04 | 6689 | SPIB | NM_003121 | 294732 | 276715 | 1.26 | 1.08 | -5.60 | -3.50 |
| 16 | A05 | 6720 | SREBF1 | NM_004176 | 297084 | 267334 | 1.27 | 1.04 | -5.55 | -1.92 |
| 16 | A06 | 6722 | SRF | NM_003131 | 225673 | 250190 | 0.97 | 0.97 | 2.56 | 0.00 |
| 16 | A07 | 6736 | SRY | NM_003140 | 248538 | 292096 | 1.07 | 1.14 | -0.58 | -8.66 |
| 16 | A08 | 6756 | SSX1 | NM_005635 | 229773 | 237689 | 0.98 | 0.92 | 1.94 | 2.19 |
| 16 | A09 | 51684 | SUFU | NM_016169 | 246324 | 249000 | 1.06 | 0.97 | -0.26 | 0.00 |
| 16 | A10 | 6827 | SUPT4H1 | NM_003168 | 244099 | 253261 | 1.05 | 0.98 | 0.66 | -1.90 |
| 16 | A11 | 6862 | T | NM_003181 | 235765 | 243151 | 1.01 | 0.95 | 0.26 | 0.11 |
| 16 | A12 | 6871 | TADA2L | NM_001488 | 234481 | 235478 | 1.01 | 0.92 | 1.66 | 2.27 |
| 16 | B01 | NA | neg | NA | 265830 | 295293 | 1.14 | 1.15 | -4.79 | -10.43 |
| 16 | B02 | NA | neg | NA | 234671 | 263204 | 1.01 | 1.02 | -0.69 | -4.85 |
| 16 | B03 | 10474 | TADA3L | NM_006354 | 219117 | 241295 | 0.94 | 0.94 | 1.14 | -0.29 |
| 16 | B04 | 6881 | TAF10 | NM_006284 | 220799 | 239957 | 0.95 | 0.93 | 2.12 | 0.41 |
| 16 | B05 | 6882 | TAF11 | NM_005643 | 239325 | 243331 | 1.03 | 0.95 | 0.04 | -0.22 |
| 16 | B06 | 6883 | TAF12 | NM_005644 | 214338 | 224586 | 0.92 | 0.87 | 2.04 | 1.98 |
| 16 | B07 | 9014 | TAF1B | NM_005680 | 229075 | 226759 | 0.98 | 0.88 | -0.04 | 0.23 |
| 16 | B08 | 6875 | TAF4B | XM_290809 | 237429 | 246660 | 1.02 | 0.96 | -1.08 | -1.85 |
| 16 | B09 | 6877 | TAF5 | NM_006951 | 231129 | 240637 | 0.99 | 0.94 | -0.28 | -1.03 |
| 16 | B10 | 27097 | TAF5L | NM_014409 | 234111 | 232274 | 1.00 | 0.90 | -0.05 | -0.73 |
| 16 | B11 | 6878 | TAF6 | NM_005641 | 215637 | 220298 | 0.92 | 0.86 | 0.90 | 1.61 |
| 16 | B12 | 10629 | TAF6L | NM_006473 | 232351 | 228988 | 1.00 | 0.89 | -0.08 | 0.92 |
| 16 | C01 | NA | pos | NA | 71292 | 83708 | 0.31 | 0.33 | 18.52 | 23.96 |
| 16 | C02 | NA | NA | NA | 224947 | 235007 | 0.96 | 0.91 | -1.72 | -2.37 |
| 16 | C03 | 6879 | TAF7 | NM_005642 | 192958 | 225740 | 0.83 | 0.88 | 2.26 | -0.01 |
| 16 | C04 | 54457 | TAF7L | NM_024885 | 201890 | 221605 | 0.87 | 0.86 | 2.30 | 1.18 |
| 16 | C05 | 6880 | TAF9 | NM_003187 | 222354 | 225708 | 0.95 | 0.88 | -0.04 | 0.42 |
| 16 | C06 | 23435 | TARDBP | NM_007375 | 210386 | 216802 | 0.90 | 0.84 | 0.24 | 0.91 |
| 16 | C07 | 6908 | TBP | NM_003194 | 219473 | 223130 | 0.94 | 0.87 | -1.09 | -1.57 |
| 16 | C08 | 9519 | TBPL1 | NM_004865 | 210963 | 226003 | 0.90 | 0.88 | 0.09 | -0.68 |
| 16 | C09 | 10716 | TBR1 | NM_006593 | 217639 | 218073 | 0.93 | 0.85 | -0.82 | 0.47 |
| 16 | C10 | 9095 | TBX19 | NM_005149 | 215791 | 214085 | 0.92 | 0.83 | 0.05 | 0.01 |
| 16 | C11 | 6909 | TBX2 | NM_005994 | 231739 | 232065 | 0.99 | 0.90 | -3.54 | -2.87 |
| 16 | C12 | 30009 | TBX21 | NM_013351 | 227553 | 237928 | 0.98 | 0.93 | -1.77 | -3.06 |
| 16 | D01 | NA | neg | NA | 231926 | 263798 | 0.99 | 1.03 | -0.32 | -6.94 |
| 16 | D02 | NA | neg | NA | 236132 | 232031 | 1.01 | 0.90 | -0.87 | -1.41 |
| 16 | D03 | 50945 | TBX22 | NM_016954 | 229117 | 234377 | 0.98 | 0.91 | -0.18 | -1.08 |
| 16 | D04 | 6926 | TBX3 | NM_005996 | 236380 | 232689 | 1.01 | 0.90 | 0.08 | -0.31 |
| 16 | D05 | 9496 | TBX4 | NM_018488 | 239855 | 232139 | 1.03 | 0.90 | -0.02 | -0.26 |
| 16 | D06 | 6910 | TBX5 | NM_000192 | 209626 | 225320 | 0.90 | 0.88 | 2.66 | -0.14 |
| 16 | D07 | 6911 | TBX6 | NM_004608 | 204884 | 205952 | 0.88 | 0.80 | 3.15 | 1.86 |
| 16 | D08 | 6917 | TCEA1 | NM_006756 | 229255 | 223841 | 0.98 | 0.87 | 0.00 | 0.14 |
| 16 | D09 | 6919 | TCEA2 | NM_003195 | 229004 | 201163 | 0.98 | 0.78 | 0.00 | 3.85 |
| 16 | D10 | 6920 | TCEA3 | NM_003196 | 240707 | 214221 | 1.03 | 0.83 | -0.91 | 0.43 |
| 16 | D11 | 6924 | TCEB3 | NM_003198 | 205245 | 205758 | 0.88 | 0.80 | 2.27 | 2.15 |
| 16 | D12 | 10915 | TCERG1 | NM_006706 | 232755 | 225131 | 1.00 | 0.88 | -0.13 | -0.40 |

| | | | | | | | | | | |
|---|---|---|---|---|---|---|---|---|---|---|
| 16 | E01 | NA | neg | NA | 224967 | 289086 | 0.96 | 1.12 | -0.70 | -10.35 |
| 16 | E02 | NA | neg | NA | 229050 | 239998 | 0.98 | 0.93 | -1.24 | -1.81 |
| 16 | E03 | 6927 | TCF1 | NM_000545 | 217901 | 233790 | 0.93 | 0.91 | 0.00 | 0.01 |
| 16 | E04 | 6938 | TCF12 | NM_003205 | 240966 | 236661 | 1.03 | 0.92 | -1.83 | -0.01 |
| 16 | E05 | 6939 | TCF15 | NM_004609 | 229632 | 236654 | 0.98 | 0.92 | 0.02 | -0.06 |
| 16 | E06 | 6928 | TCF2 | NM_000458 | 230574 | 231434 | 0.99 | 0.90 | -1.40 | -0.21 |
| 16 | E07 | 6942 | TCF20 | NM_005650 | 232233 | 208540 | 1.00 | 0.81 | -1.75 | 2.40 |
| 16 | E08 | 6943 | TCF21 | NM_003206 | 219333 | 221581 | 0.94 | 0.86 | 0.00 | 1.52 |
| 16 | E09 | 6929 | TCF3 | NM_003200 | 211904 | 214949 | 0.91 | 0.84 | 0.95 | 2.44 |
| 16 | E10 | 6925 | TCF4 | NM_003199 | 213251 | 215792 | 0.91 | 0.84 | 1.40 | 1.14 |
| 16 | E11 | 6932 | TCF7 | NM_003202 | 214588 | 227953 | 0.92 | 0.89 | -0.26 | -0.72 |
| 16 | E12 | 83439 | TCF7L1 | NM_031283 | 220926 | 233389 | 0.95 | 0.91 | 0.13 | -0.84 |
| 16 | F01 | NA | NA | NA | 249556 | 261037 | 1.07 | 1.02 | -3.86 | -6.17 |
| 16 | F02 | NA | pos | NA | 77710 | 94121 | 0.33 | 0.37 | 18.78 | 22.87 |
| 16 | F03 | 6935 | TCF8 | NM_030751 | 219141 | 225549 | 0.94 | 0.88 | -0.08 | 0.74 |
| 16 | F04 | 6944 | TCFL1 | NM_005997 | 228261 | 232483 | 0.98 | 0.90 | -0.08 | 0.01 |
| 16 | F05 | 6945 | TCFL4 | NM_170607 | 214108 | 222305 | 0.92 | 0.86 | 2.15 | 1.73 |
| 16 | F06 | 7003 | TEAD1 | NM_021961 | 222368 | 230476 | 0.95 | 0.90 | -0.24 | -0.75 |
| 16 | F07 | 7005 | TEAD3 | NM_003214 | 210056 | 221578 | 0.90 | 0.86 | 1.25 | -0.57 |
| 16 | F08 | 7019 | TFAM | NM_003201 | 220936 | 231805 | 0.95 | 0.90 | -0.13 | -0.96 |
| 16 | F09 | 7020 | TFAP2A | NM_003220 | 208624 | 225857 | 0.89 | 0.88 | 1.47 | -0.16 |
| 16 | F10 | 7021 | TFAP2B | NM_003221 | 228769 | 218400 | 0.98 | 0.85 | -0.56 | -0.01 |
| 16 | F11 | 83741 | TFAP2BL1 | NM_172238 | 206772 | 207619 | 0.89 | 0.81 | 0.85 | 2.11 |
| 16 | F12 | 7022 | TFAP2C | NM_003222 | 221909 | 222215 | 0.95 | 0.86 | 0.08 | 0.40 |
| 16 | G01 | NA | neg | NA | 242817 | 251035 | 1.04 | 0.98 | -3.53 | -4.07 |
| 16 | G02 | NA | neg | NA | 230279 | 225039 | 0.99 | 0.88 | -1.88 | 0.45 |
| 16 | G03 | 7023 | TFAP4 | NM_003223 | 214266 | 232008 | 0.92 | 0.90 | 0.00 | -0.02 |
| 16 | G04 | 7024 | TFCP2 | NM_005653 | 214587 | 230021 | 0.92 | 0.89 | 1.17 | 0.80 |
| 16 | G05 | 7027 | TFDP1 | NM_007111 | 200316 | 219655 | 0.86 | 0.85 | 3.41 | 2.56 |
| 16 | G06 | 7029 | TFDP2 | NM_006286 | 224736 | 225911 | 0.96 | 0.88 | -1.11 | 0.41 |
| 16 | G07 | 7030 | TFE3 | NM_006521 | 215013 | 219207 | 0.92 | 0.85 | 0.04 | 0.20 |
| 16 | G08 | 7942 | TFEB | NM_007162 | 225699 | 229144 | 0.97 | 0.89 | -1.31 | -0.14 |
| 16 | G09 | 22797 | TFEC | NM_012252 | 215556 | 233127 | 0.92 | 0.91 | 0.00 | -1.06 |
| 16 | G10 | 7050 | TGIF | NM_003244 | 215072 | 220295 | 0.92 | 0.86 | 0.69 | 0.02 |
| 16 | G11 | 60436 | TGIF2 | NM_021809 | 214879 | 222457 | 0.92 | 0.87 | -0.78 | -0.11 |
| 16 | G12 | 90316 | TGIF2LX | NM_138960 | 226903 | 232668 | 0.97 | 0.90 | -1.13 | -1.06 |
| 16 | H01 | NA | NA | NA | 255025 | 255955 | 1.09 | 1.00 | -4.18 | -3.03 |
| 16 | H02 | NA | pos | NA | 87795 | 91450 | 0.38 | 0.36 | 17.85 | 25.60 |
| 16 | H03 | 90655 | TGIF2LY | NM_139214 | 218554 | 233655 | 0.94 | 0.91 | 0.40 | 1.59 |
| 16 | H04 | 7073 | TIAL1 | NM_003252 | 246223 | 274532 | 1.06 | 1.07 | -2.04 | -5.04 |
| 16 | H05 | 7071 | TIEG | NM_005655 | 233919 | 244914 | 1.00 | 0.95 | -0.06 | 0.06 |
| 16 | H06 | 8462 | TIEG2 | NM_003597 | 233293 | 239210 | 1.00 | 0.93 | -1.27 | 0.00 |
| 16 | H07 | 7080 | TITF1 | NM_003317 | 220672 | 232446 | 0.95 | 0.90 | 0.25 | -0.20 |
| 16 | H08 | 3195 | TLX1 | NM_005521 | 222602 | 237489 | 0.95 | 0.92 | 0.06 | 0.31 |
| 16 | H09 | 3196 | TLX2 | NM_001534 | 220876 | 237924 | 0.95 | 0.93 | 0.26 | 0.00 |
| 16 | H10 | 7110 | TMF1 | NM_007114 | 233431 | 234181 | 1.00 | 0.91 | -0.77 | -0.50 |
| 16 | H11 | 7158 | TP53BP1 | NM_005657 | 222342 | 234703 | 0.95 | 0.91 | -0.80 | -0.34 |
| 16 | H12 | 7161 | TP73 | NM_005427 | 215188 | 228350 | 0.92 | 0.89 | 1.37 | 1.59 |
| 17 | A01 | NA | pos | NA | 74482 | 81643 | 0.31 | 0.34 | 20.39 | 26.43 |
| 17 | A02 | NA | NA | NA | 254851 | 267472 | 1.04 | 1.11 | -0.72 | -5.83 |
| 17 | A03 | 8626 | TP73L | NM_003722 | 296073 | 234226 | 1.21 | 0.97 | -4.94 | 0.26 |
| 17 | A04 | 55809 | TRERF1 | NM_033502 | 246459 | 242516 | 1.01 | 1.01 | 0.89 | -0.78 |
| 17 | A05 | 9321 | TRIP11 | NM_004239 | 257154 | 235454 | 1.05 | 0.98 | -0.63 | 0.07 |
| 17 | A06 | 7227 | TRPS1 | NM_014112 | 294847 | 277180 | 1.21 | 1.15 | -4.70 | -7.89 |
| 17 | A07 | 8848 | TGFB1I4 | NM_006022 | 273919 | 230421 | 1.12 | 0.96 | -3.15 | -0.04 |
| 17 | A08 | 7270 | TTF1 | NM_007344 | 223166 | 228122 | 0.91 | 0.95 | 3.18 | 0.89 |
| 17 | A09 | 117581 | TWIST2 | NM_057179 | 251764 | 238952 | 1.03 | 0.99 | -1.31 | -2.17 |
| 17 | A10 | 27033 | TZFP | NM_014383 | 232337 | 235416 | 0.95 | 0.98 | 1.07 | -0.23 |
| 17 | A11 | 7343 | UBTF | NM_014233 | 223353 | 219928 | 0.91 | 0.92 | 0.63 | 1.20 |
| 17 | A12 | 7391 | USF1 | NM_007122 | 230823 | 234344 | 0.95 | 0.98 | 1.53 | 1.10 |
| 17 | B01 | NA | neg | NA | 293749 | 296129 | 1.20 | 1.23 | -7.39 | -11.30 |
| 17 | B02 | NA | neg | NA | 249339 | 240637 | 1.02 | 1.00 | -2.19 | -1.66 |
| 17 | B03 | 7392 | USF2 | NM_003367 | 230309 | 233014 | 0.94 | 0.97 | 0.64 | -0.02 |
| 17 | B04 | 8433 | UTF1 | NM_003577 | 242965 | 232381 | 1.00 | 0.97 | -0.81 | 0.49 |
| 17 | B05 | 25806 | VAX2 | NM_012476 | 231931 | 233432 | 0.95 | 0.97 | 0.21 | -0.07 |
| 17 | B06 | 27287 | VENTX2 | NM_014468 | 237896 | 238103 | 0.97 | 0.99 | -0.14 | -1.60 |
| 17 | B07 | 30813 | VSX1 | NM_014588 | 244093 | 227172 | 1.00 | 0.95 | -1.78 | 0.04 |
| 17 | B08 | 7490 | WT1 | NM_000378 | 245451 | 234668 | 1.01 | 0.98 | -1.54 | -0.74 |
| 17 | B09 | 7528 | YY1 | NM_003403 | 218950 | 223079 | 0.90 | 0.93 | 0.41 | 0.10 |
| 17 | B10 | 677 | ZFP36L1 | NM_004926 | 252808 | 241074 | 1.04 | 1.00 | -3.44 | -1.70 |
| 17 | B11 | 7539 | ZFP37 | NM_003408 | 195407 | 220727 | 0.80 | 0.92 | 1.79 | 0.57 |
| 17 | B12 | 51043 | ZFP67 | NM_015872 | 224634 | 227640 | 0.92 | 0.95 | 0.14 | 1.78 |
| 17 | C01 | NA | pos | NA | 77545 | 71119 | 0.32 | 0.30 | 16.48 | 26.37 |
| 17 | C02 | NA | NA | NA | 239825 | 232838 | 0.98 | 0.97 | -2.51 | -1.70 |
| 17 | C03 | 23660 | ZFP95 | NM_014569 | 185997 | 224723 | 0.76 | 0.94 | 4.40 | 0.02 |
| 17 | C04 | 7543 | ZFX | NM_003410 | 218715 | 203017 | 0.90 | 0.85 | 0.59 | 4.19 |
| 17 | C05 | 7544 | ZFY | NM_003411 | 221435 | 227646 | 0.91 | 0.95 | 0.00 | -0.46 |
| 17 | C06 | 11244 | ZHX1 | NM_007222 | 227957 | 225329 | 0.93 | 0.94 | -0.41 | -0.77 |
| 17 | C07 | 22882 | ZHX2 | NM_014943 | 211923 | 212524 | 0.87 | 0.88 | 0.56 | 1.19 |
| 17 | C08 | 7545 | ZIC1 | NM_003412 | 210547 | 212647 | 0.86 | 0.89 | 1.11 | 1.69 |
| 17 | C09 | 7695 | ZNF136 | NM_003437 | 210215 | 204761 | 0.86 | 0.85 | 0.00 | 1.88 |
| 17 | C10 | 7702 | ZNF143 | NM_003442 | 226743 | 223579 | 0.93 | 0.93 | -1.82 | -0.06 |
| 17 | C11 | 7704 | ZBTB16 | NM_006006 | 209888 | 220142 | 0.86 | 0.92 | -1.34 | -0.72 |
| 17 | C12 | 7707 | ZNF148 | NM_021964 | 214831 | 235068 | 0.88 | 0.98 | -0.14 | -0.90 |
| 17 | D01 | NA | neg | NA | 248589 | 250072 | 1.02 | 1.04 | -2.98 | -5.07 |
| 17 | D02 | NA | neg | NA | 237847 | 239841 | 0.97 | 1.00 | -1.72 | -3.29 |
| 17 | D03 | 7716 | ZNF161 | NM_007146 | 234322 | 224647 | 0.96 | 0.94 | -0.70 | -0.33 |
| 17 | D04 | 7727 | ZNF174 | NM_003450 | 221546 | 223183 | 0.91 | 0.93 | 0.83 | 0.32 |

| | | | | | | | | | | |
|---|---|---|---|---|---|---|---|---|---|---|
| 17 | D05 | 10168 | ZNF197 | NM_006991 | 215041 | 219156 | 0.88 | 0.91 | 1.31 | 0.64 |
| 17 | D06 | 7572 | ZNF24 | NM_006965 | 226285 | 215831 | 0.93 | 0.90 | 0.34 | 0.51 |
| 17 | D07 | 23528 | ZNF281 | NM_012482 | 221492 | 234001 | 0.91 | 0.97 | 0.00 | -2.91 |
| 17 | D08 | 7581 | ZNF33A | NM_006974 | 224848 | 201743 | 0.92 | 0.84 | 0.00 | 3.21 |
| 17 | D09 | 7584 | ZNF35 | NM_003420 | 196364 | 216454 | 0.80 | 0.90 | 2.18 | -0.52 |
| 17 | D10 | 6940 | ZNF354A | NM_005649 | 216349 | 213807 | 0.89 | 0.89 | -0.04 | 1.27 |
| 17 | D11 | 57541 | ZNF398 | NM_020781 | 216654 | 228217 | 0.89 | 0.95 | -1.57 | -2.49 |
| 17 | D12 | 7593 | ZNF42 | NM_003422 | 229243 | 233004 | 0.94 | 0.97 | -1.27 | -0.92 |
| 17 | E01 | NA | neg | NA | 291861 | 238245 | 1.20 | 0.99 | -7.71 | -1.77 |
| 17 | E02 | NA | neg | NA | 234694 | 227318 | 0.96 | 0.95 | -1.02 | 0.13 |
| 17 | E03 | 7596 | ZNF45 | NM_003425 | 236712 | 231882 | 0.97 | 0.97 | -0.64 | -0.35 |
| 17 | E04 | 55769 | ZNF83 | NM_018300 | 223281 | 238905 | 0.91 | 0.99 | 0.95 | -1.17 |
| 17 | E05 | 7639 | ZNF85 | NM_003429 | 232775 | 229256 | 0.95 | 0.95 | -0.43 | 0.13 |
| 17 | E06 | 7555 | ZNF9 | NM_003418 | 225875 | 223455 | 0.93 | 0.93 | 0.72 | 0.43 |
| 17 | E07 | 22806 | ZNFN1A3 | NM_012481 | 228043 | 206138 | 0.93 | 0.86 | -0.44 | 3.17 |
| 17 | E08 | 326 | AIRE | NM_000383 | 227679 | 228699 | 0.93 | 0.95 | 0.00 | -0.23 |
| 17 | E09 | 10488 | CREB3 | NM_006368 | 217851 | 209517 | 0.89 | 0.87 | 0.00 | 1.93 |
| 17 | E10 | 27336 | HTATSF1 | NM_014500 | 224561 | 230376 | 0.92 | 0.96 | -0.67 | -0.37 |
| 17 | E11 | 54700 | RRN3 | NM_018427 | 183269 | 205955 | 0.75 | 0.86 | 2.67 | 2.62 |
| 17 | E12 | 9338 | TCEAL1 | NM_004780 | 212732 | 235836 | 0.87 | 0.98 | 1.00 | -0.17 |
| 17 | F01 | NA | NA | NA | 254635 | 234386 | 1.04 | 0.98 | -4.83 | -1.66 |
| 17 | F02 | NA | pos | NA | 75567 | 100305 | 0.31 | 0.42 | 16.13 | 21.61 |
| 17 | F03 | 55290 | BRF2 | NM_018310 | 209102 | 218106 | 0.86 | 0.91 | 1.11 | 1.48 |
| 17 | F04 | 10658 | CUGBP1 | NM_006560 | 223832 | 222074 | 0.92 | 0.92 | -0.59 | 1.19 |
| 17 | F05 | 8562 | DENR | NM_003677 | 216417 | 232340 | 0.89 | 0.97 | 0.00 | -0.96 |
| 17 | F06 | 1917 | EEF1A2 | NM_001958 | 226436 | 221315 | 0.93 | 0.92 | -0.82 | 0.23 |
| 17 | F07 | 1933 | EEF1B2 | NM_001959 | 211397 | 228761 | 0.87 | 0.95 | 0.03 | -1.32 |
| 17 | F08 | 9086 | EIF1AY | NM_004681 | 224795 | 228579 | 0.92 | 0.95 | -1.14 | -0.77 |
| 17 | F09 | 8890 | EIF2B4 | NM_015636 | 206379 | 217967 | 0.85 | 0.91 | -0.14 | -0.10 |
| 17 | F10 | 8893 | EIF2B5 | NM_003907 | 205813 | 224690 | 0.84 | 0.94 | 0.04 | 0.06 |
| 17 | F11 | 8669 | EIF3S1 | NM_003758 | 193414 | 221326 | 0.79 | 0.92 | 0.00 | -0.61 |
| 17 | F12 | 8668 | EIF3S2 | NM_003757 | 184424 | 197169 | 0.76 | 0.82 | 2.83 | 5.98 |
| 17 | G01 | NA | neg | NA | 239688 | 266367 | 0.98 | 1.11 | -2.86 | -8.13 |
| 17 | G02 | NA | neg | NA | 232003 | 226503 | 0.95 | 0.94 | -1.96 | -1.21 |
| 17 | G03 | 8667 | EIF3S3 | NM_003756 | 226237 | 223112 | 0.93 | 0.93 | -0.67 | -0.31 |
| 17 | G04 | 8666 | EIF3S4 | NM_003755 | 228121 | 225486 | 0.93 | 0.94 | -0.87 | -0.32 |
| 17 | G05 | 8665 | EIF3S5 | NM_003754 | 207295 | 220965 | 0.85 | 0.92 | 1.30 | 0.09 |
| 17 | G06 | 3646 | EIF3S6 | NM_001568 | 197603 | 201161 | 0.81 | 0.84 | 2.78 | 2.81 |
| 17 | G07 | 8664 | EIF3S7 | NM_003753 | 191703 | 206495 | 0.79 | 0.86 | 2.56 | 1.62 |
| 17 | G08 | 8663 | EIF3S8 | NM_003752 | 217946 | 219582 | 0.89 | 0.91 | -0.11 | -0.13 |
| 17 | G09 | 8662 | EIF3S9 | NM_003751 | 167657 | 177283 | 0.69 | 0.74 | 4.62 | 6.05 |
| 17 | G10 | 1975 | EIF4B | NM_001417 | 207134 | 218114 | 0.85 | 0.91 | 0.11 | 0.28 |
| 17 | G11 | 1977 | EIF4E | NM_001968 | 201044 | 215802 | 0.82 | 0.90 | -0.66 | -0.57 |
| 17 | G12 | 3396 | ICT1 | NM_001545 | 228021 | 233237 | 0.93 | 0.97 | -2.05 | -1.20 |
| 17 | H01 | NA | NA | NA | 236086 | 250389 | 0.97 | 1.04 | -0.55 | -3.67 |
| 17 | H02 | NA | pos | NA | 77086 | 82353 | 0.32 | 0.34 | 18.06 | 25.50 |
| 17 | H03 | 54516 | MTRF1L | NM_019041 | 225002 | 225173 | 0.92 | 0.94 | 1.36 | 1.02 |
| 17 | H04 | 7458 | WBSCR1 | NM_022170 | 249205 | 236778 | 1.02 | 0.99 | -1.45 | -0.59 |
| 17 | H05 | 53947 | A4GALT | NM_017436 | 245052 | 232133 | 1.00 | 0.97 | -1.24 | -0.16 |
| 17 | H06 | 10157 | AASS | NM_005763 | 236244 | 228443 | 0.97 | 0.95 | 0.14 | -0.23 |
| 17 | H07 | 18 | ABAT | NM_000663 | 229723 | 236277 | 0.94 | 0.98 | 0.00 | -1.86 |
| 17 | H08 | 11057 | ABHD2 | NM_007011 | 232590 | 227859 | 0.95 | 0.95 | 0.06 | 0.13 |
| 17 | H09 | 171586 | ABHD3 | NM_138340 | 229660 | 226199 | 0.94 | 0.94 | -0.75 | -0.76 |
| 17 | H10 | 63874 | ABHD4 | NM_022060 | 217885 | 222988 | 0.89 | 0.93 | 0.74 | 1.12 |
| 17 | H11 | 51099 | ABHD5 | NM_016006 | 211489 | 215335 | 0.87 | 0.90 | 0.00 | 1.19 |
| 17 | H12 | 57406 | ABHD6 | NM_020676 | 232114 | 235118 | 0.95 | 0.98 | -0.64 | 0.17 |
| 18 | A01 | NA | pos | NA | 78989 | 90585 | 0.31 | 0.38 | 21.65 | 20.94 |
| 18 | A02 | NA | NA | NA | 287892 | 290424 | 1.14 | 1.20 | -5.40 | -5.51 |
| 18 | A03 | 79575 | ABHD8 | NM_024527 | 241237 | 283265 | 0.95 | 1.17 | 0.46 | -3.95 |
| 18 | A04 | 79852 | ABHD9 | NM_024794 | 268770 | 275137 | 1.06 | 1.14 | -2.06 | -2.52 |
| 18 | A05 | 30 | ACAA1 | NM_001607 | 295543 | 291227 | 1.17 | 1.21 | -4.93 | -3.74 |
| 18 | A06 | 33 | ACADL | NM_001608 | 231557 | 245795 | 0.92 | 1.02 | 2.67 | 0.81 |
| 18 | A07 | 34 | ACADM | NM_000016 | 299081 | 246798 | 1.18 | 1.02 | -6.71 | -0.20 |
| 18 | A08 | 35 | ACADS | NM_000017 | 241578 | 236535 | 0.96 | 0.98 | 0.15 | 1.55 |
| 18 | A09 | 36 | ACADSB | NM_001609 | 241587 | 239235 | 0.96 | 0.99 | 0.08 | 0.62 |
| 18 | A10 | 37 | ACADVL | NM_000018 | 240779 | 240201 | 0.95 | 1.00 | -0.14 | 0.08 |
| 18 | A11 | 38 | ACAT1 | NM_000019 | 238490 | 240526 | 0.94 | 1.00 | -0.31 | -0.07 |
| 18 | A12 | 59272 | ACE2 | NM_021804 | 224758 | 234634 | 0.89 | 0.97 | 4.39 | 1.94 |
| 18 | B01 | NA | neg | NA | 283795 | 287316 | 1.12 | 1.19 | -7.58 | -6.77 |
| 18 | B02 | NA | neg | NA | 243185 | 232091 | 0.96 | 0.96 | -2.32 | 0.54 |
| 18 | B03 | 51 | ACOX1 | NM_004035 | 218416 | 229656 | 0.86 | 0.95 | 0.71 | 1.48 |
| 18 | B04 | 53 | ACP2 | NM_001610 | 248456 | 247269 | 0.98 | 1.02 | -2.14 | -0.50 |
| 18 | B05 | 51205 | ACP6 | NM_016361 | 234395 | 250437 | 0.93 | 1.04 | 0.28 | -0.01 |
| 18 | B06 | 93650 | ACPT | NM_033068 | 231443 | 240924 | 0.92 | 1.00 | -0.02 | -0.21 |
| 18 | B07 | 49 | ACR | NM_001097 | 226115 | 232598 | 0.90 | 0.96 | 0.03 | 0.01 |
| 18 | B08 | 95 | ACY1 | NM_000666 | 233017 | 238280 | 0.92 | 0.99 | -1.46 | -0.35 |
| 18 | B09 | 100 | ADA | NM_000022 | 227062 | 239133 | 0.90 | 0.99 | -0.75 | -1.03 |
| 18 | B10 | 8749 | ADAM18 | NM_014237 | 224073 | 227550 | 0.89 | 0.94 | -0.68 | 0.08 |
| 18 | B11 | 11086 | ADAM29 | NM_014269 | 212696 | 222356 | 0.84 | 0.92 | 0.31 | 0.66 |
| 18 | B12 | 203102 | ADAM32 | NM_145004 | 220660 | 228885 | 0.87 | 0.95 | 2.21 | 1.03 |
| 18 | C01 | NA | pos | NA | 72947 | 76486 | 0.29 | 0.32 | 18.02 | 19.60 |
| 18 | C02 | NA | NA | NA | 230008 | 234454 | 0.91 | 0.97 | -2.32 | -1.31 |
| 18 | C03 | 140766 | ADAMTS14 | NM_080722 | 215034 | 229681 | 0.85 | 0.95 | -0.56 | -0.06 |
| 18 | C04 | 9509 | ADAMTS2 | NM_014244 | 202336 | 200894 | 0.80 | 0.83 | 2.12 | 4.10 |
| 18 | C05 | 113622 | ADPRHL1 | NM_138430 | 211824 | 226244 | 0.84 | 0.94 | 1.50 | 1.65 |
| 18 | C06 | 54936 | ADPRHL2 | NM_017825 | 214640 | 217226 | 0.85 | 0.90 | 0.45 | 1.39 |
| 18 | C07 | 143 | ADPRTL1 | NM_006437 | 214695 | 244529 | 0.85 | 1.01 | -0.20 | -3.11 |
| 18 | C08 | 10039 | ADPRTL3 | NM_005485 | 207341 | 219271 | 0.82 | 0.91 | 0.16 | 0.63 |

| | | | | | | | | | | |
|---|---|---|---|---|---|---|---|---|---|---|
| 18 | C09 | 158 | ADSL | NM_000026 | 229143 | 225562 | 0.91 | 0.93 | -2.73 | -0.77 |
| 18 | C10 | 246181 | AFAR3 | NM_201252 | 191741 | 218923 | 0.76 | 0.91 | 1.80 | -0.31 |
| 18 | C11 | 175 | AGA | NM_000027 | 220626 | 215227 | 0.87 | 0.89 | -2.42 | 0.07 |
| 18 | C12 | 10554 | AGPAT1 | NM_006411 | 229922 | 233376 | 0.91 | 0.97 | -0.69 | -1.10 |
| 18 | D01 | NA | neg | NA | 262088 | 266804 | 1.04 | 1.11 | -5.34 | -6.70 |
| 18 | D02 | NA | neg | NA | 240291 | 231237 | 0.95 | 0.96 | -2.52 | -1.99 |
| 18 | D03 | 10555 | AGPAT2 | NM_006412 | 223034 | 220937 | 0.88 | 0.92 | -0.46 | -0.02 |
| 18 | D04 | 8540 | AGPS | NM_003659 | 229588 | 223296 | 0.91 | 0.93 | -0.27 | 0.03 |
| 18 | D05 | 189 | AGXT | NM_000030 | 227737 | 236599 | 0.90 | 0.98 | 0.57 | -0.83 |
| 18 | D06 | 191 | AHCY | NM_000687 | 231602 | 217700 | 0.92 | 0.90 | -0.61 | 0.21 |
| 18 | D07 | 10768 | AHCYL1 | NM_006621 | 217654 | 197854 | 0.86 | 0.82 | 0.55 | 1.96 |
| 18 | D08 | 9590 | AKAP12 | NM_005100 | 214616 | 227395 | 0.85 | 0.94 | 0.35 | -1.55 |
| 18 | D09 | 57016 | AKR1B10 | NM_020299 | 214507 | 201751 | 0.85 | 0.84 | 0.30 | 1.27 |
| 18 | D10 | 8644 | AKR1C3 | NM_003739 | 212632 | 199996 | 0.84 | 0.83 | 0.22 | 1.08 |
| 18 | D11 | 1109 | AKR1C4 | NM_001818 | 227616 | 216081 | 0.90 | 0.90 | -2.19 | -1.15 |
| 18 | D12 | 6718 | AKR1D1 | NM_005989 | 237033 | 231464 | 0.94 | 0.96 | -0.48 | -1.95 |
| 18 | E01 | NA | neg | NA | 294886 | 256798 | 1.17 | 1.06 | -10.56 | -3.57 |
| 18 | E02 | NA | neg | NA | 242752 | 243182 | 0.96 | 1.01 | -3.82 | -1.77 |
| 18 | E03 | 8574 | AKR7A2 | NM_003689 | 225920 | 233963 | 0.89 | 0.97 | -1.82 | 0.06 |
| 18 | E04 | 22977 | AKR7A3 | NM_012067 | 191668 | 237504 | 0.76 | 0.98 | 3.65 | -0.05 |
| 18 | E05 | 212 | ALAS2 | NM_000032 | 226697 | 247837 | 0.90 | 1.03 | -0.28 | -0.51 |
| 18 | E06 | 8854 | ALDH1A2 | NM_003888 | 229160 | 239300 | 0.91 | 0.99 | -1.28 | -0.84 |
| 18 | E07 | 220 | ALDH1A3 | NM_000693 | 220644 | 222414 | 0.87 | 0.92 | -0.82 | 0.51 |
| 18 | E08 | 219 | ALDH1B1 | NM_000692 | 219013 | 241840 | 0.87 | 1.00 | -1.20 | -1.66 |
| 18 | E09 | 217 | ALDH2 | NM_000690 | 201604 | 208962 | 0.80 | 0.87 | 0.99 | 2.12 |
| 18 | E10 | 218 | ALDH3A1 | NM_000691 | 204936 | 219426 | 0.81 | 0.91 | 0.24 | 0.31 |
| 18 | E11 | 221 | ALDH3B1 | NM_000694 | 198901 | 226831 | 0.79 | 0.94 | 0.55 | -0.77 |
| 18 | E12 | 222 | ALDH3B2 | NM_000695 | 221299 | 226661 | 0.88 | 0.94 | 0.57 | 0.48 |
| 18 | F01 | NA | NA | NA | 275562 | 240456 | 1.09 | 1.00 | -8.65 | -2.53 |
| 18 | F02 | NA | pos | NA | 84732 | 102765 | 0.34 | 0.43 | 16.06 | 15.69 |
| 18 | F03 | 8659 | ALDH4A1 | NM_003748 | 198912 | 225815 | 0.79 | 0.94 | 1.10 | 0.02 |
| 18 | F04 | 7915 | ALDH5A1 | NM_001080 | 213287 | 228662 | 0.84 | 0.95 | 0.27 | -0.01 |
| 18 | F05 | 4329 | ALDH6A1 | NM_005589 | 222444 | 234777 | 0.88 | 0.97 | -0.31 | 0.09 |
| 18 | F06 | 501 | ALDH7A1 | NM_001182 | 214622 | 242174 | 0.85 | 1.00 | 0.02 | -2.35 |
| 18 | F07 | 226 | ALDOA | NM_000034 | 176140 | 202528 | 0.70 | 0.84 | 4.36 | 2.02 |
| 18 | F08 | 55586 | ALDRL6 | NM_017584 | 189137 | 208855 | 0.75 | 0.87 | 2.09 | 1.58 |
| 18 | F09 | 10195 | ALG3 | NM_005787 | 205339 | 207925 | 0.81 | 0.86 | -0.08 | 1.13 |
| 18 | F10 | 23600 | AMACR | NM_014324 | 213586 | 227214 | 0.85 | 0.94 | -1.46 | -1.84 |
| 18 | F11 | 84883 | AMID | NM_032797 | 214585 | 221484 | 0.85 | 0.92 | -2.07 | -1.19 |
| 18 | F12 | 272 | AMPD3 | NM_000480 | 221397 | 234134 | 0.88 | 0.97 | -0.02 | -1.63 |
| 18 | G01 | NA | neg | NA | 269087 | 239321 | 1.07 | 0.99 | -6.57 | -1.89 |
| 18 | G02 | NA | neg | NA | 223591 | 230810 | 0.89 | 0.96 | -0.68 | -0.77 |
| 18 | G03 | 275 | AMT | NM_000481 | 231189 | 232074 | 0.92 | 0.96 | -1.84 | -0.32 |
| 18 | G04 | 313 | AOAH | NM_001637 | 217267 | 219846 | 0.86 | 0.91 | 1.00 | 1.65 |
| 18 | G05 | 417 | ART1 | NM_004314 | 225895 | 226524 | 0.89 | 0.94 | 0.48 | 1.67 |
| 18 | G06 | 419 | ART3 | NM_001179 | 219448 | 224483 | 0.87 | 0.93 | 0.64 | 0.48 |
| 18 | G07 | 427 | ASAH1 | NM_004315 | 212913 | 221535 | 0.84 | 0.92 | 0.84 | -0.01 |
| 18 | G08 | 440 | ASNS | NM_001673 | 227506 | 229089 | 0.90 | 0.95 | -1.64 | -0.61 |
| 18 | G09 | 443 | ASPA | NM_000049 | 229642 | 235502 | 0.91 | 0.98 | -1.98 | -2.03 |
| 18 | G10 | 471 | ATIC | NM_004044 | 226278 | 224623 | 0.90 | 0.93 | -1.86 | -1.01 |
| 18 | G11 | 522 | ATP5J | NM_001685 | 204732 | 211672 | 0.81 | 0.88 | 0.45 | 0.60 |
| 18 | G12 | 51382 | ATP6V1D | NM_015994 | 234502 | 225499 | 0.93 | 0.93 | -0.48 | 0.00 |
| 18 | H01 | NA | NA | NA | 262166 | 230458 | 1.04 | 0.96 | -5.72 | 0.02 |
| 18 | H02 | NA | pos | NA | 71206 | 80831 | 0.28 | 0.34 | 19.00 | 19.82 |
| 18 | H03 | 9296 | ATP6V1F | NM_004231 | 208222 | 222499 | 0.82 | 0.92 | 1.08 | 1.68 |
| 18 | H04 | 26053 | AUTS2 | NM_015570 | 236489 | 237843 | 0.94 | 0.99 | -1.54 | 0.01 |
| 18 | H05 | 10331 | B3GNT3 | NM_014256 | 251045 | 244657 | 0.99 | 1.01 | -2.82 | 0.01 |
| 18 | H06 | 2683 | B4GALT1 | NM_001497 | 226641 | 238884 | 0.90 | 0.99 | -0.35 | -0.69 |
| 18 | H07 | 11285 | B4GALT7 | NM_007255 | 219236 | 237296 | 0.87 | 0.98 | -0.03 | -1.36 |
| 18 | H08 | 570 | BAAT | NM_001701 | 215588 | 227451 | 0.85 | 0.94 | -0.15 | 0.35 |
| 18 | H09 | 586 | BCAT1 | NM_005504 | 210898 | 230452 | 0.83 | 0.96 | 0.39 | -0.62 |
| 18 | H10 | 587 | BCAT2 | NM_001190 | 210436 | 223142 | 0.83 | 0.92 | 0.14 | -0.08 |
| 18 | H11 | 593 | BCKDHA | NM_000709 | 204210 | 214885 | 0.81 | 0.89 | 0.47 | 0.91 |
| 18 | H12 | 594 | BCKDHB | NM_000056 | 230259 | 231111 | 0.91 | 0.96 | 0.02 | 0.00 |
| 19 | A01 | NA | pos | NA | 80209 | 85739 | 0.32 | 0.36 | 21.43 | 28.11 |
| 19 | A02 | NA | NA | NA | 267428 | 265724 | 1.05 | 1.12 | -1.49 | -2.32 |
| 19 | A03 | 622 | BDH | NM_004051 | 254232 | 256573 | 1.00 | 1.08 | 0.31 | -0.28 |
| 19 | A04 | 629 | BF | NM_001710 | 271259 | 252228 | 1.07 | 1.06 | -1.90 | 0.49 |
| 19 | A05 | 635 | BHMT | NM_001713 | 271296 | 305001 | 1.07 | 1.28 | -0.72 | -8.50 |
| 19 | A06 | 644 | BLVRA | NM_000712 | 269054 | 244522 | 1.06 | 1.03 | -0.83 | 2.21 |
| 19 | A07 | 670 | BPHL | NM_004332 | 250293 | 253070 | 0.99 | 1.06 | -0.23 | -0.18 |
| 19 | A08 | 9577 | BRE | NM_004899 | 284838 | 259643 | 1.12 | 1.09 | -3.96 | -1.46 |
| 19 | A09 | 717 | C2 | NM_000063 | 250510 | 242876 | 0.99 | 1.02 | 0.24 | -0.19 |
| 19 | A10 | 81932 | C9ORF158 | NM_031219 | 237511 | 246012 | 0.94 | 1.04 | 1.30 | 0.16 |
| 19 | A11 | 134526 | CACH-1 | NM_130767 | 241856 | 241675 | 0.95 | 1.02 | 1.58 | 0.78 |
| 19 | A12 | 55450 | CAMKIINALPHA | NM_018584 | 224032 | 235519 | 0.88 | 0.99 | 4.43 | 2.80 |
| 19 | B01 | NA | neg | NA | 295903 | 297897 | 1.17 | 1.25 | -7.64 | -10.83 |
| 19 | B02 | NA | neg | NA | 260014 | 239384 | 1.02 | 1.01 | -3.24 | -0.94 |
| 19 | B03 | 11132 | CAPN10 | NM_021251 | 228058 | 234401 | 0.90 | 0.99 | 0.85 | 0.39 |
| 19 | B04 | 23581 | CASP14 | NM_012114 | 232637 | 235859 | 0.92 | 0.99 | 0.16 | 0.18 |
| 19 | B05 | 836 | CASP3 | NM_004346 | 247763 | 241681 | 0.98 | 1.02 | -0.51 | -0.87 |
| 19 | B06 | 839 | CASP6 | NM_001226 | 240861 | 238682 | 0.95 | 1.00 | -0.04 | 0.12 |
| 19 | B07 | 840 | CASP7 | NM_001227 | 234916 | 239068 | 0.93 | 1.01 | -1.01 | -0.89 |
| 19 | B08 | 875 | CBS | NM_000071 | 237916 | 241774 | 0.94 | 1.02 | -0.88 | -1.51 |
| 19 | B09 | 952 | CD38 | NM_001775 | 234547 | 237532 | 0.92 | 1.00 | -0.47 | -2.36 |
| 19 | B10 | 8697 | CDC23 | NM_004661 | 225772 | 229268 | 0.89 | 0.96 | 0.07 | -0.08 |
| 19 | B11 | 1066 | CES1 | NM_001266 | 225993 | 222584 | 0.89 | 0.94 | 0.86 | 0.93 |
| 19 | B12 | 51716 | CES4 | NM_016280 | 238192 | 229276 | 0.94 | 0.96 | 0.03 | 0.78 |

| | | | | | | | | | | |
|---|---|---|---|---|---|---|---|---|---|---|
| 19 | C01 | NA | pos | NA | 82737 | 79073 | 0.33 | 0.33 | 16.77 | 25.73 |
| 19 | C02 | NA | NA | NA | 231037 | 231654 | 0.91 | 0.97 | -1.38 | -0.06 |
| 19 | C03 | 51011 | CGI-105 | NM_016044 | 230470 | 250653 | 0.91 | 1.05 | -1.13 | -2.79 |
| 19 | C04 | 51005 | CGI-14 | NM_015944 | 217616 | 216699 | 0.86 | 0.91 | 0.31 | 2.99 |
| 19 | C05 | 1103 | CHAT | NM_020549 | 229887 | 222978 | 0.91 | 0.94 | -0.01 | 1.86 |
| 19 | C06 | 1117 | CHI3L2 | NM_004000 | 212894 | 220490 | 0.84 | 0.93 | 1.70 | 2.76 |
| 19 | C07 | 4166 | CHST6 | NM_021615 | 212737 | 230968 | 0.84 | 0.97 | 0.02 | 0.05 |
| 19 | C08 | 1200 | CLN2 | NM_000391 | 213421 | 232487 | 0.84 | 0.98 | 0.43 | -0.37 |
| 19 | C09 | 8192 | CLPP | NM_006012 | 215808 | 216995 | 0.85 | 0.91 | 0.14 | 0.68 |
| 19 | C10 | 1352 | COX10 | NM_001303 | 224787 | 226552 | 0.89 | 0.95 | -1.49 | -0.05 |
| 19 | C11 | 1355 | COX15 | NM_004376 | 240233 | 234393 | 0.95 | 0.99 | -2.57 | -1.50 |
| 19 | C12 | 1357 | CPA1 | NM_001868 | 235084 | 240170 | 0.93 | 1.01 | -1.28 | -1.49 |
| 19 | D01 | NA | neg | NA | 260817 | 236246 | 1.03 | 0.99 | -4.65 | -0.62 |
| 19 | D02 | NA | neg | NA | 232155 | 239119 | 0.91 | 1.01 | -1.15 | -1.11 |
| 19 | D03 | 1358 | CPA2 | NM_001869 | 223735 | 222946 | 0.88 | 0.94 | 0.07 | 2.11 |
| 19 | D04 | 93979 | CPA5 | NM_080385 | 234912 | 237833 | 0.93 | 1.00 | -1.43 | -0.37 |
| 19 | D05 | 57094 | CPA6 | NM_020361 | 245181 | 242031 | 0.97 | 1.02 | -1.51 | -1.15 |
| 19 | D06 | 1360 | CPB1 | NM_001871 | 224161 | 238814 | 0.88 | 1.00 | 0.69 | -0.12 |
| 19 | D07 | 1361 | CPB2 | NM_001872 | 202954 | 235919 | 0.80 | 0.99 | 1.59 | -0.57 |
| 19 | D08 | 130749 | CPO | NM_173077 | 205781 | 227221 | 0.81 | 0.96 | 1.74 | 0.73 |
| 19 | D09 | 1374 | CPT1A | NM_001876 | 196667 | 199259 | 0.77 | 0.84 | 2.85 | 3.89 |
| 19 | D10 | 1376 | CPT2 | NM_000098 | 216282 | 214363 | 0.85 | 0.90 | -0.08 | 2.22 |
| 19 | D11 | 1384 | CRAT | NM_000755 | 238907 | 228575 | 0.94 | 0.96 | -2.04 | -0.30 |
| 19 | D12 | 54677 | CROT | NM_021151 | 229527 | 231630 | 0.90 | 0.97 | -0.22 | 0.16 |
| 19 | E01 | NA | neg | NA | 284061 | 239527 | 1.12 | 1.01 | -6.83 | -1.83 |
| 19 | E02 | NA | neg | NA | 247884 | 234696 | 0.98 | 0.99 | -2.40 | -1.02 |
| 19 | E03 | 1428 | CRYM | NM_001888 | 229927 | 222798 | 0.91 | 0.94 | -0.02 | 1.48 |
| 19 | E04 | 51380 | CSAD | NM_015989 | 228570 | 238508 | 0.90 | 1.00 | 0.01 | -1.13 |
| 19 | E05 | 9514 | GAL3ST1 | NM_004861 | 235246 | 232750 | 0.93 | 0.98 | 0.38 | -0.23 |
| 19 | E06 | 1488 | CTBP2 | NM_001329 | 245673 | 212629 | 0.97 | 0.89 | -1.27 | 3.66 |
| 19 | E07 | 1491 | CTH | NM_001902 | 216419 | 222824 | 0.85 | 0.94 | 0.61 | 0.99 |
| 19 | E08 | 1503 | CTPS | NM_001905 | 216022 | 199841 | 0.85 | 0.84 | 1.15 | 4.71 |
| 19 | E09 | 56474 | CTPS2 | NM_019857 | 214449 | 217304 | 0.84 | 0.91 | 1.34 | 0.19 |
| 19 | E10 | 1506 | CTRL | NM_001907 | 221675 | 233715 | 0.87 | 0.98 | -0.07 | -1.70 |
| 19 | E11 | 1508 | CTSB | NM_001908 | 229072 | 228683 | 0.90 | 0.96 | -0.16 | -0.97 |
| 19 | E12 | 1075 | CTSC | NM_001814 | 233405 | 233115 | 0.92 | 0.98 | -0.03 | -0.74 |
| 19 | F01 | NA | NA | NA | 291924 | 239639 | 1.15 | 1.01 | -7.38 | -1.99 |
| 19 | F02 | NA | pos | NA | 97612 | 93294 | 0.38 | 0.39 | 16.40 | 22.75 |
| 19 | F03 | 1509 | CTSD | NM_001909 | 232928 | 243914 | 0.92 | 1.03 | 0.02 | -2.23 |
| 19 | F04 | 1510 | CTSE | NM_001910 | 232132 | 235314 | 0.91 | 0.99 | -0.01 | -0.73 |
| 19 | F05 | 8722 | CTSF | NM_003793 | 241660 | 209146 | 0.95 | 0.88 | 0.01 | 3.62 |
| 19 | F06 | 1511 | CTSG | NM_001911 | 238202 | 242808 | 0.94 | 1.02 | 0.05 | -1.58 |
| 19 | F07 | 1512 | CTSH | NM_004390 | 232751 | 218942 | 0.92 | 0.92 | -0.98 | 1.51 |
| 19 | F08 | 1514 | CTSL | NM_001912 | 238412 | 223154 | 0.94 | 0.94 | -1.17 | 0.63 |
| 19 | F09 | 1515 | CTSL2 | NM_001333 | 229923 | 211155 | 0.91 | 0.89 | -0.14 | 1.09 |
| 19 | F10 | 1519 | CTSO | NM_001334 | 225647 | 235041 | 0.89 | 0.99 | -0.14 | -2.06 |
| 19 | F11 | 1520 | CTSS | NM_004079 | 229781 | 224891 | 0.90 | 0.95 | 0.16 | -0.47 |
| 19 | F12 | 1521 | CTSW | NM_001335 | 226886 | 225013 | 0.89 | 0.95 | 1.18 | 0.49 |
| 19 | G01 | NA | neg | NA | 243499 | 229602 | 0.96 | 0.97 | -2.20 | -2.00 |
| 19 | G02 | NA | neg | NA | 231932 | 225498 | 0.91 | 0.95 | -0.78 | -1.31 |
| 19 | G03 | 1522 | CTSZ | NM_001336 | 237125 | 226036 | 0.93 | 0.95 | -1.24 | -0.91 |
| 19 | G04 | 11068 | CYB561D2 | NM_007022 | 209982 | 218621 | 0.83 | 0.92 | 1.95 | 0.38 |
| 19 | G05 | 51706 | NQO3A2 | NM_016243 | 226382 | 219089 | 0.89 | 0.92 | 1.13 | 0.23 |
| 19 | G06 | 51700 | CYB5R2 | NM_016229 | 232210 | 224467 | 0.91 | 0.94 | 0.04 | -0.19 |
| 19 | G07 | 1584 | CYP11B1 | NM_000497 | 216487 | 216949 | 0.85 | 0.91 | 0.27 | 0.14 |
| 19 | G08 | 1589 | CYP21A2 | NM_000500 | 223004 | 217612 | 0.88 | 0.92 | -0.04 | -0.14 |
| 19 | G09 | 1593 | CYP27A1 | NM_000784 | 234637 | 213706 | 0.92 | 0.90 | -1.46 | -1.05 |
| 19 | G10 | 1558 | CYP2C8 | NM_000770 | 208168 | 199221 | 0.82 | 0.84 | 1.25 | 2.29 |
| 19 | G11 | 1559 | CYP2C9 | NM_000771 | 228418 | 210267 | 0.90 | 0.88 | -0.42 | 0.30 |
| 19 | G12 | 9420 | CYP7B1 | NM_004820 | 238121 | 226276 | 0.94 | 0.95 | -0.94 | -1.43 |
| 19 | H01 | NA | NA | NA | 243601 | 242445 | 0.96 | 1.02 | -2.44 | -2.93 |
| 19 | H02 | NA | pos | NA | 77457 | 83197 | 0.31 | 0.35 | 17.89 | 24.00 |
| 19 | H03 | 1610 | DAO | NM_001917 | 228078 | 226330 | 0.90 | 0.95 | -0.36 | 0.28 |
| 19 | H04 | 51163 | DBR1 | NM_016216 | 228933 | 229323 | 0.90 | 0.96 | -0.60 | -0.18 |
| 19 | H05 | 1632 | DCI | NM_001919 | 230233 | 221461 | 0.91 | 0.93 | 0.43 | 1.08 |
| 19 | H06 | 51181 | DCXR | NM_016286 | 238379 | 233528 | 0.94 | 0.98 | -0.94 | -0.48 |
| 19 | H07 | 1644 | DDC | NM_000790 | 216945 | 225423 | 0.85 | 0.95 | -0.02 | -0.05 |
| 19 | H08 | 1666 | DECR1 | NM_001359 | 220552 | 223296 | 0.87 | 0.94 | 0.04 | 0.14 |
| 19 | H09 | 26063 | DECR2 | NM_020664 | 235333 | 223576 | 0.93 | 0.94 | -1.78 | -1.47 |
| 19 | H10 | 8560 | DEGS | NM_003676 | 212202 | 219812 | 0.84 | 0.92 | 0.52 | 0.05 |
| 19 | H11 | 8694 | DGAT1 | NM_012079 | 211227 | 216466 | 0.83 | 0.91 | 1.46 | 0.50 |
| 19 | H12 | 160851 | DGKH | NM_152910 | 224416 | 226164 | 0.88 | 0.95 | 0.51 | -0.16 |
| 20 | A01 | NA | pos | NA | 65929 | 85568 | 0.28 | 0.35 | 20.26 | 29.87 |
| 20 | A02 | NA | NA | NA | 270954 | 256871 | 1.16 | 1.07 | -4.56 | -2.68 |
| 20 | A03 | 1718 | DHCR24 | NM_014762 | 229959 | 243189 | 0.98 | 1.01 | 0.98 | 0.03 |
| 20 | A04 | 1717 | DHCR7 | NM_001360 | 263897 | 251005 | 1.13 | 1.04 | -2.22 | -0.37 |
| 20 | A05 | 1719 | DHFR | NM_000791 | 192064 | 204884 | 0.82 | 0.85 | 6.29 | 8.34 |
| 20 | A06 | 50846 | DHH | NM_021044 | 249209 | 241605 | 1.06 | 1.00 | -1.61 | 0.11 |
| 20 | A07 | 1723 | DHODH | NM_001361 | 230654 | 274995 | 0.98 | 1.14 | -0.28 | -7.28 |
| 20 | A08 | 51171 | DHRS10 | NM_016246 | 239089 | 244704 | 1.02 | 1.01 | -0.60 | -0.96 |
| 20 | A09 | 51170 | DHRS8 | NM_016245 | 222617 | 248177 | 0.95 | 1.03 | 0.78 | -0.02 |
| 20 | A10 | 1727 | DIA1 | NM_000398 | 224524 | 233222 | 0.96 | 0.97 | 0.84 | 1.11 |
| 20 | A11 | 1743 | DLST | NM_001933 | 220732 | 239581 | 0.94 | 0.99 | -0.08 | -0.66 |
| 20 | A12 | 29958 | DMGDH | NM_013391 | 231816 | 243316 | 0.99 | 1.01 | 0.06 | 0.12 |
| 20 | B01 | NA | neg | NA | 263141 | 280826 | 1.12 | 1.16 | -4.48 | -10.93 |
| 20 | B02 | NA | neg | NA | 239252 | 264810 | 1.02 | 1.10 | -1.59 | -7.89 |
| 20 | B03 | 420 | DO | NM_021071 | 219015 | 214432 | 0.93 | 0.89 | 1.44 | 1.79 |
| 20 | B04 | 1800 | DPEP1 | NM_004413 | 240635 | 234439 | 1.03 | 0.97 | -0.27 | -0.93 |

| | | | | | | | | | | |
|---|---|---|---|---|---|---|---|---|---|---|
| 20 | B05 | 64174 | DPEP2 | NM_022355 | 239737 | 235556 | 1.02 | 0.98 | -0.34 | -1.19 |
| 20 | B06 | 64180 | DPEP3 | NM_022357 | 209792 | 222184 | 0.89 | 0.92 | 2.30 | 0.10 |
| 20 | B07 | 8813 | DPM1 | NM_003859 | 220953 | 217738 | 0.94 | 0.90 | 0.04 | -0.10 |
| 20 | B08 | 10072 | DPP3 | NM_005700 | 235606 | 238078 | 1.00 | 0.99 | -1.05 | -3.40 |
| 20 | B09 | 1803 | DPP4 | NM_001935 | 227743 | 234347 | 0.97 | 0.97 | -0.70 | -1.10 |
| 20 | B10 | 1807 | DPYS | NM_001385 | 224820 | 215249 | 0.96 | 0.89 | -0.06 | 0.83 |
| 20 | B11 | 63893 | E2-230K | NM_022066 | 204916 | 209061 | 0.87 | 0.87 | 0.98 | 1.44 |
| 20 | B12 | 1891 | ECH1 | NM_001398 | 210560 | 214799 | 0.90 | 0.89 | 1.77 | 1.84 |
| 20 | C01 | NA | pos | NA | 68725 | 74731 | 0.29 | 0.31 | 18.12 | 28.38 |
| 20 | C02 | NA | NA | NA | 215910 | 233320 | 0.92 | 0.97 | 0.30 | -1.75 |
| 20 | C03 | 1892 | ECHS1 | NM_004092 | 235561 | 217559 | 1.00 | 0.90 | -1.50 | 1.35 |
| 20 | C04 | 1944 | EFNA3 | NM_004952 | 214047 | 224527 | 0.91 | 0.93 | 2.01 | 1.11 |
| 20 | C05 | 1945 | EFNA4 | NM_005227 | 230295 | 230831 | 0.98 | 0.96 | -0.13 | -0.14 |
| 20 | C06 | 1949 | EFNB3 | NM_001406 | 221533 | 229505 | 0.94 | 0.95 | -0.05 | -1.14 |
| 20 | C07 | 54583 | EGLN1 | NM_022051 | 212506 | 217475 | 0.91 | 0.90 | 0.12 | 0.10 |
| 20 | C08 | 1991 | ELA2 | NM_001972 | 205951 | 211674 | 0.88 | 0.88 | 1.61 | 1.77 |
| 20 | C09 | 60528 | ELAC2 | NM_018127 | 218575 | 230703 | 0.93 | 0.96 | -0.52 | -0.25 |
| 20 | C10 | 22875 | ENPP4 | NM_014936 | 216182 | 225999 | 0.92 | 0.94 | 0.06 | -1.06 |
| 20 | C11 | 59084 | ENPP5 | NM_021572 | 217172 | 217637 | 0.93 | 0.90 | -1.44 | -0.04 |
| 20 | C12 | 2052 | EPHX1 | NM_000120 | 213735 | 225082 | 0.91 | 0.93 | 0.45 | 0.04 |
| 20 | D01 | NA | neg | NA | 234669 | 242823 | 1.00 | 1.01 | -2.35 | -4.09 |
| 20 | D02 | NA | neg | NA | 233822 | 239410 | 1.00 | 0.99 | -2.25 | -3.44 |
| 20 | D03 | 9601 | ERP70 | NM_004911 | 223600 | 223755 | 0.95 | 0.93 | -0.43 | -0.35 |
| 20 | D04 | 2098 | ESD | NM_001984 | 220032 | 220034 | 0.94 | 0.91 | 0.91 | 1.43 |
| 20 | D05 | 2110 | ETFDH | NM_004453 | 241737 | 229348 | 1.03 | 0.95 | -1.90 | -0.39 |
| 20 | D06 | 2131 | EXT1 | NM_000127 | 217771 | 220394 | 0.93 | 0.91 | 0.02 | 0.06 |
| 20 | D07 | 2132 | EXT2 | NM_000401 | 188686 | 205168 | 0.80 | 0.85 | 2.62 | 1.91 |
| 20 | D08 | 2134 | EXTL1 | NM_004455 | 216211 | 218472 | 0.92 | 0.91 | -0.02 | -0.05 |
| 20 | D09 | 2135 | EXTL2 | NM_001439 | 221378 | 225916 | 0.94 | 0.94 | -1.25 | 0.13 |
| 20 | D10 | 2137 | EXTL3 | NM_001440 | 214503 | 222125 | 0.91 | 0.92 | -0.13 | -0.86 |
| 20 | D11 | 2160 | F11 | NM_000128 | 201448 | 210584 | 0.86 | 0.87 | 0.08 | 0.77 |
| 20 | D12 | 2161 | F12 | NM_000505 | 209979 | 227219 | 0.90 | 0.94 | 0.52 | -0.90 |
| 20 | E01 | NA | neg | NA | 242961 | 252053 | 1.04 | 1.05 | -2.92 | -4.66 |
| 20 | E02 | NA | neg | NA | 234461 | 230905 | 1.00 | 0.96 | -1.89 | -0.64 |
| 20 | E03 | 23305 | ACSL6 | NM_015256 | 223070 | 228297 | 0.95 | 0.95 | 0.07 | -0.03 |
| 20 | E04 | 2184 | FAH | NM_000137 | 231438 | 235204 | 0.99 | 0.98 | -0.04 | -0.26 |
| 20 | E05 | 8789 | FBP2 | NM_003837 | 228536 | 221860 | 0.97 | 0.92 | 0.13 | 2.22 |
| 20 | E06 | 2218 | FCMD | NM_006731 | 238129 | 237628 | 1.02 | 0.99 | -2.01 | -2.02 |
| 20 | E07 | 2232 | FDXR | NM_004110 | 215120 | 215817 | 0.92 | 0.90 | -0.14 | 1.08 |
| 20 | E08 | 53834 | FGFRL1 | NM_021923 | 191362 | 218796 | 0.82 | 0.91 | 3.43 | 1.07 |
| 20 | E09 | 2272 | FHIT | NM_002012 | 206097 | 213436 | 0.88 | 0.89 | 1.04 | 3.69 |
| 20 | E10 | 51303 | FKBP11 | NM_016594 | 209165 | 223758 | 0.89 | 0.93 | 0.96 | 0.02 |
| 20 | E11 | 79147 | FKRP | NM_024301 | 220008 | 228342 | 0.94 | 0.95 | -1.73 | -1.42 |
| 20 | E12 | 55301 | THEDC1 | NM_018324 | 230061 | 228921 | 0.98 | 0.95 | -1.48 | -0.04 |
| 20 | F01 | NA | NA | NA | 239985 | 280192 | 1.02 | 1.16 | -3.51 | -12.12 |
| 20 | F02 | NA | pos | NA | 92069 | 93331 | 0.39 | 0.39 | 14.40 | 23.38 |
| 20 | F03 | 65264 | FLJ13855 | NM_023079 | 213768 | 211976 | 0.91 | 0.88 | 0.24 | 0.95 |
| 20 | F04 | 84909 | C9ORF3 | NM_032823 | 248621 | 231333 | 1.06 | 0.96 | -3.07 | -1.65 |
| 20 | F05 | 54928 | FLJ20421 | NM_017813 | 214559 | 221659 | 0.91 | 0.92 | 0.87 | 0.14 |
| 20 | F06 | 64772 | FLJ21865 | NM_022759 | 199625 | 215031 | 0.85 | 0.89 | 1.70 | 0.15 |
| 20 | F07 | 2326 | FMO1 | NM_002021 | 206388 | 211921 | 0.88 | 0.88 | -0.04 | -0.31 |
| 20 | F08 | 2329 | FMO4 | NM_002022 | 211692 | 214331 | 0.90 | 0.89 | 0.02 | -0.20 |
| 20 | F09 | 2330 | FMO5 | NM_001461 | 211060 | 221919 | 0.90 | 0.92 | -0.51 | -0.04 |
| 20 | F10 | 2339 | FNTA | NM_002027 | 211794 | 217392 | 0.90 | 0.90 | -0.31 | -0.89 |
| 20 | F11 | 2342 | FNTB | NM_002028 | 194767 | 205531 | 0.83 | 0.85 | 0.37 | 0.80 |
| 20 | F12 | 2346 | FOLH1 | NM_004476 | 210494 | 217343 | 0.90 | 0.90 | -0.06 | 0.04 |
| 20 | G01 | NA | neg | NA | 227367 | 230980 | 0.97 | 0.96 | -1.05 | -1.79 |
| 20 | G02 | NA | neg | NA | 232942 | 227530 | 0.99 | 0.94 | -1.73 | -1.13 |
| 20 | G03 | 64400 | FTS | NM_022476 | 224011 | 222749 | 0.96 | 0.92 | -0.07 | -0.11 |
| 20 | G04 | 24140 | FTSJ1 | NM_012280 | 230614 | 226492 | 0.98 | 0.94 | 0.04 | 0.26 |
| 20 | G05 | 2517 | FUCA1 | NM_000147 | 224925 | 224660 | 0.96 | 0.93 | 0.55 | 0.56 |
| 20 | G06 | 5045 | FURIN | NM_002569 | 221504 | 221325 | 0.94 | 0.92 | -0.02 | -0.06 |
| 20 | G07 | 2523 | FUT1 | NM_000148 | 237215 | 228151 | 1.01 | 0.95 | -2.84 | -2.40 |
| 20 | G08 | 2524 | FUT2 | NM_000511 | 203459 | 207794 | 0.87 | 0.86 | 1.94 | 2.03 |
| 20 | G09 | 2528 | FUT6 | NM_000150 | 206066 | 226751 | 0.88 | 0.94 | 1.02 | 0.02 |
| 20 | G10 | 2530 | FUT8 | NM_178154 | 204321 | 218012 | 0.87 | 0.90 | 1.52 | -0.02 |
| 20 | G11 | 2531 | FVT1 | NM_002035 | 211571 | 214697 | 0.90 | 0.89 | -0.73 | 0.04 |
| 20 | G12 | 486 | FXYD2 | NM_001680 | 219635 | 235146 | 0.94 | 0.98 | -0.23 | -2.35 |
| 20 | H01 | NA | NA | NA | 237212 | 247999 | 1.01 | 1.03 | -2.56 | -4.98 |
| 20 | H02 | NA | pos | NA | 78665 | 77529 | 0.34 | 0.32 | 16.64 | 27.41 |
| 20 | H03 | 2538 | G6PC | NM_000151 | 226571 | 233982 | 0.97 | 0.97 | -0.69 | -2.20 |
| 20 | H04 | 2539 | G6PD | NM_000402 | 202981 | 214507 | 0.87 | 0.89 | 3.07 | 2.58 |
| 20 | H05 | 2571 | GAD1 | NM_000817 | 233214 | 231881 | 0.99 | 0.96 | -0.77 | -0.77 |
| 20 | H06 | 2581 | GALC | NM_000153 | 217010 | 222499 | 0.93 | 0.92 | 0.21 | -0.24 |
| 20 | H07 | 2582 | GALE | NM_000403 | 206487 | 214377 | 0.88 | 0.89 | 0.57 | 0.26 |
| 20 | H08 | 2583 | GALGT | NM_001478 | 218666 | 218406 | 0.93 | 0.91 | -0.21 | 0.05 |
| 20 | H09 | 2588 | GALNS | NM_000512 | 207671 | 208576 | 0.89 | 0.87 | 0.51 | 3.52 |
| 20 | H10 | 2592 | GALT | NM_000155 | 222096 | 216560 | 0.95 | 0.90 | -0.94 | 0.30 |
| 20 | H11 | 2593 | GAMT | NM_000156 | 195959 | 215373 | 0.84 | 0.89 | 0.84 | -0.05 |
| 20 | H12 | 2597 | GAPDH | NM_002046 | 218116 | 230393 | 0.93 | 0.96 | -0.36 | -1.41 |
| 21 | A01 | NA | NA | NA | 285075 | 304349 | 1.15 | 1.25 | -3.98 | -3.11 |
| 21 | A02 | NA | pos | NA | 173215 | 149577 | 0.70 | 0.62 | 11.05 | 13.57 |
| 21 | A03 | 2618 | GART | NM_000819 | 296140 | 277907 | 1.19 | 1.15 | -5.25 | -0.26 |
| 21 | A04 | 2628 | GATM | NM_001482 | 299052 | 323711 | 1.20 | 1.33 | -5.06 | -5.14 |
| 21 | A05 | 2629 | GBA | NM_000157 | 305850 | 247646 | 1.23 | 1.02 | -5.70 | 2.45 |
| 21 | A06 | 2639 | GCDH | NM_000159 | 275286 | 282452 | 1.11 | 1.16 | -2.55 | -0.59 |
| 21 | A07 | 2643 | GCH1 | NM_000161 | 248043 | 269609 | 1.00 | 1.11 | 1.01 | 0.19 |
| 21 | A08 | 2651 | GCNT2 | NM_001491 | 250408 | 276593 | 1.01 | 1.14 | 0.67 | -0.21 |

| | | | | | | | | | | |
|---|---|---|---|---|---|---|---|---|---|---|
| 21 | A09 | 2653 | GCSH | NM_004483 | 231308 | 289321 | 0.93 | 1.19 | 1.48 | -1.46 |
| 21 | A10 | 2677 | GGCX | NM_000821 | 233262 | 252395 | 0.94 | 1.04 | 1.42 | 2.57 |
| 21 | A11 | 8836 | GGH | NM_003878 | 237518 | 236673 | 0.96 | 0.98 | 1.56 | 3.00 |
| 21 | A12 | 9453 | GGPS1 | NM_004837 | 255401 | 256899 | 1.03 | 1.06 | -0.67 | 2.76 |
| 21 | B01 | NA | neg | NA | 285855 | 289966 | 1.15 | 1.19 | -6.40 | -4.33 |
| 21 | B02 | NA | neg | NA | 248961 | 226676 | 1.00 | 0.93 | -1.45 | 2.49 |
| 21 | B03 | 2717 | GLA | NM_000169 | 229158 | 239557 | 0.92 | 0.99 | 1.42 | 1.10 |
| 21 | B04 | 2720 | GLB1 | NM_000404 | 235017 | 253167 | 0.95 | 1.04 | 1.22 | -0.30 |
| 21 | B05 | 2731 | GLDC | NM_000170 | 248475 | 246068 | 1.00 | 1.01 | -0.32 | -0.15 |
| 21 | B06 | 51022 | GLRX2 | NM_016066 | 245022 | 233683 | 0.99 | 0.96 | -0.81 | 1.90 |
| 21 | B07 | 51557 | GLULD1 | NM_016571 | 231201 | 241045 | 0.93 | 0.99 | 0.95 | 0.50 |
| 21 | B08 | 2766 | GMPR | NM_006877 | 246751 | 250587 | 0.99 | 1.03 | -1.16 | -0.18 |
| 21 | B09 | 51292 | GMPR2 | NM_016576 | 226030 | 256097 | 0.91 | 1.06 | -0.13 | -0.65 |
| 21 | B10 | 2790 | GNG10 | NM_004125 | 225632 | 264335 | 0.91 | 1.09 | 0.12 | -1.48 |
| 21 | B11 | 8443 | GNPAT | NM_014236 | 233640 | 237723 | 0.94 | 0.98 | -0.24 | 0.12 |
| 21 | B12 | 10007 | GNPDA1 | NM_005471 | 225928 | 236472 | 0.91 | 0.97 | 0.96 | 2.19 |
| 21 | C01 | NA | NA | NA | 256990 | 240983 | 1.04 | 0.99 | -2.25 | -0.65 |
| 21 | C02 | NA | pos | NA | 187374 | 162923 | 0.75 | 0.67 | 7.10 | 7.77 |
| 21 | C03 | 2799 | GNS | NM_002076 | 234861 | 236154 | 0.95 | 0.97 | 0.94 | -0.13 |
| 21 | C04 | 10243 | GPHN | NM_020806 | 247985 | 207835 | 1.00 | 0.86 | -0.25 | 2.99 |
| 21 | C05 | 9380 | GRHPR | NM_012203 | 207312 | 219278 | 0.84 | 0.90 | 5.49 | 1.15 |
| 21 | C06 | 2923 | GRP58 | NM_005313 | 243577 | 234914 | 0.98 | 0.97 | -0.33 | 0.17 |
| 21 | C07 | 10533 | APG7L | NM_006395 | 251695 | 208932 | 1.01 | 0.86 | -1.53 | 2.36 |
| 21 | C08 | 2938 | GSTA1 | NM_145740 | 227942 | 193728 | 0.92 | 0.80 | 1.64 | 4.36 |
| 21 | C09 | 2944 | GSTM1 | NM_000561 | 234206 | 238445 | 0.94 | 0.98 | -0.95 | -0.34 |
| 21 | C10 | 2947 | GSTM3 | NM_000849 | 228742 | 240389 | 0.92 | 0.99 | -0.02 | -0.50 |
| 21 | C11 | 9446 | GSTO1 | NM_004832 | 207600 | 243188 | 0.84 | 1.00 | 3.54 | -2.07 |
| 21 | C12 | 2977 | GUCY1A2 | NM_000855 | 235104 | 245344 | 0.95 | 1.01 | 0.01 | -0.37 |
| 21 | D01 | NA | neg | NA | 234608 | 243366 | 0.95 | 1.00 | -0.17 | -1.24 |
| 21 | D02 | NA | neg | NA | 250570 | 243729 | 1.01 | 1.00 | -2.32 | -1.28 |
| 21 | D03 | 2982 | GUCY1A3 | NM_000856 | 234959 | 283916 | 0.95 | 1.17 | -0.01 | -5.61 |
| 21 | D04 | 2983 | GUCY1B3 | NM_000857 | 232849 | 223061 | 0.94 | 0.92 | 0.86 | 1.01 |
| 21 | D05 | 2990 | GUSB | NM_000181 | 241157 | 213084 | 0.97 | 0.88 | 0.01 | 1.48 |
| 21 | D06 | 2997 | GYS1 | NM_002103 | 235455 | 241295 | 0.95 | 0.99 | -0.17 | -0.85 |
| 21 | D07 | 2998 | GYS2 | NM_021957 | 242120 | 251617 | 0.98 | 1.04 | -1.17 | -2.58 |
| 21 | D08 | 3001 | GZMA | NM_006144 | 230078 | 210777 | 0.93 | 0.87 | 0.43 | 2.18 |
| 21 | D09 | 3002 | GZMB | NM_004131 | 217955 | 226151 | 0.88 | 0.93 | 0.30 | 0.65 |
| 21 | D10 | 50814 | NSDHL | NM_015922 | 234318 | 240589 | 0.94 | 0.99 | -1.70 | -0.86 |
| 21 | D11 | 9555 | H2AFY | NM_004893 | 221517 | 221053 | 0.89 | 0.91 | 0.73 | -0.02 |
| 21 | D12 | 3030 | HADHA | NM_000182 | 228956 | 238643 | 0.92 | 0.98 | -0.10 | 0.02 |
| 21 | E01 | NA | neg | NA | 247727 | 233003 | 1.00 | 0.96 | -2.29 | -0.83 |
| 21 | E02 | NA | neg | NA | 245904 | 224028 | 0.99 | 0.92 | -2.05 | 0.13 |
| 21 | E03 | 3033 | HADHSC | NM_005327 | 234677 | 224076 | 0.95 | 0.92 | -0.33 | 0.13 |
| 21 | E04 | 3029 | HAGH | NM_005326 | 243311 | 218630 | 0.98 | 0.90 | -0.91 | 0.78 |
| 21 | E05 | 3034 | HAL | NM_002108 | 234688 | 231475 | 0.95 | 0.95 | 0.52 | -1.22 |
| 21 | E06 | 9407 | HAT | NM_004262 | 209624 | 228386 | 0.84 | 0.94 | 2.94 | -0.17 |
| 21 | E07 | 3073 | HEXA | NM_000520 | 214733 | 203619 | 0.87 | 0.84 | 2.15 | 1.89 |
| 21 | E08 | 3074 | HEXB | NM_000521 | 239179 | 244083 | 0.96 | 1.01 | -1.15 | -2.12 |
| 21 | E09 | 3081 | HGD | NM_000187 | 205363 | 220602 | 0.83 | 0.91 | 1.64 | 0.54 |
| 21 | E10 | 3083 | HGFAC | NM_001528 | 218867 | 216494 | 0.88 | 0.89 | 0.02 | 1.03 |
| 21 | E11 | 55662 | HIF1AN | NM_017902 | 224509 | 230420 | 0.90 | 0.95 | -0.02 | -1.74 |
| 21 | E12 | 3141 | HLCS | NM_000411 | 227537 | 233224 | 0.92 | 0.96 | -0.26 | -0.11 |
| 21 | F01 | NA | pos | NA | 138750 | 128077 | 0.56 | 0.53 | 12.60 | 11.44 |
| 21 | F02 | NA | NA | NA | 239838 | 242640 | 0.97 | 1.00 | -0.98 | -0.91 |
| 21 | F03 | 3145 | HMBS | NM_000190 | 234091 | 232029 | 0.94 | 0.96 | 0.01 | 0.23 |
| 21 | F04 | 3155 | HMGCL | NM_000191 | 236640 | 254157 | 0.95 | 1.05 | 0.25 | -2.09 |
| 21 | F05 | 3156 | HMGCR | NM_000859 | 230979 | 227780 | 0.93 | 0.94 | 1.28 | 0.15 |
| 21 | F06 | 3157 | HMGCS1 | NM_002130 | 219389 | 238811 | 0.88 | 0.98 | 1.88 | -0.33 |
| 21 | F07 | 3158 | HMGCS2 | NM_005518 | 249101 | 231838 | 1.00 | 0.96 | -2.21 | -0.19 |
| 21 | F08 | 3162 | HMOX1 | NM_002133 | 235665 | 237653 | 0.95 | 0.98 | -0.43 | -0.46 |
| 21 | F09 | 3163 | HMOX2 | NM_002134 | 223277 | 218850 | 0.90 | 0.90 | -0.51 | 1.69 |
| 21 | F10 | 3176 | HNMT | NM_006895 | 223687 | 213752 | 0.90 | 0.88 | -0.37 | 2.29 |
| 21 | F11 | 3275 | HRMT1L1 | NM_001535 | 226266 | 226275 | 0.91 | 0.93 | 0.00 | -0.33 |
| 21 | F12 | 3276 | HRMT1L2 | NM_001536 | 223247 | 233349 | 0.90 | 0.96 | 0.57 | 0.84 |
| 21 | G01 | NA | neg | NA | 248577 | 264408 | 1.00 | 1.09 | -2.58 | -4.01 |
| 21 | G02 | NA | neg | NA | 247858 | 241974 | 1.00 | 1.00 | -2.49 | -1.59 |
| 21 | G03 | 3290 | HSD11B1 | NM_005525 | 236105 | 240770 | 0.95 | 0.99 | -0.70 | -1.46 |
| 21 | G04 | 3291 | HSD11B2 | NM_000196 | 232256 | 225031 | 0.94 | 0.93 | 0.40 | 0.30 |
| 21 | G05 | 3292 | HSD17B1 | NM_000413 | 238481 | 234683 | 0.96 | 0.97 | -0.16 | -1.35 |
| 21 | G06 | 51144 | HSD17B12 | NM_016142 | 228248 | 218589 | 0.92 | 0.90 | 0.26 | 1.10 |
| 21 | G07 | 3294 | HSD17B2 | NM_002153 | 229053 | 233821 | 0.92 | 0.96 | 0.05 | -1.16 |
| 21 | G08 | 3293 | HSD17B3 | NM_000197 | 236723 | 224775 | 0.95 | 0.93 | -1.00 | 0.18 |
| 21 | G09 | 51478 | HSD17B7 | NM_016371 | 215263 | 229231 | 0.87 | 0.94 | 0.13 | -0.18 |
| 21 | G10 | 29089 | HSPC150 | NM_014176 | 226076 | 227421 | 0.91 | 0.94 | -1.13 | 0.06 |
| 21 | G11 | 3373 | HYAL1 | NM_007312 | 223288 | 210239 | 0.90 | 0.87 | -0.04 | 0.65 |
| 21 | G12 | 3423 | IDS | NM_000202 | 224021 | 234665 | 0.90 | 0.97 | 0.03 | -0.05 |
| 21 | H01 | NA | pos | NA | 123552 | 101395 | 0.50 | 0.42 | 15.34 | 14.28 |
| 21 | H02 | NA | NA | NA | 284386 | 295882 | 1.15 | 1.22 | -6.26 | -6.68 |
| 21 | H03 | 3425 | IDUA | NM_000203 | 233804 | 232491 | 0.94 | 0.96 | 0.75 | 0.15 |
| 21 | H04 | 3426 | IF | NM_000204 | 308324 | 250432 | 1.24 | 1.03 | -8.68 | -1.72 |
| 21 | H05 | 10437 | IFI30 | NM_006332 | 245800 | 250480 | 0.99 | 1.03 | -0.01 | -2.34 |
| 21 | H06 | 3615 | IMPDH2 | NM_000884 | 237329 | 226332 | 0.96 | 0.93 | 0.17 | 0.98 |
| 21 | H07 | 8821 | INPP4B | NM_003866 | 238205 | 234157 | 0.96 | 0.96 | -0.05 | -0.47 |
| 21 | H08 | 3632 | INPP5A | NM_005539 | 232081 | 225681 | 0.93 | 0.93 | 0.76 | 0.80 |
| 21 | H09 | 3633 | INPP5B | NM_005540 | 231673 | 232537 | 0.93 | 0.96 | -0.94 | 0.18 |
| 21 | H10 | 140838 | HDHD4 | NM_152667 | 225392 | 235255 | 0.91 | 0.97 | 0.10 | -0.06 |
| 21 | H11 | 2678 | GGT1 | NM_005265 | 231422 | 222732 | 0.93 | 0.92 | 0.00 | 0.02 |
| 21 | H12 | 2679 | GGT2 | XM_290331 | 232776 | 241048 | 0.94 | 0.99 | -0.01 | -0.02 |

| | | | | | | | | | | |
|---|---|---|---|---|---|---|---|---|---|---|
| 22 | A01 | NA | NA | NA | 293750 | 292824 | 1.19 | 1.20 | -6.07 | -6.40 |
| 22 | A02 | NA | pos | NA | 141974 | 137515 | 0.57 | 0.56 | 14.56 | 12.47 |
| 22 | A03 | 3635 | INPP5D | NM_005541 | 279200 | 279867 | 1.13 | 1.14 | -2.87 | -3.71 |
| 22 | A04 | 56623 | INPP5E | NM_019892 | 241057 | 225460 | 0.97 | 0.92 | 1.06 | 2.18 |
| 22 | A05 | 3669 | ISG20 | NM_002201 | 299213 | 237807 | 1.21 | 0.97 | -5.33 | 1.03 |
| 22 | A06 | 3704 | ITPA | NM_033453 | 294156 | 297195 | 1.19 | 1.21 | -5.78 | -6.57 |
| 22 | A07 | 3712 | IVD | NM_002225 | 294063 | 242166 | 1.19 | 0.99 | -6.25 | 0.00 |
| 22 | A08 | 23262 | KIAA0433 | NM_015216 | 256684 | 230224 | 1.04 | 0.94 | -1.02 | 0.73 |
| 22 | A09 | 23028 | AOF2 | NM_015013 | 241339 | 237314 | 0.97 | 0.97 | 1.04 | 0.00 |
| 22 | A10 | 23382 | KIAA0828 | NM_015328 | 233771 | 231621 | 0.94 | 0.95 | 3.14 | 0.37 |
| 22 | A11 | 23324 | MAN2B2 | XM_052620 | 235844 | 238763 | 0.95 | 0.98 | 1.17 | -0.15 |
| 22 | A12 | 57552 | KIAA1363 | NM_020792 | 240292 | 251796 | 0.97 | 1.03 | 1.02 | -1.65 |
| 22 | B01 | NA | neg | NA | 308254 | 289680 | 1.24 | 1.18 | -10.22 | -7.29 |
| 22 | B02 | NA | neg | NA | 226743 | 229640 | 0.92 | 0.94 | 0.86 | 0.00 |
| 22 | B03 | 3817 | KLK2 | NM_005551 | 219565 | 223750 | 0.89 | 0.91 | 3.06 | 1.82 |
| 22 | B04 | 354 | KLK3 | NM_001648 | 231712 | 220516 | 0.94 | 0.90 | 0.15 | 1.51 |
| 22 | B05 | 3818 | KLKB1 | NM_000892 | 245847 | 217873 | 0.99 | 0.89 | -0.25 | 2.17 |
| 22 | B06 | 8942 | KYNU | NM_003937 | 217651 | 232038 | 0.88 | 0.95 | 2.45 | 0.07 |
| 22 | B07 | 9215 | LARGE | NM_004737 | 227102 | 232162 | 0.92 | 0.95 | 0.68 | -0.07 |
| 22 | B08 | 51451 | LCMT1 | NM_016015 | 236073 | 241534 | 0.95 | 0.99 | -0.40 | -1.93 |
| 22 | B09 | 64129 | LCN7 | NM_022164 | 245042 | 245983 | 0.99 | 1.00 | -1.64 | -2.33 |
| 22 | B10 | 3938 | LCT | NM_002299 | 243932 | 249474 | 0.98 | 1.02 | -0.41 | -3.08 |
| 22 | B11 | 79042 | LENG5 | NM_024075 | 229558 | 229269 | 0.93 | 0.94 | -0.15 | -0.27 |
| 22 | B12 | 5641 | LGMN | NM_005606 | 224813 | 218951 | 0.91 | 0.89 | 0.95 | 1.05 |
| 22 | C01 | NA | NA | NA | 292630 | 307793 | 1.18 | 1.26 | -8.52 | -9.19 |
| 22 | C02 | NA | pos | NA | 134453 | 144571 | 0.54 | 0.59 | 12.99 | 10.64 |
| 22 | C03 | 64077 | LHPP | NM_022126 | 239028 | 235353 | 0.96 | 0.96 | 0.00 | 0.72 |
| 22 | C04 | 3988 | LIPA | NM_000235 | 213050 | 245356 | 0.86 | 1.00 | 2.27 | -1.20 |
| 22 | C05 | 89782 | LMLN | NM_033029 | 242248 | 244294 | 0.98 | 1.00 | -0.19 | -0.73 |
| 22 | C06 | 134147 | LOC134147 | NM_138809 | 234076 | 218031 | 0.94 | 0.89 | -0.20 | 2.08 |
| 22 | C07 | 51619 | LOC51619 | NM_015983 | 237361 | 243539 | 0.96 | 0.99 | -1.14 | -1.14 |
| 22 | C08 | 63929 | LOC63929 | NM_022098 | 214917 | 214023 | 0.87 | 0.87 | 2.06 | 1.72 |
| 22 | C09 | 9227 | LRAT | NM_004744 | 229877 | 235341 | 0.93 | 0.96 | 0.00 | -0.73 |
| 22 | C10 | 4047 | LSS | NM_002340 | 246717 | 215349 | 1.00 | 0.88 | -1.21 | 1.37 |
| 22 | C11 | 10434 | LYPLA1 | NM_006330 | 219266 | 230733 | 0.88 | 0.94 | 0.82 | -0.14 |
| 22 | C12 | 11313 | LYPLA2 | NM_007260 | 227814 | 228975 | 0.92 | 0.94 | 0.12 | 0.14 |
| 22 | D01 | NA | neg | NA | 287298 | 252070 | 1.16 | 1.03 | -8.27 | -3.40 |
| 22 | D02 | NA | neg | NA | 236232 | 240595 | 0.95 | 0.98 | -1.33 | -2.00 |
| 22 | D03 | 23659 | LYPLA3 | NM_012320 | 238921 | 232763 | 0.96 | 0.95 | -0.47 | 0.06 |
| 22 | D04 | 4124 | MAN2A1 | NM_002372 | 238749 | 224067 | 0.96 | 0.91 | -1.71 | 0.40 |
| 22 | D05 | 4125 | MAN2B1 | NM_000528 | 235960 | 243739 | 0.95 | 1.00 | 0.19 | -1.64 |
| 22 | D06 | 4126 | MANBA | NM_005908 | 239265 | 227529 | 0.97 | 0.93 | -1.39 | -0.06 |
| 22 | D07 | 4128 | MAOA | NM_000240 | 223289 | 209324 | 0.90 | 0.85 | 0.29 | 2.04 |
| 22 | D08 | 23118 | MAP3K7IP2 | NM_015093 | 213749 | 226692 | 0.86 | 0.93 | 1.73 | -0.80 |
| 22 | D09 | 58478 | MASA | NM_021204 | 227722 | 210569 | 0.92 | 0.86 | -0.19 | 1.30 |
| 22 | D10 | 8720 | MBTPS1 | NM_003791 | 237425 | 221160 | 0.96 | 0.90 | -0.43 | -0.31 |
| 22 | D11 | 64087 | MCCC2 | NM_022132 | 217990 | 200916 | 0.88 | 0.82 | 0.51 | 2.50 |
| 22 | D12 | 4199 | ME1 | NM_002395 | 221636 | 223291 | 0.89 | 0.91 | 0.48 | -0.14 |
| 22 | E01 | NA | neg | NA | 300856 | 284740 | 1.21 | 1.16 | -10.45 | -7.49 |
| 22 | E02 | NA | neg | NA | 243480 | 234452 | 0.98 | 0.96 | -2.65 | -1.38 |
| 22 | E03 | 4232 | MEST | NM_002402 | 233011 | 224096 | 0.94 | 0.92 | 0.00 | 0.99 |
| 22 | E04 | 23173 | METAP1 | NM_015143 | 253179 | 237258 | 1.02 | 0.97 | -4.00 | -1.32 |
| 22 | E05 | 80168 | MOGAT2 | NM_025098 | 229021 | 226867 | 0.92 | 0.93 | 0.80 | 0.29 |
| 22 | E06 | 11320 | MGAT4A | NM_012214 | 226612 | 226053 | 0.91 | 0.92 | 0.00 | 0.00 |
| 22 | E07 | 66005 | MGC3234 | NM_023947 | 225044 | 225055 | 0.91 | 0.92 | -0.28 | 0.00 |
| 22 | E08 | 148789 | MGC39558 | NM_152490 | 222975 | 218518 | 0.90 | 0.89 | 0.15 | 0.07 |
| 22 | E09 | 79154 | MGC4172 | NM_024308 | 208880 | 179646 | 0.84 | 0.73 | 2.04 | 4.93 |
| 22 | E10 | 10724 | MGEA5 | NM_012215 | 228776 | 230994 | 0.92 | 0.94 | 0.41 | -1.63 |
| 22 | E11 | 4257 | MGST1 | NM_020300 | 219366 | 220942 | 0.89 | 0.90 | 0.00 | -0.06 |
| 22 | E12 | 23417 | MLYCD | NM_012213 | 237281 | 225598 | 0.96 | 0.92 | -1.98 | -0.55 |
| 22 | F01 | NA | pos | NA | 117727 | 102760 | 0.48 | 0.42 | 15.07 | 15.54 |
| 22 | F02 | NA | NA | NA | 233496 | 231251 | 0.94 | 0.94 | -0.66 | -0.07 |
| 22 | F03 | 118856 | MMP21 | NM_147191 | 244760 | 248249 | 0.99 | 1.01 | -0.97 | -1.02 |
| 22 | F04 | 64066 | MMP27 | NM_022122 | 228005 | 233961 | 0.92 | 0.96 | 0.05 | 0.00 |
| 22 | F05 | 4337 | MOCS1 | NM_005943 | 225703 | 230171 | 0.91 | 0.94 | 1.88 | 0.81 |
| 22 | F06 | 4338 | MOCS2 | NM_004531 | 230757 | 233701 | 0.93 | 0.95 | 0.06 | 0.00 |
| 22 | F07 | 10884 | MRPS30 | NM_016640 | 231946 | 233169 | 0.94 | 0.95 | -0.59 | -0.06 |
| 22 | F08 | 4482 | MSRA | NM_012331 | 229083 | 236839 | 0.92 | 0.97 | -0.05 | -1.23 |
| 22 | F09 | 4507 | MTAP | NM_002451 | 220401 | 230807 | 0.89 | 0.94 | 1.10 | -0.36 |
| 22 | F10 | 4524 | MTHFR | NM_005957 | 225430 | 221140 | 0.91 | 0.90 | 1.50 | 0.49 |
| 22 | F11 | 4552 | MTRR | NM_002454 | 226551 | 227624 | 0.91 | 0.93 | -0.35 | 0.06 |
| 22 | F12 | 4669 | NAGLU | NM_000263 | 233995 | 222109 | 0.94 | 0.91 | -0.90 | 0.80 |
| 22 | G01 | NA | neg | NA | 252084 | 249174 | 1.02 | 1.02 | -4.40 | -2.75 |
| 22 | G02 | NA | neg | NA | 233173 | 235718 | 0.94 | 0.96 | -1.83 | -1.11 |
| 22 | G03 | 10 | NAT2 | NM_000015 | 226710 | 237638 | 0.91 | 0.97 | 0.27 | -0.23 |
| 22 | G04 | 9027 | NAT8 | NM_003960 | 224086 | 229886 | 0.90 | 0.94 | -0.63 | 0.00 |
| 22 | G05 | 51167 | NCB5OR | NM_016230 | 238337 | 235070 | 0.96 | 0.96 | -1.05 | -0.29 |
| 22 | G06 | 96764 | NCOA6IP | NM_024831 | 222278 | 229524 | 0.90 | 0.94 | 0.00 | 0.00 |
| 22 | G07 | 64579 | NDST4 | NM_022569 | 216666 | 216846 | 0.87 | 0.89 | 0.28 | 1.42 |
| 22 | G08 | 4720 | NDUFS2 | NM_004550 | 219789 | 220964 | 0.89 | 0.90 | 0.00 | 0.20 |
| 22 | G09 | 4724 | NDUFS4 | NM_002495 | 226561 | 223755 | 0.91 | 0.91 | -0.94 | 0.00 |
| 22 | G10 | 374291 | NDUFS7 | NM_024407 | 221620 | 218507 | 0.89 | 0.89 | 0.80 | 0.31 |
| 22 | G11 | 4729 | NDUFV2 | NM_021074 | 215037 | 199725 | 0.87 | 0.82 | 0.00 | 2.94 |
| 22 | G12 | 4758 | NEU1 | NM_000434 | 227620 | 232804 | 0.92 | 0.95 | -1.25 | -1.00 |
| 22 | H01 | NA | pos | NA | 88576 | 122270 | 0.36 | 0.50 | 19.20 | 13.41 |
| 22 | H02 | NA | NA | NA | 304330 | 266830 | 1.23 | 1.09 | -10.13 | -4.14 |
| 22 | H03 | 56954 | NIT2 | NM_020202 | 229088 | 242330 | 0.92 | 0.99 | 1.32 | -0.06 |
| 22 | H04 | 4837 | NNMT | NM_006169 | 229935 | 241909 | 0.93 | 0.99 | -0.05 | -0.72 |

| | | | | | | | | | | |
|---|---|---|---|---|---|---|---|---|---|---|
| 22 | H05 | 23530 | NNT | NM_012343 | 236094 | 258537 | 0.95 | 1.06 | 0.63 | -2.39 |
| 22 | H06 | 4860 | NP | NM_000270 | 228587 | 235195 | 0.92 | 0.96 | 0.52 | 0.06 |
| 22 | H07 | 1728 | NQO1 | NM_000903 | 214180 | 202776 | 0.86 | 0.83 | 1.99 | 3.88 |
| 22 | H08 | 8829 | NRP1 | NM_003873 | 229869 | 229339 | 0.93 | 0.94 | 0.00 | -0.07 |
| 22 | H09 | 51251 | NT5C3 | NM_016489 | 229715 | 228131 | 0.93 | 0.93 | 0.00 | 0.21 |
| 22 | H10 | 4907 | NT5E | NM_002526 | 242215 | 232684 | 0.98 | 0.95 | -0.63 | -0.67 |
| 22 | H11 | 4521 | NUDT1 | NM_002452 | 228445 | 212694 | 0.92 | 0.87 | -0.45 | 2.11 |
| 22 | H12 | 11163 | NUDT4 | NM_019094 | 229460 | 223005 | 0.93 | 0.91 | -0.12 | 0.94 |
| 23 | A01 | NA | pos | NA | 123058 | 112396 | 0.51 | 0.45 | 14.61 | 16.47 |
| 23 | A02 | NA | NA | NA | 266183 | 239296 | 1.11 | 0.95 | -1.63 | 0.73 |
| 23 | A03 | 4942 | OAT | NM_000274 | 276751 | 293708 | 1.15 | 1.17 | -2.90 | -5.11 |
| 23 | A04 | 4953 | ODC1 | NM_002539 | 299981 | 239366 | 1.25 | 0.95 | -4.74 | 0.43 |
| 23 | A05 | 4967 | OGDH | NM_002541 | 229883 | 265756 | 0.96 | 1.06 | 2.54 | -2.86 |
| 23 | A06 | 5019 | OXCT1 | NM_000436 | 240899 | 246622 | 1.00 | 0.98 | 0.90 | 0.20 |
| 23 | A07 | 8909 | P11 | NM_006025 | 261107 | 236165 | 1.09 | 0.94 | -1.13 | 1.18 |
| 23 | A08 | 5033 | P4HA1 | NM_000917 | 251129 | 240153 | 1.04 | 0.96 | 0.91 | 0.79 |
| 23 | A09 | 5034 | P4HB | NM_000918 | 296842 | 250500 | 1.23 | 1.00 | -4.80 | -1.60 |
| 23 | A10 | 29920 | PYCR2 | NM_013328 | 231362 | 232227 | 0.96 | 0.93 | 2.63 | 1.61 |
| 23 | A11 | 5036 | PA2G4 | NM_006191 | 249903 | 245143 | 1.04 | 0.98 | -0.87 | -0.48 |
| 23 | A12 | 5046 | PCSK6 | NM_002570 | 241802 | 246947 | 1.00 | 0.98 | 1.11 | -0.25 |
| 23 | B01 | NA | neg | NA | 286039 | 298136 | 1.19 | 1.19 | -5.81 | -8.04 |
| 23 | B02 | NA | neg | NA | 232921 | 240635 | 0.97 | 0.96 | 0.22 | -0.91 |
| 23 | B03 | 5049 | PAFAH1B2 | NM_002572 | 227116 | 224376 | 0.94 | 0.89 | 0.81 | 2.02 |
| 23 | B04 | 5050 | PAFAH1B3 | NM_002573 | 237151 | 232751 | 0.99 | 0.93 | 0.47 | -0.22 |
| 23 | B05 | 5096 | PCCB | NM_000532 | 223789 | 230225 | 0.93 | 0.92 | 1.31 | 0.08 |
| 23 | B06 | 5122 | PCSK1 | NM_000439 | 235106 | 223984 | 0.98 | 0.89 | -0.36 | 1.53 |
| 23 | B07 | 9159 | PCSK7 | NM_004716 | 234225 | 229307 | 0.97 | 0.91 | 0.00 | 0.56 |
| 23 | B08 | 9131 | PDCD8 | NM_004208 | 245011 | 242252 | 1.02 | 0.97 | -0.32 | -0.94 |
| 23 | B09 | 5138 | PDE2A | NM_002599 | 227249 | 242899 | 0.94 | 0.97 | 1.17 | -2.13 |
| 23 | B10 | 5139 | PDE3A | NM_000921 | 255085 | 252850 | 1.06 | 1.01 | -1.98 | -2.42 |
| 23 | B11 | 5140 | PDE3B | NM_000922 | 231017 | 230438 | 0.96 | 0.92 | -0.65 | -0.12 |
| 23 | B12 | 5141 | PDE4A | NM_006202 | 234665 | 231133 | 0.98 | 0.92 | 0.00 | 0.25 |
| 23 | C01 | NA | pos | NA | 119030 | 110454 | 0.49 | 0.44 | 13.15 | 15.54 |
| 23 | C02 | NA | NA | NA | 258278 | 251649 | 1.07 | 1.00 | -2.66 | -1.98 |
| 23 | C03 | 5142 | PDE4B | NM_002600 | 233183 | 243535 | 0.97 | 0.97 | 0.12 | -0.06 |
| 23 | C04 | 5143 | PDE4C | NM_000923 | 221623 | 234718 | 0.92 | 0.94 | 2.24 | -0.17 |
| 23 | C05 | 5144 | PDE4D | NM_006203 | 242455 | 233052 | 1.01 | 0.93 | -0.81 | 0.03 |
| 23 | C06 | 5147 | PDE6D | NM_002601 | 219219 | 240880 | 0.91 | 0.96 | 1.44 | -0.26 |
| 23 | C07 | 5150 | PDE7A | NM_002603 | 212004 | 236546 | 0.88 | 0.94 | 2.52 | -0.04 |
| 23 | C08 | 27115 | PDE7B | NM_018945 | 247917 | 208822 | 1.03 | 0.83 | -0.65 | 3.50 |
| 23 | C09 | 5157 | PDGFRL | NM_006207 | 243113 | 214127 | 1.01 | 0.85 | -0.63 | 1.74 |
| 23 | C10 | 5160 | PDHA1 | NM_000284 | 238422 | 223134 | 0.99 | 0.89 | -0.09 | 1.57 |
| 23 | C11 | 5161 | PDHA2 | NM_005390 | 206502 | 230878 | 0.86 | 0.92 | 2.13 | 0.12 |
| 23 | C12 | 5162 | PDHB | NM_000925 | 242476 | 242907 | 1.01 | 0.97 | -0.89 | -0.92 |
| 23 | D01 | NA | neg | NA | 257316 | 309481 | 1.07 | 1.23 | -2.52 | -8.77 |
| 23 | D02 | NA | neg | NA | 239721 | 248620 | 1.00 | 0.99 | -0.52 | -1.22 |
| 23 | D03 | 64714 | PDIP | NM_006849 | 221632 | 249647 | 0.92 | 0.99 | 1.46 | -0.43 |
| 23 | D04 | 10954 | PDIR | NM_006810 | 221521 | 235148 | 0.92 | 0.94 | 2.28 | 0.17 |
| 23 | D05 | 55066 | PDPR | NM_017990 | 243989 | 253372 | 1.01 | 1.01 | -0.95 | -2.11 |
| 23 | D06 | 55825 | PECR | NM_018441 | 242448 | 243432 | 1.01 | 0.97 | -1.16 | -0.20 |
| 23 | D07 | 56652 | PEO1 | NM_021830 | 240916 | 226665 | 1.00 | 0.90 | -0.73 | 1.57 |
| 23 | D08 | 5223 | PGAM1 | NM_002629 | 250864 | 216229 | 1.04 | 0.86 | -0.95 | 2.97 |
| 23 | D09 | 5229 | PGGT1B | NM_005023 | 237435 | 251147 | 0.99 | 1.00 | 0.05 | -2.47 |
| 23 | D10 | 5257 | PHKB | NM_000293 | 228942 | 253279 | 0.95 | 1.01 | 1.01 | -1.79 |
| 23 | D11 | 162466 | PHOSPHO1 | NM_178500 | 211546 | 218332 | 0.88 | 0.87 | 1.59 | 2.06 |
| 23 | D12 | 29085 | PHPT1 | NM_014172 | 235157 | 234559 | 0.98 | 0.93 | -0.03 | 0.50 |
| 23 | E01 | NA | neg | NA | 241516 | 301811 | 1.00 | 1.20 | -1.73 | -9.26 |
| 23 | E02 | NA | neg | NA | 237699 | 244832 | 0.99 | 0.98 | -1.29 | -2.20 |
| 23 | E03 | 5277 | PIGA | NM_002641 | 221480 | 218052 | 0.92 | 0.87 | 0.48 | 2.04 |
| 23 | E04 | 5289 | PIK3C3 | NM_002647 | 240448 | 222407 | 1.00 | 0.89 | -0.88 | 0.30 |
| 23 | E05 | 5293 | PIK3CD | NM_005026 | 223059 | 212260 | 0.93 | 0.85 | 0.42 | 1.54 |
| 23 | E06 | 8395 | PIP5K1B | NM_003558 | 220146 | 221967 | 0.91 | 0.88 | 0.36 | 1.02 |
| 23 | E07 | 23396 | PIP5K1C | NM_012398 | 231510 | 244830 | 0.96 | 0.98 | -0.66 | -2.14 |
| 23 | E08 | 200576 | PIP5K3 | NM_015040 | 225799 | 239224 | 0.94 | 0.95 | 0.89 | -1.34 |
| 23 | E09 | 5321 | PLA2G4A | NM_024420 | 234556 | 205944 | 0.97 | 0.82 | -0.63 | 1.69 |
| 23 | E10 | 5327 | PLAT | NM_000930 | 231884 | 229565 | 0.96 | 0.91 | -0.32 | -0.30 |
| 23 | E11 | 5328 | PLAU | NM_002658 | 212388 | 228361 | 0.88 | 0.91 | 0.49 | -0.63 |
| 23 | E12 | 5331 | PLCB3 | NM_000932 | 243903 | 238971 | 1.01 | 0.95 | -2.02 | -1.49 |
| 23 | F01 | NA | NA | NA | 248137 | 312033 | 1.03 | 1.24 | -2.14 | -9.29 |
| 23 | F02 | NA | pos | NA | 142723 | 149301 | 0.59 | 0.60 | 9.82 | 10.89 |
| 23 | F03 | 5332 | PLCB4 | NM_000933 | 233981 | 250310 | 0.97 | 1.00 | -0.61 | -0.72 |
| 23 | F04 | 5337 | PLD1 | NM_002662 | 220570 | 243083 | 0.92 | 0.97 | 1.72 | -1.03 |
| 23 | F05 | 5351 | PLOD | NM_000302 | 245505 | 229947 | 1.02 | 0.92 | -1.79 | 0.59 |
| 23 | F06 | 5373 | PMM2 | NM_000303 | 215105 | 246125 | 0.89 | 0.98 | 1.27 | -0.74 |
| 23 | F07 | 5406 | PNLIP | NM_000936 | 227399 | 237249 | 0.95 | 0.95 | 0.14 | 0.04 |
| 23 | F08 | 5407 | PNLIPRP1 | NM_006229 | 241171 | 240681 | 1.00 | 0.96 | -0.52 | -0.28 |
| 23 | F09 | 5409 | PNMT | NM_002686 | 232422 | 217388 | 0.97 | 0.87 | -0.05 | 1.51 |
| 23 | F10 | 5433 | POLR2D | NM_004805 | 231177 | 237380 | 0.96 | 0.95 | 0.09 | -0.03 |
| 23 | F11 | 10585 | POMT1 | NM_007171 | 222473 | 230923 | 0.92 | 0.92 | -0.32 | 0.29 |
| 23 | F12 | 5444 | PON1 | NM_000446 | 228020 | 231920 | 0.95 | 0.92 | 0.12 | 0.62 |
| 23 | G01 | NA | neg | NA | 241532 | 253231 | 1.00 | 1.01 | -1.33 | -2.90 |
| 23 | G02 | NA | neg | NA | 232398 | 232718 | 0.97 | 0.93 | -0.29 | -0.36 |
| 23 | G03 | 5445 | PON2 | NM_000305 | 230272 | 232742 | 0.96 | 0.93 | -0.12 | 0.55 |
| 23 | G04 | 5446 | PON3 | NM_000940 | 245696 | 223623 | 1.02 | 0.89 | -1.07 | 0.48 |
| 23 | G05 | 5447 | POR | NM_000941 | 223780 | 227648 | 0.93 | 0.91 | 0.74 | -0.03 |
| 23 | G06 | 27068 | PPA2 | NM_006903 | 246412 | 236810 | 1.02 | 0.94 | -2.22 | -0.49 |
| 23 | G07 | 8611 | PPAP2A | NM_003711 | 229201 | 235545 | 0.95 | 0.94 | 0.00 | -0.65 |
| 23 | G08 | 51645 | PPIL1 | NM_016059 | 220595 | 231062 | 0.92 | 0.92 | 1.88 | 0.01 |

| | | | | | | | | | | |
|---|---|---|---|---|---|---|---|---|---|---|
| 23 | G09 | 5498 | PPOX | NM_000309 | 224555 | 218612 | 0.93 | 0.87 | 0.91 | 0.46 |
| 23 | G10 | 5538 | PPT1 | NM_000310 | 234675 | 217327 | 0.98 | 0.87 | -0.24 | 1.56 |
| 23 | G11 | 5547 | PRCP | NM_005040 | 218793 | 233261 | 0.91 | 0.93 | 0.16 | -0.90 |
| 23 | G12 | 5557 | PRIM1 | NM_000946 | 229578 | 238622 | 0.95 | 0.95 | 0.00 | -1.11 |
| 23 | H01 | NA | NA | NA | 276315 | 281972 | 1.15 | 1.12 | -4.82 | -5.14 |
| 23 | H02 | NA | pos | NA | 136746 | 177082 | 0.57 | 0.71 | 11.02 | 7.87 |
| 23 | H03 | 5625 | PRODH | NM_016335 | 249828 | 247421 | 1.04 | 0.99 | -1.88 | 0.06 |
| 23 | H04 | 58510 | PRODH2 | NM_021232 | 244463 | 251516 | 1.02 | 1.00 | -0.47 | -1.65 |
| 23 | H05 | 10279 | PRSS16 | NM_005865 | 238030 | 248472 | 0.99 | 0.99 | -0.42 | -1.29 |
| 23 | H06 | 5651 | PRSS7 | NM_002772 | 244402 | 236626 | 1.02 | 0.94 | -1.53 | 0.86 |
| 23 | H07 | 219595 | PSMAL/GCP III | NM_153696 | 231727 | 241799 | 0.96 | 0.96 | 0.17 | -0.10 |
| 23 | H08 | 5699 | PSMB10 | NM_002801 | 238430 | 241959 | 0.99 | 0.96 | 0.32 | -0.01 |
| 23 | H09 | 5692 | PSMB4 | NM_002796 | 234638 | 236666 | 0.98 | 0.94 | 0.22 | -0.46 |
| 23 | H10 | 5694 | PSMB6 | NM_002798 | 234810 | 240336 | 0.98 | 0.96 | 0.20 | 0.03 |
| 23 | H11 | 5698 | PSMB9 | NM_002800 | 225671 | 233527 | 0.94 | 0.93 | -0.16 | 0.39 |
| 23 | H12 | 9791 | PTDSS1 | NM_014754 | 224371 | 233031 | 0.93 | 0.93 | 1.05 | 0.90 |
| 24 | A01 | NA | pos | NA | 130141 | 123344 | 0.52 | 0.50 | 8.92 | 17.20 |
| 24 | A02 | NA | NA | NA | 304717 | 239303 | 1.22 | 0.97 | -6.12 | 2.48 |
| 24 | A03 | 5740 | PTGIS | NM_000961 | 235482 | 289169 | 0.95 | 1.17 | 0.58 | -3.11 |
| 24 | A04 | 65263 | PYCRL | NM_023078 | 241393 | 303194 | 0.97 | 1.23 | 0.03 | -5.06 |
| 24 | A05 | 5860 | QDPR | NM_000320 | 229384 | 302636 | 0.92 | 1.23 | 1.50 | -4.80 |
| 24 | A06 | 5875 | RABGGTA | NM_004581 | 234956 | 302099 | 0.94 | 1.22 | -0.11 | -5.36 |
| 24 | A07 | 5876 | RABGGTB | NM_004582 | 244255 | 243182 | 0.98 | 0.98 | -2.00 | 1.89 |
| 24 | A08 | 9986 | RCE1 | NM_005133 | 286326 | 250303 | 1.15 | 1.01 | -4.23 | 0.36 |
| 24 | A09 | 5959 | RDH5 | NM_002905 | 225397 | 243748 | 0.91 | 0.99 | 0.20 | 1.25 |
| 24 | A10 | 50700 | RDH8 | NM_015725 | 234757 | 263108 | 0.94 | 1.07 | -0.33 | -0.47 |
| 24 | A11 | 5972 | REN | NM_000537 | 231418 | 232453 | 0.93 | 0.94 | -0.02 | 1.92 |
| 24 | A12 | 10248 | POP7 | NM_005837 | 226265 | 231049 | 0.91 | 0.94 | 0.65 | 3.11 |
| 24 | B01 | NA | neg | NA | 250310 | 264834 | 1.01 | 1.07 | -2.18 | -4.66 |
| 24 | B02 | NA | neg | NA | 252240 | 263526 | 1.01 | 1.07 | -2.34 | -4.49 |
| 24 | B03 | 6240 | RRM1 | NM_001033 | 214945 | 227841 | 0.86 | 0.92 | 1.61 | 0.78 |
| 24 | B04 | 6241 | RRM2 | NM_001034 | 177710 | 219129 | 0.71 | 0.89 | 4.78 | 1.72 |
| 24 | B05 | 50484 | RRM2B | NM_015713 | 253527 | 240270 | 1.02 | 0.97 | -1.33 | -0.78 |
| 24 | B06 | 55811 | SAC | NM_018417 | 206912 | 224065 | 0.83 | 0.91 | 1.56 | 0.65 |
| 24 | B07 | 6296 | SAH | NM_005622 | 212279 | 221868 | 0.85 | 0.90 | 0.01 | 0.70 |
| 24 | B08 | 1757 | SARDH | NM_007101 | 231583 | 227565 | 0.93 | 0.92 | -0.26 | -0.65 |
| 24 | B09 | 6303 | SAT | NM_002970 | 240928 | 226606 | 0.97 | 0.92 | -1.88 | -0.47 |
| 24 | B10 | 6307 | SC4MOL | NM_006745 | 244157 | 231787 | 0.98 | 0.94 | -1.88 | -0.39 |
| 24 | B11 | 6391 | SDHC | NM_003001 | 222661 | 224374 | 0.89 | 0.91 | -0.01 | -0.95 |
| 24 | B12 | 113675 | SDSL | NM_138432 | 207600 | 221892 | 0.83 | 0.90 | 1.52 | 0.37 |
| 24 | C01 | NA | pos | NA | 108257 | 97572 | 0.44 | 0.40 | 9.58 | 16.43 |
| 24 | C02 | NA | NA | NA | 250815 | 245077 | 1.01 | 0.99 | -2.70 | -2.29 |
| 24 | C03 | 29843 | SENP1 | NM_014554 | 225732 | 233878 | 0.91 | 0.95 | 0.20 | -0.12 |
| 24 | C04 | 80854 | SET7 | NM_030648 | 239594 | 231035 | 0.96 | 0.94 | -1.04 | 0.07 |
| 24 | C05 | 387893 | SET8 | NM_020382 | 203133 | 228281 | 0.82 | 0.92 | 2.53 | 0.60 |
| 24 | C06 | 81537 | SGPP1 | NM_030791 | 212687 | 213269 | 0.85 | 0.86 | 0.58 | 1.88 |
| 24 | C07 | 6448 | SGSH | NM_000199 | 191596 | 231071 | 0.77 | 0.94 | 1.31 | -0.61 |
| 24 | C08 | 23677 | SH3BP4 | NM_014521 | 209697 | 204911 | 0.84 | 0.83 | 1.15 | 2.09 |
| 24 | C09 | 6469 | SHH | NM_000193 | 228697 | 220129 | 0.92 | 0.89 | -1.31 | 0.21 |
| 24 | C10 | 6470 | SHMT1 | NM_004169 | 233046 | 250204 | 0.94 | 1.01 | -1.41 | -2.86 |
| 24 | C11 | 6472 | SHMT2 | NM_005412 | 219046 | 216409 | 0.88 | 0.88 | -0.18 | -0.07 |
| 24 | C12 | 6480 | SIAT1 | NM_003032 | 233799 | 238027 | 0.94 | 0.96 | -1.22 | -1.81 |
| 24 | D01 | NA | neg | NA | 250612 | 294306 | 1.01 | 1.19 | -2.26 | -7.70 |
| 24 | D02 | NA | neg | NA | 247298 | 249602 | 0.99 | 1.01 | -1.98 | -2.02 |
| 24 | D03 | 6482 | SIAT4A | NM_003033 | 239605 | 254168 | 0.96 | 1.03 | -0.57 | -1.86 |
| 24 | D04 | 8869 | SIAT9 | NM_003896 | 234923 | 238747 | 0.94 | 0.97 | -0.21 | -0.07 |
| 24 | D05 | 10419 | SKB1 | NM_006109 | 237150 | 232341 | 0.95 | 0.94 | 0.03 | 0.93 |
| 24 | D06 | 11001 | SLC27A2 | NM_003645 | 224502 | 234124 | 0.90 | 0.95 | -0.01 | 0.08 |
| 24 | D07 | 6519 | SLC3A1 | NM_000341 | 207401 | 235511 | 0.83 | 0.95 | 0.37 | -0.33 |
| 24 | D08 | 11039 | SMA4 | NM_021652 | 224372 | 238258 | 0.90 | 0.97 | 0.31 | -1.30 |
| 24 | D09 | 6609 | SMPD1 | NM_000543 | 208405 | 246440 | 0.84 | 1.00 | 0.86 | -2.29 |
| 24 | D10 | 55512 | SMPD3 | NM_018667 | 215338 | 226788 | 0.87 | 0.92 | 0.54 | 0.95 |
| 24 | D11 | 8435 | SOAT2 | NM_003578 | 227494 | 219252 | 0.91 | 0.89 | -0.48 | 0.41 |
| 24 | D12 | 6697 | SPR | NM_003124 | 225066 | 228921 | 0.90 | 0.93 | -0.05 | 0.19 |
| 24 | E01 | NA | neg | NA | 245765 | 243293 | 0.99 | 0.99 | -1.86 | -0.61 |
| 24 | E02 | NA | neg | NA | 244998 | 226686 | 0.98 | 0.92 | -1.79 | 1.50 |
| 24 | E03 | 10558 | SPTLC1 | NM_006415 | 229809 | 242597 | 0.92 | 0.98 | 0.25 | 0.22 |
| 24 | E04 | 6713 | SQLE | NM_003129 | 232693 | 231724 | 0.94 | 0.94 | -0.03 | 1.44 |
| 24 | E05 | 6715 | SRD5A1 | NM_001047 | 237592 | 242788 | 0.95 | 0.98 | -0.03 | 0.22 |
| 24 | E06 | 6716 | SRD5A2 | NM_000348 | 241572 | 214272 | 0.97 | 0.87 | -1.50 | 3.21 |
| 24 | E07 | 6723 | SRM | NM_003132 | 205134 | 245517 | 0.82 | 0.99 | 0.55 | -0.99 |
| 24 | E08 | 6768 | ST14 | NM_021978 | 228982 | 247331 | 0.92 | 1.00 | -0.11 | -1.84 |
| 24 | E09 | 6764 | ST5 | NM_005418 | 223143 | 251328 | 0.90 | 1.02 | -0.42 | -2.30 |
| 24 | E10 | 55959 | SULF2 | NM_018837 | 217661 | 227995 | 0.87 | 0.92 | 0.33 | 1.41 |
| 24 | E11 | 6817 | SULT1A1 | NM_001055 | 218147 | 232318 | 0.88 | 0.94 | 0.30 | -0.64 |
| 24 | E12 | 6834 | SURF1 | NM_003172 | 223831 | 236686 | 0.90 | 0.96 | 0.05 | -0.19 |
| 24 | F01 | NA | NA | NA | 262174 | 293712 | 1.05 | 1.19 | -3.15 | -7.08 |
| 24 | F02 | NA | pos | NA | 150207 | 146758 | 0.60 | 0.59 | 6.49 | 11.57 |
| 24 | F03 | 8871 | SYNJ2 | NM_003898 | 236450 | 242776 | 0.95 | 0.98 | -0.20 | 0.12 |
| 24 | F04 | 6888 | TALDO1 | NM_006755 | 230059 | 245449 | 0.92 | 0.99 | 0.32 | -0.38 |
| 24 | F05 | 6901 | TAZ | NM_000116 | 217306 | 238445 | 0.87 | 0.97 | 1.84 | 0.69 |
| 24 | F06 | 6916 | TBXAS1 | NM_001061 | 223037 | 248109 | 0.90 | 1.00 | 0.22 | -1.16 |
| 24 | F07 | 55775 | TDP1 | NM_018319 | 236130 | 237886 | 0.95 | 0.96 | -2.00 | -0.09 |
| 24 | F08 | 51106 | TFB1M | NM_016020 | 241498 | 225377 | 0.97 | 0.91 | -1.06 | 0.87 |
| 24 | F09 | 64216 | TFB2M | NM_022366 | 207393 | 197874 | 0.83 | 0.80 | 1.06 | 4.41 |
| 24 | F10 | 7036 | TFR2 | NM_003227 | 299111 | 244590 | 1.20 | 0.99 | -6.57 | -0.77 |
| 24 | F11 | 7037 | TFRC | NM_003234 | 206559 | 213092 | 0.83 | 0.86 | 1.42 | 1.73 |
| 24 | F12 | 26027 | THEA | NM_015547 | 228967 | 245111 | 0.92 | 0.99 | -0.28 | -1.33 |

| | | | | | | | | | | |
|---|---|---|---|---|---|---|---|---|---|---|
| 24 | G01 | NA | neg | NA | 256738 | 244182 | 1.03 | 0.99 | -2.82 | -1.31 |
| 24 | G02 | NA | neg | NA | 235186 | 241277 | 0.95 | 0.98 | -0.97 | -0.94 |
| 24 | G03 | 7108 | TM7SF2 | NM_003273 | 242189 | 248285 | 0.97 | 1.01 | -0.83 | -1.09 |
| 24 | G04 | 64699 | TMPRSS3 | NM_024022 | 231261 | 232644 | 0.93 | 0.94 | 0.07 | 0.73 |
| 24 | G05 | 7167 | TPI1 | NM_000365 | 243403 | 246758 | 0.98 | 1.00 | -0.55 | -0.87 |
| 24 | G06 | 7172 | TPMT | NM_000367 | 223780 | 235549 | 0.90 | 0.95 | 0.01 | -0.08 |
| 24 | G07 | 8295 | TRRAP | NM_003496 | 211423 | 232364 | 0.85 | 0.94 | -0.01 | 0.09 |
| 24 | G08 | 81567 | TXNDC5 | NM_022085 | 226323 | 224967 | 0.91 | 0.91 | 0.11 | 0.41 |
| 24 | G09 | 7299 | TYR | NM_000372 | 220377 | 226594 | 0.89 | 0.92 | -0.20 | 0.26 |
| 24 | G10 | 7306 | TYRP1 | NM_000550 | 204749 | 230176 | 0.82 | 0.93 | 1.42 | 0.55 |
| 24 | G11 | 53347 | UBASH3A | NM_018961 | 221374 | 227625 | 0.89 | 0.92 | 0.01 | -0.63 |
| 24 | G12 | 7317 | UBE1 | NM_003334 | 229907 | 238907 | 0.92 | 0.97 | -0.50 | -1.06 |
| 24 | H01 | NA | NA | NA | 285901 | 271145 | 1.15 | 1.10 | -4.11 | -4.63 |
| 24 | H02 | NA | pos | NA | 135617 | 140501 | 0.55 | 0.57 | 8.84 | 11.95 |
| 24 | H03 | 9039 | UBE1C | NM_003968 | 292600 | 233598 | 1.18 | 0.95 | -3.95 | 0.88 |
| 24 | H04 | 7319 | UBE2A | NM_003336 | 312010 | 258558 | 1.25 | 1.05 | -5.66 | -2.46 |
| 24 | H05 | 7320 | UBE2B | NM_003337 | 264596 | 242385 | 1.06 | 0.98 | -1.15 | -0.22 |
| 24 | H06 | 11065 | UBE2C | NM_007019 | 242233 | 236372 | 0.97 | 0.96 | -0.35 | -0.08 |
| 24 | H07 | 7322 | UBE2D2 | NM_003339 | 235917 | 228625 | 0.95 | 0.93 | -0.90 | 0.67 |
| 24 | H08 | 7323 | UBE2D3 | NM_003340 | 237537 | 231845 | 0.95 | 0.94 | 0.36 | -0.36 |
| 24 | H09 | 7324 | UBE2E1 | NM_003341 | 223174 | 231060 | 0.90 | 0.94 | 0.78 | -0.21 |
| 24 | H10 | 10477 | UBE2E3 | NM_006357 | 220796 | 232222 | 0.89 | 0.94 | 1.26 | 0.39 |
| 24 | H11 | 7327 | UBE2G2 | NM_003343 | 224410 | 222883 | 0.90 | 0.90 | 0.97 | 0.07 |
| 24 | H12 | 7328 | UBE2H | NM_003344 | 225354 | 225034 | 0.91 | 0.91 | 1.12 | 0.81 |
| 25 | A01 | NA | pos | NA | 117173 | 118181 | 0.46 | 0.45 | 12.46 | 17.41 |
| 25 | A02 | NA | NA | NA | 238891 | 288031 | 0.94 | 1.09 | -1.99 | -5.00 |
| 25 | A03 | 7329 | UBE2I | NM_003345 | 228202 | 303152 | 0.90 | 1.15 | 0.03 | -5.63 |
| 25 | A04 | 51465 | UBE2J1 | NM_016021 | 230901 | 259255 | 0.91 | 0.98 | -0.08 | 0.21 |
| 25 | A05 | 118424 | UBE2J2 | NM_058167 | 276377 | 254705 | 1.09 | 0.97 | -5.20 | 0.10 |
| 25 | A06 | 55585 | UBE2Q | NM_017582 | 231529 | 279625 | 0.91 | 1.06 | -0.02 | -2.79 |
| 25 | A07 | 54926 | UBE2R2 | NM_017811 | 233420 | 242592 | 0.92 | 0.92 | -2.59 | 1.09 |
| 25 | A08 | 7336 | UBE2V2 | NM_003350 | 237501 | 247270 | 0.94 | 0.94 | -1.71 | 0.15 |
| 25 | A09 | 8266 | UBL4 | NM_014235 | 220194 | 246043 | 0.87 | 0.93 | 0.05 | -0.07 |
| 25 | A10 | 55293 | UEV3 | NM_018314 | 207067 | 251622 | 0.82 | 0.95 | 1.27 | -0.32 |
| 25 | A11 | 7353 | UFD1L | NM_005659 | 211205 | 303730 | 0.83 | 1.15 | 0.95 | -7.53 |
| 25 | A12 | 7357 | UGCG | NM_003358 | 220809 | 241905 | 0.87 | 0.92 | 0.05 | 0.98 |
| 25 | B01 | NA | neg | NA | 303306 | 295279 | 1.20 | 1.12 | -9.20 | -8.82 |
| 25 | B02 | NA | neg | NA | 266284 | 244205 | 1.05 | 0.93 | -4.80 | -2.08 |
| 25 | B03 | 54659 | UGT1A3 | NM_019093 | 229836 | 227995 | 0.91 | 0.87 | 0.28 | 1.43 |
| 25 | B04 | 54657 | UGT1A4 | NM_007120 | 232714 | 246690 | 0.92 | 0.94 | 0.16 | -0.99 |
| 25 | B05 | 54578 | UGT1A6 | NM_001072 | 232918 | 244172 | 0.92 | 0.93 | 0.41 | -1.37 |
| 25 | B06 | 54576 | UGT1A8 | NM_019076 | 236295 | 231471 | 0.93 | 0.88 | -0.14 | 0.71 |
| 25 | B07 | 54600 | UGT1A9 | NM_021027 | 211894 | 216356 | 0.84 | 0.82 | 0.42 | 1.69 |
| 25 | B08 | 7366 | UGT2B15 | NM_001076 | 240076 | 228820 | 0.95 | 0.87 | -1.57 | -0.27 |
| 25 | B09 | 7363 | UGT2B4 | NM_021139 | 226992 | 231170 | 0.89 | 0.88 | -0.31 | -0.96 |
| 25 | B10 | 7368 | UGT8 | NM_003360 | 245316 | 228950 | 0.97 | 0.87 | -2.83 | -0.19 |
| 25 | B11 | 7372 | UMPS | NM_000373 | 224329 | 223532 | 0.88 | 0.85 | -0.16 | 0.19 |
| 25 | B12 | 7374 | UNG | NM_003362 | 222826 | 221762 | 0.88 | 0.84 | 0.26 | 0.78 |
| 25 | C01 | NA | pos | NA | 112808 | 140065 | 0.44 | 0.53 | 12.50 | 11.66 |
| 25 | C02 | NA | NA | NA | 254084 | 244047 | 1.00 | 0.93 | -4.28 | -2.06 |
| 25 | C03 | 10309 | UNG2 | NM_021147 | 236440 | 247789 | 0.93 | 0.94 | -1.44 | -1.19 |
| 25 | C04 | 7378 | UPP1 | NM_003364 | 213479 | 231749 | 0.84 | 0.88 | 1.51 | 0.98 |
| 25 | C05 | 10975 | UQCR | NM_006830 | 228416 | 221768 | 0.90 | 0.84 | 0.01 | 1.59 |
| 25 | C06 | 7384 | UQCRC1 | NM_003365 | 213177 | 223853 | 0.84 | 0.85 | 1.67 | 1.72 |
| 25 | C07 | 7385 | UQCRC2 | NM_003366 | 200239 | 231279 | 0.79 | 0.88 | 0.87 | -0.28 |
| 25 | C08 | 7386 | UQCRFS1 | NM_006003 | 216175 | 213864 | 0.85 | 0.81 | 0.33 | 1.70 |
| 25 | C09 | 7388 | UQCRH | NM_006004 | 216718 | 230122 | 0.85 | 0.87 | -0.02 | -0.82 |
| 25 | C10 | 7389 | UROD | NM_000374 | 225637 | 225412 | 0.89 | 0.86 | -1.42 | 0.28 |
| 25 | C11 | 7390 | UROS | NM_000375 | 218793 | 243795 | 0.86 | 0.93 | -0.44 | -2.48 |
| 25 | C12 | 8876 | VNN1 | NM_004666 | 236073 | 250420 | 0.93 | 0.95 | -2.25 | -3.00 |
| 25 | D01 | NA | neg | NA | 258472 | 293768 | 1.02 | 1.11 | -3.87 | -8.20 |
| 25 | D02 | NA | neg | NA | 251231 | 282848 | 0.99 | 1.07 | -3.01 | -6.76 |
| 25 | D03 | 8875 | VNN2 | NM_004665 | 253885 | 249627 | 1.00 | 0.95 | -2.57 | -1.02 |
| 25 | D04 | 83451 | WBSCR21 | NM_031295 | 233428 | 245013 | 0.92 | 0.93 | 0.08 | -0.35 |
| 25 | D05 | 7511 | XPNPEP1 | NM_020383 | 249839 | 232826 | 0.98 | 0.88 | -1.60 | 0.54 |
| 25 | D06 | 10269 | ZMPSTE24 | NM_005857 | 238822 | 245461 | 0.94 | 0.93 | -0.43 | -0.72 |
| 25 | D07 | 28 | ABO | NM_020469 | 204502 | 230227 | 0.81 | 0.87 | 1.30 | 0.28 |
| 25 | D08 | 26 | ABP1 | NM_001091 | 193755 | 229959 | 0.76 | 0.87 | 3.93 | -0.01 |
| 25 | D09 | 31 | ACACA | NM_198834 | 245355 | 236564 | 0.97 | 0.90 | -2.48 | -1.26 |
| 25 | D10 | 32 | ACACB | NM_001093 | 205724 | 200930 | 0.81 | 0.76 | 1.88 | 3.92 |
| 25 | D11 | 55902 | ACAS2 | NM_018677 | 221833 | 228048 | 0.87 | 0.87 | 0.14 | 0.01 |
| 25 | D12 | 1636 | ACE | NM_000789 | 225497 | 229952 | 0.89 | 0.87 | -0.05 | 0.12 |
| 25 | E01 | NA | neg | NA | 241065 | 242090 | 0.95 | 0.92 | -2.39 | -1.95 |
| 25 | E02 | NA | neg | NA | 243580 | 232080 | 0.96 | 0.88 | -2.69 | -0.63 |
| 25 | E03 | 43 | ACHE | NM_000665 | 211209 | 237940 | 0.83 | 0.90 | 1.91 | -0.04 |
| 25 | E04 | 22985 | ACIN1 | NM_014977 | 236600 | 237366 | 0.93 | 0.90 | -0.89 | 0.09 |
| 25 | E05 | 8751 | ADAM15 | NM_003815 | 232641 | 237182 | 0.92 | 0.90 | -0.14 | -0.60 |
| 25 | E06 | 6868 | ADAM17 | NM_003183 | 218203 | 231349 | 0.86 | 0.88 | 1.42 | 0.57 |
| 25 | E07 | 8748 | ADAM20 | NM_003814 | 201731 | 239026 | 0.80 | 0.91 | 1.04 | -1.45 |
| 25 | E08 | 8747 | ADAM21 | NM_003813 | 233593 | 204534 | 0.92 | 0.78 | -1.39 | 2.78 |
| 25 | E09 | 10863 | ADAM28 | NM_014265 | 218344 | 206078 | 0.86 | 0.78 | 0.13 | 2.19 |
| 25 | E10 | 11085 | ADAM30 | NM_021794 | 199572 | 230866 | 0.79 | 0.88 | 2.02 | -0.60 |
| 25 | E11 | 81794 | ADAMTS10 | NM_030957 | 288453 | 216048 | 1.14 | 0.82 | -8.36 | 1.02 |
| 25 | E12 | 170689 | ADAMTS15 | NM_139055 | 221809 | 232505 | 0.87 | 0.88 | -0.20 | -0.79 |
| 25 | F01 | NA | NA | NA | 254302 | 265920 | 1.00 | 1.01 | -3.22 | -4.27 |
| 25 | F02 | NA | pos | NA | 163491 | 166875 | 0.64 | 0.63 | 7.57 | 8.79 |
| 25 | F03 | 170691 | ADAMTS17 | NM_139057 | 247846 | 229116 | 0.98 | 0.87 | -1.70 | 1.95 |
| 25 | F04 | 171019 | ADAMTS19 | NM_133638 | 242893 | 244906 | 0.96 | 0.93 | -0.89 | -0.09 |

| | | | | | | | | | | |
|---|---|---|---|---|---|---|---|---|---|---|
| 25 | F05 | 80070 | ADAMTS20 | NM_025003 | 217196 | 236650 | 0.86 | 0.90 | 2.44 | 0.29 |
| 25 | F06 | 9508 | ADAMTS3 | NM_014243 | 236306 | 248169 | 0.93 | 0.94 | 0.02 | -0.82 |
| 25 | F07 | 9507 | ADAMTS4 | NM_005099 | 240076 | 243405 | 0.95 | 0.92 | -2.77 | -1.21 |
| 25 | F08 | 11174 | ADAMTS6 | NM_014273 | 227960 | 240328 | 0.90 | 0.91 | 0.03 | -1.12 |
| 25 | F09 | 11173 | ADAMTS7 | NM_014272 | 225421 | 225164 | 0.89 | 0.85 | 0.04 | 0.50 |
| 25 | F10 | 56999 | ADAMTS9 | NM_020249 | 240070 | 231171 | 0.95 | 0.88 | -2.04 | 0.19 |
| 25 | F11 | 124 | ADH1A | NM_000667 | 224397 | 233491 | 0.88 | 0.89 | -0.01 | -0.45 |
| 25 | F12 | 126 | ADH1C | NM_000669 | 217247 | 231952 | 0.86 | 0.88 | 1.08 | 0.11 |
| 25 | G01 | NA | neg | NA | 256135 | 296065 | 1.01 | 1.12 | -2.74 | -7.92 |
| 25 | G02 | NA | neg | NA | 240089 | 236892 | 0.95 | 0.90 | -0.83 | -0.11 |
| 25 | G03 | 141 | ADPRH | NM_001125 | 239099 | 246038 | 0.94 | 0.93 | 0.04 | 0.04 |
| 25 | G04 | 10038 | ADPRTL2 | NM_005484 | 239854 | 238087 | 0.95 | 0.90 | 0.17 | 1.15 |
| 25 | G05 | 159 | ADSS | NM_001126 | 243676 | 242118 | 0.96 | 0.92 | -0.01 | -0.10 |
| 25 | G06 | 172 | AFG3L1 | NM_001132 | 227261 | 244538 | 0.90 | 0.93 | 1.79 | -0.01 |
| 25 | G07 | 57379 | AICDA | NM_020661 | 226168 | 240856 | 0.89 | 0.91 | -0.42 | -0.54 |
| 25 | G08 | 80709 | AKNA | NM_030767 | 233568 | 234256 | 0.92 | 0.89 | 0.06 | 0.01 |
| 25 | G09 | 59344 | ALOXE3 | NM_021628 | 234865 | 230944 | 0.93 | 0.88 | -0.38 | 0.07 |
| 25 | G10 | 249 | ALPL | NM_000478 | 229429 | 232655 | 0.90 | 0.88 | -0.08 | 0.32 |
| 25 | G11 | 251 | ALPPL2 | NM_031313 | 230131 | 232638 | 0.91 | 0.88 | 0.01 | -0.01 |
| 25 | G12 | 64682 | ANAPC1 | NM_022662 | 232994 | 243974 | 0.92 | 0.93 | -0.09 | -1.15 |
| 25 | H01 | NA | NA | NA | 250473 | 296171 | 0.99 | 1.12 | -2.76 | -7.43 |
| 25 | H02 | NA | pos | NA | 134378 | 163868 | 0.53 | 0.62 | 11.03 | 10.03 |
| 25 | H03 | 51433 | ANAPC5 | NM_016237 | 233819 | 238762 | 0.92 | 0.91 | -0.03 | 1.51 |
| 25 | H04 | 283 | ANG | NM_001145 | 240454 | 262298 | 0.95 | 1.00 | -0.60 | -1.55 |
| 25 | H05 | 10930 | APOBEC2 | NM_006789 | 232022 | 307732 | 0.91 | 1.17 | 0.68 | -8.25 |
| 25 | H06 | 200315 | APOBEC3A | NM_145699 | 240264 | 248141 | 0.95 | 0.94 | -0.45 | 0.01 |
| 25 | H07 | 27350 | APOBEC3C | NM_014508 | 228758 | 234578 | 0.90 | 0.89 | -1.42 | 0.79 |
| 25 | H08 | 60489 | APOBEC3G | NM_021822 | 228410 | 238264 | 0.90 | 0.90 | -0.03 | -0.02 |
| 25 | H09 | 379 | ARF4L | NM_001661 | 225581 | 231356 | 0.89 | 0.88 | 0.02 | 0.52 |
| 25 | H10 | 10139 | ARFRP1 | NM_003224 | 222199 | 252553 | 0.88 | 0.96 | 0.08 | -1.80 |
| 25 | H11 | 383 | ARG1 | NM_000045 | 216269 | 223322 | 0.85 | 0.85 | 0.96 | 1.72 |
| 25 | H12 | 388 | RHOB | NM_004040 | 223112 | 239893 | 0.88 | 0.91 | 0.39 | -0.11 |
| 26 | A01 | NA | pos | NA | 158937 | 212609 | 0.64 | 0.86 | 7.87 | 3.45 |
| 26 | A02 | NA | NA | NA | 290095 | 309814 | 1.17 | 1.25 | -4.51 | -11.89 |
| 26 | A03 | 29984 | RHOD | NM_014578 | 306076 | 306524 | 1.23 | 1.24 | -4.96 | -11.07 |
| 26 | A04 | 54509 | RHOF | NM_019034 | 296528 | 271000 | 1.19 | 1.09 | -4.56 | -4.30 |
| 26 | A05 | 391 | RHOG | NM_001665 | 305182 | 292999 | 1.23 | 1.18 | -5.01 | -8.56 |
| 26 | A06 | 9077 | ARHI | NM_004675 | 242453 | 231698 | 0.98 | 0.94 | -0.04 | 1.27 |
| 26 | A07 | 57381 | RHOJ | NM_020663 | 232473 | 229549 | 0.93 | 0.93 | 1.07 | 0.05 |
| 26 | A08 | 8153 | ARHN | NM_005440 | 234656 | 235100 | 0.94 | 0.95 | 0.17 | -0.03 |
| 26 | A09 | 23433 | RHOQ | NM_012249 | 236621 | 226925 | 0.95 | 0.92 | -0.27 | 1.14 |
| 26 | A10 | 10124 | ARL4A | NM_005738 | 232999 | 222938 | 0.94 | 0.90 | 0.04 | 1.43 |
| 26 | A11 | 410 | ARSA | NM_000487 | 229101 | 231620 | 0.92 | 0.94 | 0.67 | -0.93 |
| 26 | A12 | 411 | ARSB | NM_000046 | 210055 | 229874 | 0.84 | 0.93 | 3.03 | 0.66 |
| 26 | B01 | NA | neg | NA | 252317 | 249268 | 1.01 | 1.01 | -1.32 | -2.74 |
| 26 | B02 | NA | neg | NA | 248815 | 235265 | 1.00 | 0.95 | -0.99 | -0.53 |
| 26 | B03 | 445 | ASS | NM_000050 | 215808 | 225985 | 0.87 | 0.91 | 3.18 | 1.24 |
| 26 | B04 | 549 | AUH | NM_001698 | 256054 | 235474 | 1.03 | 0.95 | -1.12 | 0.90 |
| 26 | B05 | 11332 | BACH | NM_007274 | 289902 | 237037 | 1.17 | 0.96 | -3.95 | -0.13 |
| 26 | B06 | 10919 | BAT8 | NM_006709 | 229845 | 241491 | 0.92 | 0.98 | 0.77 | -0.67 |
| 26 | B07 | 590 | BCHE | NM_000055 | 227511 | 227250 | 0.91 | 0.92 | 1.16 | 0.01 |
| 26 | B08 | 4671 | BIRC1 | NM_004536 | 238646 | 231090 | 0.96 | 0.93 | -0.59 | 0.21 |
| 26 | B09 | 641 | BLM | NM_000057 | 250207 | 238362 | 1.01 | 0.96 | -1.93 | -1.07 |
| 26 | B10 | 83990 | BRIP1 | NM_032043 | 240639 | 229533 | 0.97 | 0.93 | -1.06 | -0.01 |
| 26 | B11 | 8045 | C11ORF13 | NM_003475 | 226196 | 224688 | 0.91 | 0.91 | 0.56 | -0.23 |
| 26 | B12 | 80267 | C1ORF22 | NM_025191 | 225756 | 231474 | 0.91 | 0.94 | 1.17 | 0.01 |
| 26 | C01 | NA | pos | NA | 138439 | 112073 | 0.56 | 0.45 | 8.49 | 19.03 |
| 26 | C02 | NA | NA | NA | 238146 | 242422 | 0.96 | 0.98 | -0.91 | -1.53 |
| 26 | C03 | 715 | C1R | NM_001733 | 238884 | 245649 | 0.96 | 0.99 | 0.07 | -1.73 |
| 26 | C04 | 716 | C1S | NM_001734 | 214049 | 241568 | 0.86 | 0.98 | 1.91 | 0.07 |
| 26 | C05 | 55741 | C20ORF31 | NM_018217 | 231369 | 225328 | 0.93 | 0.91 | 0.64 | 1.85 |
| 26 | C06 | 51750 | RTEL1 | NM_016434 | 244528 | 252034 | 0.98 | 1.02 | -1.54 | -2.21 |
| 26 | C07 | 771 | CA12 | NM_001218 | 242973 | 222585 | 0.98 | 0.90 | -1.23 | 0.87 |
| 26 | C08 | 768 | CA9 | NM_001216 | 205427 | 233406 | 0.83 | 0.94 | 1.62 | -0.03 |
| 26 | C09 | 823 | CAPN1 | NM_005186 | 218108 | 223829 | 0.88 | 0.90 | 0.16 | 1.35 |
| 26 | C10 | 824 | CAPN2 | NM_001748 | 219988 | 226069 | 0.88 | 0.91 | -0.04 | 0.67 |
| 26 | C11 | 826 | CAPNS1 | NM_001749 | 243928 | 241557 | 0.98 | 0.98 | -2.04 | -2.77 |
| 26 | C12 | 843 | CASP10 | NM_001230 | 243610 | 244778 | 0.98 | 0.99 | -1.44 | -1.96 |
| 26 | D01 | NA | neg | NA | 254184 | 289761 | 1.02 | 1.17 | -2.09 | -7.96 |
| 26 | D02 | NA | neg | NA | 242805 | 261452 | 0.98 | 1.06 | -1.02 | -3.50 |
| 26 | D03 | 835 | CASP2 | NM_001224 | 252256 | 230376 | 1.01 | 0.93 | -0.85 | 1.71 |
| 26 | D04 | 837 | CASP4 | NM_001225 | 236159 | 225494 | 0.95 | 0.91 | 0.16 | 3.64 |
| 26 | D05 | 838 | CASP5 | NM_004347 | 250102 | 249174 | 1.01 | 1.01 | -0.79 | -0.88 |
| 26 | D06 | 10694 | CCT8 | NM_006585 | 203784 | 232179 | 0.82 | 0.94 | 2.63 | 1.96 |
| 26 | D07 | 9425 | CDYL | NM_004824 | 234639 | 247622 | 0.94 | 1.00 | -0.11 | -2.04 |
| 26 | D08 | 1071 | CETP | NM_000078 | 206063 | 239458 | 0.83 | 0.97 | 1.89 | 0.05 |
| 26 | D09 | 51102 | CGI-63 | NM_016011 | 246846 | 240676 | 0.99 | 0.97 | -2.21 | -0.27 |
| 26 | D10 | 1106 | CHD2 | NM_001271 | 202728 | 241043 | 0.82 | 0.97 | 1.92 | -0.66 |
| 26 | D11 | 23507 | TA-LRRP | NM_015350 | 228068 | 229076 | 0.92 | 0.93 | -0.21 | 0.23 |
| 26 | D12 | 10883 | TAX1BP2 | U25801 | 231044 | 239095 | 0.93 | 0.97 | 0.08 | -0.03 |
| 26 | E01 | NA | neg | NA | 243481 | 245094 | 0.98 | 0.99 | -1.14 | -1.11 |
| 26 | E02 | NA | neg | NA | 239948 | 234455 | 0.97 | 0.95 | -0.81 | 0.57 |
| 26 | E03 | 6902 | TBCA | NM_004607 | 218155 | 237313 | 0.88 | 0.96 | 2.30 | 0.42 |
| 26 | E04 | 6903 | TBCC | NM_003192 | 239596 | 240937 | 0.96 | 0.97 | -0.23 | 1.01 |
| 26 | E05 | 6904 | TBCD | NM_005993 | 205474 | 237854 | 0.83 | 0.96 | 3.36 | 0.71 |
| 26 | E06 | 6905 | TBCE | NM_003193 | 250060 | 245427 | 1.01 | 0.99 | -1.79 | -0.32 |
| 26 | E07 | 6907 | TBL1X | NM_005647 | 249075 | 232032 | 1.00 | 0.94 | -1.53 | 0.23 |
| 26 | E08 | 6899 | TBX1 | NM_005992 | 227266 | 248310 | 0.91 | 1.00 | -0.17 | -1.54 |

| | | | | | | | | | | |
|---|---|---|---|---|---|---|---|---|---|---|
| 26 | E09 | 9096 | TBX18 | XM_496819 | 215201 | 238278 | 0.87 | 0.96 | 0.71 | -0.08 |
| 26 | E10 | 51224 | TCEB3B | NM_016427 | 214750 | 235619 | 0.86 | 0.95 | 0.73 | 0.01 |
| 26 | E11 | 8115 | TCL1A | NM_021966 | 236741 | 232306 | 0.95 | 0.94 | -1.09 | -0.46 |
| 26 | E12 | 9623 | TCL1B | NM_004918 | 229909 | 237734 | 0.92 | 0.96 | 0.13 | -0.01 |
| 26 | F01 | NA | NA | NA | 257392 | 308656 | 1.04 | 1.25 | -2.12 | -10.95 |
| 26 | F02 | NA | pos | NA | 161255 | 155296 | 0.65 | 0.63 | 6.95 | 13.25 |
| 26 | F03 | 6988 | TCTA | NM_022171 | 232475 | 241810 | 0.93 | 0.98 | 1.28 | -0.10 |
| 26 | F04 | 10955 | TDE1 | NM_006811 | 233145 | 270941 | 0.94 | 1.09 | 0.71 | -3.54 |
| 26 | F05 | 7004 | TEAD4 | NM_003213 | 245821 | 242764 | 0.99 | 0.98 | -0.12 | 0.13 |
| 26 | F06 | 7008 | TEF | NM_003216 | 246215 | 254171 | 0.99 | 1.03 | -1.09 | -1.51 |
| 26 | F07 | 7009 | TEGT | NM_003217 | 235173 | 253840 | 0.95 | 1.03 | 0.11 | -3.03 |
| 26 | F08 | 29844 | TFPT | NM_013342 | 213721 | 235456 | 0.86 | 0.95 | 1.44 | 0.67 |
| 26 | F09 | 7042 | TGFB2 | NM_003238 | 214120 | 224178 | 0.86 | 0.91 | 1.15 | 2.33 |
| 26 | F10 | 7059 | THBS3 | NM_007112 | 227360 | 240222 | 0.91 | 0.97 | -0.13 | -0.53 |
| 26 | F11 | 51298 | THEG | NM_016585 | 237410 | 221605 | 0.95 | 0.90 | -0.82 | 1.41 |
| 26 | F12 | 9984 | THOC1 | NM_005131 | 237159 | 234680 | 0.95 | 0.95 | -0.23 | 0.66 |
| 26 | G01 | NA | neg | NA | 258513 | 250111 | 1.04 | 1.01 | -2.60 | -1.71 |
| 26 | G02 | NA | neg | NA | 248482 | 245801 | 1.00 | 0.99 | -1.65 | -1.03 |
| 26 | G03 | 7066 | THPO | NM_000460 | 242933 | 247924 | 0.98 | 1.00 | -0.07 | -1.06 |
| 26 | G04 | 7069 | THRSP | NM_003251 | 238467 | 250191 | 0.96 | 1.01 | -0.16 | -0.26 |
| 26 | G05 | 7070 | THY1 | NM_006288 | 239452 | 250651 | 0.96 | 1.01 | 0.12 | -1.12 |
| 26 | G06 | 9220 | TIAF1 | NM_004740 | 229914 | 231537 | 0.92 | 0.94 | 0.07 | 2.06 |
| 26 | G07 | 26230 | TIAM2 | AF120323 | 234110 | 234704 | 0.94 | 0.95 | -0.16 | -0.01 |
| 26 | G08 | 23550 | TIC | NM_012455 | 228701 | 239564 | 0.92 | 0.97 | -0.34 | 0.03 |
| 26 | G09 | 26277 | TINF2 | NM_012461 | 215584 | 238525 | 0.87 | 0.96 | 0.64 | 0.06 |
| 26 | G10 | 7082 | TJP1 | NM_003257 | 212794 | 223344 | 0.86 | 0.90 | 0.88 | 2.13 |
| 26 | G11 | 7088 | TLE1 | NM_005077 | 222590 | 223409 | 0.90 | 0.90 | 0.21 | 1.13 |
| 26 | G12 | 7089 | TLE2 | NM_003260 | 233391 | 240033 | 0.94 | 0.97 | -0.24 | -0.18 |
| 26 | H01 | NA | NA | NA | 285745 | 277789 | 1.15 | 1.12 | -5.29 | -5.77 |
| 26 | H02 | NA | pos | NA | 113519 | 117387 | 0.46 | 0.47 | 10.95 | 19.53 |
| 26 | H03 | 7090 | TLE3 | NM_005078 | 270492 | 242487 | 1.09 | 0.98 | -2.80 | 0.10 |
| 26 | H04 | 7091 | TLE4 | NM_007005 | 231577 | 250874 | 0.93 | 1.01 | 0.37 | -0.07 |
| 26 | H05 | 4071 | TM4SF1 | NM_014220 | 232468 | 243614 | 0.93 | 0.98 | 0.65 | 0.30 |
| 26 | H06 | 7102 | TM4SF2 | NM_004615 | 228928 | 244479 | 0.92 | 0.99 | 0.04 | 0.32 |
| 26 | H07 | 7104 | TM4SF4 | NM_004617 | 228729 | 237242 | 0.92 | 0.96 | 0.22 | -0.11 |
| 26 | H08 | 117531 | TMC1 | NM_138691 | 230250 | 246440 | 0.93 | 1.00 | -0.61 | -0.75 |
| 26 | H09 | 10330 | TMEM4 | NM_014255 | 222768 | 241269 | 0.90 | 0.97 | -0.16 | -0.06 |
| 26 | H10 | 10329 | TMEM5 | NM_014254 | 222544 | 239730 | 0.90 | 0.97 | -0.17 | -0.15 |
| 26 | H11 | 9168 | TMSB10 | NM_021103 | 213429 | 229098 | 0.86 | 0.93 | 0.95 | 0.53 |
| 26 | H12 | 7127 | TNFAIP2 | NM_006291 | 230345 | 229719 | 0.93 | 0.93 | -0.08 | 1.75 |
| 27 | A01 | NA | pos | NA | 96472 | 134642 | 0.39 | 0.53 | 17.18 | 11.61 |
| 27 | A02 | NA | NA | NA | 294621 | 324553 | 1.18 | 1.27 | -7.11 | -10.54 |
| 27 | A03 | 7125 | TNNC2 | NM_003279 | 273099 | 271195 | 1.09 | 1.06 | -5.64 | -3.79 |
| 27 | A04 | 7136 | TNNI2 | NM_003282 | 280426 | 291661 | 1.12 | 1.15 | -5.19 | -5.71 |
| 27 | A05 | 7137 | TNNI3 | NM_000363 | 269274 | 320894 | 1.08 | 1.26 | -3.07 | -9.29 |
| 27 | A06 | 7138 | TNNT1 | NM_003283 | 242871 | 242997 | 0.97 | 0.95 | 0.47 | -0.22 |
| 27 | A07 | 7139 | TNNT2 | NM_000364 | 240190 | 225851 | 0.96 | 0.89 | 0.10 | 0.48 |
| 27 | A08 | 7143 | TNR | NM_003285 | 238187 | 229778 | 0.95 | 0.90 | 0.01 | 0.40 |
| 27 | A09 | 10766 | TOB2 | NM_016272 | 235184 | 240610 | 0.94 | 0.94 | 0.00 | -0.77 |
| 27 | A10 | 7150 | TOP1 | NM_003286 | 224415 | 220738 | 0.90 | 0.87 | 0.28 | 1.56 |
| 27 | A11 | 7153 | TOP2A | NM_001067 | 223005 | 227158 | 0.89 | 0.89 | 1.50 | 0.24 |
| 27 | A12 | 9214 | TOSO | NM_005449 | 234427 | 226771 | 0.94 | 0.89 | -1.02 | 0.72 |
| 27 | B01 | NA | neg | NA | 253059 | 299787 | 1.01 | 1.18 | -1.69 | -6.84 |
| 27 | B02 | NA | neg | NA | 248925 | 249643 | 1.00 | 0.98 | -1.18 | -0.99 |
| 27 | B03 | 11257 | TP53AP1 | NM_007233 | 226207 | 244199 | 0.91 | 0.96 | 0.43 | 0.18 |
| 27 | B04 | 9537 | TP53I11 | NM_006034 | 237111 | 264008 | 0.95 | 1.04 | 0.44 | -1.67 |
| 27 | B05 | 94241 | TP53INP1 | NM_033285 | 251947 | 248559 | 1.01 | 0.98 | -0.62 | -0.04 |
| 27 | B06 | 24150 | TP53TG3 | NM_015369 | 249307 | 238164 | 1.00 | 0.94 | 0.00 | 1.16 |
| 27 | B07 | 7162 | TPBG | NM_006670 | 245377 | 236752 | 0.98 | 0.93 | -0.21 | 0.02 |
| 27 | B08 | 7163 | TPD52 | NM_005079 | 240929 | 243625 | 0.96 | 0.96 | -0.01 | -0.40 |
| 27 | B09 | 7164 | TPD52L1 | NM_003287 | 246566 | 242445 | 0.99 | 0.95 | -1.08 | -0.17 |
| 27 | B10 | 7165 | TPD52L2 | NM_003288 | 234748 | 242579 | 0.94 | 0.95 | -0.66 | -0.17 |
| 27 | B11 | 7175 | TPR | NM_003292 | 226382 | 231436 | 0.91 | 0.91 | 1.41 | 0.56 |
| 27 | B12 | 7178 | TPT1 | NM_003295 | 219886 | 224603 | 0.88 | 0.88 | 1.08 | 1.79 |
| 27 | C01 | NA | pos | NA | 85206 | 121295 | 0.34 | 0.48 | 18.60 | 13.71 |
| 27 | C02 | NA | NA | NA | 249646 | 251424 | 1.00 | 0.99 | -1.56 | -1.47 |
| 27 | C03 | 126382 | TRA16 | NM_176880 | 234085 | 244889 | 0.94 | 0.96 | -0.82 | -0.18 |
| 27 | C04 | 7185 | TRAF1 | NM_005658 | 238346 | 249537 | 0.95 | 0.98 | 0.00 | -0.26 |
| 27 | C05 | 23471 | TRAM1 | NM_014294 | 233686 | 221245 | 0.94 | 0.87 | 1.33 | 2.87 |
| 27 | C06 | 9862 | THRAP4 | NM_014815 | 247042 | 234275 | 0.99 | 0.92 | 0.00 | 1.34 |
| 27 | C07 | 9318 | TRIP15 | NM_004236 | 240434 | 230880 | 0.96 | 0.91 | 0.11 | 0.43 |
| 27 | C08 | 9792 | SERTAD2 | XM_376059 | 209863 | 226120 | 0.84 | 0.89 | 3.52 | 1.37 |
| 27 | C09 | 8989 | TRPA1 | NM_007332 | 234736 | 237604 | 0.94 | 0.93 | 0.09 | 0.13 |
| 27 | C10 | 29850 | TRPM5 | NM_014555 | 232507 | 258211 | 0.93 | 1.01 | -0.67 | -2.26 |
| 27 | C11 | 79054 | TRPM8 | NM_024080 | 246842 | 234942 | 0.99 | 0.92 | -1.38 | -0.12 |
| 27 | C12 | 7442 | TRPV1 | NM_018727 | 246071 | 250879 | 0.99 | 0.98 | -2.41 | -1.55 |
| 27 | D01 | NA | neg | NA | 251822 | 253097 | 1.01 | 0.99 | -1.80 | -0.99 |
| 27 | D02 | NA | neg | NA | 247987 | 243909 | 0.99 | 0.96 | -1.33 | 0.08 |
| 27 | D03 | 130560 | SPATA3 | NM_139073 | 238116 | 251318 | 0.95 | 0.99 | -1.29 | -0.25 |
| 27 | D04 | 7248 | TSC1 | NM_000368 | 237992 | 234930 | 0.95 | 0.92 | 0.07 | 2.12 |
| 27 | D05 | 7260 | TSSC1 | NM_003310 | 246786 | 253879 | 0.99 | 1.00 | -0.25 | -0.26 |
| 27 | D06 | 10078 | TSSC4 | NM_005706 | 241316 | 252151 | 0.97 | 0.99 | 0.72 | -0.07 |
| 27 | D07 | 7263 | TST | NM_003312 | 242014 | 240562 | 0.97 | 0.94 | -0.07 | -0.02 |
| 27 | D08 | 7268 | TTC4 | NM_004623 | 236032 | 236139 | 0.95 | 0.93 | 0.33 | 0.87 |
| 27 | D09 | 7275 | TUB | NM_003320 | 215798 | 259362 | 0.86 | 1.02 | 2.43 | -1.74 |
| 27 | D10 | 7846 | TUBA3 | NM_006009 | 222315 | 243169 | 0.89 | 0.95 | 0.60 | 0.17 |
| 27 | D11 | 51807 | TUBA8 | NM_018943 | 236282 | 228311 | 0.95 | 0.90 | -0.07 | 1.32 |
| 27 | D12 | 10383 | TUBB2 | NM_006088 | 244012 | 243146 | 0.98 | 0.95 | -2.14 | 0.02 |

| | | | | | | | | | | |
|---|---|---|---|---|---|---|---|---|---|---|
| 27 | E01 | NA | neg | NA | 250450 | 271239 | 1.00 | 1.06 | -3.56 | -4.65 |
| 27 | E02 | NA | neg | NA | 238934 | 256304 | 0.96 | 1.01 | -2.15 | -2.91 |
| 27 | E03 | 27175 | TUBG2 | NM_016437 | 191665 | 210553 | 0.77 | 0.83 | 2.48 | 2.96 |
| 27 | E04 | 10844 | TUBGCP2 | NM_006659 | 224622 | 229653 | 0.90 | 0.90 | -0.22 | 1.20 |
| 27 | E05 | 7287 | TULP1 | NM_003322 | 225476 | 228261 | 0.90 | 0.90 | 0.44 | 1.19 |
| 27 | E06 | 7288 | TULP2 | NM_003323 | 235086 | 244135 | 0.94 | 0.96 | -0.44 | -0.68 |
| 27 | E07 | 7295 | TXN | NM_003329 | 239502 | 227872 | 0.96 | 0.89 | -1.68 | -0.08 |
| 27 | E08 | 7296 | TXNRD1 | NM_003330 | 241819 | 250006 | 0.97 | 0.98 | -2.30 | -2.28 |
| 27 | E09 | 7298 | TYMS | NM_001071 | 218132 | 230690 | 0.87 | 0.91 | 0.22 | 0.06 |
| 27 | E10 | 51271 | UBAP1 | NM_016525 | 209071 | 235108 | 0.84 | 0.92 | 0.30 | -0.44 |
| 27 | E11 | 55833 | UBAP2 | NM_018449 | 238441 | 235992 | 0.95 | 0.93 | -2.26 | -1.11 |
| 27 | E12 | 7314 | UBB | NM_018955 | 158064 | 221966 | 0.63 | 0.87 | 6.47 | 0.95 |
| 27 | F01 | NA | NA | NA | 241721 | 288861 | 0.97 | 1.13 | -0.70 | -7.10 |
| 27 | F02 | NA | pos | NA | 150094 | 143957 | 0.60 | 0.57 | 10.53 | 9.80 |
| 27 | F03 | 7316 | UBC | NM_021009 | 42600 | 119508 | 0.17 | 0.47 | 22.54 | 13.18 |
| 27 | F04 | 10537 | UBD | NM_006398 | 219823 | 234348 | 0.88 | 0.92 | 2.16 | 0.26 |
| 27 | F05 | 7318 | UBE1L | NM_003335 | 241556 | 236240 | 0.97 | 0.93 | 0.25 | -0.14 |
| 27 | F06 | 7326 | UBE2G1 | NM_003342 | 253845 | 234380 | 1.02 | 0.92 | -0.95 | 0.07 |
| 27 | F07 | 9246 | UBE2L6 | NM_004223 | 256275 | 245734 | 1.03 | 0.96 | -1.95 | -2.56 |
| 27 | F08 | 9040 | UBE2M | NM_003969 | 241729 | 232273 | 0.97 | 0.91 | -0.51 | -0.61 |
| 27 | F09 | 7334 | UBE2N | NM_003348 | 236553 | 215255 | 0.95 | 0.85 | -0.25 | 1.47 |
| 27 | F10 | 7341 | SUMO1 | NM_003352 | 228343 | 221185 | 0.91 | 0.87 | -0.28 | 0.79 |
| 27 | F11 | 5412 | UBL3 | NM_007106 | 226053 | 223460 | 0.91 | 0.88 | 1.05 | -0.05 |
| 27 | F12 | 59286 | UBL5 | NM_024292 | 207644 | 239648 | 0.83 | 0.94 | 2.18 | -1.50 |
| 27 | G01 | NA | neg | NA | 247318 | 261191 | 0.99 | 1.03 | -1.03 | -2.94 |
| 27 | G02 | NA | neg | NA | 257031 | 250463 | 1.03 | 0.98 | -2.22 | -1.68 |
| 27 | G03 | 29855 | UBN1 | NM_016936 | 232864 | 230949 | 0.93 | 0.91 | -0.43 | 1.12 |
| 27 | G04 | 56061 | UBPH | NM_019116 | 240321 | 236667 | 0.96 | 0.93 | 0.00 | 0.92 |
| 27 | G05 | 29979 | UBQLN1 | NM_013438 | 239907 | 242737 | 0.96 | 0.95 | 0.81 | 0.04 |
| 27 | G06 | 50613 | UBQLN3 | NM_017481 | 244078 | 247195 | 0.98 | 0.97 | 0.60 | -0.49 |
| 27 | G07 | 54658 | UGT1A1 | NM_000463 | 241466 | 233944 | 0.97 | 0.92 | 0.22 | -0.25 |
| 27 | G08 | 9094 | UNC119 | NM_005148 | 241336 | 231541 | 0.97 | 0.91 | -0.11 | 0.41 |
| 27 | G09 | 10497 | UNC13B | NM_006377 | 237352 | 236424 | 0.95 | 0.93 | 0.00 | -0.06 |
| 27 | G10 | 7381 | UQCRB | NM_006294 | 220771 | 227286 | 0.88 | 0.89 | 1.00 | 1.02 |
| 27 | G11 | 7401 | USH3A | NM_052995 | 238426 | 231735 | 0.95 | 0.91 | -0.11 | -0.08 |
| 27 | G12 | 7405 | UVRAG | NM_003369 | 245045 | 243741 | 0.98 | 0.96 | -2.05 | -1.05 |
| 27 | H01 | NA | NA | NA | 258777 | 288566 | 1.04 | 1.13 | -2.73 | -5.37 |
| 27 | H02 | NA | pos | NA | 113369 | 108997 | 0.45 | 0.43 | 15.10 | 15.57 |
| 27 | H03 | 8673 | VAMP8 | NM_003761 | 220993 | 255988 | 0.89 | 1.01 | 0.73 | -1.04 |
| 27 | H04 | 7411 | VBP1 | NM_003372 | 254889 | 278775 | 1.02 | 1.09 | -2.08 | -3.24 |
| 27 | H05 | 29802 | VPREB3 | NM_013378 | 254213 | 245008 | 1.02 | 0.96 | -1.24 | 0.53 |
| 27 | H06 | 6293 | VPS52 | NM_022553 | 254813 | 247803 | 1.02 | 0.97 | -1.01 | 0.19 |
| 27 | H07 | 29119 | CTNNA3 | NM_013266 | 240312 | 234334 | 0.96 | 0.92 | 0.07 | 0.46 |
| 27 | H08 | 7447 | VSNL1 | NM_003385 | 232002 | 246224 | 0.93 | 0.97 | 0.75 | -0.55 |
| 27 | H09 | 23558 | WBP2 | NM_012478 | 235517 | 233604 | 0.94 | 0.92 | -0.06 | 1.02 |
| 27 | H10 | 55695 | WBSCR20A | NM_018044 | 240278 | 249109 | 0.96 | 0.98 | -1.68 | -0.77 |
| 27 | H11 | 27159 | CHIA | NM_021797 | 234571 | 237166 | 0.94 | 0.93 | 0.07 | 0.05 |
| 27 | H12 | 30000 | TNPO2 | NM_013433 | 217567 | 241441 | 0.87 | 0.95 | 1.02 | -0.02 |
| 28 | A01 | NA | pos | NA | 126468 | 132213 | 0.50 | 0.53 | 13.38 | 16.49 |
| 28 | A02 | NA | NA | NA | 311366 | 310529 | 1.23 | 1.25 | -7.95 | -11.04 |
| 28 | A03 | 1118 | CHIT1 | NM_003465 | 251478 | 274596 | 0.99 | 1.11 | -1.08 | -4.67 |
| 28 | A04 | 56994 | CHPT1 | NM_020244 | 313461 | 232656 | 1.24 | 0.94 | -7.40 | 1.29 |
| 28 | A05 | 54108 | CHRAC1 | NM_017444 | 240602 | 281811 | 0.95 | 1.13 | 0.88 | -5.31 |
| 28 | A06 | 10845 | CLPX | NM_006660 | 236539 | 237960 | 0.93 | 0.96 | 0.65 | 0.19 |
| 28 | A07 | 26504 | CNNM4 | NM_020184 | 243133 | 252948 | 0.96 | 1.02 | 0.02 | -2.11 |
| 28 | A08 | 1312 | COMT | NM_000754 | 219556 | 223694 | 0.87 | 0.90 | 2.88 | 1.84 |
| 28 | A09 | 1362 | CPD | NM_001304 | 246434 | 240659 | 0.97 | 0.97 | -1.08 | -0.09 |
| 28 | A10 | 1363 | CPE | NM_001873 | 238438 | 228446 | 0.94 | 0.92 | -0.72 | 0.06 |
| 28 | A11 | 1368 | CPM | NM_001874 | 236188 | 233987 | 0.93 | 0.94 | -0.02 | -0.17 |
| 28 | A12 | 1369 | CPN1 | NM_001308 | 226782 | 236021 | 0.90 | 0.95 | 1.84 | 0.53 |
| 28 | B01 | NA | neg | NA | 255148 | 301209 | 1.01 | 1.21 | -2.24 | -10.63 |
| 28 | B02 | NA | neg | NA | 248815 | 238705 | 0.98 | 0.96 | -1.51 | -0.98 |
| 28 | B03 | 1373 | CPS1 | NM_001875 | 233549 | 238246 | 0.92 | 0.96 | 0.21 | -0.08 |
| 28 | B04 | 1429 | CRYZ | NM_001889 | 243500 | 237118 | 0.96 | 0.95 | -0.11 | -0.43 |
| 28 | B05 | 9946 | CRYZL1 | NM_005111 | 255376 | 248578 | 1.01 | 1.00 | -1.60 | -1.21 |
| 28 | B06 | 1487 | CTBP1 | NM_001328 | 234056 | 231641 | 0.92 | 0.93 | 0.16 | 0.14 |
| 28 | B07 | 1585 | CYP11B2 | NM_000498 | 235582 | 228777 | 0.93 | 0.92 | 0.11 | 0.59 |
| 28 | B08 | 1586 | CYP17A1 | NM_000102 | 238855 | 224226 | 0.94 | 0.90 | -0.12 | 0.73 |
| 28 | B09 | 1543 | CYP1A1 | NM_000499 | 251491 | 236108 | 0.99 | 0.95 | -2.44 | -0.42 |
| 28 | B10 | 1594 | CYP27B1 | NM_000785 | 242738 | 224195 | 0.96 | 0.90 | -2.00 | -0.32 |
| 28 | B11 | 1548 | CYP2A6 | NM_000762 | 218469 | 221213 | 0.86 | 0.89 | 1.24 | 0.77 |
| 28 | B12 | 1549 | CYP2A7 | NM_000764 | 227819 | 231163 | 0.90 | 0.93 | 0.94 | 0.25 |
| 28 | C01 | NA | pos | NA | 125170 | 127742 | 0.49 | 0.51 | 13.18 | 16.45 |
| 28 | C02 | NA | NA | NA | 250832 | 250224 | 0.99 | 1.01 | -1.32 | -2.46 |
| 28 | C03 | 1562 | CYP2C18 | NM_000772 | 240884 | 241500 | 0.95 | 0.97 | -0.21 | -0.29 |
| 28 | C04 | 1557 | CYP2C19 | NM_000769 | 223504 | 223625 | 0.88 | 0.90 | 2.63 | 1.95 |
| 28 | C05 | 1565 | CYP2D6 | NM_000106 | 244385 | 244165 | 0.97 | 0.98 | 0.10 | -0.23 |
| 28 | C06 | 1615 | DARS | NM_001349 | 239902 | 224418 | 0.95 | 0.90 | -0.08 | 1.55 |
| 28 | C07 | 1621 | DBH | NM_000787 | 219323 | 204259 | 0.87 | 0.82 | 2.42 | 4.68 |
| 28 | C08 | 1629 | DBT | NM_001918 | 245983 | 230136 | 0.97 | 0.93 | -0.51 | 0.11 |
| 28 | C09 | 9937 | DCLRE1A | NM_014881 | 212150 | 239296 | 0.84 | 0.96 | 2.52 | -0.62 |
| 28 | C10 | 1638 | DCT | NM_001922 | 224243 | 220619 | 0.89 | 0.89 | 0.57 | 0.53 |
| 28 | C11 | 1663 | DDX11 | NM_004399 | 238990 | 230523 | 0.94 | 0.93 | -0.69 | -0.37 |
| 28 | C12 | 8886 | DDX18 | NM_006773 | 248219 | 242959 | 0.98 | 0.98 | -0.98 | -1.28 |
| 28 | D01 | NA | neg | NA | 253745 | 257522 | 1.00 | 1.04 | -1.92 | -3.13 |
| 28 | D02 | NA | neg | NA | 252500 | 246099 | 1.00 | 0.99 | -1.78 | -1.37 |
| 28 | D03 | 11218 | DDX20 | NM_007204 | 252382 | 232389 | 1.00 | 0.94 | -1.81 | 1.57 |
| 28 | D04 | 1656 | DDX6 | NM_004397 | 239502 | 232948 | 0.95 | 0.94 | 0.51 | 0.97 |

| | | | | | | | | | | |
|---|---|---|---|---|---|---|---|---|---|---|
| 28 | D05 | 1676 | DFFA | NM_004401 | 246474 | 252458 | 0.97 | 1.02 | -0.42 | -1.05 |
| 28 | D06 | 1677 | DFFB | NM_004402 | 236634 | 250456 | 0.93 | 1.01 | 0.02 | -2.02 |
| 28 | D07 | 8449 | DHX16 | NM_003587 | 238121 | 242137 | 0.94 | 0.97 | -0.02 | -0.72 |
| 28 | D08 | 81889 | DKFZP566J204 | NM_031208 | 212973 | 235018 | 0.84 | 0.95 | 3.02 | -0.18 |
| 28 | D09 | 11144 | DMC1 | NM_007068 | 246416 | 224112 | 0.97 | 0.90 | -1.70 | 2.19 |
| 28 | D10 | 1773 | DNASE1 | NM_005223 | 219622 | 219535 | 0.87 | 0.88 | 0.83 | 1.16 |
| 28 | D11 | 1777 | DNASE2 | NM_001375 | 222062 | 230552 | 0.88 | 0.93 | 0.99 | 0.08 |
| 28 | D12 | 10059 | DNM1L | NM_005690 | 243822 | 243626 | 0.96 | 0.98 | -0.75 | -0.92 |
| 28 | E01 | NA | neg | NA | 251690 | 237043 | 0.99 | 0.95 | -1.56 | -2.12 |
| 28 | E02 | NA | neg | NA | 244179 | 231375 | 0.96 | 0.93 | -0.69 | -1.24 |
| 28 | E03 | 1786 | DNMT1 | NM_001379 | 220539 | 214734 | 0.87 | 0.86 | 2.00 | 2.15 |
| 28 | E04 | 1787 | DNMT2 | NM_004412 | 236901 | 222570 | 0.94 | 0.90 | 0.95 | 0.43 |
| 28 | E05 | 1788 | DNMT3A | NM_022552 | 247339 | 222512 | 0.98 | 0.90 | -0.39 | 1.42 |
| 28 | E06 | 1789 | DNMT3B | NM_006892 | 236223 | 228811 | 0.93 | 0.92 | 0.20 | -0.82 |
| 28 | E07 | 1791 | DNTT | NM_004088 | 247353 | 206440 | 0.98 | 0.83 | -0.95 | 2.65 |
| 28 | E08 | 80174 | DRF1 | NM_025104 | 248405 | 247775 | 0.98 | 1.00 | -0.93 | -4.30 |
| 28 | E09 | 1854 | DUT | NM_001948 | 212530 | 230241 | 0.84 | 0.93 | 2.34 | -0.91 |
| 28 | E10 | 1861 | DYT1 | NM_000113 | 205209 | 204820 | 0.81 | 0.82 | 2.62 | 1.28 |
| 28 | E11 | 1889 | ECE1 | NM_001397 | 233543 | 223656 | 0.92 | 0.90 | -0.21 | -1.00 |
| 28 | E12 | 9427 | ECEL1 | NM_004826 | 247561 | 225352 | 0.98 | 0.91 | -1.05 | -0.25 |
| 28 | F01 | NA | NA | NA | 244638 | 251868 | 0.97 | 1.01 | -1.37 | -3.71 |
| 28 | F02 | NA | pos | NA | 130032 | 155930 | 0.51 | 0.63 | 11.86 | 11.10 |
| 28 | F03 | 9695 | EDEM1 | XM_376201 | 229470 | 234071 | 0.91 | 0.94 | 0.34 | -0.14 |
| 28 | F04 | 9538 | EI24 | NM_004879 | 238742 | 233959 | 0.94 | 0.94 | 0.11 | -0.64 |
| 28 | F05 | 25909 | ELYS | NM_015446 | 233233 | 231744 | 0.92 | 0.93 | 0.62 | 0.69 |
| 28 | F06 | 2028 | ENPEP | NM_001977 | 232780 | 230953 | 0.92 | 0.93 | -0.02 | -0.46 |
| 28 | F07 | 5169 | ENPP3 | NM_005021 | 258596 | 244893 | 1.02 | 0.99 | -2.88 | -2.59 |
| 28 | F08 | 953 | ENTPD1 | NM_001776 | 241315 | 218561 | 0.95 | 0.88 | -0.74 | 0.91 |
| 28 | F09 | 956 | ENTPD3 | NM_001248 | 223076 | 209934 | 0.88 | 0.85 | 0.50 | 2.92 |
| 28 | F10 | 957 | ENTPD5 | NM_001249 | 230756 | 253769 | 0.91 | 1.02 | -0.95 | -5.58 |
| 28 | F11 | 2053 | EPHX2 | NM_001979 | 226126 | 216054 | 0.89 | 0.87 | 0.02 | 0.87 |
| 28 | F12 | 2067 | ERCC1 | NM_001983 | 233441 | 226388 | 0.92 | 0.91 | -0.04 | 0.28 |
| 28 | G01 | NA | neg | NA | 258694 | 250666 | 1.02 | 1.01 | -2.08 | -1.81 |
| 28 | G02 | NA | neg | NA | 277813 | 251312 | 1.10 | 1.01 | -4.28 | -1.91 |
| 28 | G03 | 2068 | ERCC2 | NM_000400 | 264685 | 243722 | 1.05 | 0.98 | -2.81 | 0.08 |
| 28 | G04 | 2073 | ERCC5 | NM_000123 | 253126 | 244063 | 1.00 | 0.98 | -0.64 | -0.48 |
| 28 | G05 | 9156 | EXO1 | NM_003686 | 237928 | 245833 | 0.94 | 0.99 | 0.99 | 0.23 |
| 28 | G06 | 2138 | EYA1 | NM_000503 | 246520 | 239319 | 0.97 | 0.96 | -0.70 | -0.03 |
| 28 | G07 | 2140 | EYA3 | NM_001990 | 239026 | 234192 | 0.94 | 0.94 | 0.29 | 0.77 |
| 28 | G08 | 2070 | EYA4 | NM_004100 | 223202 | 236282 | 0.88 | 0.95 | 2.26 | -0.11 |
| 28 | G09 | 2155 | F7 | NM_000131 | 239604 | 239364 | 0.95 | 0.96 | -0.50 | 0.09 |
| 28 | G10 | 2158 | F9 | NM_000133 | 228288 | 229097 | 0.90 | 0.92 | 0.25 | -0.06 |
| 28 | G11 | 2181 | ACSL3 | NM_004457 | 236026 | 230331 | 0.93 | 0.93 | -0.21 | 0.38 |
| 28 | G12 | 2182 | ACSL4 | NM_004458 | 239196 | 242605 | 0.94 | 0.98 | 0.21 | -0.50 |
| 28 | H01 | NA | NA | NA | 249344 | 270071 | 0.99 | 1.09 | -1.08 | -4.23 |
| 28 | H02 | NA | pos | NA | 149819 | 128589 | 0.59 | 0.52 | 10.40 | 17.62 |
| 28 | H03 | 2194 | FASN | NM_004104 | 231875 | 242250 | 0.92 | 0.98 | 0.89 | 0.89 |
| 28 | H04 | 54850 | FBXL12 | NM_017703 | 248279 | 256012 | 0.98 | 1.03 | -0.16 | -1.75 |
| 28 | H05 | 23014 | FBXO21 | NM_015002 | 246587 | 246518 | 0.97 | 0.99 | -0.10 | 0.70 |
| 28 | H06 | 26262 | FBXO23 | NM_130465 | 246317 | 242609 | 0.97 | 0.98 | -0.76 | 0.03 |
| 28 | H07 | 26261 | FBXO24 | NM_033506 | 241175 | 246792 | 0.95 | 0.99 | -0.04 | -0.59 |
| 28 | H08 | 23291 | FBXW11 | NM_012300 | 241016 | 242575 | 0.95 | 0.98 | 0.12 | -0.51 |
| 28 | H09 | 2222 | FDFT1 | NM_004462 | 229050 | 233572 | 0.90 | 0.94 | 0.64 | 1.56 |
| 28 | H10 | 2235 | FECH | NM_000140 | 231840 | 243164 | 0.92 | 0.98 | -0.25 | -1.65 |
| 28 | H11 | 2237 | FEN1 | NM_004111 | 224454 | 237036 | 0.89 | 0.95 | 1.05 | -0.08 |
| 28 | H12 | 55033 | FKBP14 | NM_017946 | 239887 | 235593 | 0.95 | 0.95 | 0.04 | 1.16 |
| 29 | A01 | NA | pos | NA | 114995 | 119325 | 0.45 | 0.46 | 15.48 | 14.62 |
| 29 | A02 | NA | NA | NA | 300770 | 317035 | 1.18 | 1.22 | -6.75 | -9.59 |
| 29 | A03 | 2286 | FKBP2 | NM_004470 | 273090 | 292217 | 1.07 | 1.12 | -3.11 | -4.91 |
| 29 | A04 | 2288 | FKBP4 | NM_002014 | 281637 | 250598 | 1.10 | 0.96 | -3.08 | -0.02 |
| 29 | A05 | 2289 | FKBP5 | NM_004117 | 253374 | 252817 | 0.99 | 0.97 | -1.04 | -1.31 |
| 29 | A06 | 8468 | FKBP6 | NM_003602 | 282637 | 247561 | 1.11 | 0.95 | -4.39 | -0.66 |
| 29 | A07 | 63943 | FKBPL | NM_022110 | 238071 | 221094 | 0.93 | 0.85 | 0.71 | 1.48 |
| 29 | A08 | 84866 | TMEM25 | NM_032780 | 239496 | 226803 | 0.94 | 0.87 | 0.04 | 0.99 |
| 29 | A09 | 54902 | FLJ20343 | NM_017775 | 240477 | 240138 | 0.94 | 0.92 | -0.08 | -0.60 |
| 29 | A10 | 2547 | G22P1 | NM_001469 | 232595 | 228458 | 0.91 | 0.88 | 0.92 | 0.40 |
| 29 | A11 | 2617 | GARS | NM_002047 | 234306 | 233816 | 0.92 | 0.90 | 0.33 | 0.04 |
| 29 | A12 | 2633 | GBP1 | NM_002053 | 226965 | 215170 | 0.89 | 0.83 | 2.59 | 3.64 |
| 29 | B01 | NA | neg | NA | 298098 | 296757 | 1.17 | 1.14 | -8.05 | -7.00 |
| 29 | B02 | NA | neg | NA | 254536 | 255646 | 1.00 | 0.98 | -2.84 | -1.96 |
| 29 | B03 | 2634 | GBP2 | NM_004120 | 233671 | 250751 | 0.91 | 0.96 | -0.02 | 0.28 |
| 29 | B04 | 80318 | GKAP1 | NM_025211 | 247073 | 253668 | 0.97 | 0.97 | -0.57 | -0.29 |
| 29 | B05 | 2820 | GPD2 | NM_000408 | 227147 | 247393 | 0.89 | 0.95 | 0.48 | -0.53 |
| 29 | B06 | 2937 | GSS | NM_000178 | 232127 | 237103 | 0.91 | 0.91 | 0.03 | 0.73 |
| 29 | B07 | 26762 | HAVCR1 | NM_012206 | 229129 | 237616 | 0.90 | 0.91 | 0.16 | -0.43 |
| 29 | B08 | 79885 | HDAC11 | NM_024827 | 245951 | 236571 | 0.96 | 0.91 | -2.35 | -0.10 |
| 29 | B09 | 8841 | HDAC3 | NM_003883 | 233342 | 236082 | 0.91 | 0.91 | -0.85 | 0.01 |
| 29 | B10 | 55869 | HDAC8 | NM_018486 | 232345 | 232843 | 0.91 | 0.89 | -0.68 | -0.02 |
| 29 | B11 | 3265 | HRAS | NM_005343 | 208426 | 202601 | 0.81 | 0.78 | 1.80 | 3.97 |
| 29 | B12 | 3312 | HSPA8 | NM_006597 | 223952 | 224993 | 0.88 | 0.86 | 1.33 | 2.55 |
| 29 | C01 | NA | pos | NA | 113394 | 119563 | 0.44 | 0.46 | 14.12 | 13.18 |
| 29 | C02 | NA | NA | NA | 239494 | 251407 | 0.94 | 0.96 | -0.96 | -2.96 |
| 29 | C03 | 8519 | IFITM1 | NM_003641 | 231074 | 249562 | 0.90 | 0.96 | 0.37 | -1.10 |
| 29 | C04 | 3508 | IGHMBP2 | NM_002180 | 213142 | 238715 | 0.83 | 0.92 | 3.56 | 0.02 |
| 29 | C05 | 3612 | IMPA1 | NM_005536 | 227728 | 220539 | 0.89 | 0.85 | 0.48 | 1.23 |
| 29 | C06 | 3613 | IMPA2 | NM_014214 | 219065 | 230204 | 0.86 | 0.88 | 1.67 | 0.05 |
| 29 | C07 | 3628 | INPP1 | NM_002194 | 244194 | 225115 | 0.95 | 0.86 | -1.57 | -0.42 |
| 29 | C08 | 28512 | NKIRAS1 | NM_020345 | 226227 | 211789 | 0.88 | 0.81 | 0.08 | 1.42 |

| | | | | | | | | | | |
|---|---|---|---|---|---|---|---|---|---|---|
| 29 | C09 | 28511 | NKIRAS2 | NM_001001349 | 227873 | 221548 | 0.89 | 0.85 | -0.12 | 0.27 |
| 29 | C10 | 23185 | KIAA0217 | XM_040265 | 228510 | 229772 | 0.89 | 0.88 | -0.14 | -1.17 |
| 29 | C11 | 23227 | KIAA0303 | XM_291141 | 227544 | 228718 | 0.89 | 0.88 | -0.41 | -0.75 |
| 29 | C12 | 25778 | DUSTYPK | XM_290898 | 238279 | 233494 | 0.93 | 0.90 | -0.31 | -0.01 |
| 29 | D01 | NA | neg | NA | 266286 | 298858 | 1.04 | 1.15 | -4.10 | -8.44 |
| 29 | D02 | NA | neg | NA | 257010 | 260585 | 1.00 | 1.00 | -2.99 | -3.75 |
| 29 | D03 | 3845 | KRAS2 | NM_004985 | 242130 | 253489 | 0.95 | 0.97 | -0.88 | -1.24 |
| 29 | D04 | 8844 | KSR | XM_290793 | 249087 | 240743 | 0.97 | 0.92 | -0.67 | 0.11 |
| 29 | D05 | 51056 | LAP3 | NM_015907 | 205626 | 213417 | 0.80 | 0.82 | 3.20 | 2.44 |
| 29 | D06 | 3931 | LCAT | NM_000229 | 210480 | 233804 | 0.82 | 0.90 | 2.77 | -0.05 |
| 29 | D07 | 79132 | LGP2 | NM_024119 | 232959 | 217778 | 0.91 | 0.84 | -0.16 | 0.81 |
| 29 | D08 | 3978 | LIG1 | NM_000234 | 206930 | 212925 | 0.81 | 0.82 | 2.46 | 1.61 |
| 29 | D09 | 8513 | LIPF | NM_004190 | 212724 | 226741 | 0.83 | 0.87 | 1.76 | -0.03 |
| 29 | D10 | 9388 | LIPG | NM_006033 | 237483 | 222785 | 0.93 | 0.85 | -1.15 | 0.02 |
| 29 | D11 | 4012 | LNPEP | NM_005575 | 224395 | 232982 | 0.88 | 0.89 | 0.04 | -0.94 |
| 29 | D12 | 4015 | LOX | NM_002317 | 236649 | 237510 | 0.93 | 0.91 | -0.04 | -0.17 |
| 29 | E01 | NA | neg | NA | 250272 | 315621 | 0.98 | 1.21 | -3.02 | -10.38 |
| 29 | E02 | NA | neg | NA | 240367 | 252914 | 0.94 | 0.97 | -1.84 | -2.70 |
| 29 | E03 | 22949 | LTB4DH | NM_012212 | 224915 | 233174 | 0.88 | 0.89 | 0.34 | 1.35 |
| 29 | E04 | 4057 | LTF | NM_002343 | 234610 | 239392 | 0.92 | 0.92 | 0.23 | 0.38 |
| 29 | E05 | 9583 | LYSAL1 | NM_004901 | 214481 | 248717 | 0.84 | 0.95 | 1.30 | -1.77 |
| 29 | E06 | 4143 | MAT1A | NM_000429 | 250649 | 241177 | 0.98 | 0.93 | -2.88 | -0.85 |
| 29 | E07 | 8930 | MBD4 | NM_003925 | 226524 | 213809 | 0.89 | 0.82 | -0.22 | 1.40 |
| 29 | E08 | 51360 | MBTPS2 | NM_015884 | 242563 | 254383 | 0.95 | 0.98 | -2.64 | -3.36 |
| 29 | E09 | 56922 | MCCC1 | NM_020166 | 211916 | 227428 | 0.83 | 0.87 | 1.02 | -0.01 |
| 29 | E10 | 4171 | MCM2 | NM_004526 | 218925 | 206335 | 0.86 | 0.79 | 0.24 | 2.15 |
| 29 | E11 | 4172 | MCM3 | NM_002388 | 227379 | 226542 | 0.89 | 0.87 | -1.16 | -0.04 |
| 29 | E12 | 4173 | MCM4 | NM_005914 | 238113 | 236972 | 0.93 | 0.91 | -1.06 | 0.01 |
| 29 | F01 | NA | NA | NA | 251227 | 249388 | 0.98 | 0.96 | -2.68 | -2.68 |
| 29 | F02 | NA | pos | NA | 133710 | 130558 | 0.52 | 0.50 | 11.38 | 11.87 |
| 29 | F03 | 4174 | MCM5 | NM_006739 | 230722 | 238447 | 0.90 | 0.91 | 0.10 | 0.30 |
| 29 | F04 | 4175 | MCM6 | NM_005915 | 235300 | 239567 | 0.92 | 0.92 | 0.60 | -0.05 |
| 29 | F05 | 4176 | MCM7 | NM_005916 | 233146 | 221755 | 0.91 | 0.85 | -0.48 | 1.12 |
| 29 | F06 | 4224 | MEP1A | NM_005588 | 219679 | 230438 | 0.86 | 0.88 | 1.28 | 0.06 |
| 29 | F07 | 4285 | MIPEP | NM_005932 | 231064 | 238279 | 0.90 | 0.91 | -0.31 | -2.00 |
| 29 | F08 | 4312 | MMP1 | NM_002421 | 216055 | 238276 | 0.84 | 0.91 | 0.99 | -1.80 |
| 29 | F09 | 4319 | MMP10 | NM_002425 | 200401 | 221519 | 0.78 | 0.85 | 2.85 | 0.30 |
| 29 | F10 | 4320 | MMP11 | NM_005940 | 225468 | 226547 | 0.88 | 0.87 | -0.09 | -0.74 |
| 29 | F11 | 4321 | MMP12 | NM_002426 | 227237 | 216202 | 0.89 | 0.83 | -0.68 | 0.82 |
| 29 | F12 | 4322 | MMP13 | NM_002427 | 256132 | 242410 | 1.00 | 0.93 | -2.76 | -1.07 |
| 29 | G01 | NA | neg | NA | 248579 | 254871 | 0.97 | 0.98 | -1.88 | -2.13 |
| 29 | G02 | NA | neg | NA | 273944 | 260851 | 1.07 | 1.00 | -4.91 | -2.86 |
| 29 | G03 | 4327 | MMP19 | NM_002429 | 250894 | 253154 | 0.98 | 0.97 | -1.83 | -0.28 |
| 29 | G04 | 4313 | MMP2 | NM_004530 | 231012 | 236910 | 0.90 | 0.91 | 1.60 | 1.50 |
| 29 | G05 | 9313 | MMP20 | NM_004771 | 247555 | 236524 | 0.97 | 0.91 | -1.71 | 0.53 |
| 29 | G06 | 8511 | MMP23A | NM_006983 | 234725 | 238563 | 0.92 | 0.92 | -0.03 | 0.29 |
| 29 | G07 | 8510 | MMP23B | NM_006983 | 227940 | 232537 | 0.89 | 0.89 | 0.55 | -0.07 |
| 29 | G08 | 64386 | MMP25 | NM_022468 | 232495 | 239182 | 0.91 | 0.92 | -0.49 | -0.68 |
| 29 | G09 | 56547 | MMP26 | NM_021801 | 230104 | 233453 | 0.90 | 0.90 | -0.21 | 0.07 |
| 29 | G10 | 79148 | MMP28 | NM_024302 | 228033 | 220132 | 0.89 | 0.84 | 0.09 | 1.27 |
| 29 | G11 | 4314 | MMP3 | NM_002422 | 224514 | 233573 | 0.88 | 0.90 | 0.13 | -0.08 |
| 29 | G12 | 4316 | MMP7 | NM_002423 | 236788 | 245661 | 0.93 | 0.94 | 0.04 | -0.24 |
| 29 | H01 | NA | NA | NA | 284839 | 253058 | 1.11 | 0.97 | -5.48 | -1.54 |
| 29 | H02 | NA | pos | NA | 129373 | 134723 | 0.51 | 0.52 | 13.12 | 12.95 |
| 29 | H03 | 4317 | MMP8 | NM_002424 | 241573 | 237351 | 0.94 | 0.91 | 0.02 | 2.02 |
| 29 | H04 | 4318 | MMP9 | NM_004994 | 252400 | 252704 | 0.99 | 0.97 | -0.23 | -0.07 |
| 29 | H05 | 64112 | MOAP1 | NM_022151 | 250191 | 256433 | 0.98 | 0.98 | -1.30 | -1.54 |
| 29 | H06 | 4350 | MPG | NM_002434 | 243378 | 245072 | 0.95 | 0.94 | -0.33 | -0.14 |
| 29 | H07 | 4351 | MPI | NM_002435 | 235675 | 234307 | 0.92 | 0.90 | 0.36 | 0.07 |
| 29 | H08 | 22808 | MRAS | NM_012219 | 234856 | 235811 | 0.92 | 0.90 | -0.04 | 0.10 |
| 29 | H09 | 4361 | MRE11A | NM_005590 | 233739 | 245260 | 0.91 | 0.94 | 0.08 | -1.01 |
| 29 | H10 | 4485 | MST1 | NM_020998 | 229697 | 242015 | 0.90 | 0.93 | 0.62 | -1.04 |
| 29 | H11 | 66036 | MTMR9 | NM_015458 | 232028 | 226929 | 0.91 | 0.87 | -0.04 | 1.10 |
| 29 | H12 | 4594 | MUT | NM_000255 | 216406 | 229687 | 0.85 | 0.88 | 3.22 | 2.08 |
| 30 | A01 | NA | pos | NA | 151109 | 156228 | 0.58 | 0.61 | 15.41 | 16.57 |
| 30 | A02 | NA | NA | NA | 281693 | 317199 | 1.08 | 1.23 | -4.45 | -10.85 |
| 30 | A03 | 4600 | MX2 | NM_002463 | 279150 | 302742 | 1.07 | 1.17 | -1.29 | -6.94 |
| 30 | A04 | 10514 | MYBBP1A | NM_014520 | 262638 | 279756 | 1.01 | 1.08 | 0.40 | -2.60 |
| 30 | A05 | 55728 | N4BP2 | NM_018177 | 260355 | 267264 | 1.00 | 1.04 | -0.70 | -0.51 |
| 30 | A06 | 4735 | NEDD5 | NM_004404 | 257218 | 255007 | 0.99 | 0.99 | 0.09 | 1.03 |
| 30 | A07 | 57486 | NLN | NM_020726 | 249549 | 264106 | 0.96 | 1.02 | 0.06 | -2.14 |
| 30 | A08 | 9520 | NPEPPS | NM_006310 | 253077 | 246281 | 0.97 | 0.95 | -0.08 | 0.77 |
| 30 | A09 | 4893 | NRAS | NM_002524 | 250812 | 252256 | 0.96 | 0.98 | -0.33 | 0.17 |
| 30 | A10 | 4913 | NTHL1 | NM_002528 | 245861 | 243554 | 0.94 | 0.94 | -1.20 | 1.74 |
| 30 | A11 | 4938 | OAS1 | NM_002534 | 245058 | 246804 | 0.94 | 0.96 | 0.06 | -0.17 |
| 30 | A12 | 4939 | OAS2 | NM_002535 | 250164 | 242879 | 0.96 | 0.94 | 0.31 | 1.26 |
| 30 | B01 | NA | neg | NA | 305429 | 284261 | 1.17 | 1.10 | -9.57 | -7.72 |
| 30 | B02 | NA | neg | NA | 261164 | 255234 | 1.00 | 0.99 | -2.83 | -2.78 |
| 30 | B03 | 4940 | OAS3 | NM_006187 | 252534 | 243978 | 0.97 | 0.95 | 1.25 | 0.60 |
| 30 | B04 | 4946 | OAZ1 | NM_004152 | 257510 | 253534 | 0.99 | 0.98 | -0.32 | -0.61 |
| 30 | B05 | 4968 | OGG1 | NM_002542 | 248405 | 249011 | 0.95 | 0.96 | -0.38 | 0.12 |
| 30 | B06 | 8473 | OGT | NM_181672 | 229804 | 241053 | 0.88 | 0.93 | 2.76 | 0.92 |
| 30 | B07 | 5009 | OTC | NM_000531 | 234953 | 246381 | 0.90 | 0.95 | 0.78 | -1.60 |
| 30 | B08 | 5051 | PAFAH2 | NM_000437 | 249097 | 249436 | 0.95 | 0.97 | -0.98 | -2.25 |
| 30 | B09 | 5053 | PAH | NM_000277 | 249862 | 243842 | 0.96 | 0.94 | -1.69 | -0.88 |
| 30 | B10 | 80025 | PANK2 | NM_024960 | 246107 | 239802 | 0.94 | 0.93 | -2.74 | -0.10 |
| 30 | B11 | 5069 | PAPPA | NM_002581 | 233537 | 226104 | 0.89 | 0.88 | 0.31 | 0.88 |
| 30 | B12 | 5091 | PC | NM_000920 | 231446 | 233135 | 0.89 | 0.90 | 1.65 | 0.44 |

| | | | | | | | | | | |
|---|---|---|---|---|---|---|---|---|---|---|
| 30 | C01 | NA | pos | NA | 152793 | 147458 | 0.59 | 0.57 | 13.55 | 16.52 |
| 30 | C02 | NA | NA | NA | 260051 | 260268 | 1.00 | 1.01 | -2.76 | -2.70 |
| 30 | C03 | 5095 | PCCA | NM_000282 | 260606 | 253076 | 1.00 | 0.98 | -0.07 | -0.02 |
| 30 | C04 | 5108 | PCM1 | NM_006197 | 261198 | 254799 | 1.00 | 0.99 | -0.98 | 0.11 |
| 30 | C05 | 5111 | PCNA | NM_002592 | 242701 | 255645 | 0.93 | 0.99 | 0.38 | -0.08 |
| 30 | C06 | 5119 | PCOLN3 | NM_002768 | 239593 | 251703 | 0.92 | 0.98 | 1.17 | 0.04 |
| 30 | C07 | 5136 | PDE1A | NM_005019 | 243969 | 241124 | 0.93 | 0.93 | -0.69 | 0.23 |
| 30 | C08 | 5153 | PDE1B | NM_000924 | 204299 | 230143 | 0.78 | 0.89 | 5.74 | 1.97 |
| 30 | C09 | 5137 | PDE1C | NM_005020 | 219205 | 239394 | 0.84 | 0.93 | 2.87 | 0.81 |
| 30 | C10 | 8654 | PDE5A | NM_001083 | 226620 | 245527 | 0.87 | 0.95 | 0.12 | -0.14 |
| 30 | C11 | 5152 | PDE9A | NM_002606 | 239883 | 246171 | 0.92 | 0.95 | -0.75 | -1.61 |
| 30 | C12 | 5190 | PEX6 | NM_000287 | 243648 | 250086 | 0.93 | 0.97 | -0.31 | -1.51 |
| 30 | D01 | NA | neg | NA | 256386 | 277035 | 0.98 | 1.07 | -3.20 | -7.48 |
| 30 | D02 | NA | neg | NA | 261887 | 257726 | 1.00 | 1.00 | -4.04 | -4.19 |
| 30 | D03 | 5251 | PHEX | NM_000444 | 256211 | 255284 | 0.98 | 0.99 | -0.40 | -2.32 |
| 30 | D04 | 26227 | PHGDH | NM_006623 | 246085 | 235051 | 0.94 | 0.91 | 0.32 | 1.54 |
| 30 | D05 | 5300 | PIN1 | NM_006221 | 253607 | 251228 | 0.97 | 0.97 | -2.27 | -1.25 |
| 30 | D06 | 5319 | PLA2G1B | NM_000928 | 237423 | 243476 | 0.91 | 0.94 | 0.51 | -0.48 |
| 30 | D07 | 5320 | PLA2G2A | NM_000300 | 232343 | 230136 | 0.89 | 0.89 | 0.08 | 0.17 |
| 30 | D08 | 26279 | PLA2G2D | NM_012400 | 243075 | 230375 | 0.93 | 0.89 | -1.15 | 0.00 |
| 30 | D09 | 30814 | PLA2G2E | NM_014589 | 232094 | 231557 | 0.89 | 0.90 | -0.08 | 0.22 |
| 30 | D10 | 50487 | PLA2G3 | NM_015715 | 208990 | 232798 | 0.80 | 0.90 | 1.81 | 0.10 |
| 30 | D11 | 5322 | PLA2G5 | NM_000929 | 220206 | 225453 | 0.84 | 0.87 | 1.25 | -0.01 |
| 30 | D12 | 7941 | PLA2G7 | NM_005084 | 244190 | 241430 | 0.94 | 0.94 | -1.38 | -1.97 |
| 30 | E01 | NA | neg | NA | 280314 | 271744 | 1.07 | 1.05 | -5.28 | -6.15 |
| 30 | E02 | NA | neg | NA | 246777 | 253574 | 0.95 | 0.98 | -0.18 | -3.05 |
| 30 | E03 | 5340 | PLG | NM_000301 | 263367 | 239024 | 1.01 | 0.93 | 0.07 | 0.88 |
| 30 | E04 | 57026 | PDXP | NM_020315 | 260945 | 246047 | 1.00 | 0.95 | -0.38 | 0.11 |
| 30 | E05 | 5422 | POLA | NM_016937 | 265458 | 246003 | 1.02 | 0.95 | -2.51 | 0.08 |
| 30 | E06 | 5424 | POLD1 | NM_002691 | 257917 | 243462 | 0.99 | 0.94 | -1.05 | -0.04 |
| 30 | E07 | 5426 | POLE | NM_006231 | 243408 | 236075 | 0.93 | 0.91 | -0.04 | -0.40 |
| 30 | E08 | 5428 | POLG | NM_002693 | 251143 | 239914 | 0.96 | 0.93 | -0.82 | -1.18 |
| 30 | E09 | 5429 | POLH | NM_006502 | 232907 | 233453 | 0.89 | 0.90 | 1.36 | 0.34 |
| 30 | E10 | 27343 | POLL | NM_013274 | 209863 | 246948 | 0.80 | 0.96 | 3.24 | -1.87 |
| 30 | E11 | 5430 | POLR2A | NM_000937 | 175936 | 206989 | 0.67 | 0.80 | 9.54 | 3.58 |
| 30 | E12 | 5431 | POLR2B | NM_000938 | 230985 | 240989 | 0.88 | 0.93 | 2.19 | -1.45 |
| 30 | F01 | NA | NA | NA | 246652 | 264728 | 0.94 | 1.03 | -2.67 | -5.14 |
| 30 | F02 | NA | pos | NA | 140241 | 131871 | 0.54 | 0.51 | 13.51 | 17.50 |
| 30 | F03 | 5436 | POLR2G | NM_002696 | 248879 | 242999 | 0.95 | 0.94 | -0.24 | 0.02 |
| 30 | F04 | 11044 | POLS | NM_006999 | 235150 | 246217 | 0.90 | 0.95 | 1.03 | -0.11 |
| 30 | F05 | 10775 | POP4 | NM_006627 | 228989 | 241920 | 0.88 | 0.94 | 0.52 | 0.58 |
| 30 | F06 | 5464 | PP | NM_021129 | 235456 | 238379 | 0.90 | 0.92 | -0.15 | 0.63 |
| 30 | F07 | 56342 | PPAN | NM_020230 | 243844 | 233641 | 0.93 | 0.91 | -2.62 | -0.17 |
| 30 | F08 | 5471 | PPAT | NM_002703 | 227407 | 218922 | 0.87 | 0.85 | 0.28 | 2.21 |
| 30 | F09 | 8500 | PPFIA1 | NM_003626 | 224403 | 237882 | 0.86 | 0.92 | 0.14 | -0.61 |
| 30 | F10 | 8499 | PPFIA2 | NM_003625 | 208731 | 221940 | 0.80 | 0.86 | 0.90 | 2.20 |
| 30 | F11 | 8541 | PPFIA3 | NM_003660 | 228338 | 236624 | 0.87 | 0.92 | -0.94 | -1.66 |
| 30 | F12 | 8497 | PPFIA4 | XM_046751 | 230931 | 231395 | 0.88 | 0.90 | -0.32 | -0.01 |
| 30 | G01 | NA | neg | NA | 260966 | 255063 | 1.00 | 0.99 | -2.76 | -1.42 |
| 30 | G02 | NA | neg | NA | 258426 | 258493 | 0.99 | 1.00 | -2.38 | -2.01 |
| 30 | G03 | 5481 | PPID | NM_005038 | 252260 | 256675 | 0.97 | 0.99 | 1.34 | -0.24 |
| 30 | G04 | 9360 | PPIG | NM_004792 | 248121 | 251981 | 0.95 | 0.98 | 1.15 | 0.98 |
| 30 | G05 | 64840 | PORCN | NM_022825 | 235274 | 264037 | 0.90 | 1.02 | 1.65 | -1.12 |
| 30 | G06 | 5501 | PPP1CC | NM_002710 | 248775 | 255424 | 0.95 | 0.99 | -0.09 | -0.20 |
| 30 | G07 | 4659 | PPP1R12A | NM_002480 | 245325 | 240106 | 0.94 | 0.93 | -0.76 | 0.79 |
| 30 | G08 | 54776 | PPP1R12C | NM_017607 | 232551 | 244035 | 0.89 | 0.95 | 1.58 | 0.00 |
| 30 | G09 | 23368 | PPP1R13B | NM_015316 | 238502 | 247444 | 0.91 | 0.96 | 0.08 | -0.17 |
| 30 | G10 | 94274 | PPP1R14A | NM_033256 | 229118 | 233097 | 0.88 | 0.90 | -0.12 | 2.37 |
| 30 | G11 | 26472 | PPP1R14B | XM_370630 | 238870 | 232151 | 0.91 | 0.90 | -0.46 | 1.17 |
| 30 | G12 | 81706 | PPP1R14C | NM_030949 | 247299 | 243468 | 0.95 | 0.94 | -0.72 | 0.01 |
| 30 | H01 | NA | NA | NA | 293268 | 292068 | 1.12 | 1.13 | -7.78 | -8.76 |
| 30 | H02 | NA | pos | NA | 123088 | 137575 | 0.47 | 0.53 | 18.10 | 17.56 |
| 30 | H03 | 54866 | PPP1R14D | NM_017726 | 245817 | 247840 | 0.94 | 0.96 | 2.21 | 0.23 |
| 30 | H04 | 23645 | PPP1R15A | NM_014330 | 260804 | 257615 | 1.00 | 1.00 | -0.89 | -1.01 |
| 30 | H05 | 84919 | PPP1R15B | NM_032833 | 242323 | 244018 | 0.93 | 0.95 | 0.48 | 1.26 |
| 30 | H06 | 5502 | PPP1R1A | NM_006741 | 249124 | 257180 | 0.95 | 1.00 | -0.25 | -1.53 |
| 30 | H07 | 5504 | PPP1R2 | NM_006241 | 239377 | 236398 | 0.92 | 0.92 | 0.04 | 0.39 |
| 30 | H08 | 90673 | PPP1R3E | XM_033391 | 241718 | 238026 | 0.93 | 0.92 | 0.08 | -0.01 |
| 30 | H09 | 5534 | PPP3R1 | NM_000945 | 240474 | 243745 | 0.92 | 0.94 | -0.33 | -0.57 |
| 30 | H10 | 55370 | PPP4R1L | XM_086650 | 247352 | 242796 | 0.95 | 0.94 | -3.00 | -0.32 |
| 30 | H11 | 151987 | PPP4R2 | NM_174907 | 235542 | 232906 | 0.90 | 0.90 | -0.06 | 0.01 |
| 30 | H12 | 5558 | PRIM2A | NM_000947 | 224813 | 235392 | 0.86 | 0.91 | 2.59 | 0.35 |
| 31 | A01 | NA | pos | NA | 58197 | 40051 | 0.26 | 0.20 | 12.69 | 18.53 |
| 31 | A02 | NA | NA | NA | 237983 | 208064 | 1.05 | 1.06 | -2.51 | -2.37 |
| 31 | A03 | 5624 | PROC | NM_000312 | 255662 | 180876 | 1.13 | 0.93 | -3.39 | 0.11 |
| 31 | A04 | 8858 | PROZ | NM_003891 | 196934 | 156530 | 0.87 | 0.80 | 1.67 | 3.94 |
| 31 | A05 | 5644 | PRSS1 | NM_002769 | 253566 | 220767 | 1.12 | 1.13 | -4.08 | -2.55 |
| 31 | A06 | 5654 | PRSS11 | NM_002775 | 214122 | 201456 | 0.94 | 1.03 | -0.10 | 0.22 |
| 31 | A07 | 5645 | PRSS2 | NM_002770 | 193772 | 189622 | 0.85 | 0.97 | 1.30 | 0.04 |
| 31 | A08 | 27429 | PRSS25 | NM_013247 | 196615 | 203620 | 0.87 | 1.04 | 0.34 | -2.20 |
| 31 | A09 | 5682 | PSMA1 | NM_002786 | 188209 | 184965 | 0.83 | 0.95 | 1.62 | -0.02 |
| 31 | A10 | 5831 | PYCR1 | NM_006907 | 196995 | 175810 | 0.87 | 0.90 | 0.10 | 1.77 |
| 31 | A11 | 5873 | RAB27A | NM_004580 | 203372 | 195457 | 0.90 | 1.00 | -0.43 | -1.85 |
| 31 | A12 | 5874 | RAB27B | NM_004163 | 224447 | 204339 | 0.99 | 1.05 | -1.13 | -1.36 |
| 31 | B01 | NA | neg | NA | 235587 | 195629 | 1.04 | 1.00 | -0.46 | 1.04 |
| 31 | B02 | NA | neg | NA | 218138 | 195393 | 0.96 | 1.00 | 1.02 | 1.07 |
| 31 | B03 | 9364 | RAB28 | NM_004249 | 234801 | 173876 | 1.03 | 0.89 | 0.22 | 2.84 |
| 31 | B04 | 27314 | RAB30 | NM_014488 | 224094 | 202101 | 0.99 | 1.03 | 1.22 | 0.13 |

| | | | | | | | | | | |
|---|---|---|---|---|---|---|---|---|---|---|
| 31 | B05 | 84084 | RAB6C | NM_032144 | 223395 | 204014 | 0.98 | 1.04 | 0.32 | 1.40 |
| 31 | B06 | 11159 | RABL2A | NM_007082 | 234867 | 225236 | 1.04 | 1.15 | 0.00 | -0.87 |
| 31 | B07 | 11158 | RABL2B | NM_007081 | 230626 | 201570 | 1.02 | 1.03 | 0.04 | 0.41 |
| 31 | B08 | 5880 | RAC2 | NM_002872 | 234516 | 201336 | 1.03 | 1.03 | -1.01 | -0.06 |
| 31 | B09 | 5810 | RAD1 | NM_002853 | 229211 | 204133 | 1.01 | 1.04 | 0.00 | -0.54 |
| 31 | B10 | 10111 | RAD50 | NM_005732 | 236626 | 204690 | 1.04 | 1.05 | -1.41 | 0.04 |
| 31 | B11 | 5889 | RAD51C | NM_002876 | 242097 | 213670 | 1.07 | 1.09 | -1.86 | -2.25 |
| 31 | B12 | 5890 | RAD51L1 | NM_002877 | 234036 | 209704 | 1.03 | 1.07 | -0.09 | -0.17 |
| 31 | C01 | NA | pos | NA | 53819 | 45283 | 0.24 | 0.23 | 13.72 | 18.72 |
| 31 | C02 | NA | NA | NA | 209209 | 210779 | 0.92 | 1.08 | 0.59 | -1.86 |
| 31 | C03 | 5892 | RAD51L3 | NM_002878 | 209837 | 209432 | 0.92 | 1.07 | 1.15 | -2.60 |
| 31 | C04 | 25788 | RAD54B | NM_012415 | 213752 | 198319 | 0.94 | 1.01 | 0.91 | -0.41 |
| 31 | C05 | 5897 | RAG2 | NM_000536 | 213431 | 194368 | 0.94 | 0.99 | -0.02 | 1.58 |
| 31 | C06 | 5903 | RANBP2 | NM_006267 | 220891 | 208585 | 0.97 | 1.07 | 0.00 | 0.18 |
| 31 | C07 | 5906 | RAP1A | NM_002884 | 190634 | 196981 | 0.84 | 1.01 | 2.24 | -0.04 |
| 31 | C08 | 5911 | RAP2A | NM_021033 | 212546 | 195961 | 0.94 | 1.00 | -0.34 | -0.41 |
| 31 | C09 | 5949 | RBP3 | NM_002900 | 217661 | 177208 | 0.96 | 0.91 | -0.21 | 1.79 |
| 31 | C10 | 9401 | RECQL4 | NM_004260 | 189522 | 163341 | 0.84 | 0.84 | 1.39 | 4.17 |
| 31 | C11 | 9400 | RECQL5 | NM_004259 | 206139 | 186991 | 0.91 | 0.96 | 0.00 | 0.05 |
| 31 | C12 | 5976 | RENT1 | NM_002911 | 232866 | 218452 | 1.03 | 1.12 | -1.17 | -2.27 |
| 31 | D01 | NA | neg | NA | 253703 | 176492 | 1.12 | 0.90 | -3.88 | 3.40 |
| 31 | D02 | NA | neg | NA | 207390 | 203037 | 0.91 | 1.04 | 0.04 | 0.09 |
| 31 | D03 | 51455 | REV1L | NM_016316 | 219131 | 188993 | 0.97 | 0.97 | -0.34 | 0.94 |
| 31 | D04 | 5980 | REV3L | NM_002912 | 216572 | 204076 | 0.95 | 1.04 | -0.03 | -0.13 |
| 31 | D05 | 27289 | RND1 | NM_014470 | 177513 | 227785 | 0.78 | 1.17 | 2.31 | -1.58 |
| 31 | D06 | 22836 | RHOBTB3 | NM_014899 | 192680 | 220295 | 0.85 | 1.13 | 1.68 | -0.28 |
| 31 | D07 | 6014 | RIT2 | NM_002930 | 196207 | 195586 | 0.86 | 1.00 | 1.06 | 1.13 |
| 31 | D08 | 8635 | RNASET2 | NM_003730 | 208446 | 197049 | 0.92 | 1.01 | -0.70 | 0.46 |
| 31 | D09 | 8731 | RNMT | NM_003799 | NA | 209538 | NA | 1.07 | NA | -1.23 |
| 31 | D10 | 6051 | RNPEP | NM_020216 | 198794 | 205381 | 0.88 | 1.05 | -0.10 | -0.06 |
| 31 | D11 | 6236 | RRAD | NM_004165 | 197795 | 194951 | 0.87 | 1.00 | 0.00 | 0.05 |
| 31 | D12 | 6237 | RRAS | NM_006270 | 202978 | 206834 | 0.89 | 1.06 | 0.65 | 0.17 |
| 31 | E01 | NA | neg | NA | 254335 | 181115 | 1.12 | 0.93 | -4.41 | 1.57 |
| 31 | E02 | NA | neg | NA | 214619 | 224909 | 0.95 | 1.15 | -1.06 | -3.88 |
| 31 | E03 | 22800 | RRAS2 | NM_012250 | 195124 | 188606 | 0.86 | 0.96 | 1.21 | -0.26 |
| 31 | E04 | 10633 | RRP22 | NM_006477 | 233937 | 189845 | 1.03 | 0.97 | -1.98 | 0.39 |
| 31 | E05 | 8607 | RUVBL1 | NM_003707 | 196059 | 205239 | 0.86 | 1.05 | 0.26 | -0.02 |
| 31 | E06 | 10856 | RUVBL2 | NM_006666 | 177006 | 193381 | 0.78 | 0.99 | 2.52 | 1.82 |
| 31 | E07 | 6319 | SCD | NM_005063 | 206183 | 189657 | 0.91 | 0.97 | -0.26 | 0.62 |
| 31 | E08 | 83852 | SETDB2 | NM_031915 | 172123 | 183275 | 0.76 | 0.94 | 1.89 | 0.92 |
| 31 | E09 | 6419 | SETMAR | NM_006515 | 162470 | 189463 | 0.72 | 0.97 | 3.27 | 0.02 |
| 31 | E10 | 6456 | SH3GL2 | NM_003026 | 198164 | 217175 | 0.87 | 1.11 | -0.52 | -2.78 |
| 31 | E11 | 7979 | SHFM1 | NM_006304 | 209310 | 195920 | 0.92 | 1.00 | -1.45 | -1.32 |
| 31 | E12 | 23411 | SIRT1 | NM_012238 | 208478 | 203366 | 0.92 | 1.04 | -0.30 | -0.65 |
| 31 | F01 | NA | NA | NA | 245164 | 187555 | 1.08 | 0.96 | -4.03 | 1.05 |
| 31 | F02 | NA | pos | NA | 62275 | 70757 | 0.27 | 0.36 | 11.43 | 15.58 |
| 31 | F03 | 22933 | SIRT2 | NM_012237 | 207391 | 190487 | 0.91 | 0.97 | -0.22 | -0.22 |
| 31 | F04 | 56916 | SMARCAD1 | NM_020159 | 214133 | 206160 | 0.94 | 1.05 | -0.70 | -1.36 |
| 31 | F05 | 50485 | SMARCAL1 | NM_014140 | 194347 | 207010 | 0.86 | 1.06 | 0.02 | 0.03 |
| 31 | F06 | 6648 | SOD2 | NM_000636 | 202430 | 211720 | 0.89 | 1.08 | -0.02 | -0.18 |
| 31 | F07 | 6677 | SPAM1 | NM_003117 | 198899 | 205236 | 0.88 | 1.05 | -0.04 | -1.04 |
| 31 | F08 | 51062 | SPG3A | NM_015915 | 181902 | 192437 | 0.80 | 0.98 | 0.67 | 0.06 |
| 31 | F09 | 6687 | SPG7 | NM_003119 | 188851 | 172909 | 0.83 | 0.88 | 0.65 | 2.35 |
| 31 | F10 | 23626 | SPO11 | NM_012444 | 196288 | 195590 | 0.87 | 1.00 | -0.76 | 0.18 |
| 31 | F11 | 6783 | SULT1E1 | NM_005420 | 177991 | 187950 | 0.78 | 0.96 | 0.80 | -0.05 |
| 31 | F12 | 412 | STS | NM_000351 | 180376 | 179500 | 0.80 | 0.92 | 1.69 | 2.60 |
| 31 | G01 | NA | neg | NA | 250441 | 180942 | 1.10 | 0.93 | -4.66 | 0.26 |
| 31 | G02 | NA | neg | NA | 211441 | 201072 | 0.93 | 1.03 | -1.36 | -2.24 |
| 31 | G03 | 6821 | SUOX | NM_000456 | 191443 | 170410 | 0.84 | 0.87 | 0.94 | 0.67 |
| 31 | G04 | 6839 | SUV39H1 | NM_003173 | 203320 | 178237 | 0.90 | 0.91 | 0.03 | 0.50 |
| 31 | G05 | 79723 | SUV39H2 | NM_024670 | 204290 | 233560 | 0.90 | 1.19 | -1.01 | -4.88 |
| 31 | G06 | 8867 | SYNJ1 | NM_003895 | 206052 | 194823 | 0.91 | 1.00 | -0.51 | 0.31 |
| 31 | G07 | 6894 | TARBP1 | NM_005646 | 205075 | 207506 | 0.90 | 1.06 | -0.74 | -2.93 |
| 31 | G08 | 6898 | TAT | NM_000353 | 209175 | 186005 | 0.92 | 0.95 | -1.82 | -0.75 |
| 31 | G09 | 10728 | TEBP | NM_006601 | 194769 | 193717 | 0.86 | 0.99 | -0.03 | -1.85 |
| 31 | G10 | 7011 | TEP1 | NM_007110 | 182887 | 186645 | 0.81 | 0.95 | 0.19 | -0.32 |
| 31 | G11 | 7015 | TERT | NM_003219 | 175525 | 167299 | 0.77 | 0.86 | 0.83 | 0.91 |
| 31 | G12 | 7051 | TGM1 | NM_000359 | 197078 | 184312 | 0.87 | 0.94 | 0.09 | 0.39 |
| 31 | H01 | NA | NA | NA | 238680 | 177460 | 1.05 | 0.91 | -0.49 | 4.26 |
| 31 | H02 | NA | pos | NA | 65014 | 61985 | 0.29 | 0.32 | 14.19 | 18.63 |
| 31 | H03 | 7052 | TGM2 | NM_004613 | 266933 | 205331 | 1.18 | 1.05 | -2.27 | -0.11 |
| 31 | H04 | 7054 | TH | NM_000360 | 266889 | 257284 | 1.18 | 1.32 | -2.17 | -5.76 |
| 31 | H05 | 7086 | TKT | NM_001064 | 255294 | 222836 | 1.13 | 1.14 | -2.15 | 0.02 |
| 31 | H06 | 8277 | TKTL1 | NM_012253 | 216595 | 227895 | 0.95 | 1.17 | 1.77 | -0.23 |
| 31 | H07 | 7092 | TLL1 | NM_012464 | 245979 | 237731 | 1.08 | 1.22 | -1.03 | -3.12 |
| 31 | H08 | 7145 | TNS | NM_022648 | 206422 | 198656 | 0.91 | 1.02 | 1.59 | 1.24 |
| 31 | H09 | 116447 | TOP1MT | NM_052963 | 236761 | 202756 | 1.04 | 1.04 | -0.41 | 0.60 |
| 31 | H10 | 7155 | TOP2B | NM_001068 | 217864 | 213154 | 0.96 | 1.09 | 0.41 | -0.04 |
| 31 | H11 | 7156 | TOP3A | NM_004618 | 212417 | 197028 | 0.94 | 1.01 | 0.88 | 0.78 |
| 31 | H12 | 8940 | TOP3B | NM_003935 | 220611 | 205001 | 0.97 | 1.05 | 1.27 | 1.38 |
| 32 | A01 | NA | pos | NA | 56624 | 54349 | 0.26 | 0.23 | 19.21 | 13.75 |
| 32 | A02 | NA | NA | NA | 200990 | 231380 | 0.92 | 0.97 | 0.23 | -2.38 |
| 32 | A03 | 11073 | TOPBP1 | NM_007027 | 203371 | 268280 | 0.93 | 1.12 | 0.04 | -4.76 |
| 32 | A04 | 11277 | TREX1 | NM_032166 | 206582 | 194865 | 0.94 | 0.82 | -0.43 | 1.16 |
| 32 | A05 | 11219 | TREX2 | NM_007205 | 219700 | 201258 | 1.00 | 0.84 | -2.46 | 0.20 |
| 32 | A06 | 64282 | PAPD5 | NM_022447 | 199642 | 220655 | 0.91 | 0.92 | 0.19 | -1.23 |
| 32 | A07 | 51095 | TRNT1 | NM_016000 | 211630 | 233954 | 0.97 | 0.98 | -0.60 | -2.77 |
| 32 | A08 | 23020 | U5-200KD | NM_014014 | 178424 | 194640 | 0.81 | 0.81 | 3.15 | 0.15 |

| | | | | | | | | | | |
|---|---|---|---|---|---|---|---|---|---|---|
| 32 | A09 | 7332 | UBE2L3 | NM_003347 | 192730 | 195847 | 0.88 | 0.82 | 0.31 | 0.18 |
| 32 | A10 | 7453 | WARS | NM_004184 | 186632 | 203190 | 0.85 | 0.85 | 2.61 | 0.23 |
| 32 | A11 | 7486 | WRN | NM_000553 | 204307 | 206612 | 0.93 | 0.86 | -0.48 | -0.16 |
| 32 | A12 | 7512 | XPNPEP2 | NM_003399 | 210834 | 224080 | 0.96 | 0.94 | -0.05 | -1.61 |
| 32 | B01 | NA | neg | NA | 236657 | 270326 | 1.08 | 1.13 | -3.73 | -4.81 |
| 32 | B02 | NA | neg | NA | 209161 | 236295 | 0.95 | 0.99 | -0.11 | -1.71 |
| 32 | B03 | 7517 | XRCC3 | NM_005432 | 194903 | 185001 | 0.89 | 0.77 | 1.89 | 3.95 |
| 32 | B04 | 10730 | YME1L1 | NM_014263 | 200834 | 201562 | 0.92 | 0.84 | 1.06 | 1.67 |
| 32 | B05 | 84914 | ZNF587 | NM_032828 | 195463 | 212706 | 0.89 | 0.89 | 1.46 | 0.28 |
| 32 | B06 | 203068 | OK/SW-CL.56 | NM_178014 | 212467 | 224933 | 0.97 | 0.94 | -0.76 | -0.50 |
| 32 | B07 | 7280 | TUBB | NM_001069 | 203860 | 215073 | 0.93 | 0.90 | 1.16 | 0.07 |
| 32 | B08 | 10382 | TUBB5 | NM_006087 | 210000 | 209997 | 0.96 | 0.88 | -0.27 | -0.13 |
| 32 | B09 | 11095 | ADAMTS8 | NM_007037 | 205170 | 208973 | 0.94 | 0.87 | -0.59 | 0.11 |
| 32 | B10 | 27032 | ATP2C1 | NM_014382 | 209780 | 219175 | 0.96 | 0.92 | 0.30 | -0.10 |
| 32 | B11 | 3756 | KCNH1 | NM_002238 | 220904 | 223374 | 1.01 | 0.93 | -1.93 | -0.57 |
| 32 | B12 | 27133 | KCNH5 | NM_139318 | 224211 | 219648 | 1.02 | 0.92 | -1.08 | -0.08 |
| 32 | C01 | NA | pos | NA | 58379 | 73178 | 0.27 | 0.31 | 17.82 | 12.17 |
| 32 | C02 | NA | NA | NA | 206980 | 249239 | 0.94 | 1.04 | -1.72 | -3.87 |
| 32 | C03 | 6891 | TAP2 | NM_000544 | 190016 | 216624 | 0.87 | 0.91 | 0.63 | 0.08 |
| 32 | C04 | 6892 | TAPBP | NM_172208 | 170603 | 209863 | 0.78 | 0.88 | 3.13 | -0.08 |
| 32 | C05 | 81792 | ADAMTS12 | NM_030955 | 189637 | 244008 | 0.87 | 1.02 | 0.33 | -3.56 |
| 32 | C06 | 10376 | K-ALPHA-1 | NM_006082 | 156368 | 162317 | 0.71 | 0.68 | 4.72 | 4.21 |
| 32 | C07 | 84617 | MGC4083 | NM_032525 | 189432 | 192619 | 0.86 | 0.81 | 1.16 | 1.13 |
| 32 | C08 | 4745 | NELL1 | NM_006157 | 215248 | 225798 | 0.98 | 0.94 | -2.86 | -2.55 |
| 32 | C09 | 5413 | PNUTL1 | NM_002688 | 204605 | 207107 | 0.93 | 0.87 | -2.41 | -0.71 |
| 32 | C10 | 5414 | PNUTL2 | NM_004574 | 199878 | 206060 | 0.91 | 0.86 | -0.30 | 0.10 |
| 32 | C11 | 5771 | PTPN2 | NM_002828 | 195040 | 199263 | 0.89 | 0.83 | -0.43 | 0.64 |
| 32 | C12 | 5649 | RELN | NM_005045 | 228292 | 227946 | 1.04 | 0.95 | -3.51 | -1.83 |
| 32 | D01 | NA | neg | NA | 249358 | 271615 | 1.14 | 1.14 | -5.90 | -5.99 |
| 32 | D02 | NA | neg | NA | 214795 | 207599 | 0.98 | 0.87 | -1.36 | -0.16 |
| 32 | D03 | 58988 | RPS6KB2 | NM_021485 | 202722 | 217479 | 0.93 | 0.91 | 0.36 | -0.08 |
| 32 | D04 | 7277 | TUBA1 | NM_006000 | 182522 | 198783 | 0.83 | 0.83 | 2.96 | 0.85 |
| 32 | D05 | 7278 | TUBA2 | NM_006001 | 203048 | 187470 | 0.93 | 0.78 | -0.04 | 1.51 |
| 32 | D06 | 81027 | TUBB1 | NM_030773 | 205063 | 195947 | 0.94 | 0.82 | -0.29 | 1.07 |
| 32 | D07 | 10381 | TUBB4 | NM_006086 | 185427 | 201704 | 0.85 | 0.84 | 3.08 | 0.22 |
| 32 | D08 | 56604 | TUBB4Q | NM_020040 | 211591 | 237658 | 0.97 | 0.99 | -0.98 | -3.71 |
| 32 | D09 | 51174 | TUBD1 | NM_016261 | 205067 | 224552 | 0.94 | 0.94 | -1.08 | -2.38 |
| 32 | D10 | 51175 | TUBE1 | NM_016262 | 215901 | 223078 | 0.99 | 0.93 | -1.01 | -1.53 |
| 32 | D11 | 7283 | TUBG1 | NM_001070 | 201813 | 205617 | 0.92 | 0.86 | 0.08 | -0.02 |
| 32 | D12 | 9429 | ABCG2 | NM_004827 | 211521 | 206789 | 0.97 | 0.87 | 0.09 | 0.02 |
| 32 | E01 | NA | neg | NA | 222398 | 241594 | 1.02 | 1.01 | -2.80 | -2.98 |
| 32 | E02 | NA | neg | NA | 215736 | 216323 | 0.98 | 0.91 | -1.93 | -0.68 |
| 32 | E03 | 130 | ADH6 | NM_000672 | 202370 | 203806 | 0.92 | 0.85 | -0.04 | 1.44 |
| 32 | E04 | 131 | ADH7 | NM_000673 | 214295 | 211658 | 0.98 | 0.89 | -1.67 | -0.05 |
| 32 | E05 | 79572 | AFURS1 | NM_024524 | 182893 | 208928 | 0.83 | 0.87 | 2.16 | -0.17 |
| 32 | E06 | 1645 | AKR1C1 | NM_001353 | 182565 | 212810 | 0.83 | 0.89 | 2.22 | -0.19 |
| 32 | E07 | 1646 | AKR1C2 | NM_001354 | 208499 | 169025 | 0.95 | 0.71 | -0.40 | 3.47 |
| 32 | E08 | 242 | ALOX12B | NM_001139 | 194667 | 194308 | 0.89 | 0.81 | 0.80 | 0.51 |
| 32 | E09 | 247 | ALOX15B | NM_001141 | 193277 | 201123 | 0.88 | 0.84 | 0.02 | 0.03 |
| 32 | E10 | 316 | AOX1 | NM_001159 | 209690 | 216270 | 0.96 | 0.91 | -0.64 | -0.63 |
| 32 | E11 | 23200 | ATP11B | XM_087254 | 192395 | 214560 | 0.88 | 0.90 | 0.87 | -0.56 |
| 32 | E12 | 477 | ATP1A2 | NM_000702 | 210712 | 204371 | 0.96 | 0.86 | -0.25 | 0.51 |
| 32 | F01 | NA | NA | NA | 258454 | 255250 | 1.18 | 1.07 | -8.10 | -4.88 |
| 32 | F02 | NA | pos | NA | 66600 | 73142 | 0.30 | 0.31 | 17.13 | 11.71 |
| 32 | F03 | 487 | ATP2A1 | NM_004320 | 202833 | 196896 | 0.93 | 0.82 | -0.66 | 1.42 |
| 32 | F04 | 489 | ATP2A3 | NM_005173 | 194130 | 207117 | 0.89 | 0.87 | 0.43 | -0.29 |
| 32 | F05 | 525 | ATP6V1B1 | NM_001692 | 194870 | 198036 | 0.89 | 0.83 | 0.03 | 0.17 |
| 32 | F06 | 51606 | ATP6V1H | NM_015941 | 196697 | 196229 | 0.90 | 0.82 | -0.19 | 0.66 |
| 32 | F07 | 538 | ATP7A | NM_000052 | 198534 | 210121 | 0.91 | 0.88 | 0.36 | -0.93 |
| 32 | F08 | 540 | ATP7B | NM_000053 | 195669 | 183428 | 0.89 | 0.77 | 0.11 | 0.85 |
| 32 | F09 | 10396 | ATP8A1 | NM_006095 | 189427 | 202258 | 0.86 | 0.85 | -0.02 | -0.73 |
| 32 | F10 | 51761 | ATP8A2 | NM_016529 | 204443 | 223149 | 0.93 | 0.93 | -0.50 | -1.91 |
| 32 | F11 | 5205 | ATP8B1 | NM_005603 | 195396 | 201036 | 0.89 | 0.84 | -0.08 | 0.02 |
| 32 | F12 | 10079 | ATP9A | XM_030577 | 204168 | 203021 | 0.93 | 0.85 | 0.05 | -0.02 |
| 32 | G01 | NA | neg | NA | 224141 | 270569 | 1.02 | 1.13 | -3.50 | -6.77 |
| 32 | G02 | NA | neg | NA | 198994 | 229087 | 0.91 | 0.96 | -0.19 | -2.99 |
| 32 | G03 | 9973 | CCS | NM_005125 | 204462 | 208527 | 0.93 | 0.87 | -0.78 | -0.13 |
| 32 | G04 | 1080 | CFTR | NM_000492 | 206623 | 198069 | 0.94 | 0.83 | -1.12 | 0.05 |
| 32 | G05 | 57130 | ATP13A | NM_020410 | 211655 | 209069 | 0.97 | 0.87 | -2.09 | -1.33 |
| 32 | G06 | 1353 | COX11 | NM_004375 | 190212 | 211171 | 0.87 | 0.88 | 0.75 | -1.19 |
| 32 | G07 | 1356 | CP | NM_000096 | 204616 | 195336 | 0.93 | 0.82 | -0.36 | -0.07 |
| 32 | G08 | 8895 | CPNE3 | NM_003909 | 198080 | 185940 | 0.90 | 0.78 | -0.11 | 0.13 |
| 32 | G09 | 1588 | CYP19A1 | NM_000103 | 180389 | 189171 | 0.82 | 0.79 | 1.26 | -0.03 |
| 32 | G10 | 1545 | CYP1B1 | NM_000104 | 181551 | 190463 | 0.83 | 0.80 | 2.60 | 0.57 |
| 32 | G11 | 1659 | DHX8 | NM_004941 | 172907 | 169722 | 0.79 | 0.71 | 2.97 | 2.38 |
| 32 | G12 | 1738 | DLD | NM_000108 | 204190 | 197248 | 0.93 | 0.83 | 0.14 | 0.02 |
| 32 | H01 | NA | NA | NA | 257430 | 244053 | 1.18 | 1.02 | -4.94 | -0.94 |
| 32 | H02 | NA | pos | NA | 73771 | 71316 | 0.34 | 0.30 | 19.21 | 14.79 |
| 32 | H03 | 1806 | DPYD | NM_000110 | 265166 | 256502 | 1.21 | 1.07 | -5.84 | -1.10 |
| 32 | H04 | 10146 | G3BP | NM_005754 | 283746 | 268241 | 1.30 | 1.12 | -8.33 | -2.94 |
| 32 | H05 | 23400 | HSA9947 | NM_022089 | 218315 | 255844 | 1.00 | 1.07 | -0.03 | -2.18 |
| 32 | H06 | 3295 | HSD17B4 | NM_000414 | 249492 | 233427 | 1.14 | 0.98 | -4.12 | 0.19 |
| 32 | H07 | 9914 | KIAA0703 | NM_014861 | 268995 | 259110 | 1.23 | 1.08 | -5.89 | -2.47 |
| 32 | H08 | 3990 | LIPC | NM_000236 | 215053 | 227191 | 0.98 | 0.95 | 0.58 | -0.22 |
| 32 | H09 | 4023 | LPL | NM_000237 | 206080 | 199632 | 0.94 | 0.84 | 0.80 | 2.43 |
| 32 | H10 | 4687 | NCF1 | NM_000265 | 206796 | 217055 | 0.94 | 0.91 | 2.20 | 1.56 |
| 32 | H11 | 4719 | NDUFS1 | NM_005006 | 209023 | 207674 | 0.95 | 0.87 | 1.15 | 2.33 |
| 32 | H12 | 4843 | NOS2A | NM_000625 | 227127 | 219297 | 1.04 | 0.92 | 0.05 | 1.42 |

| | | | | | | | | | | |
|---|---|---|---|---|---|---|---|---|---|---|
| 33 | A01 | NA | pos | NA | 55550 | 63249 | 0.23 | 0.27 | 13.39 | 12.64 |
| 33 | A02 | NA | NA | NA | 202089 | 212227 | 0.83 | 0.89 | -0.33 | -0.80 |
| 33 | A03 | 79400 | NOX5 | NM_024505 | 200113 | 211290 | 0.83 | 0.89 | 0.02 | 0.00 |
| 33 | A04 | 8050 | PDHX | NM_003477 | 179995 | 131913 | 0.74 | 0.56 | 2.12 | 7.55 |
| 33 | A05 | 5730 | PTGDS | NM_000954 | 208600 | 197407 | 0.86 | 0.83 | 0.23 | 0.58 |
| 33 | A06 | 9543 | PUNC | NM_004884 | 186797 | 184155 | 0.77 | 0.77 | 1.37 | 1.46 |
| 33 | A07 | 8766 | RAB11A | NM_004663 | 212481 | 215691 | 0.88 | 0.91 | -1.63 | -0.90 |
| 33 | A08 | 9230 | RAB11B | NM_004218 | 197906 | 203003 | 0.82 | 0.85 | -0.63 | 0.00 |
| 33 | A09 | 5872 | RAB13 | NM_002870 | 188537 | 190586 | 0.78 | 0.80 | -0.12 | 0.15 |
| 33 | A10 | 22931 | RAB18 | NM_021252 | 188657 | 202786 | 0.78 | 0.85 | 0.09 | -1.58 |
| 33 | A11 | 5861 | RAB1A | NM_004161 | 197017 | 209388 | 0.81 | 0.88 | -0.02 | -1.10 |
| 33 | A12 | 9609 | RAB36 | NM_004914 | 223861 | 229315 | 0.92 | 0.96 | -1.53 | -1.65 |
| 33 | B01 | NA | neg | NA | 260017 | 273256 | 1.07 | 1.15 | -5.85 | -6.69 |
| 33 | B02 | NA | neg | NA | 212691 | 219108 | 0.88 | 0.92 | -1.42 | -1.80 |
| 33 | B03 | 23682 | RAB38 | NM_022337 | 193400 | 189910 | 0.80 | 0.80 | 0.55 | 1.54 |
| 33 | B04 | 5864 | RAB3A | NM_002866 | 201751 | 188604 | 0.83 | 0.79 | -0.01 | 2.05 |
| 33 | B05 | 5865 | RAB3B | NM_002867 | 182374 | 189529 | 0.75 | 0.80 | 2.59 | 0.90 |
| 33 | B06 | 5868 | RAB5A | NM_004162 | 190357 | 195520 | 0.79 | 0.82 | 0.94 | 0.05 |
| 33 | B07 | 5878 | RAB5C | NM_004583 | 192273 | 201298 | 0.79 | 0.85 | 0.17 | 0.01 |
| 33 | B08 | 5870 | RAB6A | NM_002869 | 206789 | 198864 | 0.85 | 0.84 | -1.55 | -0.01 |
| 33 | B09 | 51560 | RAB6B | NM_016577 | 193475 | 196006 | 0.80 | 0.82 | -0.68 | -0.72 |
| 33 | B10 | 7879 | RAB7 | NM_004637 | 210696 | 211737 | 0.87 | 0.89 | -2.07 | -2.78 |
| 33 | B11 | 8934 | RAB7L1 | NM_003929 | 200542 | 209582 | 0.83 | 0.88 | -0.45 | -1.50 |
| 33 | B12 | 9367 | RAB9A | NM_004251 | 206322 | 208381 | 0.85 | 0.88 | 0.02 | -0.14 |
| 33 | C01 | NA | pos | NA | 61344 | 58023 | 0.25 | 0.24 | 13.21 | 12.02 |
| 33 | C02 | NA | NA | NA | 212764 | 222724 | 0.88 | 0.94 | -0.97 | -2.83 |
| 33 | C03 | 5901 | RAN | NM_006325 | 192105 | 185420 | 0.79 | 0.78 | 1.13 | 1.24 |
| 33 | C04 | 6390 | SDHB | NM_003000 | 225901 | 230957 | 0.93 | 0.97 | -1.82 | -2.48 |
| 33 | C05 | 23071 | TXNDC4 | XM_088476 | 220678 | 174038 | 0.91 | 0.73 | -0.54 | 1.59 |
| 33 | C06 | 51741 | WWOX | NM_016373 | 204115 | 187725 | 0.84 | 0.79 | 0.11 | 0.04 |
| 33 | C07 | 847 | CAT | NM_001752 | 200093 | 200267 | 0.83 | 0.84 | -0.11 | -0.60 |
| 33 | C08 | 8288 | EPX | NM_000502 | 173363 | 190937 | 0.72 | 0.80 | 2.03 | 0.00 |
| 33 | C09 | 2877 | GPX2 | NM_002083 | 197655 | 180166 | 0.82 | 0.76 | -0.62 | 0.00 |
| 33 | C10 | 2954 | GSTZ1 | NM_001513 | 189628 | 167021 | 0.78 | 0.70 | 0.36 | 0.55 |
| 33 | C11 | 5052 | PRDX1 | NM_002574 | 181612 | 197228 | 0.75 | 0.83 | 1.78 | -1.09 |
| 33 | C12 | 7001 | PRDX2 | NM_005809 | 215498 | 219279 | 0.89 | 0.92 | -0.39 | -1.83 |
| 33 | D01 | NA | neg | NA | 241978 | 269168 | 1.00 | 1.13 | -3.56 | -5.72 |
| 33 | D02 | NA | neg | NA | 210277 | 214981 | 0.87 | 0.90 | -0.59 | -0.84 |
| 33 | D03 | 10935 | PRDX3 | NM_006793 | 205870 | 211920 | 0.85 | 0.89 | -0.02 | 0.15 |
| 33 | D04 | 10549 | PRDX4 | NM_006406 | 207957 | 216997 | 0.86 | 0.91 | 0.01 | 0.08 |
| 33 | D05 | 9588 | PRDX6 | NM_004905 | 217533 | 218595 | 0.90 | 0.92 | -0.09 | -1.13 |
| 33 | D06 | 5742 | PTGS1 | NM_000962 | 202983 | 172955 | 0.84 | 0.73 | 0.36 | 2.68 |
| 33 | D07 | 5743 | PTGS2 | NM_000963 | 193660 | 214639 | 0.80 | 0.90 | 0.64 | -0.60 |
| 33 | D08 | 10587 | TXNRD2 | NM_006440 | 189945 | 201527 | 0.78 | 0.85 | 0.63 | 0.34 |
| 33 | D09 | 7837 | D2S448 | XM_056455 | 204946 | 195476 | 0.85 | 0.82 | -1.15 | -0.08 |
| 33 | D10 | 50506 | DUOX2 | NM_014080 | 212309 | 198316 | 0.88 | 0.83 | -1.62 | -0.97 |
| 33 | D11 | 4353 | MPO | NM_000250 | 202544 | 193840 | 0.84 | 0.82 | -0.03 | 0.51 |
| 33 | D12 | 6649 | SOD3 | NM_003102 | 210005 | 226116 | 0.87 | 0.95 | 0.27 | -1.15 |
| 33 | E01 | NA | neg | NA | 247281 | 218802 | 1.02 | 0.92 | -6.33 | -1.87 |
| 33 | E02 | NA | neg | NA | 218790 | 231926 | 0.90 | 0.98 | -3.66 | -3.05 |
| 33 | E03 | 7173 | TPO | NM_000547 | 202668 | 212145 | 0.84 | 0.89 | -1.99 | -0.56 |
| 33 | E04 | 306 | ANXA3 | NM_005139 | 215788 | 212572 | 0.89 | 0.89 | -2.99 | -0.20 |
| 33 | E05 | 842 | CASP9 | NM_001229 | 182501 | 208687 | 0.75 | 0.88 | 0.91 | -0.92 |
| 33 | E06 | 2159 | F10 | NM_000504 | 183743 | 195497 | 0.76 | 0.82 | -0.11 | -0.04 |
| 33 | E07 | 4324 | MMP15 | NM_002428 | 175043 | 176550 | 0.72 | 0.74 | 0.11 | 2.15 |
| 33 | E08 | 4325 | MMP16 | NM_005941 | 192051 | 205525 | 0.79 | 0.86 | -1.84 | -0.70 |
| 33 | E09 | 4326 | MMP17 | NM_016155 | 154045 | 182516 | 0.64 | 0.77 | 1.34 | 0.40 |
| 33 | E10 | 10893 | MMP24 | NM_006690 | 169410 | 179477 | 0.70 | 0.76 | 0.13 | 0.04 |
| 33 | E11 | 4650 | MYO9B | NM_004145 | 171284 | 183830 | 0.71 | 0.77 | 0.62 | 0.73 |
| 33 | E12 | 5511 | PPP1R8 | NM_002713 | 208324 | 204150 | 0.86 | 0.86 | -1.84 | 0.14 |
| 33 | F01 | NA | NA | NA | 228181 | 267995 | 0.94 | 1.13 | -2.80 | -4.89 |
| 33 | F02 | NA | pos | NA | 57888 | 84073 | 0.24 | 0.35 | 13.15 | 11.70 |
| 33 | F03 | 5520 | PPP2R2A | NM_002717 | 199182 | 221582 | 0.82 | 0.93 | 0.08 | 0.00 |
| 33 | F04 | 28227 | PR48 | NM_013239 | 199633 | 198695 | 0.82 | 0.84 | 0.26 | 2.46 |
| 33 | F05 | 9329 | GTF3C4 | NM_012204 | 209853 | 215885 | 0.87 | 0.91 | 0.09 | -0.16 |
| 33 | F06 | 9044 | BTAF1 | NM_003972 | 224162 | 214567 | 0.92 | 0.90 | -2.15 | -0.35 |
| 33 | F07 | 2023 | ENO1 | NM_001428 | 197883 | 200051 | 0.82 | 0.84 | -0.29 | 1.44 |
| 33 | F08 | 2033 | EP300 | NM_001429 | 206852 | 213709 | 0.85 | 0.90 | -1.49 | -0.03 |
| 33 | F09 | 2074 | ERCC6 | NM_000124 | 177236 | 188245 | 0.73 | 0.79 | 0.91 | 1.30 |
| 33 | F10 | 55284 | FLJ11011 | NM_018299 | 190367 | 195659 | 0.79 | 0.82 | -0.09 | -0.01 |
| 33 | F11 | 2962 | GTF2F1 | NM_002096 | 209468 | 213189 | 0.86 | 0.90 | -1.21 | -0.51 |
| 33 | F12 | 2963 | GTF2F2 | NM_004128 | 200971 | 210087 | 0.83 | 0.88 | 0.59 | 1.02 |
| 33 | G01 | NA | neg | NA | 253230 | 273211 | 1.04 | 1.15 | -4.99 | -6.32 |
| 33 | G02 | NA | neg | NA | 242706 | 243431 | 1.00 | 1.02 | -4.01 | -3.64 |
| 33 | G03 | 3065 | HDAC1 | NM_004964 | 223761 | 220579 | 0.92 | 0.93 | -2.07 | -0.87 |
| 33 | G04 | 83933 | HDAC10 | NM_032019 | 203378 | 216205 | 0.84 | 0.91 | 0.06 | -0.08 |
| 33 | G05 | 3066 | HDAC2 | NM_001527 | 245331 | 216035 | 1.01 | 0.91 | -3.08 | -1.13 |
| 33 | G06 | 10014 | HDAC5 | NM_005474 | 206443 | 210561 | 0.85 | 0.89 | -0.34 | -0.95 |
| 33 | G07 | 51564 | HDAC7A | NM_015401 | 205515 | 204747 | 0.85 | 0.86 | -0.85 | 0.06 |
| 33 | G08 | 9734 | HDAC9 | NM_058176 | 182209 | 202669 | 0.75 | 0.85 | 0.97 | 0.00 |
| 33 | G09 | 3093 | HIP2 | NM_005339 | 186743 | 192009 | 0.77 | 0.81 | 0.17 | 0.00 |
| 33 | G10 | 10524 | HTATIP | NM_182710 | 162758 | 176439 | 0.67 | 0.74 | 2.64 | 0.77 |
| 33 | G11 | 3608 | ILF2 | NM_004515 | 197953 | 189877 | 0.82 | 0.80 | 0.02 | 0.63 |
| 33 | G12 | 5087 | PBX1 | NM_002585 | 209054 | 200506 | 0.86 | 0.84 | -0.02 | 0.92 |
| 33 | H01 | NA | NA | NA | 240271 | 256392 | 0.99 | 1.08 | -1.15 | -1.76 |
| 33 | H02 | NA | pos | NA | 59350 | 78859 | 0.24 | 0.33 | 15.79 | 14.26 |
| 33 | H03 | 5089 | PBX2 | NM_002586 | 253438 | 282149 | 1.05 | 1.19 | -2.22 | -3.37 |
| 33 | H04 | 5090 | PBX3 | NM_006195 | 245266 | 282566 | 1.01 | 1.19 | -1.24 | -3.02 |

| | | | | | | | | | | |
|---|---|---|---|---|---|---|---|---|---|---|
| 33 | H05 | 80714 | PBX4 | NM_025245 | 242541 | 235523 | 1.00 | 0.99 | -0.19 | 0.16 |
| 33 | H06 | 8850 | PCAF | NM_003884 | 234875 | 237382 | 0.97 | 1.00 | -0.38 | -0.32 |
| 33 | H07 | 22921 | MSRB | NM_012228 | 213337 | 239300 | 0.88 | 1.01 | 1.04 | -0.01 |
| 33 | H08 | 85441 | PRIC285 | NM_033405 | 208632 | 216775 | 0.86 | 0.91 | 1.12 | 1.78 |
| 33 | H09 | 3516 | RBPSUH | NM_005349 | 215318 | 229381 | 0.89 | 0.97 | 0.12 | -0.32 |
| 33 | H10 | 6595 | SMARCA2 | NM_003070 | 220563 | 218652 | 0.91 | 0.92 | -0.14 | 0.01 |
| 33 | H11 | 6597 | SMARCA4 | NM_003072 | 193338 | 200566 | 0.80 | 0.84 | 3.08 | 2.72 |
| 33 | H12 | 8467 | SMARCA5 | NM_003601 | 214184 | 211006 | 0.88 | 0.89 | 2.13 | 3.03 |
| 34 | A01 | NA | pos | NA | 52282 | 55264 | 0.21 | 0.22 | 14.67 | 16.33 |
| 34 | A02 | NA | NA | NA | 214477 | 231574 | 0.87 | 0.94 | -0.89 | -4.16 |
| 34 | A03 | 10847 | SRCAP | NM_006662 | 218288 | 182468 | 0.89 | 0.74 | -0.41 | 3.14 |
| 34 | A04 | 6830 | SUPT6H | NM_003170 | 170278 | 160076 | 0.69 | 0.65 | 2.84 | 4.33 |
| 34 | A05 | 80155 | TBDN100 | NM_057175 | 208715 | 195885 | 0.85 | 0.79 | 0.24 | 0.12 |
| 34 | A06 | 7157 | TP53 | NM_000546 | 183392 | 190344 | 0.75 | 0.77 | 2.54 | 0.52 |
| 34 | A07 | 7251 | TSG101 | NM_006292 | 232497 | 220449 | 0.95 | 0.89 | -2.84 | -2.78 |
| 34 | A08 | 8458 | TTF2 | NM_003594 | 210305 | 189515 | 0.86 | 0.77 | -1.67 | -0.10 |
| 34 | A09 | 7335 | UBE2V1 | NM_021988 | 207006 | 197436 | 0.84 | 0.80 | -0.23 | -0.24 |
| 34 | A10 | 57109 | XPMC2H | NM_020385 | 185910 | 182555 | 0.76 | 0.74 | 1.90 | 0.20 |
| 34 | A11 | 8891 | EIF2B3 | NM_020365 | 189717 | 194254 | 0.77 | 0.79 | 0.76 | -1.23 |
| 34 | A12 | 1983 | EIF5 | NM_001969 | 226423 | 214921 | 0.92 | 0.87 | -0.61 | -1.68 |
| 34 | B01 | NA | neg | NA | 258427 | 265016 | 1.05 | 1.07 | -5.88 | -6.42 |
| 34 | B02 | NA | neg | NA | 213155 | 249691 | 0.87 | 1.01 | -1.53 | -4.63 |
| 34 | B03 | 5188 | PET112L | NM_004564 | 215886 | 196669 | 0.88 | 0.80 | -0.94 | 3.12 |
| 34 | B04 | 394 | ARHGAP5 | NM_001173 | 208013 | 219829 | 0.85 | 0.89 | -1.55 | -0.98 |
| 34 | B05 | 57448 | BIRC6 | NM_016252 | 194274 | 199300 | 0.79 | 0.81 | 0.86 | 1.36 |
| 34 | B06 | 1121 | CHM | NM_000390 | 201622 | 210360 | 0.82 | 0.85 | 0.02 | -0.17 |
| 34 | B07 | 1122 | CHML | NM_001821 | 193291 | 203930 | 0.79 | 0.83 | 0.15 | 0.77 |
| 34 | B08 | 4258 | MGST2 | NM_002413 | 187887 | 201818 | 0.77 | 0.82 | -0.29 | 0.10 |
| 34 | B09 | 5148 | PDE6G | NM_002602 | 196762 | 208178 | 0.80 | 0.84 | -0.01 | 0.14 |
| 34 | B10 | 5149 | PDE6H | NM_006205 | 198072 | 204019 | 0.81 | 0.83 | -0.03 | -0.67 |
| 34 | B11 | 10531 | PITRM1 | NM_014889 | 187173 | 198838 | 0.76 | 0.80 | 0.24 | -0.13 |
| 34 | B12 | 51400 | PME-1 | NM_016147 | 200131 | 216300 | 0.82 | 0.88 | 1.15 | -0.20 |
| 34 | C01 | NA | pos | NA | 63264 | 66138 | 0.26 | 0.27 | 11.70 | 16.12 |
| 34 | C02 | NA | NA | NA | 223302 | 236077 | 0.91 | 0.96 | -3.66 | -3.63 |
| 34 | C03 | 5506 | PPP1R3A | NM_002711 | 194524 | 220368 | 0.79 | 0.89 | -0.05 | -0.22 |
| 34 | C04 | 5973 | RENBP | NM_002910 | 178931 | 198597 | 0.73 | 0.80 | 0.09 | 0.90 |
| 34 | C05 | 5055 | SERPINB2 | NM_002575 | 209328 | 194372 | 0.85 | 0.79 | -1.74 | 1.35 |
| 34 | C06 | 5054 | SERPINE1 | NM_000602 | 167478 | 186411 | 0.68 | 0.75 | 2.14 | 2.03 |
| 34 | C07 | 8228 | DXS1283E | XM_047871 | 181189 | 188576 | 0.74 | 0.76 | 0.16 | 1.97 |
| 34 | C08 | 50640 | IPLA2(GAMMA) | XM_291241 | 172948 | 209935 | 0.71 | 0.85 | -0.01 | -1.43 |
| 34 | C09 | 8398 | PLA2G6 | NM_003560 | 184509 | 211574 | 0.75 | 0.86 | 0.01 | -0.84 |
| 34 | C10 | 10152 | ABI2 | NM_005759 | 213229 | 194972 | 0.87 | 0.79 | -2.64 | -0.20 |
| 34 | C11 | 102 | ADAM10 | NM_001110 | 173318 | 191650 | 0.71 | 0.78 | 0.42 | 0.13 |
| 34 | C12 | 4185 | ADAM11 | NM_002390 | 246827 | 218197 | 1.01 | 0.88 | -4.49 | -1.01 |
| 34 | D01 | NA | neg | NA | 252839 | 275500 | 1.03 | 1.11 | -5.30 | -9.66 |
| 34 | D02 | NA | neg | NA | 213905 | 232680 | 0.87 | 0.94 | -1.56 | -4.68 |
| 34 | D03 | 2515 | ADAM2 | NM_001464 | 189710 | 200071 | 0.77 | 0.81 | 1.61 | 0.70 |
| 34 | D04 | 53616 | ADAM22 | NM_004194 | 179717 | 188423 | 0.73 | 0.76 | 1.21 | 0.64 |
| 34 | D05 | 8745 | ADAM23 | NM_003812 | 204251 | 208509 | 0.83 | 0.84 | -0.06 | -1.74 |
| 34 | D06 | 27299 | ADAMDEC1 | NM_014479 | 202480 | 195544 | 0.83 | 0.79 | -0.02 | -0.48 |
| 34 | D07 | 9510 | ADAMTS1 | NM_006988 | 207881 | 191695 | 0.85 | 0.78 | -1.21 | 0.17 |
| 34 | D08 | 11093 | ADAMTS13 | NM_139025 | 172053 | 186122 | 0.70 | 0.75 | 1.27 | -0.10 |
| 34 | D09 | 11096 | ADAMTS5 | NM_007038 | 196756 | 189389 | 0.80 | 0.77 | 0.03 | 0.30 |
| 34 | D10 | 389 | RHOC | NM_175744 | 203006 | 213239 | 0.83 | 0.86 | -0.47 | -3.76 |
| 34 | D11 | 51752 | ARTS-1 | NM_016442 | 210497 | 218625 | 0.86 | 0.88 | -1.96 | -4.45 |
| 34 | D12 | 438 | ASMT | NM_004043 | 203040 | 195378 | 0.83 | 0.79 | 0.91 | 0.20 |
| 34 | E01 | NA | neg | NA | 256636 | 244643 | 1.05 | 0.99 | -5.63 | -5.23 |
| 34 | E02 | NA | neg | NA | 199016 | 225336 | 0.81 | 0.91 | -0.10 | -2.99 |
| 34 | E03 | 649 | BMP1 | NM_001199 | 199527 | 211407 | 0.81 | 0.86 | 0.70 | 0.22 |
| 34 | E04 | 825 | CAPN3 | NM_000070 | 177374 | 206470 | 0.72 | 0.84 | 1.46 | -0.61 |
| 34 | E05 | 841 | CASP8 | NM_001228 | 203392 | 211642 | 0.83 | 0.86 | 0.06 | -1.26 |
| 34 | E06 | 8837 | CFLAR | NM_003879 | 207110 | 194497 | 0.84 | 0.79 | -0.43 | 0.48 |
| 34 | E07 | 8532 | CPZ | NM_003652 | 159821 | 201831 | 0.65 | 0.82 | 3.43 | -0.17 |
| 34 | E08 | 6372 | CXCL6 | NM_002993 | 184544 | 176835 | 0.75 | 0.72 | 0.10 | 1.82 |
| 34 | E09 | 1857 | DVL3 | NM_004423 | 197820 | 205472 | 0.81 | 0.83 | -0.04 | -0.73 |
| 34 | E10 | 1890 | ECGF1 | NM_001953 | 217436 | 219177 | 0.89 | 0.89 | -1.82 | -3.61 |
| 34 | E11 | 128178 | EDARADD | NM_080738 | 198537 | 185534 | 0.81 | 0.75 | -0.78 | 0.23 |
| 34 | E12 | 2147 | F2 | NM_000506 | 237324 | 198770 | 0.97 | 0.80 | -2.35 | 0.65 |
| 34 | F01 | NA | NA | NA | 263873 | 227862 | 1.08 | 0.92 | -6.60 | -3.18 |
| 34 | F02 | NA | pos | NA | 54433 | 81454 | 0.22 | 0.33 | 13.49 | 13.84 |
| 34 | F03 | 2166 | FAAH | NM_001441 | 201043 | 218533 | 0.82 | 0.88 | 0.28 | -0.51 |
| 34 | F04 | 2280 | FKBP1A | NM_000801 | 190700 | 197677 | 0.78 | 0.80 | -0.09 | 0.51 |
| 34 | F05 | 2287 | FKBP3 | NM_002013 | 210838 | 205461 | 0.86 | 0.83 | -0.93 | -0.44 |
| 34 | F06 | 2767 | GNA11 | NM_002067 | 190874 | 198010 | 0.78 | 0.80 | 0.85 | 0.17 |
| 34 | F07 | 2771 | GNAI2 | NM_002070 | 194313 | 222839 | 0.79 | 0.90 | -0.15 | -2.51 |
| 34 | F08 | 2821 | GPI | NM_000175 | 181248 | 192124 | 0.74 | 0.78 | 0.14 | 0.14 |
| 34 | F09 | 3082 | HGF | NM_000601 | 188512 | 201276 | 0.77 | 0.81 | 0.58 | -0.14 |
| 34 | F10 | 3549 | IHH | XM_050846 | 195236 | 184999 | 0.80 | 0.75 | 0.03 | 0.46 |
| 34 | F11 | 51135 | IRAK4 | NM_016123 | 198412 | 190504 | 0.81 | 0.77 | -1.04 | -0.24 |
| 34 | F12 | 84695 | LOXL3 | NM_032603 | 210404 | 184861 | 0.86 | 0.75 | -0.04 | 2.37 |
| 34 | G01 | NA | neg | NA | 252858 | 268756 | 1.03 | 1.09 | -5.34 | -7.98 |
| 34 | G02 | NA | neg | NA | 237514 | 227587 | 0.97 | 0.92 | -3.87 | -3.20 |
| 34 | G03 | 4052 | LTBP1 | NM_000627 | 206812 | 222464 | 0.84 | 0.90 | -0.07 | -1.01 |
| 34 | G04 | 9372 | ZFYVE9 | NM_004799 | 201286 | 234622 | 0.82 | 0.95 | -0.90 | -3.84 |
| 34 | G05 | 10905 | MAN1A2 | NM_006699 | 192018 | 195543 | 0.78 | 0.79 | 1.08 | 0.66 |
| 34 | G06 | 8638 | OASL | NM_003733 | 208647 | 218545 | 0.85 | 0.88 | -0.65 | -2.26 |
| 34 | G07 | 10135 | PBEF1 | NM_005746 | 159590 | 175374 | 0.65 | 0.71 | 3.38 | 2.96 |
| 34 | G08 | 5151 | PDE8A | NM_002605 | 184824 | 195106 | 0.75 | 0.79 | 0.00 | -0.25 |

| | | | | | | | | | | |
|---|---|---|---|---|---|---|---|---|---|---|
| 34 | G09 | 8622 | PDE8B | NM_003719 | 196684 | 196478 | 0.80 | 0.79 | 0.00 | 0.37 |
| 34 | G10 | 8993 | PGLYRP1 | NM_005091 | 182189 | 186446 | 0.74 | 0.75 | 1.49 | 0.24 |
| 34 | G11 | 5333 | PLCD1 | NM_006225 | 192181 | 184181 | 0.78 | 0.75 | -0.24 | 0.44 |
| 34 | G12 | 5335 | PLCG1 | NM_002660 | 211612 | 208207 | 0.86 | 0.84 | 0.04 | -0.40 |
| 34 | H01 | NA | NA | NA | 253891 | 269056 | 1.04 | 1.09 | -1.46 | -4.08 |
| 34 | H02 | NA | pos | NA | 66027 | 75882 | 0.27 | 0.31 | 16.56 | 18.38 |
| 34 | H03 | 5336 | PLCG2 | NM_002661 | 246994 | 264085 | 1.01 | 1.07 | 0.05 | -1.91 |
| 34 | H04 | 136853 | SRCRB4D | NM_080744 | 283915 | 239926 | 1.16 | 0.97 | -4.85 | -0.51 |
| 34 | H05 | 7039 | TGFA | NM_003236 | 268896 | 236205 | 1.10 | 0.96 | -2.32 | -0.12 |
| 34 | H06 | 834 | CASP1 | NM_001223 | 257907 | 251475 | 1.05 | 1.02 | -1.40 | -2.14 |
| 34 | H07 | 8567 | MADD | NM_003682 | 252063 | 266626 | 1.03 | 1.08 | -1.51 | -3.71 |
| 34 | H08 | 1387 | CREBBP | NM_004380 | 226366 | 225620 | 0.92 | 0.91 | 0.00 | 0.14 |
| 34 | H09 | 8202 | NCOA3 | NM_006534 | 238062 | 214657 | 0.97 | 0.87 | 0.00 | 2.20 |
| 34 | H10 | 9412 | SURB7 | NM_004264 | 208192 | 188336 | 0.85 | 0.76 | 2.97 | 3.97 |
| 34 | H11 | 344 | APOC2 | NM_000483 | 225339 | 215400 | 0.92 | 0.87 | 0.56 | 0.76 |
| 34 | H12 | 4018 | LPA | NM_005577 | 238168 | 221897 | 0.97 | 0.90 | 1.47 | 1.96 |
| 35 | A01 | NA | pos | NA | 49919 | 49113 | 0.21 | 0.21 | 16.60 | 15.57 |
| 35 | A02 | NA | NA | NA | 202313 | 198306 | 0.85 | 0.84 | -0.65 | 0.32 |
| 35 | A03 | 4948 | OCA2 | NM_000275 | 192110 | 188406 | 0.81 | 0.80 | 1.57 | 2.01 |
| 35 | A04 | 5476 | PPGB | NM_000308 | 197815 | 192883 | 0.83 | 0.82 | 0.28 | 1.50 |
| 35 | A05 | 29 | ABR | NM_001092 | 186156 | 194507 | 0.79 | 0.82 | 2.38 | 1.37 |
| 35 | A06 | 57679 | ALS2 | NM_020919 | 207270 | 199772 | 0.87 | 0.85 | 0.00 | -0.02 |
| 35 | A07 | 51129 | ANGPTL4 | NM_016109 | 198336 | 215223 | 0.84 | 0.91 | 0.00 | -0.77 |
| 35 | A08 | 10564 | ARFGEF2 | NM_006420 | 182014 | 192759 | 0.77 | 0.82 | 1.46 | 0.02 |
| 35 | A09 | 397 | ARHGDIB | NM_001175 | 188045 | 189435 | 0.79 | 0.80 | -0.04 | -0.06 |
| 35 | A10 | 398 | ARHGDIG | NM_001176 | 196216 | 195833 | 0.83 | 0.83 | -0.24 | -0.13 |
| 35 | A11 | 50650 | ARHGEF3 | NM_019555 | 194924 | 212114 | 0.82 | 0.90 | -0.15 | -1.88 |
| 35 | A12 | 50649 | ARHGEF4 | NM_015320 | 193935 | 203053 | 0.82 | 0.86 | -0.39 | 0.03 |
| 35 | B01 | NA | neg | NA | 258616 | 254996 | 1.09 | 1.08 | -6.62 | -5.47 |
| 35 | B02 | NA | neg | NA | 214635 | 217118 | 0.91 | 0.92 | -1.64 | -1.60 |
| 35 | B03 | 9459 | ARHGEF6 | NM_004840 | 215148 | 207824 | 0.91 | 0.88 | -0.64 | 0.04 |
| 35 | B04 | 8874 | ARHGEF7 | NM_003899 | 190429 | 217581 | 0.80 | 0.92 | 1.52 | -1.02 |
| 35 | B05 | 7873 | ARMET | NM_006010 | 210743 | 204349 | 0.89 | 0.87 | 0.00 | 0.37 |
| 35 | B06 | 8412 | BCAR3 | NM_003567 | 174962 | 195701 | 0.74 | 0.83 | 4.06 | 0.40 |
| 35 | B07 | 23786 | BCL2L13 | NM_015367 | 201898 | 214711 | 0.85 | 0.91 | 0.00 | -0.71 |
| 35 | B08 | 10565 | BIG1 | NM_006421 | 161270 | 152284 | 0.68 | 0.65 | 4.21 | 4.17 |
| 35 | B09 | 663 | BNIP2 | NM_004330 | 198200 | 197404 | 0.84 | 0.84 | -0.79 | -0.86 |
| 35 | B10 | 720 | C4A | NM_007293 | 198297 | 188506 | 0.84 | 0.80 | -0.07 | 0.63 |
| 35 | B11 | 26256 | CABYR | NM_012189 | 194009 | 195932 | 0.82 | 0.83 | 0.36 | -0.22 |
| 35 | B12 | 9722 | CAPON | NM_014697 | 201819 | 203655 | 0.85 | 0.86 | -0.88 | -0.03 |
| 35 | C01 | NA | pos | NA | 62083 | 62095 | 0.26 | 0.26 | 15.50 | 14.21 |
| 35 | C02 | NA | NA | NA | 219259 | 220645 | 0.93 | 0.94 | -2.30 | -1.99 |
| 35 | C03 | 831 | CAST | NM_173061 | 214466 | 208192 | 0.91 | 0.88 | -0.69 | -0.04 |
| 35 | C04 | 1475 | CSTA | NM_005213 | 183039 | 210475 | 0.77 | 0.89 | 2.22 | -0.33 |
| 35 | C05 | 1476 | CSTB | NM_000100 | 190682 | 162698 | 0.80 | 0.69 | 2.14 | 4.59 |
| 35 | C06 | 8655 | DNCL1 | NM_003746 | 210009 | 198403 | 0.89 | 0.84 | -0.04 | 0.09 |
| 35 | C07 | 1793 | DOCK1 | NM_001380 | 194440 | 195258 | 0.82 | 0.83 | 0.71 | 1.24 |
| 35 | C08 | 9166 | EBAG9 | NM_004215 | 199216 | 192321 | 0.84 | 0.82 | -0.22 | 0.04 |
| 35 | C09 | 26269 | FBXO8 | NM_012180 | 189718 | 175403 | 0.80 | 0.74 | 0.04 | 1.35 |
| 35 | C10 | 55103 | RALGPS2 | NM_018037 | 195816 | 213650 | 0.83 | 0.91 | 0.07 | -1.98 |
| 35 | C11 | 2622 | GAS8 | NM_001481 | 203820 | 205629 | 0.86 | 0.87 | -0.89 | -1.25 |
| 35 | C12 | 2664 | GDI1 | NM_001493 | 217614 | 225774 | 0.92 | 0.96 | -2.80 | -2.32 |
| 35 | D01 | NA | neg | NA | 253566 | 263846 | 1.07 | 1.12 | -5.25 | -5.30 |
| 35 | D02 | NA | neg | NA | 228544 | 224716 | 0.96 | 0.95 | -2.42 | -1.30 |
| 35 | D03 | 2665 | GDI2 | NM_001494 | 219282 | 216681 | 0.93 | 0.92 | -0.31 | 0.21 |
| 35 | D04 | 2760 | GM2A | NM_000405 | 213294 | 221893 | 0.90 | 0.94 | -0.28 | -0.38 |
| 35 | D05 | 2874 | GPS2 | NM_004489 | 222238 | 191169 | 0.94 | 0.81 | -0.51 | 2.80 |
| 35 | D06 | 23092 | ARHGAP26 | NM_015071 | 212968 | 212879 | 0.90 | 0.90 | 0.55 | -0.28 |
| 35 | D07 | 2981 | GUCA2B | NM_007102 | 179405 | 219163 | 0.76 | 0.93 | 3.34 | -0.09 |
| 35 | D08 | 8997 | KALRN | NM_003947 | 176631 | 214153 | 0.75 | 0.91 | 3.26 | -1.08 |
| 35 | D09 | 80316 | I-4 | NM_025210 | 195805 | 194026 | 0.83 | 0.82 | 0.28 | 0.56 |
| 35 | D10 | 3476 | IGBP1 | NM_001551 | 215696 | 210559 | 0.91 | 0.89 | -1.25 | -0.55 |
| 35 | D11 | 51195 | RAPGEFL1 | NM_016339 | 207154 | 203288 | 0.87 | 0.86 | -0.34 | 0.11 |
| 35 | D12 | 79109 | MAPKAP1 | NM_024117 | 195013 | 200488 | 0.82 | 0.85 | 0.68 | 1.37 |
| 35 | E01 | NA | neg | NA | 245345 | 247065 | 1.04 | 1.05 | -4.50 | -4.98 |
| 35 | E02 | NA | neg | NA | 212570 | 215473 | 0.90 | 0.91 | -0.79 | -1.75 |
| 35 | E03 | 22924 | MAPRE3 | NM_012326 | 194815 | 192797 | 0.82 | 0.82 | 2.28 | 1.25 |
| 35 | E04 | 4168 | MCF2 | NM_005369 | 226176 | 214412 | 0.95 | 0.91 | -1.91 | -1.01 |
| 35 | E05 | 4763 | NF1 | NM_000267 | 214114 | 213961 | 0.90 | 0.91 | 0.23 | -0.93 |
| 35 | E06 | 4947 | OAZ2 | NM_002537 | 225308 | 196253 | 0.95 | 0.83 | -1.02 | 0.02 |
| 35 | E07 | 51686 | OAZ3 | NM_016178 | 183712 | 204831 | 0.78 | 0.87 | 2.67 | -0.02 |
| 35 | E08 | 4983 | OPHN1 | NM_002547 | 206767 | 169343 | 0.87 | 0.72 | -0.32 | 2.10 |
| 35 | E09 | 9411 | PARG1 | NM_004815 | 209432 | 198372 | 0.88 | 0.84 | -1.44 | -1.28 |
| 35 | E10 | 11142 | PKIG | NM_007066 | 188776 | 184049 | 0.80 | 0.78 | 1.62 | 0.76 |
| 35 | E11 | 11070 | PL6 | NM_007024 | 204650 | 187293 | 0.86 | 0.79 | -0.23 | 0.34 |
| 35 | E12 | 9373 | PLAA | NM_004253 | 187022 | 205861 | 0.79 | 0.87 | 1.41 | -0.57 |
| 35 | F01 | NA | NA | NA | 269322 | 249323 | 1.14 | 1.06 | -8.06 | -4.99 |
| 35 | F02 | NA | pos | NA | 58865 | 68658 | 0.25 | 0.29 | 15.76 | 13.47 |
| 35 | F03 | 6992 | PPP1R11 | NM_021959 | 204906 | 208488 | 0.86 | 0.88 | 0.29 | -0.14 |
| 35 | F04 | 4660 | PPP1R12B | NM_002481 | 178496 | 178301 | 0.75 | 0.76 | 2.63 | 2.89 |
| 35 | F05 | 5510 | PPP1R7 | NM_002712 | 208668 | 219957 | 0.88 | 0.93 | 0.00 | -1.33 |
| 35 | F06 | 5522 | PPP2R2C | NM_020416 | 208771 | 183153 | 0.88 | 0.78 | 0.00 | 1.58 |
| 35 | F07 | 5524 | PPP2R4 | NM_021131 | 211383 | 206521 | 0.89 | 0.88 | -1.31 | 0.02 |
| 35 | F08 | 9267 | PSCD1 | NM_004762 | 203332 | 192246 | 0.86 | 0.81 | -0.79 | -0.02 |
| 35 | F09 | 9266 | PSCD2 | NM_004228 | 182168 | 187378 | 0.77 | 0.79 | 0.79 | 0.06 |
| 35 | F10 | 5720 | PSME1 | NM_176783 | 203021 | 203658 | 0.86 | 0.86 | -0.84 | -1.02 |
| 35 | F11 | 5863 | RGL2 | NM_004761 | 193737 | 187146 | 0.82 | 0.79 | 0.15 | 0.57 |
| 35 | F12 | 25782 | RAB3-GAP150 | NM_012414 | 200372 | 210717 | 0.85 | 0.89 | -0.95 | -0.85 |

| | | | | | | | | | | |
|---|---|---|---|---|---|---|---|---|---|---|
| 35 | G01 | NA | neg | NA | 215521 | 254543 | 0.91 | 1.08 | -1.67 | -4.98 |
| 35 | G02 | NA | neg | NA | 247545 | 222369 | 1.04 | 0.94 | -5.29 | -1.69 |
| 35 | G03 | 5900 | RALGDS | NM_006266 | 208296 | 233078 | 0.88 | 0.99 | 0.21 | -2.10 |
| 35 | G04 | 5905 | RANGAP1 | NM_002883 | 220429 | 204683 | 0.93 | 0.87 | -1.81 | 0.74 |
| 35 | G05 | 5909 | RAP1GA1 | NM_002885 | 225745 | 215966 | 0.95 | 0.92 | -1.63 | -0.37 |
| 35 | G06 | 5910 | RAP1GDS1 | NM_021159 | 197430 | 210621 | 0.83 | 0.89 | 1.59 | -0.68 |
| 35 | G07 | 5921 | RASA1 | NM_002890 | 204300 | 186558 | 0.86 | 0.79 | -0.20 | 2.61 |
| 35 | G08 | 5922 | RASA2 | NM_006506 | 201585 | 202367 | 0.85 | 0.86 | -0.28 | -0.51 |
| 35 | G09 | 22821 | RASA3 | NM_007368 | 198266 | 201828 | 0.84 | 0.86 | -0.72 | -0.87 |
| 35 | G10 | 9462 | RASAL2 | NM_004841 | 184783 | 194944 | 0.78 | 0.83 | 1.52 | 0.41 |
| 35 | G11 | 5923 | RASGRF1 | NM_002891 | 194714 | 194108 | 0.82 | 0.82 | 0.34 | 0.41 |
| 35 | G12 | 8434 | RECK | NM_021111 | 191151 | 190651 | 0.81 | 0.81 | 0.39 | 1.74 |
| 35 | H01 | NA | NA | NA | 254646 | 254870 | 1.07 | 1.08 | -3.03 | -3.09 |
| 35 | H02 | NA | pos | NA | 58942 | 80036 | 0.25 | 0.34 | 19.13 | 14.78 |
| 35 | H03 | 23179 | RGL1 | NM_015149 | 239128 | 275711 | 1.01 | 1.17 | -0.21 | -4.53 |
| 35 | H04 | 266747 | RGR | NM_153615 | 247635 | 227629 | 1.05 | 0.96 | -1.82 | 0.33 |
| 35 | H05 | 6050 | RNH | NM_002939 | 274718 | 280453 | 1.16 | 1.19 | -4.10 | -5.04 |
| 35 | H06 | 51156 | SERPINA10 | NM_016186 | 264573 | 260730 | 1.12 | 1.11 | -2.94 | -3.88 |
| 35 | H07 | 5104 | SERPINA5 | NM_000624 | 257416 | 210741 | 1.09 | 0.89 | -3.14 | 2.06 |
| 35 | H08 | 1992 | SERPINB1 | NM_030666 | 224279 | 229492 | 0.95 | 0.97 | 0.22 | -1.36 |
| 35 | H09 | 5275 | SERPINB13 | NM_012397 | 210605 | 205166 | 0.89 | 0.87 | 0.95 | 0.71 |
| 35 | H10 | 6317 | SERPINB3 | NM_006919 | 207815 | 216590 | 0.88 | 0.92 | 1.99 | 0.13 |
| 35 | H11 | 6318 | SERPINB4 | NM_002974 | 202351 | 217991 | 0.85 | 0.92 | 2.55 | -0.11 |
| 35 | H12 | 5268 | SERPINB5 | NM_002639 | 213096 | 216295 | 0.90 | 0.92 | 0.98 | 1.05 |
| 36 | A01 | NA | pos | NA | 51344 | 54851 | 0.22 | 0.23 | 14.33 | 19.48 |
| 36 | A02 | NA | NA | NA | 226746 | 236973 | 0.97 | 0.98 | -2.98 | -5.72 |
| 36 | A03 | 5176 | SERPINF1 | NM_002615 | 241160 | 213719 | 1.03 | 0.89 | -3.58 | 0.00 |
| 36 | A04 | 710 | SERPING1 | NM_000062 | 205881 | 200753 | 0.88 | 0.83 | -1.01 | 0.02 |
| 36 | A05 | 5274 | SERPINI1 | NM_005025 | 197430 | 194840 | 0.85 | 0.81 | 2.18 | 0.32 |
| 36 | A06 | 5276 | SERPINI2 | NM_006217 | 200311 | 197134 | 0.86 | 0.82 | 0.29 | 0.70 |
| 36 | A07 | 10044 | SH2D3C | NM_005489 | 205107 | 217333 | 0.88 | 0.90 | -0.10 | -3.28 |
| 36 | A08 | 6590 | SLPI | NM_003064 | 184359 | 191005 | 0.79 | 0.79 | 0.26 | 0.19 |
| 36 | A09 | 6655 | SOS2 | NM_006939 | 193206 | 192489 | 0.83 | 0.80 | -0.49 | -0.77 |
| 36 | A10 | 6690 | SPINK1 | NM_003122 | 200031 | 196861 | 0.86 | 0.82 | -1.25 | 0.00 |
| 36 | A11 | 11005 | SPINK5 | NM_006846 | 187219 | 200539 | 0.80 | 0.83 | 0.07 | -1.12 |
| 36 | A12 | 10653 | SPINT2 | NM_021102 | 193758 | 211213 | 0.83 | 0.88 | 0.34 | -2.33 |
| 36 | B01 | NA | neg | NA | 265536 | 268778 | 1.14 | 1.11 | -6.05 | -6.80 |
| 36 | B02 | NA | neg | NA | 239322 | 234736 | 1.02 | 0.97 | -3.47 | -2.09 |
| 36 | B03 | 7035 | TFPI | NM_006287 | 190092 | 237732 | 0.81 | 0.99 | 2.22 | 0.00 |
| 36 | B04 | 7076 | TIMP1 | NM_003254 | 195776 | 220662 | 0.84 | 0.92 | 0.74 | 0.58 |
| 36 | B05 | 7077 | TIMP2 | NM_003255 | 227608 | 221271 | 0.97 | 0.92 | -0.04 | -0.02 |
| 36 | B06 | 7078 | TIMP3 | NM_000362 | 210338 | 244606 | 0.90 | 1.01 | 0.06 | -2.55 |
| 36 | B07 | 7079 | TIMP4 | NM_003256 | 203439 | 217736 | 0.87 | 0.90 | 0.83 | -0.02 |
| 36 | B08 | 7454 | WAS | NM_000377 | 209243 | 226704 | 0.90 | 0.94 | -1.44 | -1.43 |
| 36 | B09 | 58189 | WFDC1 | NM_021197 | 195055 | 205241 | 0.84 | 0.85 | 0.08 | 0.79 |
| 36 | B10 | 58 | ACTA1 | NM_001100 | 218457 | 220856 | 0.94 | 0.92 | -2.31 | 0.00 |
| 36 | B11 | 59 | ACTA2 | NM_001613 | 206514 | 195931 | 0.88 | 0.81 | -1.08 | 2.83 |
| 36 | B12 | 60 | ACTB | NM_001101 | 212152 | 203177 | 0.91 | 0.84 | -0.72 | 2.10 |
| 36 | C01 | NA | pos | NA | 67570 | 65713 | 0.29 | 0.27 | 14.28 | 20.21 |
| 36 | C02 | NA | NA | NA | 225062 | 224912 | 0.96 | 0.93 | -1.26 | -1.82 |
| 36 | C03 | 70 | ACTC | NM_005159 | 230692 | 233981 | 0.99 | 0.97 | -0.99 | -0.57 |
| 36 | C04 | 71 | ACTG1 | NM_001614 | 185615 | 220796 | 0.79 | 0.92 | 2.54 | -0.53 |
| 36 | C05 | 72 | ACTG2 | NM_001615 | 221515 | 213154 | 0.95 | 0.88 | 1.36 | 0.02 |
| 36 | C06 | 51412 | ACTL6 | NM_016188 | 189009 | 217331 | 0.81 | 0.90 | 2.96 | 0.14 |
| 36 | C07 | 10120 | ACTR1B | NM_005735 | 219284 | 192829 | 0.94 | 0.80 | 0.06 | 2.34 |
| 36 | C08 | 10513 | APPBP2 | NM_006380 | 189901 | 208780 | 0.81 | 0.87 | 1.27 | -0.04 |
| 36 | C09 | 1639 | DCTN1 | NM_004082 | 215285 | 200910 | 0.92 | 0.83 | -1.11 | 0.30 |
| 36 | C10 | 140735 | DLC2 | NM_080677 | 211475 | 215197 | 0.91 | 0.89 | -0.83 | -0.31 |
| 36 | C11 | 27019 | DNAI1 | NM_012144 | 204553 | 202465 | 0.88 | 0.84 | -0.08 | 0.84 |
| 36 | C12 | 1780 | DNCI1 | NM_004411 | 213756 | 223693 | 0.92 | 0.93 | -0.08 | -1.83 |
| 36 | D01 | NA | neg | NA | 261193 | 269626 | 1.12 | 1.12 | -6.26 | -8.47 |
| 36 | D02 | NA | neg | NA | 227648 | 234601 | 0.98 | 0.97 | -2.95 | -3.63 |
| 36 | D03 | 1781 | DNCI2 | NM_001378 | 203515 | 217498 | 0.87 | 0.90 | 0.26 | 1.24 |
| 36 | D04 | 64837 | KLC2 | NM_022822 | 217372 | 220860 | 0.93 | 0.92 | -2.02 | -1.00 |
| 36 | D05 | 147700 | KLC2L | NM_177417 | 224065 | 216274 | 0.96 | 0.90 | -0.32 | -0.88 |
| 36 | D06 | 3831 | KNS2 | NM_005552 | 203001 | 186333 | 0.87 | 0.77 | 0.15 | 3.96 |
| 36 | D07 | 6993 | TCTEL1 | NM_006519 | 206355 | 183624 | 0.88 | 0.76 | -0.10 | 3.15 |
| 36 | D08 | 1778 | DNCH1 | NM_001376 | 192214 | 209771 | 0.82 | 0.87 | -0.39 | -0.64 |
| 36 | D09 | 4621 | MYH3 | NM_002470 | 190359 | 199204 | 0.82 | 0.83 | -0.08 | 0.07 |
| 36 | D10 | 4622 | MYH4 | NM_017533 | 174395 | 186885 | 0.75 | 0.77 | 1.40 | 3.14 |
| 36 | D11 | 58498 | MYL7 | NM_021223 | 188257 | 207884 | 0.81 | 0.86 | 0.09 | -0.38 |
| 36 | D12 | 4644 | MYO5A | NM_000259 | 197700 | 207760 | 0.85 | 0.86 | 0.08 | -0.09 |
| 36 | E01 | NA | neg | NA | 253739 | 256599 | 1.09 | 1.06 | -5.31 | -8.78 |
| 36 | E02 | NA | neg | NA | 216139 | 233870 | 0.93 | 0.97 | -1.60 | -5.64 |
| 36 | E03 | 4647 | MYO7A | NM_000260 | 201939 | 212476 | 0.86 | 0.88 | 0.63 | -0.18 |
| 36 | E04 | 4976 | OPA1 | NM_015560 | 198539 | 197532 | 0.85 | 0.82 | 0.04 | 0.11 |
| 36 | E05 | 86 | BAF53A | NM_004301 | 181102 | 189809 | 0.78 | 0.79 | 4.13 | 0.67 |
| 36 | E06 | 4625 | MYH7 | NM_000257 | 209540 | 183686 | 0.90 | 0.76 | -0.29 | 2.21 |
| 36 | E07 | 4626 | MYH8 | NM_002472 | 197746 | 196118 | 0.85 | 0.81 | 0.96 | -0.69 |
| 36 | E08 | 51168 | MYO15A | NM_016239 | 192965 | 186428 | 0.83 | 0.77 | -0.26 | 0.48 |
| 36 | E09 | 4646 | MYO6 | NM_004999 | 195543 | 185344 | 0.84 | 0.77 | -0.39 | -0.13 |
| 36 | E10 | 183 | AGT | NM_000029 | 164822 | 181072 | 0.71 | 0.75 | 2.56 | 1.84 |
| 36 | E11 | 27329 | ANGPTL3 | NM_014495 | 192056 | 203912 | 0.82 | 0.85 | -0.07 | -1.94 |
| 36 | E12 | 392 | ARHGAP1 | NM_004308 | 203912 | 199272 | 0.87 | 0.83 | -0.33 | -1.03 |
| 36 | F01 | NA | NA | NA | 254512 | 262618 | 1.09 | 1.09 | -5.98 | -7.85 |
| 36 | F02 | NA | pos | NA | 61745 | 78844 | 0.26 | 0.33 | 13.05 | 17.58 |
| 36 | F03 | 9826 | ARHGEF11 | NM_014784 | 201506 | 202056 | 0.86 | 0.84 | 0.08 | 3.03 |
| 36 | F04 | 23365 | ARHGEF12 | NM_015313 | 192048 | 198412 | 0.82 | 0.82 | 0.10 | 1.75 |

| | | | | | | | | | | |
|---|---|---|---|---|---|---|---|---|---|---|
| 36 | F05 | 10392 | CARD4 | NM_006092 | 226636 | 243877 | 0.97 | 1.01 | -0.95 | -5.05 |
| 36 | F06 | 1794 | DOCK2 | NM_004946 | 201297 | 213410 | 0.86 | 0.88 | -0.06 | -0.14 |
| 36 | F07 | 10818 | FRS2 | NM_006654 | 212054 | 190704 | 0.91 | 0.79 | -1.04 | 1.82 |
| 36 | F08 | 3084 | NRG1 | NM_013956 | 181120 | 184967 | 0.78 | 0.77 | 0.32 | 2.44 |
| 36 | F09 | 9693 | RAPGEF2 | NM_014247 | 175393 | 216741 | 0.75 | 0.90 | 1.01 | -2.71 |
| 36 | F10 | 5618 | PRLR | NM_000949 | 165230 | 207822 | 0.71 | 0.86 | 1.93 | -0.10 |
| 36 | F11 | 9265 | PSCD3 | NM_004227 | 193549 | 209155 | 0.83 | 0.87 | -0.81 | -0.90 |
| 36 | F12 | 25780 | RASGRP3 | NM_015376 | 199363 | 203922 | 0.85 | 0.85 | -0.47 | 0.09 |
| 36 | G01 | NA | neg | NA | 217520 | 247578 | 0.93 | 1.03 | -1.21 | -5.66 |
| 36 | G02 | NA | neg | NA | 216426 | 232706 | 0.93 | 0.96 | -1.10 | -3.60 |
| 36 | G03 | 7356 | SCGB1A1 | NM_003357 | 214494 | 233971 | 0.92 | 0.97 | -0.08 | -1.28 |
| 36 | G04 | 7074 | TIAM1 | NM_003253 | 204806 | 217835 | 0.88 | 0.90 | -0.04 | -0.82 |
| 36 | G05 | 8740 | TNFSF14 | NM_003807 | 227947 | 207111 | 0.98 | 0.86 | 0.04 | 0.14 |
| 36 | G06 | 10451 | VAV3 | NM_006113 | 213302 | 226650 | 0.91 | 0.94 | -0.13 | -1.86 |
| 36 | G07 | 177 | AGER | NM_001136 | 213475 | 207860 | 0.91 | 0.86 | -0.06 | -0.45 |
| 36 | G08 | 57491 | AHRR | NM_020731 | 217547 | 203138 | 0.93 | 0.84 | -2.15 | 0.04 |
| 36 | G09 | 23746 | AIPL1 | NM_014336 | 184608 | 198423 | 0.79 | 0.82 | 1.22 | -0.07 |
| 36 | G10 | 55911 | APOB48R | NM_018690 | 187706 | 202891 | 0.80 | 0.84 | 0.83 | 0.69 |
| 36 | G11 | 10603 | APS | NM_020979 | 192238 | 199300 | 0.82 | 0.83 | 0.44 | 0.57 |
| 36 | G12 | 8312 | AXIN1 | NM_003502 | 202259 | 191083 | 0.87 | 0.79 | 0.37 | 1.98 |
| 36 | H01 | NA | NA | NA | 254315 | 264155 | 1.09 | 1.10 | -3.30 | -6.88 |
| 36 | H02 | NA | pos | NA | 62197 | 98481 | 0.27 | 0.41 | 15.66 | 16.04 |
| 36 | H03 | 8313 | AXIN2 | NM_004655 | 236400 | 211093 | 1.01 | 0.88 | -0.71 | 2.96 |
| 36 | H04 | 682 | BSG | NM_001728 | 255196 | 219737 | 1.09 | 0.91 | -3.48 | -0.02 |
| 36 | H05 | 696 | BTN1A1 | NM_001732 | 253443 | 227633 | 1.09 | 0.94 | -0.94 | -1.62 |
| 36 | H06 | 9332 | CD163 | NM_004244 | 232165 | 231676 | 0.99 | 0.96 | -0.45 | -1.48 |
| 36 | H07 | 930 | CD19 | NM_001770 | 217561 | 212272 | 0.93 | 0.88 | 1.08 | 0.02 |
| 36 | H08 | 131450 | CD200R | NM_138806 | 206168 | 218173 | 0.88 | 0.90 | 0.51 | -0.97 |
| 36 | H09 | 922 | CD5L | NM_005894 | 207764 | 203910 | 0.89 | 0.85 | 0.47 | 0.25 |
| 36 | H10 | 972 | CD74 | NM_004355 | 225738 | 217464 | 0.97 | 0.90 | -1.39 | -0.26 |
| 36 | H11 | 973 | CD79A | NM_001783 | 192670 | 208447 | 0.83 | 0.86 | 1.94 | 0.38 |
| 36 | H12 | 974 | CD79B | NM_000626 | 204497 | 211700 | 0.88 | 0.88 | 1.68 | 0.20 |
| 37 | A01 | NA | pos | NA | 60325 | 42750 | 0.25 | 0.17 | 24.82 | 18.62 |
| 37 | A02 | NA | NA | NA | 250036 | 214222 | 1.02 | 0.86 | 0.68 | -0.99 |
| 37 | A03 | 8314 | BAP1 | NM_004656 | 262356 | 217378 | 1.07 | 0.87 | -0.40 | -0.66 |
| 37 | A04 | 8315 | BRAP | NM_006768 | 287707 | 205219 | 1.17 | 0.82 | -2.74 | 0.96 |
| 37 | A05 | 1540 | CYLD | NM_015247 | 265692 | 211305 | 1.08 | 0.84 | -0.14 | -0.04 |
| 37 | A06 | 10208 | C13ORF22 | NM_005800 | 257345 | 210505 | 1.05 | 0.84 | 0.17 | 0.50 |
| 37 | A07 | 9759 | HDAC4 | NM_006037 | 260287 | 208934 | 1.06 | 0.83 | -1.56 | 0.16 |
| 37 | A08 | 10013 | HDAC6 | NM_006044 | 245689 | 198995 | 1.00 | 0.79 | 0.49 | -0.26 |
| 37 | A09 | 220594 | LOC220594 | NM_145809 | 222062 | 198852 | 0.90 | 0.79 | 3.26 | 0.12 |
| 37 | A10 | 55611 | OTUB1 | NM_017670 | 254326 | 199703 | 1.03 | 0.80 | -0.32 | -0.37 |
| 37 | A11 | 7128 | TNFAIP3 | NM_006290 | 243836 | 196739 | 0.99 | 0.79 | 0.65 | 0.07 |
| 37 | A12 | 7345 | UCHL1 | NM_004181 | 258433 | 225870 | 1.05 | 0.90 | 1.52 | -2.88 |
| 37 | B01 | NA | neg | NA | 265245 | 260014 | 1.08 | 1.04 | -2.68 | -4.43 |
| 37 | B02 | NA | neg | NA | 246529 | 265109 | 1.00 | 1.06 | -0.30 | -5.02 |
| 37 | B03 | 7347 | UCHL3 | NM_006002 | 271338 | 238626 | 1.10 | 0.95 | -2.97 | -1.29 |
| 37 | B04 | 51377 | UCHL5 | NM_015984 | 218529 | 221764 | 0.89 | 0.89 | 4.64 | 0.87 |
| 37 | B05 | 7398 | USP1 | NM_003368 | 256668 | 238086 | 1.04 | 0.95 | -0.42 | -1.30 |
| 37 | B06 | 9100 | USP10 | NM_005153 | 259867 | 229835 | 1.06 | 0.92 | -1.58 | 0.08 |
| 37 | B07 | 8237 | USP11 | NM_004651 | 237829 | 242977 | 0.97 | 0.97 | -0.13 | -1.93 |
| 37 | B08 | 219333 | USP12 | NM_182488 | 237619 | 213099 | 0.97 | 0.85 | 0.09 | -0.08 |
| 37 | B09 | 8975 | USP13 | NM_003940 | 220147 | 216653 | 0.89 | 0.86 | 2.08 | -0.12 |
| 37 | B10 | 9097 | USP14 | NM_005151 | 219656 | 207282 | 0.89 | 0.83 | 2.66 | 0.56 |
| 37 | B11 | 9958 | USP15 | NM_006313 | 214736 | 199991 | 0.87 | 0.80 | 2.92 | 1.50 |
| 37 | B12 | 10600 | USP16 | NM_006447 | 272614 | 216127 | 1.11 | 0.86 | -1.71 | 0.03 |
| 37 | C01 | NA | pos | NA | 67593 | 62668 | 0.27 | 0.25 | 18.00 | 17.90 |
| 37 | C02 | NA | NA | NA | 253868 | 219873 | 1.03 | 0.88 | -5.70 | -0.09 |
| 37 | C03 | 11274 | USP18 | NM_017414 | 174921 | 204086 | 0.71 | 0.81 | 4.83 | 2.42 |
| 37 | C04 | 10869 | USP19 | XM_496642 | 219248 | 237754 | 0.89 | 0.95 | 0.08 | -1.20 |
| 37 | C05 | 9099 | USP2 | NM_004205 | 205430 | 200346 | 0.83 | 0.80 | 1.63 | 2.77 |
| 37 | C06 | 10868 | USP20 | NM_006676 | 213033 | 170904 | 0.87 | 0.68 | -0.08 | 6.58 |
| 37 | C07 | 27005 | USP21 | NM_012475 | 200235 | 226256 | 0.81 | 0.90 | 0.19 | -0.26 |
| 37 | C08 | 23326 | USP22 | XM_042698 | 201299 | 232381 | 0.82 | 0.93 | 0.25 | -2.53 |
| 37 | C09 | 23358 | USP24 | XM_165973 | 204349 | 186463 | 0.83 | 0.74 | -0.37 | 3.09 |
| 37 | C10 | 29761 | USP25 | NM_013396 | 216436 | 207758 | 0.88 | 0.83 | -1.39 | 0.26 |
| 37 | C11 | 83844 | USP26 | NM_031907 | 229269 | 217337 | 0.93 | 0.87 | -3.39 | -0.73 |
| 37 | C12 | 57646 | USP28 | NM_020886 | 274858 | 230210 | 1.12 | 0.92 | -6.46 | -1.82 |
| 37 | D01 | NA | neg | NA | 263570 | 253008 | 1.07 | 1.01 | -6.99 | -3.91 |
| 37 | D02 | NA | neg | NA | 266287 | 264868 | 1.08 | 1.06 | -7.34 | -5.27 |
| 37 | D03 | 57663 | USP29 | NM_020903 | 218516 | 228482 | 0.89 | 0.91 | -0.77 | -0.41 |
| 37 | D04 | 9960 | USP3 | NM_006537 | 200327 | 197747 | 0.81 | 0.79 | 2.43 | 3.34 |
| 37 | D05 | 84749 | USP30 | NM_032663 | 216697 | 238689 | 0.88 | 0.95 | 0.14 | -1.65 |
| 37 | D06 | 57478 | USP31 | NM_020718 | 209546 | 214201 | 0.85 | 0.86 | 0.30 | 1.59 |
| 37 | D07 | 23032 | USP33 | NM_015017 | 200312 | 225052 | 0.81 | 0.90 | 0.13 | -0.16 |
| 37 | D08 | 57558 | USP35 | XM_290527 | 218841 | 201167 | 0.89 | 0.80 | -2.04 | 1.01 |
| 37 | D09 | 57602 | USP36 | NM_025090 | 201500 | 218760 | 0.82 | 0.87 | -0.07 | -0.64 |
| 37 | D10 | 57695 | USP37 | NM_020935 | 200347 | 203011 | 0.81 | 0.81 | 0.60 | 0.77 |
| 37 | D11 | 84640 | USP38 | NM_032557 | 215088 | 211555 | 0.87 | 0.84 | -1.64 | -0.10 |
| 37 | D12 | 7375 | USP4 | NM_003363 | 231865 | 213086 | 0.94 | 0.85 | -1.05 | 0.10 |
| 37 | E01 | NA | neg | NA | 244671 | 241787 | 0.99 | 0.97 | -5.35 | -4.31 |
| 37 | E02 | NA | neg | NA | 214961 | 233768 | 0.87 | 0.93 | -1.57 | -3.40 |
| 37 | E03 | 84132 | USP42 | XM_166526 | 203361 | 206989 | 0.83 | 0.83 | 0.40 | 0.36 |
| 37 | E04 | 84101 | USP44 | NM_032147 | 220597 | 236333 | 0.90 | 0.94 | -0.91 | -2.76 |
| 37 | E05 | 64854 | USP46 | NM_022832 | 203781 | 205753 | 0.83 | 0.82 | 1.03 | 0.43 |
| 37 | E06 | 8078 | USP5 | NM_003481 | 222831 | 237315 | 0.91 | 0.95 | -2.15 | -2.74 |
| 37 | E07 | 9924 | USP52 | NM_014871 | 182014 | 200357 | 0.74 | 0.80 | 1.69 | 0.98 |
| 37 | E08 | 9098 | USP6 | NM_004505 | 205669 | 190116 | 0.84 | 0.76 | -1.12 | 0.59 |

| | | | | | | | | | | |
|---|---|---|---|---|---|---|---|---|---|---|
| 37 | E09 | 7874 | USP7 | NM_003470 | 192694 | 201196 | 0.78 | 0.80 | 0.29 | -0.32 |
| 37 | E10 | 9101 | USP8 | NM_005154 | 200802 | 215348 | 0.82 | 0.86 | -0.22 | -2.33 |
| 37 | E11 | 8239 | USP9X | NM_004652 | 198534 | 191117 | 0.81 | 0.76 | -0.30 | 0.55 |
| 37 | E12 | 8287 | USP9Y | NM_004654 | 216301 | 207256 | 0.88 | 0.83 | 0.17 | -0.92 |
| 37 | F01 | NA | NA | NA | 248013 | 244372 | 1.01 | 0.98 | -4.46 | -3.33 |
| 37 | F02 | NA | pos | NA | 67807 | 87327 | 0.28 | 0.35 | 18.47 | 14.63 |
| 37 | F03 | 8916 | HERC3 | NM_014606 | 207788 | 224498 | 0.84 | 0.90 | 1.15 | -0.36 |
| 37 | F04 | 84961 | FBXL20 | NM_032875 | 212263 | 222239 | 0.86 | 0.89 | 1.47 | 0.13 |
| 37 | F05 | 373 | TRIM23 | NM_001656 | 218517 | 220378 | 0.89 | 0.88 | 0.47 | 0.04 |
| 37 | F06 | 580 | BARD1 | NM_000465 | 192800 | 225301 | 0.78 | 0.90 | 2.99 | -0.08 |
| 37 | F07 | 1107 | CHD3 | NM_005852 | 219382 | 215376 | 0.89 | 0.86 | -1.74 | 0.54 |
| 37 | F08 | 8454 | CUL1 | NM_003592 | 212280 | 202661 | 0.86 | 0.81 | -0.65 | 0.43 |
| 37 | F09 | 8453 | CUL2 | NM_003591 | 207782 | 202420 | 0.84 | 0.81 | -0.31 | 0.82 |
| 37 | F10 | 8452 | CUL3 | NM_003590 | 213984 | 217428 | 0.87 | 0.87 | -0.58 | -1.29 |
| 37 | F11 | 8451 | CUL4A | NM_003589 | 210501 | 209798 | 0.86 | 0.84 | -0.50 | -0.31 |
| 37 | F12 | 8450 | CUL4B | NM_003588 | 225418 | 210742 | 0.92 | 0.84 | 0.33 | -0.03 |
| 37 | G01 | NA | neg | NA | 233373 | 247951 | 0.95 | 0.99 | -2.06 | -3.48 |
| 37 | G02 | NA | neg | NA | 245600 | 229133 | 1.00 | 0.91 | -3.62 | -1.33 |
| 37 | G03 | 55008 | HERC6 | NM_017912 | 230012 | 215719 | 0.93 | 0.86 | -1.15 | 0.90 |
| 37 | G04 | 84759 | NSPC1 | NM_032673 | 228636 | 231913 | 0.93 | 0.93 | -0.08 | -0.72 |
| 37 | G05 | 5896 | RAG1 | NM_000448 | 234030 | 200796 | 0.95 | 0.80 | -0.98 | 2.53 |
| 37 | G06 | 7337 | UBE3A | NM_000462 | 220785 | 229301 | 0.90 | 0.92 | -0.04 | -0.29 |
| 37 | G07 | 10075 | HUWE1 | NM_031407 | 211751 | 233382 | 0.86 | 0.93 | -0.24 | -1.27 |
| 37 | G08 | 7428 | VHL | NM_000551 | 209330 | 212165 | 0.85 | 0.85 | 0.26 | -0.40 |
| 37 | G09 | 10393 | ANAPC10 | NM_014885 | 208948 | 202459 | 0.85 | 0.81 | 0.07 | 1.07 |
| 37 | G10 | 10771 | ZMYND11 | NM_006624 | 206161 | 182527 | 0.84 | 0.73 | 0.95 | 2.96 |
| 37 | G11 | 10668 | CGRRF1 | NM_006568 | 208365 | 209995 | 0.85 | 0.84 | 0.30 | -0.07 |
| 37 | G12 | 5977 | DPF2 | NM_006268 | 220417 | 212013 | 0.90 | 0.85 | 1.49 | 0.08 |
| 37 | H01 | NA | NA | NA | 252876 | 233403 | 1.03 | 0.93 | -3.92 | -0.90 |
| 37 | H02 | NA | pos | NA | 66506 | 108063 | 0.27 | 0.43 | 19.79 | 13.43 |
| 37 | H03 | 113878 | DTX2 | NM_020892 | 215628 | 213111 | 0.88 | 0.85 | 1.30 | 2.11 |
| 37 | H04 | 114907 | FBXO32 | NM_058229 | 237049 | 234734 | 0.96 | 0.94 | -0.53 | -0.13 |
| 37 | H05 | 4008 | LMO7 | NM_005358 | 236752 | 273766 | 0.96 | 1.09 | -0.70 | -4.90 |
| 37 | H06 | 84708 | LNX | NM_032622 | 225032 | 239280 | 0.91 | 0.96 | 0.04 | -0.51 |
| 37 | H07 | 117584 | LOC117584 | NM_057178 | 212787 | 213543 | 0.86 | 0.85 | 0.25 | 1.92 |
| 37 | H08 | 57534 | MIB1 | NM_020774 | 216933 | 215989 | 0.88 | 0.86 | -0.09 | 0.08 |
| 37 | H09 | 4281 | MID1 | NM_000381 | 221836 | 225966 | 0.90 | 0.90 | -0.95 | -0.70 |
| 37 | H10 | 4331 | MNAT1 | NM_002431 | 216785 | 218714 | 0.88 | 0.87 | 0.22 | -0.26 |
| 37 | H11 | 115426 | UHRF2 | NM_152306 | 208214 | 204626 | 0.85 | 0.82 | 0.94 | 1.46 |
| 37 | H12 | 64324 | NSD1 | NM_022455 | 238393 | 203973 | 0.97 | 0.81 | -0.17 | 1.91 |
| 38 | A01 | NA | pos | NA | 59049 | 63201 | 0.24 | 0.25 | 20.42 | 13.18 |
| 38 | A02 | NA | NA | NA | 200482 | 253170 | 0.80 | 1.02 | -0.52 | -3.56 |
| 38 | A03 | 5192 | PEX10 | NM_002617 | 201739 | 241043 | 0.81 | 0.97 | 0.37 | -1.69 |
| 38 | A04 | 5193 | PEX12 | NM_000286 | 211384 | 232637 | 0.85 | 0.93 | 0.02 | -0.58 |
| 38 | A05 | 5828 | PXMP3 | NM_000318 | 187610 | 214262 | 0.75 | 0.86 | 0.82 | 0.01 |
| 38 | A06 | 56852 | RAD18 | NM_020165 | 206587 | 216622 | 0.83 | 0.87 | -0.94 | -0.53 |
| 38 | A07 | 5930 | RBBP6 | NM_006910 | 195783 | 208998 | 0.79 | 0.84 | 0.04 | -0.02 |
| 38 | A08 | 9618 | TRAF4 | NM_004295 | 211059 | 207171 | 0.85 | 0.83 | -1.82 | 0.37 |
| 38 | A09 | 10346 | TRIM22 | NM_006074 | 181106 | 194034 | 0.73 | 0.78 | 1.19 | 0.87 |
| 38 | A10 | 7726 | TRIM26 | NM_003449 | 199221 | 214113 | 0.80 | 0.86 | -0.50 | 0.24 |
| 38 | A11 | 81603 | TRIM8 | NM_030912 | 201642 | 207066 | 0.81 | 0.83 | -1.90 | -0.01 |
| 38 | A12 | 54904 | WHSC1L1 | NM_017778 | 198000 | 205225 | 0.79 | 0.82 | -0.02 | 1.27 |
| 38 | B01 | NA | neg | NA | 262692 | 273781 | 1.05 | 1.10 | -7.96 | -5.43 |
| 38 | B02 | NA | neg | NA | 218465 | 219278 | 0.88 | 0.88 | -1.41 | -0.62 |
| 38 | B03 | 329 | BIRC2 | NM_001166 | 225244 | 221943 | 0.90 | 0.89 | -1.34 | -0.06 |
| 38 | B04 | 330 | BIRC3 | NM_001165 | 195314 | 200621 | 0.78 | 0.80 | 4.16 | 2.19 |
| 38 | B05 | 331 | BIRC4 | NM_001167 | 217171 | 226527 | 0.87 | 0.91 | -1.79 | -1.12 |
| 38 | B06 | 2966 | GTF2H2 | NM_001515 | 210128 | 209248 | 0.84 | 0.84 | 0.30 | 0.07 |
| 38 | B07 | 8085 | MLL2 | NM_003482 | 203667 | 207900 | 0.82 | 0.83 | 0.64 | 0.02 |
| 38 | B08 | 5371 | PML | NM_002675 | 214424 | 219453 | 0.86 | 0.88 | -0.55 | -0.77 |
| 38 | B09 | 6015 | RING1 | NM_002931 | 211106 | 211757 | 0.85 | 0.85 | -1.48 | -0.74 |
| 38 | B10 | 6047 | RNF4 | NM_002938 | 193761 | 209634 | 0.78 | 0.84 | 2.07 | 0.58 |
| 38 | B11 | 10155 | TRIM28 | NM_005762 | 200337 | 192709 | 0.80 | 0.77 | 0.06 | 1.20 |
| 38 | B12 | 22954 | TRIM32 | NM_012210 | 210246 | 221063 | 0.84 | 0.89 | -0.07 | -0.18 |
| 38 | C01 | NA | pos | NA | 60120 | 73688 | 0.24 | 0.30 | 21.79 | 11.72 |
| 38 | C02 | NA | NA | NA | 233836 | 226201 | 0.94 | 0.91 | -3.93 | -1.72 |
| 38 | C03 | 997 | CDC34 | NM_004359 | 237696 | 243098 | 0.95 | 0.97 | -3.43 | -2.40 |
| 38 | C04 | 4193 | MDM2 | NM_002392 | 221475 | 191463 | 0.89 | 0.77 | 0.05 | 2.51 |
| 38 | C05 | 4194 | MDM4 | NM_002393 | 183601 | 208461 | 0.74 | 0.84 | 2.94 | -0.01 |
| 38 | C06 | 5071 | PARK2 | NM_004562 | 182063 | 205331 | 0.73 | 0.82 | 4.22 | -0.07 |
| 38 | C07 | 7321 | UBE2D1 | NM_003338 | 201445 | 187757 | 0.81 | 0.75 | 0.73 | 1.31 |
| 38 | C08 | 9354 | UBE4A | NM_004788 | 219465 | 215621 | 0.88 | 0.86 | -1.54 | -0.91 |
| 38 | C09 | 51529 | ANAPC11 | NM_016476 | 185535 | 166428 | 0.74 | 0.67 | 2.06 | 2.77 |
| 38 | C10 | 29945 | ANAPC4 | NM_013367 | 206834 | 213506 | 0.83 | 0.86 | -0.10 | -0.24 |
| 38 | C11 | 51434 | ANAPC7 | NM_016238 | 199507 | 194840 | 0.80 | 0.78 | -0.06 | 0.53 |
| 38 | C12 | 29882 | ANAPC2 | NM_013366 | 210163 | 213314 | 0.84 | 0.86 | -0.29 | 0.02 |
| 38 | D01 | NA | neg | NA | 254866 | 270124 | 1.02 | 1.08 | -7.16 | -5.81 |
| 38 | D02 | NA | neg | NA | 240080 | 222409 | 0.96 | 0.89 | -4.97 | -1.61 |
| 38 | D03 | 25820 | ARIH1 | NM_005744 | 213663 | 220769 | 0.86 | 0.89 | 0.02 | -0.66 |
| 38 | D04 | 51283 | BFAR | NM_016561 | 221139 | 205387 | 0.89 | 0.82 | -0.02 | 1.06 |
| 38 | D05 | 51317 | BHC80 | NM_016621 | 212994 | 204564 | 0.85 | 0.82 | -1.52 | 0.11 |
| 38 | D06 | 112401 | BIRC8 | NM_033341 | 231027 | 190006 | 0.93 | 0.76 | -3.15 | 1.06 |
| 38 | D07 | 648 | BMI1 | NM_005180 | 192713 | 182189 | 0.77 | 0.73 | 1.91 | 1.58 |
| 38 | D08 | 672 | BRCA1 | NM_007294 | 180686 | 203821 | 0.72 | 0.82 | 4.09 | -0.09 |
| 38 | D09 | 8945 | BTRC | NM_003939 | 194157 | 193379 | 0.78 | 0.78 | 0.67 | 0.17 |
| 38 | D10 | 10616 | C20ORF18 | NM_006462 | 204731 | 211256 | 0.82 | 0.85 | 0.10 | -0.27 |
| 38 | D11 | 23304 | C6ORF133 | NM_015255 | 208841 | 214717 | 0.84 | 0.86 | -1.55 | -1.44 |
| 38 | D12 | 23624 | CBLC | NM_012116 | 208054 | 214535 | 0.83 | 0.86 | -0.10 | -0.31 |

| | | | | | | | | | | |
|---|---|---|---|---|---|---|---|---|---|---|
| 38 | E01 | NA | neg | NA | 248923 | 272476 | 1.00 | 1.09 | -7.19 | -5.44 |
| 38 | E02 | NA | neg | NA | 232966 | 227484 | 0.93 | 0.91 | -4.83 | -1.47 |
| 38 | E03 | 51191 | HERC5 | NM_016323 | 213344 | 202147 | 0.86 | 0.81 | -0.85 | 1.56 |
| 38 | E04 | 1108 | CHD4 | NM_001273 | 214536 | 224238 | 0.86 | 0.90 | 0.05 | -0.02 |
| 38 | E05 | 55743 | CHFR | NM_018223 | 195240 | 187875 | 0.78 | 0.75 | 0.19 | 2.16 |
| 38 | E06 | 4850 | CNOT4 | NM_013316 | 189674 | 224263 | 0.76 | 0.90 | 2.06 | -1.38 |
| 38 | E07 | 51366 | DD5 | NM_015902 | 202594 | 220791 | 0.81 | 0.89 | -0.47 | -1.24 |
| 38 | E08 | 26091 | HERC4 | NM_015601 | 193432 | 182994 | 0.78 | 0.73 | 1.29 | 2.32 |
| 38 | E09 | 22992 | FBXL11 | NM_012308 | 215487 | 203843 | 0.86 | 0.82 | -3.40 | -0.17 |
| 38 | E10 | 25827 | FBXL2 | NM_012157 | 199854 | 219133 | 0.80 | 0.88 | -0.10 | -0.38 |
| 38 | E11 | 26224 | FBXL3A | NM_012158 | 192572 | 204761 | 0.77 | 0.82 | -0.06 | 0.01 |
| 38 | E12 | 26223 | FBXL3P | NM_012159 | 198847 | 215977 | 0.80 | 0.87 | 0.35 | 0.14 |
| 38 | F01 | NA | NA | NA | 247835 | 250384 | 0.99 | 1.00 | -6.68 | -3.84 |
| 38 | F02 | NA | pos | NA | 79017 | 76828 | 0.32 | 0.31 | 18.31 | 11.46 |
| 38 | F03 | 26235 | FBXL4 | NM_012160 | 210056 | 205940 | 0.84 | 0.83 | -0.02 | 0.88 |
| 38 | F04 | 26234 | FBXL5 | NM_012161 | 220201 | 219800 | 0.88 | 0.88 | -0.45 | 0.02 |
| 38 | F05 | 26233 | FBXL6 | NM_012162 | 226088 | 227141 | 0.91 | 0.91 | -4.03 | -1.65 |
| 38 | F06 | 23194 | FBXL7 | NM_012304 | 206142 | 200930 | 0.83 | 0.81 | -0.03 | 0.33 |
| 38 | F07 | 26231 | FBXL9 | NM_012163 | 213603 | 193211 | 0.86 | 0.77 | -1.75 | 0.84 |
| 38 | F08 | 26267 | FBXO10 | XM_291314 | 189210 | 204280 | 0.76 | 0.82 | 2.26 | 0.09 |
| 38 | F09 | 80204 | FBXO11 | NM_012167 | 194182 | 203658 | 0.78 | 0.82 | 0.10 | -0.50 |
| 38 | F10 | 26232 | FBXO2 | NM_012168 | 199314 | 224681 | 0.80 | 0.90 | 0.33 | -1.22 |
| 38 | F11 | 26263 | FBXO22 | NM_012170 | 191932 | 202919 | 0.77 | 0.81 | 0.38 | -0.17 |
| 38 | F12 | 26260 | FBXO25 | NM_012173 | 203452 | 213867 | 0.82 | 0.86 | 0.02 | -0.02 |
| 38 | G01 | NA | neg | NA | 249569 | 254672 | 1.00 | 1.02 | -6.63 | -4.57 |
| 38 | G02 | NA | neg | NA | 267799 | 244157 | 1.07 | 0.98 | -9.33 | -3.64 |
| 38 | G03 | 26273 | FBXO3 | NM_012175 | 211241 | 211326 | 0.85 | 0.85 | 0.11 | 0.06 |
| 38 | G04 | 26272 | FBXO4 | NM_012176 | 229759 | 227753 | 0.92 | 0.91 | -1.55 | -1.03 |
| 38 | G05 | 26271 | FBXO5 | NM_012177 | 193987 | 190866 | 0.78 | 0.77 | 1.03 | 1.20 |
| 38 | G06 | 26270 | FBXO6 | NM_018438 | 209055 | 195772 | 0.84 | 0.78 | -0.15 | 0.43 |
| 38 | G07 | 25793 | FBXO7 | NM_012179 | 211067 | 221612 | 0.85 | 0.89 | -1.07 | -2.01 |
| 38 | G08 | 26268 | FBXO9 | NM_012347 | 202796 | 205095 | 0.81 | 0.82 | 0.55 | -0.33 |
| 38 | G09 | 26190 | FBXW2 | NM_012164 | 197611 | 190562 | 0.79 | 0.76 | -0.10 | 0.30 |
| 38 | G10 | 26226 | SHFM3P1 | AF174606 | 207795 | 191919 | 0.83 | 0.77 | -0.62 | 1.32 |
| 38 | G11 | 55294 | FBXW7 | NM_018315 | 192386 | 197862 | 0.77 | 0.79 | 0.62 | -0.08 |
| 38 | G12 | 26259 | FBXW8 | NM_012174 | 202861 | 212136 | 0.81 | 0.85 | 0.41 | -0.22 |
| 38 | H01 | NA | NA | NA | 259768 | 264607 | 1.04 | 1.06 | -5.74 | -2.94 |
| 38 | H02 | NA | pos | NA | 69343 | 83176 | 0.28 | 0.33 | 22.45 | 13.05 |
| 38 | H03 | 65259 | FLJ23360 | NM_023076 | 218420 | 206305 | 0.88 | 0.83 | 1.45 | 3.00 |
| 38 | H04 | 51343 | FZR1 | NM_016263 | 242588 | 269465 | 0.97 | 1.08 | -1.05 | -2.21 |
| 38 | H05 | 25831 | HECTD1 | NM_015382 | 218468 | 257466 | 0.88 | 1.03 | -0.19 | -2.17 |
| 38 | H06 | 8925 | HERC1 | NM_003922 | 224055 | 233849 | 0.90 | 0.94 | 0.03 | -0.42 |
| 38 | H07 | 83737 | ITCH | NM_031483 | 220314 | 240892 | 0.88 | 0.97 | -0.04 | -1.21 |
| 38 | H08 | 9690 | UBE3C | NM_014671 | 240657 | 221834 | 0.97 | 0.89 | -2.65 | 0.70 |
| 38 | H09 | 23072 | NEDL1 | NM_015052 | 214310 | 229164 | 0.86 | 0.92 | -0.17 | -0.60 |
| 38 | H10 | 220972 | MIR | NM_145021 | 206680 | 222540 | 0.83 | 0.89 | 1.95 | 1.12 |
| 38 | H11 | 58508 | MLL3 | NM_021230 | 195950 | 208447 | 0.79 | 0.84 | 2.50 | 1.49 |
| 38 | H12 | 4734 | NEDD4 | NM_006154 | 217258 | 233232 | 0.87 | 0.94 | 0.68 | 0.42 |
| 39 | A01 | NA | pos | NA | 54766 | 53363 | 0.24 | 0.24 | 17.98 | 18.14 |
| 39 | A02 | NA | NA | NA | 199703 | 200970 | 0.88 | 0.89 | -1.10 | -0.12 |
| 39 | A03 | 23327 | NEDD4L | NM_015277 | 193594 | 204503 | 0.85 | 0.90 | 0.82 | 0.21 |
| 39 | A04 | 4738 | NEDD8 | NM_006156 | 213070 | 220227 | 0.94 | 0.97 | -1.68 | -0.80 |
| 39 | A05 | 9148 | NEURL | NM_004210 | 200730 | 197748 | 0.88 | 0.87 | 0.03 | 0.14 |
| 39 | A06 | 23113 | PARC | NM_015089 | 190654 | 204217 | 0.84 | 0.90 | 0.19 | -0.40 |
| 39 | A07 | 5888 | RAD51 | NM_002875 | 167111 | 174527 | 0.74 | 0.77 | 3.09 | 2.95 |
| 39 | A08 | 5913 | RAPSN | NM_005055 | 177095 | 184690 | 0.78 | 0.82 | 1.62 | 1.00 |
| 39 | A09 | 9978 | RBX1 | NM_014248 | 197311 | 203544 | 0.87 | 0.90 | -1.03 | -0.13 |
| 39 | A10 | 10206 | RFP2 | NM_005798 | 195959 | 207596 | 0.86 | 0.92 | -1.15 | -1.10 |
| 39 | A11 | 7703 | RNF110 | NM_007144 | 189416 | 201602 | 0.83 | 0.89 | -0.01 | -0.31 |
| 39 | A12 | 11236 | RNF139 | NM_007218 | 209507 | 202154 | 0.92 | 0.89 | -1.22 | 0.40 |
| 39 | B01 | NA | neg | NA | 236935 | 247508 | 1.04 | 1.09 | -4.60 | -4.06 |
| 39 | B02 | NA | neg | NA | 226175 | 223658 | 1.00 | 0.99 | -3.19 | -1.11 |
| 39 | B03 | 9781 | RNF144 | NM_014746 | 201992 | 222195 | 0.89 | 0.98 | 1.11 | -0.17 |
| 39 | B04 | 55072 | RNF31 | NM_017999 | 225420 | 228465 | 0.99 | 1.01 | -1.91 | -0.01 |
| 39 | B05 | 140545 | RNF32 | NM_030936 | 206771 | 213424 | 0.91 | 0.94 | 0.63 | 0.01 |
| 39 | B06 | 152006 | RNF38 | NM_022781 | 202675 | 223046 | 0.89 | 0.99 | 0.00 | -0.91 |
| 39 | B07 | 6049 | RNF6 | NM_005977 | 201572 | 217395 | 0.89 | 0.96 | -0.05 | -0.54 |
| 39 | B08 | 9616 | RNF7 | NM_014245 | 209338 | 201762 | 0.92 | 0.89 | -1.23 | 0.70 |
| 39 | B09 | 9025 | RNF8 | NM_003958 | 191253 | 205918 | 0.84 | 0.91 | 1.16 | 1.39 |
| 39 | B10 | 6468 | SHFM3 | NM_022039 | 197895 | 200025 | 0.87 | 0.88 | -0.01 | 1.65 |
| 39 | B11 | 6477 | SIAH1 | NM_003031 | 182340 | 215728 | 0.80 | 0.95 | 2.32 | -0.24 |
| 39 | B12 | 6500 | SKP1A | NM_006930 | 219863 | 205448 | 0.97 | 0.91 | -1.19 | 1.81 |
| 39 | C01 | NA | pos | NA | 59286 | 64569 | 0.26 | 0.29 | 19.30 | 17.88 |
| 39 | C02 | NA | NA | NA | 220883 | 212241 | 0.97 | 0.94 | -1.97 | -0.38 |
| 39 | C03 | 6502 | SKP2 | NM_005983 | 214269 | 211019 | 0.94 | 0.93 | 0.01 | 0.53 |
| 39 | C04 | 6596 | SMARCA3 | NM_003071 | 215626 | 236532 | 0.95 | 1.05 | -0.10 | -1.69 |
| 39 | C05 | 57154 | SMURF1 | NM_020429 | 216881 | 178929 | 0.96 | 0.79 | -0.18 | 3.59 |
| 39 | C06 | 64750 | SMURF2 | NM_022739 | 206702 | 190159 | 0.91 | 0.84 | 0.00 | 2.47 |
| 39 | C07 | 6737 | SSA1 | NM_003141 | 183277 | 199243 | 0.81 | 0.88 | 2.88 | 1.02 |
| 39 | C08 | 10273 | STUB1 | NM_005861 | 196011 | 227966 | 0.86 | 1.01 | 1.05 | -3.23 |
| 39 | C09 | 6921 | TCEB1 | NM_005648 | 208679 | 225720 | 0.92 | 1.00 | -0.61 | -1.75 |
| 39 | C10 | 10210 | TOPORS | NM_005802 | 209440 | 212065 | 0.92 | 0.94 | -1.00 | -0.53 |
| 39 | C11 | 54476 | TRIAD3 | NM_207111 | 203813 | 201074 | 0.90 | 0.89 | 0.01 | 0.88 |
| 39 | C12 | 51592 | TRIM33 | NM_015906 | 212154 | 222346 | 0.93 | 0.98 | 0.35 | -0.97 |
| 39 | D01 | NA | neg | NA | 207520 | 231259 | 0.91 | 1.02 | -1.17 | -2.85 |
| 39 | D02 | NA | neg | NA | 227662 | 228479 | 1.00 | 1.01 | -3.82 | -2.50 |
| 39 | D03 | 4591 | TRIM37 | NM_015294 | 219744 | 219008 | 0.97 | 0.97 | -1.67 | -0.57 |
| 39 | D04 | 10293 | TRIP | NM_005879 | 190590 | 224455 | 0.84 | 0.99 | 2.23 | -0.31 |

| | | | | | | | | | | |
|---|---|---|---|---|---|---|---|---|---|---|
| 39 | D05 | 22888 | UBCE7IP5 | NM_014948 | 208375 | 207183 | 0.92 | 0.92 | -0.03 | -0.01 |
| 39 | D06 | 89910 | UBE3B | NM_130466 | 213715 | 208761 | 0.94 | 0.92 | -1.89 | 0.05 |
| 39 | D07 | 10277 | UBE4B | NM_006048 | 196938 | 218270 | 0.87 | 0.97 | 0.12 | -1.45 |
| 39 | D08 | 197131 | UBR1 | NM_174916 | 196394 | 220679 | 0.87 | 0.98 | 0.04 | -2.44 |
| 39 | D09 | 29128 | UHRF1 | NM_013282 | 198501 | 204248 | 0.87 | 0.90 | -0.23 | 0.79 |
| 39 | D10 | 7468 | WHSC1 | NM_007331 | 194168 | 203433 | 0.86 | 0.90 | 0.05 | 0.43 |
| 39 | D11 | 11059 | WWP1 | NM_007013 | 211102 | 207138 | 0.93 | 0.92 | -1.91 | 0.02 |
| 39 | D12 | 11060 | WWP2 | NM_007014 | 205712 | 210651 | 0.91 | 0.93 | 0.24 | 0.37 |
| 39 | E01 | NA | neg | NA | 237229 | 265365 | 1.05 | 1.17 | -5.52 | -8.42 |
| 39 | E02 | NA | neg | NA | 216460 | 221604 | 0.95 | 0.98 | -2.78 | -3.01 |
| 39 | E03 | 8924 | HERC2 | NM_004667 | 203830 | 218564 | 0.90 | 0.97 | -0.01 | -1.87 |
| 39 | E04 | 4799 | NFX1 | NM_002504 | 201609 | 210925 | 0.89 | 0.93 | 0.35 | 0.01 |
| 39 | E05 | 9604 | RNF14 | NM_004290 | 195490 | 214267 | 0.86 | 0.95 | 1.24 | -2.25 |
| 39 | E06 | 6478 | SIAH2 | NM_005067 | 193818 | 195361 | 0.85 | 0.86 | 0.29 | 0.35 |
| 39 | E07 | 267 | AMFR | NM_001144 | 197778 | 195625 | 0.87 | 0.87 | -0.42 | 0.00 |
| 39 | E08 | 9320 | TRIP12 | NM_004238 | 211297 | 182972 | 0.93 | 0.81 | -2.36 | 0.86 |
| 39 | E09 | 867 | CBL | NM_005188 | 183581 | 198618 | 0.81 | 0.88 | 1.30 | 0.13 |
| 39 | E10 | 7186 | TRAF2 | NM_021138 | 192402 | 196201 | 0.85 | 0.87 | -0.16 | -0.03 |
| 39 | E11 | 7187 | TRAF3 | NM_003300 | 194176 | 192512 | 0.86 | 0.85 | -0.12 | 0.47 |
| 39 | E12 | 7188 | TRAF5 | NM_004619 | 204109 | 226014 | 0.90 | 1.00 | 0.01 | -2.89 |
| 39 | F01 | NA | NA | NA | 217441 | 241211 | 0.96 | 1.07 | -2.71 | -4.86 |
| 39 | F02 | NA | pos | NA | 67170 | 98103 | 0.30 | 0.43 | 17.07 | 12.84 |
| 39 | F03 | 7189 | TRAF6 | NM_004620 | 211691 | 217824 | 0.93 | 0.96 | -0.84 | -1.20 |
| 39 | F04 | 1840 | DTX1 | NM_004416 | 190451 | 187868 | 0.84 | 0.83 | 2.02 | 3.44 |
| 39 | F05 | 8805 | TIF1 | NM_003852 | 197494 | 200152 | 0.87 | 0.89 | 1.17 | 0.08 |
| 39 | F06 | 89970 | KIAA1972 | NM_133368 | 204666 | 190977 | 0.90 | 0.84 | -0.93 | 1.47 |
| 39 | F07 | 1813 | DRD2 | NM_000795 | 195720 | 191273 | 0.86 | 0.85 | 0.05 | 1.12 |
| 39 | F08 | 1814 | DRD3 | NM_000796 | 191556 | 203985 | 0.84 | 0.90 | 0.44 | -1.15 |
| 39 | F09 | 1815 | DRD4 | NM_000797 | 198721 | 219280 | 0.88 | 0.97 | -0.49 | -1.84 |
| 39 | F10 | 30817 | EMR2 | NM_013447 | 192699 | 186240 | 0.85 | 0.82 | 0.01 | 1.78 |
| 39 | F11 | 3357 | HTR2B | NM_000867 | 206836 | 208848 | 0.91 | 0.92 | -1.58 | -0.97 |
| 39 | F12 | 26245 | OR2M4 | XM_371358 | 205807 | 207922 | 0.91 | 0.92 | -0.01 | -0.07 |
| 39 | G01 | NA | neg | NA | 234282 | 216887 | 1.03 | 0.96 | -4.43 | -0.89 |
| 39 | G02 | NA | neg | NA | 207113 | 222620 | 0.91 | 0.98 | -0.85 | -1.60 |
| 39 | G03 | 5020 | OXT | NM_000915 | 209543 | 214497 | 0.92 | 0.95 | -0.06 | 0.17 |
| 39 | G04 | 5029 | P2RY2 | NM_002564 | 208798 | 207063 | 0.92 | 0.92 | 0.10 | 2.02 |
| 39 | G05 | 9052 | RAI3 | NM_003979 | 219929 | 213384 | 0.97 | 0.94 | -1.28 | -0.60 |
| 39 | G06 | 6745 | SSR1 | NM_003144 | 199273 | 211093 | 0.88 | 0.93 | 0.28 | -0.05 |
| 39 | G07 | 6746 | SSR2 | NM_003145 | 213555 | 208079 | 0.94 | 0.92 | -1.80 | 0.00 |
| 39 | G08 | 1133 | CHRM5 | NM_012125 | 202247 | 202436 | 0.89 | 0.90 | -0.47 | 0.00 |
| 39 | G09 | 887 | CCKBR | NM_176875 | 191764 | 219728 | 0.85 | 0.97 | 0.92 | -0.94 |
| 39 | G10 | 221188 | GPR114 | NM_153837 | 196048 | 208356 | 0.86 | 0.92 | 0.06 | 0.00 |
| 39 | G11 | 59352 | LGR6 | XM_097508 | 186334 | 193352 | 0.82 | 0.86 | 1.61 | 1.91 |
| 39 | G12 | 26492 | OR8G2 | XM_370662 | 213001 | 214491 | 0.94 | 0.95 | -0.46 | 0.07 |
| 39 | H01 | NA | NA | NA | 260448 | 248976 | 1.15 | 1.10 | -5.92 | -3.83 |
| 39 | H02 | NA | pos | NA | 61725 | 85387 | 0.27 | 0.38 | 20.24 | 16.40 |
| 39 | H03 | 338557 | GPR120 | NM_181745 | 223530 | 213292 | 0.99 | 0.94 | 0.05 | 1.35 |
| 39 | H04 | 131601 | TPRA40 | NM_016372 | 261208 | 228517 | 1.15 | 1.01 | -4.85 | 0.40 |
| 39 | H05 | 886 | CCKAR | NM_000730 | 233771 | 220748 | 1.03 | 0.98 | -1.15 | -0.48 |
| 39 | H06 | 1132 | CHRM4 | NM_000741 | 226830 | 236458 | 1.00 | 1.05 | -1.40 | -2.16 |
| 39 | H07 | 2587 | GALR1 | NM_001480 | 215784 | 216366 | 0.95 | 0.96 | -0.14 | 0.00 |
| 39 | H08 | 8484 | GALR3 | NM_003614 | 213821 | 210718 | 0.94 | 0.93 | -0.04 | 0.00 |
| 39 | H09 | 3060 | HCRT | NM_001524 | 211826 | 216119 | 0.93 | 0.96 | 0.23 | 0.54 |
| 39 | H10 | 3061 | HCRTR1 | NM_001525 | 207499 | 216721 | 0.91 | 0.96 | 0.51 | 0.00 |
| 39 | H11 | 3062 | HCRTR2 | NM_001526 | 208355 | 217251 | 0.92 | 0.96 | 0.67 | -0.02 |
| 39 | H12 | 51454 | GULP1 | NM_016315 | 218181 | 235354 | 0.96 | 1.04 | 0.81 | -1.48 |
| 40 | A01 | NA | pos | NA | 58389 | 59829 | 0.26 | 0.27 | 19.84 | 26.55 |
| 40 | A02 | NA | NA | NA | 202950 | 226737 | 0.89 | 1.03 | -0.57 | -6.19 |
| 40 | A03 | 1062 | CENPE | NM_001813 | 197085 | 198478 | 0.87 | 0.90 | 0.15 | 0.29 |
| 40 | A04 | 26153 | KIF26A | XM_050278 | 196909 | 198454 | 0.87 | 0.90 | 0.83 | 0.69 |
| 40 | A05 | 55614 | C20ORF23 | NM_017683 | 194286 | 205751 | 0.86 | 0.94 | 2.18 | -0.16 |
| 40 | A06 | 3832 | KIF11 | NM_004523 | 173001 | 177784 | 0.76 | 0.81 | 3.89 | 5.43 |
| 40 | A07 | 113220 | KIF12 | NM_138424 | 199724 | 200226 | 0.88 | 0.91 | 0.00 | -0.67 |
| 40 | A08 | 63971 | KIF13A | NM_022113 | 192090 | 195412 | 0.85 | 0.89 | 0.00 | -0.37 |
| 40 | A09 | 10749 | KIF1C | NM_006612 | 199034 | 196938 | 0.88 | 0.90 | -1.08 | -1.47 |
| 40 | A10 | 3796 | KIF2 | NM_004520 | 200205 | 191457 | 0.88 | 0.87 | -1.75 | -0.79 |
| 40 | A11 | 55605 | KIF21A | NM_017641 | 194542 | 180810 | 0.86 | 0.82 | -0.08 | 0.14 |
| 40 | A12 | 3835 | KIF22 | NM_007317 | 201743 | 191656 | 0.89 | 0.87 | -0.10 | 0.28 |
| 40 | B01 | NA | neg | NA | 241495 | 245970 | 1.06 | 1.12 | -4.25 | -6.61 |
| 40 | B02 | NA | neg | NA | 221101 | 218041 | 0.97 | 0.99 | -1.37 | -1.13 |
| 40 | B03 | 3834 | KIF25 | NM_005355 | 222884 | 224316 | 0.98 | 1.02 | -1.73 | -1.42 |
| 40 | B04 | 11004 | KIF2C | NM_006845 | 215235 | 216053 | 0.95 | 0.98 | 0.00 | 0.60 |
| 40 | B05 | 11127 | KIF3A | NM_007054 | 235994 | 221417 | 1.04 | 1.01 | -1.95 | 0.13 |
| 40 | B06 | 3797 | KIF3C | NM_002254 | 204696 | 206287 | 0.90 | 0.94 | 1.18 | 3.19 |
| 40 | B07 | 24137 | KIF4A | NM_012310 | 206957 | 210488 | 0.91 | 0.96 | 0.73 | 0.67 |
| 40 | B08 | 3799 | KIF5B | NM_004521 | 204569 | 212584 | 0.90 | 0.97 | 0.00 | -0.38 |
| 40 | B09 | 3800 | KIF5C | XM_377774 | 197843 | 211286 | 0.87 | 0.96 | 0.84 | -0.93 |
| 40 | B10 | 64147 | KIF9 | NM_022342 | 186150 | 192575 | 0.82 | 0.88 | 1.99 | 2.35 |
| 40 | B11 | 3833 | KIFC1 | XM_371813 | 208594 | 199385 | 0.92 | 0.91 | -0.31 | -0.14 |
| 40 | B12 | 3801 | KIFC3 | NM_005550 | 219973 | 211486 | 0.97 | 0.96 | -0.92 | -0.25 |
| 40 | C01 | NA | pos | NA | 63249 | 64711 | 0.28 | 0.29 | 20.69 | 27.76 |
| 40 | C02 | NA | NA | NA | 216107 | 216430 | 0.95 | 0.99 | -0.90 | -2.00 |
| 40 | C03 | 56992 | KNSL7 | NM_020242 | 213331 | 210840 | 0.94 | 0.96 | -0.61 | 0.04 |
| 40 | C04 | 84643 | LOC84643 | NM_032559 | 223092 | 220101 | 0.98 | 1.00 | -1.33 | -1.39 |
| 40 | C05 | 23095 | KIF1B | NM_015074 | 223876 | 230092 | 0.99 | 1.05 | -0.47 | -2.76 |
| 40 | C06 | 9585 | MPHOSPH1 | NM_016195 | 218365 | 218718 | 0.96 | 1.00 | -0.98 | -0.44 |
| 40 | C07 | 547 | KIF1A | NM_004321 | 183025 | 199813 | 0.81 | 0.91 | 3.89 | 1.58 |
| 40 | C08 | 9928 | KIF14 | NM_014875 | 191291 | 204516 | 0.84 | 0.93 | 1.64 | 0.01 |

| | | | | | | | | | | |
|---|---|---|---|---|---|---|---|---|---|---|
| 40 | C09 | 57576 | KIF17 | NM_020816 | 181891 | 200535 | 0.80 | 0.91 | 2.87 | -0.01 |
| 40 | C10 | 10112 | KIF20A | NM_005733 | 195328 | 194256 | 0.86 | 0.88 | 0.47 | 0.83 |
| 40 | C11 | 9493 | KIF23 | NM_004856 | 197886 | 188966 | 0.87 | 0.86 | 0.97 | 0.71 |
| 40 | C12 | 3798 | KIF5A | NM_004984 | 225717 | 207358 | 0.99 | 0.94 | -1.95 | -0.63 |
| 40 | D01 | NA | neg | NA | 243708 | 220509 | 1.07 | 1.00 | -6.18 | -2.55 |
| 40 | D02 | NA | neg | NA | 221317 | 218583 | 0.97 | 1.00 | -3.02 | -2.17 |
| 40 | D03 | 81930 | KIF18A | NM_031217 | 176969 | 169279 | 0.78 | 0.77 | 3.14 | 8.44 |
| 40 | D04 | 9371 | KIF3B | NM_004798 | 203847 | 216804 | 0.90 | 0.99 | 0.00 | -0.49 |
| 40 | D05 | 54 | ACP5 | NM_001611 | 218770 | 217232 | 0.96 | 0.99 | -1.13 | 0.01 |
| 40 | D06 | 5530 | PPP3CA | NM_000944 | 200844 | 206192 | 0.88 | 0.94 | 0.11 | 2.28 |
| 40 | D07 | 10519 | CIB1 | NM_006384 | 200725 | 197387 | 0.88 | 0.90 | 0.00 | 2.31 |
| 40 | D08 | 5535 | PPP3R2 | NM_147180 | 182332 | 191751 | 0.80 | 0.87 | 1.53 | 2.77 |
| 40 | D09 | 5723 | PSPH | NM_004577 | 185288 | 209035 | 0.82 | 0.95 | 1.00 | -1.42 |
| 40 | D10 | 52 | ACP1 | NM_004300 | 192921 | 209878 | 0.85 | 0.96 | -0.58 | -1.98 |
| 40 | D11 | 55 | ACPP | NM_001099 | 206089 | 208485 | 0.91 | 0.95 | -1.57 | -2.86 |
| 40 | D12 | 8556 | CDC14A | NM_003672 | 215011 | 205509 | 0.95 | 0.94 | -1.83 | -0.01 |
| 40 | E01 | NA | neg | NA | 225086 | 245369 | 0.99 | 1.12 | -3.69 | -9.16 |
| 40 | E02 | NA | neg | NA | 229207 | 214003 | 1.01 | 0.97 | -4.27 | -3.00 |
| 40 | E03 | 8555 | CDC14B | NM_003671 | 197121 | 205143 | 0.87 | 0.93 | 0.15 | -0.32 |
| 40 | E04 | 993 | CDC25A | NM_001789 | 197156 | 210450 | 0.87 | 0.96 | 0.80 | -0.97 |
| 40 | E05 | 994 | CDC25B | NM_004358 | 201732 | 209118 | 0.89 | 0.95 | 1.14 | -0.13 |
| 40 | E06 | 995 | CDC25C | NM_001790 | 209696 | 222419 | 0.92 | 1.01 | -1.28 | -2.64 |
| 40 | E07 | 58190 | CTDSP1 | NM_021198 | 203570 | 184260 | 0.90 | 0.84 | -0.54 | 3.15 |
| 40 | E08 | 8446 | DUSP11 | NM_003584 | 193215 | 194674 | 0.85 | 0.89 | -0.15 | 0.46 |
| 40 | E09 | 7957 | EPM2A | NM_005670 | 193833 | 192353 | 0.85 | 0.88 | -0.34 | 0.12 |
| 40 | E10 | 3636 | INPPL1 | NM_001567 | 185848 | 201891 | 0.82 | 0.92 | 0.28 | -2.15 |
| 40 | E11 | 114971 | LOC114971 | XM_374879 | 200722 | 181279 | 0.88 | 0.83 | -0.95 | 0.74 |
| 40 | E12 | 333926 | PPP2CZ | NM_005167 | 194946 | 179031 | 0.86 | 0.82 | 0.87 | 3.45 |
| 40 | F01 | NA | NA | NA | 238792 | 215099 | 1.05 | 0.98 | -5.00 | -2.73 |
| 40 | F02 | NA | pos | NA | 80747 | 80095 | 0.36 | 0.36 | 17.31 | 23.76 |
| 40 | F03 | 4534 | MTM1 | NM_000252 | 202408 | 206188 | 0.89 | 0.94 | 0.03 | -0.04 |
| 40 | F04 | 8613 | PPAP2B | NM_003713 | 207402 | 222054 | 0.91 | 1.01 | -0.02 | -2.76 |
| 40 | F05 | 8612 | PPAP2C | NM_003712 | 212345 | 208583 | 0.93 | 0.95 | 0.26 | 0.47 |
| 40 | F06 | 8493 | PPM1D | NM_003620 | 203209 | 212491 | 0.89 | 0.97 | 0.26 | -0.20 |
| 40 | F07 | 5507 | PPP1R3C | NM_005398 | 209090 | 217110 | 0.92 | 0.99 | -0.70 | -2.80 |
| 40 | F08 | 8781 | PSPHL | BC065228 | 208384 | 199572 | 0.92 | 0.91 | -1.67 | -0.01 |
| 40 | F09 | 5728 | PTEN | NM_000314 | 199907 | 188436 | 0.88 | 0.86 | -0.58 | 1.38 |
| 40 | F10 | 11191 | PTENP1 | BC038293 | 194223 | 192649 | 0.86 | 0.88 | -0.28 | 0.15 |
| 40 | F11 | 5784 | PTPN14 | NM_005401 | 197801 | 183696 | 0.87 | 0.84 | 0.08 | 0.76 |
| 40 | F12 | 10076 | PTPRU | NM_005704 | 191560 | 199041 | 0.84 | 0.91 | 1.96 | 0.01 |
| 40 | G01 | NA | neg | NA | 215568 | 230522 | 0.95 | 1.05 | -1.42 | -5.47 |
| 40 | G02 | NA | neg | NA | 244638 | 211401 | 1.08 | 0.96 | -5.52 | -1.72 |
| 40 | G03 | 93492 | TPTE2 | NM_130785 | 204936 | 202771 | 0.90 | 0.92 | -0.03 | 0.92 |
| 40 | G04 | 9150 | CTDP1 | NM_004715 | 209164 | 206963 | 0.92 | 0.94 | 0.03 | 0.49 |
| 40 | G05 | 57171 | DOLPP1 | NM_020438 | 213869 | 212467 | 0.94 | 0.97 | 0.35 | -0.01 |
| 40 | G06 | 11266 | DUSP12 | NM_007240 | 212378 | 214106 | 0.93 | 0.98 | -0.73 | -0.24 |
| 40 | G07 | 51207 | DUSP13 | NM_016364 | 214002 | 221465 | 0.94 | 1.01 | -1.09 | -3.38 |
| 40 | G08 | 11072 | DUSP14 | NM_007026 | 199033 | 203419 | 0.88 | 0.93 | -0.05 | -0.48 |
| 40 | G09 | 128853 | DUSP15 | NM_080611 | 208212 | 196869 | 0.92 | 0.90 | -1.45 | 0.01 |
| 40 | G10 | 80824 | DUSP16 | NM_030640 | 177652 | 175399 | 0.78 | 0.80 | 2.36 | 3.82 |
| 40 | G11 | 150290 | DUSP18 | NM_152511 | 190385 | 196482 | 0.84 | 0.89 | 1.43 | -1.47 |
| 40 | G12 | 142679 | DUSP19 | NM_080876 | 203390 | 197164 | 0.90 | 0.90 | 0.60 | 0.67 |
| 40 | H01 | NA | NA | NA | 231649 | 216750 | 1.02 | 0.99 | -1.70 | -0.68 |
| 40 | H02 | NA | pos | NA | 57491 | 64230 | 0.25 | 0.29 | 22.89 | 29.24 |
| 40 | H03 | 63904 | DUSP21 | NM_022076 | 220510 | 222054 | 0.97 | 1.01 | -0.24 | -0.78 |
| 40 | H04 | 1845 | DUSP3 | NM_004090 | 224432 | 216656 | 0.99 | 0.99 | -0.13 | 0.67 |
| 40 | H05 | 1852 | DUSP9 | NM_001395 | 232249 | 203099 | 1.02 | 0.93 | -0.26 | 3.92 |
| 40 | H06 | 23770 | FKBP8 | NM_012181 | 222046 | 222524 | 0.98 | 1.01 | -0.11 | 0.20 |
| 40 | H07 | 79948 | FLJ11535 | NM_024888 | 214872 | 220504 | 0.95 | 1.00 | 0.78 | -1.10 |
| 40 | H08 | 54935 | DUSP23 | NM_017823 | 200836 | 190704 | 0.88 | 0.87 | 1.69 | 4.10 |
| 40 | H09 | 2819 | GPD1 | NM_005276 | 209616 | 206746 | 0.92 | 0.94 | 0.34 | 0.16 |
| 40 | H10 | 57460 | PPM1H | XM_051093 | 211311 | 206313 | 0.93 | 0.94 | -0.40 | -0.15 |
| 40 | H11 | 78986 | MGC1136 | NM_024025 | 207589 | 212137 | 0.91 | 0.97 | 0.99 | -2.45 |
| 40 | H12 | 168448 | MGC26484 | XM_171149 | 221008 | 216730 | 0.97 | 0.99 | 0.10 | -1.08 |
| 41 | A01 | NA | pos | NA | 52435 | 67060 | 0.23 | 0.27 | 24.46 | 16.92 |
| 41 | A02 | NA | NA | NA | 246240 | 257856 | 1.10 | 1.02 | -5.46 | -2.77 |
| 41 | A03 | 51657 | MK-STYX | NM_016086 | 214828 | 232345 | 0.96 | 0.92 | -1.63 | 0.30 |
| 41 | A04 | 8776 | MTMR1 | NM_003828 | 203699 | 243816 | 0.91 | 0.96 | 1.54 | -0.29 |
| 41 | A05 | 8898 | MTMR2 | NM_016156 | 206596 | 251487 | 0.92 | 0.99 | 3.52 | -1.67 |
| 41 | A06 | 9110 | MTMR4 | NM_004687 | 227546 | 218514 | 1.02 | 0.86 | -0.08 | 1.07 |
| 41 | A07 | 9107 | MTMR6 | NM_004685 | 210763 | 255093 | 0.94 | 1.01 | 0.46 | -2.46 |
| 41 | A08 | 9108 | MTMR7 | XM_044727 | 213993 | 228527 | 0.96 | 0.90 | 0.08 | 0.97 |
| 41 | A09 | 55613 | MTMR8 | NM_017677 | 191505 | 225523 | 0.86 | 0.89 | 2.18 | -0.23 |
| 41 | A10 | 57546 | PDP2 | NM_020786 | 205135 | 223300 | 0.92 | 0.88 | -0.54 | -0.02 |
| 41 | A11 | 23239 | PLEKHE1 | NM_194449 | 213687 | 211514 | 0.96 | 0.84 | -2.30 | 0.48 |
| 41 | A12 | 5475 | PPEF1 | NM_006240 | 212475 | 230556 | 0.95 | 0.91 | -0.70 | 0.01 |
| 41 | B01 | NA | neg | NA | 219961 | 269917 | 0.98 | 1.07 | -0.93 | -3.94 |
| 41 | B02 | NA | neg | NA | 247033 | 231472 | 1.10 | 0.91 | -5.11 | 0.02 |
| 41 | B03 | 5470 | PPEF2 | NM_006239 | 197107 | 218143 | 0.88 | 0.86 | 1.58 | 1.83 |
| 41 | B04 | 5495 | PPM1B | NM_002706 | 211991 | 222509 | 0.95 | 0.88 | 0.73 | 1.97 |
| 41 | B05 | 22843 | PPM1E | NM_014906 | 249975 | 251160 | 1.12 | 0.99 | -2.70 | -1.56 |
| 41 | B06 | 9647 | PPM1F | NM_014634 | 226518 | 222967 | 1.01 | 0.88 | 0.55 | 0.68 |
| 41 | B07 | 5496 | PPM1G | NM_002707 | 239045 | 232814 | 1.07 | 0.92 | -3.44 | -0.09 |
| 41 | B08 | 151742 | PPM1L | NM_139245 | 222537 | 240314 | 1.00 | 0.95 | -0.77 | -0.18 |
| 41 | B09 | 54704 | PPM2C | NM_018444 | 208265 | 228667 | 0.93 | 0.90 | 0.06 | -0.49 |
| 41 | B10 | 5499 | PPP1CA | NM_002708 | 205105 | 210084 | 0.92 | 0.83 | -0.07 | 1.41 |
| 41 | B11 | 5500 | PPP1CB | NM_002709 | 186028 | 219123 | 0.83 | 0.87 | 2.44 | -0.24 |
| 41 | B12 | 84988 | PPP1R16A | NM_032902 | 222817 | 230557 | 1.00 | 0.91 | -1.83 | 0.08 |

| | | | | | | | | | | |
|---|---|---|---|---|---|---|---|---|---|---|
| 41 | C01 | NA | pos | NA | 64709 | 92923 | 0.29 | 0.37 | 23.54 | 12.63 |
| 41 | C02 | NA | NA | NA | 217957 | 229465 | 0.97 | 0.91 | -0.12 | -1.45 |
| 41 | C03 | 79660 | PPP1R3B | NM_024607 | 205286 | 214992 | 0.92 | 0.85 | 0.82 | 0.47 |
| 41 | C04 | 5509 | PPP1R3D | NM_006242 | 218913 | 220235 | 0.98 | 0.87 | 0.17 | 0.52 |
| 41 | C05 | 5518 | PPP2R1A | NM_014225 | 237221 | 191741 | 1.06 | 0.76 | -0.23 | 2.88 |
| 41 | C06 | 5519 | PPP2R1B | NM_002716 | 209835 | 195814 | 0.94 | 0.77 | 3.63 | 1.80 |
| 41 | C07 | 5525 | PPP2R5A | NM_006243 | 236181 | 185208 | 1.06 | 0.73 | -2.49 | 3.14 |
| 41 | C08 | 5526 | PPP2R5B | NM_006244 | 228018 | 226753 | 1.02 | 0.90 | -1.11 | -0.46 |
| 41 | C09 | 5527 | PPP2R5C | NM_002719 | 238865 | 219452 | 1.07 | 0.87 | -4.16 | -1.22 |
| 41 | C10 | 5528 | PPP2R5D | NM_006245 | 203701 | 215438 | 0.91 | 0.85 | 0.66 | -0.83 |
| 41 | C11 | 5529 | PPP2R5E | NM_006246 | 205953 | 212335 | 0.92 | 0.84 | -0.13 | -1.22 |
| 41 | C12 | 5533 | PPP3CC | NM_005605 | 213789 | 232532 | 0.96 | 0.92 | 0.07 | -1.81 |
| 41 | D01 | NA | neg | NA | 227313 | 261178 | 1.02 | 1.03 | -2.93 | -4.82 |
| 41 | D02 | NA | neg | NA | 229698 | 220027 | 1.03 | 0.87 | -3.30 | -0.57 |
| 41 | D03 | 9989 | PPP4R1 | NM_005134 | 210946 | 219183 | 0.94 | 0.87 | -1.42 | -0.05 |
| 41 | D04 | 5536 | PPP5C | NM_006247 | 208953 | 224181 | 0.93 | 0.89 | 0.34 | 0.03 |
| 41 | D05 | 5537 | PPP6C | NM_002721 | 241518 | 210283 | 1.08 | 0.83 | -2.26 | 0.88 |
| 41 | D06 | 7803 | PTP4A1 | NM_003463 | 208833 | 206771 | 0.93 | 0.82 | 2.42 | 0.58 |
| 41 | D07 | 8073 | PTP4A2 | NM_003479 | 210789 | 223144 | 0.94 | 0.88 | 0.06 | -0.87 |
| 41 | D08 | 11156 | PTP4A3 | NM_007079 | 216455 | 219664 | 0.97 | 0.87 | -0.69 | 0.18 |
| 41 | D09 | 138639 | PTPDC1 | NM_152422 | 195547 | 204987 | 0.87 | 0.81 | 1.16 | 0.18 |
| 41 | D10 | 9200 | PTPLA | NM_014241 | 221753 | 215732 | 0.99 | 0.85 | -3.50 | -0.95 |
| 41 | D11 | 201562 | PTPLB | NM_198402 | 186971 | 216712 | 0.84 | 0.86 | 1.43 | -1.77 |
| 41 | D12 | 5770 | PTPN1 | NM_002827 | 205885 | 214268 | 0.92 | 0.85 | -0.07 | -0.01 |
| 41 | E01 | NA | neg | NA | 217379 | 260345 | 0.97 | 1.03 | -1.20 | -5.01 |
| 41 | E02 | NA | neg | NA | 229740 | 225723 | 1.03 | 0.89 | -3.11 | -1.44 |
| 41 | E03 | 5781 | PTPN11 | NM_002834 | 199223 | 215556 | 0.89 | 0.85 | 0.58 | 0.05 |
| 41 | E04 | 5782 | PTPN12 | NM_002835 | 213468 | 222019 | 0.95 | 0.88 | -0.17 | -0.03 |
| 41 | E05 | 5783 | PTPN13 | NM_006264 | 226662 | 206979 | 1.01 | 0.82 | 0.23 | 0.94 |
| 41 | E06 | 11099 | PTPN21 | NM_007039 | 225259 | 225112 | 1.01 | 0.89 | 0.08 | -1.59 |
| 41 | E07 | 26191 | PTPN22 | NM_012411 | 212892 | 215089 | 0.95 | 0.85 | -0.06 | -0.31 |
| 41 | E08 | 25930 | PTPN23 | NM_015466 | 208002 | 199371 | 0.93 | 0.79 | 0.81 | 2.00 |
| 41 | E09 | 5774 | PTPN3 | NM_002829 | 204736 | 202855 | 0.92 | 0.80 | -0.06 | 0.12 |
| 41 | E10 | 5775 | PTPN4 | NM_002830 | 218631 | 205245 | 0.98 | 0.81 | -2.82 | -0.14 |
| 41 | E11 | 5778 | PTPN7 | NM_002832 | 208121 | 196306 | 0.93 | 0.78 | -1.64 | 0.06 |
| 41 | E12 | 5780 | PTPN9 | NM_002833 | 206107 | 234552 | 0.92 | 0.93 | 0.09 | -2.38 |
| 41 | F01 | NA | NA | NA | 207968 | 251560 | 0.93 | 0.99 | 1.22 | -3.32 |
| 41 | F02 | NA | pos | NA | 81988 | 132872 | 0.37 | 0.53 | 20.66 | 8.93 |
| 41 | F03 | 5786 | PTPRA | NM_002836 | 213030 | 228847 | 0.95 | 0.90 | -0.58 | -0.54 |
| 41 | F04 | 5787 | PTPRB | NM_002837 | 238246 | 209175 | 1.07 | 0.83 | -3.03 | 2.08 |
| 41 | F05 | 5788 | PTPRC | NM_002838 | 230260 | 232211 | 1.03 | 0.92 | 0.64 | -0.88 |
| 41 | F06 | 5789 | PTPRD | NM_002839 | 240552 | 222885 | 1.08 | 0.88 | -1.32 | -0.58 |
| 41 | F07 | 5791 | PTPRE | NM_006504 | 205139 | 196463 | 0.92 | 0.78 | 2.10 | 2.39 |
| 41 | F08 | 5792 | PTPRF | NM_002840 | 205573 | 229683 | 0.92 | 0.91 | 2.15 | -0.35 |
| 41 | F09 | 5794 | PTPRH | NM_002842 | 215129 | 212049 | 0.96 | 0.84 | -0.70 | -0.04 |
| 41 | F10 | 5796 | PTPRK | NM_002844 | 201104 | 211262 | 0.90 | 0.83 | 0.85 | 0.02 |
| 41 | F11 | 5797 | PTPRM | NM_002845 | 211607 | 203481 | 0.95 | 0.80 | -1.21 | 0.10 |
| 41 | F12 | 5798 | PTPRN | NM_002846 | 209326 | 206391 | 0.94 | 0.82 | 0.56 | 1.31 |
| 41 | G01 | NA | neg | NA | 215137 | 245732 | 0.96 | 0.97 | -0.09 | -3.38 |
| 41 | G02 | NA | neg | NA | 216548 | 278037 | 0.97 | 1.10 | -0.31 | -6.71 |
| 41 | G03 | 5799 | PTPRN2 | NM_002847 | 224462 | 223423 | 1.00 | 0.88 | -2.55 | -0.64 |
| 41 | G04 | 5800 | PTPRO | NM_002848 | 221684 | 223418 | 0.99 | 0.88 | -0.67 | -0.05 |
| 41 | G05 | 5802 | PTPRS | NM_002850 | 229311 | 204304 | 1.03 | 0.81 | 0.59 | 1.34 |
| 41 | G06 | 5803 | PTPRZ1 | NM_002851 | 232962 | 221141 | 1.04 | 0.87 | -0.35 | -1.06 |
| 41 | G07 | 8732 | RNGTT | NM_003800 | 219343 | 212394 | 0.98 | 0.84 | -0.30 | 0.09 |
| 41 | G08 | 54434 | SSH1 | NM_018984 | 208707 | 206578 | 0.93 | 0.82 | 1.47 | 1.37 |
| 41 | G09 | 85464 | SSH2 | NM_033389 | 210319 | 204784 | 0.94 | 0.81 | -0.16 | 0.04 |
| 41 | G10 | 54961 | SSH3 | NM_017857 | 200887 | 203512 | 0.90 | 0.80 | 0.68 | 0.16 |
| 41 | G11 | 6815 | STYX | NM_145251 | 198913 | 198706 | 0.89 | 0.79 | 0.55 | -0.06 |
| 41 | G12 | 160760 | TA-PP2C | NM_139283 | 210717 | 213592 | 0.94 | 0.84 | 0.14 | -0.10 |
| 41 | H01 | NA | NA | NA | 214716 | 254255 | 0.96 | 1.00 | 1.15 | -0.78 |
| 41 | H02 | NA | pos | NA | 91181 | 113158 | 0.41 | 0.45 | 20.22 | 13.78 |
| 41 | H03 | 23371 | TENC1 | NM_015319 | 197281 | 256924 | 0.88 | 1.02 | 2.83 | -0.62 |
| 41 | H04 | 92609 | TIMM50 | XM_053074 | 260947 | 277240 | 1.17 | 1.10 | -5.55 | -2.12 |
| 41 | H05 | 7179 | TPTE | NM_013315 | 297765 | 269155 | 1.33 | 1.06 | -8.80 | -1.87 |
| 41 | H06 | 8897 | MTMR3 | NM_021090 | 245130 | 280355 | 1.10 | 1.11 | -1.05 | -3.69 |
| 41 | H07 | 5494 | PPM1A | NM_021003 | 213682 | 212986 | 0.96 | 0.84 | 1.76 | 3.51 |
| 41 | H08 | 5532 | PPP3CB | NM_021132 | 226386 | 267364 | 1.01 | 1.06 | -0.08 | -1.42 |
| 41 | H09 | 10454 | MAP3K7IP1 | NM_006116 | 213825 | 226268 | 0.96 | 0.89 | 0.48 | 1.31 |
| 41 | H10 | 5521 | PPP2R2B | NM_004576 | 212517 | 220527 | 0.95 | 0.87 | 0.07 | 1.89 |
| 41 | H11 | 9562 | MINPP1 | NM_004897 | 209277 | 225741 | 0.94 | 0.89 | 0.13 | 0.63 |
| 41 | H12 | 1500 | CTNND1 | NM_001331 | 224722 | 217363 | 1.00 | 0.86 | -0.84 | 2.99 |
| 42 | A01 | NA | pos | NA | 58066 | 67209 | 0.25 | 0.29 | 16.97 | 15.70 |
| 42 | A02 | NA | NA | NA | 230972 | 216034 | 1.00 | 0.92 | -3.74 | -1.78 |
| 42 | A03 | 26469 | PTPN18 | NM_014369 | 215411 | 216689 | 0.93 | 0.92 | -1.01 | -0.92 |
| 42 | A04 | 1365 | CLDN3 | NM_001306 | 198383 | 207609 | 0.85 | 0.88 | 0.77 | 0.03 |
| 42 | A05 | 1364 | CLDN4 | NM_001305 | 215520 | 206117 | 0.93 | 0.88 | -0.39 | -0.12 |
| 42 | A06 | 8218 | CLTCL1 | NM_001835 | 215055 | 202041 | 0.93 | 0.86 | -0.87 | 0.38 |
| 42 | A07 | 10871 | CMRF35 | NM_006678 | 200684 | 203068 | 0.86 | 0.86 | -0.42 | -0.27 |
| 42 | A08 | 1271 | CNTFR | NM_001842 | 198189 | 197254 | 0.85 | 0.84 | 0.42 | 0.43 |
| 42 | A09 | 1398 | CRK | NM_005206 | 188528 | 199858 | 0.81 | 0.85 | 0.86 | -0.01 |
| 42 | A10 | 64109 | CRLF2 | NM_022148 | 188024 | 189600 | 0.81 | 0.81 | 0.92 | 0.71 |
| 42 | A11 | 1438 | CSF2RA | NM_006140 | 192257 | 192967 | 0.83 | 0.82 | 0.37 | 0.11 |
| 42 | A12 | 1525 | CXADR | NM_001338 | 214450 | 212665 | 0.92 | 0.90 | -2.04 | -1.90 |
| 42 | B01 | NA | neg | NA | 251381 | 226674 | 1.08 | 0.96 | -4.26 | -1.18 |
| 42 | B02 | NA | neg | NA | 213255 | 213571 | 0.92 | 0.91 | 0.31 | 0.36 |
| 42 | B03 | 1630 | DCC | NM_005215 | 221380 | 203012 | 0.95 | 0.86 | 0.20 | 2.54 |
| 42 | B04 | 10682 | EBP | NM_006579 | 216786 | 237032 | 0.93 | 1.01 | 0.49 | -1.58 |

| | | | | | | | | | | |
|---|---|---|---|---|---|---|---|---|---|---|
| 42 | B05 | 10913 | EDAR | NM_022336 | 225021 | 215371 | 0.97 | 0.92 | 0.39 | 0.64 |
| 42 | B06 | 2152 | F3 | NM_001993 | 221544 | 220998 | 0.95 | 0.94 | 0.28 | 0.00 |
| 42 | B07 | 115350 | FCRH1 | NM_052938 | 212855 | 212742 | 0.92 | 0.90 | 0.04 | 0.44 |
| 42 | B08 | 115352 | FCRH3 | NM_052939 | 223392 | 227036 | 0.96 | 0.97 | -0.67 | -1.22 |
| 42 | B09 | 28982 | FLVCR | NM_014053 | 222600 | 228871 | 0.96 | 0.97 | -1.30 | -1.57 |
| 42 | B10 | 27315 | FRAG1 | NM_014489 | 213179 | 211402 | 0.92 | 0.90 | -0.16 | 0.00 |
| 42 | B11 | 84824 | FREB | NM_032738 | 215058 | 210646 | 0.93 | 0.90 | -0.43 | -0.11 |
| 42 | B12 | 10468 | FST | NM_013409 | 213872 | 212064 | 0.92 | 0.90 | -0.04 | 0.02 |
| 42 | C01 | NA | pos | NA | 75123 | 126507 | 0.32 | 0.54 | 15.87 | 9.09 |
| 42 | C02 | NA | NA | NA | 206269 | 212278 | 0.89 | 0.90 | 0.17 | -0.98 |
| 42 | C03 | 2674 | GFRA1 | NM_005264 | 216639 | 190383 | 0.93 | 0.81 | -0.20 | 2.53 |
| 42 | C04 | 64096 | GFRA4 | NM_022139 | 184840 | 201737 | 0.80 | 0.86 | 3.34 | 1.08 |
| 42 | C05 | 2690 | GHR | NM_000163 | 188580 | 207068 | 0.81 | 0.88 | 3.78 | 0.12 |
| 42 | C06 | 2697 | GJA1 | NM_000165 | 185301 | 209360 | 0.80 | 0.89 | 3.64 | -0.12 |
| 42 | C07 | 9527 | GOSR1 | NM_004871 | 203290 | 201741 | 0.88 | 0.86 | 0.21 | 0.24 |
| 42 | C08 | 9570 | GOSR2 | NM_004287 | 207964 | 201263 | 0.90 | 0.86 | 0.20 | 0.31 |
| 42 | C09 | 64582 | GPR135 | NM_022571 | 214295 | 214642 | 0.92 | 0.91 | -1.28 | -1.39 |
| 42 | C10 | 350383 | GPR142 | NM_181790 | 209625 | 214939 | 0.90 | 0.91 | -0.71 | -1.91 |
| 42 | C11 | 10750 | GRAP | NM_006613 | 216281 | 218349 | 0.93 | 0.93 | -1.56 | -2.51 |
| 42 | C12 | 9402 | GRAP2 | NM_004810 | 230896 | 230288 | 1.00 | 0.98 | -3.06 | -3.61 |
| 42 | D01 | NA | neg | NA | 242947 | 254633 | 1.05 | 1.08 | -3.39 | -4.67 |
| 42 | D02 | NA | neg | NA | 223348 | 249538 | 0.96 | 1.06 | -1.04 | -4.07 |
| 42 | D03 | 2887 | GRB10 | NM_001001549 | 206006 | 222793 | 0.89 | 0.95 | 1.91 | 0.01 |
| 42 | D04 | 2888 | GRB14 | NM_004490 | 218648 | 202050 | 0.94 | 0.86 | 0.13 | 2.33 |
| 42 | D05 | 2886 | GRB7 | NM_005310 | 220943 | 191994 | 0.95 | 0.82 | 0.74 | 3.18 |
| 42 | D06 | 2890 | GRIA1 | NM_000827 | 224993 | 207797 | 0.97 | 0.88 | -0.28 | 1.35 |
| 42 | D07 | 2891 | GRIA2 | NM_000826 | 213239 | 214545 | 0.92 | 0.91 | -0.14 | 0.02 |
| 42 | D08 | 2892 | GRIA3 | NM_000828 | 209290 | 217081 | 0.90 | 0.92 | 0.88 | -0.26 |
| 42 | D09 | 2893 | GRIA4 | NM_000829 | 224182 | 215574 | 0.97 | 0.92 | -1.63 | -0.21 |
| 42 | D10 | 2895 | GRID2 | NM_001510 | 219257 | 213570 | 0.94 | 0.91 | -1.03 | -0.46 |
| 42 | D11 | 2898 | GRIK2 | NM_021956 | 215658 | 213316 | 0.93 | 0.91 | -0.64 | -0.63 |
| 42 | D12 | 2899 | GRIK3 | NM_000831 | 208705 | 210642 | 0.90 | 0.90 | 0.44 | -0.02 |
| 42 | E01 | NA | neg | NA | 246864 | 217289 | 1.06 | 0.92 | -5.12 | -1.66 |
| 42 | E02 | NA | neg | NA | 207282 | 209256 | 0.89 | 0.89 | -0.38 | -0.72 |
| 42 | E03 | 2900 | GRIK4 | NM_014619 | 198792 | 211245 | 0.86 | 0.90 | 1.51 | -0.01 |
| 42 | E04 | 2901 | GRIK5 | NM_002088 | 223303 | 200530 | 0.96 | 0.85 | -1.69 | 1.13 |
| 42 | E05 | 116443 | GRIN3A | NM_133445 | 223635 | 224479 | 0.96 | 0.95 | -0.84 | -2.01 |
| 42 | E06 | 81488 | GRINL1A | NM_015532 | 193001 | 190422 | 0.83 | 0.81 | 2.29 | 2.02 |
| 42 | E07 | 84868 | HAVCR2 | NM_032782 | 198844 | 212911 | 0.86 | 0.90 | 0.32 | -1.16 |
| 42 | E08 | 3077 | HFE | NM_000410 | 202696 | 200987 | 0.87 | 0.85 | 0.41 | 0.26 |
| 42 | E09 | 3105 | HLA-A | NM_002116 | 189583 | 190917 | 0.82 | 0.81 | 1.25 | 1.31 |
| 42 | E10 | 3106 | HLA-B | NM_005514 | 210993 | 197906 | 0.91 | 0.84 | -1.30 | 0.00 |
| 42 | E11 | 3107 | HLA-C | NM_002117 | 202544 | 203740 | 0.87 | 0.87 | -0.33 | -0.88 |
| 42 | E12 | 3109 | HLA-DMB | NM_002118 | 207974 | 207298 | 0.90 | 0.88 | -0.74 | -1.00 |
| 42 | F01 | NA | NA | NA | 251513 | 241284 | 1.08 | 1.03 | -5.94 | -4.11 |
| 42 | F02 | NA | pos | NA | 89035 | 135049 | 0.38 | 0.57 | 13.52 | 8.37 |
| 42 | F03 | 3111 | HLA-DOA | NM_002119 | 219675 | 214189 | 0.95 | 0.91 | -1.26 | 0.01 |
| 42 | F04 | 3112 | HLA-DOB | NM_002120 | 208093 | 216778 | 0.90 | 0.92 | -0.13 | -0.41 |
| 42 | F05 | 3115 | HLA-DPB1 | NM_002121 | 203017 | 209424 | 0.87 | 0.89 | 1.36 | 0.13 |
| 42 | F06 | 3117 | HLA-DQA1 | NM_002122 | 204193 | 226678 | 0.88 | 0.96 | 0.69 | -1.87 |
| 42 | F07 | 3119 | HLA-DQB1 | NM_002123 | 199664 | 206377 | 0.86 | 0.88 | -0.04 | -0.02 |
| 42 | F08 | 3122 | HLA-DRA | NM_019111 | 210090 | 209645 | 0.91 | 0.89 | -0.74 | -0.39 |
| 42 | F09 | 3125 | HLA-DRB3 | NM_022555 | 201803 | 194738 | 0.87 | 0.83 | -0.47 | 1.23 |
| 42 | F10 | 3133 | HLA-E | NM_005516 | 196542 | 202217 | 0.85 | 0.86 | 0.16 | -0.13 |
| 42 | F11 | 3135 | HLA-G | NM_002127 | 194754 | 198383 | 0.84 | 0.84 | 0.33 | 0.12 |
| 42 | F12 | 353164 | HT2R55 | NM_181429 | 199260 | 201473 | 0.86 | 0.86 | 0.04 | 0.05 |
| 42 | G01 | NA | neg | NA | 240756 | 259193 | 1.04 | 1.10 | -4.02 | -5.70 |
| 42 | G02 | NA | neg | NA | 213394 | 243863 | 0.92 | 1.04 | -0.74 | -3.89 |
| 42 | G03 | 3590 | IL11RA | NM_004512 | 209470 | 220151 | 0.90 | 0.94 | 0.60 | -0.17 |
| 42 | G04 | 3597 | IL13RA1 | NM_001560 | 227194 | 217935 | 0.98 | 0.93 | -1.79 | -0.03 |
| 42 | G05 | 8807 | IL18RAP | NM_003853 | 237444 | 232604 | 1.02 | 0.99 | -2.13 | -2.08 |
| 42 | G06 | 11141 | IL1RAPL1 | NM_014271 | 220188 | 215169 | 0.95 | 0.91 | -0.60 | 0.00 |
| 42 | G07 | 53832 | IL20RA | NM_014432 | 215258 | 218208 | 0.93 | 0.93 | -1.28 | -0.90 |
| 42 | G08 | 116379 | IL22RA2 | NM_052962 | 217421 | 204513 | 0.94 | 0.87 | -0.99 | 0.73 |
| 42 | G09 | 149233 | IL23R | NM_144701 | 185704 | 209473 | 0.80 | 0.89 | 2.08 | 0.01 |
| 42 | G10 | 163702 | IL28RA | NM_170743 | 196734 | 192581 | 0.85 | 0.82 | 0.77 | 1.52 |
| 42 | G11 | 23547 | ILT7 | NM_012276 | 189778 | 200286 | 0.82 | 0.85 | 1.56 | 0.41 |
| 42 | G12 | 342510 | IREM2 | NM_181449 | 185491 | 194074 | 0.80 | 0.82 | 2.32 | 1.44 |
| 42 | H01 | NA | NA | NA | 241540 | 247544 | 1.04 | 1.05 | -1.34 | -2.90 |
| 42 | H02 | NA | pos | NA | 85258 | 86481 | 0.37 | 0.37 | 17.37 | 16.02 |
| 42 | H03 | 3708 | ITPR1 | NM_002222 | 258651 | 248176 | 1.11 | 1.05 | -2.52 | -2.03 |
| 42 | H04 | 3710 | ITPR3 | NM_002224 | 279340 | 272082 | 1.20 | 1.16 | -5.26 | -4.96 |
| 42 | H05 | 3757 | KCNH2 | NM_000238 | 270809 | 230950 | 1.17 | 0.98 | -3.35 | -0.45 |
| 42 | H06 | 23416 | KCNH3 | NM_012284 | 267061 | 241541 | 1.15 | 1.03 | -3.44 | -1.67 |
| 42 | H07 | 23415 | KCNH4 | NM_012285 | 226345 | 216310 | 0.98 | 0.92 | 0.16 | 0.76 |
| 42 | H08 | 90134 | KCNH7 | NM_033272 | 233973 | 280703 | 1.01 | 1.19 | -0.20 | -6.79 |
| 42 | H09 | 131096 | KCNH8 | NM_144633 | 222303 | 207602 | 0.96 | 0.88 | 0.47 | 1.67 |
| 42 | H10 | 11015 | KDELR3 | NM_006855 | 200450 | 203209 | 0.86 | 0.86 | 3.10 | 1.70 |
| 42 | H11 | 57292 | KIR2DL5 | NM_020535 | 209396 | 200674 | 0.90 | 0.85 | 1.98 | 1.80 |
| 42 | H12 | 3123 | HLA-DRB1 | NM_002124 | 211892 | 214672 | 0.91 | 0.91 | 1.93 | 0.45 |
| 43 | A01 | NA | pos | NA | 70312 | 75078 | 0.32 | 0.33 | 15.73 | 13.66 |
| 43 | A02 | NA | NA | NA | 192720 | 222730 | 0.87 | 0.97 | 0.26 | -1.71 |
| 43 | A03 | 3921 | LAMR1 | NM_002295 | 127598 | 129985 | 0.58 | 0.57 | 9.09 | 8.87 |
| 43 | A04 | 27040 | LAT | NM_014387 | 200335 | 219277 | 0.91 | 0.96 | 0.08 | -0.32 |
| 43 | A05 | 3953 | LEPR | NM_002303 | 199017 | 210305 | 0.90 | 0.92 | -0.85 | 0.03 |
| 43 | A06 | 3976 | LIF | NM_002309 | 184851 | 206904 | 0.84 | 0.90 | 1.16 | 0.20 |
| 43 | A07 | 10859 | LILRB1 | NM_006669 | 206501 | 207226 | 0.94 | 0.91 | -0.84 | -0.40 |
| 43 | A08 | 10288 | LILRB2 | NM_005874 | 201431 | 202566 | 0.91 | 0.89 | -0.76 | 0.08 |

| | | | | | | | | | | |
|---|---|---|---|---|---|---|---|---|---|---|
| 43 | A09 | 11006 | LILRB4 | NM_006847 | 188738 | 202352 | 0.86 | 0.88 | 0.10 | -0.10 |
| 43 | A10 | 10990 | LILRB5 | NM_006840 | 184782 | 197246 | 0.84 | 0.86 | 0.00 | -0.02 |
| 43 | A11 | 55716 | LIMR | NM_018113 | 196115 | 202340 | 0.89 | 0.88 | -1.70 | -0.11 |
| 43 | A12 | 4037 | LRP3 | NM_002333 | 198220 | 205034 | 0.90 | 0.90 | 0.00 | 1.37 |
| 43 | B01 | NA | neg | NA | 235424 | 231191 | 1.07 | 1.01 | -3.10 | -1.42 |
| 43 | B02 | NA | neg | NA | 219160 | 222787 | 0.99 | 0.97 | -1.04 | -0.54 |
| 43 | B03 | 4041 | LRP5 | NM_002335 | 208662 | 212971 | 0.95 | 0.93 | 0.88 | 1.40 |
| 43 | B04 | 4059 | LU | NM_005581 | 213218 | 221591 | 0.97 | 0.97 | 0.49 | 0.62 |
| 43 | B05 | 283316 | M160 | NM_174941 | 225103 | 222116 | 1.02 | 0.97 | -2.11 | -0.03 |
| 43 | B06 | 4074 | M6PR | NM_002355 | 225004 | 222016 | 1.02 | 0.97 | -1.88 | -0.20 |
| 43 | B07 | 84441 | MAML2 | NM_032427 | 226974 | 219988 | 1.03 | 0.96 | -1.39 | -0.56 |
| 43 | B08 | 8685 | MARCO | NM_006770 | 213883 | 225483 | 0.97 | 0.99 | -0.29 | -1.13 |
| 43 | B09 | 4163 | MCC | NM_002387 | 207234 | 211763 | 0.94 | 0.93 | -0.20 | 0.10 |
| 43 | B10 | 4179 | MCP | NM_002389 | 199374 | 207982 | 0.90 | 0.91 | 0.20 | 0.04 |
| 43 | B11 | 4352 | MPL | NM_005373 | 196437 | 208017 | 0.89 | 0.91 | 0.30 | 0.47 |
| 43 | B12 | 164091 | MPRA | NM_178422 | 209906 | 233900 | 0.95 | 1.02 | 0.56 | -0.46 |
| 43 | C01 | NA | pos | NA | 72022 | 89006 | 0.33 | 0.39 | 17.47 | 13.61 |
| 43 | C02 | NA | NA | NA | 225340 | 226759 | 1.02 | 0.99 | -1.91 | -0.73 |
| 43 | C03 | 931 | MS4A1 | NM_021950 | 194264 | 211749 | 0.88 | 0.93 | 2.62 | 1.76 |
| 43 | C04 | 64231 | MS4A6A | NM_022349 | 190082 | 210655 | 0.86 | 0.92 | 3.33 | 1.98 |
| 43 | C05 | 58475 | MS4A7 | NM_021201 | 182019 | 214352 | 0.83 | 0.94 | 3.26 | 1.01 |
| 43 | C06 | 83661 | MS4A8B | NM_031457 | 214702 | 184803 | 0.97 | 0.81 | -0.67 | 3.90 |
| 43 | C07 | 4481 | MSR1 | NM_002445 | 211112 | 218603 | 0.96 | 0.96 | 0.53 | -0.18 |
| 43 | C08 | 23625 | MTVR1 | NM_152832 | 229584 | 217592 | 1.04 | 0.95 | -2.36 | -0.08 |
| 43 | C09 | 4653 | MYOC | NM_000261 | 204942 | 228769 | 0.93 | 1.00 | 0.00 | -1.44 |
| 43 | C10 | 9437 | NCR1 | NM_004829 | 200304 | 217259 | 0.91 | 0.95 | 0.00 | -0.70 |
| 43 | C11 | 259197 | NCR3 | NM_147130 | 202784 | 220104 | 0.92 | 0.96 | -0.59 | -0.55 |
| 43 | C12 | 4804 | NGFR | NM_002507 | 220880 | 231254 | 1.00 | 1.01 | -0.91 | 0.04 |
| 43 | D01 | NA | neg | NA | 256350 | 254046 | 1.16 | 1.11 | -6.16 | -5.06 |
| 43 | D02 | NA | neg | NA | 215350 | 222547 | 0.98 | 0.97 | -0.97 | -1.78 |
| 43 | D03 | 349667 | RTN4RL2 | NM_178570 | 213566 | 216973 | 0.97 | 0.95 | -0.15 | -0.27 |
| 43 | D04 | 146760 | RTN4RL1 | NM_178568 | 220385 | 225565 | 1.00 | 0.99 | -0.83 | -1.05 |
| 43 | D05 | 146722 | NKIR | NM_139018 | 198507 | 219291 | 0.90 | 0.96 | 0.85 | -0.99 |
| 43 | D06 | 4864 | NPC1 | NM_000271 | 211099 | 195527 | 0.96 | 0.85 | -0.53 | 1.30 |
| 43 | D07 | 23467 | NPTXR | NM_014293 | 215284 | 200828 | 0.98 | 0.88 | -0.33 | 0.18 |
| 43 | D08 | 8828 | NRP2 | NM_003872 | 207015 | 193882 | 0.94 | 0.85 | 0.17 | 0.90 |
| 43 | D09 | 8439 | NSMAF | NM_003580 | 201342 | 188169 | 0.91 | 0.82 | 0.13 | 1.30 |
| 43 | D10 | 266743 | NXF | NM_178864 | 165086 | 192056 | 0.75 | 0.84 | 4.12 | 0.44 |
| 43 | D11 | 126014 | OSCAR | NM_130771 | 180576 | 203730 | 0.82 | 0.89 | 1.89 | -0.34 |
| 43 | D12 | 9180 | OSMR | NM_003999 | 216501 | 222005 | 0.98 | 0.97 | -0.69 | -0.48 |
| 43 | E01 | NA | neg | NA | 219109 | 240574 | 0.99 | 1.05 | -1.87 | -5.20 |
| 43 | E02 | NA | neg | NA | 221603 | 219682 | 1.01 | 0.96 | -2.18 | -3.02 |
| 43 | E03 | 5133 | PDCD1 | NM_005018 | 228942 | 210180 | 1.04 | 0.92 | -2.51 | -1.11 |
| 43 | E04 | 5187 | PER1 | NM_002616 | 211150 | 227078 | 0.96 | 0.99 | -0.08 | -2.76 |
| 43 | E05 | 8864 | PER2 | NM_003894 | 191905 | 186005 | 0.87 | 0.81 | 1.26 | 0.93 |
| 43 | E06 | 5314 | PKHD1 | NM_138694 | 202833 | 231640 | 0.92 | 1.01 | 0.09 | -4.00 |
| 43 | E07 | 5329 | PLAUR | NM_002659 | 206820 | 182031 | 0.94 | 0.80 | 0.33 | 0.59 |
| 43 | E08 | 55558 | PLXNA3 | NM_017514 | 177701 | 170723 | 0.81 | 0.75 | 3.45 | 1.77 |
| 43 | E09 | 5365 | PLXNB3 | NM_005393 | 199118 | 179465 | 0.90 | 0.78 | 0.00 | 0.66 |
| 43 | E10 | 55629 | PNRC2 | NM_017761 | 207283 | 181203 | 0.94 | 0.79 | -1.63 | 0.02 |
| 43 | E11 | 114908 | PORIMIN | NM_052932 | 192888 | 203939 | 0.88 | 0.89 | -0.08 | -1.90 |
| 43 | E12 | 5544 | PRB3 | NM_006249 | 207737 | 202990 | 0.94 | 0.89 | 0.00 | -0.04 |
| 43 | F01 | NA | NA | NA | 213694 | 229575 | 0.97 | 1.00 | -0.20 | -1.41 |
| 43 | F02 | NA | pos | NA | 71139 | 133424 | 0.32 | 0.58 | 17.82 | 8.60 |
| 43 | F03 | 10544 | PROCR | NM_006404 | 215694 | 222314 | 0.98 | 0.97 | 0.15 | 0.27 |
| 43 | F04 | 5662 | PSD | NM_002779 | 226576 | 230220 | 1.03 | 1.01 | -1.05 | -0.44 |
| 43 | F05 | 26470 | PSK-1 | NM_012410 | 228600 | 232735 | 1.04 | 1.02 | -2.40 | -1.29 |
| 43 | F06 | 5727 | PTCH | NM_000264 | 199180 | 222023 | 0.90 | 0.97 | 1.53 | -0.36 |
| 43 | F07 | 8643 | PTCH2 | NM_003738 | 189609 | 189720 | 0.86 | 0.83 | 3.48 | 2.44 |
| 43 | F08 | 5817 | PVR | NM_006505 | 214067 | 216287 | 0.97 | 0.95 | -0.17 | -0.33 |
| 43 | F09 | 5819 | PVRL2 | NM_002856 | 193204 | 207732 | 0.88 | 0.91 | 1.72 | 0.36 |
| 43 | F10 | 10266 | RAMP2 | NM_005854 | 208037 | 216201 | 0.94 | 0.94 | -0.75 | -0.98 |
| 43 | F11 | 10268 | RAMP3 | NM_005856 | 204823 | 204997 | 0.93 | 0.90 | -0.61 | 0.63 |
| 43 | F12 | 51720 | RAP80 | NM_016290 | 209039 | 206680 | 0.95 | 0.90 | 0.82 | 2.22 |
| 43 | G01 | NA | neg | NA | 234633 | 237609 | 1.06 | 1.04 | -2.83 | -2.52 |
| 43 | G02 | NA | neg | NA | 217078 | 226448 | 0.99 | 0.99 | -0.61 | -1.35 |
| 43 | G03 | 26575 | RGS17 | NM_012419 | 228449 | 231474 | 1.04 | 1.01 | -1.45 | -0.95 |
| 43 | G04 | 6091 | ROBO1 | NM_002941 | 214777 | 218183 | 0.97 | 0.95 | 0.46 | 0.54 |
| 43 | G05 | 65078 | RTN4R | NM_023004 | 193245 | 218653 | 0.88 | 0.96 | 2.09 | -0.10 |
| 43 | G06 | 8935 | SCAP2 | NM_003930 | 212174 | 210011 | 0.96 | 0.92 | -0.09 | 0.62 |
| 43 | G07 | 949 | SCARB1 | NM_005505 | 206621 | 223361 | 0.94 | 0.98 | 1.35 | -1.34 |
| 43 | G08 | 29106 | SCG3 | NM_013243 | 199995 | 189393 | 0.91 | 0.83 | 1.63 | 2.20 |
| 43 | G09 | 27020 | SDFR1 | NM_012428 | 211881 | 215986 | 0.96 | 0.94 | -0.62 | -0.77 |
| 43 | G10 | 10371 | SEMA3A | NM_006080 | 210978 | 211876 | 0.96 | 0.93 | -1.10 | -0.80 |
| 43 | G11 | 6405 | SEMA3F | NM_004186 | 199461 | 207397 | 0.91 | 0.91 | 0.08 | 0.11 |
| 43 | G12 | 10507 | SEMA4D | NM_006378 | 218269 | 211777 | 0.99 | 0.93 | -0.33 | 1.41 |
| 43 | H01 | NA | NA | NA | 237061 | 241288 | 1.08 | 1.05 | -1.13 | -1.06 |
| 43 | H02 | NA | pos | NA | 78260 | 97156 | 0.36 | 0.42 | 18.95 | 13.94 |
| 43 | H03 | 10501 | SEMA6B | NM_020241 | 268481 | 262726 | 1.22 | 1.15 | -4.50 | -2.37 |
| 43 | H04 | 6422 | SFRP1 | NM_003012 | 237715 | 237990 | 1.08 | 1.04 | -0.43 | 0.32 |
| 43 | H05 | 6423 | SFRP2 | XM_050625 | 238199 | 227449 | 1.08 | 0.99 | -1.58 | 0.82 |
| 43 | H06 | 6424 | SFRP4 | NM_003014 | 225984 | 273581 | 1.03 | 1.20 | 0.17 | -4.16 |
| 43 | H07 | 6425 | SFRP5 | NM_003015 | 241610 | 219504 | 1.10 | 0.96 | -1.07 | 0.90 |
| 43 | H08 | 51463 | SH120 | NM_016334 | 220459 | 238222 | 1.00 | 1.04 | 1.05 | -1.05 |
| 43 | H09 | 4068 | SH2D1A | NM_002351 | 224269 | 229034 | 1.02 | 1.00 | -0.18 | -0.29 |
| 43 | H10 | 6450 | SH3BGR | NM_007341 | 205189 | 203863 | 0.93 | 0.89 | 1.64 | 1.87 |
| 43 | H11 | 9467 | SH3BP5 | NM_004844 | 205452 | 216741 | 0.93 | 0.95 | 1.33 | 0.97 |
| 43 | H12 | 124583 | ENTPD8 | NM_138793 | 222937 | 248363 | 1.01 | 1.09 | 1.09 | -0.56 |

| | | | | | | | | | | |
|---|---|---|---|---|---|---|---|---|---|---|
| 44 | A01 | NA | pos | NA | 56156 | 65716 | 0.24 | 0.26 | 14.64 | 17.79 |
| 44 | A02 | NA | NA | NA | 195703 | 225491 | 0.83 | 0.91 | -0.22 | -2.38 |
| 44 | A03 | 6461 | SHB | NM_003028 | 195905 | 233352 | 0.83 | 0.94 | 0.95 | -0.96 |
| 44 | A04 | 53358 | SHC3 | NM_016848 | 186747 | 206786 | 0.79 | 0.83 | 1.41 | 1.41 |
| 44 | A05 | 10572 | SIVA | NM_006427 | 193987 | 218084 | 0.82 | 0.88 | 0.14 | -0.30 |
| 44 | A06 | 6503 | SLA | NM_006748 | 201687 | 206733 | 0.86 | 0.83 | -0.05 | 0.03 |
| 44 | A07 | 114836 | SLAMF6 | NM_052931 | 212847 | 204259 | 0.90 | 0.82 | -2.97 | -0.21 |
| 44 | A08 | 51429 | SNX9 | NM_016224 | 198700 | 194687 | 0.84 | 0.78 | -0.16 | 1.47 |
| 44 | A09 | 6653 | SORL1 | NM_003105 | 189278 | 200789 | 0.80 | 0.81 | 0.07 | -0.07 |
| 44 | A10 | 79368 | SPAP1 | NM_030764 | 185064 | 200446 | 0.79 | 0.81 | 0.26 | -0.24 |
| 44 | A11 | 8027 | STAM | NM_003473 | 195288 | 201068 | 0.83 | 0.81 | -0.31 | 0.21 |
| 44 | A12 | 3925 | STMN1 | NM_005563 | 202232 | 204559 | 0.86 | 0.82 | -1.04 | 0.98 |
| 44 | B01 | NA | neg | NA | 260243 | 267271 | 1.11 | 1.07 | -5.34 | -6.58 |
| 44 | B02 | NA | neg | NA | 219373 | 227784 | 0.93 | 0.92 | -0.99 | -1.59 |
| 44 | B03 | 4070 | TACSTD2 | NM_002353 | 220113 | 237207 | 0.94 | 0.95 | 0.12 | -0.37 |
| 44 | B04 | 5726 | TAS2R38 | NM_176817 | 214942 | 227235 | 0.91 | 0.91 | 0.15 | -0.10 |
| 44 | B05 | 10342 | TFG | NM_006070 | 199457 | 221761 | 0.85 | 0.89 | 1.31 | 0.30 |
| 44 | B06 | 9392 | TGFBRAP1 | NM_004257 | 225088 | 215742 | 0.96 | 0.87 | -0.80 | -0.03 |
| 44 | B07 | 10226 | M6PRBP1 | NM_005817 | 222735 | 195968 | 0.95 | 0.79 | -2.27 | 1.91 |
| 44 | B08 | 114609 | TIRAP | NM_052887 | 220903 | 225008 | 0.94 | 0.90 | -0.78 | -1.29 |
| 44 | B09 | 81793 | TLR10 | NM_030956 | 207025 | 224796 | 0.88 | 0.90 | -0.07 | -2.02 |
| 44 | B10 | 7100 | TLR5 | NM_003268 | 191795 | 201338 | 0.82 | 0.81 | 1.29 | 0.72 |
| 44 | B11 | 51284 | TLR7 | NM_016562 | 203160 | 210968 | 0.86 | 0.85 | 0.60 | 0.04 |
| 44 | B12 | 54106 | TLR9 | NM_017442 | 210781 | 219270 | 0.90 | 0.88 | -0.20 | 0.20 |
| 44 | C01 | NA | pos | NA | 77119 | 80276 | 0.33 | 0.32 | 15.11 | 17.79 |
| 44 | C02 | NA | NA | NA | 231799 | 233322 | 0.99 | 0.94 | -1.36 | -1.53 |
| 44 | C03 | 8794 | TNFRSF10C | NM_003841 | 215953 | 244814 | 0.92 | 0.98 | 1.52 | -0.57 |
| 44 | C04 | 8793 | TNFRSF10D | NM_003840 | 182054 | 231471 | 0.77 | 0.93 | 4.61 | 0.13 |
| 44 | C05 | 8792 | TNFRSF11A | NM_003839 | 224956 | 183926 | 0.96 | 0.74 | -0.45 | 5.85 |
| 44 | C06 | 23495 | TNFRSF13B | NM_012452 | 213885 | 179589 | 0.91 | 0.72 | 1.35 | 5.30 |
| 44 | C07 | 608 | TNFRSF17 | NM_001192 | 192554 | 218433 | 0.82 | 0.88 | 1.89 | -0.17 |
| 44 | C08 | 84957 | TNFRSF19L | NM_032871 | 220536 | 219755 | 0.94 | 0.88 | 0.22 | 0.14 |
| 44 | C09 | 27242 | TNFRSF21 | NM_014452 | 227315 | 213541 | 0.97 | 0.86 | -1.28 | 0.16 |
| 44 | C10 | 958 | TNFRSF5 | NM_001250 | 214321 | 214050 | 0.91 | 0.86 | -0.15 | -0.12 |
| 44 | C11 | 355 | TNFRSF6 | NM_000043 | 229285 | 231819 | 0.97 | 0.93 | -1.23 | -1.83 |
| 44 | C12 | 943 | TNFRSF8 | NM_001243 | 227195 | 239146 | 0.97 | 0.96 | -0.99 | -1.55 |
| 44 | D01 | NA | neg | NA | 260225 | 254428 | 1.11 | 1.02 | -5.68 | -5.99 |
| 44 | D02 | NA | neg | NA | 223499 | 212990 | 0.95 | 0.86 | -1.77 | -0.76 |
| 44 | D03 | 3604 | TNFRSF9 | NM_001561 | 223518 | 218702 | 0.95 | 0.88 | -0.58 | 0.93 |
| 44 | D04 | 7159 | TP53BP2 | NM_005426 | 214604 | 217431 | 0.91 | 0.87 | -0.15 | 0.10 |
| 44 | D05 | 8717 | TRADD | NM_003789 | 195875 | 201352 | 0.83 | 0.81 | 1.35 | 1.85 |
| 44 | D06 | 54210 | TREM1 | NM_018643 | 213893 | 193429 | 0.91 | 0.78 | 0.05 | 1.75 |
| 44 | D07 | 54209 | TREM2 | NM_018965 | 199264 | 203419 | 0.85 | 0.82 | -0.12 | -0.07 |
| 44 | D08 | 124599 | TREM5 | NM_174892 | 215845 | 210178 | 0.92 | 0.84 | -0.58 | -0.45 |
| 44 | D09 | 7223 | TRPC4 | NM_016179 | 202033 | 225610 | 0.86 | 0.91 | 0.12 | -3.16 |
| 44 | D10 | 57113 | TRPC7 | NM_020389 | 202114 | 210894 | 0.86 | 0.85 | -0.15 | -1.52 |
| 44 | D11 | 54795 | TRPM4 | NM_017636 | 202599 | 195598 | 0.86 | 0.79 | 0.31 | 0.94 |
| 44 | D12 | 51393 | TRPV2 | NM_016113 | 193899 | 214254 | 0.82 | 0.86 | 1.26 | -0.20 |
| 44 | E01 | NA | neg | NA | 260668 | 259039 | 1.11 | 1.04 | -7.33 | -7.59 |
| 44 | E02 | NA | neg | NA | 227857 | 212959 | 0.97 | 0.86 | -3.84 | -1.77 |
| 44 | E03 | 162514 | TRPV3 | NM_145068 | 204103 | 215094 | 0.87 | 0.86 | -0.12 | 0.37 |
| 44 | E04 | 7305 | TYROBP | NM_003332 | 214257 | 226841 | 0.91 | 0.91 | -1.72 | -2.10 |
| 44 | E05 | 219699 | UNC5B | NM_170744 | 187222 | 215373 | 0.80 | 0.87 | 0.66 | -0.94 |
| 44 | E06 | 8633 | UNC5C | NM_003728 | 187044 | 182322 | 0.79 | 0.73 | 1.30 | 2.14 |
| 44 | E07 | 10163 | WASF2 | NM_006990 | 177302 | 178932 | 0.75 | 0.72 | 0.61 | 2.01 |
| 44 | E08 | 7471 | WNT1 | NM_005430 | 208137 | 205765 | 0.88 | 0.83 | -1.37 | -0.91 |
| 44 | E09 | 80326 | WNT10A | NM_025216 | 177593 | 190791 | 0.75 | 0.77 | 1.11 | 0.22 |
| 44 | E10 | 7480 | WNT10B | NM_003394 | 183920 | 185398 | 0.78 | 0.75 | 0.18 | 0.68 |
| 44 | E11 | 7481 | WNT11 | NM_004626 | 196664 | 202241 | 0.84 | 0.81 | -0.66 | -0.91 |
| 44 | E12 | 51384 | WNT16 | NM_016087 | 214650 | 206425 | 0.91 | 0.83 | -2.56 | -0.23 |
| 44 | F01 | NA | NA | NA | 256995 | 222331 | 1.09 | 0.89 | -6.66 | -2.27 |
| 44 | F02 | NA | pos | NA | 77584 | 115959 | 0.33 | 0.47 | 12.44 | 11.16 |
| 44 | F03 | 7472 | WNT2 | NM_003391 | 224026 | 213429 | 0.95 | 0.86 | -1.96 | 1.27 |
| 44 | F04 | 7482 | WNT2B | NM_004185 | 197611 | 225602 | 0.84 | 0.91 | 0.33 | -1.26 |
| 44 | F05 | 7473 | WNT3 | NM_030753 | 224076 | 233646 | 0.95 | 0.94 | -2.98 | -2.56 |
| 44 | F06 | 89780 | WNT3A | NM_033131 | 215249 | 227938 | 0.91 | 0.92 | -1.41 | -2.93 |
| 44 | F07 | 54361 | WNT4 | NM_030761 | 182873 | 196966 | 0.78 | 0.79 | 0.30 | 0.42 |
| 44 | F08 | 81029 | WNT5B | NM_030775 | 187663 | 191668 | 0.80 | 0.77 | 1.09 | 1.56 |
| 44 | F09 | 7475 | WNT6 | NM_006522 | 191625 | 196956 | 0.81 | 0.79 | -0.10 | 0.13 |
| 44 | F10 | 7477 | WNT7B | NM_058238 | 186898 | 199916 | 0.79 | 0.80 | 0.15 | -0.47 |
| 44 | F11 | 7478 | WNT8A | NM_031933 | 198849 | 201606 | 0.85 | 0.81 | -0.61 | -0.14 |
| 44 | F12 | 7479 | WNT8B | NM_003393 | 191390 | 195496 | 0.81 | 0.79 | 0.20 | 1.84 |
| 44 | G01 | NA | neg | NA | 242761 | 255443 | 1.03 | 1.03 | -4.37 | -5.60 |
| 44 | G02 | NA | neg | NA | 217762 | 243242 | 0.93 | 0.98 | -1.71 | -4.06 |
| 44 | G03 | 7483 | WNT9A | NM_003395 | 207651 | 225775 | 0.88 | 0.91 | 0.56 | 0.56 |
| 44 | G04 | 7484 | WNT9B | NM_003396 | 217774 | 196779 | 0.93 | 0.79 | -1.04 | 3.23 |
| 44 | G05 | 9466 | IL27RA | NM_004843 | 204715 | 212400 | 0.87 | 0.85 | -0.14 | 0.98 |
| 44 | G06 | 7783 | ZP2 | NM_003460 | 207631 | 215990 | 0.88 | 0.87 | 0.17 | -0.57 |
| 44 | G07 | 133 | ADM | NM_001124 | 208800 | 206482 | 0.89 | 0.83 | -1.68 | 0.07 |
| 44 | G08 | 181 | AGRP | NM_001138 | 203723 | 211888 | 0.87 | 0.85 | 0.16 | -0.14 |
| 44 | G09 | 11216 | AKAP10 | NM_007202 | 198955 | 211889 | 0.85 | 0.85 | -0.10 | -0.90 |
| 44 | G10 | 10142 | AKAP9 | NM_005751 | 206705 | 200312 | 0.88 | 0.80 | -1.19 | 0.34 |
| 44 | G11 | 214 | ALCAM | NM_001627 | 190795 | 207534 | 0.81 | 0.83 | 1.02 | -0.04 |
| 44 | G12 | 268 | AMH | NM_000479 | 196753 | 218845 | 0.84 | 0.88 | 0.40 | -0.26 |
| 44 | H01 | NA | NA | NA | 244435 | 247319 | 1.04 | 0.99 | -2.78 | -2.36 |
| 44 | H02 | NA | pos | NA | 78949 | 106919 | 0.34 | 0.43 | 14.84 | 15.36 |
| 44 | H03 | 284 | ANGPT1 | NM_001146 | 252157 | 267495 | 1.07 | 1.07 | -2.41 | -2.50 |
| 44 | H04 | 285 | ANGPT2 | NM_001147 | 237128 | 246053 | 1.01 | 0.99 | -1.33 | -0.78 |

| | | | | | | | | | | |
|---|---|---|---|---|---|---|---|---|---|---|
| 44 | H05 | 9068 | ANGPTL1 | NM_004673 | 230073 | 257437 | 0.98 | 1.03 | -1.07 | -2.50 |
| 44 | H06 | 23452 | ANGPTL2 | NM_012098 | 230675 | 230263 | 0.98 | 0.93 | -0.51 | -0.17 |
| 44 | H07 | 23780 | APOL2 | NM_030882 | 208540 | 225104 | 0.89 | 0.90 | 0.12 | -0.07 |
| 44 | H08 | 374 | AREG | NM_001657 | 213470 | 220698 | 0.91 | 0.89 | 0.89 | 0.96 |
| 44 | H09 | 433 | ASGR2 | NM_001181 | 206272 | 221718 | 0.88 | 0.89 | 0.89 | 0.07 |
| 44 | H10 | 434 | ASIP | NM_001672 | 213555 | 219492 | 0.91 | 0.88 | -0.15 | 0.12 |
| 44 | H11 | 551 | AVP | NM_000490 | 211081 | 198887 | 0.90 | 0.80 | 0.63 | 3.26 |
| 44 | H12 | 9530 | BAG4 | NM_004874 | 210743 | 214942 | 0.90 | 0.86 | 0.68 | 2.45 |
| 45 | A01 | NA | pos | NA | 61698 | 63687 | 0.29 | 0.26 | 18.82 | 13.11 |
| 45 | A02 | NA | NA | NA | 192429 | 201584 | 0.89 | 0.81 | 0.23 | -0.45 |
| 45 | A03 | 8915 | BCL10 | NM_003921 | 185790 | 205050 | 0.86 | 0.83 | 1.87 | 0.32 |
| 45 | A04 | 627 | BDNF | NM_001709 | 194066 | 195907 | 0.90 | 0.79 | 0.11 | 0.41 |
| 45 | A05 | 9210 | BMP15 | NM_005448 | 209375 | 213522 | 0.97 | 0.86 | -1.39 | -1.65 |
| 45 | A06 | 650 | BMP2 | NM_001200 | 201362 | 211357 | 0.93 | 0.85 | -1.27 | -1.51 |
| 45 | A07 | 651 | BMP3 | NM_001201 | 180030 | 205505 | 0.84 | 0.83 | 1.99 | -1.64 |
| 45 | A08 | 652 | BMP4 | NM_001202 | 194292 | 198608 | 0.90 | 0.80 | -0.05 | 0.11 |
| 45 | A09 | 653 | BMP5 | NM_021073 | 184850 | 185461 | 0.86 | 0.75 | 0.04 | 0.70 |
| 45 | A10 | 654 | BMP6 | NM_001718 | 199154 | 198224 | 0.92 | 0.80 | -1.13 | -0.07 |
| 45 | A11 | 655 | BMP7 | NM_001719 | 187471 | 186878 | 0.87 | 0.75 | 0.06 | 0.76 |
| 45 | A12 | 656 | BMP8B | NM_001720 | 199611 | 219602 | 0.93 | 0.89 | -0.40 | -1.99 |
| 45 | B01 | NA | neg | NA | 248754 | 265457 | 1.15 | 1.07 | -5.22 | -4.55 |
| 45 | B02 | NA | neg | NA | 206013 | 237831 | 0.96 | 0.96 | 0.86 | -1.83 |
| 45 | B03 | 9256 | BZRAP1 | NM_004758 | 211648 | 227345 | 0.98 | 0.92 | 0.76 | 0.30 |
| 45 | B04 | 706 | BZRP | NM_000714 | 213661 | 227221 | 0.99 | 0.92 | -0.11 | -0.49 |
| 45 | B05 | 54360 | C17 | NM_018659 | 225029 | 218238 | 1.04 | 0.88 | -1.05 | 0.06 |
| 45 | B06 | 22918 | C1QR1 | NM_012072 | 213427 | 225437 | 0.99 | 0.91 | -0.42 | -0.72 |
| 45 | B07 | 885 | CCK | NM_000729 | 215890 | 210957 | 1.00 | 0.85 | -0.54 | 0.00 |
| 45 | B08 | 6346 | CCL1 | NM_002981 | 202237 | 217701 | 0.94 | 0.88 | 1.39 | 0.41 |
| 45 | B09 | 6356 | CCL11 | NM_002986 | 206241 | 215056 | 0.96 | 0.87 | -0.44 | -0.04 |
| 45 | B10 | 6357 | CCL13 | NM_005408 | 208425 | 224351 | 0.97 | 0.91 | 0.12 | -0.47 |
| 45 | B11 | 6358 | CCL14 | NM_004166 | 198221 | 212288 | 0.92 | 0.86 | 1.10 | 0.44 |
| 45 | B12 | 6359 | CCL15 | NM_004167 | 205134 | 221553 | 0.95 | 0.89 | 1.38 | 0.00 |
| 45 | C01 | NA | pos | NA | 75026 | 78298 | 0.35 | 0.32 | 18.37 | 13.03 |
| 45 | C02 | NA | NA | NA | 208869 | 238989 | 0.97 | 0.97 | -0.67 | -2.77 |
| 45 | C03 | 6360 | CCL16 | NM_004590 | 207060 | 207451 | 0.96 | 0.84 | 0.28 | 1.44 |
| 45 | C04 | 6362 | CCL18 | NM_002988 | 193286 | 197940 | 0.90 | 0.80 | 1.66 | 1.56 |
| 45 | C05 | 6363 | CCL19 | NM_006274 | 208044 | 208115 | 0.97 | 0.84 | 0.24 | 0.23 |
| 45 | C06 | 6364 | CCL20 | NM_004591 | 193150 | 191626 | 0.90 | 0.77 | 1.34 | 1.78 |
| 45 | C07 | 6366 | CCL21 | NM_002989 | 209641 | 201927 | 0.97 | 0.82 | -0.78 | 0.07 |
| 45 | C08 | 6367 | CCL22 | NM_002990 | 196314 | 214056 | 0.91 | 0.86 | 1.10 | -0.06 |
| 45 | C09 | 6369 | CCL24 | NM_002991 | 207787 | 209041 | 0.96 | 0.84 | -1.79 | -0.27 |
| 45 | C10 | 10344 | CCL26 | NM_006072 | 202950 | 215089 | 0.94 | 0.87 | -0.23 | -0.38 |
| 45 | C11 | 10850 | CCL27 | NM_006664 | 219441 | 219972 | 1.02 | 0.89 | -3.05 | -1.14 |
| 45 | C12 | 56477 | CCL28 | NM_019846 | 211653 | 233350 | 0.98 | 0.94 | -0.67 | -1.99 |
| 45 | D01 | NA | neg | NA | 217832 | 246793 | 1.01 | 1.00 | -1.29 | -3.27 |
| 45 | D02 | NA | neg | NA | 212992 | 264665 | 0.99 | 1.07 | -0.60 | -5.03 |
| 45 | D03 | 6349 | CCL3L1 | NM_021006 | 205455 | 201794 | 0.95 | 0.82 | 1.16 | 2.26 |
| 45 | D04 | 6352 | CCL5 | NM_002985 | 193557 | 201896 | 0.90 | 0.82 | 2.28 | 1.44 |
| 45 | D05 | 6354 | CCL7 | NM_006273 | 226342 | 216709 | 1.05 | 0.88 | -1.71 | -0.34 |
| 45 | D06 | 6355 | CCL8 | NM_005623 | 197630 | 206943 | 0.92 | 0.84 | 1.35 | 0.54 |
| 45 | D07 | 929 | CD14 | NM_000591 | 208543 | 218736 | 0.97 | 0.88 | 0.03 | -1.32 |
| 45 | D08 | 912 | CD1D | NM_001766 | 210174 | 215628 | 0.98 | 0.87 | -0.21 | 0.06 |
| 45 | D09 | 914 | CD2 | NM_001767 | 210802 | 219471 | 0.98 | 0.89 | -1.56 | -1.03 |
| 45 | D10 | 10332 | CD209L | NM_014257 | 211041 | 213227 | 0.98 | 0.86 | -0.73 | 0.07 |
| 45 | D11 | 940 | CD28 | NM_006139 | 202718 | 215597 | 0.94 | 0.87 | -0.01 | -0.44 |
| 45 | D12 | 945 | CD33 | NM_001772 | 211440 | 216306 | 0.98 | 0.87 | 0.01 | -0.04 |
| 45 | E01 | NA | neg | NA | 236801 | 248321 | 1.10 | 1.00 | -5.23 | -4.17 |
| 45 | E02 | NA | neg | NA | 205176 | 222202 | 0.95 | 0.90 | -0.73 | -1.60 |
| 45 | E03 | 948 | CD36 | NM_000072 | 208374 | 220939 | 0.97 | 0.89 | -0.49 | -0.37 |
| 45 | E04 | 915 | CD3D | NM_000732 | 194424 | 222399 | 0.90 | 0.90 | 0.91 | -1.32 |
| 45 | E05 | 917 | CD3G | NM_000073 | 186363 | 188733 | 0.87 | 0.76 | 2.73 | 1.66 |
| 45 | E06 | 919 | CD3Z | NM_000734 | 175511 | 191696 | 0.81 | 0.77 | 3.26 | 1.30 |
| 45 | E07 | 960 | CD44 | NM_000610 | 199187 | 197816 | 0.92 | 0.80 | 0.12 | 0.00 |
| 45 | E08 | 921 | CD5 | NM_014207 | 206949 | 194333 | 0.96 | 0.79 | -1.00 | 1.41 |
| 45 | E09 | 923 | CD6 | NM_006725 | 180401 | 212477 | 0.84 | 0.86 | 1.52 | -1.08 |
| 45 | E10 | 969 | CD69 | NM_001781 | 198004 | 201216 | 0.92 | 0.81 | -0.12 | 0.51 |
| 45 | E11 | 971 | CD72 | NM_001782 | 198477 | 209121 | 0.92 | 0.84 | -0.65 | -0.55 |
| 45 | E12 | 925 | CD8A | NM_001768 | 204928 | 208245 | 0.95 | 0.84 | -0.30 | 0.00 |
| 45 | F01 | NA | NA | NA | 230461 | 247888 | 1.07 | 1.00 | -4.06 | -3.83 |
| 45 | F02 | NA | pos | NA | 73252 | 106555 | 0.34 | 0.43 | 18.30 | 10.07 |
| 45 | F03 | 926 | CD8B1 | NM_004931 | 214296 | 232858 | 0.99 | 0.94 | -1.07 | -1.25 |
| 45 | F04 | 8099 | CDK2AP1 | NM_004642 | 219952 | 222033 | 1.02 | 0.90 | -2.45 | -0.99 |
| 45 | F05 | 1081 | CGA | NM_000735 | 207623 | 230164 | 0.96 | 0.93 | -0.02 | -2.12 |
| 45 | F06 | 1114 | CHGB | NM_001819 | 204641 | 195231 | 0.95 | 0.79 | -0.62 | 1.24 |
| 45 | F07 | 51192 | CKLF | NM_016326 | 184156 | 186300 | 0.85 | 0.75 | 2.53 | 1.42 |
| 45 | F08 | 112616 | CKLFSF7 | NM_138410 | 198232 | 225745 | 0.92 | 0.91 | 0.51 | -1.39 |
| 45 | F09 | 23529 | CLC | NM_013246 | 184779 | 194739 | 0.86 | 0.79 | 1.17 | 0.95 |
| 45 | F10 | 51266 | CLEC2 | NM_016509 | 172135 | 201657 | 0.80 | 0.81 | 3.83 | 0.76 |
| 45 | F11 | 338339 | CLECSF8 | NM_080387 | 195719 | 211503 | 0.91 | 0.85 | 0.01 | -0.49 |
| 45 | F12 | 1270 | CNTF | NM_000614 | 204763 | 206185 | 0.95 | 0.83 | -0.01 | 0.50 |
| 45 | G01 | NA | neg | NA | 205193 | 250443 | 0.95 | 1.01 | -0.68 | -3.54 |
| 45 | G02 | NA | neg | NA | 221176 | 221530 | 1.03 | 0.89 | -2.96 | -0.69 |
| 45 | G03 | 1378 | CR1 | NM_000573 | 220153 | 228813 | 1.02 | 0.92 | -2.12 | -0.30 |
| 45 | G04 | 1380 | CR2 | NM_001877 | 207529 | 214734 | 0.96 | 0.87 | -0.90 | 0.27 |
| 45 | G05 | 1392 | CRH | NM_000756 | 205787 | 209026 | 0.96 | 0.84 | 0.02 | 0.50 |
| 45 | G06 | 1435 | CSF1 | NM_000757 | 205695 | 218899 | 0.95 | 0.88 | -0.98 | -0.54 |
| 45 | G07 | 1437 | CSF2 | NM_000758 | 200600 | 209711 | 0.93 | 0.85 | -0.03 | -0.34 |
| 45 | G08 | 1439 | CSF2RB | NM_000395 | 201331 | 227957 | 0.93 | 0.92 | -0.14 | -1.06 |

| | | | | | | | | | | |
|---|---|---|---|---|---|---|---|---|---|---|
| 45 | G09 | 1440 | CSF3 | NM_000759 | 188834 | 198055 | 0.88 | 0.80 | 0.38 | 1.17 |
| 45 | G10 | 1441 | CSF3R | NM_000760 | 193575 | 226699 | 0.90 | 0.92 | 0.57 | -1.16 |
| 45 | G11 | 1444 | CSHL1 | NM_001318 | 184495 | 204789 | 0.86 | 0.83 | 1.39 | 0.71 |
| 45 | G12 | 1489 | CTF1 | NM_001330 | 183610 | 207266 | 0.85 | 0.84 | 2.78 | 0.94 |
| 45 | H01 | NA | NA | NA | 246105 | 247163 | 1.14 | 1.00 | -5.14 | -1.91 |
| 45 | H02 | NA | pos | NA | 74944 | 97654 | 0.35 | 0.39 | 19.21 | 12.80 |
| 45 | H03 | 6376 | CX3CL1 | NM_002996 | 216868 | 252108 | 1.01 | 1.02 | -0.28 | -1.29 |
| 45 | H04 | 6373 | CXCL11 | NM_005409 | 227555 | 233540 | 1.06 | 0.94 | -2.38 | -0.27 |
| 45 | H05 | 10563 | CXCL13 | NM_006419 | 215365 | 228072 | 1.00 | 0.92 | 0.03 | -0.06 |
| 45 | H06 | 26999 | CYFIP2 | NM_014376 | 205461 | 241980 | 0.95 | 0.98 | 0.42 | -1.50 |
| 45 | H07 | 1605 | DAG1 | NM_004393 | 224667 | 215146 | 1.04 | 0.87 | -2.09 | 0.44 |
| 45 | H08 | 1622 | DBI | NM_020548 | 209577 | 232122 | 0.97 | 0.94 | 0.05 | -0.17 |
| 45 | H09 | 9936 | DCL-1 | NM_014880 | 201355 | 222872 | 0.93 | 0.90 | -0.04 | 0.04 |
| 45 | H10 | 9993 | DGCR2 | NM_005137 | 198775 | 214056 | 0.92 | 0.86 | 1.19 | 1.39 |
| 45 | H11 | 1729 | DIAPH1 | NM_005219 | 205425 | 206735 | 0.95 | 0.84 | -0.22 | 1.83 |
| 45 | H12 | 1325 | CORT | NM_001302 | 167091 | 184501 | 0.78 | 0.75 | 6.50 | 4.48 |
| 46 | A01 | NA | pos | NA | 67921 | 75893 | 0.31 | 0.34 | 19.35 | 16.54 |
| 46 | A02 | NA | NA | NA | 211440 | 243012 | 0.96 | 1.08 | -1.90 | -5.11 |
| 46 | A03 | 9231 | DLG5 | NM_004747 | 210724 | 224807 | 0.96 | 1.00 | -1.82 | -1.93 |
| 46 | A04 | 10683 | DLL3 | NM_016941 | 198420 | 194998 | 0.90 | 0.86 | 0.60 | 2.88 |
| 46 | A05 | 2352 | FOLR3 | NM_000804 | 205418 | 204767 | 0.93 | 0.91 | 0.03 | 0.12 |
| 46 | A06 | 10817 | FRS3 | NM_006653 | 204612 | 194754 | 0.93 | 0.86 | -0.18 | 0.32 |
| 46 | A07 | 2487 | FRZB | NM_001463 | 191544 | 212475 | 0.87 | 0.94 | 2.12 | -0.10 |
| 46 | A08 | 2488 | FSHB | NM_000510 | 190146 | 198596 | 0.86 | 0.88 | 0.13 | 0.39 |
| 46 | A09 | 2549 | GAB1 | NM_002039 | 193180 | 199912 | 0.88 | 0.89 | 0.85 | -0.56 |
| 46 | A10 | 2520 | GAS | NM_000805 | 198730 | 199572 | 0.90 | 0.88 | -1.60 | -0.56 |
| 46 | A11 | 2657 | GDF1 | NM_001492 | 193686 | 205158 | 0.88 | 0.91 | -0.03 | -1.02 |
| 46 | A12 | 2662 | GDF10 | NM_004962 | 197168 | 209989 | 0.89 | 0.93 | -0.30 | 0.12 |
| 46 | B01 | NA | neg | NA | 264440 | 242460 | 1.20 | 1.07 | -6.86 | -3.50 |
| 46 | B02 | NA | neg | NA | 214689 | 222287 | 0.97 | 0.99 | 0.50 | -0.88 |
| 46 | B03 | 10220 | GDF11 | NM_005811 | 205996 | 217751 | 0.93 | 0.97 | 1.76 | 0.52 |
| 46 | B04 | 9518 | GDF15 | NM_004864 | 221639 | 215361 | 1.00 | 0.95 | 0.05 | 1.78 |
| 46 | B05 | 2658 | GDF2 | NM_016204 | 224556 | 218510 | 1.02 | 0.97 | 0.08 | -0.12 |
| 46 | B06 | 8200 | GDF5 | NM_000557 | 233715 | 233495 | 1.06 | 1.03 | -1.60 | -3.16 |
| 46 | B07 | 2660 | GDF8 | NM_005259 | 235006 | 222487 | 1.07 | 0.99 | -1.43 | 0.15 |
| 46 | B08 | 2661 | GDF9 | NM_005260 | 229984 | 218728 | 1.04 | 0.97 | -2.88 | -0.68 |
| 46 | B09 | 2668 | GDNF | NM_000514 | 216368 | 200336 | 0.98 | 0.89 | 0.31 | 0.92 |
| 46 | B10 | 2676 | GFRA3 | NM_001496 | 213782 | 212015 | 0.97 | 0.94 | -0.94 | -0.64 |
| 46 | B11 | 2689 | GH2 | NM_002059 | 210957 | 205479 | 0.96 | 0.91 | 0.30 | 0.47 |
| 46 | B12 | 2691 | GHRH | NM_021081 | 214982 | 226750 | 0.97 | 1.00 | -0.05 | -0.52 |
| 46 | C01 | NA | pos | NA | 70897 | 90283 | 0.32 | 0.40 | 20.96 | 16.46 |
| 46 | C02 | NA | NA | NA | 218635 | 224852 | 0.99 | 1.00 | -0.91 | -0.97 |
| 46 | C03 | 51738 | GHRL | NM_016362 | 229178 | 214411 | 1.04 | 0.95 | -2.50 | 1.20 |
| 46 | C04 | 2695 | GIP | NM_004123 | 217072 | 238603 | 0.98 | 1.06 | -0.11 | -0.98 |
| 46 | C05 | 2734 | GLG1 | NM_012201 | 192638 | 196555 | 0.87 | 0.87 | 3.98 | 2.97 |
| 46 | C06 | 2796 | GNRH1 | NM_000825 | 211862 | 199085 | 0.96 | 0.88 | 0.80 | 1.54 |
| 46 | C07 | 2797 | GNRH2 | NM_001501 | 215656 | 225668 | 0.98 | 1.00 | 0.60 | -0.02 |
| 46 | C08 | 2811 | GP1BA | NM_000173 | 201531 | 202806 | 0.91 | 0.90 | 0.50 | 1.62 |
| 46 | C09 | 51206 | GP6 | NM_016363 | 215955 | 214391 | 0.98 | 0.95 | -0.46 | -0.65 |
| 46 | C10 | 2885 | GRB2 | NM_002086 | 202065 | 217516 | 0.92 | 0.96 | -0.04 | -1.11 |
| 46 | C11 | 2922 | GRP | NM_002091 | 207235 | 210852 | 0.94 | 0.93 | 0.03 | 0.02 |
| 46 | C12 | 57817 | HAMP | NM_021175 | 218673 | 227576 | 0.99 | 1.01 | -1.43 | -0.38 |
| 46 | D01 | NA | neg | NA | 231419 | 253249 | 1.05 | 1.12 | -2.04 | -5.59 |
| 46 | D02 | NA | neg | NA | 224004 | 210186 | 1.02 | 0.93 | -0.94 | -0.01 |
| 46 | D03 | 3068 | HDGF | NM_004494 | 221751 | 213669 | 1.01 | 0.95 | -0.64 | 0.35 |
| 46 | D04 | 3108 | HLA-DMA | NM_006120 | 213239 | 223897 | 0.97 | 0.99 | 1.22 | -0.02 |
| 46 | D05 | 9324 | HMGN3 | NM_004242 | 208065 | 196985 | 0.94 | 0.87 | 2.46 | 1.97 |
| 46 | D06 | 3375 | IAPP | NM_000415 | 195552 | 188050 | 0.89 | 0.83 | 3.98 | 2.03 |
| 46 | D07 | 3383 | ICAM1 | NM_000201 | 228964 | 218051 | 1.04 | 0.97 | -0.60 | 0.02 |
| 46 | D08 | 3384 | ICAM2 | NM_000873 | 206083 | 210898 | 0.93 | 0.93 | 0.59 | -0.37 |
| 46 | D09 | 3385 | ICAM3 | NM_002162 | 225443 | 196743 | 1.02 | 0.87 | -1.11 | 0.69 |
| 46 | D10 | 3446 | IFNA10 | NM_002171 | 215751 | 205127 | 0.98 | 0.91 | -1.30 | -0.44 |
| 46 | D11 | 3447 | IFNA13 | NM_006900 | 222769 | 212284 | 1.01 | 0.94 | -1.51 | -1.11 |
| 46 | D12 | 3448 | IFNA14 | NM_002172 | 208749 | 218256 | 0.95 | 0.97 | 0.81 | -0.12 |
| 46 | E01 | NA | neg | NA | 220968 | 266108 | 1.00 | 1.18 | -2.96 | -8.86 |
| 46 | E02 | NA | neg | NA | 219589 | 227820 | 1.00 | 1.01 | -2.75 | -3.90 |
| 46 | E03 | 3449 | IFNA16 | NM_002173 | 189499 | 215566 | 0.86 | 0.96 | 1.67 | -1.50 |
| 46 | E04 | 3451 | IFNA17 | NM_021268 | 200923 | 205428 | 0.91 | 0.91 | 0.58 | 0.77 |
| 46 | E05 | 3440 | IFNA2 | NM_000605 | 228238 | 201900 | 1.03 | 0.89 | -3.00 | -0.27 |
| 46 | E06 | 3452 | IFNA21 | NM_002175 | 210348 | 184819 | 0.95 | 0.82 | -0.68 | 0.85 |
| 46 | E07 | 3441 | IFNA4 | NM_021068 | 194512 | 220189 | 0.88 | 0.98 | 2.03 | -1.86 |
| 46 | E08 | 3442 | IFNA5 | NM_002169 | 182726 | 165066 | 0.83 | 0.73 | 1.58 | 3.97 |
| 46 | E09 | 3443 | IFNA6 | NM_021002 | 203381 | 202199 | 0.92 | 0.90 | -0.31 | -1.62 |
| 46 | E10 | 3445 | IFNA8 | NM_002170 | 189313 | 183755 | 0.86 | 0.81 | 0.15 | 0.72 |
| 46 | E11 | 3454 | IFNAR1 | NM_000629 | 196981 | 190177 | 0.89 | 0.84 | -0.16 | 0.16 |
| 46 | E12 | 3455 | IFNAR2 | NM_000874 | 199340 | 206116 | 0.90 | 0.91 | -0.27 | -0.14 |
| 46 | F01 | NA | NA | NA | 216447 | 247654 | 0.98 | 1.10 | -0.44 | -4.93 |
| 46 | F02 | NA | pos | NA | 86882 | 107890 | 0.39 | 0.48 | 18.75 | 13.17 |
| 46 | F03 | 3456 | IFNB1 | NM_002176 | 217426 | 218553 | 0.99 | 0.97 | -0.61 | -0.35 |
| 46 | F04 | 3458 | IFNG | NM_000619 | 229158 | 239463 | 1.04 | 1.06 | -1.75 | -2.10 |
| 46 | F05 | 3459 | IFNGR1 | NM_000416 | 230175 | 234745 | 1.04 | 1.04 | -1.43 | -2.99 |
| 46 | F06 | 3467 | IFNW1 | NM_002177 | 214042 | 205689 | 0.97 | 0.91 | 0.63 | -0.32 |
| 46 | F07 | 3479 | IGF1 | NM_000618 | 213814 | 200600 | 0.97 | 0.89 | 1.03 | 2.21 |
| 46 | F08 | 3481 | IGF2 | NM_000612 | 217212 | 206304 | 0.98 | 0.91 | -1.67 | 0.16 |
| 46 | F09 | 3550 | IK | NM_006083 | 148116 | 144445 | 0.67 | 0.64 | 9.73 | 7.40 |
| 46 | F10 | 8517 | IKBKG | NM_003639 | 195991 | 192071 | 0.89 | 0.85 | 1.01 | 1.18 |
| 46 | F11 | 3586 | IL10 | NM_000572 | 217422 | 204563 | 0.99 | 0.91 | -1.33 | -0.17 |
| 46 | F12 | 3587 | IL10RA | NM_001558 | 205446 | 208386 | 0.93 | 0.92 | 0.68 | 1.09 |

| | | | | | | | | | | |
|---|---|---|---|---|---|---|---|---|---|---|
| 46 | G01 | NA | neg | NA | 220251 | 220332 | 1.00 | 0.98 | -1.79 | -2.09 |
| 46 | G02 | NA | neg | NA | 211303 | 223451 | 0.96 | 0.99 | -0.47 | -2.49 |
| 46 | G03 | 3588 | IL10RB | NM_000628 | 203828 | 197235 | 0.92 | 0.87 | 0.61 | 1.72 |
| 46 | G04 | 3589 | IL11 | NM_000641 | 220917 | 217748 | 1.00 | 0.97 | -1.32 | 0.02 |
| 46 | G05 | 3593 | IL12B | NM_002187 | 215370 | 208867 | 0.98 | 0.93 | -0.03 | -0.33 |
| 46 | G06 | 3594 | IL12RB1 | NM_005535 | 212636 | 220689 | 0.96 | 0.98 | 0.04 | -2.95 |
| 46 | G07 | 3595 | IL12RB2 | NM_001559 | 220929 | 207412 | 1.00 | 0.92 | -0.82 | 0.64 |
| 46 | G08 | 3598 | IL13RA2 | NM_000640 | 215073 | 203422 | 0.97 | 0.90 | -2.14 | -0.16 |
| 46 | G09 | 3600 | IL15 | NM_000585 | 210559 | 212223 | 0.95 | 0.94 | -0.31 | -2.07 |
| 46 | G10 | 3601 | IL15RA | NM_002189 | 197190 | 185316 | 0.89 | 0.82 | 0.04 | 1.36 |
| 46 | G11 | 3603 | IL16 | NM_004513 | 189314 | 198062 | 0.86 | 0.88 | 2.04 | -0.02 |
| 46 | G12 | 3605 | IL17 | NM_002190 | 198701 | 185222 | 0.90 | 0.82 | 0.89 | 3.40 |
| 46 | H01 | NA | NA | NA | 233509 | 249515 | 1.06 | 1.11 | -2.52 | -1.05 |
| 46 | H02 | NA | pos | NA | 238708 | 265889 | 1.08 | 1.18 | -3.29 | -3.17 |
| 46 | H03 | 27190 | IL17B | NM_014443 | 200142 | 251196 | 0.91 | 1.11 | 2.39 | -0.45 |
| 46 | H04 | 27189 | IL17C | NM_013278 | 220655 | 270625 | 1.00 | 1.20 | -0.05 | -2.01 |
| 46 | H05 | 23765 | IL17R | NM_014339 | 228527 | 237826 | 1.04 | 1.05 | -0.74 | 0.74 |
| 46 | H06 | 55540 | IL17RB | NM_018725 | 221573 | 241623 | 1.00 | 1.07 | -0.04 | -0.85 |
| 46 | H07 | 3606 | IL18 | NM_001562 | 228888 | 264149 | 1.04 | 1.17 | -0.76 | -1.89 |
| 46 | H08 | 10068 | IL18BP | NM_173043 | 209833 | 243324 | 0.95 | 1.08 | -0.13 | -0.51 |
| 46 | H09 | 8809 | IL18R1 | NM_003855 | 208352 | 229103 | 0.94 | 1.02 | 1.26 | 0.56 |
| 46 | H10 | 29949 | IL19 | NM_013371 | 205583 | 229633 | 0.93 | 1.02 | 0.04 | 0.44 |
| 46 | H11 | 3552 | IL1A | NM_000575 | 205495 | 211001 | 0.93 | 0.94 | 0.88 | 3.12 |
| 46 | H12 | 3553 | IL1B | NM_000576 | 212723 | 221478 | 0.96 | 0.98 | 0.05 | 3.53 |
| 47 | A01 | NA | pos | NA | 58301 | 63558 | 0.25 | 0.28 | 20.15 | 17.23 |
| 47 | A02 | NA | NA | NA | 218421 | 209678 | 0.94 | 0.92 | -1.58 | -0.34 |
| 47 | A03 | 26525 | IL1F5 | NM_012275 | 208446 | 209844 | 0.90 | 0.92 | 1.33 | 0.80 |
| 47 | A04 | 27179 | IL1F6 | NM_014440 | 197645 | 200295 | 0.85 | 0.88 | 2.91 | 1.21 |
| 47 | A05 | 27178 | IL1F7 | NM_014439 | 191519 | 216843 | 0.83 | 0.95 | 2.30 | 0.06 |
| 47 | A06 | 27177 | IL1F8 | NM_014438 | 211735 | 213430 | 0.91 | 0.93 | -0.16 | -0.56 |
| 47 | A07 | 56300 | IL1F9 | NM_019618 | 204046 | 202219 | 0.88 | 0.89 | 0.18 | 0.78 |
| 47 | A08 | 3554 | IL1R1 | NM_000877 | 195032 | 190532 | 0.84 | 0.83 | 1.30 | 1.34 |
| 47 | A09 | 7850 | IL1R2 | NM_004633 | 208243 | 211067 | 0.90 | 0.92 | -0.62 | -0.94 |
| 47 | A10 | 3556 | IL1RAP | NM_002182 | 209267 | 216402 | 0.90 | 0.95 | -0.64 | -1.64 |
| 47 | A11 | 26280 | IL1RAPL2 | NM_017416 | 203216 | 197256 | 0.88 | 0.86 | -0.54 | -0.03 |
| 47 | A12 | 9173 | IL1RL1 | NM_003856 | 210192 | 215146 | 0.91 | 0.94 | -0.24 | -1.25 |
| 47 | B01 | NA | neg | NA | 234193 | 234118 | 1.01 | 1.03 | -2.20 | -2.99 |
| 47 | B02 | NA | neg | NA | 219934 | 222489 | 0.95 | 0.97 | -0.27 | -1.59 |
| 47 | B03 | 8808 | IL1RL2 | NM_003854 | 227608 | 218148 | 0.98 | 0.96 | 0.25 | 0.10 |
| 47 | B04 | 3557 | IL1RN | NM_000577 | 219258 | 207867 | 0.95 | 0.91 | 1.49 | 0.60 |
| 47 | B05 | 50615 | IL21R | NM_021798 | 221225 | 215011 | 0.95 | 0.94 | -0.21 | 0.57 |
| 47 | B06 | 50616 | IL22 | NM_020525 | 229862 | 221512 | 0.99 | 0.97 | -1.11 | -1.23 |
| 47 | B07 | 58985 | IL22RA1 | NM_021258 | 220239 | 219236 | 0.95 | 0.96 | -0.50 | -0.97 |
| 47 | B08 | 11009 | IL24 | NM_006850 | 225621 | 227574 | 0.97 | 1.00 | -1.33 | -2.81 |
| 47 | B09 | 55801 | IL26 | NM_018402 | 221688 | 218363 | 0.96 | 0.96 | -0.93 | -1.52 |
| 47 | B10 | 3559 | IL2RA | NM_000417 | 202565 | 205501 | 0.87 | 0.90 | 1.78 | -0.04 |
| 47 | B11 | 3560 | IL2RB | NM_000878 | 188792 | 199171 | 0.81 | 0.87 | 2.93 | 0.03 |
| 47 | B12 | 3561 | IL2RG | NM_000206 | 201086 | 200876 | 0.87 | 0.88 | 2.51 | 0.76 |
| 47 | C01 | NA | pos | NA | 72789 | 104832 | 0.31 | 0.46 | 16.93 | 13.91 |
| 47 | C02 | NA | NA | NA | 221210 | 213766 | 0.95 | 0.94 | -3.21 | 0.81 |
| 47 | C03 | 3562 | IL3 | NM_000588 | 210011 | 233104 | 0.91 | 1.02 | -0.14 | -0.34 |
| 47 | C04 | 3563 | IL3RA | NM_002183 | 202121 | 226128 | 0.87 | 0.99 | 1.05 | -0.24 |
| 47 | C05 | 3565 | IL4 | NM_000589 | 188668 | 231492 | 0.81 | 1.01 | 1.43 | -0.06 |
| 47 | C06 | 3566 | IL4R | NM_000418 | 200104 | 209644 | 0.86 | 0.92 | 0.16 | 1.55 |
| 47 | C07 | 3567 | IL5 | NM_000879 | 200468 | 227199 | 0.87 | 1.00 | -0.59 | -0.57 |
| 47 | C08 | 3568 | IL5RA | NM_000564 | 220035 | 225660 | 0.95 | 0.99 | -3.35 | -1.23 |
| 47 | C09 | 3569 | IL6 | NM_000600 | 176101 | 214767 | 0.76 | 0.94 | 2.48 | 0.26 |
| 47 | C10 | 3570 | IL6R | NM_000565 | 190405 | 190420 | 0.82 | 0.83 | 0.66 | 3.13 |
| 47 | C11 | 3572 | IL6ST | NM_002184 | 191786 | 198002 | 0.83 | 0.87 | -0.25 | 1.53 |
| 47 | C12 | 3574 | IL7 | NM_000880 | 211298 | 217951 | 0.91 | 0.95 | -1.64 | 0.06 |
| 47 | D01 | NA | neg | NA | 241023 | 242787 | 1.04 | 1.06 | -5.06 | -3.51 |
| 47 | D02 | NA | neg | NA | 229288 | 222386 | 0.99 | 0.97 | -3.47 | -1.06 |
| 47 | D03 | 3575 | IL7R | NM_002185 | 235303 | 229555 | 1.02 | 1.01 | -2.73 | -0.75 |
| 47 | D04 | 3578 | IL9 | NM_000590 | 217199 | 206438 | 0.94 | 0.90 | -0.16 | 1.29 |
| 47 | D05 | 3581 | IL9R | NM_002186 | 202008 | 214934 | 0.87 | 0.94 | 0.46 | 1.10 |
| 47 | D06 | 3623 | INHA | NM_002191 | 201635 | 227212 | 0.87 | 1.00 | 0.79 | -1.40 |
| 47 | D07 | 3624 | INHBA | NM_002192 | 202228 | 185897 | 0.87 | 0.81 | 0.01 | 3.56 |
| 47 | D08 | 3625 | INHBB | NM_002193 | 201631 | 217666 | 0.87 | 0.95 | -0.01 | -1.11 |
| 47 | D09 | 3626 | INHBC | NM_005538 | 199684 | 217426 | 0.86 | 0.95 | 0.12 | -0.89 |
| 47 | D10 | 3630 | INS | NM_000207 | 204853 | 208791 | 0.88 | 0.91 | -0.46 | 0.09 |
| 47 | D11 | 3640 | INSL3 | NM_005543 | 209560 | 204482 | 0.90 | 0.90 | -1.82 | -0.09 |
| 47 | D12 | 3641 | INSL4 | NM_002195 | 201682 | 198727 | 0.87 | 0.87 | 0.50 | 1.54 |
| 47 | E01 | NA | neg | NA | 249585 | 262748 | 1.08 | 1.15 | -6.60 | -6.47 |
| 47 | E02 | NA | neg | NA | 217858 | 216340 | 0.94 | 0.95 | -2.30 | -0.89 |
| 47 | E03 | 10022 | INSL5 | NM_005478 | 211916 | 219524 | 0.91 | 0.96 | 0.06 | -0.10 |
| 47 | E04 | 11172 | INSL6 | NM_007179 | 229685 | 224235 | 0.99 | 0.98 | -2.24 | -1.41 |
| 47 | E05 | 8660 | IRS2 | NM_003749 | 201974 | 198684 | 0.87 | 0.87 | 0.08 | 2.50 |
| 47 | E06 | 8471 | IRS4 | NM_003604 | 211823 | 197614 | 0.91 | 0.87 | -0.97 | 1.61 |
| 47 | E07 | 8515 | ITGA10 | NM_003637 | 192520 | 201022 | 0.83 | 0.88 | 0.94 | 1.18 |
| 47 | E08 | 3673 | ITGA2 | NM_002203 | 222528 | 204715 | 0.96 | 0.90 | -3.23 | -0.10 |
| 47 | E09 | 3674 | ITGA2B | NM_000419 | 198046 | 203806 | 0.85 | 0.89 | -0.04 | 0.19 |
| 47 | E10 | 3675 | ITGA3 | NM_002204 | 192294 | 204534 | 0.83 | 0.90 | 0.86 | 0.04 |
| 47 | E11 | 3676 | ITGA4 | NM_000885 | 191847 | 206051 | 0.83 | 0.90 | 0.20 | -0.83 |
| 47 | E12 | 3678 | ITGA5 | NM_002205 | 203285 | 209184 | 0.88 | 0.92 | -0.10 | -0.28 |
| 47 | F01 | NA | NA | NA | 239586 | 261109 | 1.03 | 1.14 | -5.30 | -5.16 |
| 47 | F02 | NA | pos | NA | 79553 | 126908 | 0.34 | 0.56 | 16.42 | 10.97 |
| 47 | F03 | 3655 | ITGA6 | NM_000210 | 212440 | 222354 | 0.92 | 0.97 | -0.06 | 0.66 |
| 47 | F04 | 3679 | ITGA7 | NM_002206 | 212896 | 227933 | 0.92 | 1.00 | -0.01 | -0.74 |

| | | | | | | | | | | |
|---|---|---|---|---|---|---|---|---|---|---|
| 47 | F05 | 8516 | ITGA8 | XM_167711 | 212195 | 238004 | 0.92 | 1.04 | -1.35 | -1.12 |
| 47 | F06 | 3680 | ITGA9 | NM_002207 | 199031 | 230178 | 0.86 | 1.01 | 0.71 | -1.20 |
| 47 | F07 | 3682 | ITGAE | NM_002208 | 198869 | 218767 | 0.86 | 0.96 | 0.03 | 0.16 |
| 47 | F08 | 3683 | ITGAL | NM_002209 | 198308 | 208409 | 0.86 | 0.91 | 0.01 | 0.56 |
| 47 | F09 | 3684 | ITGAM | NM_000632 | 190585 | 203086 | 0.82 | 0.89 | 0.93 | 1.38 |
| 47 | F10 | 3687 | ITGAX | NM_000887 | 202390 | 213795 | 0.87 | 0.94 | -0.56 | 0.04 |
| 47 | F11 | 3688 | ITGB1 | NM_002211 | 194435 | 211583 | 0.84 | 0.93 | -0.20 | -0.39 |
| 47 | F12 | 3689 | ITGB2 | NM_000211 | 199317 | 216586 | 0.86 | 0.95 | 0.39 | -0.06 |
| 47 | G01 | NA | neg | NA | 256794 | 247959 | 1.11 | 1.09 | -7.03 | -4.23 |
| 47 | G02 | NA | neg | NA | 215328 | 215891 | 0.93 | 0.95 | -1.40 | -0.38 |
| 47 | G03 | 3690 | ITGB3 | NM_000212 | 218390 | 210021 | 0.94 | 0.92 | -0.26 | 1.50 |
| 47 | G04 | 23421 | ITGB3BP | NM_014288 | 217199 | 214307 | 0.94 | 0.94 | 0.01 | 0.24 |
| 47 | G05 | 3691 | ITGB4 | NM_000213 | 214509 | 230888 | 0.93 | 1.01 | -1.06 | -0.92 |
| 47 | G06 | 3693 | ITGB5 | NM_002213 | 204722 | 194594 | 0.88 | 0.85 | 0.55 | 2.42 |
| 47 | G07 | 3694 | ITGB6 | NM_000888 | 203630 | 219554 | 0.88 | 0.96 | -0.01 | -0.59 |
| 47 | G08 | 3714 | JAG2 | NM_002226 | 198654 | 206801 | 0.86 | 0.91 | 0.57 | 0.10 |
| 47 | G09 | 4254 | KITLG | NM_000899 | 201568 | 210794 | 0.87 | 0.92 | 0.04 | -0.19 |
| 47 | G10 | 10748 | KLRA1 | NM_006611 | 205376 | 222330 | 0.89 | 0.97 | -0.36 | -1.64 |
| 47 | G11 | 8302 | KLRC4 | NM_013431 | 173937 | 189879 | 0.75 | 0.83 | 3.19 | 1.57 |
| 47 | G12 | 3824 | KLRD1 | NM_002262 | 216278 | 217247 | 0.93 | 0.95 | -1.31 | -0.79 |
| 47 | H01 | NA | NA | NA | 244536 | 250267 | 1.06 | 1.10 | -3.18 | -3.04 |
| 47 | H02 | NA | pos | NA | 83806 | 109914 | 0.36 | 0.48 | 18.63 | 13.84 |
| 47 | H03 | 51348 | KLRF1 | NM_016523 | 223435 | 251288 | 0.96 | 1.10 | 1.24 | -2.00 |
| 47 | H04 | 3930 | LBR | NM_002296 | 240226 | 238731 | 1.04 | 1.05 | -0.93 | -1.22 |
| 47 | H05 | 3952 | LEP | NM_000230 | 223415 | 245130 | 0.96 | 1.07 | -0.08 | -1.16 |
| 47 | H06 | 3956 | LGALS1 | NM_002305 | 235537 | 222341 | 1.02 | 0.97 | -1.45 | 0.56 |
| 47 | H07 | 3959 | LGALS3BP | NM_005567 | 232603 | 228176 | 1.00 | 1.00 | -1.75 | -0.16 |
| 47 | H08 | 3965 | LGALS9 | NM_002308 | 204126 | 208197 | 0.88 | 0.91 | 2.01 | 1.40 |
| 47 | H09 | 3972 | LHB | NM_000894 | 218459 | 213765 | 0.94 | 0.94 | -0.07 | 0.92 |
| 47 | H10 | 29121 | LLT1 | NM_013269 | 216226 | 224674 | 0.93 | 0.98 | 0.36 | -0.45 |
| 47 | H11 | 53353 | LRP1B | NM_018557 | 196785 | 196242 | 0.85 | 0.86 | 2.27 | 2.27 |
| 47 | H12 | 4036 | LRP2 | NM_004525 | 222003 | 221867 | 0.96 | 0.97 | 0.10 | 0.12 |
| 48 | A01 | NA | pos | NA | 73094 | 60011 | 0.32 | 0.27 | 17.12 | 15.09 |
| 48 | A02 | NA | NA | NA | 212148 | 221466 | 0.93 | 1.01 | -1.77 | -2.28 |
| 48 | A03 | 4040 | LRP6 | NM_002336 | 202863 | 216393 | 0.89 | 0.99 | 0.13 | -1.62 |
| 48 | A04 | 7804 | LRP8 | NM_004631 | 193263 | 204167 | 0.85 | 0.93 | 1.95 | -0.05 |
| 48 | A05 | 4043 | LRPAP1 | NM_002337 | 207654 | 206828 | 0.91 | 0.95 | -0.70 | -1.18 |
| 48 | A06 | 4046 | LSP1 | NM_002339 | 199927 | 200309 | 0.88 | 0.92 | -0.11 | 0.06 |
| 48 | A07 | 4049 | LTA | NM_000595 | 202553 | 218460 | 0.89 | 1.00 | -0.12 | -1.18 |
| 48 | A08 | 4050 | LTB | NM_002341 | 195915 | 198039 | 0.86 | 0.91 | 0.44 | 0.51 |
| 48 | A09 | 8425 | LTBP4 | NM_003573 | 201564 | 190364 | 0.89 | 0.87 | -1.47 | 0.10 |
| 48 | A10 | 4055 | LTBR | NM_002342 | 187876 | 184167 | 0.83 | 0.84 | 0.28 | 1.68 |
| 48 | A11 | 4064 | LY64 | NM_005582 | 177145 | 184969 | 0.78 | 0.85 | 0.48 | 0.25 |
| 48 | A12 | 4091 | SMAD6 | NM_005585 | 200720 | 210084 | 0.88 | 0.96 | -0.27 | -1.19 |
| 48 | B01 | NA | neg | NA | 236058 | 216871 | 1.04 | 0.99 | -3.44 | -0.35 |
| 48 | B02 | NA | neg | NA | 215282 | 220283 | 0.95 | 1.01 | -0.61 | -0.72 |
| 48 | B03 | 4192 | MDK | NM_002391 | 216360 | 214569 | 0.95 | 0.98 | -0.13 | 0.00 |
| 48 | B04 | 8190 | MIA | NM_006533 | 224601 | 226927 | 0.99 | 1.04 | -0.73 | -1.07 |
| 48 | B05 | 4360 | MRC1 | NM_002438 | 219511 | 209214 | 0.97 | 0.96 | -0.73 | -0.01 |
| 48 | B06 | 4615 | MYD88 | NM_002468 | 211115 | 226094 | 0.93 | 1.03 | -0.05 | -1.28 |
| 48 | B07 | 4690 | NCK1 | NM_006153 | 209718 | 233615 | 0.92 | 1.07 | 0.49 | -1.37 |
| 48 | B08 | 8440 | NCK2 | NM_003581 | 217613 | 208198 | 0.96 | 0.95 | -0.93 | 0.85 |
| 48 | B09 | 4693 | NDP | NM_000266 | 201811 | 198730 | 0.89 | 0.91 | 0.08 | 0.63 |
| 48 | B10 | 4803 | NGFB | NM_002506 | 196065 | 212903 | 0.86 | 0.97 | 0.75 | 0.02 |
| 48 | B11 | 4828 | NMB | NM_021077 | 185421 | 187119 | 0.82 | 0.86 | 0.94 | 1.45 |
| 48 | B12 | 10874 | NMU | NM_006681 | 209397 | 212359 | 0.92 | 0.97 | 0.13 | 0.00 |
| 48 | C01 | NA | pos | NA | 75778 | 95713 | 0.33 | 0.44 | 17.55 | 12.01 |
| 48 | C02 | NA | NA | NA | 212298 | 218514 | 0.93 | 1.00 | -0.99 | -1.20 |
| 48 | C03 | 7044 | EBAF | NM_003240 | 236855 | 222450 | 1.04 | 1.02 | -3.69 | -1.51 |
| 48 | C04 | 2812 | GP1BB | NM_000407 | 212348 | 210829 | 0.93 | 0.96 | 0.15 | -0.01 |
| 48 | C05 | 54567 | DLL4 | NM_019074 | 216578 | 202855 | 0.95 | 0.93 | -1.12 | 0.01 |
| 48 | C06 | 1755 | DMBT1 | NM_004406 | 200879 | 194838 | 0.88 | 0.89 | 0.56 | 1.41 |
| 48 | C07 | 9046 | DOK2 | NM_003974 | 216711 | 220200 | 0.95 | 1.01 | -1.24 | -0.60 |
| 48 | C08 | 55715 | DOK4 | NM_018110 | 198434 | 236006 | 0.87 | 1.08 | 0.90 | -2.81 |
| 48 | C09 | 55816 | DOK5 | NM_177959 | 185322 | 183677 | 0.81 | 0.84 | 1.53 | 1.58 |
| 48 | C10 | 1839 | DTR | NM_001945 | 189709 | 193498 | 0.83 | 0.88 | 0.83 | 1.44 |
| 48 | C11 | 1855 | DVL1 | NM_004421 | 203363 | 188870 | 0.89 | 0.86 | -2.28 | 0.59 |
| 48 | C12 | 1856 | DVL2 | NM_004422 | 205546 | 213759 | 0.90 | 0.98 | -0.13 | -0.82 |
| 48 | D01 | NA | neg | NA | 224310 | 244161 | 0.99 | 1.12 | -1.63 | -2.71 |
| 48 | D02 | NA | neg | NA | 231020 | 208757 | 1.02 | 0.95 | -2.54 | 1.10 |
| 48 | D03 | 1896 | ED1 | NM_001399 | 237621 | 231065 | 1.04 | 1.06 | -2.80 | -1.19 |
| 48 | D04 | 10085 | EDIL3 | NM_005711 | 212681 | 218280 | 0.94 | 1.00 | 1.10 | 0.44 |
| 48 | D05 | 1906 | EDN1 | NM_001955 | 202993 | 234850 | 0.89 | 1.07 | 1.72 | -2.19 |
| 48 | D06 | 1908 | EDN3 | NM_000114 | 211970 | 194898 | 0.93 | 0.89 | 0.05 | 2.65 |
| 48 | D07 | 1942 | EFNA1 | NM_004428 | 187290 | 226425 | 0.82 | 1.03 | 3.75 | -0.02 |
| 48 | D08 | 1943 | EFNA2 | NM_001405 | 212676 | 221538 | 0.94 | 1.01 | -0.04 | -0.01 |
| 48 | D09 | 1946 | EFNA5 | NM_001962 | 208638 | 206067 | 0.92 | 0.94 | -0.64 | 0.42 |
| 48 | D10 | 1947 | EFNB1 | NM_004429 | 177856 | 208728 | 0.78 | 0.95 | 3.43 | 1.04 |
| 48 | D11 | 1948 | EFNB2 | NM_004093 | 211903 | 217644 | 0.93 | 0.99 | -2.45 | -1.26 |
| 48 | D12 | 1950 | EGF | NM_001963 | 213088 | 217627 | 0.94 | 0.99 | -0.16 | 0.00 |
| 48 | E01 | NA | neg | NA | 230497 | 215867 | 1.01 | 0.99 | -3.12 | -1.26 |
| 48 | E02 | NA | neg | NA | 218563 | 227561 | 0.96 | 1.04 | -1.50 | -2.52 |
| 48 | E03 | 25975 | EGFL6 | NM_015507 | 205627 | 205172 | 0.90 | 0.94 | 0.90 | 0.00 |
| 48 | E04 | 2056 | EPO | NM_000799 | 217693 | 207462 | 0.96 | 0.95 | -0.22 | 0.01 |
| 48 | E05 | 58513 | EPS15L1 | NM_021235 | 199444 | 187210 | 0.88 | 0.86 | 1.56 | 1.34 |
| 48 | E06 | 2069 | EREG | NM_001432 | 203087 | 199755 | 0.89 | 0.91 | 0.61 | 0.53 |
| 48 | E07 | 11082 | ESM1 | NM_007036 | 217479 | 179921 | 0.96 | 0.82 | -1.00 | 3.38 |
| 48 | E08 | 8772 | FADD | NM_003824 | 213706 | 206605 | 0.94 | 0.94 | -0.83 | 0.01 |

| | | | | | | | | | | |
|---|---|---|---|---|---|---|---|---|---|---|
| 48 | E09 | 2204 | FCAR | NM_002000 | 185445 | 205209 | 0.82 | 0.94 | 1.86 | -1.09 |
| 48 | E10 | 2205 | FCER1A | NM_002001 | 202570 | 208538 | 0.89 | 0.95 | -0.57 | -0.53 |
| 48 | E11 | 2207 | FCER1G | NM_004106 | 192371 | 202431 | 0.85 | 0.93 | -0.44 | -1.22 |
| 48 | E12 | 2208 | FCER2 | NM_002002 | 205337 | 215105 | 0.90 | 0.98 | 0.25 | -1.32 |
| 48 | F01 | NA | NA | NA | 247820 | 204612 | 1.09 | 0.94 | -4.52 | 0.27 |
| 48 | F02 | NA | pos | NA | 87905 | 110646 | 0.39 | 0.51 | 17.21 | 10.37 |
| 48 | F03 | 2209 | FCGR1A | NM_000566 | 223405 | 207958 | 0.98 | 0.95 | -0.57 | 0.01 |
| 48 | F04 | 2212 | FCGR2A | NM_021642 | 224170 | 222261 | 0.99 | 1.02 | -0.15 | -1.27 |
| 48 | F05 | 2213 | FCGR2B | NM_004001 | 198687 | 195476 | 0.87 | 0.89 | 2.61 | 0.77 |
| 48 | F06 | 2215 | FCGR3B | NM_000570 | 223964 | 225907 | 0.98 | 1.03 | -1.28 | -1.96 |
| 48 | F07 | 2217 | FCGRT | NM_004107 | 216232 | 214105 | 0.95 | 0.98 | 0.12 | 0.02 |
| 48 | F08 | 10116 | FEM1B | NM_015322 | 203384 | 207115 | 0.89 | 0.95 | 1.52 | 0.27 |
| 48 | F09 | 2246 | FGF1 | NM_000800 | 204076 | 217167 | 0.90 | 0.99 | 0.28 | -2.05 |
| 48 | F10 | 2255 | FGF10 | NM_004465 | 207382 | 206697 | 0.91 | 0.94 | -0.28 | -0.02 |
| 48 | F11 | 2256 | FGF11 | NM_004112 | 203113 | 205207 | 0.89 | 0.94 | -0.95 | -1.20 |
| 48 | F12 | 2258 | FGF13 | NM_004114 | 193722 | 197556 | 0.85 | 0.90 | 2.78 | 0.89 |
| 48 | G01 | NA | neg | NA | 242858 | 217315 | 1.07 | 0.99 | -4.12 | -1.00 |
| 48 | G02 | NA | neg | NA | 217708 | 223171 | 0.96 | 1.02 | -0.70 | -1.63 |
| 48 | G03 | 2259 | FGF14 | NM_004115 | 214377 | 208457 | 0.94 | 0.95 | 0.39 | 0.06 |
| 48 | G04 | 8823 | FGF16 | NM_003868 | 208197 | 205768 | 0.92 | 0.94 | 1.74 | 0.61 |
| 48 | G05 | 8822 | FGF17 | NM_003867 | 212540 | 194119 | 0.93 | 0.89 | 0.46 | 1.02 |
| 48 | G06 | 8817 | FGF18 | NM_003862 | 214947 | 209165 | 0.95 | 0.96 | -0.33 | -0.06 |
| 48 | G07 | 9965 | FGF19 | NM_005117 | 224236 | 206528 | 0.99 | 0.94 | -1.24 | 0.94 |
| 48 | G08 | 26281 | FGF20 | NM_019851 | 225013 | 218624 | 0.99 | 1.00 | -1.69 | -0.87 |
| 48 | G09 | 26291 | FGF21 | NM_019113 | 218943 | 216670 | 0.96 | 0.99 | -2.01 | -1.90 |
| 48 | G10 | 8074 | FGF23 | NM_020638 | 210254 | 214779 | 0.92 | 0.98 | -0.94 | -0.78 |
| 48 | G11 | 2248 | FGF3 | NM_005247 | 188012 | 197291 | 0.83 | 0.90 | 0.83 | -0.25 |
| 48 | G12 | 2249 | FGF4 | NM_002007 | 201171 | 194713 | 0.88 | 0.89 | 1.49 | 1.29 |
| 48 | H01 | NA | NA | NA | 257052 | 222206 | 1.13 | 1.02 | -4.97 | 0.43 |
| 48 | H02 | NA | pos | NA | 94322 | 92399 | 0.41 | 0.42 | 17.14 | 14.39 |
| 48 | H03 | 2250 | FGF5 | NM_004464 | 216613 | 226636 | 0.95 | 1.04 | 1.16 | 0.06 |
| 48 | H04 | 2251 | FGF6 | NM_020996 | 236494 | 224430 | 1.04 | 1.03 | -1.02 | 0.55 |
| 48 | H05 | 2252 | FGF7 | NM_002009 | 227204 | 222962 | 1.00 | 1.02 | -0.46 | -0.13 |
| 48 | H06 | 2253 | FGF8 | NM_006119 | 213999 | 230750 | 0.94 | 1.05 | 0.88 | -0.43 |
| 48 | H07 | 2254 | FGF9 | NM_002010 | 215813 | 211722 | 0.95 | 0.97 | 0.98 | 2.33 |
| 48 | H08 | 2277 | FIGF | NM_004469 | 220204 | 229247 | 0.97 | 1.05 | 0.04 | -0.06 |
| 48 | H09 | 2316 | FLNA | NM_001456 | 212654 | 218064 | 0.94 | 1.00 | -0.08 | -0.10 |
| 48 | H10 | 2323 | FLT3LG | NM_001459 | 215316 | 226752 | 0.95 | 1.04 | -0.55 | -0.12 |
| 48 | H11 | 2348 | FOLR1 | NM_000802 | 198791 | 203663 | 0.87 | 0.93 | 0.44 | 1.02 |
| 48 | H12 | 2350 | FOLR2 | NM_000803 | 232716 | 211898 | 1.02 | 0.97 | -1.72 | 1.40 |
| 49 | A01 | NA | pos | NA | 70034 | 72444 | 0.30 | 0.31 | 18.86 | 16.93 |
| 49 | A02 | NA | NA | NA | 203180 | 217045 | 0.88 | 0.92 | -0.15 | -1.23 |
| 49 | A03 | 4856 | NOV | NM_002514 | 203753 | 218106 | 0.89 | 0.93 | 0.87 | -0.24 |
| 49 | A04 | 4878 | NPPA | NM_006172 | 200583 | 204934 | 0.87 | 0.87 | 0.50 | 0.97 |
| 49 | A05 | 7293 | TNFRSF4 | NM_003327 | 206815 | 213575 | 0.90 | 0.91 | 0.00 | 0.26 |
| 49 | A06 | 8743 | TNFSF10 | NM_003810 | 199448 | 209352 | 0.87 | 0.89 | 0.35 | 0.94 |
| 49 | A07 | 8600 | TNFSF11 | NM_003701 | 190648 | 201339 | 0.83 | 0.85 | 1.69 | 0.50 |
| 49 | A08 | 8742 | TNFSF12 | NM_153012 | 212359 | 201170 | 0.92 | 0.85 | -1.80 | 0.74 |
| 49 | A09 | 8741 | TNFSF13 | NM_003808 | 204678 | 216385 | 0.89 | 0.92 | -0.42 | -1.14 |
| 49 | A10 | 10673 | TNFSF13B | NM_006573 | 194977 | 210804 | 0.85 | 0.89 | 0.00 | -1.66 |
| 49 | A11 | 9966 | TNFSF15 | NM_005118 | 190074 | 202300 | 0.83 | 0.86 | -0.03 | -0.65 |
| 49 | A12 | 8995 | TNFSF18 | NM_005092 | 204334 | 216612 | 0.89 | 0.92 | -0.30 | -1.58 |
| 49 | B01 | NA | neg | NA | 270328 | 256691 | 1.18 | 1.09 | -8.02 | -5.13 |
| 49 | B02 | NA | neg | NA | 225093 | 230837 | 0.98 | 0.98 | -1.56 | -1.88 |
| 49 | B03 | 7292 | TNFSF4 | NM_003326 | 205535 | 219756 | 0.89 | 0.93 | 2.34 | 0.63 |
| 49 | B04 | 959 | TNFSF5 | NM_000074 | 217120 | 219869 | 0.95 | 0.93 | -0.14 | 0.18 |
| 49 | B05 | 356 | TNFSF6 | NM_000639 | 218937 | 232468 | 0.95 | 0.99 | 0.00 | -1.03 |
| 49 | B06 | 970 | TNFSF7 | NM_001252 | 221266 | 221081 | 0.96 | 0.94 | -1.04 | 0.55 |
| 49 | B07 | 944 | TNFSF8 | NM_001244 | 220143 | 219840 | 0.96 | 0.93 | -0.80 | -0.74 |
| 49 | B08 | 8744 | TNFSF9 | NM_003811 | 211780 | 216404 | 0.92 | 0.92 | 0.00 | -0.09 |
| 49 | B09 | 10140 | TOB1 | NM_005749 | 217181 | 217870 | 0.95 | 0.92 | -0.48 | -0.24 |
| 49 | B10 | 54472 | TOLLIP | NM_019009 | 185273 | 199460 | 0.81 | 0.85 | 3.11 | 0.85 |
| 49 | B11 | 9540 | TP53I3 | NM_004881 | 193531 | 200553 | 0.84 | 0.85 | 1.20 | 0.65 |
| 49 | B12 | 10131 | TRAP1 | NM_016292 | 213691 | 216125 | 0.93 | 0.92 | 0.09 | -0.43 |
| 49 | C01 | NA | pos | NA | 73052 | 107667 | 0.32 | 0.46 | 18.99 | 12.96 |
| 49 | C02 | NA | NA | NA | 232573 | 230002 | 1.01 | 0.98 | -3.79 | -2.40 |
| 49 | C03 | 7200 | TRH | NM_007117 | 221087 | 220365 | 0.96 | 0.93 | -1.05 | -0.07 |
| 49 | C04 | 7205 | TRIP6 | NM_003302 | 206038 | 208339 | 0.90 | 0.88 | 0.28 | 1.00 |
| 49 | C05 | 221037 | JMJD1C | NM_004241 | 186681 | 221803 | 0.81 | 0.94 | 3.44 | -0.32 |
| 49 | C06 | 7349 | UCN | NM_003353 | 194181 | 240411 | 0.85 | 1.02 | 1.67 | -2.51 |
| 49 | C07 | 7422 | VEGF | NM_003376 | 222784 | 208574 | 0.97 | 0.88 | -2.34 | 0.05 |
| 49 | C08 | 7423 | VEGFB | NM_003377 | 195005 | 206640 | 0.85 | 0.88 | 1.24 | 0.51 |
| 49 | C09 | 7424 | VEGFC | NM_005429 | 207635 | 201052 | 0.90 | 0.85 | -0.28 | 1.25 |
| 49 | C10 | 7425 | VGF | NM_003378 | 180590 | 199496 | 0.79 | 0.85 | 2.61 | 0.22 |
| 49 | C11 | 8838 | WISP3 | NM_003880 | 207542 | 215263 | 0.90 | 0.91 | -1.96 | -1.82 |
| 49 | C12 | 7476 | WNT7A | NM_004625 | 213363 | 215044 | 0.93 | 0.91 | -1.03 | -0.92 |
| 49 | D01 | NA | neg | NA | 246587 | 253593 | 1.07 | 1.08 | -4.78 | -4.37 |
| 49 | D02 | NA | neg | NA | 234223 | 236768 | 1.02 | 1.00 | -3.02 | -2.25 |
| 49 | D03 | 6375 | XCL1 | NM_002995 | 219460 | 227268 | 0.96 | 0.96 | 0.20 | 0.07 |
| 49 | D04 | 6846 | XCL2 | NM_003175 | 210992 | 225686 | 0.92 | 0.96 | 0.58 | -0.18 |
| 49 | D05 | 60401 | XEDAR | NM_021783 | 220140 | 219751 | 0.96 | 0.93 | -0.33 | 0.94 |
| 49 | D06 | 7784 | ZP3 | NM_007155 | 209362 | 229000 | 0.91 | 0.97 | 0.51 | -0.08 |
| 49 | D07 | 57007 | CMKOR1 | NM_020311 | 215998 | 203429 | 0.94 | 0.86 | -0.36 | 1.70 |
| 49 | D08 | 10060 | ABCC9 | NM_005691 | 210166 | 222965 | 0.92 | 0.95 | 0.08 | -0.54 |
| 49 | D09 | 66008 | ALS2CR3 | NM_015049 | 197075 | 224483 | 0.86 | 0.95 | 2.24 | -0.70 |
| 49 | D10 | 80833 | APOL3 | NM_145640 | 208006 | 215718 | 0.91 | 0.91 | -0.29 | -0.82 |
| 49 | D11 | 10776 | ARPP-19 | NM_006628 | 206543 | 203241 | 0.90 | 0.86 | -0.81 | 0.69 |
| 49 | D12 | 1134 | CHRNA1 | NM_000079 | 213856 | 213444 | 0.93 | 0.91 | -0.09 | 0.28 |

| | | | | | | | | | | |
|---|---|---|---|---|---|---|---|---|---|---|
| 49 | E01 | NA | neg | NA | 216122 | 234925 | 0.94 | 1.00 | -1.87 | -3.59 |
| 49 | E02 | NA | neg | NA | 216410 | 219944 | 0.94 | 0.93 | -1.91 | -1.71 |
| 49 | E03 | 57053 | CHRNA10 | NM_020402 | 212141 | 216178 | 0.92 | 0.92 | -0.20 | -0.11 |
| 49 | E04 | 1135 | CHRNA2 | NM_000742 | 226400 | 192692 | 0.99 | 0.82 | -3.06 | 2.39 |
| 49 | E05 | 1136 | CHRNA3 | NM_000743 | 182731 | 204329 | 0.80 | 0.87 | 3.58 | 1.30 |
| 49 | E06 | 1137 | CHRNA4 | NM_000744 | 199191 | 215297 | 0.87 | 0.91 | 0.52 | 0.08 |
| 49 | E07 | 1138 | CHRNA5 | NM_000745 | 204795 | 215940 | 0.89 | 0.92 | -0.20 | -1.44 |
| 49 | E08 | 8973 | CHRNA6 | NM_004198 | 217641 | 212761 | 0.95 | 0.90 | -2.42 | -0.83 |
| 49 | E09 | 1139 | CHRNA7 | NM_000746 | 189943 | 207433 | 0.83 | 0.88 | 1.82 | -0.13 |
| 49 | E10 | 55584 | CHRNA9 | NM_017581 | 194570 | 186569 | 0.85 | 0.79 | 0.19 | 1.27 |
| 49 | E11 | 1140 | CHRNB1 | NM_000747 | 189050 | 203711 | 0.82 | 0.86 | 0.25 | -0.94 |
| 49 | E12 | 1141 | CHRNB2 | NM_000748 | 205128 | 196700 | 0.89 | 0.83 | -0.28 | 0.81 |
| 49 | F01 | NA | NA | NA | 245223 | 232389 | 1.07 | 0.99 | -6.23 | -2.18 |
| 49 | F02 | NA | pos | NA | 79642 | 127131 | 0.35 | 0.54 | 17.42 | 11.04 |
| 49 | F03 | 1142 | CHRNB3 | NM_000749 | 224714 | 223092 | 0.98 | 0.95 | -2.20 | 0.12 |
| 49 | F04 | 1144 | CHRND | NM_000751 | 229121 | 242108 | 1.00 | 1.03 | -3.65 | -2.71 |
| 49 | F05 | 1145 | CHRNE | NM_000080 | 195621 | 216806 | 0.85 | 0.92 | 1.53 | 0.84 |
| 49 | F06 | 8029 | CUBN | NM_001081 | 221772 | 229255 | 0.97 | 0.97 | -2.91 | -0.58 |
| 49 | F07 | 2554 | GABRA1 | NM_000806 | 196172 | 211031 | 0.85 | 0.90 | 0.82 | 0.27 |
| 49 | F08 | 2555 | GABRA2 | NM_000807 | 213834 | 215558 | 0.93 | 0.91 | -2.09 | -0.08 |
| 49 | F09 | 2556 | GABRA3 | NM_000808 | 199269 | 188351 | 0.87 | 0.80 | 0.28 | 3.37 |
| 49 | F10 | 2557 | GABRA4 | NM_000809 | 194638 | 207199 | 0.85 | 0.88 | -0.03 | -0.22 |
| 49 | F11 | 2558 | GABRA5 | NM_000810 | 189127 | 192268 | 0.82 | 0.82 | 0.03 | 1.59 |
| 49 | F12 | 2559 | GABRA6 | NM_000811 | 198269 | 214121 | 0.86 | 0.91 | 0.49 | -0.28 |
| 49 | G01 | NA | neg | NA | 253737 | 236626 | 1.10 | 1.00 | -6.03 | -2.70 |
| 49 | G02 | NA | neg | NA | 216103 | 231208 | 0.94 | 0.98 | -0.65 | -2.02 |
| 49 | G03 | 2560 | GABRB1 | NM_000812 | 232942 | 229590 | 1.01 | 0.97 | -1.95 | -0.69 |
| 49 | G04 | 2561 | GABRB2 | NM_000813 | 212526 | 222773 | 0.93 | 0.94 | 0.14 | -0.27 |
| 49 | G05 | 2562 | GABRB3 | NM_000814 | 218686 | 225581 | 0.95 | 0.96 | -0.34 | -0.26 |
| 49 | G06 | 2563 | GABRD | NM_000815 | 231708 | 216679 | 1.01 | 0.92 | -2.90 | 1.01 |
| 49 | G07 | 2564 | GABRE | NM_004961 | 203275 | 213699 | 0.89 | 0.91 | 1.23 | -0.05 |
| 49 | G08 | 2565 | GABRG1 | NM_173536 | 209249 | 214315 | 0.91 | 0.91 | 0.00 | 0.08 |
| 49 | G09 | 2566 | GABRG2 | NM_198904 | 206976 | 211100 | 0.90 | 0.90 | 0.60 | 0.52 |
| 49 | G10 | 2567 | GABRG3 | NM_033223 | 204393 | 200910 | 0.89 | 0.85 | 0.00 | 0.58 |
| 49 | G11 | 2568 | GABRP | NM_014211 | 207738 | 216130 | 0.90 | 0.92 | -1.20 | -1.39 |
| 49 | G12 | 55879 | GABRQ | NM_018558 | 202282 | 204123 | 0.88 | 0.87 | 1.35 | 0.99 |
| 49 | H01 | NA | NA | NA | 245668 | 239369 | 1.07 | 1.02 | -3.41 | -2.05 |
| 49 | H02 | NA | pos | NA | 110566 | 95397 | 0.48 | 0.40 | 15.89 | 16.03 |
| 49 | H03 | 2569 | GABRR1 | NM_002042 | 223686 | 207926 | 0.97 | 0.88 | 0.83 | 3.03 |
| 49 | H04 | 2570 | GABRR2 | NM_002043 | 252765 | 252741 | 1.10 | 1.07 | -4.15 | -3.04 |
| 49 | H05 | 2741 | GLRA1 | NM_000171 | 233606 | 245230 | 1.02 | 1.04 | -1.01 | -1.73 |
| 49 | H06 | 2742 | GLRA2 | NM_002063 | 224097 | 245111 | 0.98 | 1.04 | -0.35 | -1.57 |
| 49 | H07 | 8001 | GLRA3 | NM_006529 | 220732 | 222636 | 0.96 | 0.94 | 0.20 | -0.18 |
| 49 | H08 | 2902 | GRIN1 | NM_000832 | 214478 | 216032 | 0.93 | 0.92 | 0.71 | 0.86 |
| 49 | H09 | 2903 | GRIN2A | NM_000833 | 224474 | 222134 | 0.98 | 0.94 | -0.43 | 0.13 |
| 49 | H10 | 2904 | GRIN2B | NM_000834 | 216087 | 218038 | 0.94 | 0.92 | -0.21 | -0.58 |
| 49 | H11 | 2906 | GRIN2D | NM_000836 | 196892 | 198081 | 0.86 | 0.84 | 1.81 | 1.87 |
| 49 | H12 | 170572 | HTR3C | NM_130770 | 205018 | 207912 | 0.89 | 0.88 | 2.42 | 1.50 |
| 50 | A01 | NA | pos | NA | 74598 | 72221 | 0.32 | 0.32 | 22.32 | 25.84 |
| 50 | A02 | NA | NA | NA | 228022 | 206016 | 0.96 | 0.91 | -1.10 | -0.42 |
| 50 | A03 | 11018 | IL1RL1LG | NM_006858 | 201369 | 210632 | 0.85 | 0.93 | 2.86 | -0.14 |
| 50 | A04 | 11014 | KDELR2 | NM_006854 | 207717 | 209876 | 0.88 | 0.93 | 0.51 | -1.14 |
| 50 | A05 | 3949 | LDLR | NM_000527 | 220190 | 214717 | 0.93 | 0.95 | -1.24 | -0.41 |
| 50 | A06 | 4035 | LRP1 | NM_002332 | 189355 | 195310 | 0.80 | 0.86 | 4.60 | 1.75 |
| 50 | A07 | 5023 | P2RX1 | NM_002558 | 220929 | 203147 | 0.93 | 0.90 | -0.66 | 0.08 |
| 50 | A08 | 22953 | P2RX2 | NM_012226 | 202979 | 196078 | 0.86 | 0.87 | 3.72 | 0.45 |
| 50 | A09 | 5024 | P2RX3 | NM_002559 | 232182 | 210979 | 0.98 | 0.93 | -0.54 | -2.70 |
| 50 | A10 | 5025 | P2RX4 | NM_002560 | 233061 | 196471 | 0.98 | 0.87 | -1.77 | 0.00 |
| 50 | A11 | 5027 | P2RX7 | NM_002562 | 225234 | 181854 | 0.95 | 0.80 | 0.16 | 0.59 |
| 50 | A12 | 5191 | PEX7 | NM_000288 | 231488 | 204745 | 0.98 | 0.90 | -0.19 | 0.00 |
| 50 | B01 | NA | neg | NA | 238170 | 257425 | 1.01 | 1.14 | -4.27 | -7.29 |
| 50 | B02 | NA | neg | NA | 221417 | 219155 | 0.94 | 0.97 | -1.72 | 0.23 |
| 50 | B03 | 5830 | PEX5 | NM_000319 | 209690 | 219958 | 0.89 | 0.97 | -0.03 | 1.26 |
| 50 | B04 | 6262 | RYR2 | NM_001035 | 200595 | 221408 | 0.85 | 0.98 | -0.02 | -0.18 |
| 50 | B05 | 6263 | RYR3 | NM_001036 | 213095 | 226658 | 0.90 | 1.00 | -1.78 | 0.47 |
| 50 | B06 | 114815 | SORCS1 | NM_052918 | 210705 | 221221 | 0.89 | 0.98 | -0.28 | -0.11 |
| 50 | B07 | 57537 | SORCS2 | NM_020777 | 205372 | 226696 | 0.87 | 1.00 | 0.09 | -1.31 |
| 50 | B08 | 22986 | SORCS3 | NM_014978 | 216563 | 215398 | 0.91 | 0.95 | 0.02 | -0.12 |
| 50 | B09 | 9326 | TRIP3 | NM_004773 | 216673 | 214142 | 0.92 | 0.94 | 0.21 | -0.10 |
| 50 | B10 | 56302 | TRPV5 | NM_019841 | 213551 | 201800 | 0.90 | 0.89 | -0.41 | 2.18 |
| 50 | B11 | 55503 | TRPV6 | NM_018646 | 212365 | 201062 | 0.90 | 0.89 | 0.51 | 0.05 |
| 50 | B12 | 7436 | VLDLR | NM_003383 | 211861 | 201058 | 0.89 | 0.89 | 1.18 | 3.95 |
| 50 | C01 | NA | pos | NA | 80276 | 86476 | 0.34 | 0.38 | 19.68 | 24.60 |
| 50 | C02 | NA | NA | NA | 224565 | 223622 | 0.95 | 0.99 | -2.34 | -2.32 |
| 50 | C03 | 939 | TNFRSF7 | NM_001242 | 208275 | 222312 | 0.88 | 0.98 | 0.03 | -0.88 |
| 50 | C04 | 2099 | ESR1 | NM_000125 | 202790 | 193101 | 0.86 | 0.85 | -0.51 | 3.70 |
| 50 | C05 | 196 | AHR | NM_001621 | 193683 | 211406 | 0.82 | 0.93 | 1.04 | 1.79 |
| 50 | C06 | 9049 | AIP | NM_003977 | 196619 | 189599 | 0.83 | 0.84 | 1.72 | 4.43 |
| 50 | C07 | 367 | AR | NM_000044 | 227670 | 226563 | 0.96 | 1.00 | -3.46 | -2.96 |
| 50 | C08 | 405 | ARNT | NM_001668 | 215870 | 205674 | 0.91 | 0.91 | -0.02 | 0.12 |
| 50 | C09 | 9915 | ARNT2 | NM_014862 | 223436 | 206049 | 0.94 | 0.91 | -0.97 | -0.18 |
| 50 | C10 | 406 | ARNTL | NM_001178 | 219703 | 219683 | 0.93 | 0.97 | -1.49 | -3.00 |
| 50 | C11 | 56938 | ARNTL2 | NM_020183 | 214138 | 190365 | 0.90 | 0.84 | 0.09 | 0.47 |
| 50 | C12 | 941 | CD80 | NM_005191 | 206701 | 215358 | 0.87 | 0.95 | 1.82 | -0.53 |
| 50 | D01 | NA | neg | NA | 268276 | 236144 | 1.13 | 1.04 | -9.73 | -4.56 |
| 50 | D02 | NA | neg | NA | 260099 | 222683 | 1.10 | 0.98 | -8.48 | -1.91 |
| 50 | D03 | 942 | CD86 | NM_006889 | 216904 | 226838 | 0.92 | 1.00 | -2.00 | -1.54 |
| 50 | D04 | 9575 | CLOCK | NM_004898 | 195393 | 211201 | 0.83 | 0.93 | -0.09 | 0.38 |

| | | | | | | | | | | |
|---|---|---|---|---|---|---|---|---|---|---|
| 50 | D05 | 29883 | CNOT7 | NM_013354 | 197763 | 218439 | 0.84 | 0.96 | -0.30 | 0.64 |
| 50 | D06 | 133396 | IL31RA | NM_139017 | 206641 | 202473 | 0.87 | 0.89 | -0.52 | 2.13 |
| 50 | D07 | 9282 | CRSP2 | NM_004229 | 200966 | 210003 | 0.85 | 0.93 | -0.09 | 0.52 |
| 50 | D08 | 9440 | CRSP6 | NM_004268 | 194928 | 213168 | 0.82 | 0.94 | 2.46 | -1.13 |
| 50 | D09 | 9441 | CRSP7 | NM_004831 | 212016 | 212135 | 0.90 | 0.94 | 0.06 | -1.15 |
| 50 | D10 | 9443 | CRSP9 | NM_004270 | 201795 | 201466 | 0.85 | 0.89 | 0.53 | 0.80 |
| 50 | D11 | 2034 | EPAS1 | NM_001430 | 182773 | 196060 | 0.77 | 0.86 | 4.16 | -0.42 |
| 50 | D12 | 2078 | ERG | NM_004449 | 212732 | 219949 | 0.90 | 0.97 | 0.19 | -1.20 |
| 50 | E01 | NA | neg | NA | 239107 | 225813 | 1.01 | 1.00 | -4.42 | -3.50 |
| 50 | E02 | NA | neg | NA | 213793 | 210658 | 0.90 | 0.93 | -0.56 | -0.53 |
| 50 | E03 | 2100 | ESR2 | NM_001437 | 215744 | 213297 | 0.91 | 0.94 | -0.96 | 0.14 |
| 50 | E04 | 2101 | ESRRA | NM_004451 | 207727 | 188012 | 0.88 | 0.83 | -1.12 | 3.95 |
| 50 | E05 | 2103 | ESRRB | NM_004452 | 198763 | 216624 | 0.84 | 0.96 | 0.40 | 0.02 |
| 50 | E06 | 2104 | ESRRG | NM_001438 | 199000 | 231030 | 0.84 | 1.02 | 1.50 | -4.45 |
| 50 | E07 | 3091 | HIF1A | NM_001530 | 204108 | 209651 | 0.86 | 0.92 | 0.28 | -0.39 |
| 50 | E08 | 64344 | HIF3A | NM_022462 | 206657 | 186969 | 0.87 | 0.82 | 1.53 | 3.04 |
| 50 | E09 | 3159 | HMGA1 | NM_002131 | 226366 | 196853 | 0.96 | 0.87 | -1.27 | 0.87 |
| 50 | E10 | 3174 | HNF4G | NM_004133 | 202363 | 207659 | 0.85 | 0.92 | 1.29 | -1.39 |
| 50 | E11 | 9859 | KAB | NM_014812 | 217574 | 189188 | 0.92 | 0.83 | -0.30 | -0.05 |
| 50 | E12 | 8648 | NCOA1 | NM_003743 | 233355 | 213968 | 0.99 | 0.94 | -2.10 | -1.00 |
| 50 | F01 | NA | NA | NA | 263703 | 235390 | 1.11 | 1.04 | -8.08 | -3.58 |
| 50 | F02 | NA | pos | NA | 71890 | 116648 | 0.30 | 0.51 | 21.21 | 19.73 |
| 50 | F03 | 10499 | NCOA2 | NM_006540 | 212814 | 229577 | 0.90 | 1.01 | -0.42 | -1.25 |
| 50 | F04 | 23054 | NCOA6 | NM_014071 | 198429 | 220201 | 0.84 | 0.97 | 0.40 | -0.56 |
| 50 | F05 | 4793 | NFKBIB | NM_001001716 | 221375 | 228863 | 0.93 | 1.01 | -2.95 | -0.58 |
| 50 | F06 | 4851 | NOTCH1 | NM_017617 | 211275 | 227928 | 0.89 | 1.01 | -0.27 | -2.04 |
| 50 | F07 | 4853 | NOTCH2 | NM_024408 | 205016 | 200810 | 0.87 | 0.89 | 0.24 | 3.15 |
| 50 | F08 | 4854 | NOTCH3 | NM_000435 | 227309 | 201673 | 0.96 | 0.89 | -1.53 | 1.96 |
| 50 | F09 | 4855 | NOTCH4 | NM_004557 | 214775 | 184049 | 0.91 | 0.81 | 0.59 | 5.19 |
| 50 | F10 | 4861 | NPAS1 | NM_002517 | 208830 | 209563 | 0.88 | 0.92 | 0.41 | 0.03 |
| 50 | F11 | 190 | NR0B1 | NM_000475 | 230481 | 198423 | 0.97 | 0.88 | -2.17 | -0.06 |
| 50 | F12 | 8431 | NR0B2 | NM_021969 | 212447 | 206515 | 0.90 | 0.91 | 1.19 | 2.26 |
| 50 | G01 | NA | neg | NA | 231368 | 228580 | 0.98 | 1.01 | -1.97 | -3.72 |
| 50 | G02 | NA | neg | NA | 235368 | 227522 | 0.99 | 1.00 | -2.58 | -3.52 |
| 50 | G03 | 9572 | NR1D1 | NM_021724 | 212081 | 213885 | 0.90 | 0.94 | 0.87 | 0.35 |
| 50 | G04 | 9975 | NR1D2 | NM_005126 | 208601 | 208882 | 0.88 | 0.92 | 0.02 | 0.18 |
| 50 | G05 | 7376 | NR1H2 | NM_007121 | 205589 | 218460 | 0.87 | 0.96 | 0.64 | -0.02 |
| 50 | G06 | 10062 | NR1H3 | NM_005693 | 217545 | 219582 | 0.92 | 0.97 | -0.06 | -1.88 |
| 50 | G07 | 9971 | NR1H4 | NM_005123 | 202050 | 209732 | 0.85 | 0.93 | 1.87 | -0.08 |
| 50 | G08 | 8856 | NR1I2 | NM_003889 | 235319 | 207115 | 0.99 | 0.91 | -1.57 | -0.59 |
| 50 | G09 | 9970 | NR1I3 | NM_005122 | 226772 | 202439 | 0.96 | 0.89 | -0.06 | 0.10 |
| 50 | G10 | 7181 | NR2C1 | NM_003297 | 215746 | 202194 | 0.91 | 0.89 | 0.53 | 0.00 |
| 50 | G11 | 7182 | NR2C2 | NM_003298 | 229891 | 186762 | 0.97 | 0.82 | -0.90 | 0.75 |
| 50 | G12 | 7101 | NR2E1 | NM_003269 | 233768 | 210496 | 0.99 | 0.93 | -0.89 | 0.00 |
| 50 | H01 | NA | NA | NA | 240428 | 239831 | 1.02 | 1.06 | -5.53 | -4.32 |
| 50 | H02 | NA | pos | NA | 75321 | 116914 | 0.32 | 0.52 | 19.68 | 19.81 |
| 50 | H03 | 10002 | NR2E3 | NM_014249 | 199375 | 217211 | 0.84 | 0.96 | 0.63 | 1.31 |
| 50 | H04 | 7025 | NR2F1 | NM_005654 | 190041 | 241253 | 0.80 | 1.06 | 0.68 | -4.56 |
| 50 | H05 | 7026 | NR2F2 | NM_021005 | 193544 | 245145 | 0.82 | 1.08 | 0.30 | -3.64 |
| 50 | H06 | 2063 | NR2F6 | NM_005234 | 202562 | 217676 | 0.86 | 0.96 | 0.06 | 0.11 |
| 50 | H07 | 2908 | NR3C1 | NM_000176 | 201466 | 215855 | 0.85 | 0.95 | -0.22 | 0.33 |
| 50 | H08 | 4306 | NR3C2 | NM_000901 | 216749 | 230336 | 0.92 | 1.02 | -0.92 | -3.53 |
| 50 | H09 | 3164 | NR4A1 | NM_002135 | 194877 | 207723 | 0.82 | 0.92 | 2.63 | 0.68 |
| 50 | H10 | 4929 | NR4A2 | NM_006186 | 222488 | 213281 | 0.94 | 0.94 | -2.68 | -0.56 |
| 50 | H11 | 8013 | NR4A3 | NM_006981 | 210296 | 199674 | 0.89 | 0.88 | -0.09 | -0.17 |
| 50 | H12 | 2516 | NR5A1 | NM_004959 | 232321 | 198953 | 0.98 | 0.88 | -2.85 | 3.88 |
| 51 | A01 | NA | pos | NA | 52679 | 34987 | 0.23 | 0.16 | 19.16 | 23.47 |
| 51 | A02 | NA | NA | NA | 256934 | 277167 | 1.11 | 1.27 | -1.79 | -3.00 |
| 51 | A03 | 2494 | NR5A2 | NM_003822 | 233608 | 261085 | 1.01 | 1.20 | 1.79 | -1.03 |
| 51 | A04 | 2649 | NR6A1 | NM_001489 | 257238 | NA | 1.11 | NA | -0.71 | NA |
| 51 | A05 | 7849 | PAX8 | NM_003466 | 251784 | 249269 | 1.09 | 1.15 | -0.47 | 1.00 |
| 51 | A06 | 133522 | PPARGC1B | NM_133263 | 273967 | 287380 | 1.18 | 1.32 | -2.73 | -2.56 |
| 51 | A07 | 5241 | PGR | NM_000926 | 233061 | 286702 | 1.01 | 1.32 | -0.02 | -3.27 |
| 51 | A08 | 5356 | PLRG1 | NM_002669 | 227549 | 230785 | 0.98 | 1.06 | 0.33 | 1.86 |
| 51 | A09 | 5465 | PPARA | NM_005036 | 227064 | 244225 | 0.98 | 1.12 | 0.78 | 0.02 |
| 51 | A10 | 5469 | PPARBP | NM_004774 | 220769 | 226980 | 0.95 | 1.04 | 0.02 | 1.91 |
| 51 | A11 | 5467 | PPARD | NM_006238 | 225486 | 248038 | 0.98 | 1.14 | -0.65 | -0.63 |
| 51 | A12 | 5468 | PPARG | NM_005037 | 229553 | 259549 | 0.99 | 1.19 | 1.52 | 0.00 |
| 51 | B01 | NA | neg | NA | 257344 | 201803 | 1.11 | 0.93 | -3.88 | 1.47 |
| 51 | B02 | NA | neg | NA | 254650 | 221722 | 1.10 | 1.02 | -3.61 | -0.71 |
| 51 | B03 | 5914 | RARA | NM_000964 | 188866 | 178855 | 0.82 | 0.82 | 4.33 | 4.19 |
| 51 | B04 | 5915 | RARB | NM_000965 | 214802 | 214783 | 0.93 | 0.99 | 1.59 | -0.22 |
| 51 | B05 | 5916 | RARG | NM_000966 | 222384 | 203519 | 0.96 | 0.94 | 0.50 | 2.23 |
| 51 | B06 | 5966 | REL | NM_002908 | 227166 | 205170 | 0.98 | 0.94 | 0.02 | 2.65 |
| 51 | B07 | 5970 | RELA | NM_021975 | 213634 | 220293 | 0.92 | 1.01 | -0.08 | 0.21 |
| 51 | B08 | 6095 | RORA | NM_002943 | 190108 | 221744 | 0.82 | 1.02 | 2.12 | -0.92 |
| 51 | B09 | 6096 | RORB | NM_006914 | 224268 | 228346 | 0.97 | 1.05 | -0.98 | -2.01 |
| 51 | B10 | 6097 | RORC | NM_005060 | 222103 | 224558 | 0.96 | 1.03 | -2.17 | -1.60 |
| 51 | B11 | 6256 | RXRA | NM_002957 | 201007 | 209851 | 0.87 | 0.96 | -0.19 | -0.22 |
| 51 | B12 | 6257 | RXRB | NM_021976 | 224538 | 208384 | 0.97 | 0.96 | -0.02 | 1.82 |
| 51 | C01 | NA | pos | NA | 70030 | 52932 | 0.30 | 0.24 | 12.96 | 17.62 |
| 51 | C02 | NA | NA | NA | 179217 | 186867 | 0.78 | 0.86 | 1.76 | 2.98 |
| 51 | C03 | 6258 | RXRG | NM_006917 | 199631 | 213439 | 0.86 | 0.98 | 0.86 | 0.29 |
| 51 | C04 | 4902 | NRTN | NM_004558 | 187171 | 235342 | 0.81 | 1.08 | 2.05 | -2.58 |
| 51 | C05 | 4879 | NPPB | NM_002521 | 204057 | 224543 | 0.88 | 1.03 | 0.01 | -0.18 |
| 51 | C06 | 29982 | NRBF2 | NM_030759 | 198527 | 254325 | 0.86 | 1.17 | 0.58 | -2.84 |
| 51 | C07 | 4908 | NTF3 | NM_002527 | 194666 | 207746 | 0.84 | 0.95 | -0.50 | 1.47 |
| 51 | C08 | 4909 | NTF5 | NM_006179 | 175517 | 197535 | 0.76 | 0.91 | 1.24 | 1.60 |

| | | | | | | | | | | |
|---|---|---|---|---|---|---|---|---|---|---|
| 51 | C09 | 4922 | NTS | NM_006183 | 205186 | 199773 | 0.89 | 0.92 | -1.39 | 0.99 |
| 51 | C10 | 11054 | OGFR | NM_007346 | 177918 | 230266 | 0.77 | 1.06 | -0.01 | -2.34 |
| 51 | C11 | 4973 | OLR1 | NM_002543 | 228741 | 216983 | 0.99 | 1.00 | -5.40 | -1.12 |
| 51 | C12 | 4978 | OPCML | NM_002545 | 222630 | 222301 | 0.96 | 1.02 | -2.19 | 0.18 |
| 51 | D01 | NA | neg | NA | 278991 | 218844 | 1.21 | 1.01 | -5.95 | 0.19 |
| 51 | D02 | NA | neg | NA | 228249 | 224415 | 0.99 | 1.03 | -0.75 | -0.42 |
| 51 | D03 | 5008 | OSM | NM_020530 | 234009 | 225695 | 1.01 | 1.04 | -0.15 | -0.35 |
| 51 | D04 | 5154 | PDGFA | NM_002607 | 225320 | 214070 | 0.97 | 0.98 | 0.66 | 0.44 |
| 51 | D05 | 5155 | PDGFB | NM_002608 | 220040 | 252551 | 0.95 | 1.16 | 0.89 | -2.55 |
| 51 | D06 | 5179 | PENK | NM_006211 | 228919 | 233219 | 0.99 | 1.07 | -0.02 | 0.17 |
| 51 | D07 | 5196 | PF4 | NM_002619 | 187833 | 235380 | 0.81 | 1.08 | 2.72 | -0.85 |
| 51 | D08 | 5228 | PGF | NM_002632 | 237614 | 213475 | 1.03 | 0.98 | -2.61 | 0.56 |
| 51 | D09 | 10424 | PGRMC2 | NM_006320 | 232528 | 241750 | 1.01 | 1.11 | -1.68 | -2.90 |
| 51 | D10 | 55023 | PHIP | NM_017934 | 202295 | 200855 | 0.87 | 0.92 | 0.01 | 1.57 |
| 51 | D11 | 5364 | PLXNB1 | NM_002673 | 193045 | 188018 | 0.83 | 0.86 | 0.77 | 2.74 |
| 51 | D12 | 10154 | PLXNC1 | NM_005761 | 227318 | 231914 | 0.98 | 1.07 | -0.15 | -0.17 |
| 51 | E01 | NA | neg | NA | 225628 | 194086 | 0.98 | 0.89 | -0.92 | 2.72 |
| 51 | E02 | NA | neg | NA | 220819 | 216378 | 0.95 | 0.99 | -0.42 | 0.28 |
| 51 | E03 | 5367 | PMCH | NM_002674 | 226839 | 229047 | 0.98 | 1.05 | 0.15 | -0.89 |
| 51 | E04 | 5443 | POMC | NM_000939 | 240676 | 216038 | 1.04 | 0.99 | -1.35 | 0.05 |
| 51 | E05 | 5617 | PRL | NM_000948 | 203791 | 214044 | 0.88 | 0.98 | 2.12 | 1.49 |
| 51 | E06 | 84432 | PROK1 | NM_032414 | 235413 | 231135 | 1.02 | 1.06 | -1.12 | 0.22 |
| 51 | E07 | 5623 | PSPN | NM_004158 | 199164 | 228979 | 0.86 | 1.05 | 1.12 | -0.33 |
| 51 | E08 | 5744 | PTHLH | NM_002820 | 225266 | 225131 | 0.97 | 1.03 | -1.77 | -0.89 |
| 51 | E09 | 5818 | PVRL1 | NM_002855 | 197699 | 214057 | 0.86 | 0.98 | 1.46 | -0.05 |
| 51 | E10 | 11331 | REA | NM_007273 | 171630 | 173124 | 0.74 | 0.80 | 2.72 | 4.42 |
| 51 | E11 | 6013 | RLN1 | NM_006911 | 197788 | 214174 | 0.86 | 0.98 | -0.15 | -0.29 |
| 51 | E12 | 6019 | RLN2 | NM_005059 | 227493 | 223620 | 0.98 | 1.03 | -0.60 | 0.56 |
| 51 | F01 | NA | NA | NA | 225021 | 160471 | 0.97 | 0.74 | -1.79 | 4.90 |
| 51 | F02 | NA | pos | NA | 80126 | 146740 | 0.35 | 0.67 | 13.06 | 6.40 |
| 51 | F03 | 6277 | S100A6 | NM_014624 | 231973 | 209993 | 1.00 | 0.96 | -1.32 | -0.29 |
| 51 | F04 | 6280 | S100A9 | NM_002965 | 229624 | 211920 | 0.99 | 0.97 | -1.16 | -0.99 |
| 51 | F05 | 6320 | SCGF | NM_002975 | 216220 | 212368 | 0.94 | 0.98 | -0.10 | 0.18 |
| 51 | F06 | 9255 | SCYE1 | NM_004757 | 219319 | 219469 | 0.95 | 1.01 | -0.41 | 0.01 |
| 51 | F07 | 6404 | SELPLG | NM_003006 | 199904 | 201756 | 0.86 | 0.93 | 0.10 | 1.16 |
| 51 | F08 | 6504 | SLAMF1 | NM_003037 | 202014 | 206712 | 0.87 | 0.95 | -0.33 | -0.36 |
| 51 | F09 | 8835 | SOCS2 | NM_003877 | 196359 | 196201 | 0.85 | 0.90 | 0.65 | 0.42 |
| 51 | F10 | 6693 | SPN | NM_003123 | 180390 | 202745 | 0.78 | 0.93 | 0.88 | -0.30 |
| 51 | F11 | 6734 | SRPR | NM_003139 | 183560 | 182977 | 0.79 | 0.84 | 0.37 | 1.63 |
| 51 | F12 | 29967 | LRP12 | NM_013437 | 209587 | 215241 | 0.91 | 0.99 | 0.29 | -0.01 |
| 51 | G01 | NA | neg | NA | 234203 | 188689 | 1.01 | 0.87 | -2.83 | 2.11 |
| 51 | G02 | NA | neg | NA | 220292 | 224673 | 0.95 | 1.03 | -1.41 | -1.82 |
| 51 | G03 | 6863 | TAC1 | NM_003182 | 222743 | 202390 | 0.96 | 0.93 | -0.47 | 0.83 |
| 51 | G04 | 6866 | TAC3 | NM_013251 | 223763 | 205491 | 0.97 | 0.94 | -0.66 | 0.01 |
| 51 | G05 | 6997 | TDGF1 | NM_003212 | 216081 | 223057 | 0.93 | 1.03 | -0.18 | -0.69 |
| 51 | G06 | 7038 | TG | NM_003235 | 212617 | 222312 | 0.92 | 1.02 | 0.18 | -0.01 |
| 51 | G07 | 7040 | TGFB1 | NM_000660 | 221122 | 217007 | 0.96 | 1.00 | -2.17 | -0.21 |
| 51 | G08 | 7043 | TGFB3 | NM_003239 | 189135 | 203568 | 0.82 | 0.94 | 0.89 | 0.28 |
| 51 | G09 | 7045 | TGFBI | NM_000358 | 191880 | 181210 | 0.83 | 0.83 | 1.01 | 2.35 |
| 51 | G10 | 7049 | TGFBR3 | NM_003243 | 198541 | 205080 | 0.86 | 0.94 | -1.08 | -0.26 |
| 51 | G11 | 7056 | THBD | NM_000361 | 182064 | 198553 | 0.79 | 0.91 | 0.43 | 0.22 |
| 51 | G12 | 7097 | TLR2 | NM_003264 | 201072 | 219527 | 0.87 | 1.01 | 1.07 | -0.18 |
| 51 | H01 | NA | NA | NA | 211869 | 129160 | 0.92 | 0.59 | 0.20 | 10.93 |
| 51 | H02 | NA | pos | NA | 80793 | 145116 | 0.35 | 0.67 | 13.64 | 9.18 |
| 51 | H03 | 51311 | TLR8 | NM_016610 | 246368 | 226631 | 1.07 | 1.04 | -2.15 | 0.49 |
| 51 | H04 | 7124 | TNF | NM_000594 | 217470 | 226679 | 0.94 | 1.04 | 0.73 | 0.00 |
| 51 | H05 | 4982 | TNFRSF11B | NM_002546 | 221620 | 246445 | 0.96 | 1.13 | -0.01 | -0.94 |
| 51 | H06 | 51330 | TNFRSF12A | NM_016639 | 213828 | 264999 | 0.92 | 1.22 | 0.80 | -2.36 |
| 51 | H07 | 8764 | TNFRSF14 | NM_003820 | 206990 | 231205 | 0.90 | 1.06 | 0.02 | 0.54 |
| 51 | H08 | 8784 | TNFRSF18 | NM_004195 | 221532 | 229733 | 0.96 | 1.06 | -1.69 | -0.28 |
| 51 | H09 | 55504 | TNFRSF19 | NM_018647 | 215360 | 224059 | 0.93 | 1.03 | -0.65 | -0.02 |
| 51 | H10 | 7132 | TNFRSF1A | NM_001065 | 210679 | 221463 | 0.91 | 1.02 | -1.58 | 0.26 |
| 51 | H11 | 7133 | TNFRSF1B | NM_001066 | 192024 | 202586 | 0.83 | 0.93 | 0.15 | 2.09 |
| 51 | H12 | 8718 | TNFRSF25 | NM_003790 | 218543 | 238965 | 0.95 | 1.10 | 0.02 | 0.00 |
| 52 | A01 | NA | pos | NA | 54196 | 46425 | 0.22 | 0.19 | 23.26 | 22.68 |
| 52 | A02 | NA | NA | NA | 227178 | 234906 | 0.93 | 0.98 | 0.77 | 0.76 |
| 52 | A03 | 6492 | SIM1 | NM_005068 | 255664 | 236655 | 1.04 | 0.99 | -1.65 | 1.67 |
| 52 | A04 | 6493 | SIM2 | NM_005069 | 232980 | 277751 | 0.95 | 1.16 | 0.54 | -3.08 |
| 52 | A05 | 6772 | STAT1 | NM_007315 | 253854 | 252296 | 1.03 | 1.06 | -0.07 | -1.04 |
| 52 | A06 | 6773 | STAT2 | NM_005419 | 238061 | 228796 | 0.97 | 0.96 | -0.55 | 1.45 |
| 52 | A07 | 6775 | STAT4 | NM_003151 | 247304 | 248656 | 1.01 | 1.04 | -2.01 | -1.70 |
| 52 | A08 | 6776 | STAT5A | NM_003152 | 237766 | 254908 | 0.97 | 1.07 | -0.71 | -1.84 |
| 52 | A09 | 6777 | STAT5B | NM_012448 | 214242 | 224761 | 0.87 | 0.94 | 2.06 | 0.21 |
| 52 | A10 | 6778 | STAT6 | NM_003153 | 213927 | 242134 | 0.87 | 1.01 | 1.70 | -0.06 |
| 52 | A11 | 7041 | TGFB1I1 | NM_015927 | 215497 | 232160 | 0.88 | 0.97 | 0.07 | 0.10 |
| 52 | A12 | 7067 | THRA | NM_003250 | 208611 | 222889 | 0.85 | 0.93 | 3.90 | 2.31 |
| 52 | B01 | NA | neg | NA | 323824 | 257938 | 1.32 | 1.08 | -15.90 | -6.07 |
| 52 | B02 | NA | neg | NA | 218315 | 225825 | 0.89 | 0.95 | -2.18 | -2.33 |
| 52 | B03 | 7068 | THRB | NM_000461 | 213859 | 189993 | 0.87 | 0.80 | -0.31 | 2.95 |
| 52 | B04 | 9968 | TNRC11 | NM_005120 | 191353 | 221662 | 0.78 | 0.93 | 1.85 | -0.70 |
| 52 | B05 | 9967 | THRAP3 | NM_005119 | 212784 | 203435 | 0.87 | 0.85 | 1.18 | 0.50 |
| 52 | B06 | 9969 | THRAP1 | NM_005121 | 200277 | 204587 | 0.82 | 0.86 | 0.27 | 0.12 |
| 52 | B07 | 10025 | THRAP5 | NM_005481 | 201482 | 205900 | 0.82 | 0.86 | -0.15 | -0.88 |
| 52 | B08 | 9319 | TRIP13 | NM_004237 | 202385 | 218736 | 0.82 | 0.92 | -0.21 | -1.77 |
| 52 | B09 | 9325 | TRIP4 | NM_016213 | 197430 | 191223 | 0.80 | 0.80 | 0.15 | -0.03 |
| 52 | B10 | 51567 | TTRAP | NM_016614 | 212662 | 205392 | 0.87 | 0.86 | -2.24 | 0.06 |
| 52 | B11 | 7421 | VDR | NM_000376 | 224243 | 226101 | 0.91 | 0.95 | -5.17 | -3.35 |
| 52 | B12 | 29079 | VDRIP | NM_014166 | 204920 | 202053 | 0.83 | 0.85 | 0.29 | 0.59 |

| | | | | | | | | | | |
|---|---|---|---|---|---|---|---|---|---|---|
| 52 | C01 | NA | pos | NA | 58765 | 67618 | 0.24 | 0.28 | 17.88 | 17.34 |
| 52 | C02 | NA | NA | NA | 175091 | 184603 | 0.71 | 0.77 | 2.75 | 3.73 |
| 52 | C03 | 393 | ARHGAP4 | NM_001666 | 198908 | 207955 | 0.81 | 0.87 | 0.94 | 2.13 |
| 52 | C04 | 395 | ARHGAP6 | NM_001174 | 198883 | 203433 | 0.81 | 0.85 | 0.19 | 2.69 |
| 52 | C05 | 9138 | ARHGEF1 | NM_004706 | 198332 | 202520 | 0.81 | 0.85 | 2.37 | 1.88 |
| 52 | C06 | 1894 | ECT2 | NM_018098 | 197068 | 216155 | 0.80 | 0.91 | 0.00 | 0.04 |
| 52 | C07 | 2057 | EPOR | NM_000121 | 195047 | 183029 | 0.79 | 0.77 | 0.00 | 3.05 |
| 52 | C08 | 667 | BPAG1 | NM_001723 | 187119 | 215110 | 0.76 | 0.90 | 1.09 | -0.08 |
| 52 | C09 | 1499 | CTNNB1 | NM_001904 | 211863 | 227421 | 0.86 | 0.95 | -2.42 | -2.97 |
| 52 | C10 | 182 | JAG1 | NM_000214 | 202833 | 223685 | 0.83 | 0.94 | -1.65 | -0.80 |
| 52 | C11 | 91179 | SCARF2 | NM_153334 | 186030 | 214259 | 0.76 | 0.90 | -0.88 | -0.70 |
| 52 | C12 | 9353 | SLIT2 | NM_004787 | 230691 | 238093 | 0.94 | 1.00 | -3.75 | -2.33 |
| 52 | D01 | NA | neg | NA | 285029 | 245326 | 1.16 | 1.03 | -9.42 | -4.47 |
| 52 | D02 | NA | neg | NA | 264420 | 231964 | 1.08 | 0.97 | -6.74 | -2.91 |
| 52 | D03 | 9516 | LITAF | NM_004862 | 215253 | 223835 | 0.88 | 0.94 | 0.94 | -0.85 |
| 52 | D04 | 4090 | SMAD5 | NM_005903 | 229406 | 210050 | 0.93 | 0.88 | -1.67 | 0.78 |
| 52 | D05 | 10001 | MED6 | NM_005466 | 203989 | 206617 | 0.83 | 0.87 | 3.75 | 0.26 |
| 52 | D06 | 27229 | 76P | NM_014444 | 236302 | 207077 | 0.96 | 0.87 | -2.99 | -0.04 |
| 52 | D07 | 10881 | ACTL7A | NM_006687 | 175104 | 182050 | 0.71 | 0.76 | 4.71 | 2.03 |
| 52 | D08 | 10880 | ACTL7B | NM_006686 | 231810 | 222435 | 0.94 | 0.93 | -2.61 | -2.07 |
| 52 | D09 | 265 | AMELX | NM_001142 | 227262 | 191836 | 0.93 | 0.80 | -2.30 | 0.03 |
| 52 | D10 | 287 | ANK2 | NM_001148 | 206446 | 209031 | 0.84 | 0.88 | 0.00 | -0.23 |
| 52 | D11 | 288 | ANK3 | NM_001149 | 192378 | 181404 | 0.78 | 0.76 | 0.41 | 1.99 |
| 52 | D12 | 10095 | ARPC1B | NM_005720 | 218128 | 216396 | 0.89 | 0.91 | 0.00 | -0.95 |
| 52 | E01 | NA | neg | NA | 254081 | 269303 | 1.03 | 1.13 | -5.44 | -6.60 |
| 52 | E02 | NA | neg | NA | 228493 | 227403 | 0.93 | 0.95 | -2.12 | -1.73 |
| 52 | E03 | 10109 | ARPC2 | NM_005731 | 202811 | 224688 | 0.83 | 0.94 | 2.51 | -0.30 |
| 52 | E04 | 10094 | ARPC3 | NM_005719 | 218032 | 232557 | 0.89 | 0.97 | -0.23 | -1.18 |
| 52 | E05 | 633 | BGN | NM_001711 | 235510 | 237615 | 0.96 | 1.00 | -0.39 | -2.69 |
| 52 | E06 | 857 | CAV1 | NM_001753 | 212998 | 210802 | 0.87 | 0.88 | 0.00 | 0.18 |
| 52 | E07 | 9076 | CLDN1 | NM_021101 | 190132 | 196108 | 0.77 | 0.82 | 2.71 | 1.05 |
| 52 | E08 | 9071 | CLDN10 | NM_006984 | 210343 | 208089 | 0.86 | 0.87 | 0.14 | 0.25 |
| 52 | E09 | 5010 | CLDN11 | NM_005602 | 209907 | 194453 | 0.85 | 0.81 | -0.09 | 0.38 |
| 52 | E10 | 9069 | CLDN12 | NM_012129 | 206126 | 187028 | 0.84 | 0.78 | 0.00 | 2.99 |
| 52 | E11 | 23562 | CLDN14 | NM_012130 | 177515 | 205747 | 0.72 | 0.86 | 2.30 | -0.19 |
| 52 | E12 | 9075 | CLDN2 | NM_020384 | 225253 | 220572 | 0.92 | 0.92 | -0.97 | -0.78 |
| 52 | F01 | NA | NA | NA | 218226 | 223727 | 0.89 | 0.94 | -2.15 | -2.19 |
| 52 | F02 | NA | pos | NA | 65991 | 126377 | 0.27 | 0.53 | 17.64 | 9.13 |
| 52 | F03 | 7122 | CLDN5 | NM_003277 | 209177 | 211903 | 0.85 | 0.89 | 0.31 | 0.30 |
| 52 | F04 | 1300 | COL10A1 | NM_000493 | 202354 | 212279 | 0.82 | 0.89 | 0.44 | 0.29 |
| 52 | F05 | 1302 | COL11A2 | NM_080679 | 221928 | 204037 | 0.90 | 0.85 | 0.00 | 0.33 |
| 52 | F06 | 1303 | COL12A1 | NM_004370 | 200386 | 211735 | 0.82 | 0.89 | 0.27 | -0.82 |
| 52 | F07 | 1307 | COL16A1 | NM_001856 | 200461 | 194516 | 0.82 | 0.82 | 0.00 | 0.34 |
| 52 | F08 | 1308 | COL17A1 | NM_000494 | 200926 | 201869 | 0.82 | 0.85 | -0.01 | 0.08 |
| 52 | F09 | 1277 | COL1A1 | NM_000088 | 203560 | 200116 | 0.83 | 0.84 | -0.64 | -1.17 |
| 52 | F10 | 1278 | COL1A2 | NM_000089 | 194157 | 221114 | 0.79 | 0.93 | 0.18 | -1.87 |
| 52 | F11 | 1281 | COL3A1 | NM_000090 | 185152 | 197279 | 0.75 | 0.83 | -0.07 | -0.10 |
| 52 | F12 | 1282 | COL4A1 | NM_001845 | 208363 | 207565 | 0.85 | 0.87 | -0.15 | -0.16 |
| 52 | G01 | NA | neg | NA | 236655 | 254892 | 0.96 | 1.07 | -4.55 | -5.72 |
| 52 | G02 | NA | neg | NA | 237056 | 219848 | 0.97 | 0.92 | -4.61 | -1.64 |
| 52 | G03 | 1284 | COL4A2 | NM_001846 | 218728 | 222336 | 0.89 | 0.93 | -0.94 | -0.82 |
| 52 | G04 | 1286 | COL4A4 | NM_000092 | 207094 | 218104 | 0.84 | 0.91 | -0.19 | -0.29 |
| 52 | G05 | 1287 | COL4A5 | NM_000495 | NA | 213166 | NA | 0.89 | NA | -0.63 |
| 52 | G06 | 1290 | COL5A2 | NM_000393 | 197075 | 206773 | 0.80 | 0.87 | 0.70 | -0.14 |
| 52 | G07 | 50509 | COL5A3 | NM_015719 | 195286 | 212945 | 0.80 | 0.89 | 0.67 | -1.70 |
| 52 | G08 | 1297 | COL9A1 | NM_001851 | 194067 | 195528 | 0.79 | 0.82 | 0.88 | 0.92 |
| 52 | G09 | 1298 | COL9A2 | NM_001852 | 195017 | 185555 | 0.79 | 0.78 | 0.47 | 0.63 |
| 52 | G10 | 1299 | COL9A3 | NM_001853 | 199734 | 185487 | 0.81 | 0.78 | -0.55 | 2.38 |
| 52 | G11 | 1409 | CRYAA | NM_000394 | 190871 | 195726 | 0.78 | 0.82 | -0.82 | 0.18 |
| 52 | G12 | 1410 | CRYAB | NM_001885 | 207202 | 205755 | 0.84 | 0.86 | 0.00 | 0.16 |
| 52 | H01 | NA | NA | NA | 214702 | 223374 | 0.87 | 0.94 | 0.41 | 0.58 |
| 52 | H02 | NA | pos | NA | 69630 | 85433 | 0.28 | 0.36 | 19.27 | 16.62 |
| 52 | H03 | 1411 | CRYBA1 | NM_005208 | 260380 | 244573 | 1.06 | 1.02 | -4.24 | -0.77 |
| 52 | H04 | 1412 | CRYBA2 | NM_005209 | 228709 | 235133 | 0.93 | 0.99 | -0.89 | 0.36 |
| 52 | H05 | 1417 | CRYBB3 | NM_004076 | 247470 | 232555 | 1.01 | 0.97 | -1.22 | -0.26 |
| 52 | H06 | 1419 | CRYGB | NM_005210 | 290711 | 233030 | 1.18 | 0.98 | -9.37 | -0.57 |
| 52 | H07 | 1420 | CRYGC | NM_020989 | 216693 | 223877 | 0.88 | 0.94 | -0.01 | -0.34 |
| 52 | H08 | 8030 | CCDC6 | NM_005436 | 217006 | 216945 | 0.88 | 0.91 | 0.01 | 1.06 |
| 52 | H09 | 7818 | DAP3 | NM_004632 | 214152 | 251680 | 0.87 | 1.05 | 0.09 | -4.44 |
| 52 | H10 | 11258 | DCTN3 | NM_007234 | 205922 | 215367 | 0.84 | 0.90 | 0.76 | 1.53 |
| 52 | H11 | 1832 | DSP | NM_004415 | 195716 | 207444 | 0.80 | 0.87 | 0.66 | 1.45 |
| 52 | H12 | 1893 | ECM1 | NM_004425 | 214991 | 212323 | 0.88 | 0.89 | 1.10 | 2.02 |
| 53 | A01 | NA | pos | NA | 43422 | 59523 | 0.19 | 0.24 | 21.10 | 20.87 |
| 53 | A02 | NA | NA | NA | 219023 | 258876 | 0.95 | 1.06 | -0.39 | -5.51 |
| 53 | A03 | 2006 | ELN | NM_000501 | 220455 | 197916 | 0.95 | 0.81 | 0.43 | 4.06 |
| 53 | A04 | 10117 | ENAM | NM_031889 | 234364 | 242569 | 1.01 | 1.00 | -2.44 | -2.11 |
| 53 | A05 | 2125 | EVPL | NM_001988 | 203474 | 229092 | 0.88 | 0.94 | 2.14 | -0.70 |
| 53 | A06 | 2312 | FLG | XM_048104 | 216637 | 221247 | 0.94 | 0.91 | 0.93 | -0.76 |
| 53 | A07 | 1647 | GADD45A | NM_001924 | 240917 | 218059 | 1.04 | 0.90 | -3.84 | -0.07 |
| 53 | A08 | 10912 | GADD45G | NM_006705 | 225580 | 215203 | 0.97 | 0.88 | -1.05 | -0.45 |
| 53 | A09 | 2670 | GFAP | NM_002055 | 202864 | 204186 | 0.88 | 0.84 | 1.34 | 1.00 |
| 53 | A10 | 3339 | HSPG2 | NM_005529 | 216251 | 213185 | 0.93 | 0.88 | -0.84 | 0.07 |
| 53 | A11 | 51350 | HUMCYT2A | NM_015848 | 202511 | 204875 | 0.87 | 0.84 | -0.04 | 1.59 |
| 53 | A12 | 9119 | K6HF | NM_004693 | 226234 | 208490 | 0.98 | 0.86 | 0.06 | 1.21 |
| 53 | B01 | NA | neg | NA | 231824 | 259792 | 1.00 | 1.07 | -3.00 | -6.63 |
| 53 | B02 | NA | neg | NA | 231398 | 238882 | 1.00 | 0.98 | -2.94 | -3.86 |
| 53 | B03 | 319101 | K6IRS3 | NM_175068 | 207269 | 222267 | 0.89 | 0.91 | 1.00 | -0.16 |
| 53 | B04 | 3858 | KRT10 | NM_000421 | 206426 | 220898 | 0.89 | 0.91 | -0.06 | -0.23 |

| | | | | | | | | | | |
|---|---|---|---|---|---|---|---|---|---|---|
| 53 | B05 | 3859 | KRT12 | NM_000223 | 210150 | 216310 | 0.91 | 0.89 | 0.28 | 0.00 |
| 53 | B06 | 3860 | KRT13 | NM_002274 | 227093 | 199893 | 0.98 | 0.82 | -1.39 | 1.07 |
| 53 | B07 | 3861 | KRT14 | NM_000526 | 216159 | 205958 | 0.93 | 0.85 | -1.85 | 0.54 |
| 53 | B08 | 3866 | KRT15 | NM_002275 | 212960 | 212384 | 0.92 | 0.87 | -0.55 | -1.07 |
| 53 | B09 | 3880 | KRT19 | NM_002276 | 205056 | 208774 | 0.89 | 0.86 | 0.03 | -0.60 |
| 53 | B10 | 54474 | KRT20 | NM_019010 | 198927 | 197847 | 0.86 | 0.81 | 0.23 | 1.11 |
| 53 | B11 | 3849 | KRT2A | NM_000423 | 180040 | 187435 | 0.78 | 0.77 | 1.66 | 2.91 |
| 53 | B12 | 3850 | KRT3 | NM_057088 | 218605 | 210150 | 0.94 | 0.86 | -0.06 | 0.00 |
| 53 | C01 | NA | pos | NA | 53897 | 62068 | 0.23 | 0.26 | 18.55 | 20.77 |
| 53 | C02 | NA | NA | NA | 166623 | NA | 0.72 | NA | 4.75 | NA |
| 53 | C03 | 3851 | KRT4 | NM_002272 | 183933 | 195518 | 0.79 | 0.80 | 3.63 | 4.62 |
| 53 | C04 | 3852 | KRT5 | NM_000424 | 199242 | 210185 | 0.86 | 0.86 | 0.59 | 2.42 |
| 53 | C05 | 3853 | KRT6A | NM_005554 | 210014 | 209760 | 0.91 | 0.86 | 0.07 | 2.10 |
| 53 | C06 | 3854 | KRT6B | NM_005555 | 196750 | 221094 | 0.85 | 0.91 | 2.09 | -0.50 |
| 53 | C07 | 140446 | KRT6C | NM_058242 | 208648 | 218810 | 0.90 | 0.90 | -1.16 | 0.07 |
| 53 | C08 | 3855 | KRT7 | NM_005556 | 234942 | 214179 | 1.01 | 0.88 | -3.46 | -0.07 |
| 53 | C09 | 3856 | KRT8 | NM_002273 | 203694 | 233081 | 0.88 | 0.96 | -0.03 | -2.58 |
| 53 | C10 | 3886 | KRTHA5 | NM_002280 | 208643 | 225334 | 0.90 | 0.93 | -1.18 | -1.29 |
| 53 | C11 | 8689 | KRTHA6 | NM_003771 | 191401 | 219977 | 0.83 | 0.90 | 0.04 | -0.17 |
| 53 | C12 | 3981 | LIG4 | NM_002312 | 222367 | 213528 | 0.96 | 0.88 | -0.74 | 0.79 |
| 53 | D01 | NA | neg | NA | 280407 | 258869 | 1.21 | 1.06 | -8.03 | -6.00 |
| 53 | D02 | NA | neg | NA | 221577 | 209079 | 0.96 | 0.86 | -0.83 | 0.59 |
| 53 | D03 | 84823 | LMNB2 | NM_032737 | 225276 | 228402 | 0.97 | 0.94 | -0.29 | -0.47 |
| 53 | D04 | 4014 | LOR | NM_000427 | 212869 | 222931 | 0.92 | 0.92 | 0.06 | 0.00 |
| 53 | D05 | 4130 | MAP1A | NM_002373 | 224059 | 211977 | 0.97 | 0.87 | -0.51 | 1.08 |
| 53 | D06 | 4131 | MAP1B | NM_005909 | 209111 | 205699 | 0.90 | 0.85 | 1.73 | 0.81 |
| 53 | D07 | 9053 | MAP7 | NM_003980 | 201057 | 226325 | 0.87 | 0.93 | 0.91 | -1.65 |
| 53 | D08 | 4137 | MAPT | NM_005910 | 211642 | 195191 | 0.91 | 0.80 | 0.53 | 1.71 |
| 53 | D09 | 4148 | MATN3 | NM_002381 | 214438 | 217236 | 0.93 | 0.89 | -0.20 | -1.22 |
| 53 | D10 | 4155 | MBP | NM_002385 | 209356 | 206009 | 0.90 | 0.85 | -0.13 | 0.53 |
| 53 | D11 | 4284 | MIP | NM_012064 | 197411 | 214325 | 0.85 | 0.88 | 0.45 | -0.15 |
| 53 | D12 | 4359 | MPZ | NM_000530 | 228670 | 213948 | 0.99 | 0.88 | -0.37 | 0.00 |
| 53 | E01 | NA | neg | NA | 269221 | 267435 | 1.16 | 1.10 | -8.34 | -8.89 |
| 53 | E02 | NA | neg | NA | 215529 | 198656 | 0.93 | 0.82 | -1.77 | 0.21 |
| 53 | E03 | 28998 | MRPL13 | NM_014078 | 214555 | 210393 | 0.93 | 0.87 | -0.66 | 0.16 |
| 53 | E04 | 10573 | MRPL28 | NM_006428 | 196712 | 189731 | 0.85 | 0.78 | 0.35 | 2.63 |
| 53 | E05 | 64983 | MRPL32 | NM_031903 | 228687 | 215290 | 0.99 | 0.89 | -2.76 | -1.12 |
| 53 | E06 | 64963 | MRPS11 | NM_022839 | 243804 | 192323 | 1.05 | 0.79 | -4.21 | 0.82 |
| 53 | E07 | 9423 | NTN1 | NM_004822 | 179794 | 195739 | 0.78 | 0.80 | 1.83 | 0.63 |
| 53 | E08 | 4917 | NTN2L | NM_006181 | 181912 | 196001 | 0.79 | 0.81 | 2.48 | -0.16 |
| 53 | E09 | 59277 | NTN4 | NM_021229 | 178613 | 185705 | 0.77 | 0.76 | 2.50 | 1.20 |
| 53 | E10 | 4926 | NUMA1 | NM_006185 | 177020 | 207397 | 0.76 | 0.85 | 2.14 | -1.41 |
| 53 | E11 | 5411 | PNN | NM_002687 | 190623 | 215764 | 0.82 | 0.89 | -0.40 | -2.10 |
| 53 | E12 | 8557 | TCAP | NM_003673 | 215005 | 205528 | 0.93 | 0.85 | -0.39 | -0.64 |
| 53 | F01 | NA | NA | NA | 205509 | 239867 | 0.89 | 0.99 | -0.84 | -4.45 |
| 53 | F02 | NA | pos | NA | 67984 | 132703 | 0.29 | 0.55 | 15.99 | 9.73 |
| 53 | F03 | 7007 | TECTA | NM_005422 | 205742 | 220026 | 0.89 | 0.90 | 0.12 | -0.32 |
| 53 | F04 | 7311 | UBA52 | NM_003333 | 121713 | NA | 0.53 | NA | 9.24 | NA |
| 53 | F05 | 7399 | USH2A | NM_007123 | 204290 | 197056 | 0.88 | 0.81 | -0.07 | 2.09 |
| 53 | F06 | 7414 | VCL | NM_003373 | 203007 | 200805 | 0.88 | 0.83 | 0.49 | 0.50 |
| 53 | F07 | 87 | ACTN1 | NM_001102 | 184931 | 212056 | 0.80 | 0.87 | 0.90 | -0.73 |
| 53 | F08 | 88 | ACTN2 | NM_001103 | 204151 | 194610 | 0.88 | 0.80 | -0.53 | 0.82 |
| 53 | F09 | 89 | ACTN3 | NM_001104 | 200178 | 181880 | 0.86 | 0.75 | -0.44 | 2.50 |
| 53 | F10 | 81 | ACTN4 | NM_004924 | 196111 | 202778 | 0.85 | 0.83 | -0.49 | 0.00 |
| 53 | F11 | 118 | ADD1 | NM_001119 | 185553 | 217592 | 0.80 | 0.89 | -0.08 | -1.54 |
| 53 | F12 | 120 | ADD3 | NM_016824 | 207030 | 213718 | 0.89 | 0.88 | 0.30 | -0.93 |
| 53 | G01 | NA | neg | NA | 238074 | 247565 | 1.03 | 1.02 | -4.65 | -5.70 |
| 53 | G02 | NA | neg | NA | 213127 | 213375 | 0.92 | 0.88 | -1.60 | -1.17 |
| 53 | G03 | 57180 | ARP3BETA | NM_020445 | 209174 | 211233 | 0.90 | 0.87 | -0.12 | 0.62 |
| 53 | G04 | 421 | ARVCF | NM_001670 | 211568 | 212010 | 0.91 | 0.87 | -1.58 | 0.26 |
| 53 | G05 | 79937 | CNTNAP3 | NM_033655 | 203391 | 211138 | 0.88 | 0.87 | 0.22 | 0.00 |
| 53 | G06 | 989 | CDC10 | NM_001788 | 212459 | 218327 | 0.92 | 0.90 | -0.49 | -2.05 |
| 53 | G07 | 26047 | CNTNAP2 | NM_014141 | 192484 | 220252 | 0.83 | 0.91 | 0.15 | -2.04 |
| 53 | G08 | 1301 | COL11A1 | NM_001854 | 195023 | 197724 | 0.84 | 0.81 | 0.76 | 0.18 |
| 53 | G09 | 7373 | COL14A1 | XM_044622 | 212792 | 211155 | 0.92 | 0.87 | -1.81 | -1.60 |
| 53 | G10 | 80781 | COL18A1 | NM_030582 | 180962 | 201036 | 0.78 | 0.83 | 1.54 | 0.00 |
| 53 | G11 | 1310 | COL19A1 | NM_001858 | 194640 | 203082 | 0.84 | 0.83 | -1.01 | 0.15 |
| 53 | G12 | 1288 | COL4A6 | NM_001847 | 207384 | 213452 | 0.90 | 0.88 | 0.43 | -1.12 |
| 53 | H01 | NA | NA | NA | 215272 | 210456 | 0.93 | 0.87 | 1.99 | 2.11 |
| 53 | H02 | NA | pos | NA | 68023 | 106506 | 0.29 | 0.44 | 20.01 | 15.87 |
| 53 | H03 | 1289 | COL5A1 | NM_000093 | 273395 | 268700 | 1.18 | 1.10 | -4.13 | -4.09 |
| 53 | H04 | 1291 | COL6A1 | NM_001848 | 235759 | 284465 | 1.02 | 1.17 | -0.69 | -6.43 |
| 53 | H05 | 1292 | COL6A2 | NM_001849 | 242068 | 233592 | 1.05 | 0.96 | -0.66 | -0.07 |
| 53 | H06 | 1296 | COL8A2 | NM_005202 | 259234 | 229269 | 1.12 | 0.94 | -2.36 | -0.60 |
| 53 | H07 | 1311 | COMP | NM_000095 | 226477 | 224004 | 0.98 | 0.92 | -0.15 | 0.36 |
| 53 | H08 | 11151 | CORO1A | NM_007074 | 214670 | 220455 | 0.93 | 0.91 | 2.21 | 0.07 |
| 53 | H09 | 23418 | CRB1 | NM_012076 | 207024 | 216411 | 0.89 | 0.89 | 2.75 | 0.60 |
| 53 | H10 | 1496 | CTNNA2 | NM_004389 | 224013 | 224764 | 0.97 | 0.92 | 0.13 | -0.24 |
| 53 | H11 | 1501 | CTNND2 | NM_001332 | 209644 | 211480 | 0.91 | 0.87 | 1.00 | 1.94 |
| 53 | H12 | 1756 | DMD | NM_000109 | 224764 | 222591 | 0.97 | 0.92 | 2.15 | 0.57 |
| 54 | A01 | NA | pos | NA | 44382 | 54884 | 0.18 | 0.24 | 16.13 | 15.88 |
| 54 | A02 | NA | NA | NA | 222037 | 214732 | 0.91 | 0.92 | 0.02 | 0.31 |
| 54 | A03 | 1821 | DRP2 | NM_001939 | 288653 | 259498 | 1.18 | 1.11 | -4.66 | -2.29 |
| 54 | A04 | 2035 | EPB41 | NM_004437 | 257557 | 231789 | 1.05 | 0.99 | -1.94 | -0.13 |
| 54 | A05 | 2036 | EPB41L1 | NM_012156 | 226327 | 233528 | 0.93 | 1.00 | -0.76 | -1.81 |
| 54 | A06 | 2038 | EPB42 | NM_000119 | 220664 | 227970 | 0.90 | 0.98 | 0.38 | -0.98 |
| 54 | A07 | 2195 | FAT | NM_005245 | 241763 | 220014 | 0.99 | 0.94 | -1.82 | 0.13 |
| 54 | A08 | 2196 | FAT2 | NM_001447 | 215626 | 220624 | 0.88 | 0.95 | -0.18 | -0.15 |

| | | | | | | | | | | |
|---|---|---|---|---|---|---|---|---|---|---|
| 54 | A09 | 2197 | FAU | NM_001997 | 122843 | 116396 | 0.50 | 0.50 | 9.04 | 9.33 |
| 54 | A10 | 2192 | FBLN1 | NM_001996 | 205895 | 207497 | 0.84 | 0.89 | 1.25 | 0.45 |
| 54 | A11 | 2200 | FBN1 | NM_000138 | 214748 | 204537 | 0.88 | 0.88 | 0.14 | 0.50 |
| 54 | A12 | 2201 | FBN2 | NM_001999 | 221626 | 206277 | 0.91 | 0.88 | 0.34 | 1.17 |
| 54 | B01 | NA | neg | NA | 257563 | 232993 | 1.05 | 1.00 | -5.11 | -3.24 |
| 54 | B02 | NA | neg | NA | 224749 | 212306 | 0.92 | 0.91 | -2.14 | -1.22 |
| 54 | B03 | 2335 | FN1 | NM_002026 | 195058 | 199819 | 0.80 | 0.86 | 1.92 | 1.76 |
| 54 | B04 | 284217 | LAMA1 | NM_005559 | 190817 | 202175 | 0.78 | 0.87 | 2.20 | 0.99 |
| 54 | B05 | 3909 | LAMA3 | NM_000227 | 196003 | 192118 | 0.80 | 0.82 | 0.08 | 0.46 |
| 54 | B06 | 3912 | LAMB1 | NM_002291 | 195749 | 202733 | 0.80 | 0.87 | 0.74 | -0.28 |
| 54 | B07 | 3913 | LAMB2 | NM_002292 | 202393 | 204619 | 0.83 | 0.88 | -0.15 | -0.13 |
| 54 | B08 | 3914 | LAMB3 | NM_000228 | 196618 | 193045 | 0.80 | 0.83 | -0.37 | 0.78 |
| 54 | B09 | 3915 | LAMC1 | NM_002293 | 202831 | 210594 | 0.83 | 0.90 | -0.13 | -1.62 |
| 54 | B10 | 3918 | LAMC2 | NM_005562 | 214082 | 208406 | 0.88 | 0.89 | -1.40 | -1.40 |
| 54 | B11 | 10319 | LAMC3 | NM_006059 | 196733 | 209556 | 0.81 | 0.90 | -0.14 | -1.76 |
| 54 | B12 | 3996 | LLGL1 | NM_004140 | 196106 | 198275 | 0.80 | 0.85 | 0.75 | 0.19 |
| 54 | C01 | NA | pos | NA | 52717 | 66165 | 0.22 | 0.28 | 13.04 | 12.65 |
| 54 | C02 | NA | NA | NA | 168515 | 180298 | 0.69 | 0.77 | 2.54 | 1.53 |
| 54 | C03 | 4000 | LMNA | NM_170707 | 207512 | 213451 | 0.85 | 0.92 | 0.36 | 0.07 |
| 54 | C04 | 4060 | LUM | NM_002345 | 201776 | 205392 | 0.83 | 0.88 | 0.78 | 0.31 |
| 54 | C05 | 4133 | MAP2 | NM_002374 | 192977 | 227286 | 0.79 | 0.97 | -0.08 | -3.34 |
| 54 | C06 | 4146 | MATN1 | NM_002379 | 199190 | 188167 | 0.82 | 0.81 | -0.01 | 0.77 |
| 54 | C07 | 9782 | MATR3 | NM_018834 | 198235 | 176737 | 0.81 | 0.76 | -0.20 | 2.22 |
| 54 | C08 | 4256 | MGP | NM_000900 | 161684 | 200538 | 0.66 | 0.86 | 2.37 | -0.32 |
| 54 | C09 | 65008 | MRPL1 | NM_020236 | 196624 | 191665 | 0.80 | 0.82 | 0.01 | -0.14 |
| 54 | C10 | 4607 | MYBPC3 | NM_000256 | 218288 | 184564 | 0.89 | 0.79 | -2.21 | 0.55 |
| 54 | C11 | 4635 | MYL4 | NM_002476 | 206471 | 189519 | 0.85 | 0.81 | -1.45 | -0.17 |
| 54 | C12 | 4703 | NEB | NM_004543 | 199511 | 210169 | 0.82 | 0.90 | 0.01 | -1.34 |
| 54 | D01 | NA | neg | NA | 272957 | 253292 | 1.12 | 1.09 | -6.97 | -4.84 |
| 54 | D02 | NA | neg | NA | 243077 | 233443 | 0.99 | 1.00 | -4.26 | -2.91 |
| 54 | D03 | 4744 | NEFH | NM_021076 | 225638 | 227809 | 0.92 | 0.98 | -1.32 | -0.59 |
| 54 | D04 | 4771 | NF2 | NM_000268 | 199718 | 214799 | 0.82 | 0.92 | 0.93 | 0.13 |
| 54 | D05 | 4809 | NHP2L1 | NM_005008 | 135293 | 142782 | 0.55 | 0.61 | 5.12 | 5.64 |
| 54 | D06 | 4811 | NID | NM_002508 | 209330 | 194831 | 0.86 | 0.84 | -0.96 | 0.86 |
| 54 | D07 | 4867 | NPHP1 | NM_000272 | 192087 | 228746 | 0.79 | 0.98 | 0.32 | -2.11 |
| 54 | D08 | 261734 | NPHP4 | NM_015102 | 186146 | 203242 | 0.76 | 0.87 | 0.12 | 0.16 |
| 54 | D09 | 5317 | PKP1 | NM_000299 | 198766 | 213675 | 0.81 | 0.92 | -0.22 | -1.54 |
| 54 | D10 | 5318 | PKP2 | NM_004572 | 184951 | 222755 | 0.76 | 0.96 | 0.78 | -2.43 |
| 54 | D11 | 11187 | PKP3 | NM_007183 | 202665 | 176300 | 0.83 | 0.76 | -1.14 | 1.86 |
| 54 | D12 | 8502 | PKP4 | NM_003628 | 200718 | 204806 | 0.82 | 0.88 | -0.13 | -0.07 |
| 54 | E01 | NA | neg | NA | 252437 | 236256 | 1.03 | 1.01 | -6.41 | -5.07 |
| 54 | E02 | NA | neg | NA | 207187 | 215864 | 0.85 | 0.93 | -2.30 | -3.08 |
| 54 | E03 | 5339 | PLEC1 | NM_000445 | 216736 | 217471 | 0.89 | 0.93 | -1.81 | -1.47 |
| 54 | E04 | 5962 | RDX | NM_002906 | 222066 | 214672 | 0.91 | 0.92 | -2.39 | -1.74 |
| 54 | E05 | 6187 | RPS2 | NM_002952 | 156300 | 159509 | 0.64 | 0.68 | 1.92 | 2.12 |
| 54 | E06 | 6227 | RPS21 | NM_001024 | 153454 | 181356 | 0.63 | 0.78 | 2.81 | 0.28 |
| 54 | E07 | 6188 | RPS3 | NM_001005 | 134664 | 122789 | 0.55 | 0.53 | 4.23 | 6.33 |
| 54 | E08 | 6193 | RPS5 | NM_001009 | 126781 | 139083 | 0.52 | 0.60 | 4.21 | 4.52 |
| 54 | E09 | 10174 | SCAM-1 | NM_005775 | 186297 | 177021 | 0.76 | 0.76 | -0.39 | 0.14 |
| 54 | E10 | 6586 | SLIT3 | NM_003062 | 201208 | 216017 | 0.82 | 0.93 | -2.00 | -3.66 |
| 54 | E11 | 6525 | SMTN | NM_006932 | 171977 | 177848 | 0.70 | 0.76 | 0.35 | -0.18 |
| 54 | E12 | 6710 | SPTB | NM_000347 | 206054 | 214010 | 0.84 | 0.92 | -1.91 | -2.86 |
| 54 | F01 | NA | NA | NA | 218835 | 225788 | 0.90 | 0.97 | -2.06 | -2.52 |
| 54 | F02 | NA | pos | NA | 55761 | 99244 | 0.23 | 0.43 | 12.73 | 9.81 |
| 54 | F03 | 6779 | STATH | NM_003154 | 211038 | 218770 | 0.86 | 0.94 | 0.01 | -0.07 |
| 54 | F04 | 7058 | THBS2 | NM_003247 | 220584 | 213931 | 0.90 | 0.92 | -0.96 | -0.14 |
| 54 | F05 | 7060 | THBS4 | NM_003248 | 189523 | 201686 | 0.78 | 0.86 | 0.20 | -0.46 |
| 54 | F06 | 7168 | TPM1 | NM_000366 | 207157 | 196541 | 0.85 | 0.84 | -0.76 | 0.34 |
| 54 | F07 | 7169 | TPM2 | NM_003289 | 191011 | 196337 | 0.78 | 0.84 | 0.42 | 0.69 |
| 54 | F08 | 7171 | TPM4 | NM_003290 | 188806 | 213783 | 0.77 | 0.92 | -0.12 | -1.23 |
| 54 | F09 | 9499 | TTID | NM_006790 | 185258 | 186225 | 0.76 | 0.80 | 1.01 | 0.77 |
| 54 | F10 | 10426 | TUBGCP3 | NM_006322 | 190482 | 177724 | 0.78 | 0.76 | 0.28 | 1.61 |
| 54 | F11 | 7402 | UTRN | NM_007124 | 196278 | 202457 | 0.80 | 0.87 | -0.56 | -1.05 |
| 54 | F12 | 7431 | VIM | NM_003380 | 199419 | 199631 | 0.82 | 0.86 | -0.01 | 0.07 |
| 54 | G01 | NA | neg | NA | 245526 | 246958 | 1.01 | 1.06 | -4.77 | -4.69 |
| 54 | G02 | NA | neg | NA | 238401 | 210907 | 0.98 | 0.90 | -4.12 | -1.18 |
| 54 | G03 | 1285 | COL4A3 | NM_000091 | 208026 | 205872 | 0.85 | 0.88 | -0.01 | 1.07 |
| 54 | G04 | 1293 | COL6A3 | NM_004369 | 202231 | 209263 | 0.83 | 0.90 | 0.42 | 0.20 |
| 54 | G05 | 1294 | COL7A1 | NM_000094 | 202119 | 204494 | 0.83 | 0.88 | -1.23 | -0.85 |
| 54 | G06 | 3730 | KAL1 | NM_000216 | 195541 | 207528 | 0.80 | 0.89 | 0.01 | -0.85 |
| 54 | G07 | 5627 | PROS1 | NM_000313 | 210632 | 211471 | 0.86 | 0.91 | -1.65 | -0.90 |
| 54 | G08 | 7057 | THBS1 | NM_003246 | 196885 | 207892 | 0.81 | 0.89 | -1.14 | -0.77 |
| 54 | G09 | 9112 | MTA1 | NM_004689 | 174648 | 186684 | 0.71 | 0.80 | 1.68 | 0.61 |
| 54 | G10 | 2107 | ETF1 | NM_004730 | 162344 | 163021 | 0.66 | 0.70 | 2.54 | 2.92 |
| 54 | G11 | 7980 | TFPI2 | NM_006528 | 178860 | 188773 | 0.73 | 0.81 | 0.74 | 0.17 |
| 54 | G12 | 10686 | CLDN16 | NM_006580 | 200270 | 200523 | 0.82 | 0.86 | -0.38 | -0.13 |
| 54 | H01 | NA | NA | NA | 213016 | 220854 | 0.87 | 0.95 | 0.81 | -0.35 |
| 54 | H02 | NA | pos | NA | 59139 | 105652 | 0.24 | 0.45 | 14.77 | 10.87 |
| 54 | H03 | 5473 | PPBP | NM_002704 | 236930 | 218288 | 0.97 | 0.94 | 0.01 | 1.66 |
| 54 | H04 | 163732 | CITED4 | NM_133467 | 240510 | 237980 | 0.98 | 1.02 | -0.42 | -0.80 |
| 54 | H05 | 4848 | CNOT2 | NM_014515 | 233407 | 207055 | 0.96 | 0.89 | -1.43 | 0.70 |
| 54 | H06 | 8726 | EED | NM_003797 | 229521 | 223542 | 0.94 | 0.96 | -0.44 | -0.61 |
| 54 | H07 | 80314 | EPC1 | NM_025209 | 219831 | 222925 | 0.90 | 0.96 | 0.15 | -0.21 |
| 54 | H08 | 2959 | GTF2B | NM_001514 | 211672 | 216987 | 0.87 | 0.93 | 0.15 | 0.15 |
| 54 | H09 | 2960 | GTF2E1 | NM_005513 | 222336 | 213847 | 0.91 | 0.92 | -0.01 | -0.23 |
| 54 | H10 | 2961 | GTF2E2 | NM_002095 | 222450 | 216115 | 0.91 | 0.93 | -0.28 | -0.45 |
| 54 | H11 | 4086 | SMAD1 | NM_005900 | 191539 | 195120 | 0.78 | 0.84 | 2.22 | 1.35 |
| 54 | H12 | 286 | ANK1 | NM_000037 | 201742 | 211148 | 0.83 | 0.91 | 2.12 | 0.64 |

| | | | | | | | | | | |
|---|---|---|---|---|---|---|---|---|---|---|
| 55 | A01 | NA | pos | NA | 49508 | 397852 | 0.21 | 1.66 | 20.28 | -24.21 |
| 55 | A02 | NA | NA | NA | 253970 | 244210 | 1.09 | 1.02 | -3.20 | -2.57 |
| 55 | A03 | 5245 | PHB | NM_002634 | 223551 | 259884 | 0.96 | 1.08 | 1.37 | -2.71 |
| 55 | A04 | 8464 | SUPT3H | NM_003599 | 243675 | 229162 | 1.05 | 0.96 | -1.15 | 0.13 |
| 55 | A05 | 9015 | TAF1A | NM_005681 | 217895 | 267912 | 0.94 | 1.12 | 0.77 | -6.00 |
| 55 | A06 | 9013 | TAF1C | NM_005679 | 213873 | 229672 | 0.92 | 0.96 | 1.98 | 0.32 |
| 55 | A07 | 56172 | ANKH | NM_054027 | 257967 | 229152 | 1.11 | 0.96 | -3.94 | -0.35 |
| 55 | A08 | 163 | AP2B1 | NM_001282 | 223132 | 218029 | 0.96 | 0.91 | 0.04 | 0.24 |
| 55 | A09 | 8546 | AP3B1 | NM_003664 | 222008 | 216886 | 0.96 | 0.90 | 0.35 | 0.51 |
| 55 | A10 | 341 | APOC1 | NM_001645 | 227433 | 229211 | 0.98 | 0.96 | -0.04 | -0.65 |
| 55 | A11 | 346 | APOC4 | NM_001646 | 234215 | 223533 | 1.01 | 0.93 | -1.75 | -0.15 |
| 55 | A12 | 55937 | APOM | NM_019101 | 249601 | 221831 | 1.08 | 0.93 | -1.57 | 1.20 |
| 55 | B01 | NA | neg | NA | 253124 | 256673 | 1.09 | 1.07 | -5.56 | -5.68 |
| 55 | B02 | NA | neg | NA | 235370 | 235225 | 1.01 | 0.98 | -3.52 | -2.66 |
| 55 | B03 | 358 | AQP1 | NM_000385 | 193142 | 220409 | 0.83 | 0.92 | 2.41 | 1.49 |
| 55 | B04 | 359 | AQP2 | NM_000486 | 208288 | 221380 | 0.90 | 0.92 | 0.46 | -0.13 |
| 55 | B05 | 360 | AQP3 | NM_004925 | 215680 | 213858 | 0.93 | 0.89 | -1.43 | 0.26 |
| 55 | B06 | 361 | AQP4 | NM_001650 | 209733 | 212302 | 0.90 | 0.89 | 0.00 | 1.40 |
| 55 | B07 | 362 | AQP5 | NM_001651 | 206216 | 209372 | 0.89 | 0.87 | -0.45 | 1.08 |
| 55 | B08 | 363 | AQP6 | NM_001652 | 202142 | 210969 | 0.87 | 0.88 | 0.00 | -0.12 |
| 55 | B09 | 364 | AQP7 | NM_001170 | 203634 | 210258 | 0.88 | 0.88 | 0.01 | 0.08 |
| 55 | B10 | 50617 | ATP6V0A4 | NM_020632 | 220197 | 216153 | 0.95 | 0.90 | -1.66 | -0.17 |
| 55 | B11 | 781 | CACNA2D1 | NM_000722 | 193364 | 221868 | 0.83 | 0.93 | 0.49 | -1.27 |
| 55 | B12 | 117155 | CATSPER2 | NM_054020 | 215605 | 222092 | 0.93 | 0.93 | -0.12 | -0.20 |
| 55 | C01 | NA | pos | NA | 53699 | 94270 | 0.23 | 0.39 | 15.72 | 15.88 |
| 55 | C02 | NA | NA | NA | 181842 | 193213 | 0.78 | 0.81 | 1.00 | 1.94 |
| 55 | C03 | 23230 | VPS13A | NM_015186 | 218123 | 203688 | 0.94 | 0.85 | -2.08 | 2.53 |
| 55 | C04 | 9635 | CLCA2 | NM_006536 | 192477 | 210205 | 0.83 | 0.88 | 0.66 | 0.13 |
| 55 | C05 | 1180 | CLCN1 | NM_000083 | 187380 | 187076 | 0.81 | 0.78 | 0.20 | 2.71 |
| 55 | C06 | 1181 | CLCN2 | NM_004366 | 185286 | 194304 | 0.80 | 0.81 | 1.19 | 2.62 |
| 55 | C07 | 1184 | CLCN5 | NM_000084 | 170644 | 184023 | 0.74 | 0.77 | 2.02 | 3.34 |
| 55 | C08 | 1186 | CLCN7 | NM_001287 | 208806 | 204070 | 0.90 | 0.85 | -2.39 | -0.46 |
| 55 | C09 | 1187 | CLCNKA | NM_004070 | 189573 | 218089 | 0.82 | 0.91 | 0.00 | -2.34 |
| 55 | C10 | 25932 | CLIC4 | NM_013943 | 191602 | 206945 | 0.83 | 0.86 | 0.00 | -0.19 |
| 55 | C11 | 53405 | CLIC5 | NM_016929 | 209922 | 218176 | 0.90 | 0.91 | -3.03 | -2.07 |
| 55 | C12 | 25839 | COG4 | NM_145818 | 223711 | 225038 | 0.96 | 0.94 | -2.67 | -1.93 |
| 55 | D01 | NA | neg | NA | 274393 | 275904 | 1.18 | 1.15 | -7.73 | -8.10 |
| 55 | D02 | NA | neg | NA | 221121 | 239505 | 0.95 | 1.00 | -1.61 | -2.98 |
| 55 | D03 | 27132 | CPNE7 | NM_014427 | 217142 | 233927 | 0.94 | 0.98 | -0.07 | -0.12 |
| 55 | D04 | 1497 | CTNS | NM_004937 | 237764 | 216716 | 1.02 | 0.90 | -2.65 | 0.82 |
| 55 | D05 | 9547 | CXCL14 | NM_004887 | 212992 | 203359 | 0.92 | 0.85 | -0.85 | 2.02 |
| 55 | D06 | 2054 | EPIM | NM_001980 | 217376 | 232647 | 0.94 | 0.97 | -0.60 | -1.17 |
| 55 | D07 | 2108 | ETFA | NM_000126 | 202440 | 221155 | 0.87 | 0.92 | 0.26 | -0.29 |
| 55 | D08 | 2109 | ETFB | NM_001985 | 195890 | 211307 | 0.84 | 0.88 | 0.98 | 0.12 |
| 55 | D09 | 5348 | FXYD1 | NM_005031 | 222259 | 218234 | 0.96 | 0.91 | -1.86 | -0.75 |
| 55 | D10 | 11345 | GABARAPL2 | NM_007285 | 200452 | 221110 | 0.86 | 0.92 | 0.88 | -0.58 |
| 55 | D11 | 2701 | GJA4 | NM_002060 | 199190 | 210808 | 0.86 | 0.88 | 0.09 | 0.58 |
| 55 | D12 | 10052 | GJA7 | NM_005497 | 215856 | 221582 | 0.93 | 0.92 | 0.12 | 0.16 |
| 55 | E01 | NA | neg | NA | 265643 | 240019 | 1.14 | 1.00 | -7.63 | -4.15 |
| 55 | E02 | NA | neg | NA | 225028 | 222120 | 0.97 | 0.93 | -2.97 | -1.63 |
| 55 | E03 | 2705 | GJB1 | NM_000166 | 207943 | 211160 | 0.90 | 0.88 | 0.07 | 1.98 |
| 55 | E04 | 2706 | GJB2 | NM_004004 | 214208 | 232167 | 0.92 | 0.97 | -0.85 | -2.47 |
| 55 | E05 | 2707 | GJB3 | NM_024009 | 195388 | 211686 | 0.84 | 0.88 | 0.27 | -0.26 |
| 55 | E06 | 127534 | GJB4 | NM_153212 | 187378 | 222720 | 0.81 | 0.93 | 1.94 | -0.88 |
| 55 | E07 | 2709 | GJB5 | NM_005268 | 201717 | 209184 | 0.87 | 0.87 | -0.57 | 0.29 |
| 55 | E08 | 10804 | GJB6 | NM_006783 | 172813 | 201472 | 0.74 | 0.84 | 2.73 | 0.40 |
| 55 | E09 | 3043 | HBB | NM_000518 | 190458 | 181730 | 0.82 | 0.76 | 0.88 | 3.28 |
| 55 | E10 | 3047 | HBG1 | NM_000559 | 200743 | 207878 | 0.86 | 0.87 | -0.06 | 0.17 |
| 55 | E11 | 3736 | KCNA1 | NM_000217 | 208215 | 223333 | 0.90 | 0.93 | -1.85 | -2.29 |
| 55 | E12 | 3738 | KCNA3 | NM_002232 | 215272 | 216027 | 0.93 | 0.90 | -0.72 | -0.16 |
| 55 | F01 | NA | NA | NA | 222594 | 253809 | 0.96 | 1.06 | -2.12 | -6.92 |
| 55 | F02 | NA | pos | NA | 67345 | 169637 | 0.29 | 0.71 | 15.71 | 4.94 |
| 55 | F03 | 8514 | KCNAB2 | NM_003636 | 221563 | 220173 | 0.95 | 0.92 | -0.92 | -0.11 |
| 55 | F04 | 9196 | KCNAB3 | NM_004732 | 213182 | 214039 | 0.92 | 0.89 | -0.17 | -0.74 |
| 55 | F05 | 3750 | KCND1 | NM_004979 | 201257 | 198628 | 0.87 | 0.83 | 0.16 | 0.76 |
| 55 | F06 | 3751 | KCND2 | NM_012281 | 215764 | 202974 | 0.93 | 0.85 | -0.75 | 1.08 |
| 55 | F07 | 3752 | KCND3 | NM_004980 | 196961 | 196537 | 0.85 | 0.82 | 0.55 | 1.25 |
| 55 | F08 | 3753 | KCNE1 | NM_000219 | 216002 | 212423 | 0.93 | 0.89 | -1.66 | -1.97 |
| 55 | F09 | 10008 | KCNE3 | NM_005472 | 206591 | 206139 | 0.89 | 0.86 | -0.40 | -0.98 |
| 55 | F10 | 81033 | KCNH6 | NM_030779 | 196347 | 199611 | 0.85 | 0.83 | 1.01 | 0.52 |
| 55 | F11 | 3767 | KCNJ11 | NM_000525 | 191417 | 200160 | 0.82 | 0.83 | 0.65 | 0.15 |
| 55 | F12 | 3759 | KCNJ2 | NM_000891 | 204907 | 215333 | 0.88 | 0.90 | 1.04 | -0.89 |
| 55 | G01 | NA | neg | NA | 228809 | 240597 | 0.99 | 1.00 | -3.53 | -5.09 |
| 55 | G02 | NA | neg | NA | 222250 | 219583 | 0.96 | 0.92 | -2.77 | -2.13 |
| 55 | G03 | 3764 | KCNJ8 | NM_004982 | 206701 | 219266 | 0.89 | 0.91 | 0.10 | -0.02 |
| 55 | G04 | 56659 | KCNK13 | NM_022054 | 197928 | 205918 | 0.85 | 0.86 | 0.90 | 0.38 |
| 55 | G05 | 3777 | KCNK3 | NM_002246 | 207450 | 235835 | 0.89 | 0.98 | -1.24 | -4.51 |
| 55 | G06 | 51305 | KCNK9 | NM_016601 | 218558 | 216574 | 0.94 | 0.90 | -1.77 | -0.87 |
| 55 | G07 | 3784 | KCNQ1 | NM_000218 | 197978 | 217781 | 0.85 | 0.91 | -0.26 | -1.78 |
| 55 | G08 | 3785 | KCNQ2 | NM_004518 | 196858 | 208201 | 0.85 | 0.87 | -0.16 | -1.40 |
| 55 | G09 | 3786 | KCNQ3 | NM_004519 | 197103 | 198850 | 0.85 | 0.83 | 0.00 | 0.02 |
| 55 | G10 | 9132 | KCNQ4 | NM_004700 | NA | 185578 | NA | 0.77 | NA | 2.46 |
| 55 | G11 | 3788 | KCNS2 | XM_043106 | 184823 | 195646 | 0.80 | 0.82 | 0.71 | 0.75 |
| 55 | G12 | 57582 | KCNT1 | XM_029962 | 201824 | 206142 | 0.87 | 0.86 | 0.70 | 0.38 |
| 55 | H01 | NA | NA | NA | 213494 | 212245 | 0.92 | 0.89 | -0.61 | -0.18 |
| 55 | H02 | NA | pos | NA | 71339 | 116693 | 0.31 | 0.49 | 15.72 | 13.28 |
| 55 | H03 | 169522 | KCNV2 | NM_133497 | 218575 | 225542 | 0.94 | 0.94 | -0.11 | 0.02 |
| 55 | H04 | 3840 | KPNA4 | NM_002268 | 214368 | 232657 | 0.92 | 0.97 | 0.17 | -2.47 |

| | | | | | | | | | | |
|---|---|---|---|---|---|---|---|---|---|---|
| 55 | H05 | 23633 | KPNA6 | NM_012316 | 208171 | 224899 | 0.90 | 0.94 | -0.16 | -2.05 |
| 55 | H06 | 57192 | MCOLN1 | NM_020533 | 213296 | 219159 | 0.92 | 0.91 | 0.00 | -0.32 |
| 55 | H07 | 55283 | MCOLN3 | NM_018298 | 199626 | 214664 | 0.86 | 0.90 | 0.71 | -0.42 |
| 55 | H08 | 23209 | MLC1 | NM_015166 | 205558 | 202772 | 0.89 | 0.85 | 0.00 | 0.28 |
| 55 | H09 | 7991 | TUSC3 | NM_006765 | 216161 | 205645 | 0.93 | 0.86 | -1.03 | -0.02 |
| 55 | H10 | 63908 | NAPB | NM_022080 | 196761 | 198303 | 0.85 | 0.83 | 1.43 | 1.59 |
| 55 | H11 | 4688 | NCF2 | NM_000433 | 201930 | 204827 | 0.87 | 0.85 | -0.09 | 0.38 |
| 55 | H12 | 4928 | NUP98 | NM_005387 | 177301 | 171671 | 0.76 | 0.72 | 4.68 | 6.15 |
| 56 | A01 | NA | pos | NA | 49837 | 58872 | 0.20 | 0.26 | 24.26 | 22.46 |
| 56 | A02 | NA | NA | NA | 225743 | 241474 | 0.92 | 1.06 | -0.19 | -2.08 |
| 56 | A03 | 29107 | NXT1 | NM_013248 | 262728 | 253780 | 1.07 | 1.11 | -2.82 | -2.29 |
| 56 | A04 | 5194 | PEX13 | NM_002618 | 230915 | 233032 | 0.94 | 1.02 | 0.00 | 0.00 |
| 56 | A05 | 5284 | PIGR | NM_002644 | 224906 | 223521 | 0.92 | 0.98 | 0.00 | 0.11 |
| 56 | A06 | 6248 | RSC1A1 | NM_006511 | 220898 | 222266 | 0.90 | 0.97 | 0.23 | 0.22 |
| 56 | A07 | 9522 | SCAMP1 | NM_004866 | 243428 | 232048 | 1.00 | 1.02 | -3.17 | -0.59 |
| 56 | A08 | 10067 | SCAMP3 | NM_005698 | 231349 | 214568 | 0.95 | 0.94 | -0.46 | 2.20 |
| 56 | A09 | 6323 | SCN1A | NM_006920 | 213454 | 218609 | 0.87 | 0.96 | 0.80 | -0.04 |
| 56 | A10 | 6324 | SCN1B | NM_001037 | 210708 | 220605 | 0.86 | 0.97 | 1.82 | -0.41 |
| 56 | A11 | 6326 | SCN2A2 | NM_021007 | 207799 | 218482 | 0.85 | 0.96 | 0.80 | 0.00 |
| 56 | A12 | 6328 | SCN3A | NM_006922 | 299741 | 217034 | 1.23 | 0.95 | -10.04 | 2.78 |
| 56 | B01 | NA | neg | NA | 239630 | 228430 | 0.98 | 1.00 | -5.26 | -2.30 |
| 56 | B02 | NA | neg | NA | 220298 | 223951 | 0.90 | 0.98 | -2.57 | -1.70 |
| 56 | B03 | 6329 | SCN4A | NM_000334 | 219893 | 217127 | 0.90 | 0.95 | 0.00 | 0.66 |
| 56 | B04 | 6331 | SCN5A | NM_000335 | 198609 | 228527 | 0.81 | 1.00 | 1.35 | -1.37 |
| 56 | B05 | 6337 | SCNN1A | NM_001038 | 195459 | 209110 | 0.80 | 0.92 | 0.95 | 0.07 |
| 56 | B06 | 6338 | SCNN1B | NM_000336 | 222482 | 219428 | 0.91 | 0.96 | -3.13 | -1.37 |
| 56 | B07 | 6340 | SCNN1G | NM_001039 | 221107 | 212679 | 0.90 | 0.93 | -3.20 | 0.04 |
| 56 | B08 | 6554 | SLC10A1 | NM_003049 | 205459 | 216532 | 0.84 | 0.95 | 0.00 | -0.04 |
| 56 | B09 | 6555 | SLC10A2 | NM_000452 | 197428 | 223606 | 0.81 | 0.98 | -0.11 | -2.69 |
| 56 | B10 | 6556 | SLC11A1 | NM_000578 | 226333 | 225398 | 0.93 | 0.99 | -3.49 | -3.03 |
| 56 | B11 | 4891 | SLC11A2 | NM_000617 | 187434 | 202524 | 0.77 | 0.89 | 0.49 | 0.17 |
| 56 | B12 | 6557 | SLC12A1 | NM_000338 | 201996 | 206917 | 0.83 | 0.91 | 0.40 | 2.16 |
| 56 | C01 | NA | pos | NA | 62034 | NA | 0.25 | NA | 20.31 | NA |
| 56 | C02 | NA | NA | NA | 197742 | 192006 | 0.81 | 0.84 | 1.45 | 2.63 |
| 56 | C03 | 6558 | SLC12A2 | NM_001046 | 212713 | 222296 | 0.87 | 0.97 | 1.89 | 0.00 |
| 56 | C04 | 9990 | SLC12A6 | NM_005135 | 219123 | NA | 0.90 | NA | -0.61 | NA |
| 56 | C05 | 6563 | SLC14A1 | NM_015865 | 197121 | 211543 | 0.81 | 0.93 | 1.61 | -0.21 |
| 56 | C06 | 8170 | SLC14A2 | NM_007163 | 194562 | 192190 | 0.80 | 0.84 | 1.64 | 2.32 |
| 56 | C07 | 6566 | SLC16A1 | NM_003051 | 194833 | 180239 | 0.80 | 0.79 | 1.34 | 4.44 |
| 56 | C08 | 26503 | SLC17A5 | NM_012434 | 278341 | 216231 | 1.14 | 0.95 | -9.24 | 0.04 |
| 56 | C09 | 6505 | SLC1A1 | NM_004170 | 209304 | 202494 | 0.86 | 0.89 | -0.87 | 0.19 |
| 56 | C10 | 116085 | SLC22A12 | NM_144585 | 208955 | 205175 | 0.85 | 0.90 | -0.18 | -0.27 |
| 56 | C11 | 6584 | SLC22A5 | NM_003060 | 196042 | 226037 | 0.80 | 0.99 | 0.18 | -2.95 |
| 56 | C12 | 1836 | SLC26A2 | NM_000112 | 230776 | 231688 | 0.94 | 1.01 | -2.71 | -1.13 |
| 56 | D01 | NA | neg | NA | 287220 | 238621 | 1.17 | 1.04 | -9.37 | -2.87 |
| 56 | D02 | NA | neg | NA | 247313 | 228494 | 1.01 | 1.00 | -3.83 | -1.51 |
| 56 | D03 | 5172 | SLC26A4 | NM_000441 | 243642 | 227937 | 1.00 | 1.00 | -0.80 | 0.00 |
| 56 | D04 | 6513 | SLC2A1 | NM_006516 | 209045 | 212126 | 0.85 | 0.93 | 2.40 | 1.63 |
| 56 | D05 | 6514 | SLC2A2 | NM_000340 | 209613 | 209786 | 0.86 | 0.92 | 1.49 | 0.78 |
| 56 | D06 | 6515 | SLC2A3 | NM_006931 | 228499 | 220789 | 0.93 | 0.97 | -1.47 | -0.76 |
| 56 | D07 | 6517 | SLC2A4 | NM_001042 | 215508 | 195953 | 0.88 | 0.86 | 0.08 | 3.08 |
| 56 | D08 | 6518 | SLC2A5 | NM_003039 | 194197 | 225673 | 0.79 | 0.99 | 4.07 | -0.47 |
| 56 | D09 | 29988 | SLC2A8 | NM_014580 | 220750 | 239136 | 0.90 | 1.05 | -0.85 | -3.98 |
| 56 | D10 | 56606 | SLC2A9 | NM_020041 | 210495 | 201548 | 0.86 | 0.88 | 1.22 | 0.97 |
| 56 | D11 | 7781 | SLC30A3 | NM_003459 | 211032 | 209685 | 0.86 | 0.92 | -0.29 | 0.00 |
| 56 | D12 | 55630 | SLC39A4 | NM_017767 | 223454 | 229545 | 0.91 | 1.00 | -0.08 | -0.08 |
| 56 | E01 | NA | neg | NA | 256309 | 271356 | 1.05 | 1.19 | -6.16 | -7.64 |
| 56 | E02 | NA | neg | NA | 241779 | 222612 | 0.99 | 0.97 | -4.14 | -1.09 |
| 56 | E03 | 6521 | SLC4A1 | NM_000342 | 220862 | 206995 | 0.90 | 0.91 | 1.28 | 2.45 |
| 56 | E04 | 6508 | SLC4A3 | NM_005070 | 218527 | 208050 | 0.89 | 0.91 | 0.00 | 1.81 |
| 56 | E05 | 8671 | SLC4A4 | NM_003759 | 235922 | 231892 | 0.96 | 1.02 | -3.26 | -2.56 |
| 56 | E06 | 6523 | SLC5A1 | NM_000343 | 192364 | 213249 | 0.79 | 0.93 | 2.47 | -0.11 |
| 56 | E07 | 6524 | SLC5A2 | NM_003041 | 208773 | 201262 | 0.85 | 0.88 | -0.08 | 2.00 |
| 56 | E08 | 6528 | SLC5A5 | NM_000453 | 185290 | 218563 | 0.76 | 0.96 | 4.22 | 0.12 |
| 56 | E09 | 6530 | SLC6A2 | NM_001043 | 209789 | 188854 | 0.86 | 0.83 | -0.41 | 2.41 |
| 56 | E10 | 6532 | SLC6A4 | NM_001045 | 210834 | 237814 | 0.86 | 1.04 | 0.08 | -4.27 |
| 56 | E11 | 6535 | SLC6A8 | NM_005629 | 203699 | 225322 | 0.83 | 0.99 | -0.36 | -2.47 |
| 56 | E12 | 9056 | SLC7A7 | NM_003982 | 215075 | 228048 | 0.88 | 1.00 | 0.00 | -0.25 |
| 56 | F01 | NA | NA | NA | 208310 | 243021 | 0.85 | 1.06 | 0.41 | -4.57 |
| 56 | F02 | NA | pos | NA | 69025 | 108568 | 0.28 | 0.48 | 19.77 | 13.49 |
| 56 | F03 | 11136 | SLC7A9 | NM_014270 | 246498 | 225650 | 1.01 | 0.99 | -2.38 | -0.80 |
| 56 | F04 | 6579 | SLCO1A2 | NM_005075 | 211370 | 226457 | 0.86 | 0.99 | 0.90 | -1.41 |
| 56 | F05 | 6804 | STX1A | NM_004603 | 219961 | 202846 | 0.90 | 0.89 | -1.13 | 0.60 |
| 56 | F06 | 10312 | TCIRG1 | NM_006019 | 208063 | 191072 | 0.85 | 0.84 | 0.19 | 2.12 |
| 56 | F07 | 6949 | TCOF1 | NM_000356 | 195381 | 212892 | 0.80 | 0.93 | 1.69 | -0.30 |
| 56 | F08 | 1678 | TIMM8A | NM_004085 | 216901 | 216448 | 0.89 | 0.95 | -0.27 | -0.34 |
| 56 | F09 | 10040 | TOM1L1 | NM_005486 | 194377 | 199001 | 0.79 | 0.87 | 1.64 | 0.30 |
| 56 | F10 | 219931 | TPCN2 | NM_139075 | 211359 | 193873 | 0.86 | 0.85 | -0.08 | 0.89 |
| 56 | F11 | 7220 | TRPC1 | NM_003304 | 205584 | 209583 | 0.84 | 0.92 | -0.71 | -1.09 |
| 56 | F12 | 7222 | TRPC3 | NM_003305 | 213822 | 213184 | 0.87 | 0.93 | 0.08 | 1.01 |
| 56 | G01 | NA | neg | NA | 250840 | 259264 | 1.03 | 1.13 | -6.26 | -5.80 |
| 56 | G02 | NA | neg | NA | 238666 | 227624 | 0.98 | 1.00 | -4.57 | -1.55 |
| 56 | G03 | 7224 | TRPC5 | NM_012471 | 216457 | 236779 | 0.89 | 1.04 | 1.03 | -1.34 |
| 56 | G04 | 7225 | TRPC6 | NM_004621 | 229296 | 217770 | 0.94 | 0.95 | -2.36 | 0.72 |
| 56 | G05 | 4308 | TRPM1 | NM_002420 | 206309 | 214998 | 0.84 | 0.94 | 0.00 | -0.07 |
| 56 | G06 | 7226 | TRPM2 | NM_003307 | 229345 | 213134 | 0.94 | 0.93 | -3.53 | 0.11 |
| 56 | G07 | 80036 | TRPM3 | NM_020952 | 208902 | 218060 | 0.85 | 0.95 | -0.95 | -0.04 |
| 56 | G08 | 59341 | TRPV4 | NM_021625 | NA | 290186 | NA | 1.27 | NA | -9.29 |

| | | | | | | | | | | | |
|---|---|---|---|---|---|---|---|---|---|---|---|
| 56 | G09 | 55240 | TSAP6 | NM_018234 | 199891 | 208055 | 0.82 | 0.91 | 0.11 | 0.04 |
| 56 | G10 | 7439 | VMD2 | NM_004183 | 211584 | 196140 | 0.87 | 0.86 | -0.88 | 1.55 |
| 56 | G11 | 7504 | XK | NM_021083 | 192250 | 208179 | 0.79 | 0.91 | 0.37 | 0.05 |
| 56 | G12 | 19 | ABCA1 | NM_005502 | 204084 | 231661 | 0.83 | 1.01 | 0.67 | -0.52 |
| 56 | H01 | NA | NA | NA | 223211 | 245957 | 0.91 | 1.08 | 0.11 | -2.75 |
| 56 | H02 | NA | pos | NA | 59843 | 106794 | 0.24 | 0.47 | 22.82 | 15.95 |
| 56 | H03 | 5243 | ABCB1 | NM_000927 | 242124 | 230689 | 0.99 | 1.01 | 0.00 | 0.74 |
| 56 | H04 | 22 | ABCB7 | NM_004299 | 230930 | 235760 | 0.94 | 1.03 | -0.05 | -0.44 |
| 56 | H05 | 4363 | ABCC1 | NM_004996 | 231086 | 232053 | 0.94 | 1.02 | -0.91 | -1.10 |
| 56 | H06 | 1244 | ABCC2 | NM_000392 | 223599 | 225732 | 0.91 | 0.99 | -0.19 | -0.31 |
| 56 | H07 | 8714 | ABCC3 | NM_003786 | 219237 | 227789 | 0.90 | 1.00 | 0.15 | -0.08 |
| 56 | H08 | 368 | ABCC6 | NM_001171 | 222141 | 227795 | 0.91 | 1.00 | 0.77 | 0.36 |
| 56 | H09 | 215 | ABCD1 | NM_000033 | 217586 | 219026 | 0.89 | 0.96 | 0.18 | -0.17 |
| 56 | H10 | 174 | AFP | NM_001134 | 221179 | 215037 | 0.90 | 0.94 | 0.32 | 0.27 |
| 56 | H11 | 310 | ANXA7 | NM_001156 | 214508 | 213229 | 0.88 | 0.93 | -0.18 | 0.64 |
| 56 | H12 | 335 | APOA1 | NM_000039 | 227160 | 236582 | 0.93 | 1.04 | 0.00 | 0.08 |
| 57 | A01 | NA | pos | NA | 59557 | 49426 | 0.25 | 0.20 | 20.85 | 27.74 |
| 57 | A02 | NA | NA | NA | 221325 | 233207 | 0.94 | 0.94 | 1.09 | -1.09 |
| 57 | A03 | 336 | APOA2 | NM_001643 | 262794 | 282501 | 1.11 | 1.14 | -2.87 | -7.65 |
| 57 | A04 | 337 | APOA4 | NM_000482 | 246475 | 233620 | 1.04 | 0.95 | -0.86 | 0.63 |
| 57 | A05 | 345 | APOC3 | NM_000040 | 231912 | 240874 | 0.98 | 0.98 | -0.01 | -0.21 |
| 57 | A06 | 347 | APOD | NM_001647 | 230301 | 225699 | 0.97 | 0.91 | 0.00 | 0.67 |
| 57 | A07 | 350 | APOH | NM_000042 | 225919 | 221286 | 0.96 | 0.90 | -0.05 | 0.20 |
| 57 | A08 | 8542 | APOL1 | NM_003661 | 230330 | 238636 | 0.97 | 0.97 | -0.02 | -2.92 |
| 57 | A09 | 636 | BICD1 | NM_001714 | 210109 | 222617 | 0.89 | 0.90 | 2.65 | -0.04 |
| 57 | A10 | 23299 | BICD2 | NM_015250 | 215025 | 220071 | 0.91 | 0.89 | 1.14 | 0.01 |
| 57 | A11 | 773 | CACNA1A | NM_000068 | 206133 | 217956 | 0.87 | 0.88 | 0.57 | -0.01 |
| 57 | A12 | 775 | CACNA1C | NM_000719 | 204945 | 217384 | 0.87 | 0.88 | 3.03 | 2.17 |
| 57 | B01 | NA | neg | NA | 230614 | 266228 | 0.98 | 1.08 | -2.12 | -7.74 |
| 57 | B02 | NA | neg | NA | 204230 | 228025 | 0.86 | 0.92 | 1.10 | -1.74 |
| 57 | B03 | 776 | CACNA1D | NM_000720 | 217409 | NA | 0.92 | NA | 0.59 | NA |
| 57 | B04 | 777 | CACNA1E | NM_000721 | 204823 | 228265 | 0.87 | 0.92 | 2.15 | 0.00 |
| 57 | B05 | 778 | CACNA1F | NM_005183 | 210825 | 226170 | 0.89 | 0.92 | 0.49 | 0.63 |
| 57 | B06 | 8912 | CACNA1H | NM_021098 | 213353 | 219394 | 0.90 | 0.89 | 0.00 | 0.19 |
| 57 | B07 | 8911 | CACNA1I | NM_021096 | 209728 | 218374 | 0.89 | 0.88 | -0.15 | -0.81 |
| 57 | B08 | 11126 | CD160 | NM_007053 | 213138 | 222478 | 0.90 | 0.90 | 0.00 | -1.85 |
| 57 | B09 | 1015 | CDH17 | NM_004063 | 217397 | 218636 | 0.92 | 0.89 | -0.31 | -0.88 |
| 57 | B10 | 1261 | CNGA3 | NM_001298 | 213290 | 216923 | 0.90 | 0.88 | -0.72 | -0.96 |
| 57 | B11 | 1258 | CNGB1 | NM_001297 | 194995 | 199062 | 0.82 | 0.81 | -0.14 | 1.48 |
| 57 | B12 | 1434 | CSE1L | NM_001316 | 206032 | 217148 | 0.87 | 0.88 | 0.81 | 0.74 |
| 57 | C01 | NA | pos | NA | 55663 | 102707 | 0.24 | 0.42 | 18.58 | 18.37 |
| 57 | C02 | NA | NA | NA | 166547 | 188809 | 0.70 | 0.76 | 5.04 | 4.87 |
| 57 | C03 | 1537 | CYC1 | NM_001916 | 204929 | 227302 | 0.87 | 0.92 | 1.45 | 0.00 |
| 57 | C04 | 2168 | FABP1 | NM_001443 | 246141 | 231186 | 1.04 | 0.94 | -3.56 | 0.00 |
| 57 | C05 | 2171 | FABP5 | NM_001444 | 192091 | 233146 | 0.81 | 0.94 | 2.11 | -0.01 |
| 57 | C06 | 2172 | FABP6 | NM_001445 | 199879 | 206475 | 0.85 | 0.84 | 0.98 | 2.67 |
| 57 | C07 | 2173 | FABP7 | NM_001446 | 176753 | 209492 | 0.75 | 0.85 | 3.21 | 1.04 |
| 57 | C08 | 2230 | FDX1 | NM_004109 | 229878 | 205590 | 0.97 | 0.83 | -2.71 | 1.26 |
| 57 | C09 | 11337 | GABARAP | NM_007278 | 211534 | 215658 | 0.89 | 0.87 | -0.26 | 0.04 |
| 57 | C10 | 2694 | GIF | NM_005142 | 224311 | 217449 | 0.95 | 0.88 | -2.74 | -0.59 |
| 57 | C11 | 57120 | GOPC | NM_020399 | 186215 | 211579 | 0.79 | 0.86 | 0.26 | -0.02 |
| 57 | C12 | 3039 | HBA1 | NM_000558 | 226040 | 230893 | 0.96 | 0.94 | -2.30 | -0.96 |
| 57 | D01 | NA | neg | NA | 236431 | 270849 | 1.00 | 1.10 | -1.95 | -7.38 |
| 57 | D02 | NA | neg | NA | 278826 | 239089 | 1.18 | 0.97 | -7.13 | -2.40 |
| 57 | D03 | 348980 | HCN1 | NM_021072 | 225786 | 239936 | 0.96 | 0.97 | 0.45 | -1.36 |
| 57 | D04 | 610 | HCN2 | NM_001194 | 232480 | 216636 | 0.98 | 0.88 | -0.35 | 2.90 |
| 57 | D05 | 57657 | HCN3 | NM_020897 | 235548 | 237169 | 1.00 | 0.96 | -1.65 | -0.02 |
| 57 | D06 | 10021 | HCN4 | NM_005477 | 199249 | 242510 | 0.84 | 0.98 | 2.60 | -2.36 |
| 57 | D07 | 3069 | HDLBP | NM_005336 | 207104 | 217423 | 0.88 | 0.88 | 1.05 | 0.42 |
| 57 | D08 | 3744 | KCNA10 | NM_005549 | 272392 | 225701 | 1.15 | 0.91 | -6.36 | -1.28 |
| 57 | D09 | 3737 | KCNA2 | NM_004974 | 229799 | 220840 | 0.97 | 0.89 | -0.95 | -0.15 |
| 57 | D10 | 3739 | KCNA4 | NM_002233 | 214525 | 208407 | 0.91 | 0.84 | 0.00 | 1.45 |
| 57 | D11 | 3741 | KCNA5 | NM_002234 | 198257 | 215291 | 0.84 | 0.87 | 0.34 | 0.01 |
| 57 | D12 | 3742 | KCNA6 | NM_002235 | 219900 | 228308 | 0.93 | 0.93 | 0.00 | 0.07 |
| 57 | E01 | NA | neg | NA | 266565 | 254548 | 1.13 | 1.03 | -6.05 | -5.27 |
| 57 | E02 | NA | neg | NA | 220921 | 232293 | 0.93 | 0.94 | -0.47 | -1.78 |
| 57 | E03 | 3743 | KCNA7 | NM_031886 | 217795 | 224979 | 0.92 | 0.91 | 1.01 | 0.54 |
| 57 | E04 | 3745 | KCNB1 | NM_004975 | 223421 | 240063 | 0.95 | 0.97 | 0.35 | -1.22 |
| 57 | E05 | 9312 | KCNB2 | NM_004770 | 238714 | 234159 | 1.01 | 0.95 | -2.45 | 0.01 |
| 57 | E06 | 3746 | KCNC1 | NM_004976 | 222411 | 220562 | 0.94 | 0.89 | -0.64 | 0.64 |
| 57 | E07 | 3747 | KCNC2 | NM_139136 | 219093 | 218134 | 0.93 | 0.88 | -0.82 | -0.14 |
| 57 | E08 | 3748 | KCNC3 | NM_004977 | 197710 | 212371 | 0.84 | 0.86 | 2.35 | 0.37 |
| 57 | E09 | 3749 | KCNC4 | NM_004978 | 181011 | 178714 | 0.77 | 0.72 | 4.60 | 6.01 |
| 57 | E10 | 3754 | KCNF1 | NM_002236 | 203523 | 214879 | 0.86 | 0.87 | 0.94 | -0.01 |
| 57 | E11 | 3755 | KCNG1 | NM_002237 | 204445 | 213028 | 0.86 | 0.86 | -0.83 | -0.08 |
| 57 | E12 | 26251 | KCNG2 | NM_012283 | 219340 | 227775 | 0.93 | 0.92 | -0.34 | -0.29 |
| 57 | F01 | NA | NA | NA | 229900 | 232269 | 0.97 | 0.94 | -2.53 | -3.27 |
| 57 | F02 | NA | pos | NA | 57086 | 146046 | 0.24 | 0.59 | 18.58 | 10.26 |
| 57 | F03 | 170850 | KCNG3 | NM_133329 | 222072 | 218462 | 0.94 | 0.89 | -0.48 | 0.07 |
| 57 | F04 | 3758 | KCNJ1 | NM_000220 | 205179 | 228380 | 0.87 | 0.93 | 1.61 | -0.88 |
| 57 | F05 | 3778 | KCNMA1 | NM_002247 | 211020 | 219301 | 0.89 | 0.89 | -0.03 | 0.85 |
| 57 | F06 | 3782 | KCNN3 | NM_002249 | 207913 | 225730 | 0.88 | 0.91 | 0.16 | -1.66 |
| 57 | F07 | 3787 | KCNS1 | NM_002251 | 188609 | 206814 | 0.80 | 0.84 | 1.93 | 0.14 |
| 57 | F08 | 3790 | KCNS3 | NM_002252 | 190339 | 202561 | 0.81 | 0.82 | 2.29 | 0.41 |
| 57 | F09 | 27012 | KCNV1 | NM_014379 | 210472 | 197125 | 0.89 | 0.80 | 0.03 | 1.63 |
| 57 | F10 | 80731 | KIAA1679 | XM_046570 | 209917 | 216274 | 0.89 | 0.88 | -0.81 | -1.72 |
| 57 | F11 | 23046 | KIF21B | XM_371332 | 206572 | 223806 | 0.87 | 0.91 | -2.06 | -3.26 |
| 57 | F12 | 3836 | KPNA1 | NM_002264 | 212190 | 216850 | 0.90 | 0.88 | -0.44 | -0.07 |

| | | | | | | | | | | |
|---|---|---|---|---|---|---|---|---|---|---|
| 57 | G01 | NA | neg | NA | 236303 | 256693 | 1.00 | 1.04 | -2.75 | -7.09 |
| 57 | G02 | NA | neg | NA | 237051 | 225541 | 1.00 | 0.91 | -2.85 | -2.21 |
| 57 | G03 | 3838 | KPNA2 | NM_002266 | 226425 | 231032 | 0.96 | 0.94 | -0.45 | -1.90 |
| 57 | G04 | 3839 | KPNA3 | NM_002267 | 233564 | 214515 | 0.99 | 0.87 | -1.30 | 1.30 |
| 57 | G05 | 3837 | KPNB1 | NM_002265 | 215316 | 222551 | 0.91 | 0.90 | 0.01 | 0.34 |
| 57 | G06 | 3842 | TNPO1 | NM_002270 | 229622 | 216373 | 0.97 | 0.88 | -1.93 | -0.19 |
| 57 | G07 | 3934 | LCN2 | NM_005564 | 216436 | 224220 | 0.92 | 0.91 | -0.90 | -2.58 |
| 57 | G08 | 4170 | MCL1 | NM_021960 | 207557 | 207563 | 0.88 | 0.84 | 0.75 | -0.37 |
| 57 | G09 | 4547 | MTP | NM_000253 | 205286 | 206511 | 0.87 | 0.84 | 1.23 | 0.16 |
| 57 | G10 | 10230 | NBR2 | NM_005821 | 207920 | 197428 | 0.88 | 0.80 | 0.00 | 1.24 |
| 57 | G11 | 9972 | NUP153 | NM_005124 | 193167 | 199767 | 0.82 | 0.81 | 0.14 | 0.52 |
| 57 | G12 | 63970 | P53AIP1 | NM_022112 | 208534 | 217506 | 0.88 | 0.88 | 0.57 | -0.17 |
| 57 | H01 | NA | NA | NA | 200414 | 199539 | 0.85 | 0.81 | 2.49 | 3.92 |
| 57 | H02 | NA | pos | NA | 71326 | 106631 | 0.30 | 0.43 | 18.26 | 18.50 |
| 57 | H03 | 5174 | PDZK1 | NM_002614 | 233548 | 218268 | 0.99 | 0.88 | -0.46 | 2.16 |
| 57 | H04 | 8682 | PEA15 | NM_003768 | 223028 | 271259 | 0.94 | 1.10 | 0.85 | -5.55 |
| 57 | H05 | 5311 | PKD2 | NM_000297 | 213140 | 244913 | 0.90 | 0.99 | 1.13 | -1.11 |
| 57 | H06 | 9033 | PKD2L1 | NM_016112 | 228463 | 233880 | 0.97 | 0.95 | -0.93 | -0.88 |
| 57 | H07 | 27039 | PKD2L2 | NM_014386 | 215675 | 222333 | 0.91 | 0.90 | 0.05 | -0.23 |
| 57 | H08 | 10343 | PKDREJ | NM_006071 | 220704 | 211755 | 0.93 | 0.86 | 0.00 | 1.03 |
| 57 | H09 | 5359 | PLSCR1 | NM_021105 | 222627 | 221337 | 0.94 | 0.90 | -0.03 | -0.11 |
| 57 | H10 | 57048 | PLSCR3 | NM_020360 | 212437 | 217749 | 0.90 | 0.88 | 0.30 | 0.11 |
| 57 | H11 | 64284 | RAB17 | NM_022449 | 204374 | 210324 | 0.86 | 0.85 | -0.37 | 0.91 |
| 57 | H12 | 55647 | RAB20 | NM_017817 | 220224 | 209611 | 0.93 | 0.85 | 0.00 | 3.12 |
| 58 | A01 | NA | pos | NA | 49790 | 72713 | 0.21 | 0.30 | 22.94 | 26.27 |
| 58 | A02 | NA | NA | NA | 251437 | 276472 | 1.07 | 1.13 | -3.89 | -6.70 |
| 58 | A03 | 23011 | RAB21 | NM_014999 | 265535 | 250255 | 1.13 | 1.02 | -4.51 | -0.90 |
| 58 | A04 | 51715 | RAB23 | NM_016277 | 221900 | 226403 | 0.95 | 0.93 | 0.87 | 2.08 |
| 58 | A05 | 57111 | RAB25 | NM_020387 | 228229 | 247383 | 0.97 | 1.01 | -0.13 | -1.78 |
| 58 | A06 | 9363 | RAB33A | NM_004794 | 224558 | 233191 | 0.96 | 0.95 | -0.04 | 0.18 |
| 58 | A07 | 83452 | RAB33B | NM_031296 | 233425 | 265127 | 1.00 | 1.08 | -1.16 | -4.74 |
| 58 | A08 | 51209 | RAB9B | NM_016370 | 215432 | 205988 | 0.92 | 0.84 | 0.13 | 3.62 |
| 58 | A09 | 6334 | SCN8A | NM_014191 | 208962 | 239763 | 0.89 | 0.98 | 1.25 | -0.92 |
| 58 | A10 | 6397 | SEC14L1 | NM_003003 | 212182 | 228418 | 0.91 | 0.93 | 0.05 | -0.03 |
| 58 | A11 | 266629 | SEC14L3 | NM_174975 | 223259 | 225713 | 0.95 | 0.92 | -1.32 | 0.03 |
| 58 | A12 | 10802 | SEC24A | XM_094581 | 215095 | 218832 | 0.92 | 0.90 | 0.68 | 3.08 |
| 58 | B01 | NA | neg | NA | 237947 | 251202 | 1.02 | 1.03 | -3.38 | -5.86 |
| 58 | B02 | NA | neg | NA | 215734 | 232117 | 0.92 | 0.95 | -0.43 | -2.78 |
| 58 | B03 | 10560 | SLC19A2 | NM_006996 | 222281 | 217887 | 0.95 | 0.89 | -0.04 | 1.09 |
| 58 | B04 | 6576 | SLC25A1 | NM_005984 | 210884 | 223364 | 0.90 | 0.91 | 1.05 | -0.68 |
| 58 | B05 | 10165 | SLC25A13 | NM_014251 | 225186 | 205261 | 0.96 | 0.84 | -1.01 | 1.78 |
| 58 | B06 | 10166 | SLC25A15 | NM_014252 | 214246 | 219604 | 0.92 | 0.90 | 0.04 | -0.87 |
| 58 | B07 | 60386 | SLC25A19 | NM_021734 | 224996 | 218498 | 0.96 | 0.89 | -1.32 | -0.44 |
| 58 | B08 | 788 | SLC25A20 | NM_000387 | 204613 | 216952 | 0.87 | 0.89 | 0.28 | -1.41 |
| 58 | B09 | 9481 | SLC25A27 | NM_004277 | 244967 | 207736 | 1.05 | 0.85 | -4.83 | 1.02 |
| 58 | B10 | 292 | SLC25A5 | NM_001152 | 216471 | 226771 | 0.92 | 0.93 | -1.81 | -3.02 |
| 58 | B11 | 6546 | SLC8A1 | NM_021097 | 198509 | 203016 | 0.85 | 0.83 | 0.69 | 0.45 |
| 58 | B12 | 6770 | STAR | NM_000349 | 204744 | 199371 | 0.87 | 0.82 | 0.77 | 2.98 |
| 58 | C01 | NA | pos | NA | 51845 | 62745 | 0.22 | 0.26 | 20.29 | 25.81 |
| 58 | C02 | NA | NA | NA | 174189 | 192230 | 0.74 | 0.79 | 4.01 | 4.86 |
| 58 | C03 | 6853 | SYN1 | NM_006950 | 200469 | 230478 | 0.86 | 0.94 | 1.78 | 0.23 |
| 58 | C04 | 6857 | SYT1 | NM_005639 | 221412 | 226967 | 0.95 | 0.93 | -1.44 | -0.08 |
| 58 | C05 | 6890 | TAP1 | NM_000593 | 194133 | 205120 | 0.83 | 0.84 | 2.03 | 2.98 |
| 58 | C06 | 6948 | TCN2 | NM_000355 | 206743 | 206257 | 0.88 | 0.84 | -0.05 | 2.47 |
| 58 | C07 | 10972 | TMP21 | NM_006827 | 204252 | 223227 | 0.87 | 0.91 | 0.35 | -0.03 |
| 58 | C08 | 7126 | TNFAIP1 | NM_021137 | 201576 | 215184 | 0.86 | 0.88 | -0.40 | 0.05 |
| 58 | C09 | 10043 | TOM1 | NM_005488 | 229067 | 219417 | 0.98 | 0.90 | -3.81 | 0.30 |
| 58 | C10 | 7274 | TTPA | NM_000370 | 194279 | 216742 | 0.83 | 0.89 | 0.06 | -0.22 |
| 58 | C11 | 7276 | TTR | NM_000371 | 183509 | 219076 | 0.78 | 0.90 | 1.60 | -0.97 |
| 58 | C12 | 7350 | UCP1 | NM_021833 | 223855 | 232573 | 0.96 | 0.95 | -2.86 | -1.22 |
| 58 | D01 | NA | neg | NA | 260765 | 273866 | 1.11 | 1.12 | -5.78 | -9.75 |
| 58 | D02 | NA | neg | NA | 230229 | 247565 | 0.98 | 1.01 | -1.72 | -5.50 |
| 58 | D03 | 7351 | UCP2 | NM_003355 | 227844 | 224643 | 0.97 | 0.92 | -0.14 | -0.22 |
| 58 | D04 | 7352 | UCP3 | NM_003356 | 223572 | 214559 | 0.96 | 0.88 | 0.00 | 0.53 |
| 58 | D05 | 213 | ALB | NM_000477 | 218013 | 227307 | 0.93 | 0.93 | 0.58 | -2.01 |
| 58 | D06 | 348 | APOE | NM_000041 | 199286 | 210486 | 0.85 | 0.86 | 2.67 | 0.39 |
| 58 | D07 | 2936 | GSR | NM_000637 | 231119 | 213808 | 0.99 | 0.87 | -1.50 | 0.09 |
| 58 | D08 | 4504 | MT3 | NM_005954 | 191459 | 198518 | 0.82 | 0.81 | 2.67 | 1.35 |
| 58 | D09 | 114112 | TXNRD3 | XM_051264 | 213921 | 215320 | 0.91 | 0.88 | -0.07 | -0.43 |
| 58 | D10 | 2 | A2M | NM_000014 | 211296 | 206633 | 0.90 | 0.85 | -0.48 | 0.02 |
| 58 | D11 | 55738 | ARFGAP1 | NM_018209 | 208438 | 204592 | 0.89 | 0.84 | 0.00 | -0.03 |
| 58 | D12 | 26286 | ARFGAP3 | NM_014570 | NA | 218734 | NA | 0.89 | NA | -0.38 |
| 58 | E01 | NA | neg | NA | 238728 | 250368 | 1.02 | 1.02 | -3.32 | -5.25 |
| 58 | E02 | NA | neg | NA | 227124 | 226938 | 0.97 | 0.93 | -1.78 | -1.46 |
| 58 | E03 | 10527 | IPO7 | NM_006391 | 223175 | 222951 | 0.95 | 0.91 | 0.01 | 0.75 |
| 58 | E04 | 5877 | RABIF | NM_002871 | 189808 | 198041 | 0.81 | 0.81 | 4.02 | 3.90 |
| 58 | E05 | 8795 | TNFRSF10B | NM_003842 | 223952 | 220038 | 0.96 | 0.90 | -0.69 | -0.13 |
| 58 | E06 | 9605 | C16ORF7 | NM_004913 | 188368 | 226123 | 0.80 | 0.93 | 3.65 | -1.44 |
| 58 | E07 | 3064 | HD | NM_002111 | 206729 | 217237 | 0.88 | 0.89 | 1.27 | 0.24 |
| 58 | E08 | 23541 | SEC14L2 | NM_012429 | 208751 | 215077 | 0.89 | 0.88 | -0.11 | -0.63 |
| 58 | E09 | 1811 | SLC26A3 | NM_000111 | 190606 | 209952 | 0.81 | 0.86 | 2.56 | 1.14 |
| 58 | E10 | 3933 | LCN1 | NM_002297 | 215730 | 210233 | 0.92 | 0.86 | -1.55 | 0.14 |
| 58 | E11 | 5350 | PLN | NM_002667 | 204953 | 219247 | 0.88 | 0.90 | 0.00 | -1.70 |
| 58 | E12 | 8086 | AAAS | NM_015665 | 216400 | 222974 | 0.92 | 0.91 | -0.62 | -0.36 |
| 58 | F01 | NA | NA | NA | 219892 | 253605 | 0.94 | 1.04 | -2.87 | -7.41 |
| 58 | F02 | NA | pos | NA | 305522 | 120276 | 1.31 | 0.49 | -14.26 | 14.17 |
| 58 | F03 | 13 | AADAC | NM_001086 | 207879 | 224315 | 0.89 | 0.92 | -0.01 | -1.10 |
| 58 | F04 | 89876 | AAT1 | NM_033364 | 210000 | 229696 | 0.90 | 0.94 | -0.72 | -2.86 |

| | | | | | | | | | | |
|---|---|---|---|---|---|---|---|---|---|---|
| 58 | F05 | 24 | ABCA4 | NM_000350 | 199137 | 208341 | 0.85 | 0.85 | 0.57 | 0.13 |
| 58 | F06 | 8647 | ABCB11 | NM_003742 | 196949 | 190319 | 0.84 | 0.78 | 0.46 | 2.72 |
| 58 | F07 | 10057 | ABCC5 | NM_005688 | 202320 | 208426 | 0.86 | 0.85 | -0.19 | 0.03 |
| 58 | F08 | 6833 | ABCC8 | NM_000352 | 191761 | 201449 | 0.82 | 0.82 | 0.11 | -0.05 |
| 58 | F09 | 3983 | ABLIM1 | NM_002313 | 194419 | 205233 | 0.83 | 0.84 | 0.01 | 0.27 |
| 58 | F10 | 10449 | ACAA2 | NM_006111 | 179768 | 201121 | 0.77 | 0.82 | 1.19 | -0.02 |
| 58 | F11 | 39 | ACAT2 | NM_005891 | 208057 | 214290 | 0.89 | 0.88 | -2.47 | -2.53 |
| 58 | F12 | 8309 | ACOX2 | NM_003500 | 202606 | 210197 | 0.87 | 0.86 | -0.83 | 0.07 |
| 58 | G01 | NA | neg | NA | 238192 | 241321 | 1.02 | 0.99 | -2.86 | -3.35 |
| 58 | G02 | NA | neg | NA | 230196 | 222209 | 0.98 | 0.91 | -1.79 | -0.26 |
| 58 | G03 | 10121 | ACTR1A | NM_005736 | 221384 | 253576 | 0.95 | 1.04 | 0.64 | -3.77 |
| 58 | G04 | 10097 | ACTR2 | NM_005722 | 223001 | 224326 | 0.95 | 0.92 | 0.00 | 0.08 |
| 58 | G05 | 10096 | ACTR3 | NM_005721 | 224463 | 216954 | 0.96 | 0.89 | -0.36 | 0.81 |
| 58 | G06 | 97 | ACYP1 | XM_352906 | 227808 | 221021 | 0.97 | 0.90 | -1.20 | -0.18 |
| 58 | G07 | 57405 | SPC25 | NM_020675 | 212658 | 233870 | 0.91 | 0.96 | 0.88 | -2.02 |
| 58 | G08 | 8038 | ADAM12 | NM_003474 | 213722 | 212986 | 0.91 | 0.87 | -0.37 | 0.15 |
| 58 | G09 | 1596 | ADAM3B | Y10615 | 212860 | 221329 | 0.91 | 0.91 | 0.00 | -0.27 |
| 58 | G10 | 170690 | ADAMTS16 | NM_139056 | 207469 | 202570 | 0.89 | 0.83 | -0.05 | 1.81 |
| 58 | G11 | 170692 | ADAMTS18 | NM_139054 | 200667 | 199437 | 0.86 | 0.82 | 0.96 | 1.94 |
| 58 | G12 | 92949 | ADAMTSL1 | NM_052866 | 203599 | 223843 | 0.87 | 0.92 | 1.48 | -0.07 |
| 58 | H01 | NA | NA | NA | 201643 | 205234 | 0.86 | 0.84 | 1.18 | 1.83 |
| 58 | H02 | NA | pos | NA | 61778 | 95792 | 0.26 | 0.39 | 19.79 | 19.55 |
| 58 | H03 | 125 | ADH1B | NM_000668 | 213257 | 224859 | 0.91 | 0.92 | 0.90 | 0.22 |
| 58 | H04 | 127 | ADH4 | NM_000670 | 224622 | 234767 | 0.96 | 0.96 | -1.04 | -2.26 |
| 58 | H05 | 128 | ADH5 | NM_000671 | 214583 | 221145 | 0.92 | 0.90 | 0.13 | -0.53 |
| 58 | H06 | 142 | PARP1 | NM_001618 | 233670 | 220670 | 1.00 | 0.90 | -2.81 | -0.78 |
| 58 | H07 | 158056 | AEGP | XM_088463 | 211615 | 216800 | 0.90 | 0.89 | 0.19 | 0.09 |
| 58 | H08 | 10962 | AF1Q | NM_006818 | 219574 | 210252 | 0.94 | 0.86 | -1.97 | -0.07 |
| 58 | H09 | 178 | AGL | NM_000028 | 206673 | 221576 | 0.88 | 0.91 | 0.00 | -0.96 |
| 58 | H10 | 57085 | AGTRAP | NM_020350 | 198190 | 203383 | 0.85 | 0.83 | 0.36 | 1.03 |
| 58 | H11 | 10598 | AHSA1 | NM_012111 | 207173 | 203244 | 0.89 | 0.83 | -0.73 | 0.67 |
| 58 | H12 | 9863 | AIP1 | NM_012301 | 208535 | 216576 | 0.89 | 0.89 | 0.00 | 0.45 |
| 59 | A01 | NA | pos | NA | 47217 | 62531 | 0.20 | 0.27 | 21.24 | 26.16 |
| 59 | A02 | NA | NA | NA | 223023 | 241831 | 0.95 | 1.06 | 0.30 | -3.88 |
| 59 | A03 | 158798 | AKAP28 | NM_178813 | 286412 | 242169 | 1.21 | 1.06 | -4.75 | -1.74 |
| 59 | A04 | 54998 | AKIP | NM_017900 | 238356 | 228187 | 1.01 | 1.00 | 0.71 | 0.08 |
| 59 | A05 | 10327 | AKR1A1 | NM_006066 | 232279 | 216572 | 0.99 | 0.95 | 0.67 | 1.55 |
| 59 | A06 | 231 | AKR1B1 | NM_001628 | 270475 | 224295 | 1.15 | 0.98 | -3.83 | 0.54 |
| 59 | A07 | 83592 | AKR1CL2 | NM_031436 | 236338 | 217937 | 1.00 | 0.95 | -1.72 | 0.18 |
| 59 | A08 | 210 | ALAD | NM_000031 | 225590 | 202246 | 0.96 | 0.89 | 0.00 | 1.69 |
| 59 | A09 | 216 | ALDH1A1 | NM_000689 | 206917 | 216554 | 0.88 | 0.95 | 0.84 | -1.38 |
| 59 | A10 | 224 | ALDH3A2 | NM_000382 | 213252 | 218623 | 0.90 | 0.96 | 0.38 | -0.08 |
| 59 | A11 | 223 | ALDH9A1 | NM_000696 | 231893 | 224009 | 0.98 | 0.98 | -1.71 | -2.24 |
| 59 | A12 | 229 | ALDOB | NM_000035 | 225427 | 228954 | 0.96 | 1.00 | 0.00 | -1.78 |
| 59 | B01 | NA | neg | NA | 234614 | 230817 | 1.00 | 1.01 | -2.71 | -4.29 |
| 59 | B02 | NA | neg | NA | 212096 | 207554 | 0.90 | 0.91 | -0.03 | -0.40 |
| 59 | B03 | 230 | ALDOC | NM_005165 | 230117 | 218992 | 0.98 | 0.96 | 0.33 | -0.11 |
| 59 | B04 | 29929 | ALG6 | NM_013339 | 209742 | 199092 | 0.89 | 0.87 | 2.49 | 2.70 |
| 59 | B05 | 8846 | ALKBH | NM_006020 | 193691 | 199426 | 0.82 | 0.87 | 3.64 | 2.17 |
| 59 | B06 | 7840 | ALMS1 | NM_015120 | 227397 | 212740 | 0.96 | 0.93 | -0.32 | 0.22 |
| 59 | B07 | 240 | ALOX5 | NM_000698 | 232789 | 214005 | 0.99 | 0.94 | -2.92 | -1.42 |
| 59 | B08 | 248 | ALPI | NM_001631 | 218406 | 216420 | 0.93 | 0.95 | -0.77 | -2.95 |
| 59 | B09 | 250 | ALPP | NM_001632 | 214262 | 198215 | 0.91 | 0.87 | -1.66 | -0.57 |
| 59 | B10 | 130540 | ALS2CR12 | NM_139163 | 216897 | 216905 | 0.92 | 0.95 | -1.68 | -2.05 |
| 59 | B11 | 65068 | ALS2CR14 | NM_178231 | 187753 | 194409 | 0.80 | 0.85 | 1.92 | 0.47 |
| 59 | B12 | 65062 | ALS2CR4 | NM_152388 | 199351 | 204162 | 0.85 | 0.89 | 1.48 | 0.11 |
| 59 | C01 | NA | pos | NA | 60040 | 73611 | 0.25 | 0.32 | 19.13 | 23.27 |
| 59 | C02 | NA | NA | NA | 180834 | 176629 | 0.77 | 0.77 | 4.74 | 6.01 |
| 59 | C03 | 79800 | ALS2CR8 | NM_024744 | 237807 | 225615 | 1.01 | 0.99 | 0.46 | 0.00 |
| 59 | C04 | 9949 | AMMECR1 | NM_015365 | 274623 | 193141 | 1.16 | 0.85 | -4.20 | 4.92 |
| 59 | C05 | 81693 | AMN | NM_030943 | 236792 | 226973 | 1.00 | 0.99 | -0.45 | -1.23 |
| 59 | C06 | 270 | AMPD1 | NM_000036 | 220292 | 222663 | 0.93 | 0.98 | 1.57 | -0.22 |
| 59 | C07 | 57559 | AMSH-LP | NM_020799 | 204156 | 182085 | 0.87 | 0.80 | 1.53 | 5.15 |
| 59 | C08 | 56311 | ANKRD7 | NM_019644 | 225733 | 206137 | 0.96 | 0.90 | -0.60 | 0.00 |
| 59 | C09 | 54443 | ANLN | NM_018685 | 198149 | 193074 | 0.84 | 0.85 | 1.30 | 1.52 |
| 59 | C10 | 23520 | ANP32C | NM_012403 | 225558 | 213508 | 0.96 | 0.94 | -1.67 | -0.25 |
| 59 | C11 | 23519 | ANP32D | NM_012404 | 205808 | 201326 | 0.87 | 0.88 | 0.82 | 0.53 |
| 59 | C12 | 290 | ANPEP | NM_001150 | 230953 | 220319 | 0.98 | 0.97 | -1.24 | -1.37 |
| 59 | D01 | NA | neg | NA | 289741 | 276564 | 1.23 | 1.21 | -8.96 | -9.70 |
| 59 | D02 | NA | neg | NA | 254506 | 225742 | 1.08 | 0.99 | -4.76 | -1.18 |
| 59 | D03 | 301 | ANXA1 | NM_000700 | 257908 | 231806 | 1.09 | 1.02 | -2.66 | 0.00 |
| 59 | D04 | 302 | ANXA2 | NM_004039 | 238524 | 208975 | 1.01 | 0.92 | -0.62 | 3.30 |
| 59 | D05 | 309 | ANXA6 | NM_001155 | 228351 | 225306 | 0.97 | 0.99 | -0.17 | 0.09 |
| 59 | D06 | 244 | ANXA8 | NM_001630 | 224700 | 233827 | 0.95 | 1.02 | 0.32 | -1.05 |
| 59 | D07 | 8416 | ANXA9 | NM_003568 | 192291 | 219327 | 0.82 | 0.96 | 2.22 | -0.05 |
| 59 | D08 | 11276 | AP1GBP1 | NM_007247 | 201691 | 194474 | 0.86 | 0.85 | 1.54 | 2.99 |
| 59 | D09 | 327 | APEH | NM_001640 | 222793 | 208339 | 0.94 | 0.91 | -2.36 | 0.00 |
| 59 | D10 | 328 | APEX1 | NM_001641 | 198103 | 215661 | 0.84 | 0.94 | 0.88 | 0.43 |
| 59 | D11 | 9140 | APG12L | NM_004707 | 213220 | 215946 | 0.90 | 0.95 | -0.79 | -0.88 |
| 59 | D12 | 9474 | APG5L | NM_004849 | 213004 | 220954 | 0.90 | 0.97 | 0.17 | -0.44 |
| 59 | E01 | NA | neg | NA | 260030 | 253153 | 1.10 | 1.11 | -6.25 | -8.30 |
| 59 | E02 | NA | neg | NA | 236964 | 217554 | 1.00 | 0.95 | -3.51 | -2.33 |
| 59 | E03 | 8539 | API5 | NM_006595 | 219874 | 215602 | 0.93 | 0.94 | 1.04 | 0.19 |
| 59 | E04 | 80350 | LPAL2 | NM_024492 | 227943 | 219377 | 0.97 | 0.96 | -0.20 | -0.97 |
| 59 | E05 | 338 | APOB | NM_000384 | 218519 | 221697 | 0.93 | 0.97 | 0.17 | -1.83 |
| 59 | E06 | 339 | APOBEC1 | NM_001644 | 211667 | 220087 | 0.90 | 0.96 | 1.04 | -1.27 |
| 59 | E07 | 9582 | APOBEC3B | NM_004900 | 215437 | 184885 | 0.91 | 0.81 | -1.37 | 3.20 |
| 59 | E08 | 140564 | APOBEC3D | AL022318 | 206278 | 195697 | 0.87 | 0.86 | 0.16 | 0.26 |

| | | | | | | | | | | |
|---|---|---|---|---|---|---|---|---|---|---|
| 59 | E09 | 200316 | APOBEC3F | NM_145298 | 187512 | 193259 | 0.80 | 0.85 | 1.01 | 0.00 |
| 59 | E10 | 319 | APOF | NM_001638 | 201432 | 189184 | 0.85 | 0.83 | -0.35 | 2.34 |
| 59 | E11 | 353 | APRT | NM_000485 | 200841 | 203875 | 0.85 | 0.89 | -0.15 | -1.38 |
| 59 | E12 | 54840 | APTX | NM_175069 | 222987 | NA | 0.95 | NA | -1.85 | NA |
| 59 | F01 | NA | NA | NA | 222932 | 243257 | 0.95 | 1.07 | -2.50 | -7.00 |
| 59 | F02 | NA | pos | NA | 67744 | 136144 | 0.29 | 0.60 | 15.98 | 10.95 |
| 59 | F03 | 343 | AQP8 | NM_001169 | 221769 | 212378 | 0.94 | 0.93 | 0.15 | 0.37 |
| 59 | F04 | 366 | AQP9 | NM_020980 | 231947 | 222995 | 0.98 | 0.98 | -1.34 | -1.93 |
| 59 | F05 | 375 | ARF1 | NM_001658 | 234217 | 209174 | 0.99 | 0.92 | -2.37 | -0.09 |
| 59 | F06 | 377 | ARF3 | NM_001659 | 219738 | 212244 | 0.93 | 0.93 | -0.59 | -0.32 |
| 59 | F07 | 378 | ARF4 | NM_001660 | 196341 | 204829 | 0.83 | 0.90 | 0.24 | -0.50 |
| 59 | F08 | 381 | ARF5 | NM_001662 | 202030 | 195124 | 0.86 | 0.85 | 0.00 | 0.00 |
| 59 | F09 | 382 | ARF6 | NM_001663 | 198677 | 190599 | 0.84 | 0.83 | -0.99 | 0.09 |
| 59 | F10 | 26119 | ARH | NM_015627 | 191740 | 200520 | 0.81 | 0.88 | 0.14 | 0.08 |
| 59 | F11 | 387 | RHOA | NM_001664 | 185247 | 182766 | 0.79 | 0.80 | 1.04 | 1.80 |
| 59 | F12 | 390 | ARHE | NM_005168 | NA | 201122 | NA | 0.88 | NA | 0.00 |
| 59 | G01 | NA | neg | NA | 216644 | 245675 | 0.92 | 1.08 | -1.00 | -7.00 |
| 59 | G02 | NA | neg | NA | 224709 | 209732 | 0.95 | 0.92 | -1.96 | -0.97 |
| 59 | G03 | 23779 | ARHGAP8 | NM_181334 | 230542 | 220564 | 0.98 | 0.97 | -0.15 | -0.59 |
| 59 | G04 | 9639 | ARHGEF10 | NM_014629 | 218494 | 214371 | 0.93 | 0.94 | 1.02 | -0.08 |
| 59 | G05 | 22899 | ARHGEF15 | NM_173728 | 223931 | 220116 | 0.95 | 0.96 | -0.39 | -1.51 |
| 59 | G06 | 27237 | ARHGEF16 | NM_014448 | 235110 | 211151 | 1.00 | 0.92 | -1.67 | 0.27 |
| 59 | G07 | 23229 | ARHGEF9 | XM_377014 | 206717 | 203996 | 0.88 | 0.89 | -0.24 | 0.05 |
| 59 | G08 | 171177 | RHOV | NM_133639 | 201110 | 206124 | 0.85 | 0.90 | 0.86 | -1.43 |
| 59 | G09 | 26225 | ARL5 | NM_012097 | 191380 | 193567 | 0.81 | 0.85 | 0.63 | 0.00 |
| 59 | G10 | 51329 | ARL6IP4 | NM_016638 | 200373 | 188199 | 0.85 | 0.82 | -0.14 | 2.55 |
| 59 | G11 | 10552 | ARPC1A | NM_006409 | 199071 | 193556 | 0.84 | 0.85 | 0.15 | 0.40 |
| 59 | G12 | 10093 | ARPC4 | NM_005718 | 205900 | 203579 | 0.87 | 0.89 | 0.27 | 0.00 |
| 59 | H01 | NA | NA | NA | 186844 | 201143 | 0.79 | 0.88 | 3.20 | 1.76 |
| 59 | H02 | NA | pos | NA | 57439 | 100008 | 0.24 | 0.44 | 18.61 | 18.71 |
| 59 | H03 | 10092 | ARPC5 | NM_005717 | 240143 | 217765 | 1.02 | 0.95 | -0.64 | 1.17 |
| 59 | H04 | 415 | ARSE | NM_000047 | 230848 | 227124 | 0.98 | 0.99 | 0.20 | -0.92 |
| 59 | H05 | 51676 | ASB2 | NM_016150 | 213486 | 213841 | 0.91 | 0.94 | 1.51 | 0.83 |
| 59 | H06 | 432 | ASGR1 | NM_001671 | 219773 | 215973 | 0.93 | 0.95 | 0.81 | 0.76 |
| 59 | H07 | 435 | ASL | NM_000048 | 207460 | 213083 | 0.88 | 0.93 | 0.32 | -0.18 |
| 59 | H08 | 259266 | ASPM | NM_018136 | 216653 | 209634 | 0.92 | 0.92 | -0.34 | -0.73 |
| 59 | H09 | 79058 | ASPSCR1 | NM_024083 | 207500 | 200179 | 0.88 | 0.88 | -0.63 | 0.18 |
| 59 | H10 | 1386 | ATF2 | NM_001880 | 202801 | 213468 | 0.86 | 0.94 | 0.22 | -0.39 |
| 59 | H11 | 80063 | ATF7IP2 | NM_024997 | 211240 | 206037 | 0.90 | 0.90 | -0.65 | -0.40 |
| 59 | H12 | 488 | ATP2A2 | NM_001681 | 215236 | 209232 | 0.91 | 0.92 | -0.19 | 0.34 |
| 60 | A01 | NA | pos | NA | 53044 | 68945 | 0.22 | 0.27 | 28.11 | 28.05 |
| 60 | A02 | NA | NA | NA | 252192 | 256354 | 1.03 | 1.02 | -4.54 | -3.11 |
| 60 | A03 | 509 | ATP5C1 | NM_005174 | 234147 | 287375 | 0.96 | 1.14 | -0.73 | -6.97 |
| 60 | A04 | 10476 | ATP5H | NM_006356 | 275987 | 242423 | 1.13 | 0.96 | -7.51 | -0.22 |
| 60 | A05 | 57198 | ATP8B2 | NM_020452 | 217018 | 237502 | 0.89 | 0.94 | 1.99 | 1.33 |
| 60 | A06 | 546 | ATRX | NM_000489 | 231925 | 247043 | 0.95 | 0.98 | -0.20 | -1.17 |
| 60 | A07 | 64651 | AXUD1 | NM_033027 | 209884 | 235630 | 0.86 | 0.93 | 1.95 | 0.22 |
| 60 | A08 | 567 | B2M | NM_004048 | 219515 | 317905 | 0.90 | 1.26 | -0.25 | -13.23 |
| 60 | A09 | 573 | BAG1 | NM_004323 | 220845 | 238376 | 0.90 | 0.94 | -1.90 | -1.51 |
| 60 | A10 | 10458 | BAIAP2 | NM_006340 | 217449 | 222709 | 0.89 | 0.88 | 0.20 | 0.43 |
| 60 | A11 | 578 | BAK1 | NM_001188 | 192008 | 199005 | 0.79 | 0.79 | 2.35 | 4.64 |
| 60 | A12 | 54971 | BANP | NM_017869 | 210771 | 207877 | 0.86 | 0.82 | 2.70 | 4.74 |
| 60 | B01 | NA | neg | NA | 276151 | 255046 | 1.13 | 1.01 | -10.80 | -5.92 |
| 60 | B02 | NA | neg | NA | 228798 | 249515 | 0.94 | 0.99 | -3.03 | -5.00 |
| 60 | B03 | 55973 | BCAP29 | NM_018844 | 215159 | 217405 | 0.88 | 0.86 | 0.05 | 1.64 |
| 60 | B04 | 8538 | BARX2 | NM_003658 | 232051 | 222860 | 0.95 | 0.88 | -2.64 | 0.01 |
| 60 | B05 | 317716 | BASE | NM_173859 | 211975 | 221816 | 0.87 | 0.88 | 0.49 | 0.91 |
| 60 | B06 | 10409 | BASP1 | NM_006317 | 197533 | 226929 | 0.81 | 0.90 | 3.11 | -0.85 |
| 60 | B07 | 581 | BAX | NM_004324 | 207584 | 218444 | 0.85 | 0.87 | 0.00 | 0.05 |
| 60 | B08 | 27113 | BBC3 | NM_014417 | 212467 | 220914 | 0.87 | 0.88 | -1.43 | -0.13 |
| 60 | B09 | 582 | BBS1 | NM_024649 | 225468 | 211092 | 0.92 | 0.84 | -4.99 | 0.00 |
| 60 | B10 | 583 | BBS2 | NM_031885 | 224453 | 221877 | 0.92 | 0.88 | -3.28 | -2.46 |
| 60 | B11 | 585 | BBS4 | NM_033028 | 191278 | 205286 | 0.78 | 0.81 | 0.14 | 0.57 |
| 60 | B12 | 55212 | BBS7 | NM_018190 | 212984 | 222069 | 0.87 | 0.88 | 0.00 | -0.64 |
| 60 | C01 | NA | pos | NA | 67621 | 138420 | 0.28 | 0.55 | 23.68 | 13.45 |
| 60 | C02 | NA | NA | NA | 171991 | 191451 | 0.70 | 0.76 | 6.57 | 4.63 |
| 60 | C03 | 9564 | BCAR1 | NM_014567 | 216757 | 225788 | 0.89 | 0.89 | 0.08 | 0.22 |
| 60 | C04 | 8537 | BCAS1 | NM_003657 | 207549 | 219529 | 0.85 | 0.87 | 1.67 | 0.54 |
| 60 | C05 | 56647 | BCCIP | NM_016567 | 207704 | 234304 | 0.85 | 0.93 | 1.48 | -1.19 |
| 60 | C06 | 597 | BCL2A1 | NM_004049 | 190271 | 221044 | 0.78 | 0.88 | 4.59 | 0.10 |
| 60 | C07 | 598 | BCL2L1 | NM_138578 | 214719 | 218937 | 0.88 | 0.87 | -0.88 | -0.05 |
| 60 | C08 | 10017 | BCL2L10 | NM_020396 | 206013 | 225423 | 0.84 | 0.89 | -0.08 | -0.90 |
| 60 | C09 | 10018 | BCL2L11 | NM_006538 | 210324 | 229903 | 0.86 | 0.91 | -2.22 | -3.15 |
| 60 | C10 | 79370 | BCL2L14 | NM_030766 | 202073 | 206332 | 0.83 | 0.82 | 0.68 | 0.10 |
| 60 | C11 | 599 | BCL2L2 | NM_004050 | 205828 | 208213 | 0.84 | 0.83 | -1.96 | 0.06 |
| 60 | C12 | 602 | BCL3 | NM_005178 | 217459 | 235482 | 0.89 | 0.93 | -0.44 | -2.90 |
| 60 | D01 | NA | neg | NA | 288407 | 283890 | 1.18 | 1.13 | -11.66 | -9.12 |
| 60 | D02 | NA | neg | NA | 243314 | 246983 | 1.00 | 0.98 | -4.27 | -2.98 |
| 60 | D03 | 607 | BCL9 | NM_004326 | 208178 | 230006 | 0.85 | 0.91 | 2.34 | 1.14 |
| 60 | D04 | 7851 | BENE | NM_005434 | 216346 | 232523 | 0.89 | 0.92 | 1.08 | 0.00 |
| 60 | D05 | 8419 | BFSP2 | NM_003571 | 227715 | 241040 | 0.93 | 0.96 | -0.95 | -0.69 |
| 60 | D06 | 201163 | BHD | NM_144606 | 223491 | 223331 | 0.92 | 0.89 | 0.00 | 1.34 |
| 60 | D07 | 25893 | BIA2 | NM_015431 | 214561 | 220650 | 0.88 | 0.87 | 0.00 | 1.28 |
| 60 | D08 | 637 | BID | NM_001196 | 210266 | 219800 | 0.86 | 0.87 | 0.08 | 1.66 |
| 60 | D09 | 638 | BIK | NM_001197 | 194817 | 220662 | 0.80 | 0.87 | 1.17 | 0.00 |
| 60 | D10 | 274 | BIN1 | NM_004305 | 215645 | 228126 | 0.88 | 0.90 | -0.70 | -1.90 |
| 60 | D11 | 51411 | BIN2 | NM_016187 | 199940 | 227042 | 0.82 | 0.90 | -0.14 | -1.45 |
| 60 | D12 | 10904 | BLCAP | NM_006698 | 222752 | 228487 | 0.91 | 0.91 | -0.46 | -0.11 |

| | | | | | | | | | | |
|---|---|---|---|---|---|---|---|---|---|---|
| 60 | E01 | NA | neg | NA | 262128 | 281861 | 1.07 | 1.12 | -7.84 | -10.39 |
| 60 | E02 | NA | neg | NA | 205805 | 224835 | 0.84 | 0.89 | 1.39 | -0.91 |
| 60 | E03 | 642 | BLMH | NM_000386 | 207579 | 228535 | 0.85 | 0.91 | 1.95 | -0.22 |
| 60 | E04 | 645 | BLVRB | NM_000713 | 223650 | NA | 0.92 | NA | -0.61 | NA |
| 60 | E05 | 8548 | BLZF1 | NM_003666 | 219852 | 216057 | 0.90 | 0.86 | -0.15 | 1.85 |
| 60 | E06 | 55839 | BM039 | NM_018455 | 224484 | NA | 0.92 | NA | -0.65 | NA |
| 60 | E07 | 662 | BNIP1 | NM_001205 | 190584 | 209725 | 0.78 | 0.83 | 3.44 | 1.49 |
| 60 | E08 | 665 | BNIP3L | NM_004331 | 196091 | 221262 | 0.80 | 0.88 | 1.91 | -0.20 |
| 60 | E09 | 666 | BOK | NM_032515 | 189643 | 206138 | 0.78 | 0.82 | 1.53 | 0.81 |
| 60 | E10 | 669 | BPGM | NM_001724 | 212187 | 207635 | 0.87 | 0.82 | -0.62 | -0.10 |
| 60 | E11 | 671 | BPI | NM_001725 | 206224 | 213782 | 0.84 | 0.85 | -1.66 | -0.86 |
| 60 | E12 | 10380 | BPNT1 | NM_006085 | 216075 | 217439 | 0.88 | 0.86 | 0.15 | 0.11 |
| 60 | F01 | NA | NA | NA | 236685 | 252589 | 0.97 | 1.00 | -4.77 | -6.32 |
| 60 | F02 | NA | pos | NA | 65474 | 178752 | 0.27 | 0.71 | 23.30 | 5.96 |
| 60 | F03 | 8019 | BRD3 | NM_007371 | 216328 | 232719 | 0.89 | 0.92 | -0.58 | -1.71 |
| 60 | F04 | 23476 | BRD4 | NM_014299 | 209538 | 223539 | 0.86 | 0.89 | 0.61 | -0.91 |
| 60 | F05 | 29117 | BRD7 | NM_013263 | 211351 | 225268 | 0.87 | 0.89 | 0.15 | -0.47 |
| 60 | F06 | 26228 | BRDG1 | NM_012108 | 213823 | 217727 | 0.88 | 0.86 | 0.00 | -0.13 |
| 60 | F07 | 140707 | BRI3BP | NM_080626 | 206006 | 217541 | 0.84 | 0.86 | -0.18 | -0.60 |
| 60 | F08 | 26580 | BSCL2 | NM_032667 | 199590 | 214528 | 0.82 | 0.85 | 0.24 | 0.13 |
| 60 | F09 | 7809 | BSND | NM_057176 | 189807 | 204025 | 0.78 | 0.81 | 0.41 | 0.37 |
| 60 | F10 | 686 | BTD | NM_000060 | 201743 | 192265 | 0.83 | 0.76 | 0.00 | 1.66 |
| 60 | F11 | 689 | BTF3 | NM_001207 | 194818 | 196858 | 0.80 | 0.78 | -0.88 | 1.16 |
| 60 | F12 | 10950 | BTG3 | NM_006806 | 212432 | 210585 | 0.87 | 0.83 | -0.35 | 0.46 |
| 60 | G01 | NA | neg | NA | 245033 | 258989 | 1.00 | 1.03 | -6.22 | -8.48 |
| 60 | G02 | NA | neg | NA | 220127 | 223569 | 0.90 | 0.89 | -2.14 | -2.59 |
| 60 | G03 | 54766 | BTG4 | NM_017589 | 212540 | 219915 | 0.87 | 0.87 | -0.05 | -0.68 |
| 60 | G04 | 9184 | BUB3 | NM_004725 | 199562 | 211460 | 0.82 | 0.84 | 2.16 | 0.00 |
| 60 | G05 | 118924 | C10ORF4 | NM_145246 | 223510 | 213030 | 0.92 | 0.84 | -1.93 | 0.47 |
| 60 | G06 | 8872 | C10ORF7 | NM_006023 | 229036 | 210356 | 0.94 | 0.83 | -2.58 | 0.00 |
| 60 | G07 | 219771 | C10ORF9 | NM_145012 | 203948 | 215075 | 0.84 | 0.85 | 0.07 | -1.29 |
| 60 | G08 | 745 | C11ORF9 | NM_013279 | 216732 | 206154 | 0.89 | 0.82 | -2.65 | 0.42 |
| 60 | G09 | 221150 | C13ORF3 | NM_145061 | 179772 | 185326 | 0.74 | 0.73 | 1.97 | 2.38 |
| 60 | G10 | 84520 | C14ORF142 | NM_032490 | 201201 | 191945 | 0.82 | 0.76 | 0.00 | 0.62 |
| 60 | G11 | 10712 | C1ORF2 | NM_006589 | 186939 | 197600 | 0.77 | 0.78 | 0.32 | -0.06 |
| 60 | G12 | 79577 | HRPT2 | NM_024529 | 209794 | 215607 | 0.86 | 0.85 | 0.00 | -1.47 |
| 60 | H01 | NA | NA | NA | NA | 211220 | NA | 0.84 | NA | 0.82 |
| 60 | H02 | NA | pos | NA | 62726 | 130756 | 0.26 | 0.52 | 25.67 | 14.20 |
| 60 | H03 | 712 | C1QA | NM_015991 | 231606 | 210497 | 0.95 | 0.83 | -1.16 | 2.24 |
| 60 | H04 | 713 | C1QB | NM_000491 | 229557 | 217744 | 0.94 | 0.86 | -0.74 | 0.31 |
| 60 | H05 | 714 | C1QG | NM_172369 | 226998 | 227052 | 0.93 | 0.90 | -0.49 | -0.51 |
| 60 | H06 | 24141 | C20ORF103 | NM_012261 | 221916 | 215423 | 0.91 | 0.85 | 0.60 | 0.51 |
| 60 | H07 | 25876 | C20ORF28 | NM_015417 | 218060 | 217010 | 0.89 | 0.86 | -0.23 | -0.25 |
| 60 | H08 | 55245 | C20ORF44 | NM_018244 | 209505 | 213335 | 0.86 | 0.85 | 0.55 | 0.59 |
| 60 | H09 | 55321 | C20ORF46 | NM_018354 | 206595 | 212550 | 0.85 | 0.84 | -0.41 | -0.79 |
| 60 | H10 | 54014 | C21ORF107 | NM_018963 | 212091 | 214208 | 0.87 | 0.85 | 0.23 | -1.73 |
| 60 | H11 | 51374 | C2ORF28 | NM_016085 | 199571 | 211934 | 0.82 | 0.84 | 0.27 | -1.08 |
| 60 | H12 | 10138 | YAF2 | NM_005748 | 199238 | 213337 | 0.82 | 0.85 | 3.74 | 0.26 |
| 61 | A01 | NA | pos | NA | 66448 | 80767 | 0.22 | 0.26 | 33.93 | 36.74 |
| 61 | A02 | NA | NA | NA | 269508 | 316088 | 0.90 | 1.03 | 1.02 | -5.24 |
| 61 | A03 | 721 | C4B | NM_000592 | 273473 | 285371 | 0.92 | 0.93 | 0.79 | 0.33 |
| 61 | A04 | 729 | C6 | NM_000065 | 284967 | 312387 | 0.95 | 1.01 | -0.17 | -2.46 |
| 61 | A05 | 910 | CD1B | NM_001764 | 289038 | 283237 | 0.97 | 0.92 | -0.62 | 1.57 |
| 61 | A06 | 911 | CD1C | NM_001765 | 275822 | 296303 | 0.92 | 0.96 | 0.20 | -1.70 |
| 61 | A07 | 934 | CD24 | NM_013230 | 294830 | 292588 | 0.99 | 0.95 | -3.24 | -1.06 |
| 61 | A08 | 51744 | CD244 | NM_016382 | 285404 | 283255 | 0.96 | 0.92 | -1.90 | -0.33 |
| 61 | A09 | 23607 | CD2AP | NM_012120 | 253997 | 279418 | 0.85 | 0.91 | 2.94 | 0.71 |
| 61 | A10 | 10421 | CD2BP2 | NM_006110 | 267099 | 272753 | 0.89 | 0.89 | 0.57 | 0.55 |
| 61 | A11 | 951 | CD37 | NM_001774 | 281278 | 282322 | 0.94 | 0.92 | -2.80 | -1.58 |
| 61 | A12 | 963 | CD53 | NM_000560 | 273058 | 285724 | 0.91 | 0.93 | 1.32 | 0.39 |
| 61 | B01 | NA | neg | NA | 301751 | 313998 | 1.01 | 1.02 | -4.26 | -4.93 |
| 61 | B02 | NA | neg | NA | 285754 | 285957 | 0.96 | 0.93 | -1.67 | 0.08 |
| 61 | B03 | 966 | CD59 | NM_000611 | 278359 | 287206 | 0.93 | 0.93 | -0.06 | -0.05 |
| 61 | B04 | 967 | CD63 | NM_001780 | 277846 | 290873 | 0.93 | 0.94 | 0.93 | 1.33 |
| 61 | B05 | 968 | CD68 | NM_001251 | 275464 | 287675 | 0.92 | 0.93 | 1.52 | 0.73 |
| 61 | B06 | 9308 | CD83 | NM_004233 | 282096 | 286168 | 0.94 | 0.93 | -0.88 | 0.05 |
| 61 | B07 | 8832 | CD84 | NM_003874 | 282447 | 287505 | 0.95 | 0.93 | -1.30 | -0.21 |
| 61 | B08 | 4267 | CD99 | NM_002414 | 273123 | 284479 | 0.91 | 0.92 | 0.03 | -0.60 |
| 61 | B09 | 146059 | CDAN1 | NM_138477 | 264129 | 298446 | 0.88 | 0.97 | 1.23 | -2.74 |
| 61 | B10 | 8881 | CDC16 | NM_003903 | 272917 | 281791 | 0.91 | 0.91 | -0.44 | -1.12 |
| 61 | B11 | 991 | CDC20 | NM_001255 | 262131 | 269173 | 0.88 | 0.87 | 0.24 | 0.71 |
| 61 | B12 | 246184 | CDC26 | NM_139286 | 285381 | 287119 | 0.96 | 0.93 | -0.74 | 0.09 |
| 61 | C01 | NA | pos | NA | 74965 | 116779 | 0.25 | 0.38 | 32.31 | 30.20 |
| 61 | C02 | NA | NA | NA | 242909 | 249748 | 0.81 | 0.81 | 5.10 | 6.48 |
| 61 | C03 | 996 | CDC27 | NM_001256 | 276527 | 286309 | 0.93 | 0.93 | 0.06 | 0.06 |
| 61 | C04 | 998 | CDC42 | NM_001791 | 295926 | 283450 | 0.99 | 0.92 | -2.18 | 2.60 |
| 61 | C05 | 11135 | CDC42EP1 | NM_007061 | 272175 | 291476 | 0.91 | 0.95 | 1.87 | 0.00 |
| 61 | C06 | 8318 | CDC45L | NM_003504 | 276108 | 286143 | 0.92 | 0.93 | -0.09 | 0.00 |
| 61 | C07 | 128869 | CDC91L1 | NM_080476 | 268787 | 278534 | 0.90 | 0.90 | 0.74 | 1.34 |
| 61 | C08 | 83540 | CDCA1 | NM_031423 | 248115 | 249476 | 0.83 | 0.81 | 3.91 | 5.59 |
| 61 | C09 | 55038 | CDCA4 | NM_017955 | 267918 | 291300 | 0.90 | 0.95 | 0.44 | -1.52 |
| 61 | C10 | 83879 | CDCA7 | NM_031942 | 273269 | 280617 | 0.91 | 0.91 | -0.67 | -0.96 |
| 61 | C11 | 1001 | CDH3 | NM_001793 | 263108 | 279847 | 0.88 | 0.91 | -0.09 | -1.24 |
| 61 | C12 | 1029 | CDKN2A | NM_000077 | 298608 | 290497 | 1.00 | 0.94 | -3.06 | -0.57 |
| 61 | D01 | NA | neg | NA | 325065 | 314554 | 1.09 | 1.02 | -8.39 | -6.56 |
| 61 | D02 | NA | neg | NA | 296239 | 302328 | 0.99 | 0.98 | -3.72 | -4.37 |
| 61 | D03 | 50937 | CDON | NM_016952 | 293093 | 302335 | 0.98 | 0.98 | -2.79 | -4.28 |
| 61 | D04 | 1038 | CDR1 | NM_004065 | 283166 | 290791 | 0.95 | 0.94 | -0.28 | -0.19 |

| | | | | | | | | | | |
|---|---|---|---|---|---|---|---|---|---|---|
| 61 | D05 | 1039 | CDR2 | XM_071866 | 279882 | 288357 | 0.94 | 0.94 | 0.46 | -0.93 |
| 61 | D06 | 1041 | CDSN | NM_001264 | 274983 | 276748 | 0.92 | 0.90 | -0.08 | 0.20 |
| 61 | D07 | 10218 | CDT6 | NM_021146 | 277733 | 267825 | 0.93 | 0.87 | -0.88 | 1.77 |
| 61 | D08 | 1087 | CEACAM7 | NM_006890 | 270621 | 282664 | 0.91 | 0.92 | 0.09 | -1.81 |
| 61 | D09 | 1053 | CEBPE | NM_001805 | 258568 | 259048 | 0.87 | 0.84 | 1.79 | 2.76 |
| 61 | D10 | 51148 | CEECAM1 | NM_016174 | 259453 | 264306 | 0.87 | 0.86 | 1.40 | 0.47 |
| 61 | D11 | 1056 | CEL | NM_001807 | 259554 | 263393 | 0.87 | 0.85 | 0.31 | 0.21 |
| 61 | D12 | 1063 | CENPF | NM_016343 | 285776 | 296950 | 0.96 | 0.96 | -1.15 | -3.20 |
| 61 | E01 | NA | neg | NA | 305596 | 319116 | 1.02 | 1.04 | -4.92 | -7.48 |
| 61 | E02 | NA | neg | NA | 299444 | 278654 | 1.00 | 0.90 | -3.92 | -0.27 |
| 61 | E03 | 64946 | CENPH | NM_022909 | 277250 | 277407 | 0.93 | 0.90 | 0.09 | 0.05 |
| 61 | E04 | 55835 | CENPJ | NM_018451 | 261951 | 289316 | 0.88 | 0.94 | 3.47 | -0.04 |
| 61 | E05 | 11064 | CEP1 | NM_007018 | 287749 | 279744 | 0.96 | 0.91 | -0.51 | 0.49 |
| 61 | E06 | 8824 | CES2 | NM_003869 | 270910 | 295647 | 0.91 | 0.96 | 0.90 | -3.29 |
| 61 | E07 | 90634 | CG018 | NM_052818 | 263336 | 272560 | 0.88 | 0.88 | 1.77 | 0.81 |
| 61 | E08 | 1082 | CGB | NM_000737 | 267674 | 273415 | 0.90 | 0.89 | 0.88 | -0.27 |
| 61 | E09 | 93659 | CGB5 | NM_033043 | 278232 | 268134 | 0.93 | 0.87 | -1.08 | 1.02 |
| 61 | E10 | 94115 | CGB8 | NM_033183 | 276557 | 290560 | 0.93 | 0.94 | -1.06 | -4.33 |
| 61 | E11 | 1105 | CHD1 | NM_001270 | 264156 | 267571 | 0.88 | 0.87 | -0.12 | -0.65 |
| 61 | E12 | 80205 | CHD9 | NM_025134 | 284216 | 275099 | 0.95 | 0.89 | -0.58 | 0.58 |
| 61 | F01 | NA | NA | NA | 294175 | 319407 | 0.98 | 1.04 | -3.59 | -7.15 |
| 61 | F02 | NA | pos | NA | 102027 | 199024 | 0.34 | 0.65 | 27.55 | 14.33 |
| 61 | F03 | 26511 | CHIC2 | NM_012110 | 291273 | 301073 | 0.97 | 0.98 | -2.71 | -3.78 |
| 61 | F04 | 89832 | CHRFAM7A | NM_139320 | 274661 | 295873 | 0.92 | 0.96 | 0.89 | -0.82 |
| 61 | F05 | 1143 | CHRNB4 | NM_000750 | 284203 | 284677 | 0.95 | 0.92 | -0.46 | 0.00 |
| 61 | F06 | 1146 | CHRNG | NM_005199 | 272750 | 279437 | 0.91 | 0.91 | 0.08 | 0.00 |
| 61 | F07 | 1130 | CHS1 | NM_000081 | 268950 | 281001 | 0.90 | 0.91 | 0.33 | -0.31 |
| 61 | F08 | 9391 | CIAO1 | NM_004804 | 272495 | 272519 | 0.91 | 0.88 | -0.42 | 0.27 |
| 61 | F09 | 114548 | CIAS1 | NM_004895 | 271038 | 269007 | 0.91 | 0.87 | -0.44 | 1.26 |
| 61 | F10 | 91543 | CIG5 | NM_080657 | 267036 | 271114 | 0.89 | 0.88 | -0.04 | -0.47 |
| 61 | F11 | 84916 | CIRH1A | NM_032830 | 248249 | 257336 | 0.83 | 0.84 | 1.94 | 1.57 |
| 61 | F12 | 1154 | CISH | NM_013324 | 273821 | 280419 | 0.92 | 0.91 | 0.58 | 0.02 |
| 61 | G01 | NA | neg | NA | 272115 | 283732 | 0.91 | 0.92 | -0.48 | -1.72 |
| 61 | G02 | NA | neg | NA | 298128 | 317525 | 1.00 | 1.03 | -4.70 | -7.75 |
| 61 | G03 | 23122 | CLASP2 | XM_291057 | 287605 | 285617 | 0.96 | 0.93 | -2.58 | -1.96 |
| 61 | G04 | 1188 | CLCNKB | NM_000085 | 277028 | 285772 | 0.93 | 0.93 | 0.04 | 0.04 |
| 61 | G05 | 51267 | CLEC1 | NM_016511 | 282634 | 283288 | 0.95 | 0.92 | -0.67 | -0.69 |
| 61 | G06 | 64857 | PLEKHG2 | NM_022835 | 284363 | 270734 | 0.95 | 0.88 | -2.27 | 0.61 |
| 61 | G07 | 9022 | CLIC3 | NM_004669 | 263526 | 288630 | 0.88 | 0.94 | 0.75 | -2.60 |
| 61 | G08 | 25999 | CLIPR-59 | NM_015526 | 267204 | 258323 | 0.89 | 0.84 | -0.03 | 1.87 |
| 61 | G09 | 1201 | CLN3 | NM_000086 | 270778 | 275606 | 0.91 | 0.89 | -0.87 | -0.86 |
| 61 | G10 | 1203 | CLN5 | NM_006493 | 248306 | 242960 | 0.83 | 0.79 | 2.53 | 3.62 |
| 61 | G11 | 54982 | CLN6 | NM_017882 | 256761 | 255182 | 0.86 | 0.83 | 0.09 | 1.02 |
| 61 | G12 | 2055 | CLN8 | NM_018941 | 266137 | 275444 | 0.89 | 0.89 | 1.36 | -0.02 |
| 61 | H01 | NA | NA | NA | 245100 | 244131 | 0.82 | 0.79 | 4.92 | 7.65 |
| 61 | H02 | NA | pos | NA | 77007 | 123743 | 0.26 | 0.40 | 32.16 | 29.12 |
| 61 | H03 | 1209 | CLPTM1 | NM_001294 | 268280 | 282512 | 0.90 | 0.92 | 1.58 | 0.89 |
| 61 | H04 | 22883 | CLSTN1 | NM_014944 | 283838 | 298065 | 0.95 | 0.97 | -0.04 | 0.15 |
| 61 | H05 | 1191 | CLU | NM_001831 | 280425 | 293145 | 0.94 | 0.95 | 0.72 | -0.14 |
| 61 | H06 | 1259 | CNGA1 | NM_000087 | 272941 | 300796 | 0.91 | 0.98 | 0.61 | -2.45 |
| 61 | H07 | 54714 | CNGB3 | NM_019098 | 276511 | 285762 | 0.93 | 0.93 | -0.33 | 0.21 |
| 61 | H08 | 26507 | CNNM1 | NM_020348 | 273538 | 273308 | 0.92 | 0.89 | -0.03 | 1.50 |
| 61 | H09 | 54805 | CNNM2 | NM_017649 | 277119 | 287676 | 0.93 | 0.93 | -0.87 | -0.71 |
| 61 | H10 | 26505 | CNNM3 | NM_017623 | 269990 | 272050 | 0.90 | 0.88 | 0.04 | 0.73 |
| 61 | H11 | 246175 | CNOT6L | NM_144571 | 274955 | 274985 | 0.92 | 0.89 | -1.83 | -0.21 |
| 61 | H12 | 53942 | CNTN5 | NM_014361 | 274182 | 290498 | 0.92 | 0.94 | 1.08 | -0.41 |
| 62 | A01 | NA | pos | NA | 43330 | 90621 | 0.15 | 0.31 | 27.61 | 28.99 |
| 62 | A02 | NA | NA | NA | 332949 | 320034 | 1.19 | 1.08 | -2.75 | -2.19 |
| 62 | A03 | 27255 | CNTN6 | NM_014461 | 345982 | 342817 | 1.24 | 1.16 | -4.06 | -4.40 |
| 62 | A04 | 8506 | CNTNAP1 | NM_003632 | 321634 | 316818 | 1.15 | 1.07 | -1.01 | -0.99 |
| 62 | A05 | 200025 | COAS3 | AF345651 | 319204 | 345274 | 1.14 | 1.16 | 0.88 | -5.06 |
| 62 | A06 | 1690 | COCH | NM_004086 | 328726 | 332770 | 1.18 | 1.12 | -0.86 | -3.64 |
| 62 | A07 | 1306 | COL15A1 | NM_001855 | 335703 | 335743 | 1.20 | 1.13 | -2.13 | -5.13 |
| 62 | A08 | 1280 | COL2A1 | NM_001844 | 284965 | 295905 | 1.02 | 1.00 | 2.22 | 0.99 |
| 62 | A09 | 1295 | COL8A1 | NM_001850 | 296927 | 284044 | 1.06 | 0.96 | 0.61 | 2.12 |
| 62 | A10 | 8292 | COLQ | NM_005677 | 296147 | 268420 | 1.06 | 0.90 | 0.02 | 3.84 |
| 62 | A11 | 8533 | COPS3 | NM_003653 | 290973 | 287189 | 1.04 | 0.97 | -0.02 | 1.07 |
| 62 | A12 | 10987 | COPS5 | NM_006837 | 298729 | 296937 | 1.07 | 1.00 | 0.21 | 1.05 |
| 62 | B01 | NA | neg | NA | 270091 | 312160 | 0.97 | 1.05 | 2.49 | -3.24 |
| 62 | B02 | NA | neg | NA | 294731 | 302781 | 1.05 | 1.02 | -0.10 | -1.96 |
| 62 | B03 | 10063 | COX17 | NM_005694 | 281751 | 288480 | 1.01 | 0.97 | 1.33 | 0.87 |
| 62 | B04 | 1370 | CPN2 | J05158 | 304474 | 292885 | 1.09 | 0.99 | -0.57 | 0.15 |
| 62 | B05 | 57699 | CPNE5 | NM_020939 | 321370 | 297348 | 1.15 | 1.00 | -0.69 | -0.66 |
| 62 | B06 | 1371 | CPOX | NM_000097 | 279109 | 294543 | 1.00 | 0.99 | 2.99 | -0.56 |
| 62 | B07 | 9238 | TBRG4 | NM_004749 | 307165 | 304893 | 1.10 | 1.03 | -0.49 | -3.06 |
| 62 | B08 | 9236 | CPR8 | NM_004748 | 302757 | 287799 | 1.08 | 0.97 | -1.00 | -0.03 |
| 62 | B09 | 167 | CRISP1 | NM_001131 | 289839 | 281769 | 1.04 | 0.95 | 0.00 | 0.32 |
| 62 | B10 | 9244 | CRLF1 | NM_004750 | 283473 | 292329 | 1.01 | 0.98 | 0.00 | -1.52 |
| 62 | B11 | 51379 | CRLF3 | NM_015986 | 277657 | 275535 | 0.99 | 0.93 | 0.02 | 0.54 |
| 62 | B12 | 10485 | CROC4 | NM_006365 | 287088 | 288911 | 1.03 | 0.97 | 0.08 | 0.03 |
| 62 | C01 | NA | pos | NA | 52192 | 115924 | 0.19 | 0.39 | 25.48 | 22.44 |
| 62 | C02 | NA | NA | NA | 234145 | 251284 | 0.84 | 0.85 | 6.41 | 4.04 |
| 62 | C03 | 9439 | CRSP3 | NM_004830 | 283693 | 286879 | 1.01 | 0.97 | 1.28 | 0.09 |
| 62 | C04 | 9442 | CRSP8 | NM_004269 | 284690 | 309747 | 1.02 | 1.04 | 1.66 | -3.14 |
| 62 | C05 | 56253 | CRTAM | NM_019604 | 316228 | 271909 | 1.13 | 0.92 | 0.00 | 1.80 |
| 62 | C06 | 1413 | CRYBA4 | NM_001886 | 349241 | 277524 | 1.25 | 0.94 | -4.21 | 0.75 |
| 62 | C07 | 1414 | CRYBB1 | NM_001887 | 279837 | 274140 | 1.00 | 0.92 | 2.53 | 0.13 |
| 62 | C08 | 1415 | CRYBB2 | NM_000496 | 276031 | 299044 | 0.99 | 1.01 | 1.96 | -2.55 |

| | | | | | | | | | | |
|---|---|---|---|---|---|---|---|---|---|---|
| 62 | C09 | 1418 | CRYGA | NM_014617 | 294856 | 275069 | 1.05 | 0.93 | -0.37 | 0.23 |
| 62 | C10 | 1421 | CRYGD | NM_006891 | 292821 | 274463 | 1.05 | 0.92 | -0.83 | -0.09 |
| 62 | C11 | 1427 | CRYGS | NM_017541 | 281801 | 286840 | 1.01 | 0.97 | -0.26 | -1.99 |
| 62 | C12 | 55997 | CFC1 | NM_032545 | 289324 | 311740 | 1.03 | 1.05 | 0.00 | -4.07 |
| 62 | D01 | NA | neg | NA | 247478 | 317003 | 0.88 | 1.07 | 4.70 | -3.14 |
| 62 | D02 | NA | neg | NA | 304617 | 286752 | 1.09 | 0.97 | -1.29 | 0.97 |
| 62 | D03 | 158511 | CSAG1 | NM_153478 | 289697 | 300908 | 1.04 | 1.01 | 0.34 | -0.06 |
| 62 | D04 | 1442 | CSH1 | NM_001317 | 286591 | 305210 | 1.02 | 1.03 | 1.16 | -0.77 |
| 62 | D05 | 1465 | CSRP1 | NM_004078 | 313309 | 294362 | 1.12 | 0.99 | 0.00 | 0.50 |
| 62 | D06 | 8048 | CSRP3 | NM_003476 | 296582 | 283899 | 1.06 | 0.96 | 1.00 | 1.64 |
| 62 | D07 | 1471 | CST3 | NM_000099 | 291556 | 284740 | 1.04 | 0.96 | 0.99 | 0.44 |
| 62 | D08 | 8530 | CST7 | NM_003650 | 291852 | 293181 | 1.04 | 0.99 | -0.01 | -0.01 |
| 62 | D09 | 1493 | CTLA4 | NM_005214 | 301192 | 296393 | 1.08 | 1.00 | -1.34 | -0.92 |
| 62 | D10 | 1495 | CTNNA1 | NM_001903 | 281996 | 275951 | 1.01 | 0.93 | 0.00 | 1.45 |
| 62 | D11 | 8727 | CTNNAL1 | NM_003798 | 278398 | 289702 | 1.00 | 0.98 | -0.21 | -0.63 |
| 62 | D12 | 56259 | CTNNBL1 | NM_030877 | 301446 | 294613 | 1.08 | 0.99 | -1.58 | 0.01 |
| 62 | E01 | NA | neg | NA | 249922 | 311065 | 0.89 | 1.05 | 3.90 | -4.37 |
| 62 | E02 | NA | neg | NA | 293594 | 288071 | 1.05 | 0.97 | -0.68 | -1.24 |
| 62 | E03 | 1513 | CTSK | NM_000396 | 285294 | 282560 | 1.02 | 0.95 | 0.25 | 0.39 |
| 62 | E04 | 23584 | CTXL | NM_014312 | 277473 | 267856 | 0.99 | 0.90 | 1.56 | 2.27 |
| 62 | E05 | 8065 | CUL5 | NM_003478 | 286389 | 304031 | 1.02 | 1.02 | 2.27 | -2.85 |
| 62 | E06 | 6374 | CXCL5 | NM_002994 | 309443 | 276823 | 1.11 | 0.93 | -0.90 | 0.56 |
| 62 | E07 | 1528 | CYB5 | NM_001914 | 279781 | 270175 | 1.00 | 0.91 | 1.67 | 0.38 |
| 62 | E08 | 1536 | CYBB | NM_000397 | 288882 | 272497 | 1.03 | 0.92 | -0.25 | 0.77 |
| 62 | E09 | 54205 | CYCS | NM_018947 | 268993 | 279631 | 0.96 | 0.94 | 1.48 | -0.68 |
| 62 | E10 | 7461 | CYLN2 | NM_003388 | 281841 | 274498 | 1.01 | 0.92 | -0.53 | -0.38 |
| 62 | E11 | 1583 | CYP11A1 | NM_000781 | 278229 | 274250 | 0.99 | 0.92 | -0.74 | -0.56 |
| 62 | E12 | 1544 | CYP1A2 | NM_000761 | 284972 | 297722 | 1.02 | 1.00 | -0.40 | -2.45 |
| 62 | F01 | NA | NA | NA | 213533 | 311162 | 0.76 | 1.05 | 7.08 | -3.88 |
| 62 | F02 | NA | pos | NA | 102677 | 200570 | 0.37 | 0.68 | 18.70 | 11.15 |
| 62 | F03 | 23002 | DAAM1 | NM_014992 | 284102 | 288687 | 1.02 | 0.97 | -0.25 | 0.06 |
| 62 | F04 | 1600 | DAB1 | NM_021080 | 291531 | 289393 | 1.04 | 0.98 | -0.54 | -0.15 |
| 62 | F05 | 1601 | DAB2 | NM_001343 | 282455 | 288524 | 1.01 | 0.97 | 2.06 | -0.24 |
| 62 | F06 | 51339 | DACT1 | NM_016651 | 294636 | 274245 | 1.05 | 0.92 | 0.03 | 1.42 |
| 62 | F07 | 1603 | DAD1 | NM_001344 | 289835 | 276633 | 1.04 | 0.93 | -0.01 | 0.00 |
| 62 | F08 | 1604 | DAF | NM_000574 | 280467 | 275632 | 1.00 | 0.93 | 0.01 | 0.85 |
| 62 | F09 | 1611 | DAP | NM_004394 | 323029 | 284842 | 1.15 | 0.96 | -4.81 | -0.88 |
| 62 | F10 | 55885 | LMO3 | NM_001001395 | 283628 | 274674 | 1.01 | 0.93 | -1.35 | 0.09 |
| 62 | F11 | 57055 | DAZ2 | NM_020363 | 263806 | 277794 | 0.94 | 0.94 | 0.14 | -0.54 |
| 62 | F12 | 57054 | DAZ3 | NM_020364 | 272763 | 283410 | 0.98 | 0.95 | 0.25 | -0.01 |
| 62 | G01 | NA | neg | NA | 213103 | 290829 | 0.76 | 0.98 | 7.11 | -1.06 |
| 62 | G02 | NA | neg | NA | 289318 | 287756 | 1.03 | 0.97 | -0.88 | -0.64 |
| 62 | G03 | 9802 | DAZAP2 | NM_014764 | 411122 | 294973 | 1.47 | 0.99 | -13.58 | -0.73 |
| 62 | G04 | 56942 | DC13 | NM_020188 | 281089 | 285032 | 1.00 | 0.96 | 0.54 | 0.50 |
| 62 | G05 | 64858 | DCLRE1B | NM_022836 | 307721 | 285415 | 1.10 | 0.96 | -0.61 | 0.24 |
| 62 | G06 | 64421 | DCLRE1C | NM_022487 | 284985 | 296342 | 1.02 | 1.00 | 1.03 | -1.53 |
| 62 | G07 | 1634 | DCN | NM_001920 | 300250 | 277123 | 1.07 | 0.93 | -1.11 | 0.00 |
| 62 | G08 | 10540 | DCTN2 | NM_006400 | 280362 | 282231 | 1.00 | 0.95 | 0.00 | 0.01 |
| 62 | G09 | 51164 | DCTN4 | NM_016221 | 277001 | 280457 | 0.99 | 0.94 | 0.00 | -0.23 |
| 62 | G10 | 92181 | DC-UBP | NM_152277 | 269302 | 282252 | 0.96 | 0.95 | 0.14 | -0.88 |
| 62 | G11 | 84722 | DDA3 | NM_032636 | 255755 | 267547 | 0.91 | 0.90 | 0.97 | 0.91 |
| 62 | G12 | 1642 | DDB1 | NM_001923 | 277902 | 276698 | 0.99 | 0.93 | -0.31 | 0.96 |
| 62 | H01 | NA | NA | NA | 158847 | 232886 | 0.57 | 0.78 | 13.94 | 6.89 |
| 62 | H02 | NA | pos | NA | 68061 | 146658 | 0.24 | 0.49 | 23.45 | 18.61 |
| 62 | H03 | 26512 | DDX26 | NM_012141 | 325346 | 295656 | 1.16 | 1.00 | -3.46 | -0.75 |
| 62 | H04 | 50514 | DEC1 | NM_017418 | 320064 | 287104 | 1.14 | 0.97 | -2.41 | 0.29 |
| 62 | H05 | 26297 | DELGEF | NM_012139 | 317897 | 278277 | 1.14 | 0.94 | -0.54 | 1.28 |
| 62 | H06 | 221120 | DEPC-1 | NM_139178 | 305890 | 294409 | 1.09 | 0.99 | -0.03 | -1.19 |
| 62 | H07 | 23587 | DERP6 | NM_015362 | 300394 | 284589 | 1.07 | 0.96 | 0.01 | -0.95 |
| 62 | H08 | 1674 | DES | NM_001927 | 291210 | 284940 | 1.04 | 0.96 | 0.00 | -0.29 |
| 62 | H09 | 28983 | DESC1 | NM_014058 | 269907 | 273863 | 0.96 | 0.92 | 1.88 | 0.74 |
| 62 | H10 | 1687 | DFNA5 | NM_004403 | 264135 | 254009 | 0.94 | 0.86 | 1.82 | 3.03 |
| 62 | H11 | 8214 | DGCR6 | NM_005675 | 266378 | 249414 | 0.95 | 0.84 | 0.99 | 3.44 |
| 62 | H12 | 1720 | DHFRP1 | J00146 | 285807 | 287859 | 1.02 | 0.97 | 0.00 | -0.48 |
| 63 | A01 | NA | pos | NA | 61984 | 81090 | 0.21 | 0.27 | 28.79 | 31.99 |
| 63 | A02 | NA | NA | NA | 307939 | 307249 | 1.02 | 1.02 | -2.22 | -2.17 |
| 63 | A03 | 56616 | DIABLO | NM_019887 | 317613 | 315848 | 1.05 | 1.05 | -1.57 | -3.05 |
| 63 | A04 | 1730 | DIAPH2 | NM_006729 | 332072 | 302936 | 1.10 | 1.00 | -4.41 | -1.17 |
| 63 | A05 | 81624 | DIAPH3 | NM_030932 | 289984 | 284249 | 0.96 | 0.94 | 0.05 | 1.67 |
| 63 | A06 | 84925 | DIRC2 | NM_032839 | 281614 | 277848 | 0.93 | 0.92 | 2.01 | 2.71 |
| 63 | A07 | 27185 | DISC1 | NM_018662 | 288281 | 293098 | 0.96 | 0.97 | -0.05 | -0.35 |
| 63 | A08 | 253190 | DJ222E13.1 | NM_014509 | 277484 | 277265 | 0.92 | 0.92 | 1.61 | 0.85 |
| 63 | A09 | 1736 | DKC1 | NM_001363 | 284223 | 288058 | 0.94 | 0.95 | 0.24 | -0.69 |
| 63 | A10 | 25896 | DKFZP434B168 | NM_015434 | 296046 | 306108 | 0.98 | 1.01 | -1.33 | -3.58 |
| 63 | A11 | 26084 | SGEF | NM_015595 | 301909 | 280221 | 1.00 | 0.93 | -1.60 | 0.33 |
| 63 | A12 | 151313 | DKFZP434N062 | NM_199336 | 289819 | 290051 | 0.96 | 0.96 | 1.19 | 1.43 |
| 63 | B01 | NA | neg | NA | 306709 | 308503 | 1.02 | 1.02 | -2.95 | -3.51 |
| 63 | B02 | NA | neg | NA | 286278 | 294598 | 0.95 | 0.98 | -0.38 | -1.41 |
| 63 | B03 | 200081 | DKFZP451J011 | NM_175852 | 278627 | 275327 | 0.92 | 0.91 | 2.46 | 1.92 |
| 63 | B04 | 84242 | DKFZP547D155 | NM_032282 | 290276 | 290892 | 0.96 | 0.96 | -0.03 | -0.51 |
| 63 | B05 | 84064 | DKFZP564D137 | NM_032124 | 283481 | 288267 | 0.94 | 0.95 | -0.02 | -0.09 |
| 63 | B06 | 25845 | DKFZP564I117 | AL117555 | 295436 | 286055 | 0.98 | 0.95 | -0.63 | 0.32 |
| 63 | B07 | 26022 | DKFZP564K196 | NM_015544 | 279576 | 284271 | 0.93 | 0.94 | 0.16 | -0.17 |
| 63 | B08 | 4086 | SMAD1 | NM_005900 | 283117 | 275711 | 0.94 | 0.91 | 0.01 | -0.07 |
| 63 | B09 | 56243 | KIAA1217 | NM_019590 | 282348 | 275614 | 0.94 | 0.91 | -0.41 | 0.04 |
| 63 | B10 | 6687 | SPG7 | NM_003119 | 280898 | 272299 | 0.93 | 0.90 | -0.31 | 0.38 |
| 63 | B11 | 79747 | C6orf103 | XM_371850 | 267829 | 276079 | 0.89 | 0.91 | 1.81 | -0.20 |
| 63 | B12 | 10591 | C6ORF108 | NM_006443 | 279904 | 278093 | 0.93 | 0.92 | 1.55 | 2.09 |

| | | | | | | | | | | |
|---|---|---|---|---|---|---|---|---|---|---|
| 63 | C01 | NA | pos | NA | 70469 | 84626 | 0.23 | 0.28 | 26.58 | 30.27 |
| 63 | C02 | NA | NA | NA | 241075 | 225823 | 0.80 | 0.75 | 5.08 | 8.95 |
| 63 | C03 | 57107 | C6ORF210 | NM_020381 | 316342 | 282498 | 1.05 | 0.94 | -2.54 | 0.81 |
| 63 | C04 | 730 | C7 | NM_000587 | 277914 | 281929 | 0.92 | 0.93 | 1.28 | 0.81 |
| 63 | C05 | 64327 | C7ORF2 | NM_022458 | 280261 | 289389 | 0.93 | 0.96 | 0.14 | -0.29 |
| 63 | C06 | 219285 | C7orf6 | NM_152703 | 283559 | 288470 | 0.94 | 0.96 | 0.63 | -0.07 |
| 63 | C07 | 731 | C8A | NM_000562 | 281766 | 273065 | 0.93 | 0.90 | -0.36 | 1.49 |
| 63 | C08 | 732 | C8B | NM_000066 | 263418 | 268624 | 0.87 | 0.89 | 2.25 | 0.97 |
| 63 | C09 | 735 | C9 | NM_001737 | 277938 | 290507 | 0.92 | 0.96 | -0.10 | -2.24 |
| 63 | C10 | 759 | CA1 | NM_001738 | 291661 | 305134 | 0.97 | 1.01 | -1.91 | -4.61 |
| 63 | C11 | 760 | CA2 | NM_000067 | 279282 | 279459 | 0.93 | 0.93 | 0.12 | -0.74 |
| 63 | C12 | 56997 | CABC1 | NM_020247 | 292820 | 291063 | 0.97 | 0.96 | -0.33 | 0.10 |
| 63 | D01 | NA | neg | NA | 315225 | 312174 | 1.05 | 1.03 | -4.13 | -3.42 |
| 63 | D02 | NA | neg | NA | 296163 | 300808 | 0.98 | 1.00 | -1.72 | -1.70 |
| 63 | D03 | 774 | CACNA1B | NM_000718 | 299147 | 297898 | 0.99 | 0.99 | -0.23 | -0.84 |
| 63 | D04 | 8913 | CACNA1G | NM_018896 | 281444 | 278964 | 0.93 | 0.92 | 0.98 | 1.94 |
| 63 | D05 | 779 | CACNA1S | NM_000069 | 282324 | 285678 | 0.94 | 0.95 | 0.02 | 0.95 |
| 63 | D06 | 785 | CACNB4 | NM_000726 | 282055 | 295036 | 0.94 | 0.98 | 0.96 | -0.39 |
| 63 | D07 | 793 | CALB1 | NM_004929 | 276004 | 292739 | 0.92 | 0.97 | 0.51 | -0.80 |
| 63 | D08 | 50632 | DRD1IP | NM_015722 | 282452 | 279066 | 0.94 | 0.92 | -0.01 | 0.07 |
| 63 | D09 | 820 | CAMP | NM_004345 | 278169 | 280796 | 0.92 | 0.93 | 0.01 | -0.10 |
| 63 | D10 | 23125 | CAMTA2 | NM_015099 | 280272 | 282216 | 0.93 | 0.93 | -0.33 | -0.47 |
| 63 | D11 | 10487 | CAP1 | NM_006367 | 282273 | 274948 | 0.94 | 0.91 | -0.12 | 0.62 |
| 63 | D12 | 830 | CAPZA2 | NM_006136 | 291924 | 291731 | 0.97 | 0.97 | -0.07 | 0.67 |
| 63 | E01 | NA | neg | NA | 293674 | 303096 | 0.97 | 1.00 | -2.55 | -4.14 |
| 63 | E02 | NA | neg | NA | 286426 | 280384 | 0.95 | 0.93 | -1.63 | -0.71 |
| 63 | E03 | 832 | CAPZB | NM_004930 | 290168 | 287755 | 0.96 | 0.95 | -0.24 | -1.40 |
| 63 | E04 | 9607 | CART | NM_004291 | 283498 | 277539 | 0.94 | 0.92 | -0.41 | 0.06 |
| 63 | E05 | 9994 | CASP8AP2 | NM_012115 | 258195 | 266989 | 0.86 | 0.88 | 1.93 | 1.68 |
| 63 | E06 | 26059 | CAST | NM_015576 | 291888 | 292124 | 0.97 | 0.97 | -1.42 | -2.04 |
| 63 | E07 | 117144 | CATSPER1 | NM_053054 | 269126 | 272459 | 0.89 | 0.90 | 0.24 | 0.17 |
| 63 | E08 | 858 | CAV2 | NM_001233 | 283123 | 264830 | 0.94 | 0.88 | -1.23 | 0.12 |
| 63 | E09 | 859 | CAV3 | NM_001234 | 257003 | 266551 | 0.85 | 0.88 | 1.54 | -0.04 |
| 63 | E10 | 10153 | CEBPZ | NM_005760 | 266174 | 251177 | 0.88 | 0.83 | 0.31 | 2.12 |
| 63 | E11 | 869 | CBLN1 | NM_004352 | 277959 | 282039 | 0.92 | 0.93 | -0.71 | -2.55 |
| 63 | E12 | 873 | CBR1 | NM_001757 | 278226 | 282976 | 0.92 | 0.94 | 0.52 | -0.10 |
| 63 | F01 | NA | NA | NA | 282640 | 298802 | 0.94 | 0.99 | -1.54 | -3.85 |
| 63 | F02 | NA | pos | NA | 83687 | 138062 | 0.28 | 0.46 | 23.54 | 20.43 |
| 63 | F03 | 874 | CBR3 | NM_001236 | 283439 | 275556 | 0.94 | 0.91 | 0.23 | 0.08 |
| 63 | F04 | 10951 | CBX1 | NM_006807 | 271621 | 269215 | 0.90 | 0.89 | 0.70 | 0.96 |
| 63 | F05 | 889 | CCM1 | NM_004912 | 272383 | 276345 | 0.90 | 0.92 | -0.24 | -0.09 |
| 63 | F06 | 8900 | CCNA1 | NM_003914 | 284187 | 279360 | 0.94 | 0.93 | -0.83 | -0.47 |
| 63 | F07 | 890 | CCNA2 | NM_001237 | 295609 | 263465 | 0.98 | 0.87 | -3.48 | 1.17 |
| 63 | F08 | 891 | CCNB1 | NM_031966 | 273888 | 264594 | 0.91 | 0.88 | -0.45 | -0.20 |
| 63 | F09 | 9133 | CCNB2 | NM_004701 | 255638 | 255374 | 0.85 | 0.85 | 1.33 | 1.29 |
| 63 | F10 | 85417 | CCNB3 | NM_033031 | 255241 | 265388 | 0.85 | 0.88 | 1.31 | -0.38 |
| 63 | F11 | 892 | CCNC | NM_005190 | 264869 | 261519 | 0.88 | 0.87 | 0.56 | 0.20 |
| 63 | F12 | 595 | CCND1 | NM_053056 | 281442 | 280978 | 0.93 | 0.93 | -0.27 | -0.15 |
| 63 | G01 | NA | neg | NA | 307921 | 305850 | 1.02 | 1.01 | -3.66 | -4.38 |
| 63 | G02 | NA | neg | NA | 311439 | 277616 | 1.03 | 0.92 | -4.10 | -0.12 |
| 63 | G03 | 894 | CCND2 | NM_001759 | 278457 | 271701 | 0.92 | 0.90 | 1.92 | 1.20 |
| 63 | G04 | 896 | CCND3 | NM_001760 | 286073 | 279504 | 0.95 | 0.93 | -0.05 | -0.06 |
| 63 | G05 | 23582 | CCNDBP1 | NM_012142 | 295791 | 278652 | 0.98 | 0.92 | -2.13 | 0.09 |
| 63 | G06 | 898 | CCNE1 | NM_001238 | 267335 | 245076 | 0.89 | 0.81 | 2.36 | 5.24 |
| 63 | G07 | 9134 | CCNE2 | NM_004702 | 293910 | 287910 | 0.98 | 0.95 | -2.20 | -1.99 |
| 63 | G08 | 899 | CCNF | NM_001761 | 277852 | 277698 | 0.92 | 0.92 | 0.12 | -1.64 |
| 63 | G09 | 900 | CCNG1 | NM_004060 | 280393 | 262015 | 0.93 | 0.87 | -0.72 | 0.82 |
| 63 | G10 | 901 | CCNG2 | NM_004354 | 268934 | 263459 | 0.89 | 0.87 | 0.64 | 0.44 |
| 63 | G11 | 902 | CCNH | NM_001239 | 282660 | 279818 | 0.94 | 0.93 | -0.62 | -2.04 |
| 63 | G12 | 10983 | CCNI | NM_006835 | 287202 | 286444 | 0.95 | 0.95 | 0.07 | -0.45 |
| 63 | H01 | NA | NA | NA | 262339 | 251012 | 0.87 | 0.83 | 2.33 | 6.26 |
| 63 | H02 | NA | pos | NA | 93159 | 89783 | 0.31 | 0.30 | 23.66 | 30.62 |
| 63 | H03 | 8812 | CCNK | NM_003858 | 288195 | 295820 | 0.96 | 0.98 | 0.94 | -0.08 |
| 63 | H04 | 57018 | CCNL1 | NM_020307 | 287389 | 296001 | 0.95 | 0.98 | 0.03 | -0.19 |
| 63 | H05 | 904 | CCNT1 | NM_001240 | 302700 | 302958 | 1.00 | 1.00 | -2.75 | -1.22 |
| 63 | H06 | 905 | CCNT2 | NM_058241 | 300303 | 294903 | 1.00 | 0.98 | -1.55 | 0.07 |
| 63 | H07 | 10575 | CCT4 | NM_006430 | 278030 | 276144 | 0.92 | 0.91 | 0.05 | 2.15 |
| 63 | H08 | 10693 | CCT6B | NM_006584 | 300408 | 293474 | 1.00 | 0.97 | -2.48 | -1.67 |
| 63 | H09 | 8763 | CD164 | NM_006016 | 276705 | 280377 | 0.92 | 0.93 | -0.01 | 0.41 |
| 63 | H10 | 909 | CD1A | NM_001763 | 268591 | 276104 | 0.89 | 0.91 | 0.93 | 0.89 |
| 63 | H11 | 114335 | CGB1 | NM_033377 | 275652 | 279326 | 0.91 | 0.93 | 0.51 | 0.40 |
| 63 | H12 | 114336 | CGB2 | NM_033378 | 309456 | 300314 | 1.03 | 0.99 | -2.49 | -0.18 |
| 64 | A01 | NA | pos | NA | 63573 | 103609 | 0.22 | 0.36 | 44.60 | 26.44 |
| 64 | A02 | NA | NA | NA | 241539 | 300966 | 0.84 | 1.03 | 8.82 | -1.57 |
| 64 | A03 | 55355 | KFZP762E131 | NM_018410 | 308920 | 320115 | 1.07 | 1.10 | -3.56 | -4.29 |
| 64 | A04 | 9940 | DLEC1 | NM_005106 | 274787 | 317502 | 0.95 | 1.09 | 2.34 | -3.90 |
| 64 | A05 | 8847 | DLEU2 | NM_006021 | 291666 | 344762 | 1.01 | 1.18 | -0.18 | -7.40 |
| 64 | A06 | 9787 | DLG7 | NM_014750 | 301797 | 290130 | 1.05 | 1.00 | -2.00 | 0.21 |
| 64 | A07 | 9229 | DLGAP1 | NM_004746 | 301900 | 279855 | 1.05 | 0.96 | -4.71 | 0.22 |
| 64 | A08 | 1762 | DMWD | XM_496318 | 275653 | 272899 | 0.96 | 0.94 | 1.19 | 1.27 |
| 64 | A09 | 85406 | DNAJC14 | NM_032364 | 275833 | 294603 | 0.96 | 1.01 | 1.72 | -0.52 |
| 64 | A10 | 10294 | DNAJA2 | NM_005880 | 280770 | 283329 | 0.97 | 0.97 | -0.03 | -0.22 |
| 64 | A11 | 55466 | DNAJA4 | NM_018602 | 278193 | 277538 | 0.96 | 0.95 | 0.01 | 1.31 |
| 64 | A12 | 116092 | DNTTIP1 | NM_052951 | 282868 | 282519 | 0.98 | 0.97 | 0.78 | 1.05 |
| 64 | B01 | NA | neg | NA | 283946 | 299065 | 0.98 | 1.03 | -1.48 | -3.24 |
| 64 | B02 | NA | neg | NA | 268117 | 282884 | 0.93 | 0.97 | 1.70 | -0.94 |
| 64 | B03 | 11259 | DOC1 | NM_014890 | 288436 | 261549 | 1.00 | 0.90 | -1.21 | 2.10 |
| 64 | B04 | 10263 | DOC-1R | NM_005851 | 273746 | 273978 | 0.95 | 0.94 | 0.78 | 0.34 |

| | | | | | | | | | | |
|---|---|---|---|---|---|---|---|---|---|---|
| 64 | B05 | 1795 | DOCK3 | NM_004947 | 279589 | 277974 | 0.97 | 0.96 | 0.48 | 0.15 |
| 64 | B06 | 10589 | DRAP1 | NM_006442 | 283219 | 279786 | 0.98 | 0.96 | -0.03 | -0.25 |
| 64 | B07 | 129831 | DRB1 | NM_152945 | 279092 | 277437 | 0.97 | 0.95 | -1.90 | -1.37 |
| 64 | B08 | 27340 | DRIM | NM_014503 | 272162 | 269241 | 0.94 | 0.93 | 0.12 | -0.14 |
| 64 | B09 | 1827 | DSCR1 | NM_004414 | 278849 | 287947 | 0.97 | 0.99 | -0.66 | -1.51 |
| 64 | B10 | 1834 | DSPP | NM_014208 | 272874 | 277332 | 0.95 | 0.95 | -0.21 | -1.30 |
| 64 | B11 | 11034 | DSTN | NM_006870 | 269339 | 269934 | 0.93 | 0.93 | 0.02 | 0.46 |
| 64 | B12 | 1838 | DTNB | NM_021907 | 270126 | 271089 | 0.94 | 0.93 | 1.57 | 0.74 |
| 64 | C01 | NA | pos | NA | 84934 | 126660 | 0.29 | 0.44 | 37.39 | 21.66 |
| 64 | C02 | NA | NA | NA | 243457 | 255333 | 0.84 | 0.88 | 5.52 | 3.40 |
| 64 | C03 | 84062 | DTNBP1 | NM_032122 | 272837 | 271013 | 0.95 | 0.93 | 0.78 | 1.18 |
| 64 | C04 | 8291 | DYSF | NM_003494 | 297057 | 274392 | 1.03 | 0.94 | -5.05 | 0.71 |
| 64 | C05 | 27338 | UBE2S | NM_014501 | 278902 | 289644 | 0.97 | 1.00 | -0.53 | -1.08 |
| 64 | C06 | 1870 | E2F2 | NM_004091 | 271013 | 269153 | 0.94 | 0.93 | 1.28 | 1.69 |
| 64 | C07 | 1871 | E2F3 | NM_001949 | 244676 | 253793 | 0.85 | 0.87 | 3.88 | 2.41 |
| 64 | C08 | 1874 | E2F4 | NM_001950 | 288243 | 267340 | 1.00 | 0.92 | -4.26 | 0.56 |
| 64 | C09 | 1875 | E2F5 | NM_001951 | 267286 | 286555 | 0.93 | 0.98 | 0.52 | -0.88 |
| 64 | C10 | 1876 | E2F6 | NM_001952 | 262803 | 284446 | 0.91 | 0.98 | 0.67 | -1.88 |
| 64 | C11 | 55840 | EAF2 | NM_018456 | 277490 | 280107 | 0.96 | 0.96 | -2.76 | -0.56 |
| 64 | C12 | 10148 | EBI3 | NM_005755 | 293989 | 283555 | 1.02 | 0.97 | -4.37 | -0.60 |
| 64 | D01 | NA | neg | NA | 307510 | 322363 | 1.07 | 1.11 | -5.49 | -5.21 |
| 64 | D02 | NA | neg | NA | 277774 | 279079 | 0.96 | 0.96 | 0.49 | 0.93 |
| 64 | D03 | 1842 | ECM2 | NM_001393 | 283314 | 291834 | 0.98 | 1.00 | 0.55 | -0.87 |
| 64 | D04 | 1915 | EEF1A1 | NM_001402 | 281843 | 284356 | 0.98 | 0.98 | -0.12 | 0.20 |
| 64 | D05 | 1938 | EEF2 | NM_001961 | 274745 | 275524 | 0.95 | 0.95 | 2.18 | 1.83 |
| 64 | D06 | 1962 | EHHADH | NM_001966 | 281858 | 286416 | 0.98 | 0.98 | 0.97 | 0.14 |
| 64 | D07 | 1967 | EIF2B1 | NM_001414 | 287202 | 278677 | 1.00 | 0.96 | -2.80 | -0.22 |
| 64 | D08 | 8661 | EIF3S10 | NM_003750 | 237192 | 248427 | 0.82 | 0.85 | 7.88 | 4.15 |
| 64 | D09 | 1996 | ELAVL4 | NM_021952 | 280176 | 288322 | 0.97 | 0.99 | -0.20 | -0.23 |
| 64 | D10 | 2010 | EMD | NM_000117 | 277627 | 273622 | 0.96 | 0.94 | -0.44 | 0.56 |
| 64 | D11 | 2017 | CTTN | NM_005231 | 280921 | 283638 | 0.97 | 0.97 | -1.58 | -0.15 |
| 64 | D12 | 2026 | ENO2 | NM_001975 | 280963 | 292165 | 0.97 | 1.00 | 0.12 | -0.92 |
| 64 | E01 | NA | neg | NA | 291518 | 302281 | 1.01 | 1.04 | -4.20 | -3.44 |
| 64 | E02 | NA | neg | NA | 288123 | 370428 | 1.00 | 1.27 | -3.51 | -13.11 |
| 64 | E03 | 2027 | ENO3 | NM_001976 | 278899 | 269208 | 0.97 | 0.93 | -0.49 | 1.26 |
| 64 | E04 | 5167 | ENPP1 | NM_006208 | 271694 | 279616 | 0.94 | 0.96 | 0.00 | -0.20 |
| 64 | E05 | 260429 | EOS | NM_152891 | 293712 | 260679 | 1.02 | 0.90 | -3.56 | 2.86 |
| 64 | E06 | 2058 | EPRS | NM_004446 | 268087 | 280824 | 0.93 | 0.97 | 1.82 | -0.14 |
| 64 | E07 | 2059 | EPS8 | NM_004447 | 261079 | 259111 | 0.91 | 0.89 | 0.53 | 1.48 |
| 64 | E08 | 2071 | ERCC3 | NM_000122 | 265014 | 269087 | 0.92 | 0.92 | 0.36 | 0.14 |
| 64 | E09 | 2072 | ERCC4 | NM_005236 | 269610 | 258416 | 0.93 | 0.89 | 0.00 | 2.94 |
| 64 | E10 | 2079 | ERH | NM_004450 | 265727 | 273653 | 0.92 | 0.94 | 0.03 | -0.52 |
| 64 | E11 | 10595 | ERN2 | XM_370947 | 263576 | 276709 | 0.91 | 0.95 | -0.01 | -0.25 |
| 64 | E12 | 30001 | ERO1L | NM_014584 | 292217 | 283335 | 1.01 | 0.97 | -4.06 | -0.74 |
| 64 | F01 | NA | NA | NA | 265194 | 273613 | 0.92 | 0.94 | 0.89 | -0.29 |
| 64 | F02 | NA | pos | NA | 119789 | 178169 | 0.42 | 0.61 | 30.13 | 13.26 |
| 64 | F03 | 55081 | ESRRBL1 | NM_018010 | 277600 | 274887 | 0.96 | 0.94 | -0.44 | -0.46 |
| 64 | F04 | 2115 | ETV1 | NM_004956 | 270628 | 275888 | 0.94 | 0.95 | 0.00 | -0.59 |
| 64 | F05 | 2119 | ETV5 | NM_004454 | 280743 | 271664 | 0.97 | 0.93 | -1.16 | 0.38 |
| 64 | F06 | 2121 | EVC | NM_014556 | 275938 | 283756 | 0.96 | 0.98 | 0.03 | -1.48 |
| 64 | F07 | 132884 | EVC2 | NM_147127 | 262158 | 260434 | 0.91 | 0.90 | 0.11 | 0.37 |
| 64 | F08 | 11322 | EVER1 | NM_007267 | 266367 | 272504 | 0.92 | 0.94 | -0.12 | -1.27 |
| 64 | F09 | 147138 | EVER2 | NM_152468 | 268615 | 260800 | 0.93 | 0.90 | 0.00 | 1.68 |
| 64 | F10 | 2124 | EVI2B | NM_006495 | 264323 | 261914 | 0.92 | 0.90 | 0.10 | 0.22 |
| 64 | F11 | 2139 | EYA2 | NM_005244 | 259168 | 269788 | 0.90 | 0.93 | 0.67 | -0.19 |
| 64 | F12 | 2145 | EZH1 | NM_001991 | 273857 | 270297 | 0.95 | 0.93 | -0.58 | 0.19 |
| 64 | G01 | NA | neg | NA | 354359 | 281907 | 1.23 | 0.97 | -16.48 | -1.66 |
| 64 | G02 | NA | neg | NA | 288634 | 266175 | 1.00 | 0.91 | -3.26 | 0.58 |
| 64 | G03 | 2146 | EZH2 | NM_004456 | 276042 | 271423 | 0.96 | 0.93 | 0.44 | -0.16 |
| 64 | G04 | 50848 | F11R | NM_016946 | 269002 | 263394 | 0.93 | 0.91 | 0.89 | 0.99 |
| 64 | G05 | 2162 | F13A1 | NM_000129 | 270921 | 274023 | 0.94 | 0.94 | 1.37 | -0.15 |
| 64 | G06 | 2165 | F13B | NM_001994 | 294799 | 265400 | 1.02 | 0.91 | -3.20 | 0.94 |
| 64 | G07 | 2169 | FABP2 | NM_000134 | 261281 | 280383 | 0.91 | 0.96 | 0.84 | -2.65 |
| 64 | G08 | 2170 | FABP3 | NM_004102 | 269462 | 264638 | 0.93 | 0.91 | -0.18 | -0.34 |
| 64 | G09 | 2167 | FABP4 | NM_001442 | 276840 | 268605 | 0.96 | 0.92 | -1.10 | 0.38 |
| 64 | G10 | 11124 | FAF1 | NM_007051 | 274420 | 257975 | 0.95 | 0.89 | -1.37 | 0.59 |
| 64 | G11 | 55179 | FAIM | NM_018147 | 264686 | 266053 | 0.92 | 0.91 | 0.11 | 0.15 |
| 64 | G12 | 2175 | FANCA | NM_000135 | 274357 | 271620 | 0.95 | 0.93 | -0.12 | -0.19 |
| 64 | H01 | NA | NA | NA | 231134 | 237857 | 0.80 | 0.82 | 9.37 | 4.85 |
| 64 | H02 | NA | pos | NA | 180706 | 160933 | 0.63 | 0.55 | 19.51 | 15.77 |
| 64 | H03 | 2176 | FANCC | NM_000136 | 270114 | 270911 | 0.94 | 0.93 | 2.70 | 0.16 |
| 64 | H04 | 2177 | FANCD2 | NM_033084 | 281060 | 278516 | 0.97 | 0.96 | -0.46 | -0.90 |
| 64 | H05 | 2178 | FANCE | NM_021922 | 282200 | 280323 | 0.98 | 0.96 | 0.18 | -0.79 |
| 64 | H06 | 2188 | FANCF | NM_022725 | 289599 | 278421 | 1.00 | 0.96 | -1.08 | -0.66 |
| 64 | H07 | 9855 | FARP2 | NM_014808 | 271347 | 278196 | 0.94 | 0.96 | -0.11 | -2.08 |
| 64 | H08 | 2193 | FARSLA | NM_004461 | 286464 | 265099 | 0.99 | 0.91 | -2.52 | -0.15 |
| 64 | H09 | 51341 | ZBTB7 | NM_015898 | 271298 | 271492 | 0.94 | 0.93 | 1.09 | 0.23 |
| 64 | H10 | 10516 | FBLN5 | NM_006329 | 272488 | 256354 | 0.94 | 0.88 | 0.10 | 1.07 |
| 64 | H11 | 2203 | FBP1 | NM_000507 | 272287 | 258785 | 0.94 | 0.89 | -0.34 | 1.44 |
| 64 | H12 | 2214 | FCGR3A | NM_000569 | 260878 | 262795 | 0.90 | 0.90 | 3.67 | 1.32 |
| 65 | A01 | NA | pos | NA | 61894 | 72456 | 0.21 | 0.24 | 28.01 | 31.48 |
| 65 | A02 | NA | NA | NA | 275792 | 282661 | 0.92 | 0.94 | 1.76 | 1.33 |
| 65 | A03 | 2224 | FDPS | NM_002004 | 333971 | 318761 | 1.11 | 1.06 | -3.99 | -3.79 |
| 65 | A04 | 9638 | FEZ1 | NM_005103 | 314455 | 292467 | 1.04 | 0.97 | -2.38 | 0.79 |
| 65 | A05 | 2243 | FGA | NM_000508 | 298822 | 311607 | 0.99 | 1.03 | -0.66 | -2.65 |
| 65 | A06 | 2244 | FGB | NM_005141 | 292111 | 276696 | 0.97 | 0.92 | 0.21 | 2.55 |
| 65 | A07 | 2257 | FGF12 | NM_004113 | 285427 | 303791 | 0.95 | 1.01 | 0.15 | -2.01 |
| 65 | A08 | 2247 | FGF2 | NM_002006 | 268509 | 282833 | 0.89 | 0.94 | 2.25 | 1.25 |

| | | | | | | | | | | |
|---|---|---|---|---|---|---|---|---|---|---|
| 65 | A09 | 11116 | FGFR1OP | NM_007045 | 290251 | 286416 | 0.96 | 0.95 | -0.98 | -0.09 |
| 65 | A10 | 2266 | FGG | NM_000509 | 287514 | 285338 | 0.96 | 0.95 | -0.14 | 0.09 |
| 65 | A11 | 2271 | FH | NM_000143 | 275700 | 288975 | 0.92 | 0.96 | 0.29 | -1.22 |
| 65 | A12 | 9457 | FHL5 | NM_020482 | 283769 | 274713 | 0.94 | 0.91 | 1.25 | 2.60 |
| 65 | B01 | NA | neg | NA | 311970 | 303572 | 1.04 | 1.01 | -3.79 | -2.31 |
| 65 | B02 | NA | neg | NA | 287422 | 281598 | 0.95 | 0.94 | -0.78 | 0.84 |
| 65 | B03 | 59347 | FKSG2 | NM_021631 | 284886 | 288517 | 0.95 | 0.96 | 0.92 | -0.09 |
| 65 | B04 | 2313 | FLI1 | NM_002017 | 284715 | 293468 | 0.95 | 0.97 | 0.16 | 0.00 |
| 65 | B05 | 55076 | FLJ10134 | NM_018004 | 292623 | 285511 | 0.97 | 0.95 | -1.01 | 0.45 |
| 65 | B06 | 55200 | FLJ10665 | NM_018173 | 285668 | 304635 | 0.95 | 1.01 | -0.10 | -2.10 |
| 65 | B07 | 55219 | FLJ10747 | NM_018202 | 283175 | 289104 | 0.94 | 0.96 | -0.68 | -0.55 |
| 65 | B08 | 54619 | CCNJ | NM_019084 | 289767 | 297560 | 0.96 | 0.99 | -1.47 | -1.50 |
| 65 | B09 | 55765 | FLJ10901 | NM_018265 | 266268 | 277945 | 0.88 | 0.92 | 0.86 | 0.48 |
| 65 | B10 | 55277 | FLJ10986 | NM_018291 | 280683 | 281519 | 0.93 | 0.93 | -0.41 | -0.01 |
| 65 | B11 | 55785 | FGD6 | XM_370702 | 257365 | 273210 | 0.85 | 0.91 | 1.43 | 0.39 |
| 65 | B12 | 79685 | SAP30L | NM_024632 | 283646 | 281897 | 0.94 | 0.94 | 0.16 | 0.92 |
| 65 | C01 | NA | pos | NA | 79844 | 130336 | 0.27 | 0.43 | 24.94 | 21.05 |
| 65 | C02 | NA | NA | NA | 248920 | 252742 | 0.83 | 0.84 | 4.20 | 3.50 |
| 65 | C03 | 79792 | GSDMDC1 | NM_024736 | 283375 | 277530 | 0.94 | 0.92 | 1.35 | 0.00 |
| 65 | C04 | 79888 | FLJ12443 | NM_024830 | 275464 | 293506 | 0.92 | 0.97 | 1.54 | -1.48 |
| 65 | C05 | 79648 | FLJ12847 | NM_024596 | 291478 | 278310 | 0.97 | 0.92 | -0.63 | 0.00 |
| 65 | C06 | 79968 | FLJ12973 | NM_024908 | 274224 | 262506 | 0.91 | 0.87 | 1.55 | 2.46 |
| 65 | C07 | 79935 | FLJ13265 | NM_024877 | 275941 | 274860 | 0.92 | 0.91 | 0.45 | 0.01 |
| 65 | C08 | 79875 | FLJ13710 | NM_024817 | 266081 | 305660 | 0.88 | 1.01 | 1.68 | -4.15 |
| 65 | C09 | 79788 | FLJ14345 | NM_024733 | 286633 | 250217 | 0.95 | 0.83 | -1.40 | 2.97 |
| 65 | C10 | 84904 | C9ORF100 | NM_032818 | 283620 | 271349 | 0.94 | 0.90 | -0.52 | -0.03 |
| 65 | C11 | 54848 | FLJ20184 | NM_017700 | 275382 | 263393 | 0.91 | 0.87 | -0.53 | 0.32 |
| 65 | C12 | 55631 | FLJ20331 | NM_017768 | 290487 | 299838 | 0.96 | 1.00 | -0.43 | -3.13 |
| 65 | D01 | NA | neg | NA | 320427 | 321637 | 1.06 | 1.07 | -4.48 | -6.02 |
| 65 | D02 | NA | neg | NA | 318690 | 314440 | 1.06 | 1.04 | -4.27 | -4.99 |
| 65 | D03 | 79832 | FLJ21924 | NM_024774 | 310945 | NA | 1.03 | NA | -1.93 | NA |
| 65 | D04 | 84173 | RBM29 | NM_032213 | 283237 | 288621 | 0.94 | 0.96 | 0.68 | -0.43 |
| 65 | D05 | 79637 | FLJ22160 | NM_024585 | 280319 | 270661 | 0.93 | 0.90 | 0.84 | 1.46 |
| 65 | D06 | 79651 | RHBDL6 | NM_024599 | 379791 | 278542 | 1.26 | 0.92 | -11.31 | 0.52 |
| 65 | D07 | 80162 | FLJ22635 | NM_025092 | 272081 | 272533 | 0.90 | 0.90 | 1.03 | 0.70 |
| 65 | D08 | 151126 | ZNF533 | NM_152520 | 281602 | 279251 | 0.94 | 0.93 | -0.12 | 0.00 |
| 65 | D09 | 159119 | HSFY2 | NM_153716 | 275224 | 283056 | 0.91 | 0.94 | 0.10 | -1.38 |
| 65 | D10 | 253832 | FLJ25952 | NM_153251 | 270849 | 268257 | 0.90 | 0.89 | 1.14 | 0.77 |
| 65 | D11 | 166968 | FLJ35954 | NM_152622 | 274830 | 273626 | 0.91 | 0.91 | -0.36 | -0.79 |
| 65 | D12 | 158677 | FLJ38564 | NM_152579 | 297995 | 286065 | 0.99 | 0.95 | -1.25 | -0.80 |
| 65 | E01 | NA | neg | NA | 302662 | 318525 | 1.01 | 1.06 | -4.05 | -5.95 |
| 65 | E02 | NA | neg | NA | 274695 | 298715 | 0.91 | 0.99 | -0.62 | -3.11 |
| 65 | E03 | 2318 | FLNC | NM_001458 | 278795 | 265693 | 0.93 | 0.88 | 0.27 | 1.68 |
| 65 | E04 | 2328 | FMO3 | NM_006894 | 277962 | 269490 | 0.92 | 0.89 | -0.42 | 1.94 |
| 65 | E05 | 2334 | FMR2 | NM_002025 | 267298 | 287472 | 0.89 | 0.95 | 0.69 | -1.33 |
| 65 | E06 | 10023 | FRAT1 | NM_005479 | 374590 | 283198 | 1.24 | 0.94 | -12.42 | -0.52 |
| 65 | E07 | 6624 | FSCN1 | NM_003088 | 260064 | 260141 | 0.86 | 0.86 | 0.75 | 2.11 |
| 65 | E08 | 10272 | FSTL3 | NM_005860 | 268570 | 266937 | 0.89 | 0.89 | -0.27 | 1.39 |
| 65 | E09 | 2512 | FTL | NM_000146 | 256040 | 270670 | 0.85 | 0.90 | 0.71 | 0.02 |
| 65 | E10 | 2519 | FUCA2 | NM_032020 | 279560 | 278825 | 0.93 | 0.93 | -1.68 | -1.12 |
| 65 | E11 | 2521 | FUS | NM_004960 | 272408 | 265733 | 0.90 | 0.88 | -1.82 | -0.03 |
| 65 | E12 | 2525 | FUT3 | NM_000149 | 267992 | 281956 | 0.89 | 0.94 | 0.68 | -0.58 |
| 65 | F01 | NA | NA | NA | 291634 | 295801 | 0.97 | 0.98 | -1.90 | -3.45 |
| 65 | F02 | NA | pos | NA | 107918 | 226105 | 0.36 | 0.75 | 20.64 | 6.54 |
| 65 | F03 | 2537 | G1P3 | NM_002038 | 357067 | 277387 | 1.19 | 0.92 | -8.54 | -0.75 |
| 65 | F04 | 25758 | G2 | XM_039515 | 282317 | 277789 | 0.94 | 0.92 | -0.16 | 0.00 |
| 65 | F05 | 92579 | G6PC3 | NM_138387 | 268107 | 272918 | 0.89 | 0.91 | 1.39 | 0.00 |
| 65 | F06 | 2548 | GAA | NM_000152 | 279030 | 267913 | 0.93 | 0.89 | 0.10 | 0.91 |
| 65 | F07 | 139716 | GAB3 | NM_080612 | 273903 | 261403 | 0.91 | 0.87 | -0.15 | 1.17 |
| 65 | F08 | 23710 | GABARAPL1 | NM_031412 | 271839 | 271358 | 0.90 | 0.90 | 0.12 | 0.00 |
| 65 | F09 | 2572 | GAD2 | NM_000818 | 258613 | 263852 | 0.86 | 0.88 | 1.19 | 0.24 |
| 65 | F10 | 4616 | GADD45B | NM_015675 | 254426 | 262159 | 0.85 | 0.87 | 2.20 | 0.51 |
| 65 | F11 | 2619 | GAS1 | NM_002048 | 264784 | 276879 | 0.88 | 0.92 | -0.08 | -2.39 |
| 65 | F12 | 2620 | GAS2 | NM_005256 | 284001 | 280439 | 0.94 | 0.93 | -0.49 | -1.12 |
| 65 | G01 | NA | neg | NA | 299428 | 279201 | 0.99 | 0.93 | -3.65 | -1.00 |
| 65 | G02 | NA | neg | NA | 277566 | 285849 | 0.92 | 0.95 | -0.97 | -1.95 |
| 65 | G03 | 10634 | GAS2L1 | NM_006478 | 283123 | 267579 | 0.94 | 0.89 | -0.27 | 0.73 |
| 65 | G04 | 8089 | GAS41 | NM_006530 | 270249 | 280297 | 0.90 | 0.93 | 0.53 | -0.29 |
| 65 | G05 | 8522 | GAS7 | NM_003644 | 271064 | 261607 | 0.90 | 0.87 | 0.23 | 1.70 |
| 65 | G06 | 9737 | GASP | NM_014710 | 266989 | 286284 | 0.89 | 0.95 | 0.78 | -1.65 |
| 65 | G07 | 2627 | GATA6 | NM_005257 | 292889 | 273894 | 0.97 | 0.91 | -3.28 | -0.55 |
| 65 | G08 | 352954 | GATS | NM_178831 | 274389 | 269854 | 0.91 | 0.90 | -0.99 | 0.29 |
| 65 | G09 | 2632 | GBE1 | NM_000158 | 262645 | 271397 | 0.87 | 0.90 | -0.10 | -0.76 |
| 65 | G10 | 2638 | GC | NM_000583 | 252197 | 269422 | 0.84 | 0.89 | 1.68 | -0.46 |
| 65 | G11 | 2729 | GCLC | NM_001498 | 256903 | 252478 | 0.85 | 0.84 | 0.08 | 1.18 |
| 65 | G12 | 7841 | GCS1 | NM_006302 | 274812 | 269059 | 0.91 | 0.89 | -0.16 | 0.58 |
| 65 | H01 | NA | NA | NA | 239685 | 347369 | 0.80 | 1.15 | 6.17 | -9.42 |
| 65 | H02 | NA | pos | NA | 94420 | 145314 | 0.31 | 0.48 | 24.00 | 19.56 |
| 65 | H03 | 54332 | GDAP1 | NM_018972 | 278483 | 280643 | 0.93 | 0.93 | 2.80 | 0.21 |
| 65 | H04 | 2671 | GFER | NM_005262 | 297079 | 286649 | 0.99 | 0.95 | -0.27 | 0.15 |
| 65 | H05 | 25816 | TNFAIP8 | NM_014350 | 295124 | 317010 | 0.98 | 1.05 | -0.23 | -4.90 |
| 65 | H06 | 2688 | GH1 | NM_000515 | 294577 | 290337 | 0.98 | 0.96 | -0.11 | -0.88 |
| 65 | H07 | 92283 | GIOT-1 | NM_153257 | 333609 | 279555 | 1.11 | 0.93 | -5.78 | -0.01 |
| 65 | H08 | 57165 | GJA12 | NM_020435 | 277901 | 282367 | 0.92 | 0.94 | 1.08 | -0.15 |
| 65 | H09 | 2700 | GJA3 | NM_021954 | 283385 | 275672 | 0.94 | 0.92 | -0.15 | -0.02 |
| 65 | H10 | 2702 | GJA5 | NM_005266 | 285085 | 275629 | 0.95 | 0.92 | 0.14 | 0.01 |
| 65 | H11 | 2703 | GJA8 | NM_005267 | 274453 | 269945 | 0.91 | 0.90 | 0.43 | 0.03 |
| 65 | H12 | 11010 | GLIPR1 | NM_006851 | 269442 | 267145 | 0.90 | 0.89 | 3.00 | 2.21 |

| | | | | | | | | | | |
|---|---|---|---|---|---|---|---|---|---|---|
| 66 | A01 | NA | pos | NA | 73098 | 89737 | 0.23 | 0.30 | 26.01 | 39.58 |
| 66 | A02 | NA | NA | NA | 288653 | 284226 | 0.89 | 0.96 | 1.08 | -0.33 |
| 66 | A03 | 2739 | GLO1 | NM_006708 | 326950 | 307213 | 1.01 | 1.04 | -1.68 | -3.88 |
| 66 | A04 | 2745 | GLRX | NM_002064 | 299721 | 291559 | 0.93 | 0.98 | 0.26 | -0.53 |
| 66 | A05 | 29998 | GLTSCR1 | NM_015711 | 328202 | 277074 | 1.02 | 0.93 | -1.47 | 1.86 |
| 66 | A06 | 29997 | GLTSCR2 | NM_015710 | 279106 | 275424 | 0.86 | 0.93 | 1.61 | 0.17 |
| 66 | A07 | 2746 | GLUD1 | NM_005271 | 318810 | 311956 | 0.99 | 1.05 | -2.03 | -5.41 |
| 66 | A08 | 2765 | GML | NM_002066 | 297327 | 287259 | 0.92 | 0.97 | -0.31 | -1.56 |
| 66 | A09 | 51053 | GMNN | NM_015895 | 287177 | 277489 | 0.89 | 0.94 | 0.64 | 0.02 |
| 66 | A10 | 8833 | GMPS | NM_003875 | 354994 | 273017 | 1.10 | 0.92 | -7.87 | 1.00 |
| 66 | A11 | 10399 | GNB2L1 | NM_006098 | 268258 | 270788 | 0.83 | 0.91 | 1.70 | -0.02 |
| 66 | A12 | 10578 | GNLY | NM_006433 | 281280 | 276049 | 0.87 | 0.93 | 2.33 | 2.14 |
| 66 | B01 | NA | neg | NA | 309063 | 295932 | 0.96 | 1.00 | -2.09 | -2.80 |
| 66 | B02 | NA | neg | NA | 371607 | 285123 | 1.15 | 0.96 | -9.33 | -0.59 |
| 66 | B03 | 27232 | GNMT | NM_018960 | 301584 | 281839 | 0.93 | 0.95 | 0.43 | 1.26 |
| 66 | B04 | 9950 | GOLGA5 | NM_005113 | 305810 | 280096 | 0.95 | 0.94 | -1.26 | 1.75 |
| 66 | B05 | 2804 | GOLGB1 | NM_004487 | 307760 | 286766 | 0.95 | 0.97 | 0.08 | -0.20 |
| 66 | B06 | 8733 | GPAA1 | NM_003801 | 288071 | 286986 | 0.89 | 0.97 | -0.25 | -2.28 |
| 66 | B07 | 2719 | GPC3 | NM_004484 | 303135 | 296490 | 0.94 | 1.00 | -1.03 | -2.31 |
| 66 | B08 | 57720 | GPR107 | NM_020960 | 287348 | 275298 | 0.89 | 0.93 | 0.03 | 0.82 |
| 66 | B09 | 2873 | GPS1 | NM_004127 | 288202 | 283495 | 0.89 | 0.96 | -0.29 | -1.29 |
| 66 | B10 | 2876 | GPX1 | NM_000581 | 278714 | 278482 | 0.86 | 0.94 | 0.14 | -0.20 |
| 66 | B11 | 2897 | GRIK1 | NM_000830 | 274164 | 266258 | 0.85 | 0.90 | 0.20 | 0.84 |
| 66 | B12 | 2905 | GRIN2C | NM_000835 | 294918 | 285114 | 0.91 | 0.96 | -0.06 | 0.21 |
| 66 | C01 | NA | pos | NA | 88164 | 96511 | 0.27 | 0.33 | 22.90 | 36.13 |
| 66 | C02 | NA | NA | NA | 279018 | 260052 | 0.86 | 0.88 | 0.82 | 2.57 |
| 66 | C03 | 56850 | GRIPAP1 | NM_020137 | 286091 | 272327 | 0.89 | 0.92 | 1.67 | 1.22 |
| 66 | C04 | 2935 | GSPT1 | NM_002094 | 264534 | 275155 | 0.82 | 0.93 | 2.95 | 0.77 |
| 66 | C05 | 2950 | GSTP1 | NM_000852 | 302302 | 268852 | 0.94 | 0.91 | 0.15 | 1.48 |
| 66 | C06 | 2952 | GSTT1 | NM_000853 | 288669 | 267920 | 0.89 | 0.90 | -0.88 | -0.35 |
| 66 | C07 | 2953 | GSTT2 | NM_000854 | 275061 | 265437 | 0.85 | 0.90 | 1.65 | 2.07 |
| 66 | C08 | 2968 | GTF2H4 | NM_001517 | 271617 | 291348 | 0.84 | 0.98 | 1.29 | -4.46 |
| 66 | C09 | 2976 | GTF3C2 | NM_001521 | 283485 | 265889 | 0.88 | 0.90 | -0.31 | 0.34 |
| 66 | C10 | 9328 | GTF3C5 | NM_012087 | 280616 | 269458 | 0.87 | 0.91 | -0.64 | -0.33 |
| 66 | C11 | 51512 | GTSE1 | NM_016426 | 272715 | 268895 | 0.84 | 0.91 | -0.20 | -1.69 |
| 66 | C12 | 2978 | GUCA1A | NM_000409 | 293793 | 296258 | 0.91 | 1.00 | -0.49 | -4.07 |
| 66 | D01 | NA | neg | NA | 329356 | 312472 | 1.02 | 1.05 | -3.95 | -6.05 |
| 66 | D02 | NA | neg | NA | 332321 | 297084 | 1.03 | 1.00 | -4.29 | -2.89 |
| 66 | D03 | 9626 | GUCA1C | NM_005459 | 339402 | 298898 | 1.05 | 1.01 | -3.45 | -2.09 |
| 66 | D04 | 2993 | GYPA | NM_002099 | 310728 | 289322 | 0.96 | 0.98 | -1.34 | 0.00 |
| 66 | D05 | 2995 | GYPC | NM_002101 | 322306 | 288305 | 1.00 | 0.97 | -1.11 | -0.37 |
| 66 | D06 | 283120 | H19 | AK056774 | 290058 | 273146 | 0.90 | 0.92 | 0.01 | 0.71 |
| 66 | D07 | 55506 | H2AFY2 | NM_018649 | 282052 | 275151 | 0.87 | 0.93 | 1.89 | 2.21 |
| 66 | D08 | 23526 | HA-1 | NM_012292 | 292131 | 276692 | 0.90 | 0.93 | -0.03 | 0.68 |
| 66 | D09 | 3032 | HADHB | NM_000183 | 279064 | 277934 | 0.86 | 0.94 | 1.26 | 0.00 |
| 66 | D10 | 84264 | HAGHL | NM_032304 | 274682 | 275928 | 0.85 | 0.93 | 1.09 | 0.47 |
| 66 | D11 | 10238 | HAN11 | NM_005828 | 289375 | 279610 | 0.90 | 0.94 | -1.07 | -1.75 |
| 66 | D12 | 9464 | HAND2 | NM_021973 | 298081 | 299698 | 0.92 | 1.01 | 0.06 | -2.64 |
| 66 | E01 | NA | neg | NA | 326856 | 316503 | 1.01 | 1.07 | -3.97 | -8.45 |
| 66 | E02 | NA | neg | NA | 285946 | 294260 | 0.88 | 0.99 | 0.76 | -3.88 |
| 66 | E03 | 9001 | HAP1 | NM_003949 | 279973 | 275037 | 0.87 | 0.93 | 3.11 | 1.23 |
| 66 | E04 | 55664 | CDC37L1 | NM_017913 | 298628 | 285512 | 0.92 | 0.96 | -0.25 | -0.79 |
| 66 | E05 | 8520 | HAT1 | NM_003642 | 346183 | 296682 | 1.07 | 1.00 | -4.18 | -3.66 |
| 66 | E06 | 84913 | ATOH8 | NM_032827 | 296306 | 269775 | 0.92 | 0.91 | -1.02 | -0.17 |
| 66 | E07 | 3045 | HBD | NM_000519 | 280094 | NA | 0.87 | NA | 1.81 | NA |
| 66 | E08 | 3046 | HBE1 | NM_005330 | 276338 | 271073 | 0.86 | 0.91 | 1.49 | 0.27 |
| 66 | E09 | 3048 | HBG2 | NM_000184 | 268429 | NA | 0.83 | NA | 2.18 | NA |
| 66 | E10 | 3049 | HBQ1 | NM_005331 | 297873 | 267438 | 0.92 | 0.90 | -1.90 | 0.65 |
| 66 | E11 | 3050 | HBZ | NM_005332 | 282877 | 262617 | 0.88 | 0.89 | -0.63 | 0.17 |
| 66 | E12 | 65991 | HCBP6 | NM_023934 | 294198 | 289285 | 0.91 | 0.98 | 0.20 | -2.07 |
| 66 | F01 | NA | NA | NA | 284137 | 294414 | 0.88 | 0.99 | -0.40 | -3.61 |
| 66 | F02 | NA | pos | NA | 112188 | 188336 | 0.35 | 0.64 | 19.49 | 18.16 |
| 66 | F03 | 25875 | HCCR1 | NM_015416 | 330271 | 282065 | 1.02 | 0.95 | -4.07 | 0.10 |
| 66 | F04 | 29115 | HCNGP | NM_013260 | 280316 | 283167 | 0.87 | 0.96 | 0.50 | 0.00 |
| 66 | F05 | 10870 | HCST | NM_014266 | 278159 | 283845 | 0.86 | 0.96 | 2.32 | -0.72 |
| 66 | F06 | 10403 | KNTC2 | NM_006101 | 246967 | 257558 | 0.76 | 0.87 | 3.32 | 2.64 |
| 66 | F07 | 9709 | HERPUD1 | NM_014685 | 284081 | 279785 | 0.88 | 0.94 | -0.02 | 0.00 |
| 66 | F08 | 55502 | HES6 | NM_018645 | 276253 | 274927 | 0.85 | 0.93 | 0.13 | -0.22 |
| 66 | F09 | 3075 | HF1 | NM_000186 | 277757 | 272342 | 0.86 | 0.92 | -0.27 | -0.11 |
| 66 | F10 | 9910 | HHL | NM_014857 | 269452 | 271137 | 0.83 | 0.91 | 0.02 | 0.19 |
| 66 | F11 | 10086 | HHLA1 | NM_005712 | 271631 | 264826 | 0.84 | 0.89 | -0.70 | 0.02 |
| 66 | F12 | 26275 | HIBCH | NM_014362 | 291780 | 281679 | 0.90 | 0.95 | -0.89 | -0.21 |
| 66 | G01 | NA | neg | NA | 291521 | 301406 | 0.90 | 1.02 | -0.55 | -5.06 |
| 66 | G02 | NA | neg | NA | 319467 | 292783 | 0.99 | 0.99 | -3.79 | -3.29 |
| 66 | G03 | 3094 | HINT1 | NM_005340 | 304840 | 282915 | 0.94 | 0.95 | -0.43 | -0.10 |
| 66 | G04 | 3092 | HIP1 | NM_005338 | 288493 | 282600 | 0.89 | 0.95 | 0.25 | 0.10 |
| 66 | G05 | 8479 | HIRIP3 | NM_003609 | 289487 | 272036 | 0.90 | 0.92 | 1.71 | 1.68 |
| 66 | G06 | 8351 | HIST1H3D | NM_003530 | 281813 | 279580 | 0.87 | 0.94 | -0.01 | -1.89 |
| 66 | G07 | 8366 | HIST1H4B | NM_003544 | 290125 | 275335 | 0.90 | 0.93 | -0.02 | 0.89 |
| 66 | G08 | 8364 | HIST1H4C | NM_003542 | 283856 | 272733 | 0.88 | 0.92 | -0.05 | 0.22 |
| 66 | G09 | 8362 | HIST1H4K | NM_003541 | 279114 | 278758 | 0.86 | 0.94 | 0.27 | -1.45 |
| 66 | G10 | 3096 | HIVEP1 | NM_002114 | 275581 | 282091 | 0.85 | 0.95 | 0.01 | -2.07 |
| 66 | G11 | 27126 | HMG1L10 | NM_172363 | 269842 | 268820 | 0.84 | 0.91 | 0.21 | -0.82 |
| 66 | G12 | 2180 | ACSL1 | NM_001995 | 294873 | 279461 | 0.91 | 0.94 | -0.55 | 0.23 |
| 66 | H01 | NA | NA | NA | 240427 | 255369 | 0.74 | 0.86 | 5.13 | 5.17 |
| 66 | H02 | NA | pos | NA | 86960 | 155618 | 0.27 | 0.52 | 22.88 | 25.64 |
| 66 | H03 | 54478 | FAM64A | NM_019013 | 286798 | 287862 | 0.89 | 0.97 | 1.42 | -0.32 |
| 66 | H04 | 51275 | FLJ39616 | NM_016534 | 314861 | 296213 | 0.97 | 1.00 | -3.03 | -1.91 |

| | | | | | | | | | | |
|---|---|---|---|---|---|---|---|---|---|---|
| 66 | H05 | 2304 | FOXE1 | NM_004473 | 302983 | 283089 | 0.94 | 0.95 | -0.08 | 0.20 |
| 66 | H06 | 2292 | FOXG1C | X74144 | 274722 | 270092 | 0.85 | 0.91 | 0.58 | 0.84 |
| 66 | H07 | 23401 | FRAT2 | NM_012083 | 287831 | 286634 | 0.89 | 0.97 | 0.02 | -0.64 |
| 66 | H08 | 192137 | C2orf12 | BC069763 | 300388 | 283824 | 0.93 | 0.96 | -2.19 | -1.27 |
| 66 | H09 | 29923 | HIG2 | NM_013332 | 282410 | 273447 | 0.87 | 0.92 | -0.34 | 0.43 |
| 66 | H10 | 7325 | UBE2E2 | NM_152653 | 273799 | 276774 | 0.85 | 0.93 | -0.01 | -0.19 |
| 66 | H11 | 3040 | HBA2 | NM_000517 | 267627 | 267220 | 0.83 | 0.90 | 0.23 | 0.30 |
| 66 | H12 | 51352 | WIT-1 | NM_015855 | 275767 | 276091 | 0.85 | 0.93 | 1.43 | 1.71 |
| 67 | A01 | NA | pos | NA | 67455 | 95366 | 0.23 | 0.29 | 27.18 | 32.17 |
| 67 | A02 | NA | NA | NA | 266555 | 301093 | 0.91 | 0.92 | 2.84 | 3.74 |
| 67 | A03 | 3148 | HMGB2 | NM_002129 | 307301 | 320505 | 1.05 | 0.98 | -0.95 | 0.12 |
| 67 | A04 | 3149 | HMGB3 | NM_005342 | 297574 | 331833 | 1.01 | 1.01 | 0.00 | -1.36 |
| 67 | A05 | 3503 | IGHG4 | BC025985 | 296186 | 335996 | 1.01 | 1.03 | -0.55 | -0.96 |
| 67 | A06 | 3507 | IGHM | BC020240 | 295488 | 331033 | 1.01 | 1.01 | 0.00 | 0.31 |
| 67 | A07 | 3514 | IGKC | AF113887 | 378157 | 316946 | 1.29 | 0.97 | -10.90 | 0.93 |
| 67 | A08 | 28831 | IGLJ3 | BC007782 | 279558 | 333391 | 0.95 | 1.02 | 0.41 | -0.90 |
| 67 | A09 | 3543 | IGLL1 | NM_020070 | 271407 | 305885 | 0.92 | 0.94 | 2.00 | 2.95 |
| 67 | A10 | 57818 | G6PC2 | NM_021176 | 279082 | 333393 | 0.95 | 1.02 | 0.16 | -0.21 |
| 67 | A11 | 3592 | IL12A | NM_000882 | 281752 | 337229 | 0.96 | 1.03 | -0.28 | -1.04 |
| 67 | A12 | 3596 | IL13 | NM_002188 | 271483 | 303010 | 0.93 | 0.93 | 2.33 | 3.89 |
| 67 | B01 | NA | neg | NA | 304237 | 328105 | 1.04 | 1.00 | -2.69 | 0.56 |
| 67 | B02 | NA | neg | NA | 276003 | 335387 | 0.94 | 1.03 | 0.76 | -0.44 |
| 67 | B03 | 55522 | ILK-2 | AJ277481 | 280878 | 324716 | 0.96 | 0.99 | 1.35 | 0.10 |
| 67 | B04 | 79166 | ILT10 | NM_024317 | 297054 | 327395 | 1.01 | 1.00 | -0.87 | -0.19 |
| 67 | B05 | 79168 | ILT8 | NM_024318 | 284166 | 342603 | 0.97 | 1.05 | -0.01 | -1.32 |
| 67 | B06 | 10643 | IMP-3 | NM_006547 | 281835 | 339265 | 0.96 | 1.04 | 0.75 | -0.27 |
| 67 | B07 | 3614 | IMPDH1 | NM_000883 | 273075 | 318534 | 0.93 | 0.97 | 1.02 | 1.26 |
| 67 | B08 | 3617 | IMPG1 | NM_001563 | 286676 | 348954 | 0.98 | 1.07 | -1.39 | -2.50 |
| 67 | B09 | 50939 | IMPG2 | NM_016247 | 280116 | 342993 | 0.95 | 1.05 | 0.01 | -1.63 |
| 67 | B10 | 27160 | ING2 | NM_058171 | 274104 | 332734 | 0.93 | 1.02 | -0.16 | 0.43 |
| 67 | B11 | 3631 | INPP4A | NM_001566 | 272370 | 321235 | 0.93 | 0.98 | -0.06 | 1.72 |
| 67 | B12 | 22876 | INPP5F | NM_014937 | 271992 | 315819 | 0.93 | 0.97 | 1.34 | 2.67 |
| 67 | C01 | NA | pos | NA | 81129 | 97189 | 0.28 | 0.30 | 24.05 | 32.69 |
| 67 | C02 | NA | NA | NA | 259055 | 273649 | 0.88 | 0.84 | 2.30 | 8.31 |
| 67 | C03 | 3637 | INSAF | S73205 | 273053 | 323037 | 0.93 | 0.99 | 1.77 | 0.55 |
| 67 | C04 | 3638 | INSIG1 | NM_005542 | 280092 | 319489 | 0.95 | 0.98 | 0.68 | 1.12 |
| 67 | C05 | 51141 | INSIG2 | NM_016133 | 283485 | 336548 | 0.97 | 1.03 | -0.46 | -0.27 |
| 67 | C06 | 83417 | IRTA1 | NM_031282 | 279826 | 337457 | 0.95 | 1.03 | 0.46 | 0.19 |
| 67 | C07 | 83416 | IRTA2 | NM_031281 | 250969 | 304291 | 0.86 | 0.93 | 3.20 | 3.45 |
| 67 | C08 | 10265 | IRX5 | NM_005853 | 259764 | 327548 | 0.89 | 1.00 | 1.37 | 0.68 |
| 67 | C09 | 3670 | ISL1 | NM_002202 | 287276 | 334503 | 0.98 | 1.02 | -1.40 | -0.23 |
| 67 | C10 | 22801 | ITGA11 | NM_012211 | 281478 | 344921 | 0.96 | 1.05 | -1.59 | -1.03 |
| 67 | C11 | 3681 | ITGAD | XM_496142 | 280100 | 340440 | 0.95 | 1.04 | -1.54 | -0.71 |
| 67 | C12 | 3695 | ITGB7 | NM_000889 | 290774 | 348316 | 0.99 | 1.06 | -1.49 | -1.60 |
| 67 | D01 | NA | neg | NA | 310439 | 322921 | 1.06 | 0.99 | -3.35 | 1.49 |
| 67 | D02 | NA | neg | NA | 294304 | 339693 | 1.00 | 1.04 | -1.38 | -0.83 |
| 67 | D03 | 3696 | ITGB8 | NM_002214 | 301559 | 331625 | 1.03 | 1.01 | -1.08 | -0.65 |
| 67 | D04 | 9358 | ITGBL1 | NM_004791 | 290766 | 324762 | 0.99 | 0.99 | 0.00 | 0.38 |
| 67 | D05 | 9445 | ITM2B | NM_021999 | 284269 | 341891 | 0.97 | 1.05 | 0.08 | -1.02 |
| 67 | D06 | 160897 | ITR | NM_180989 | 290720 | 340182 | 0.99 | 1.04 | -0.24 | -0.19 |
| 67 | D07 | 50618 | ITSN2 | NM_006277 | 280020 | 328128 | 0.95 | 1.00 | 0.28 | 0.14 |
| 67 | D08 | 57338 | JPH3 | NM_020655 | 279443 | 325181 | 0.95 | 0.99 | -0.41 | 0.99 |
| 67 | D09 | 8690 | JRKL | NM_003772 | 281083 | 344479 | 0.96 | 1.05 | -0.01 | -1.62 |
| 67 | D10 | 3728 | JUP | NM_002230 | 267840 | 335849 | 0.91 | 1.03 | 0.71 | 0.21 |
| 67 | D11 | 3732 | KAI1 | NM_002231 | 267106 | 330764 | 0.91 | 1.01 | 0.68 | 0.61 |
| 67 | D12 | 7881 | KCNAB1 | NM_003471 | 292526 | 343335 | 1.00 | 1.05 | -1.07 | -0.92 |
| 67 | E01 | NA | neg | NA | 298714 | 322817 | 1.02 | 0.99 | -3.34 | 0.99 |
| 67 | E02 | NA | neg | NA | 282736 | 326071 | 0.96 | 1.00 | -1.38 | 0.54 |
| 67 | E03 | 23630 | KCNE1L | NM_012282 | 283433 | 322477 | 0.97 | 0.99 | -0.28 | 0.10 |
| 67 | E04 | 56479 | KCNQ5 | NM_019842 | 276875 | 317612 | 0.94 | 0.97 | 0.28 | 0.85 |
| 67 | E05 | 3792 | KEL | NM_000420 | 268863 | 319974 | 0.92 | 0.98 | 0.54 | 1.50 |
| 67 | E06 | 11081 | KERA | NM_007035 | 295954 | 338928 | 1.01 | 1.04 | -2.30 | -0.53 |
| 67 | E07 | 9684 | LRRC14 | NM_014665 | 262619 | 326473 | 0.90 | 1.00 | 0.98 | -0.14 |
| 67 | E08 | 23027 | KIAA0033 | XM_084530 | 260628 | 328142 | 0.89 | 1.00 | 0.47 | 0.07 |
| 67 | E09 | 23008 | KIAA0265 | NM_014997 | 254008 | 324141 | 0.87 | 0.99 | 1.88 | 0.67 |
| 67 | E10 | 9896 | KIAA0274 | NM_014845 | 275588 | 339701 | 0.94 | 1.04 | -1.66 | -0.83 |
| 67 | E11 | 9719 | KIAA0605 | NM_014694 | 274863 | 340004 | 0.94 | 1.04 | -1.68 | -1.18 |
| 67 | E12 | 23023 | KIAA0779 | NM_015008 | 285265 | 340177 | 0.97 | 1.04 | -1.60 | -1.00 |
| 67 | F01 | NA | NA | NA | 286432 | 309334 | 0.98 | 0.95 | -1.24 | 3.09 |
| 67 | F02 | NA | pos | NA | 126562 | 256720 | 0.43 | 0.78 | 18.30 | 10.36 |
| 67 | F03 | 23249 | KIAA0960 | XM_371877 | 285949 | 335021 | 0.97 | 1.02 | 0.01 | -1.39 |
| 67 | F04 | 23254 | KIAA1026 | XM_048825 | 289461 | 339987 | 0.99 | 1.04 | -0.66 | -2.00 |
| 67 | F05 | 22887 | FOXJ3 | NM_014947 | 279725 | 330636 | 0.95 | 1.01 | -0.19 | 0.27 |
| 67 | F06 | 57222 | KIAA1181 | NM_020462 | 282036 | 345235 | 0.96 | 1.06 | 0.00 | -1.17 |
| 67 | F07 | 57188 | ADAMTSL3 | NM_207517 | 280363 | 336930 | 0.96 | 1.03 | -0.59 | -1.35 |
| 67 | F08 | 54625 | KIAA1268 | XM_291055 | 264978 | 323160 | 0.90 | 0.99 | 0.54 | 1.00 |
| 67 | F09 | 27143 | PALD | XM_166125 | 275308 | 328202 | 0.94 | 1.00 | -0.13 | 0.35 |
| 67 | F10 | 57707 | KIAA1609 | NM_020947 | 262429 | 331680 | 0.89 | 1.01 | 0.54 | 0.51 |
| 67 | F11 | 57718 | KIAA1622 | NM_020958 | 259049 | 333439 | 0.88 | 1.02 | 0.84 | -0.03 |
| 67 | F12 | 80726 | KIAA1683 | XM_371146 | 270926 | 334417 | 0.92 | 1.02 | 0.74 | 0.03 |
| 67 | G01 | NA | neg | NA | 292523 | 328708 | 1.00 | 1.00 | -1.86 | 0.60 |
| 67 | G02 | NA | neg | NA | 284643 | 323308 | 0.97 | 0.99 | -0.89 | 1.34 |
| 67 | G03 | 84629 | KIAA1856 | XM_376567 | 287095 | 337175 | 0.98 | 1.03 | -0.01 | -1.51 |
| 67 | G04 | 115653 | KIR3DL3 | NM_153443 | 277768 | 325431 | 0.95 | 0.99 | 0.90 | 0.19 |
| 67 | G05 | 3814 | KISS1 | NM_002256 | 279142 | 326299 | 0.95 | 1.00 | 0.01 | 1.05 |
| 67 | G06 | 11279 | KLF8 | NM_007250 | 282072 | 335703 | 0.96 | 1.03 | 0.12 | 0.34 |
| 67 | G07 | 79682 | KLIP1 | NM_024629 | 278862 | 341834 | 0.95 | 1.05 | -0.28 | -1.84 |
| 67 | G08 | 10219 | KLRG1 | NM_005810 | 278388 | 332206 | 0.95 | 1.02 | -0.97 | -0.07 |

| | | | | | | | | | | |
|---|---|---|---|---|---|---|---|---|---|---|
| 67 | G09 | 3841 | KPNA5 | NM_002269 | 283045 | 341362 | 0.96 | 1.04 | -0.95 | -1.28 |
| 67 | G10 | 3843 | KPNB3 | NM_002271 | 256792 | 331047 | 0.88 | 1.01 | 1.36 | 0.79 |
| 67 | G11 | 83999 | KREMEN1 | NM_032045 | 266505 | 334311 | 0.91 | 1.02 | 0.06 | 0.03 |
| 67 | G12 | 79412 | KREMEN2 | NM_024507 | 283008 | 336261 | 0.96 | 1.03 | -0.61 | -0.03 |
| 67 | H01 | NA | NA | NA | 254374 | 262745 | 0.87 | 0.80 | 3.71 | 8.93 |
| 67 | H02 | NA | pos | NA | 96587 | 157112 | 0.33 | 0.48 | 23.00 | 23.53 |
| 67 | H03 | 3848 | KRT1 | NM_006121 | 285433 | 321310 | 0.97 | 0.98 | 1.10 | -0.10 |
| 67 | H04 | 3868 | KRT16 | NM_005557 | 295198 | 332567 | 1.01 | 1.02 | -0.33 | -1.57 |
| 67 | H05 | 3872 | KRT17 | NM_000422 | 273735 | 317990 | 0.93 | 0.97 | 1.57 | 1.42 |
| 67 | H06 | 3875 | KRT18 | NM_000224 | 298033 | 330598 | 1.02 | 1.01 | -0.93 | 0.26 |
| 67 | H07 | 3857 | KRT9 | NM_000226 | 294640 | 342790 | 1.00 | 1.05 | -1.30 | -2.76 |
| 67 | H08 | 3887 | KRTHB1 | NM_002281 | 282406 | 327877 | 0.96 | 1.00 | -0.56 | -0.25 |
| 67 | H09 | 3892 | KRTHB6 | NM_002284 | 280033 | 324734 | 0.95 | 0.99 | 0.33 | 0.23 |
| 67 | H10 | 3895 | KTN1 | NM_004986 | 285765 | 344967 | 0.97 | 1.05 | -1.28 | -1.92 |
| 67 | H11 | 3903 | LAIR1 | NM_002287 | 264649 | 328578 | 0.90 | 1.00 | 1.19 | 0.04 |
| 67 | H12 | 3904 | LAIR2 | NM_002288 | 280497 | 325852 | 0.96 | 1.00 | 0.61 | 0.62 |
| 68 | A01 | NA | pos | NA | 67637 | 77138 | 0.23 | 0.27 | 31.17 | 35.42 |
| 68 | A02 | NA | NA | NA | 251282 | 296827 | 0.86 | 1.02 | 5.33 | -2.62 |
| 68 | A03 | 3908 | LAMA2 | NM_000426 | 316465 | 304754 | 1.08 | 1.05 | -3.56 | -2.82 |
| 68 | A04 | 3910 | LAMA4 | NM_002290 | 279960 | 325261 | 0.95 | 1.12 | 1.81 | -7.55 |
| 68 | A05 | 3920 | LAMP2 | NM_002294 | 295278 | 285252 | 1.01 | 0.98 | 0.30 | 1.00 |
| 68 | A06 | 57189 | LCHN | XM_376683 | 293875 | 282232 | 1.00 | 0.97 | -0.57 | 0.00 |
| 68 | A07 | 3939 | LDHA | NM_005566 | 309603 | 286760 | 1.05 | 0.99 | -3.04 | -0.18 |
| 68 | A08 | 3945 | LDHB | NM_002300 | 290962 | 272473 | 0.99 | 0.94 | -0.30 | 1.61 |
| 68 | A09 | 3948 | LDHC | NM_002301 | 276057 | 278376 | 0.94 | 0.96 | 1.89 | 0.39 |
| 68 | A10 | 23641 | LDOC1 | NM_012317 | 271693 | 273355 | 0.92 | 0.94 | 1.28 | -0.11 |
| 68 | A11 | 79165 | LENG1 | NM_024316 | 282249 | 272527 | 0.96 | 0.94 | -1.20 | 0.00 |
| 68 | A12 | 79143 | LENG4 | NM_024298 | 272332 | 268433 | 0.93 | 0.93 | 1.75 | 1.74 |
| 68 | B01 | NA | neg | NA | 293198 | 302901 | 1.00 | 1.04 | -1.81 | -3.56 |
| 68 | B02 | NA | neg | NA | 267489 | 289502 | 0.91 | 1.00 | 1.81 | -1.24 |
| 68 | B03 | 114823 | LENG8 | NM_052925 | 281989 | 281207 | 0.96 | 0.97 | 0.06 | 1.36 |
| 68 | B04 | 23484 | LEPROTL1 | NM_015344 | 281858 | 279755 | 0.96 | 0.97 | 0.30 | 0.43 |
| 68 | B05 | 3955 | LFNG | XM_496845 | 284977 | 290945 | 0.97 | 1.00 | 0.51 | 0.12 |
| 68 | B06 | 3958 | LGALS3 | NM_002306 | 289977 | 277846 | 0.99 | 0.96 | -1.26 | 0.87 |
| 68 | B07 | 3963 | LGALS7 | NM_002307 | 286675 | 286450 | 0.98 | 0.99 | -1.05 | -0.02 |
| 68 | B08 | 9211 | LGI1 | NM_005097 | 268316 | 283736 | 0.91 | 0.98 | 1.65 | -0.23 |
| 68 | B09 | 10184 | LHFPL2 | NM_005779 | 285453 | 281193 | 0.97 | 0.97 | -0.67 | 0.01 |
| 68 | B10 | 3975 | LHX1 | NM_005568 | 273721 | 278655 | 0.93 | 0.96 | -0.24 | -0.92 |
| 68 | B11 | 3977 | LIFR | NM_002310 | 262536 | 273204 | 0.89 | 0.94 | 0.33 | -0.01 |
| 68 | B12 | 3980 | LIG3 | NM_002311 | 276279 | 283066 | 0.94 | 0.98 | -0.05 | -0.68 |
| 68 | C01 | NA | pos | NA | 74677 | 91780 | 0.25 | 0.32 | 28.02 | 32.53 |
| 68 | C02 | NA | NA | NA | 242534 | 239435 | 0.83 | 0.83 | 4.40 | 6.97 |
| 68 | C03 | 3982 | LIM2 | NM_030657 | 286826 | 285665 | 0.98 | 0.99 | -1.54 | 0.13 |
| 68 | C04 | 3987 | LIMS1 | NM_004987 | 277446 | 269611 | 0.94 | 0.93 | 0.00 | 1.73 |
| 68 | C05 | 3991 | LIPE | NM_005357 | 265570 | 300247 | 0.90 | 1.04 | 2.32 | -1.95 |
| 68 | C06 | 51599 | LISCH7 | NM_015925 | 265491 | NA | 0.90 | NA | 1.27 | NA |
| 68 | C07 | 4001 | LMNB1 | NM_005573 | 260737 | 266123 | 0.89 | 0.92 | 1.68 | 3.04 |
| 68 | C08 | 4004 | LMO1 | NM_002315 | 285549 | 266141 | 0.97 | 0.92 | -1.70 | 2.36 |
| 68 | C09 | 4005 | LMO2 | NM_005574 | 269483 | 286103 | 0.92 | 0.99 | 0.66 | -1.30 |
| 68 | C10 | 8543 | LMO4 | NM_006769 | 265496 | 275674 | 0.90 | 0.95 | 0.00 | -0.86 |
| 68 | C11 | 81607 | PVRL4 | NM_030916 | 259088 | 270497 | 0.88 | 0.93 | -0.10 | 0.00 |
| 68 | C12 | 124220 | LOC124220 | NM_145252 | 278225 | 288971 | 0.95 | 1.00 | -1.24 | -2.16 |
| 68 | D01 | NA | neg | NA | 324117 | 315189 | 1.10 | 1.09 | -6.38 | -6.45 |
| 68 | D02 | NA | neg | NA | 284805 | 290249 | 0.97 | 1.00 | -0.84 | -2.13 |
| 68 | D03 | 253039 | LOC253039 | BC052374 | 281259 | 281241 | 0.96 | 0.97 | -0.06 | 0.60 |
| 68 | D04 | 254571 | LOC254571 | XM_170783 | 293929 | 277796 | 1.00 | 0.96 | -1.62 | 0.01 |
| 68 | D05 | 55971 | LOC55971 | NM_018842 | 295041 | 289923 | 1.00 | 1.00 | -1.12 | -0.46 |
| 68 | D06 | 56901 | LOC56901 | NM_020142 | 278186 | 291501 | 0.95 | 1.01 | 0.19 | -2.25 |
| 68 | D07 | 80298 | LOC80298 | NM_025198 | 277656 | 273708 | 0.95 | 0.94 | 0.00 | 1.43 |
| 68 | D08 | 90557 | LOC90557 | NM_138770 | 278484 | 278917 | 0.95 | 0.96 | 0.00 | -0.15 |
| 68 | D09 | 90925 | LOC90925 | NM_175870 | 266790 | 276955 | 0.91 | 0.96 | 1.74 | -0.01 |
| 68 | D10 | 93380 | LOC93380 | NM_173470 | 268171 | 264357 | 0.91 | 0.91 | 0.32 | 0.80 |
| 68 | D11 | 94431 | LOC94431 | NM_145237 | 259743 | 258137 | 0.88 | 0.89 | 0.51 | 1.84 |
| 68 | D12 | 4013 | LOH11CR2A | NM_014622 | 293107 | 297941 | 1.00 | 1.03 | -2.63 | -4.01 |
| 68 | E01 | NA | neg | NA | 313943 | 310135 | 1.07 | 1.07 | -5.64 | -6.25 |
| 68 | E02 | NA | neg | NA | 406059 | 283810 | 1.38 | 0.98 | -18.60 | -1.69 |
| 68 | E03 | 4026 | LPP | NM_005578 | 267650 | 279254 | 0.91 | 0.96 | 1.16 | 0.26 |
| 68 | E04 | 64748 | LPPR2 | NM_022737 | 277538 | 278044 | 0.94 | 0.96 | 0.00 | -0.71 |
| 68 | E05 | 9404 | LPXN | NM_004811 | 289498 | 275381 | 0.99 | 0.95 | -1.03 | 1.38 |
| 68 | E06 | 987 | LRBA | NM_006726 | 270384 | 299230 | 0.92 | 1.03 | 0.59 | -4.27 |
| 68 | E07 | 55367 | LRDD | NM_018494 | 268718 | 263780 | 0.91 | 0.91 | 0.57 | 2.47 |
| 68 | E08 | 26018 | LRIG1 | NM_015541 | 273534 | 274027 | 0.93 | 0.95 | 0.00 | 0.02 |
| 68 | E09 | 4033 | LRMP | NM_006152 | 274453 | 264295 | 0.93 | 0.91 | -0.03 | 1.50 |
| 68 | E10 | 26020 | LRP10 | NM_014045 | 269677 | 275332 | 0.92 | 0.95 | -0.58 | -1.78 |
| 68 | E11 | 84918 | LRP11 | NM_032832 | 247318 | 264922 | 0.84 | 0.91 | 1.56 | -0.01 |
| 68 | E12 | 10234 | LRRC17 | NM_005824 | 291251 | 275950 | 0.99 | 0.95 | -3.06 | -0.89 |
| 68 | F01 | NA | NA | NA | 299328 | 313261 | 1.02 | 1.08 | -2.75 | -6.38 |
| 68 | F02 | NA | pos | NA | 90303 | 151333 | 0.31 | 0.52 | 26.66 | 21.65 |
| 68 | F03 | 4048 | LTA4H | NM_000895 | 276847 | 283909 | 0.94 | 0.98 | 0.70 | -0.13 |
| 68 | F04 | 4054 | LTBP3 | NM_021070 | 283898 | 276400 | 0.97 | 0.95 | -0.07 | -0.01 |
| 68 | F05 | 4056 | LTC4S | NM_000897 | 294570 | 278534 | 1.00 | 0.96 | -0.92 | 1.24 |
| 68 | F06 | 51747 | LUC7A | NM_016424 | 269866 | 279052 | 0.92 | 0.96 | 1.49 | -0.37 |
| 68 | F07 | 4066 | LYL1 | NM_005583 | 278867 | 281616 | 0.95 | 0.97 | -0.03 | -0.21 |
| 68 | F08 | 4069 | LYZ | NM_000239 | 279217 | 276378 | 0.95 | 0.95 | 0.03 | 0.02 |
| 68 | F09 | 54585 | LZTFL1 | NM_020347 | 279888 | 276067 | 0.95 | 0.95 | 0.03 | -0.13 |
| 68 | F10 | 8216 | LZTR1 | NM_006767 | 281274 | 265709 | 0.96 | 0.92 | -1.39 | 0.29 |
| 68 | F11 | 4081 | MAB21L1 | NM_005584 | 266024 | 263532 | 0.91 | 0.91 | -0.24 | 0.64 |
| 68 | F12 | 10586 | MAB21L2 | NM_006439 | 275042 | 266445 | 0.94 | 0.92 | 0.05 | 1.17 |

| | | | | | | | | | | |
|---|---|---|---|---|---|---|---|---|---|---|
| 68 | G01 | NA | neg | NA | 294409 | 289515 | 1.00 | 1.00 | -1.59 | -2.17 |
| 68 | G02 | NA | neg | NA | 285225 | 282175 | 0.97 | 0.97 | -0.29 | -0.90 |
| 68 | G03 | 8379 | MAD1L1 | NM_003550 | 295571 | 286625 | 1.01 | 0.99 | -1.46 | -0.50 |
| 68 | G04 | 4085 | MAD2L1 | NM_002358 | 276112 | 270277 | 0.94 | 0.93 | 1.50 | 1.15 |
| 68 | G05 | 10459 | MAD2L2 | NM_006341 | 293553 | 286997 | 1.00 | 0.99 | -0.30 | -0.12 |
| 68 | G06 | 4093 | SMAD9 | NM_005905 | 301070 | 276837 | 1.02 | 0.95 | -2.42 | 0.12 |
| 68 | G07 | 139081 | MAGEC3 | NM_138702 | 271817 | 284154 | 0.93 | 0.98 | 1.43 | -0.54 |
| 68 | G08 | 9500 | MAGED1 | NM_006986 | 287809 | 283136 | 0.98 | 0.98 | -0.70 | -1.05 |
| 68 | G09 | 28986 | MAGEH1 | NM_014061 | 284181 | 260320 | 0.97 | 0.90 | -0.10 | 2.70 |
| 68 | G10 | 9794 | MAML1 | NM_014757 | 268891 | 264079 | 0.92 | 0.91 | 0.83 | 0.68 |
| 68 | G11 | 4123 | MAN2C1 | NM_006715 | 266974 | 270684 | 0.91 | 0.93 | 0.10 | -0.50 |
| 68 | G12 | 4129 | MAOB | NM_000898 | 265578 | 269857 | 0.90 | 0.93 | 1.85 | 0.68 |
| 68 | H01 | NA | NA | NA | 254852 | 254715 | 0.87 | 0.88 | 4.45 | 4.82 |
| 68 | H02 | NA | pos | NA | 76977 | 119316 | 0.26 | 0.41 | 29.47 | 28.27 |
| 68 | H03 | 4082 | MARCKS | NM_002356 | 284713 | 290244 | 0.97 | 1.00 | 0.53 | -0.16 |
| 68 | H04 | 51151 | MATP | NM_016180 | 300068 | 320070 | 1.02 | 1.10 | -1.40 | -6.50 |
| 68 | H05 | 4149 | MAX | NM_002382 | 289935 | 297171 | 0.99 | 1.03 | 0.67 | -0.91 |
| 68 | H06 | 85509 | MBD3L1 | NM_145208 | 288471 | 278711 | 0.98 | 0.96 | -0.19 | 0.76 |
| 68 | H07 | 54799 | MBTD1 | NM_017643 | 285339 | 286488 | 0.97 | 0.99 | 0.00 | 0.02 |
| 68 | H08 | 28985 | MCTS1 | NM_014060 | 281501 | 282754 | 0.96 | 0.98 | 0.65 | -0.02 |
| 68 | H09 | 9656 | MDC1 | NM_014641 | 287579 | 285426 | 0.98 | 0.98 | -0.11 | -0.68 |
| 68 | H10 | 266727 | MDGA1 | NM_153487 | 278085 | 272934 | 0.95 | 0.94 | 0.00 | 0.11 |
| 68 | H11 | 56890 | MDM1 | NM_017440 | 276245 | 272731 | 0.94 | 0.94 | -0.74 | 0.12 |
| 68 | H12 | 112950 | MED8 | NM_052877 | 264560 | 258259 | 0.90 | 0.89 | 2.46 | 3.66 |
| 69 | A01 | NA | pos | NA | 61589 | 70319 | 0.21 | 0.26 | 26.57 | 28.58 |
| 69 | A02 | NA | NA | NA | 277932 | 275687 | 0.94 | 1.03 | -0.39 | -0.47 |
| 69 | A03 | 4211 | MEIS1 | NM_002398 | 326895 | 300126 | 1.10 | 1.12 | -4.86 | -3.18 |
| 69 | A04 | 4218 | RAB8A | NM_005370 | 283336 | 287964 | 0.96 | 1.08 | 0.19 | -1.14 |
| 69 | A05 | 4225 | MEP1B | NM_005925 | 272558 | 270845 | 0.92 | 1.01 | 0.50 | 1.82 |
| 69 | A06 | 59274 | MESDC1 | NM_022566 | 278817 | 279950 | 0.94 | 1.05 | -0.13 | -0.24 |
| 69 | A07 | 23184 | MESDC2 | XM_370880 | 297098 | 267633 | 1.00 | 1.00 | -3.33 | 0.43 |
| 69 | A08 | 4242 | MFNG | NM_002405 | 298763 | 271280 | 1.01 | 1.01 | -2.98 | -0.24 |
| 69 | A09 | 8972 | MGAM | NM_004668 | 261394 | 266183 | 0.88 | 0.99 | 0.13 | -0.21 |
| 69 | A10 | 84302 | C9ORF125 | NM_032342 | 261299 | 256977 | 0.88 | 0.96 | 1.30 | 1.61 |
| 69 | A11 | 91433 | MGC14386 | XM_370908 | 267752 | 268095 | 0.90 | 1.00 | -0.18 | 0.14 |
| 69 | A12 | 79791 | FBXO31 | NM_024735 | 263331 | 259631 | 0.89 | 0.97 | 1.42 | 2.01 |
| 69 | B01 | NA | neg | NA | 297092 | 261911 | 1.00 | 0.98 | -2.66 | 0.53 |
| 69 | B02 | NA | neg | NA | 277150 | 266257 | 0.94 | 0.99 | -0.18 | -0.08 |
| 69 | B03 | 93436 | MGC19595 | NM_033415 | 366561 | 266003 | 1.24 | 0.99 | -9.68 | 0.70 |
| 69 | B04 | 84787 | MGC2705 | NM_032701 | 285631 | 277438 | 0.96 | 1.04 | 0.02 | -0.60 |
| 69 | B05 | 3444 | IFNA7 | NM_021057 | 280156 | 288091 | 0.95 | 1.08 | -0.33 | -1.57 |
| 69 | B06 | 10290 | APEG1 | NM_005876 | 279209 | 270691 | 0.94 | 1.01 | -0.06 | 0.12 |
| 69 | B07 | 115106 | CCDC5 | NM_138443 | 277086 | 261877 | 0.94 | 0.98 | -0.71 | 0.30 |
| 69 | B08 | 196051 | PPAPDC1 | XM_113641 | 275855 | 279111 | 0.93 | 1.04 | -0.01 | -2.29 |
| 69 | B09 | 3150 | HMGN1 | NM_004965 | 252097 | 269691 | 0.85 | 1.01 | 1.41 | -1.65 |
| 69 | B10 | 10473 | HMGN4 | NM_006353 | 272662 | 262520 | 0.92 | 0.98 | 0.01 | -0.12 |
| 69 | B11 | 3166 | HMX1 | NM_018942 | 250054 | 245750 | 0.84 | 0.92 | 2.15 | 2.36 |
| 69 | B12 | 3172 | HNF4A | NM_000457 | 260662 | 264247 | 0.88 | 0.99 | 1.87 | 0.42 |
| 69 | C01 | NA | pos | NA | 70088 | 128686 | 0.24 | 0.48 | 23.70 | 19.41 |
| 69 | C02 | NA | NA | NA | 234399 | 246926 | 0.79 | 0.92 | 3.22 | 2.68 |
| 69 | C03 | 3178 | HNRPA1 | NM_031157 | 290765 | 270624 | 0.98 | 1.01 | -2.16 | 0.08 |
| 69 | C04 | 3190 | HNRPK | NM_002140 | 295609 | 258971 | 1.00 | 0.97 | -3.15 | 2.04 |
| 69 | C05 | 3232 | HOXD3 | NM_006898 | 259408 | 286547 | 0.88 | 1.07 | 0.33 | -1.32 |
| 69 | C06 | 3240 | HP | NM_005143 | 258253 | 272328 | 0.87 | 1.02 | 0.62 | -0.08 |
| 69 | C07 | 3242 | HPD | NM_002150 | 243863 | 262335 | 0.82 | 0.98 | 1.50 | 0.27 |
| 69 | C08 | 3251 | HPRT1 | NM_000194 | 263549 | 259347 | 0.89 | 0.97 | -0.40 | 0.53 |
| 69 | C09 | 3257 | HPS1 | NM_000195 | 247851 | 273681 | 0.84 | 1.02 | 0.01 | -2.18 |
| 69 | C10 | 84343 | HPS3 | NM_032383 | 257296 | 279243 | 0.87 | 1.04 | -0.01 | -2.45 |
| 69 | C11 | 79803 | HPS6 | NM_024747 | 249652 | 255686 | 0.84 | 0.95 | 0.27 | 0.98 |
| 69 | C12 | 57110 | HRASLS | NM_020386 | 289025 | 270374 | 0.98 | 1.01 | -3.59 | -0.42 |
| 69 | D01 | NA | neg | NA | 304445 | 278088 | 1.03 | 1.04 | -3.56 | -0.88 |
| 69 | D02 | NA | neg | NA | 286894 | 282198 | 0.97 | 1.05 | -1.37 | -1.47 |
| 69 | D03 | 3267 | HRB | NM_004504 | 274184 | 284459 | 0.93 | 1.06 | 1.85 | -1.04 |
| 69 | D04 | 3270 | HRC | NM_002152 | 287410 | 277933 | 0.97 | 1.04 | -0.18 | 0.20 |
| 69 | D05 | 8739 | HRK | NM_003806 | 284790 | 282774 | 0.96 | 1.06 | -0.89 | 0.06 |
| 69 | D06 | 55802 | HSA275986 | NM_018403 | 279177 | 278012 | 0.94 | 1.04 | -0.04 | -0.04 |
| 69 | D07 | 55566 | HSAJ2425 | NM_017532 | 260338 | 262475 | 0.88 | 0.98 | 1.39 | 1.09 |
| 69 | D08 | 3283 | HSD3B1 | NM_000862 | 272007 | 276106 | 0.92 | 1.03 | 0.49 | -1.00 |
| 69 | D09 | 3284 | HSD3B2 | NM_000198 | 263257 | 256485 | 0.89 | 0.96 | 0.03 | 1.09 |
| 69 | D10 | 11077 | HSF2BP | NM_007031 | 263348 | 265741 | 0.89 | 0.99 | 1.19 | 0.30 |
| 69 | D11 | 3303 | HSPA1A | NM_005345 | 272885 | 283935 | 0.92 | 1.06 | -0.68 | -2.17 |
| 69 | D12 | 3315 | HSPB1 | NM_001540 | 283546 | 285274 | 0.96 | 1.07 | -0.96 | -1.69 |
| 69 | E01 | NA | neg | NA | 307791 | 264339 | 1.04 | 0.99 | -5.35 | 0.60 |
| 69 | E02 | NA | neg | NA | 295073 | 267991 | 1.00 | 1.00 | -3.76 | 0.09 |
| 69 | E03 | 8988 | HSPB3 | NM_006308 | 269911 | 274419 | 0.91 | 1.02 | 1.01 | -0.08 |
| 69 | E04 | 23640 | HSPBP1 | NM_012267 | 259361 | 271710 | 0.88 | 1.01 | 1.94 | 0.62 |
| 69 | E05 | 25852 | HSPC056 | NM_014154 | 276854 | 264686 | 0.94 | 0.99 | -1.27 | 2.16 |
| 69 | E06 | 29090 | HSPC154 | NM_014177 | 267556 | 285627 | 0.90 | 1.07 | 0.04 | -1.58 |
| 69 | E07 | 3336 | HSPE1 | NM_002157 | 260769 | 268803 | 0.88 | 1.00 | -0.03 | -0.27 |
| 69 | E08 | 54727 | HSSUCCDH | X53943 | 261842 | 263123 | 0.88 | 0.98 | 0.39 | 0.38 |
| 69 | E09 | 3344 | HTLF | NM_002158 | 261311 | 252213 | 0.88 | 0.94 | -1.09 | 1.24 |
| 69 | E10 | 3347 | HTN3 | NM_000200 | 261623 | 266425 | 0.88 | 0.99 | 0.03 | -0.25 |
| 69 | E11 | 84513 | HTPAP | NM_032483 | 259350 | 266362 | 0.88 | 0.99 | -0.36 | -0.14 |
| 69 | E12 | 3364 | HUS1 | NM_004507 | 283321 | 269755 | 0.96 | 1.01 | -2.30 | 0.05 |
| 69 | F01 | NA | NA | NA | 269631 | 272492 | 0.91 | 1.02 | -0.78 | -1.23 |
| 69 | F02 | NA | pos | NA | 88705 | 121212 | 0.30 | 0.45 | 21.77 | 20.17 |
| 69 | F03 | 135458 | HUS1B | NM_148959 | 273373 | 261260 | 0.92 | 0.98 | 0.39 | 1.11 |
| 69 | F04 | 29072 | HYPB | NM_014159 | 270308 | 272755 | 0.91 | 1.02 | 0.39 | -0.20 |

| | | | | | | | | | | |
|---|---|---|---|---|---|---|---|---|---|---|
| 69 | F05 | 25766 | HYPC | NM_012272 | 281253 | 266676 | 0.95 | 1.00 | -2.00 | 1.20 |
| 69 | F06 | 11153 | HYPE | NM_007076 | 261560 | 269244 | 0.88 | 1.01 | 0.60 | 0.06 |
| 69 | F07 | 25998 | IBTK | NM_015525 | 258769 | 270696 | 0.87 | 1.01 | 0.03 | -1.21 |
| 69 | F08 | 29851 | ICOS | NM_012092 | 263410 | 261430 | 0.89 | 0.98 | 0.01 | -0.06 |
| 69 | F09 | 3397 | ID1 | NM_002165 | 251075 | 240538 | 0.85 | 0.90 | -0.01 | 2.21 |
| 69 | F10 | 3398 | ID2 | NM_002166 | 267491 | 257480 | 0.90 | 0.96 | -0.89 | 0.33 |
| 69 | F11 | 3416 | IDE | NM_004969 | 270816 | 263103 | 0.91 | 0.98 | -1.98 | -0.36 |
| 69 | F12 | 3422 | IDI1 | NM_004508 | 270826 | 273949 | 0.91 | 1.02 | -0.93 | -1.22 |
| 69 | G01 | NA | neg | NA | 293940 | 280966 | 0.99 | 1.05 | -3.11 | -1.81 |
| 69 | G02 | NA | neg | NA | 308126 | 267677 | 1.04 | 1.00 | -4.88 | 0.07 |
| 69 | G03 | 25836 | IDN3 | NM_015384 | 285296 | 281463 | 0.96 | 1.05 | -0.39 | -1.14 |
| 69 | G04 | 8870 | IER3 | NM_003897 | 279247 | 266770 | 0.94 | 1.00 | -0.02 | 1.26 |
| 69 | G05 | 3429 | IFI27 | NM_005532 | 266902 | 282282 | 0.90 | 1.05 | 0.48 | -0.40 |
| 69 | G06 | 10561 | IFI44 | NM_006417 | 287116 | 273693 | 0.97 | 1.02 | -1.89 | 0.04 |
| 69 | G07 | 3434 | IFIT1 | NM_001548 | 262438 | 278700 | 0.89 | 1.04 | 0.27 | -1.73 |
| 69 | G08 | 3433 | IFIT2 | NM_001547 | 268830 | 256597 | 0.91 | 0.96 | 0.03 | 1.24 |
| 69 | G09 | 3437 | IFIT4 | NM_001549 | 271543 | 259020 | 0.92 | 0.97 | -1.86 | 0.21 |
| 69 | G10 | 24138 | IFIT5 | NM_012420 | 279064 | 269596 | 0.94 | 1.01 | -1.63 | -0.77 |
| 69 | G11 | 10581 | IFITM2 | NM_006435 | 237773 | 240596 | 0.80 | 0.90 | 2.84 | 3.44 |
| 69 | G12 | 10410 | IFITM3 | NM_021034 | 261480 | 269982 | 0.88 | 1.01 | 0.93 | -0.05 |
| 69 | H01 | NA | NA | NA | 231262 | 215650 | 0.78 | 0.81 | 6.05 | 7.85 |
| 69 | H02 | NA | pos | NA | 87310 | 111115 | 0.29 | 0.41 | 23.99 | 22.63 |
| 69 | H03 | 3439 | IFNA1 | NM_024013 | 272888 | 266960 | 0.92 | 1.00 | 2.50 | 1.33 |
| 69 | H04 | 3460 | IFNGR2 | NM_005534 | 295688 | 291864 | 1.00 | 1.09 | -0.73 | -1.87 |
| 69 | H05 | 3475 | IFRD1 | NM_001550 | 271969 | 282852 | 0.92 | 1.06 | 1.20 | -0.06 |
| 69 | H06 | 64108 | IFRG28 | NM_022147 | 281356 | 268709 | 0.95 | 1.00 | 0.18 | 1.17 |
| 69 | H07 | 51214 | IGF2AS | NM_016412 | 276863 | 277479 | 0.94 | 1.04 | -0.18 | -1.14 |
| 69 | H08 | 3482 | IGF2R | NM_000876 | 283103 | 267936 | 0.96 | 1.00 | -0.40 | 0.06 |
| 69 | H09 | 3502 | IGHG3 | AK090464 | 277713 | 268712 | 0.94 | 1.00 | -1.28 | -0.74 |
| 69 | H10 | 92806 | MGC16385 | NM_145039 | 279760 | 266326 | 0.94 | 0.99 | -0.37 | 0.12 |
| 69 | H11 | 5915 | RARB | NM_000965 | 269925 | 270909 | 0.91 | 1.01 | 0.18 | -0.43 |
| 69 | H12 | 7498 | XDH | NM_000379 | 269097 | 264481 | 0.91 | 0.99 | 1.33 | 1.15 |
| 70 | A01 | NA | pos | NA | 76938 | 130877 | 0.25 | 0.44 | 31.03 | 23.58 |
| 70 | A02 | NA | NA | NA | 285327 | 307556 | 0.94 | 1.04 | 2.60 | -1.27 |
| 70 | A03 | 348035 | MGC40069 | NM_182615 | 335448 | 310681 | 1.11 | 1.05 | -4.16 | -1.78 |
| 70 | A04 | 54537 | FAM35A | NM_019054 | 338523 | 318976 | 1.12 | 1.08 | -3.19 | -1.88 |
| 70 | A05 | 65009 | NDRG4 | NM_020465 | 297428 | 332961 | 0.98 | 1.13 | 1.68 | -3.83 |
| 70 | A06 | 4725 | NDUFS5 | NM_004552 | 318674 | 302086 | 1.05 | 1.02 | -0.52 | -0.28 |
| 70 | A07 | 4728 | NDUFS8 | NM_002496 | 306629 | 292841 | 1.01 | 0.99 | -0.05 | 0.87 |
| 70 | A08 | 4747 | NEFL | NM_006158 | 300710 | 289604 | 0.99 | 0.98 | 0.00 | 0.28 |
| 70 | A09 | 79661 | NEIL1 | NM_024608 | 290678 | 292514 | 0.96 | 0.99 | 0.30 | -0.46 |
| 70 | A10 | 10276 | NET1 | NM_005863 | 273008 | 282851 | 0.90 | 0.96 | 3.17 | 0.86 |
| 70 | A11 | 4794 | NFKBIE | NM_004556 | 287077 | 284843 | 0.95 | 0.96 | -0.01 | 0.75 |
| 70 | A12 | 4795 | NFKBIL1 | NM_005007 | 299640 | 275437 | 0.99 | 0.93 | 0.56 | 3.94 |
| 70 | B01 | NA | neg | NA | 304417 | 312293 | 1.00 | 1.06 | -3.00 | -5.06 |
| 70 | B02 | NA | neg | NA | 301065 | 291847 | 0.99 | 0.99 | -2.54 | -2.18 |
| 70 | B03 | 4798 | NFRKB | NM_006165 | 286808 | 270424 | 0.95 | 0.92 | -0.52 | 0.77 |
| 70 | B04 | 27018 | NGFRAP1 | NM_014380 | 295553 | 287722 | 0.97 | 0.97 | -0.33 | -0.60 |
| 70 | B05 | 64149 | NJMU-R1 | NM_022344 | 288284 | 283484 | 0.95 | 0.96 | -0.06 | 0.02 |
| 70 | B06 | 9235 | NK4 | NM_004221 | 292042 | 271791 | 0.96 | 0.92 | 0.11 | 0.87 |
| 70 | B07 | 4818 | NKG7 | NM_005601 | 294921 | 281613 | 0.97 | 0.95 | -1.45 | -0.67 |
| 70 | B08 | 4820 | NKTR | NM_005385 | 278953 | 277576 | 0.92 | 0.94 | -0.02 | -1.15 |
| 70 | B09 | 9241 | NOG | NM_005450 | 269328 | 267610 | 0.89 | 0.91 | 0.22 | -0.07 |
| 70 | B10 | 4839 | NOL1 | NM_006170 | 252281 | 240029 | 0.83 | 0.81 | 3.00 | 3.76 |
| 70 | B11 | 4842 | NOS1 | NM_000620 | 264924 | 258876 | 0.87 | 0.88 | 0.02 | 1.29 |
| 70 | B12 | 4846 | NOS3 | NM_000603 | 275093 | 281357 | 0.91 | 0.95 | 0.92 | -0.01 |
| 70 | C01 | NA | pos | NA | 75526 | 156609 | 0.25 | 0.53 | 27.17 | 17.32 |
| 70 | C02 | NA | NA | NA | 250993 | 229045 | 0.83 | 0.78 | 3.23 | 7.13 |
| 70 | C03 | 4862 | NPAS2 | NM_002518 | 275321 | 278899 | 0.91 | 0.94 | -0.01 | 0.05 |
| 70 | C04 | 10577 | NPC2 | NM_006432 | 291395 | 281411 | 0.96 | 0.95 | -0.82 | 0.76 |
| 70 | C05 | 7827 | NPHS2 | NM_014625 | 269312 | 287051 | 0.89 | 0.97 | 1.47 | -0.02 |
| 70 | C06 | 4869 | NPM1 | NM_002520 | 268972 | 281194 | 0.89 | 0.95 | 2.20 | 0.02 |
| 70 | C07 | 10641 | TUSC4 | NM_006545 | 276210 | 272281 | 0.91 | 0.92 | 0.05 | 1.12 |
| 70 | C08 | 4884 | NPTX1 | NM_002522 | 275949 | 262565 | 0.91 | 0.89 | -0.67 | 1.44 |
| 70 | C09 | 4835 | NQO2 | NM_000904 | 271367 | 275248 | 0.89 | 0.93 | -1.11 | -0.68 |
| 70 | C10 | 9542 | NRG2 | NM_004883 | 264642 | 280755 | 0.87 | 0.95 | 0.26 | -1.50 |
| 70 | C11 | 83714 | NRIP2 | NM_031474 | 257303 | 271727 | 0.85 | 0.92 | 0.01 | -0.05 |
| 70 | C12 | 64710 | NUCKS | NM_022731 | 297285 | 291760 | 0.98 | 0.99 | -3.17 | -1.01 |
| 70 | D01 | NA | neg | NA | 324942 | 299103 | 1.07 | 1.01 | -5.94 | -3.07 |
| 70 | D02 | NA | neg | NA | 280175 | 284851 | 0.92 | 0.96 | 0.17 | -1.07 |
| 70 | D03 | 10726 | NUDC | NM_006600 | 282040 | 285558 | 0.93 | 0.97 | 0.00 | -1.23 |
| 70 | D04 | 318 | NUDT2 | NM_001161 | 284859 | 269269 | 0.94 | 0.91 | 0.99 | 2.12 |
| 70 | D05 | 8650 | NUMB | NM_003744 | 286791 | 288817 | 0.95 | 0.98 | 0.00 | -0.61 |
| 70 | D06 | 8021 | NUP214 | NM_005085 | 265415 | 278396 | 0.87 | 0.94 | 3.61 | 0.07 |
| 70 | D07 | 51203 | NUSAP1 | NM_016359 | 278050 | 278158 | 0.92 | 0.94 | 0.72 | -0.05 |
| 70 | D08 | 60506 | NYX | NM_022567 | 277805 | 269834 | 0.92 | 0.91 | 0.00 | 0.07 |
| 70 | D09 | 23090 | ZNF423 | NM_015069 | 266299 | 267474 | 0.88 | 0.91 | 0.50 | 0.07 |
| 70 | D10 | 54741 | OBRGRP | NM_017526 | 278549 | 267097 | 0.92 | 0.90 | -0.72 | 0.08 |
| 70 | D11 | 84033 | OBSCN | NM_052843 | 278781 | 271015 | 0.92 | 0.92 | -2.00 | -0.29 |
| 70 | D12 | 4950 | OCLN | NM_002538 | 294153 | 294786 | 0.97 | 1.00 | -1.82 | -1.78 |
| 70 | E01 | NA | neg | NA | 302470 | 313027 | 1.00 | 1.06 | -3.20 | -4.68 |
| 70 | E02 | NA | neg | NA | 304349 | 268649 | 1.00 | 0.91 | -3.46 | 1.57 |
| 70 | E03 | 4952 | OCRL | NM_000276 | 277086 | 279654 | 0.91 | 0.95 | 0.34 | -0.05 |
| 70 | E04 | 55714 | ODZ3 | XM_371717 | 354324 | 289892 | 1.17 | 0.98 | -8.81 | -0.42 |
| 70 | E05 | 8481 | OFD1 | NM_003611 | 295379 | 273183 | 0.97 | 0.92 | -1.50 | 1.95 |
| 70 | E06 | 167826 | OLIG3 | NM_175747 | 344377 | 286556 | 1.14 | 0.97 | -7.49 | -0.72 |
| 70 | E07 | 80207 | OPA3 | NM_025136 | 254544 | 270915 | 0.84 | 0.92 | 3.60 | 1.32 |
| 70 | E08 | 10133 | OPTN | NM_021980 | 262951 | 271193 | 0.87 | 0.92 | 1.69 | 0.23 |

| | | | | | | | | | | |
|---|---|---|---|---|---|---|---|---|---|---|
| 70 | E09 | 393046 | OR2A5 | NM_012365 | 269997 | 251217 | 0.89 | 0.85 | -0.34 | 2.72 |
| 70 | E10 | 391632 | OR7E35P | AF073924 | 261433 | 270790 | 0.86 | 0.92 | 1.29 | -0.08 |
| 70 | E11 | 26494 | OR8G1P | NM_001002905 | 258537 | 271164 | 0.85 | 0.92 | 0.43 | 0.04 |
| 70 | E12 | 114884 | OSBPL10 | NM_017784 | 306734 | 287891 | 1.01 | 0.97 | -3.86 | -0.45 |
| 70 | F01 | NA | NA | NA | 299854 | 294336 | 0.99 | 1.00 | -3.16 | -3.37 |
| 70 | F02 | NA | pos | NA | 110124 | 166888 | 0.36 | 0.56 | 22.73 | 14.56 |
| 70 | F03 | 146183 | OTOA | NM_144672 | 277283 | 285610 | 0.91 | 0.97 | 0.00 | -2.21 |
| 70 | F04 | 9381 | OTOF | NM_004802 | 272374 | 274513 | 0.90 | 0.93 | 2.06 | 0.42 |
| 70 | F05 | 56914 | OTOR | NM_020157 | 282122 | 279957 | 0.93 | 0.95 | 0.00 | -0.33 |
| 70 | F06 | 124641 | OVCA2 | NM_080822 | 273084 | 269488 | 0.90 | 0.91 | 1.92 | 0.35 |
| 70 | F07 | 23533 | P101-PI3K | NM_014308 | 270763 | 270527 | 0.89 | 0.92 | 1.07 | 0.05 |
| 70 | F08 | 5026 | P2RX5 | NM_002561 | 257292 | 265749 | 0.85 | 0.90 | 2.15 | -0.32 |
| 70 | F09 | 9127 | P2RXL1 | NM_005446 | 266529 | 255591 | 0.88 | 0.87 | -0.18 | 0.78 |
| 70 | F10 | 55578 | P38IP | NM_017569 | 270449 | 270268 | 0.89 | 0.91 | -0.26 | -1.33 |
| 70 | F11 | 10130 | TXNDC7 | NM_005742 | 264890 | 262376 | 0.87 | 0.89 | -0.75 | -0.04 |
| 70 | F12 | 83638 | P5326 | NM_031450 | 276602 | 259227 | 0.91 | 0.88 | -0.07 | 2.26 |
| 70 | G01 | NA | neg | NA | 304432 | 305246 | 1.00 | 1.03 | -3.85 | -4.48 |
| 70 | G02 | NA | neg | NA | 290112 | 280258 | 0.96 | 0.95 | -1.89 | -0.96 |
| 70 | G03 | 8106 | PABPN1 | NM_004643 | 269500 | 270532 | 0.89 | 0.92 | 1.00 | 0.34 |
| 70 | G04 | 5048 | PAFAH1B1 | NM_000430 | 282803 | 270589 | 0.93 | 0.92 | 0.56 | 1.40 |
| 70 | G05 | 10606 | PAICS | NM_006452 | 278140 | 273469 | 0.92 | 0.93 | 0.47 | 1.01 |
| 70 | G06 | 24145 | PANX1 | NM_015368 | 289665 | 275140 | 0.95 | 0.93 | -0.41 | -0.02 |
| 70 | G07 | 152559 | PAQR3 | NM_177453 | 278634 | 282682 | 0.92 | 0.96 | -0.07 | -1.23 |
| 70 | G08 | 11315 | PARK7 | NM_007262 | 270666 | 266960 | 0.89 | 0.90 | 0.26 | -0.07 |
| 70 | G09 | 5076 | PAX2 | NM_000278 | 286803 | 271853 | 0.95 | 0.92 | -3.01 | -1.08 |
| 70 | G10 | 5080 | PAX6 | NM_000280 | 274251 | 259417 | 0.90 | 0.88 | -0.84 | 0.62 |
| 70 | G11 | 22976 | PAXIP1L | NM_007349 | 271029 | 269534 | 0.89 | 0.91 | -1.66 | -0.62 |
| 70 | G12 | 5037 | PBP | NM_002567 | 275125 | 278218 | 0.91 | 0.94 | 0.07 | 0.01 |
| 70 | H01 | NA | NA | NA | 259628 | 265188 | 0.86 | 0.90 | 2.89 | 2.35 |
| 70 | H02 | NA | pos | NA | 112995 | 148120 | 0.37 | 0.50 | 22.89 | 18.82 |
| 70 | H03 | 5092 | PCBD | NM_000281 | 264052 | 265589 | 0.87 | 0.90 | 2.36 | 2.23 |
| 70 | H04 | 27253 | PCDH17 | NM_014459 | 289109 | 293807 | 0.95 | 0.99 | 0.33 | -0.67 |
| 70 | H05 | 54510 | PCDH18 | NM_019035 | 288570 | 285572 | 0.95 | 0.97 | -0.33 | 0.50 |
| 70 | H06 | 5101 | PCDH9 | NM_020403 | 292062 | 289710 | 0.96 | 0.98 | -0.11 | -0.87 |
| 70 | H07 | 54825 | PC-LKC | NM_017675 | 285533 | 285935 | 0.94 | 0.97 | -0.39 | -0.49 |
| 70 | H08 | 23424 | TDRD7 | NM_014290 | 289921 | 289250 | 0.96 | 0.98 | -1.74 | -2.01 |
| 70 | H09 | 11333 | PDAP1 | NM_014891 | 267997 | 266021 | 0.88 | 0.90 | 0.18 | 0.93 |
| 70 | H10 | 11235 | PDCD10 | NM_007217 | 280826 | 280185 | 0.93 | 0.95 | -1.12 | -1.10 |
| 70 | H11 | 27250 | PDCD4 | NM_014456 | 262583 | 267059 | 0.87 | 0.90 | 0.12 | 0.92 |
| 70 | H12 | 9141 | PDCD5 | NM_004708 | 266192 | 271462 | 0.88 | 0.92 | 1.91 | 2.16 |
| 71 | A01 | NA | pos | NA | 51952 | 46560 | 0.20 | 0.17 | 18.44 | 19.15 |
| 71 | A02 | NA | NA | NA | 172977 | 174993 | 0.67 | 0.64 | 6.38 | 6.35 |
| 71 | A03 | 10015 | PDCD6IP | NM_013374 | 259211 | 261936 | 1.01 | 0.96 | -1.10 | -1.30 |
| 71 | A04 | 5145 | PDE6A | NM_000440 | 250027 | 245500 | 0.97 | 0.90 | -0.31 | 0.28 |
| 71 | A05 | 5158 | PDE6B | NM_000283 | 237305 | 250217 | 0.92 | 0.91 | 0.39 | -0.11 |
| 71 | A06 | 5146 | PDE6C | NM_006204 | 267398 | 255597 | 1.04 | 0.93 | -2.45 | -1.42 |
| 71 | A07 | 10455 | PECI | NM_006117 | 238544 | 260626 | 0.93 | 0.95 | -0.29 | -2.48 |
| 71 | A08 | 27043 | PELP1 | NM_014389 | 233987 | 254535 | 0.91 | 0.93 | -0.27 | -2.66 |
| 71 | A09 | 5184 | PEPD | NM_000285 | 213885 | 222209 | 0.83 | 0.81 | 1.52 | 0.42 |
| 71 | A10 | 64065 | PERP | NM_022121 | 229584 | 222669 | 0.89 | 0.81 | 0.22 | 0.38 |
| 71 | A11 | 23481 | PES1 | NM_014303 | 177461 | 186661 | 0.69 | 0.68 | 5.06 | 3.25 |
| 71 | A12 | 5189 | PEX1 | NM_000466 | 235468 | 245279 | 0.92 | 0.89 | 0.26 | 0.13 |
| 71 | B01 | NA | neg | NA | 281389 | 274400 | 1.10 | 1.00 | -6.70 | -5.41 |
| 71 | B02 | NA | neg | NA | 236202 | 209122 | 0.92 | 0.76 | -2.19 | 1.09 |
| 71 | B03 | 8800 | PEX11A | NM_003847 | 236061 | 256941 | 0.92 | 0.94 | -1.06 | -2.66 |
| 71 | B04 | 8799 | PEX11B | NM_003846 | 216582 | 237627 | 0.84 | 0.87 | 0.76 | -0.79 |
| 71 | B05 | 5195 | PEX14 | NM_004565 | 210602 | 209839 | 0.82 | 0.77 | 0.78 | 2.06 |
| 71 | B06 | 9409 | PEX16 | NM_004813 | 209436 | 236438 | 0.82 | 0.86 | 1.07 | -1.37 |
| 71 | B07 | 8504 | PEX3 | NM_003630 | 210799 | 227515 | 0.82 | 0.83 | 0.21 | -1.03 |
| 71 | B08 | 5199 | PFC | NM_002621 | 203948 | 202692 | 0.79 | 0.74 | 0.46 | 0.65 |
| 71 | B09 | 5202 | PFDN2 | NM_012394 | 210737 | 181795 | 0.82 | 0.66 | -0.44 | 2.59 |
| 71 | B10 | 5216 | PFN1 | NM_005022 | 211687 | 206776 | 0.82 | 0.75 | -0.27 | 0.10 |
| 71 | B11 | 5224 | PGAM2 | NM_000290 | 209885 | 189181 | 0.82 | 0.69 | -0.44 | 1.14 |
| 71 | B12 | 267004 | PGBD3 | NM_170753 | 229983 | 228837 | 0.90 | 0.83 | -1.46 | -0.09 |
| 71 | C01 | NA | pos | NA | 67528 | 73254 | 0.26 | 0.27 | 15.35 | 15.07 |
| 71 | C02 | NA | NA | NA | 190866 | 203494 | 0.74 | 0.74 | 3.05 | 2.10 |
| 71 | C03 | 27306 | PGDS | NM_014485 | 238024 | 231559 | 0.93 | 0.84 | -0.53 | 0.32 |
| 71 | C04 | 10857 | PGRMC1 | NM_006667 | 213445 | 243579 | 0.83 | 0.89 | 1.79 | -0.94 |
| 71 | C05 | 54681 | PH-4 | NM_017732 | 208545 | 223703 | 0.81 | 0.82 | 1.71 | 1.12 |
| 71 | C06 | 10077 | PHEMX | NM_005705 | 197736 | 201411 | 0.77 | 0.73 | 2.96 | 2.57 |
| 71 | C07 | 7262 | PHLDA2 | NM_003311 | 189195 | 197818 | 0.74 | 0.72 | 3.09 | 2.37 |
| 71 | C08 | 23612 | PHLDA3 | NM_012396 | 210482 | 222286 | 0.82 | 0.81 | 0.54 | -0.86 |
| 71 | C09 | 57157 | PHTF2 | NM_020432 | 228061 | 220653 | 0.89 | 0.80 | -1.44 | -0.84 |
| 71 | C10 | 5264 | PHYH | NM_006214 | 236178 | 210007 | 0.92 | 0.77 | -1.98 | 0.23 |
| 71 | C11 | 8554 | PIAS1 | NM_016166 | 221848 | 207408 | 0.86 | 0.76 | -0.91 | -0.23 |
| 71 | C12 | 27124 | PIB5PA | NM_014422 | 231855 | 240348 | 0.90 | 0.88 | -0.92 | -0.79 |
| 71 | D01 | NA | neg | NA | 259621 | 277329 | 1.01 | 1.01 | -3.35 | -5.15 |
| 71 | D02 | NA | neg | NA | 244295 | 260879 | 0.95 | 0.95 | -1.82 | -3.52 |
| 71 | D03 | 118788 | PIK3AP1 | NM_152309 | 240500 | 228176 | 0.94 | 0.83 | -0.33 | 0.76 |
| 71 | D04 | 5301 | PIN1L | NM_006222 | 237439 | 239041 | 0.92 | 0.87 | -0.15 | -0.38 |
| 71 | D05 | 54545 | PIP3AP | NM_019061 | 234119 | 232045 | 0.91 | 0.85 | -0.39 | 0.40 |
| 71 | D06 | 5306 | PITPN | NM_006224 | 231284 | 223731 | 0.90 | 0.82 | 0.06 | 0.45 |
| 71 | D07 | 9271 | PIWIL1 | NM_004764 | 235282 | 246182 | 0.92 | 0.90 | -1.06 | -2.34 |
| 71 | D08 | 55124 | PIWIL2 | NM_018068 | 220921 | 211608 | 0.86 | 0.77 | -0.06 | 0.31 |
| 71 | D09 | 63876 | PKNOX2 | NM_022062 | 217308 | 235421 | 0.85 | 0.86 | 0.08 | -2.21 |
| 71 | D10 | 8399 | PLA2G10 | NM_003561 | 205779 | 217982 | 0.80 | 0.80 | 1.50 | -0.46 |
| 71 | D11 | 8681 | PLA2G4B | NM_005090 | 212754 | 224651 | 0.83 | 0.82 | 0.44 | -1.84 |
| 71 | D12 | 8605 | PLA2G4C | NM_003706 | 223287 | 228020 | 0.87 | 0.83 | 0.38 | 0.55 |

| | | | | | | | | | | |
|---|---|---|---|---|---|---|---|---|---|---|
| 71 | E01 | NA | neg | NA | 281949 | 283731 | 1.10 | 1.03 | -5.19 | -5.14 |
| 71 | E02 | NA | neg | NA | 254144 | 261351 | 0.99 | 0.95 | -2.42 | -2.91 |
| 71 | E03 | 22925 | PLA2R1 | NM_007366 | 229240 | 248545 | 0.89 | 0.91 | 1.18 | -0.61 |
| 71 | E04 | 5330 | PLCB2 | NM_004573 | 243950 | 232906 | 0.95 | 0.85 | -0.41 | 0.89 |
| 71 | E05 | 5341 | PLEK | NM_002664 | 229816 | 245457 | 0.89 | 0.90 | 0.43 | -0.28 |
| 71 | E06 | 26499 | PLEK2 | NM_016445 | 242237 | 239313 | 0.94 | 0.87 | -0.64 | -0.45 |
| 71 | E07 | 10979 | PLEKHC1 | NM_006832 | 215322 | 193583 | 0.84 | 0.71 | 1.32 | 3.55 |
| 71 | E08 | 90480 | GADD45GIP1 | NM_052850 | 234515 | 219702 | 0.91 | 0.80 | -1.02 | 0.16 |
| 71 | E09 | 5354 | PLP1 | NM_000533 | 220384 | 205613 | 0.86 | 0.75 | 0.16 | 1.42 |
| 71 | E10 | 5361 | PLXNA1 | NM_032242 | 226857 | 220928 | 0.88 | 0.81 | -0.22 | -0.10 |
| 71 | E11 | 57671 | PLXNA4 | XM_039393 | 215791 | 213662 | 0.84 | 0.78 | 0.53 | -0.09 |
| 71 | E12 | 23420 | PM5 | NM_014287 | 238143 | 239204 | 0.93 | 0.87 | -0.71 | 0.09 |
| 71 | F01 | NA | NA | NA | 166507 | 170093 | 0.65 | 0.62 | 6.10 | 5.57 |
| 71 | F02 | NA | pos | NA | 83784 | 150141 | 0.33 | 0.55 | 14.35 | 7.56 |
| 71 | F03 | 5366 | PMAIP1 | NM_021127 | 235105 | 233670 | 0.92 | 0.85 | 0.38 | 0.25 |
| 71 | F04 | 5375 | PMP2 | NM_002677 | 236125 | 234194 | 0.92 | 0.85 | 0.15 | 0.14 |
| 71 | F05 | 5376 | PMP22 | NM_000304 | 237555 | 244926 | 0.92 | 0.89 | -0.56 | -0.85 |
| 71 | F06 | 9240 | PNMA1 | NM_006029 | 219385 | 211772 | 0.85 | 0.77 | 1.42 | 1.68 |
| 71 | F07 | 5423 | POLB | NM_002690 | 228444 | 221945 | 0.89 | 0.81 | -0.21 | 0.11 |
| 71 | F08 | 661 | POLR3D | NM_001722 | 223539 | 216783 | 0.87 | 0.79 | -0.15 | -0.16 |
| 71 | F09 | 25812 | POM121L1 | NM_014348 | 197562 | 208711 | 0.77 | 0.76 | 2.22 | 0.49 |
| 71 | F10 | 22932 | POMZP3 | NM_152992 | 255127 | 215455 | 0.99 | 0.79 | -3.26 | -0.17 |
| 71 | F11 | 5458 | POU4F2 | NM_004575 | 229605 | 208128 | 0.89 | 0.76 | -1.07 | -0.16 |
| 71 | F12 | 5460 | POU5F1 | NM_002701 | 219148 | 239484 | 0.85 | 0.87 | 0.97 | -0.56 |
| 71 | G01 | NA | neg | NA | 275440 | 284229 | 1.07 | 1.04 | -5.11 | -5.47 |
| 71 | G02 | NA | neg | NA | 231836 | 273970 | 0.90 | 1.00 | -0.76 | -4.45 |
| 71 | G03 | 5463 | POU6F1 | XM_370693 | 232137 | 237562 | 0.90 | 0.87 | 0.33 | 0.20 |
| 71 | G04 | 23532 | PRAME | NM_006115 | 231878 | 240443 | 0.90 | 0.88 | 0.23 | -0.14 |
| 71 | G05 | 5542 | PRB1 | NM_005039 | 239182 | 241765 | 0.93 | 0.88 | -1.07 | -0.20 |
| 71 | G06 | 23082 | PPRC1 | NM_015062 | 230754 | 217455 | 0.90 | 0.79 | -0.06 | 1.45 |
| 71 | G07 | 9055 | PRC1 | NM_003981 | 214368 | 210610 | 0.83 | 0.77 | 0.85 | 1.58 |
| 71 | G08 | 5546 | PRCC | NM_005973 | 217875 | 209080 | 0.85 | 0.76 | 0.07 | 0.94 |
| 71 | G09 | 56981 | PRDM11 | NM_020229 | 224875 | 226843 | 0.88 | 0.83 | -0.85 | -0.98 |
| 71 | G10 | 59336 | PRDM13 | NM_021620 | 200747 | 222268 | 0.78 | 0.81 | 1.82 | -0.52 |
| 71 | G11 | 93166 | PRDM6 | XM_049619 | 225729 | 208961 | 0.88 | 0.76 | -1.03 | 0.09 |
| 71 | G12 | 56978 | PRDM8 | NM_020226 | 227997 | 245124 | 0.89 | 0.89 | -0.26 | -0.78 |
| 71 | H01 | NA | NA | NA | 134234 | 163854 | 0.52 | 0.60 | 10.92 | 8.71 |
| 71 | H02 | NA | pos | NA | 70955 | 155747 | 0.28 | 0.57 | 17.23 | 9.52 |
| 71 | H03 | 5552 | PRG1 | NM_002727 | 240213 | 263518 | 0.94 | 0.96 | 1.48 | -0.20 |
| 71 | H04 | 10216 | PRG4 | NM_005807 | 274294 | 245801 | 1.07 | 0.90 | -2.05 | 1.51 |
| 71 | H05 | 5564 | PRKAB1 | NM_006253 | 255799 | 260654 | 1.00 | 0.95 | -0.77 | 0.11 |
| 71 | H06 | 5565 | PRKAB2 | NM_005399 | 260257 | 263622 | 1.01 | 0.96 | -1.05 | -0.97 |
| 71 | H07 | 51422 | PRKAG2 | NM_016203 | 244938 | 249466 | 0.95 | 0.91 | -0.25 | -0.11 |
| 71 | H08 | 23613 | PRKCBP1 | NM_012408 | 237597 | 243913 | 0.92 | 0.89 | 0.06 | -0.35 |
| 71 | H09 | 112464 | PRKCDBP | NM_145040 | 236746 | 243133 | 0.92 | 0.89 | -0.08 | -0.42 |
| 71 | H10 | 23627 | PRND | NM_012409 | 214719 | 230062 | 0.84 | 0.84 | 2.38 | 0.89 |
| 71 | H11 | 5621 | PRNP | NM_000311 | 229685 | 227207 | 0.89 | 0.83 | 0.53 | 0.46 |
| 71 | H12 | 29028 | ATAD2 | NM_014109 | 224101 | 226457 | 0.87 | 0.83 | 2.08 | 3.26 |
| 72 | A01 | NA | pos | NA | 52240 | 56015 | 0.19 | 0.23 | 23.39 | 23.25 |
| 72 | A02 | NA | NA | NA | 207052 | 164501 | 0.77 | 0.66 | 3.80 | 9.10 |
| 72 | A03 | 8842 | PROM1 | NM_006017 | 294313 | 265104 | 1.09 | 1.07 | -6.01 | -4.00 |
| 72 | A04 | 5629 | PROX1 | NM_002763 | 262896 | 257837 | 0.97 | 1.04 | -2.35 | -3.10 |
| 72 | A05 | 26121 | PRPF31 | NM_015629 | 242706 | 217881 | 0.90 | 0.88 | 0.91 | 2.36 |
| 72 | A06 | 27128 | PSCD4 | NM_013385 | 236993 | 276120 | 0.88 | 1.11 | 0.16 | -4.64 |
| 72 | A07 | 5664 | PSEN2 | NM_000447 | 278197 | 238586 | 1.03 | 0.96 | -5.34 | -1.23 |
| 72 | A08 | 5683 | PSMA2 | NM_002787 | 221162 | 229728 | 0.82 | 0.92 | 1.15 | 0.49 |
| 72 | A09 | 10213 | PSMD14 | NM_005805 | 167650 | 160459 | 0.62 | 0.64 | 6.77 | 8.57 |
| 72 | A10 | 5710 | PSMD4 | NM_002810 | 238962 | 228762 | 0.89 | 0.92 | -1.61 | -0.13 |
| 72 | A11 | 5711 | PSMD5 | NM_005047 | 225408 | 233331 | 0.83 | 0.94 | -0.16 | 0.13 |
| 72 | A12 | 5713 | PSMD7 | NM_002811 | 172748 | 184130 | 0.64 | 0.74 | 8.93 | 8.27 |
| 72 | B01 | NA | neg | NA | 284020 | 261237 | 1.05 | 1.05 | -8.78 | -5.73 |
| 72 | B02 | NA | neg | NA | 234816 | 228872 | 0.87 | 0.92 | -2.55 | -1.51 |
| 72 | B03 | 5714 | PSMD8 | NM_002812 | 205802 | 202917 | 0.76 | 0.82 | 2.34 | 1.91 |
| 72 | B04 | 9051 | PSTPIP1 | NM_003978 | 222245 | 209273 | 0.82 | 0.84 | -0.06 | 1.03 |
| 72 | B05 | 9050 | PSTPIP2 | NM_024430 | 211397 | 211301 | 0.78 | 0.85 | 2.02 | 1.01 |
| 72 | B06 | 5725 | PTBP1 | NM_002819 | 215303 | 240856 | 0.80 | 0.97 | 0.06 | -2.25 |
| 72 | B07 | 171558 | PTCRA | NM_138296 | 199949 | 229714 | 0.74 | 0.92 | 1.72 | -2.27 |
| 72 | B08 | 23210 | PTDSR | NM_015167 | 213028 | 216941 | 0.79 | 0.87 | -0.67 | -0.04 |
| 72 | B09 | 10408 | MYCNOS | NM_006316 | 220721 | 207173 | 0.82 | 0.83 | -2.79 | 0.27 |
| 72 | B10 | 158131 | OR1Q1 | NM_012364 | 208568 | 210515 | 0.77 | 0.85 | -0.62 | 0.04 |
| 72 | B11 | 23370 | ARHGEF18 | NM_015318 | 200647 | 218420 | 0.74 | 0.88 | 0.12 | -0.13 |
| 72 | B12 | 23203 | PMPCA | NM_015160 | 238069 | 240769 | 0.88 | 0.97 | -2.18 | -1.33 |
| 72 | C01 | NA | pos | NA | 54435 | 78000 | 0.20 | 0.31 | 21.42 | 18.59 |
| 72 | C02 | NA | NA | NA | 191913 | NA | 0.71 | NA | 4.03 | NA |
| 72 | C03 | 196383 | MGC7036 | NM_145058 | 233932 | 210136 | 0.87 | 0.84 | -0.06 | 1.39 |
| 72 | C04 | 121642 | MGC90512 | NM_001001655 | 234333 | 220411 | 0.87 | 0.89 | -0.43 | 0.00 |
| 72 | C05 | 11343 | MGLL | NM_007283 | 241904 | 225393 | 0.90 | 0.91 | -0.67 | -0.41 |
| 72 | C06 | 4261 | MHC2TA | NM_000246 | 244552 | 223347 | 0.91 | 0.90 | -2.48 | 0.45 |
| 72 | C07 | 29919 | C18ORF8 | NM_013326 | 199802 | 215895 | 0.74 | 0.87 | 2.89 | -0.05 |
| 72 | C08 | 9645 | MICAL2 | NM_014632 | 201778 | 231924 | 0.75 | 0.93 | 1.91 | -1.58 |
| 72 | C09 | 4282 | MIF | NM_002415 | 207836 | 214469 | 0.77 | 0.86 | 0.00 | -0.27 |
| 72 | C10 | 145282 | MIPOL1 | NM_138731 | 199914 | 206215 | 0.74 | 0.83 | 1.64 | 1.02 |
| 72 | C11 | 4287 | MJD | NM_004993 | 210813 | 208953 | 0.78 | 0.84 | 0.00 | 1.52 |
| 72 | C12 | 8079 | MLF2 | NM_005439 | 226505 | 233787 | 0.84 | 0.94 | 0.45 | 0.00 |
| 72 | D01 | NA | neg | NA | 275244 | 273727 | 1.02 | 1.10 | -6.12 | -7.01 |
| 72 | D02 | NA | neg | NA | 264716 | 231973 | 0.98 | 0.93 | -4.79 | -1.56 |
| 72 | D03 | 4298 | MLLT1 | NM_005934 | 236502 | 223747 | 0.88 | 0.90 | 0.00 | -0.46 |
| 72 | D04 | 65108 | MLP | NM_023009 | 230740 | 224632 | 0.85 | 0.90 | 0.41 | -0.62 |

| | | | | | | | | | | |
|---|---|---|---|---|---|---|---|---|---|---|
| 72 | D05 | 326625 | MMAB | NM_052845 | 244371 | 221762 | 0.91 | 0.89 | -0.60 | 0.00 |
| 72 | D06 | 4311 | MME | NM_000902 | 238780 | 214858 | 0.88 | 0.86 | -1.37 | 1.50 |
| 72 | D07 | 4323 | MMP14 | NM_004995 | 228291 | NA | 0.85 | NA | -0.33 | NA |
| 72 | D08 | 22915 | MMRN1 | NM_007351 | 229498 | 212633 | 0.85 | 0.85 | -1.21 | 0.87 |
| 72 | D09 | 4330 | MN1 | NM_002430 | 206722 | 223665 | 0.77 | 0.90 | 0.53 | -1.53 |
| 72 | D10 | 55034 | MOCOS | NM_017947 | 200758 | 212301 | 0.74 | 0.85 | 1.92 | 0.16 |
| 72 | D11 | 22877 | MONDOA | NM_014938 | 213839 | 226398 | 0.79 | 0.91 | 0.00 | -0.82 |
| 72 | D12 | 4345 | MOX2 | NM_005944 | 220388 | 220182 | 0.82 | 0.88 | 1.60 | 1.71 |
| 72 | E01 | NA | neg | NA | 286241 | 271549 | 1.06 | 1.09 | -9.01 | -7.05 |
| 72 | E02 | NA | neg | NA | 236750 | 236536 | 0.88 | 0.95 | -2.75 | -2.48 |
| 72 | E03 | 10200 | MPHOSPH6 | NM_005792 | 211813 | 211005 | 0.78 | 0.85 | 1.63 | 0.88 |
| 72 | E04 | 10198 | MPHOSPH9 | NM_022782 | 220779 | 214643 | 0.82 | 0.86 | 0.18 | 0.36 |
| 72 | E05 | 54852 | MPRG | NM_017705 | 232296 | 223595 | 0.86 | 0.90 | -0.57 | -0.56 |
| 72 | E06 | 4357 | MPST | NM_021126 | 216159 | 211969 | 0.80 | 0.85 | 0.00 | 1.55 |
| 72 | E07 | 25953 | MR-1 | NM_015488 | 213369 | 211829 | 0.79 | 0.85 | 0.07 | 0.09 |
| 72 | E08 | 10335 | MRVI1 | NM_006069 | 234219 | 217479 | 0.87 | 0.87 | -3.30 | -0.09 |
| 72 | E09 | 4437 | MSH3 | NM_002439 | 188640 | 211654 | 0.70 | 0.85 | 1.32 | -0.29 |
| 72 | E10 | 4478 | MSN | NM_002444 | 204386 | 204015 | 0.76 | 0.82 | -0.04 | 0.92 |
| 72 | E11 | 4500 | MT1L | NR_001447 | 208753 | 219795 | 0.77 | 0.88 | -0.85 | -0.28 |
| 72 | E12 | 27085 | MTBP | NM_022045 | 221226 | 236802 | 0.82 | 0.95 | 0.00 | -0.78 |
| 72 | F01 | NA | NA | NA | 136488 | 154805 | 0.51 | 0.62 | 10.64 | 7.32 |
| 72 | F02 | NA | pos | NA | 78567 | 123971 | 0.29 | 0.50 | 17.96 | 11.34 |
| 72 | F03 | 4515 | MTCP1 | NM_014221 | 230035 | 224300 | 0.85 | 0.90 | 0.03 | -1.72 |
| 72 | F04 | 4522 | MTHFD1 | NM_005956 | 226818 | 210737 | 0.84 | 0.85 | 0.12 | 0.00 |
| 72 | F05 | 4548 | MTR | NM_000254 | 219061 | 224693 | 0.81 | 0.90 | 1.81 | -1.57 |
| 72 | F06 | 4580 | MTX1 | NM_002455 | 221701 | 201820 | 0.82 | 0.81 | 0.00 | 2.01 |
| 72 | F07 | 4584 | MUC3A | XM_374502 | 220002 | 205850 | 0.81 | 0.83 | -0.07 | 0.00 |
| 72 | F08 | 4595 | MUTYH | NM_012222 | 278204 | 209841 | 1.03 | 0.84 | -8.16 | 0.04 |
| 72 | F09 | 4599 | MX1 | NM_002462 | 204634 | 197459 | 0.76 | 0.79 | 0.00 | 0.70 |
| 72 | F10 | 26292 | MYCBP | NM_012333 | 209370 | 209583 | 0.78 | 0.84 | 0.04 | -0.67 |
| 72 | F11 | 4633 | MYL2 | NM_000432 | 211710 | 227171 | 0.78 | 0.91 | -0.52 | -2.11 |
| 72 | F12 | 4634 | MYL3 | NM_000258 | 232470 | 224171 | 0.86 | 0.90 | -0.71 | 0.00 |
| 72 | G01 | NA | neg | NA | 276739 | 270469 | 1.03 | 1.09 | -6.53 | -6.24 |
| 72 | G02 | NA | neg | NA | 235265 | 232154 | 0.87 | 0.93 | -1.28 | -1.24 |
| 72 | G03 | 4636 | MYL5 | NM_002477 | 235254 | 219386 | 0.87 | 0.88 | -0.06 | 0.46 |
| 72 | G04 | 4637 | MYL6 | NM_021019 | 231864 | 236228 | 0.86 | 0.95 | 0.06 | -1.79 |
| 72 | G05 | 4668 | NAGA | NM_000262 | 233402 | 224399 | 0.86 | 0.90 | 0.57 | 0.00 |
| 72 | G06 | 22861 | NALP1 | NM_014922 | 218998 | 232436 | 0.81 | 0.93 | 0.92 | -0.45 |
| 72 | G07 | 126205 | NALP8 | NM_176811 | 226623 | 214256 | 0.84 | 0.86 | -0.33 | 0.44 |
| 72 | G08 | 338321 | NALP9 | NM_176820 | 205573 | 215559 | 0.76 | 0.87 | 1.60 | 0.83 |
| 72 | G09 | 79664 | NARG2 | NM_024611 | 210046 | 217517 | 0.78 | 0.87 | -0.11 | -0.39 |
| 72 | G10 | 9 | NAT1 | NM_000662 | 215022 | 223302 | 0.80 | 0.90 | -0.10 | -0.93 |
| 72 | G11 | 24142 | NAT6 | NM_012191 | 206350 | 216070 | 0.76 | 0.87 | 0.73 | 0.87 |
| 72 | G12 | 4681 | NBL1 | NM_005380 | 242766 | 235930 | 0.90 | 0.95 | -1.44 | 0.00 |
| 72 | H01 | NA | NA | NA | 138771 | 157953 | 0.51 | 0.63 | 11.40 | 7.74 |
| 72 | H02 | NA | pos | NA | 60625 | 115940 | 0.22 | 0.47 | 21.28 | 13.22 |
| 72 | H03 | 10787 | NCKAP1 | NM_013436 | 238518 | 241964 | 0.88 | 0.97 | 0.00 | -3.19 |
| 72 | H04 | 57727 | NCOA5 | NM_020967 | 240367 | 216844 | 0.89 | 0.87 | -0.55 | 0.04 |
| 72 | H05 | 135112 | NCOA7 | NM_181782 | 252564 | 218292 | 0.94 | 0.88 | -1.38 | 0.10 |
| 72 | H06 | 9436 | NCR2 | NM_004828 | 245526 | 243004 | 0.91 | 0.98 | -1.96 | -2.53 |
| 72 | H07 | 54820 | NDE1 | NM_017668 | 191912 | 189302 | 0.71 | 0.76 | 4.54 | 3.00 |
| 72 | H08 | 81565 | NDEL1 | NM_030808 | 216666 | 217256 | 0.80 | 0.87 | 0.67 | -0.09 |
| 72 | H09 | 10241 | NDP52 | NM_005831 | 215122 | 202770 | 0.80 | 0.81 | -0.28 | 0.84 |
| 72 | H10 | 10397 | NDRG1 | NM_006096 | 214933 | 211150 | 0.80 | 0.85 | 0.38 | -0.04 |
| 72 | H11 | 57447 | NDRG2 | NM_016250 | 214462 | 214736 | 0.79 | 0.86 | 0.18 | 0.35 |
| 72 | H12 | 7499 | XG | NM_175569 | 235138 | 233874 | 0.87 | 0.94 | 0.00 | -0.43 |
| 73 | A01 | NA | pos | NA | 53922 | 52326 | 0.22 | 0.20 | 20.45 | 14.93 |
| 73 | A02 | NA | NA | NA | 141772 | 184017 | 0.58 | 0.71 | 11.16 | 4.39 |
| 73 | A03 | 9536 | PTGES | NM_004878 | 259849 | 232288 | 1.06 | 0.90 | -0.41 | 0.42 |
| 73 | A04 | 5757 | PTMA | NM_002823 | 236021 | 246203 | 0.96 | 0.95 | 1.80 | -0.45 |
| 73 | A05 | 5940 | RBMY1A1 | NM_005058 | 248471 | 235199 | 1.02 | 0.91 | -0.09 | 0.07 |
| 73 | A06 | 5950 | RBP4 | NM_006744 | 278964 | 258745 | 1.14 | 1.00 | -1.07 | -0.88 |
| 73 | A07 | 11317 | RBPSUHL | NM_014276 | 255668 | 256991 | 1.04 | 0.99 | -0.37 | -1.11 |
| 73 | A08 | 80223 | RCP | NM_025151 | 233369 | 241203 | 0.95 | 0.93 | 0.86 | -0.07 |
| 73 | A09 | 5957 | RCV1 | NM_002903 | 224693 | 224057 | 0.92 | 0.87 | 0.45 | 0.45 |
| 73 | A10 | 9985 | REC8L1 | NM_005132 | 231354 | 221901 | 0.95 | 0.86 | -0.09 | 0.64 |
| 73 | A11 | 5965 | RECQL | NM_002907 | 231948 | 241681 | 0.95 | 0.93 | 0.07 | -1.34 |
| 73 | A12 | 56475 | REPRIMO | NM_019845 | 226457 | 231301 | 0.93 | 0.89 | 2.18 | 0.88 |
| 73 | B01 | NA | neg | NA | 245029 | 254043 | 1.00 | 0.98 | -3.37 | -1.99 |
| 73 | B02 | NA | neg | NA | 220667 | 225801 | 0.90 | 0.87 | -0.79 | 0.27 |
| 73 | B03 | 5986 | RFNG | BC050399 | 196671 | 227906 | 0.80 | 0.88 | 2.64 | -0.01 |
| 73 | B04 | 28984 | RGC32 | NM_014059 | 220699 | 218502 | 0.90 | 0.84 | -0.20 | 0.99 |
| 73 | B05 | 64283 | RGNEF | XM_371755 | 211536 | 194881 | 0.86 | 0.75 | 0.19 | 2.51 |
| 73 | B06 | 6005 | RHAG | NM_000324 | 196405 | 237589 | 0.80 | 0.92 | 4.03 | 0.03 |
| 73 | B07 | 6006 | RHCE | NM_020485 | 204697 | 213831 | 0.84 | 0.83 | 1.39 | 1.56 |
| 73 | B08 | 6007 | RHD | NM_016124 | 225379 | 229966 | 0.92 | 0.89 | -1.92 | 0.05 |
| 73 | B09 | 6009 | RHEB | NM_005614 | 215638 | 228308 | 0.88 | 0.88 | -2.21 | -0.67 |
| 73 | B10 | 9743 | RICS | NM_014715 | 220141 | 227764 | 0.90 | 0.88 | -2.52 | -0.61 |
| 73 | B11 | 65986 | ZBTB10 | NM_023929 | 220737 | 222164 | 0.90 | 0.86 | -2.36 | -0.56 |
| 73 | B12 | 55781 | RIOK2 | NM_018343 | 206609 | 317549 | 0.84 | 1.23 | 0.66 | -6.80 |
| 73 | C01 | NA | pos | NA | 56033 | 93405 | 0.23 | 0.36 | 13.62 | 6.88 |
| 73 | C02 | NA | NA | NA | 178289 | 193251 | 0.73 | 0.75 | 0.70 | -1.11 |
| 73 | C03 | 6017 | RLBP1 | NM_000326 | 215791 | 211978 | 0.88 | 0.82 | -2.36 | -2.72 |
| 73 | C04 | 6101 | RP1 | NM_006269 | 210762 | 231798 | 0.86 | 0.90 | -2.14 | -4.06 |
| 73 | C05 | 54165 | RP42 | NM_020640 | 229827 | 222417 | 0.94 | 0.86 | -4.73 | -3.67 |
| 73 | C06 | 57096 | RPGRIP1 | NM_020366 | 228425 | 199127 | 0.93 | 0.77 | -2.34 | -0.88 |
| 73 | C07 | 10900 | RPIP8 | NM_006695 | 200302 | 196212 | 0.82 | 0.76 | -1.13 | -1.01 |
| 73 | C08 | 6134 | RPL10 | NM_006013 | 132079 | 129209 | 0.54 | 0.50 | 4.96 | 4.13 |

| | | | | | | | | | | |
|---|---|---|---|---|---|---|---|---|---|---|
| 73 | C09 | 6135 | RPL11 | NM_000975 | 139918 | 155555 | 0.57 | 0.60 | 2.81 | 1.17 |
| 73 | C10 | 23521 | RPL13A | NM_012423 | 157402 | 136326 | 0.64 | 0.53 | 1.12 | 2.73 |
| 73 | C11 | 6161 | RPL32 | NM_000994 | 118805 | 126604 | 0.49 | 0.49 | 5.42 | 3.10 |
| 73 | C12 | 6167 | RPL37 | NM_000997 | 155690 | 171647 | 0.64 | 0.66 | 3.05 | 0.89 |
| 73 | D01 | NA | neg | NA | 260706 | 263191 | 1.07 | 1.02 | -10.46 | -7.81 |
| 73 | D02 | NA | neg | NA | 240034 | 230001 | 0.98 | 0.89 | -8.28 | -5.15 |
| 73 | D03 | 6168 | RPL37A | NM_000998 | 161851 | 156974 | 0.66 | 0.61 | 0.88 | 0.58 |
| 73 | D04 | 6125 | RPL5 | NM_000969 | 166565 | 185289 | 0.68 | 0.72 | 0.08 | -1.44 |
| 73 | D05 | 11102 | RPP14 | NM_007042 | 240736 | 228431 | 0.98 | 0.88 | -8.33 | -5.26 |
| 73 | D06 | 6217 | RPS16 | NM_001020 | 184323 | 143158 | 0.75 | 0.55 | -0.13 | 2.50 |
| 73 | D07 | 6223 | RPS19 | NM_001022 | 166388 | 176815 | 0.68 | 0.68 | 0.00 | -0.56 |
| 73 | D08 | 6232 | RPS27 | NM_001030 | NA | 179775 | NA | 0.70 | NA | -1.02 |
| 73 | D09 | 6235 | RPS29 | NM_001032 | 149430 | 146635 | 0.61 | 0.57 | -0.65 | 0.78 |
| 73 | D10 | 6191 | RPS4X | NM_001007 | 129834 | 120719 | 0.53 | 0.47 | 1.58 | 2.87 |
| 73 | D11 | 6203 | RPS9 | NM_001013 | 119223 | 137041 | 0.49 | 0.53 | 2.92 | 1.16 |
| 73 | D12 | 9125 | RQCD1 | NM_005444 | 218449 | 223683 | 0.89 | 0.86 | -6.03 | -4.38 |
| 73 | E01 | NA | neg | NA | 273906 | 267004 | 1.12 | 1.03 | -5.80 | -3.81 |
| 73 | E02 | NA | neg | NA | 244354 | 263897 | 1.00 | 1.02 | -2.68 | -3.56 |
| 73 | E03 | 58528 | RRAGD | NM_021244 | 225066 | 210837 | 0.92 | 0.82 | 0.26 | 0.58 |
| 73 | E04 | 6251 | RSU1 | NM_012425 | 225350 | 214827 | 0.92 | 0.83 | -0.08 | 0.50 |
| 73 | E05 | 27156 | RTDR1 | NM_014433 | 213945 | 242679 | 0.87 | 0.94 | 0.55 | -2.09 |
| 73 | E06 | 23429 | RYBP | NM_012234 | 242165 | 216055 | 0.99 | 0.84 | -0.19 | 0.97 |
| 73 | E07 | 6261 | RYR1 | NM_000540 | 212972 | 222014 | 0.87 | 0.86 | 1.13 | 0.12 |
| 73 | E08 | 6281 | S100A10 | NM_002966 | 213236 | 219465 | 0.87 | 0.85 | -0.02 | 0.11 |
| 73 | E09 | 6283 | S100A12 | NM_005621 | 200420 | 224148 | 0.82 | 0.87 | 0.02 | -1.12 |
| 73 | E10 | 6276 | S100A5 | NM_002962 | 195752 | 222824 | 0.80 | 0.86 | 0.67 | -0.99 |
| 73 | E11 | 6278 | S100A7 | NM_002963 | 217879 | 206358 | 0.89 | 0.80 | -1.44 | -0.08 |
| 73 | E12 | 6288 | SAA1 | NM_000331 | 231257 | 238372 | 0.95 | 0.92 | -1.33 | -1.25 |
| 73 | F01 | NA | NA | NA | 158005 | 136853 | 0.65 | 0.53 | 6.85 | 6.57 |
| 73 | F02 | NA | pos | NA | 79186 | 128846 | 0.32 | 0.50 | 15.18 | 7.21 |
| 73 | F03 | 26278 | SACS | NM_014363 | 240267 | 217501 | 0.98 | 0.84 | -0.95 | 0.01 |
| 73 | F04 | 64092 | SAMSN1 | NM_022136 | 263735 | 220812 | 1.08 | 0.85 | -3.73 | -0.01 |
| 73 | F05 | 9092 | SART1 | NM_005146 | 192057 | 188389 | 0.78 | 0.73 | 3.27 | 2.22 |
| 73 | F06 | 6302 | SAS | NM_005981 | 232285 | 223144 | 0.95 | 0.86 | 1.26 | 0.37 |
| 73 | F07 | 51119 | SBDS | NM_016038 | 217845 | 224691 | 0.89 | 0.87 | 1.02 | -0.12 |
| 73 | F08 | 6311 | SCA2 | NM_002973 | 216276 | 221001 | 0.88 | 0.85 | 0.06 | -0.05 |
| 73 | F09 | 6315 | KLHL1AS | AF126749 | 202584 | 206559 | 0.83 | 0.80 | 0.19 | 0.25 |
| 73 | F10 | 10066 | SCAMP2 | NM_005697 | 211738 | 206623 | 0.87 | 0.80 | -0.62 | 0.27 |
| 73 | F11 | 22937 | SCAP | NM_012235 | 208673 | 212026 | 0.85 | 0.82 | -0.07 | -0.57 |
| 73 | F12 | 51435 | SCARA3 | NM_016240 | 223711 | 226703 | 0.91 | 0.88 | -0.13 | -0.35 |
| 73 | G01 | NA | neg | NA | 268439 | 268829 | 1.10 | 1.04 | -4.64 | -3.76 |
| 73 | G02 | NA | neg | NA | 230840 | 238164 | 0.94 | 0.92 | -0.67 | -1.31 |
| 73 | G03 | 950 | SCARB2 | NM_005506 | 235460 | 220637 | 0.96 | 0.85 | -0.26 | -0.01 |
| 73 | G04 | 8796 | SCEL | NM_003843 | 226540 | 223442 | 0.93 | 0.86 | 0.38 | 0.01 |
| 73 | G05 | 10648 | SCGB1D1 | NM_006552 | 232251 | 219787 | 0.95 | 0.85 | -0.80 | -0.07 |
| 73 | G06 | 117156 | SCGB3A2 | NM_054023 | 244667 | 236331 | 1.00 | 0.91 | 0.13 | -0.46 |
| 73 | G07 | 22955 | SCMH1 | NM_012236 | 229225 | 210545 | 0.94 | 0.81 | 0.00 | 1.24 |
| 73 | G08 | 10389 | SCML2 | NM_006089 | 218602 | 205506 | 0.89 | 0.79 | 0.00 | 1.42 |
| 73 | G09 | 6336 | SCN10A | NM_006514 | 206227 | 215759 | 0.84 | 0.83 | -0.02 | -0.25 |
| 73 | G10 | 11280 | SCN11A | NM_014139 | 206826 | 216648 | 0.85 | 0.84 | 0.09 | -0.30 |
| 73 | G11 | 6332 | SCN7A | NM_002976 | 213956 | 194207 | 0.87 | 0.75 | -0.45 | 1.09 |
| 73 | G12 | 6335 | SCN9A | NM_002977 | 223199 | 220834 | 0.91 | 0.85 | 0.10 | 0.35 |
| 73 | H01 | NA | NA | NA | 175798 | 202213 | 0.72 | 0.78 | 6.28 | 2.10 |
| 73 | H02 | NA | pos | NA | 77215 | 105144 | 0.32 | 0.41 | 16.70 | 9.87 |
| 73 | H03 | 6341 | SCO1 | NM_004589 | 232586 | 230006 | 0.95 | 0.89 | 1.18 | -0.23 |
| 73 | H04 | 6389 | SDHA | NM_004168 | 234396 | 228557 | 0.96 | 0.88 | 0.68 | 0.13 |
| 73 | H05 | 6392 | SDHD | NM_003002 | 234582 | 220839 | 0.96 | 0.85 | 0.09 | 0.38 |
| 73 | H06 | 55635 | DEPDC1 | NM_017779 | 233237 | 237651 | 0.95 | 0.92 | 2.47 | -0.03 |
| 73 | H07 | 10640 | SEC10L1 | NM_006544 | 241204 | 229792 | 0.99 | 0.89 | -0.13 | 0.23 |
| 73 | H08 | 25956 | SEC31L2 | NM_015490 | 230848 | 239911 | 0.94 | 0.93 | -0.16 | -0.80 |
| 73 | H09 | 29950 | SERTAD1 | NM_013376 | 221173 | 227170 | 0.90 | 0.88 | -0.46 | -0.63 |
| 73 | H10 | 6400 | SEL1L | NM_005065 | 223130 | 222884 | 0.91 | 0.86 | -0.50 | -0.27 |
| 73 | H11 | 6402 | SELL | NM_000655 | 213882 | 213527 | 0.87 | 0.83 | 0.69 | 0.08 |
| 73 | H12 | 10512 | SEMA3C | NM_006379 | 235902 | 226649 | 0.96 | 0.88 | -0.10 | 0.42 |
| 74 | A01 | NA | pos | NA | 52877 | 63231 | 0.21 | 0.24 | 21.13 | 20.08 |
| 74 | A02 | NA | NA | NA | 164082 | 185268 | 0.66 | 0.72 | 8.48 | 6.59 |
| 74 | A03 | 8293 | SERF1A | NM_021967 | 270217 | 269787 | 1.09 | 1.04 | -2.91 | -1.17 |
| 74 | A04 | 12 | SERPINA3 | NM_001085 | 239525 | 263469 | 0.96 | 1.02 | -0.12 | -0.94 |
| 74 | A05 | 5267 | SERPINA4 | NM_006215 | 237773 | 236666 | 0.96 | 0.91 | 0.16 | 1.15 |
| 74 | A06 | 27244 | SESN1 | NM_014454 | 234906 | 246241 | 0.95 | 0.95 | 0.79 | 0.18 |
| 74 | A07 | 9869 | SETDB1 | NM_012432 | 240594 | 280769 | 0.97 | 1.09 | -0.14 | -4.69 |
| 74 | A08 | 9169 | SFRS2IP | NM_004719 | 241999 | 266077 | 0.97 | 1.03 | -0.49 | -2.59 |
| 74 | A09 | 6435 | SFTPA1 | NM_005411 | 232329 | 218033 | 0.93 | 0.84 | -0.01 | 2.33 |
| 74 | A10 | 6439 | SFTPB | NM_000542 | 223691 | 229969 | 0.90 | 0.89 | 0.28 | 0.02 |
| 74 | A11 | 6440 | SFTPC | NM_003018 | 228710 | 242236 | 0.92 | 0.94 | 0.04 | -0.01 |
| 74 | A12 | 6442 | SGCA | NM_000023 | 236444 | 240823 | 0.95 | 0.93 | 0.33 | 0.92 |
| 74 | B01 | NA | neg | NA | 246996 | 281367 | 0.99 | 1.09 | -2.05 | -7.83 |
| 74 | B02 | NA | neg | NA | 237251 | 218672 | 0.95 | 0.85 | -0.94 | -0.90 |
| 74 | B03 | 6443 | SGCB | NM_000232 | 209105 | 225424 | 0.84 | 0.87 | 2.95 | -0.06 |
| 74 | B04 | 6444 | SGCD | NM_000337 | 212212 | 208586 | 0.85 | 0.81 | 1.89 | 1.33 |
| 74 | B05 | 6445 | SGCG | NM_000231 | 218908 | 194203 | 0.88 | 0.75 | 1.21 | 2.05 |
| 74 | B06 | 9047 | SH2D2A | NM_003975 | 231767 | 207822 | 0.93 | 0.80 | 0.06 | 0.63 |
| 74 | B07 | 10045 | SH2D3A | NM_005490 | 205638 | 203444 | 0.83 | 0.79 | 2.75 | 0.06 |
| 74 | B08 | 6452 | SH3BP2 | NM_003023 | 231184 | 205728 | 0.93 | 0.80 | -0.35 | 0.29 |
| 74 | B09 | 6455 | SH3GL1 | NM_003025 | 231277 | 222472 | 0.93 | 0.86 | -0.99 | -1.95 |
| 74 | B10 | 6457 | SH3GL3 | NM_003027 | 240592 | 214903 | 0.97 | 0.83 | -2.74 | -2.12 |
| 74 | B11 | 30011 | SH3KBP1 | NM_031892 | 222050 | 215497 | 0.89 | 0.83 | -0.29 | -0.85 |
| 74 | B12 | 9644 | SH3MD1 | XM_374831 | 230544 | 230578 | 0.93 | 0.89 | -0.09 | -1.74 |

| | | | | | | | | | | |
|---|---|---|---|---|---|---|---|---|---|---|
| 74 | C01 | NA | pos | NA | 59105 | 66688 | 0.24 | 0.26 | 18.57 | 17.45 |
| 74 | C02 | NA | NA | NA | 186311 | 189327 | 0.75 | 0.73 | 4.09 | 3.89 |
| 74 | C03 | 85358 | SHANK3 | XM_037493 | 231361 | 211302 | 0.93 | 0.82 | -0.34 | 3.04 |
| 74 | C04 | 25759 | SHC2 | XM_375550 | 219126 | 247153 | 0.88 | 0.96 | 0.34 | -1.39 |
| 74 | C05 | 6476 | SI | NM_001041 | 214707 | 216827 | 0.86 | 0.84 | 0.92 | 1.09 |
| 74 | C06 | 6491 | SIL | NM_003035 | 213524 | 191931 | 0.86 | 0.74 | 1.37 | 3.93 |
| 74 | C07 | 6490 | SILV | NM_006928 | 256098 | 213769 | 1.03 | 0.83 | -3.76 | 0.46 |
| 74 | C08 | 10326 | SIRPB1 | NM_006065 | 198495 | 224197 | 0.80 | 0.87 | 2.61 | -0.21 |
| 74 | C09 | 55423 | SIRPB2 | NM_018556 | 214926 | 217177 | 0.86 | 0.84 | 0.11 | 0.17 |
| 74 | C10 | 6497 | SKI | NM_003036 | 210997 | 214613 | 0.85 | 0.83 | -0.14 | -0.54 |
| 74 | C11 | 6498 | SKIL | NM_005414 | 231026 | 223320 | 0.93 | 0.86 | -2.08 | -0.18 |
| 74 | C12 | 6559 | SLC12A3 | NM_000339 | 244365 | 235853 | 0.98 | 0.91 | -2.42 | -0.78 |
| 74 | D01 | NA | neg | NA | 250061 | 282323 | 1.01 | 1.09 | -2.98 | -6.38 |
| 74 | D02 | NA | neg | NA | 273396 | 237533 | 1.10 | 0.92 | -5.64 | -1.43 |
| 74 | D03 | 5002 | SLC22A18 | NM_002555 | 228192 | 215418 | 0.92 | 0.83 | 0.19 | 2.59 |
| 74 | D04 | 291 | SLC25A4 | NM_001151 | 222599 | 235474 | 0.90 | 0.91 | 0.12 | -0.09 |
| 74 | D05 | 8501 | SLC43A1 | NM_003627 | 236088 | 227667 | 0.95 | 0.88 | -1.33 | -0.10 |
| 74 | D06 | 22950 | SLC4A1AP | NM_018158 | 235424 | 231166 | 0.95 | 0.89 | -0.95 | -0.39 |
| 74 | D07 | 6531 | SLC6A3 | NM_001044 | 232565 | 214581 | 0.94 | 0.83 | -0.90 | 0.38 |
| 74 | D08 | 6585 | SLIT1 | NM_003061 | 195849 | 193433 | 0.79 | 0.75 | 3.08 | 3.20 |
| 74 | D09 | 6594 | SMARCA1 | NM_003069 | 226392 | 230060 | 0.91 | 0.89 | -1.02 | -1.24 |
| 74 | D10 | 6598 | SMARCB1 | NM_003073 | 199174 | 205615 | 0.80 | 0.79 | 1.38 | 0.46 |
| 74 | D11 | 6599 | SMARCC1 | NM_003074 | 214935 | 221850 | 0.86 | 0.86 | -0.07 | 0.00 |
| 74 | D12 | 6602 | SMARCD1 | NM_003076 | 221022 | 228805 | 0.89 | 0.88 | 0.41 | 0.01 |
| 74 | E01 | NA | neg | NA | 275252 | 287125 | 1.11 | 1.11 | -6.35 | -7.79 |
| 74 | E02 | NA | neg | NA | 227317 | 236078 | 0.91 | 0.91 | -0.90 | -2.15 |
| 74 | E03 | 6611 | SMS | NM_004595 | 219492 | 211794 | 0.88 | 0.82 | 0.68 | 2.12 |
| 74 | E04 | 56950 | SMYD2 | NM_020197 | 231014 | 226885 | 0.93 | 0.88 | -1.33 | -0.01 |
| 74 | E05 | 64754 | SMYD3 | NM_022743 | 235711 | 246464 | 0.95 | 0.95 | -1.79 | -3.05 |
| 74 | E06 | 10322 | SMYD5 | XM_039548 | 237786 | 221510 | 0.96 | 0.86 | -1.72 | -0.20 |
| 74 | E07 | 6617 | SNAPC1 | NM_003082 | 202807 | 210645 | 0.82 | 0.81 | 1.98 | -0.06 |
| 74 | E08 | 6622 | SNCA | NM_000345 | 223758 | 193265 | 0.90 | 0.75 | -0.59 | 2.35 |
| 74 | E09 | 79753 | SNIP1 | NM_024700 | 202233 | 210877 | 0.81 | 0.82 | 1.23 | 0.01 |
| 74 | E10 | 8303 | SNN | NM_003498 | 170049 | 151798 | 0.68 | 0.59 | 4.20 | 5.54 |
| 74 | E11 | 8926 | SNURF | NM_005678 | 204632 | 202761 | 0.82 | 0.78 | 0.60 | 1.23 |
| 74 | E12 | 27131 | SNX5 | NM_014426 | 238860 | 234029 | 0.96 | 0.90 | -2.12 | -1.44 |
| 74 | F01 | NA | NA | NA | 142081 | 166801 | 0.57 | 0.64 | 8.32 | 5.93 |
| 74 | F02 | NA | pos | NA | 82573 | 126094 | 0.33 | 0.49 | 15.09 | 10.43 |
| 74 | F03 | 6646 | SOAT1 | NM_003101 | 222940 | 238943 | 0.90 | 0.92 | -0.19 | -0.47 |
| 74 | F04 | 9021 | SOCS3 | NM_003955 | 230722 | 228456 | 0.93 | 0.88 | -1.79 | 0.23 |
| 74 | F05 | 122809 | SOCS4 | NM_080867 | 199103 | 227990 | 0.80 | 0.88 | 1.89 | -0.59 |
| 74 | F06 | 6647 | SOD1 | NM_000454 | 218967 | 222907 | 0.88 | 0.86 | -0.06 | 0.06 |
| 74 | F07 | 6652 | SORD | NM_003104 | 215787 | 215212 | 0.87 | 0.83 | 0.02 | -0.15 |
| 74 | F08 | 50964 | SOST | NM_025237 | 211028 | 218122 | 0.85 | 0.84 | 0.37 | 0.01 |
| 74 | F09 | 6658 | SOX3 | NM_005634 | 198185 | 212003 | 0.80 | 0.82 | 1.21 | 0.30 |
| 74 | F10 | 6668 | SP2 | NM_003110 | 201489 | 205803 | 0.81 | 0.80 | 0.14 | -0.02 |
| 74 | F11 | 6674 | SPAG1 | NM_003114 | 205981 | 216275 | 0.83 | 0.84 | -0.04 | 0.15 |
| 74 | F12 | 9576 | SPAG6 | NM_012443 | 227428 | 224769 | 0.92 | 0.87 | -1.31 | -0.01 |
| 74 | G01 | NA | neg | NA | 250926 | 279734 | 1.01 | 1.08 | -3.34 | -6.64 |
| 74 | G02 | NA | neg | NA | 228809 | 237604 | 0.92 | 0.92 | -0.83 | -1.98 |
| 74 | G03 | 9043 | SPAG9 | NM_003971 | 233579 | 233432 | 0.94 | 0.90 | -0.68 | 0.06 |
| 74 | G04 | 23111 | SPG20 | NM_015087 | 213765 | 229739 | 0.86 | 0.89 | 0.87 | 0.01 |
| 74 | G05 | 10638 | SPHAR | NM_006542 | 227617 | 223507 | 0.92 | 0.86 | -0.63 | -0.18 |
| 74 | G06 | 121599 | SPIC | NM_152323 | 223304 | 232518 | 0.90 | 0.90 | 0.17 | -1.08 |
| 74 | G07 | 10927 | SPIN | NM_006717 | 221890 | 208194 | 0.89 | 0.80 | 0.05 | 0.55 |
| 74 | G08 | 10252 | SPRY1 | NM_005841 | 226815 | 217629 | 0.91 | 0.84 | -0.70 | -0.01 |
| 74 | G09 | 10253 | SPRY2 | NM_005842 | 215640 | 222789 | 0.87 | 0.86 | -0.06 | -0.97 |
| 74 | G10 | 10251 | SPRY3 | NM_005840 | 213642 | 207508 | 0.86 | 0.80 | -0.52 | -0.28 |
| 74 | G11 | 6708 | SPTA1 | NM_003126 | 203922 | 207179 | 0.82 | 0.80 | 0.92 | 1.08 |
| 74 | G12 | 6721 | SREBF2 | NM_004599 | 221539 | 214848 | 0.89 | 0.83 | 0.09 | 1.01 |
| 74 | H01 | NA | NA | NA | 165325 | 154559 | 0.67 | 0.60 | 7.54 | 8.46 |
| 74 | H02 | NA | pos | NA | 79377 | 106855 | 0.32 | 0.41 | 17.31 | 13.74 |
| 74 | H03 | 6731 | SRP72 | NM_006947 | 233423 | 249715 | 0.94 | 0.97 | 0.48 | -0.48 |
| 74 | H04 | 23524 | SRRM2 | NM_016333 | 249941 | 230407 | 1.01 | 0.89 | -2.11 | 1.19 |
| 74 | H05 | 6760 | SS18 | NM_005637 | 233464 | 232409 | 0.94 | 0.90 | -0.16 | 0.10 |
| 74 | H06 | 8082 | SSPN | NM_005086 | 238133 | 234637 | 0.96 | 0.91 | -0.38 | -0.06 |
| 74 | H07 | 10534 | SSSCA1 | NM_006396 | 232529 | 243079 | 0.94 | 0.94 | -0.02 | -2.05 |
| 74 | H08 | 54879 | ST7L | NM_017744 | 227616 | 234038 | 0.92 | 0.90 | 0.35 | -0.57 |
| 74 | H09 | 6769 | STAC | NM_003149 | 225012 | 225395 | 0.91 | 0.87 | 0.01 | -0.01 |
| 74 | H10 | 10254 | STAM2 | NM_005843 | 222420 | 213535 | 0.89 | 0.83 | -0.38 | 0.31 |
| 74 | H11 | 137735 | STARS | NM_139166 | 216734 | 228302 | 0.87 | 0.88 | 0.60 | 0.00 |
| 74 | H12 | 6774 | STAT3 | NM_003150 | 222865 | 220360 | 0.90 | 0.85 | 1.07 | 1.66 |
| 75 | A01 | NA | pos | NA | 53800 | 67412 | 0.20 | 0.25 | 19.23 | 15.62 |
| 75 | A02 | NA | NA | NA | 184929 | 191792 | 0.68 | 0.71 | 6.31 | 4.80 |
| 75 | A03 | 55250 | STATIP1 | NM_018255 | 264176 | 272348 | 0.98 | 1.01 | -0.22 | -2.09 |
| 75 | A04 | 10963 | STIP1 | NM_006819 | 263856 | 267495 | 0.98 | 0.99 | -0.84 | -1.17 |
| 75 | A05 | 114790 | STK11IP | NM_052902 | 261129 | 248625 | 0.97 | 0.92 | -0.03 | 0.28 |
| 75 | A06 | 11075 | STMN2 | NM_007029 | 245616 | 238619 | 0.91 | 0.88 | 1.47 | 0.33 |
| 75 | A07 | 50861 | STMN3 | NM_015894 | 260485 | 255255 | 0.96 | 0.95 | -1.24 | -1.27 |
| 75 | A08 | 2040 | STOM | NM_004099 | 230065 | 241495 | 0.85 | 0.89 | 1.12 | 0.07 |
| 75 | A09 | 161497 | STRC | NM_153700 | 245406 | 216880 | 0.91 | 0.80 | 0.00 | 2.80 |
| 75 | A10 | 27284 | SULT1B1 | NM_014465 | 242204 | 242573 | 0.90 | 0.90 | 0.00 | -0.27 |
| 75 | A11 | 6822 | SULT2A1 | NM_003167 | 231407 | 246745 | 0.86 | 0.91 | 1.26 | -0.09 |
| 75 | A12 | 6829 | SUPT5H | NM_003169 | 236420 | 231845 | 0.88 | 0.86 | 1.33 | 2.12 |
| 75 | B01 | NA | neg | NA | 270034 | 273167 | 1.00 | 1.01 | -5.40 | -4.58 |
| 75 | B02 | NA | neg | NA | 238980 | 227023 | 0.88 | 0.84 | -2.34 | -0.57 |
| 75 | B03 | 6867 | TACC1 | NM_006283 | 214450 | 207172 | 0.79 | 0.77 | 1.36 | 1.27 |
| 75 | B04 | 10579 | TACC2 | NM_006997 | 215740 | 201992 | 0.80 | 0.75 | 0.59 | 2.22 |

| | | | | | | | | | | |
|---|---|---|---|---|---|---|---|---|---|---|
| 75 | B05 | 10460 | TACC3 | NM_006342 | 209111 | 199400 | 0.77 | 0.74 | 1.78 | 2.25 |
| 75 | B06 | 4072 | TACSTD1 | NM_002354 | 229379 | 229378 | 0.85 | 0.85 | -0.25 | -1.17 |
| 75 | B07 | 6884 | TAF13 | NM_005645 | 211753 | 232620 | 0.78 | 0.86 | 0.25 | -1.61 |
| 75 | B08 | 8148 | TAF15 | NM_003487 | 242838 | 235780 | 0.90 | 0.87 | -3.45 | -1.74 |
| 75 | B09 | 6873 | TAF2 | NM_003184 | 218440 | 222803 | 0.81 | 0.82 | -0.66 | -0.03 |
| 75 | B10 | 6874 | TAF4 | NM_003185 | 231527 | 230928 | 0.86 | 0.85 | -2.27 | -1.57 |
| 75 | B11 | 6876 | TAGLN | NM_003186 | 227510 | 214260 | 0.84 | 0.79 | -1.68 | 0.43 |
| 75 | B12 | 8407 | TAGLN2 | NM_003564 | 205792 | 229310 | 0.76 | 0.85 | 1.03 | 0.03 |
| 75 | C01 | NA | pos | NA | 70724 | 69585 | 0.26 | 0.26 | 14.34 | 13.74 |
| 75 | C02 | NA | NA | NA | 188964 | 189003 | 0.70 | 0.70 | 2.68 | 3.36 |
| 75 | C03 | 6767 | ST13 | NM_003932 | 239709 | 203423 | 0.89 | 0.75 | -1.04 | 2.22 |
| 75 | C04 | 6607 | SMN2 | NM_017411 | 205468 | 242838 | 0.76 | 0.90 | 1.69 | -0.70 |
| 75 | C05 | 5763 | PTMS | NM_002824 | 203304 | 228096 | 0.75 | 0.84 | 2.44 | 0.38 |
| 75 | C06 | 5764 | PTN | NM_002825 | 201771 | 216958 | 0.75 | 0.80 | 2.56 | 0.53 |
| 75 | C07 | 140885 | PTPNS1 | NM_080792 | 228577 | 221042 | 0.85 | 0.82 | -1.32 | 0.02 |
| 75 | C08 | 128646 | PTPNS1L2 | NM_178460 | 209223 | 245295 | 0.77 | 0.91 | -0.05 | -1.94 |
| 75 | C09 | 5790 | PTPRCAP | NM_005608 | 212667 | 229899 | 0.79 | 0.85 | 0.00 | -0.02 |
| 75 | C10 | 5805 | PTS | NM_000317 | NA | 215805 | NA | 0.80 | NA | 0.37 |
| 75 | C11 | 754 | PTTG1IP | NM_004339 | 211385 | 297089 | 0.78 | 1.10 | 0.00 | -6.14 |
| 75 | C12 | 10744 | PTTG2 | NM_006607 | 236255 | 247583 | 0.87 | 0.92 | -1.88 | -0.93 |
| 75 | D01 | NA | neg | NA | 265234 | 293294 | 0.98 | 1.09 | -3.96 | -5.85 |
| 75 | D02 | NA | neg | NA | 275844 | 239752 | 1.02 | 0.89 | -5.01 | -1.19 |
| 75 | D03 | 26255 | PTTG3 | NM_021000 | 238105 | 224704 | 0.88 | 0.83 | 0.00 | 0.22 |
| 75 | D04 | 25945 | PVRL3 | NM_015480 | 230960 | 233247 | 0.86 | 0.86 | 0.05 | -0.02 |
| 75 | D05 | 5820 | PVT1 | XM_372058 | 247888 | 229817 | 0.92 | 0.85 | -1.08 | 0.08 |
| 75 | D06 | 5824 | PEX19 | NM_002857 | 235776 | 199553 | 0.87 | 0.74 | 0.09 | 1.90 |
| 75 | D07 | 5827 | PXMP2 | NM_018663 | 214349 | 224377 | 0.79 | 0.83 | 0.96 | -0.41 |
| 75 | D08 | 5836 | PYGL | NM_002863 | 232995 | 217549 | 0.86 | 0.81 | -1.52 | 0.33 |
| 75 | D09 | 5837 | PYGM | NM_005609 | 236926 | 230512 | 0.88 | 0.85 | -1.52 | -0.22 |
| 75 | D10 | 5768 | QSCN6 | NM_002826 | 218296 | 218230 | 0.81 | 0.81 | 0.00 | 0.02 |
| 75 | D11 | 10890 | RAB10 | NM_016131 | 231740 | 233495 | 0.86 | 0.86 | -1.13 | -0.76 |
| 75 | D12 | 5862 | RAB2 | NM_002865 | 223420 | 249850 | 0.83 | 0.93 | 0.26 | -1.28 |
| 75 | E01 | NA | neg | NA | 271305 | 287546 | 1.00 | 1.06 | -5.37 | -4.93 |
| 75 | E02 | NA | neg | NA | 234680 | 356994 | 0.87 | 1.32 | -1.76 | -10.97 |
| 75 | E03 | 57403 | RAB22A | NM_020673 | 225677 | 232377 | 0.84 | 0.86 | 0.41 | -0.03 |
| 75 | E04 | 11031 | RAB31 | NM_006868 | 236872 | 225092 | 0.88 | 0.83 | -1.34 | 1.10 |
| 75 | E05 | 11021 | RAB35 | NM_006861 | 230615 | 236499 | 0.85 | 0.88 | -0.18 | -0.08 |
| 75 | E06 | 9545 | RAB3D | NM_004283 | 226606 | 219329 | 0.84 | 0.81 | 0.18 | 0.59 |
| 75 | E07 | 5866 | RAB3IL1 | NM_013401 | 228228 | 210562 | 0.85 | 0.78 | -1.22 | 1.20 |
| 75 | E08 | 5867 | RAB4A | NM_004578 | 201649 | 239572 | 0.75 | 0.89 | 0.76 | -1.17 |
| 75 | E09 | 5869 | RAB5B | NM_002868 | 204405 | 232528 | 0.76 | 0.86 | 0.88 | 0.02 |
| 75 | E10 | 9366 | RAB9P1 | NR_000039 | 223619 | 223331 | 0.83 | 0.83 | -1.33 | -0.02 |
| 75 | E11 | 10244 | RAB9P40 | NM_005833 | 208567 | 202189 | 0.77 | 0.75 | 0.35 | 2.37 |
| 75 | E12 | 10567 | RABAC1 | NM_006423 | 233763 | 243001 | 0.87 | 0.90 | -1.57 | -0.27 |
| 75 | F01 | NA | NA | NA | 182867 | 164118 | 0.68 | 0.61 | 3.92 | 5.67 |
| 75 | F02 | NA | pos | NA | 101574 | 109966 | 0.38 | 0.41 | 11.93 | 10.38 |
| 75 | F03 | 11020 | RABL4 | NM_006860 | 235146 | 236758 | 0.87 | 0.88 | 0.05 | -0.54 |
| 75 | F04 | 5881 | RAC3 | NM_005052 | 229641 | 211547 | 0.85 | 0.78 | -0.05 | 2.16 |
| 75 | F05 | 5893 | RAD52 | NM_002879 | 228970 | 247600 | 0.85 | 0.92 | 0.55 | -1.17 |
| 75 | F06 | 8438 | RAD54L | NM_003579 | 235155 | 228510 | 0.87 | 0.85 | -0.09 | -0.33 |
| 75 | F07 | 5883 | RAD9A | NM_004584 | 223608 | 208347 | 0.83 | 0.77 | -0.19 | 1.27 |
| 75 | F08 | 10742 | RAI2 | NM_021785 | 200666 | 224346 | 0.74 | 0.83 | 1.43 | 0.02 |
| 75 | F09 | 10928 | RALBP1 | NM_006788 | 210105 | 220135 | 0.78 | 0.81 | 0.89 | 0.97 |
| 75 | F10 | 9649 | RALGPS1A | NM_014636 | 221062 | 225484 | 0.82 | 0.83 | -0.50 | -0.33 |
| 75 | F11 | 51514 | RAMP | NM_016448 | 208566 | 204672 | 0.77 | 0.76 | 0.92 | 2.03 |
| 75 | F12 | 5902 | RANBP1 | NM_002882 | 226223 | 238835 | 0.84 | 0.88 | -0.26 | -0.03 |
| 75 | G01 | NA | neg | NA | 279006 | 267044 | 1.03 | 0.99 | -5.27 | -3.61 |
| 75 | G02 | NA | neg | NA | NA | 258632 | NA | 0.96 | NA | -2.88 |
| 75 | G03 | 26953 | RANBP6 | NM_012416 | 238476 | 226371 | 0.88 | 0.84 | 0.00 | 0.03 |
| 75 | G04 | 10048 | RANBP9 | NM_005493 | 228385 | 237973 | 0.85 | 0.88 | 0.35 | -0.47 |
| 75 | G05 | 5908 | RAP1B | NM_015646 | 242376 | 236294 | 0.90 | 0.87 | -0.49 | -0.52 |
| 75 | G06 | 5912 | RAP2B | NM_002886 | 240253 | 234653 | 0.89 | 0.87 | -0.31 | -1.20 |
| 75 | G07 | 65059 | RAPH1 | NM_025252 | 222646 | 219346 | 0.82 | 0.81 | 0.19 | -0.02 |
| 75 | G08 | 57521 | Raptor | NM_020761 | 225210 | 211855 | 0.83 | 0.78 | -0.70 | 0.78 |
| 75 | G09 | 5918 | RARRES1 | NM_002888 | 217674 | 224289 | 0.81 | 0.83 | 0.43 | 0.28 |
| 75 | G10 | 5919 | RARRES2 | NM_002889 | 216383 | 217721 | 0.80 | 0.81 | 0.24 | 0.02 |
| 75 | G11 | 5920 | RARRES3 | NM_004585 | 220808 | 234978 | 0.82 | 0.87 | 0.00 | -0.93 |
| 75 | G12 | 10156 | RASA4 | NM_006989 | 238533 | 225236 | 0.88 | 0.83 | -1.19 | 0.82 |
| 75 | H01 | NA | NA | NA | NA | 177518 | NA | 0.66 | NA | 5.42 |
| 75 | H02 | NA | pos | NA | 74227 | 86013 | 0.27 | 0.32 | 16.64 | 13.37 |
| 75 | H03 | 9770 | RASSF2 | NM_014737 | 263942 | 252545 | 0.98 | 0.93 | -0.78 | -1.00 |
| 75 | H04 | 30062 | RAX | NM_013435 | 254106 | 246683 | 0.94 | 0.91 | -0.46 | 0.02 |
| 75 | H05 | 9821 | RB1CC1 | NM_014781 | 254666 | 249025 | 0.94 | 0.92 | 0.03 | -0.38 |
| 75 | H06 | 5928 | RBBP4 | NM_005610 | 256997 | 249931 | 0.95 | 0.93 | -0.23 | -1.28 |
| 75 | H07 | 5929 | RBBP5 | NM_005057 | 232378 | 233230 | 0.86 | 0.86 | 0.96 | 0.02 |
| 75 | H08 | 5931 | RBBP7 | NM_002893 | 235095 | 235417 | 0.87 | 0.87 | 0.05 | -0.02 |
| 75 | H09 | 5932 | RBBP8 | NM_002894 | 242095 | 247565 | 0.90 | 0.92 | -0.25 | -0.50 |
| 75 | H10 | 10741 | RBBP9 | NM_006606 | 213120 | 221472 | 0.79 | 0.82 | 2.29 | 0.94 |
| 75 | H11 | 5933 | RBL1 | NM_002895 | 238582 | 237567 | 0.88 | 0.88 | -0.03 | 0.09 |
| 75 | H12 | 27303 | RBMS3 | NM_014483 | 240846 | 236109 | 0.89 | 0.87 | 0.31 | 1.12 |
| 76 | A01 | NA | pos | NA | 53860 | 65645 | 0.22 | 0.25 | 21.83 | 31.82 |
| 76 | A02 | NA | NA | NA | 193979 | 167261 | 0.78 | 0.64 | 5.94 | 13.52 |
| 76 | A03 | 9948 | WDR1 | NM_005112 | 277915 | 259170 | 1.11 | 0.99 | -3.05 | -2.57 |
| 76 | A04 | 55764 | WDR10 | NM_018262 | 262935 | 247516 | 1.05 | 0.95 | -0.05 | 0.12 |
| 76 | A05 | 55717 | WDR11 | NM_018117 | 245874 | 247529 | 0.99 | 0.95 | 0.06 | -0.18 |
| 76 | A06 | 55759 | WDR12 | NM_018256 | 249046 | 225101 | 1.00 | 0.86 | -0.31 | 3.28 |
| 76 | A07 | 64743 | WDR13 | NM_017883 | 264004 | 261467 | 1.06 | 1.00 | -1.79 | -4.76 |
| 76 | A08 | 10885 | WDR3 | NM_006784 | 226141 | 229535 | 0.91 | 0.88 | 1.85 | 2.14 |

| | | | | | | | | | | | |
|---|---|---|---|---|---|---|---|---|---|---|---|
| 76 | A09 | 10785 | WDR4 | NM_018669 | 242255 | 227476 | 0.97 | 0.87 | -0.64 | 1.40 |
| 76 | A10 | 11091 | WDR5 | NM_017588 | 234211 | 224811 | 0.94 | 0.86 | 0.39 | 0.12 |
| 76 | A11 | 11180 | WDR6 | NM_018031 | 228678 | 230370 | 0.92 | 0.88 | 0.36 | -0.82 |
| 76 | A12 | 23335 | WDR7 | NM_015285 | 230917 | 251584 | 0.93 | 0.96 | 2.34 | -0.72 |
| 76 | B01 | NA | neg | NA | 253479 | 262693 | 1.02 | 1.01 | -4.13 | -8.93 |
| 76 | B02 | NA | neg | NA | 224981 | 222994 | 0.90 | 0.85 | -0.90 | -1.78 |
| 76 | B03 | 49856 | WDR8 | NM_017818 | 208549 | 200044 | 0.84 | 0.77 | 1.50 | 2.81 |
| 76 | B04 | 7466 | WFS1 | NM_006005 | 232932 | 227325 | 0.93 | 0.87 | 0.03 | -1.51 |
| 76 | B05 | 7469 | WHSC2 | NM_005663 | 214388 | 207922 | 0.86 | 0.80 | 0.31 | 1.68 |
| 76 | B06 | 7474 | WNT5A | NM_003392 | 213421 | 214471 | 0.86 | 0.82 | 0.41 | -0.07 |
| 76 | B07 | 9589 | WTAP | NM_004906 | 220428 | 226285 | 0.88 | 0.87 | -0.17 | -3.69 |
| 76 | B08 | 7494 | XBP1 | NM_005080 | 237620 | 212357 | 0.95 | 0.81 | -2.77 | -0.04 |
| 76 | B09 | 7508 | XPC | NM_004628 | 217091 | 204848 | 0.87 | 0.79 | -1.11 | 0.21 |
| 76 | B10 | 7514 | XPO1 | NM_003400 | 192092 | 193347 | 0.77 | 0.74 | 1.85 | 0.52 |
| 76 | B11 | 11260 | XPOT | NM_007235 | 221265 | 216897 | 0.89 | 0.83 | -2.12 | -3.66 |
| 76 | B12 | 7516 | XRCC2 | NM_005431 | 222409 | 217881 | 0.89 | 0.83 | -0.02 | 0.08 |
| 76 | C01 | NA | pos | NA | 64030 | 59144 | 0.26 | 0.23 | 17.04 | 30.72 |
| 76 | C02 | NA | NA | NA | 209317 | 163939 | 0.84 | 0.63 | 0.57 | 11.84 |
| 76 | C03 | 7518 | XRCC4 | NM_003401 | 212778 | 213041 | 0.85 | 0.82 | 0.71 | 3.46 |
| 76 | C04 | 7520 | XRCC5 | NM_021141 | 201351 | 238252 | 0.81 | 0.91 | 3.30 | -0.48 |
| 76 | C05 | NA | NA | NA | 214360 | 217336 | 0.86 | 0.83 | 0.01 | 2.98 |
| 76 | C06 | NA | NA | NA | 213426 | 225742 | 0.86 | 0.87 | 0.10 | 0.88 |
| 76 | C07 | NA | NA | NA | 214719 | 222126 | 0.86 | 0.85 | 0.17 | 0.05 |
| 76 | C08 | NA | NA | NA | 210497 | 229096 | 0.84 | 0.88 | -0.01 | -0.06 |
| 76 | C09 | NA | NA | NA | 232517 | 226938 | 0.93 | 0.87 | -3.16 | -0.78 |
| 76 | C10 | NA | NA | NA | 225213 | 228027 | 0.90 | 0.87 | -2.22 | -2.74 |
| 76 | C11 | NA | NA | NA | 235545 | 212443 | 0.94 | 0.81 | -4.04 | 0.13 |
| 76 | C12 | NA | NA | NA | 244457 | 249764 | 0.98 | 0.96 | -2.82 | -2.67 |
| 76 | D01 | NA | neg | NA | 286087 | 285641 | 1.15 | 1.09 | -6.23 | -10.47 |
| 76 | D02 | NA | neg | NA | 244750 | 235697 | 0.98 | 0.90 | -1.54 | -1.47 |
| 76 | D03 | NA | NA | NA | 247982 | 229243 | 0.99 | 0.88 | -1.37 | 0.15 |
| 76 | D04 | NA | NA | NA | 221547 | 237486 | 0.89 | 0.91 | 2.92 | -0.74 |
| 76 | D05 | NA | NA | NA | 242306 | 238292 | 0.97 | 0.91 | -1.25 | -1.19 |
| 76 | D06 | NA | NA | NA | 220915 | 224118 | 0.89 | 0.86 | 1.16 | 0.79 |
| 76 | D07 | NA | NA | NA | 211303 | 230390 | 0.85 | 0.88 | 2.46 | -1.83 |
| 76 | D08 | NA | NA | NA | 227173 | 232657 | 0.91 | 0.89 | 0.01 | -1.09 |
| 76 | D09 | NA | NA | NA | 206519 | 214842 | 0.83 | 0.82 | 1.69 | 1.01 |
| 76 | D10 | NA | NA | NA | 222597 | 209170 | 0.89 | 0.80 | -0.01 | 0.26 |
| 76 | D11 | NA | NA | NA | 230252 | 210013 | 0.92 | 0.80 | -1.54 | 0.17 |
| 76 | D12 | NA | NA | NA | 241198 | 233184 | 0.97 | 0.89 | -0.55 | -0.08 |
| 76 | E01 | NA | neg | NA | 289170 | 281377 | 1.16 | 1.08 | -6.04 | -9.59 |
| 76 | E02 | NA | neg | NA | 245674 | 231731 | 0.98 | 0.89 | -1.11 | -0.65 |
| 76 | E03 | NA | NA | NA | 235303 | 225846 | 0.94 | 0.87 | 0.60 | 0.87 |
| 76 | E04 | NA | NA | NA | 256384 | 234558 | 1.03 | 0.90 | -0.50 | -0.10 |
| 76 | E05 | NA | NA | NA | 236001 | 237282 | 0.95 | 0.91 | -0.01 | -0.90 |
| 76 | E06 | NA | NA | NA | 252629 | 228671 | 1.01 | 0.88 | -1.91 | 0.07 |
| 76 | E07 | NA | NA | NA | 191772 | 207615 | 0.77 | 0.80 | 5.21 | 2.38 |
| 76 | E08 | NA | NA | NA | 242456 | 224305 | 0.97 | 0.86 | -1.19 | 0.52 |
| 76 | E09 | NA | NA | NA | 197765 | 224829 | 0.79 | 0.86 | 3.22 | -0.69 |
| 76 | E10 | NA | NA | NA | 227099 | 211878 | 0.91 | 0.81 | 0.01 | -0.12 |
| 76 | E11 | NA | NA | NA | 222112 | 210585 | 0.89 | 0.81 | -0.08 | 0.18 |
| 76 | E12 | NA | NA | NA | 240928 | 234133 | 0.97 | 0.90 | 0.02 | -0.14 |
| 76 | F01 | NA | NA | NA | 192487 | 160906 | 0.77 | 0.62 | 3.78 | 11.44 |
| 76 | F02 | NA | pos | NA | 93148 | 70461 | 0.37 | 0.27 | 15.04 | 27.73 |
| 76 | F03 | NA | NA | NA | 232185 | 227803 | 0.93 | 0.87 | -0.19 | -0.15 |
| 76 | F04 | NA | NA | NA | 242183 | 221889 | 0.97 | 0.85 | -0.03 | 1.51 |
| 76 | F05 | NA | NA | NA | 225558 | 222917 | 0.90 | 0.85 | 0.04 | 1.02 |
| 76 | F06 | NA | NA | NA | 226335 | 228108 | 0.91 | 0.87 | -0.06 | -0.49 |
| 76 | F07 | NA | NA | NA | 236661 | 216212 | 0.95 | 0.83 | -1.02 | 0.17 |
| 76 | F08 | NA | NA | NA | 215023 | 230371 | 0.86 | 0.88 | 0.78 | -1.24 |
| 76 | F09 | NA | NA | NA | 210440 | 205073 | 0.84 | 0.79 | 0.64 | 2.21 |
| 76 | F10 | NA | NA | NA | 226780 | 213366 | 0.91 | 0.82 | -1.09 | -1.05 |
| 76 | F11 | NA | NA | NA | 204975 | 263173 | 0.82 | 1.01 | 0.72 | -9.96 |
| 76 | F12 | NA | NA | NA | 229016 | 225114 | 0.92 | 0.86 | 0.23 | 0.82 |
| 76 | G01 | NA | neg | NA | 273307 | 259178 | 1.10 | 0.99 | -4.73 | -6.86 |
| 76 | G02 | NA | neg | NA | 231025 | 277949 | 0.93 | 1.07 | 0.06 | -10.24 |
| 76 | G03 | NA | NA | NA | 252945 | 227232 | 1.01 | 0.87 | -1.89 | -0.65 |
| 76 | G04 | NA | NA | NA | 252290 | 226372 | 1.01 | 0.87 | -0.52 | 0.10 |
| 76 | G05 | NA | NA | NA | 252882 | 224246 | 1.01 | 0.86 | -2.41 | 0.18 |
| 76 | G06 | NA | NA | NA | 320028 | 228364 | 1.28 | 0.88 | -10.03 | -1.14 |
| 76 | G07 | NA | NA | NA | 226487 | 213300 | 0.91 | 0.82 | 0.79 | 0.09 |
| 76 | G08 | NA | NA | NA | 219693 | 212590 | 0.88 | 0.81 | 0.90 | 1.36 |
| 76 | G09 | NA | NA | NA | 214144 | 216299 | 0.86 | 0.83 | 0.87 | -0.42 |
| 76 | G10 | NA | NA | NA | 216020 | 213436 | 0.87 | 0.82 | 0.78 | -1.67 |
| 76 | G11 | NA | NA | NA | 216403 | 205230 | 0.87 | 0.79 | 0.08 | -0.13 |
| 76 | G12 | NA | NA | NA | 237368 | 224145 | 0.95 | 0.86 | -0.07 | 0.39 |
| 76 | H01 | NA | NA | NA | 220923 | 162501 | 0.89 | 0.62 | 2.39 | 12.29 |
| 76 | H02 | NA | pos | NA | 106236 | 75796 | 0.43 | 0.29 | 15.39 | 27.90 |
| 76 | H03 | NA | NA | NA | 245007 | 237466 | 0.98 | 0.91 | 0.19 | -0.76 |
| 76 | H04 | NA | NA | NA | 243443 | 235846 | 0.98 | 0.90 | 1.66 | 0.13 |
| 76 | H05 | NA | NA | NA | 266156 | 247477 | 1.07 | 0.95 | -2.73 | -2.27 |
| 76 | H06 | NA | NA | NA | 241361 | 324836 | 0.97 | 1.24 | 0.06 | -16.78 |
| 76 | H07 | NA | NA | NA | 248556 | 223714 | 1.00 | 0.86 | -0.54 | -0.05 |
| 76 | H08 | NA | NA | NA | 241649 | 229564 | 0.97 | 0.88 | -0.41 | 0.04 |
| 76 | H09 | NA | NA | NA | 239780 | 224770 | 0.96 | 0.86 | -0.85 | -0.21 |
| 76 | H10 | NA | NA | NA | 233833 | 209662 | 0.94 | 0.80 | -0.06 | 0.75 |
| 76 | H11 | NA | NA | NA | 221412 | 211481 | 0.89 | 0.81 | 0.69 | 0.48 |
| 76 | H12 | NA | NA | NA | 236050 | 227820 | 0.95 | 0.87 | 1.26 | 1.46 |
