# Supplementary material for: An RNA Interference Lethality Screen of the Human Druggable Genome to Identify Molecular Vulnerabilities in Epithelial Ovarian Cancer
Source: PLoS One. 2012 Oct 9;7(10):e47086. doi: 10.1371/journal.pone.0047086 (PMC3467214; doi:10.1371/journal.pone.0047086)
Supplement: Table S2 — List of 300 genes targeted by this custom siRNA library purchased from Qiagen. Each well contains a pool of 4 siRNA duplexes targeting the indicated gene. The siRNA pools are arrayed into five 96-well plates. (PDF) [file pone.0047086.s008.pdf]

Supplementary Table S2

List of 300 genes targeted by this custom siRNA library purchased from Qiagen.

Each well contains a pool of 4 siRNA duplexes targeting the indicated gene. The siRNA pools are arrayed into five 96-well plates.

PLATE ID	WELL	LOCUS ID	NCBI GENE SYMBOL	siRNA TARGET SEQUENCE 1	PRODUCT 1 ID	siRNA TARGET SEQUENCE 2	PRODUCT 2 ID	siRNA TARGET SEQUENCE 3	PRODUCT 3 ID	siRNA TARGET SEQUENCE 4	PRODUCT 4 ID
900097-1	B2	3832	*KIF11*	ACGGAGGAGATAGAACGTTTA	SI02653693	GCCGATAAGATAGAAGATCAA	SI02653770	CTCGGGAAGCTGGAAATATAA	SI03019793	AACTGGATCGTAAGAAGGCAG	1024738
900097-1	B3	4660	*PPP1R12B*	CGGGAGGTAGTAATCCTACAA	SI00038472	CAGACACGAAGGTCTACTCAA	SI03063403	CATGTAAGACTTCTACCCTAA	SI03074078	CCCACCTGCTATGGACAAATA	SI03076374
900097-1	B4	3486	*IGFBP3*	CAAGGTTAATGTGGAGCTCAA	SI02780589	CCAAATATAGTAAGATCTATA	SI02623866	GAGAAGATATGTTCTTGTTAA	SI02623873	CACCCATGTTTGTTGAACTTA	SI02623880
900097-1	B5	7027	*TFDP1*	CAGAACCTTAGTCCCGGGAAA	SI00094654	ATTGACTTTATTGTCCCTTAA	SI03052462	CACATTTGAAATCCACGATGA	SI03056858	CAGCAGAATGCAACTTTCTAA	SI03065594
900097-1	B6	128178	*EDARADD*	CCGGATGTTGAAACAACCACA	SI00148841	CCCTAGCACTTTATCCTTTAA	SI00153664	TACCCACAGCATTATATGTAA	SI02647778	TTGCTCAATGATCAGGACTTA	SI03024357
900097-1	B7	5151	*PDE8A*	ACGGAGATAATATACAACAAA	SI00040250	CAGGTCTATCAGATCATCAAA	SI00040257	CCCGGATACATTCCATGACAA	SI00040264	CCGGACCCTAATCAAACGAAT	SI03081757
900097-1	B8	2906	*GRIN2D*	CCGCTACTGGTCGGTCGACAA	SI00016765	CCGGGATTACTCCTTCAATGA	SI00016772	GAGGATTTGGTCCCTCAACTA	SI00016779	CTGCGTGATTATGGTTTCCTT	SI00016786
900097-1	B9	10460	*TACC3*	CAGGAAGTTCTGAGAACCAAA	SI00738423	AAGGAACTTTCCAAAGCTGAA	SI00738430	CTGCAGGTCTTAAACGACAAA	SI00738444	CCCGTTCGGAGGTGACACCAA	SI03186568
900097-1	B10	3849	*KRT2*	CTGGTGGAAGCCGGAGATCAA	SI00464772	AAGCAGCACCATTTCATCAAA	SI00464779	AAGCAGTGTAAGAATGTGCAA	SI00464786	CAGGATCTTGTGGAGGATTAT	SI00464793
900097-1	B11	8891	*EIF2B3*	CCGGAGTGAACTGATTCCATA	SI00377349	AAAGAGGAGGATCTAAAGAAA	SI00377363	CCAGTTGGGAACAAACCTTTA	SI00377370	AGGATTGAAGCCAAAGCTAAA	SI03145975
900097-1	C2	5764	*PTN*	TACCGTAGGCTTTGTATTCAA	SI00043750	CTGGAGCTGAGTGCAAGCAAA	SI00043764	CAGTGAGTCATCCGTCCAGAA	SI03073049	CTGACCAAGCCCAAACCTCAA	SI03093083
900097-1	C3	1621	*DBH*	CACGTACTGGTGCTACATTAA	SI00015617	CCGGGAGTGGGAGATCGTGAA	SI00015624	GCGGGCTAATGAGCCAATAAA	SI00015638	CTCAGGCATCCGCTTGTACTA	SI03088785
900097-1	C4	5901	*RAN*	TAGTGTGAAGTTGATACGCAA	SI00087493	TTGGGAATTCTTGCTGTTAAA	SI00087507	TTGGCTAGGTAGGTCACCTA	SI03025064	AATGTGGTTTGTACATATCAA	SI03037447
900097-1	C5	1938	*EEF2*	CCGCGCCATCATGGACAAGAA	SI00376369	TGCCGTTTCTTTCAATATTTA	SI00376376	CTGGCCGAGGACATCGATAAA	SI00376383	TTGGAATAATAAATTTGATAA	SI00376390
900097-1	C6	2308	*FOXO1*	AACCAAGTAGCCTGTTATCAA	SI02781415	CCCGAGTTTAGTAACAGTGCA	SI02781450	AAGAGCTGCATCCATGGACAA	SI04435144	CTCGAACTAGCTCAAATGCTA	SI00031437
900097-1	C7	5791	*PTPRE*	CCGAGTGATCCTTTCCATGAA	SI02665635	ACCACGGGCAATTAAACTTTA	SI00089068	GACCATCGTCATGTTAACAAA	SI00089075	CAGTGAATTCACAACCCTGAA	SI00089082
900097-1	C8	6235	*RPS29*	GAAGGATATCGGTTTCATTAA	SI00707749	CCGCCAGTGTTTCCGTCAGTA	SI00707756	CTCGCTCTTGTCGTGTCTGTT	SI03204446	TCGTTGCACTGCTGAGAGCAA	SI03235113
900097-1	C9	3543	*IGLL1*	ACCCTCTTTGTCAACCAGAAA	SI00446628	CACAAGGACCTCTGACCCTGA	SI00446635	CCCTAAATAAATACCCTCTTT	SI00446642	ACCCTAAATAAATACCCTCTT	SI00446649
900097-1	C10	6188	*RPS3*	GAGGTTGTGGTGTCTGGGAAA	SI00707791	CGGCAAGATGGCAGTGCAAAT	SI00707798	AAGAGGAAGTTTGTCGCTGAT	SI03128741	CTGGAGTTGAGGTGCGAGTTA	SI03210935
900097-1	C11	1316	*KLF6*	CTGCCTAGAGCTGGAACGTTA	SI00025074	GAGGTGGATCCTGTAGGCTAA	SI00025081	CACGGCCAAGTTTACCTCCGA	SI00025095	CAGGAAGATCTGTGGACCAAA	SI03068968
900097-1	D2	2633	*GBP1*	CAGGAGAAGTTTATTCTTCCA	SI00425495	AAGGCATGTACCATAAGCTAA	SI00425502	AACACTCATACTTGAAATCTT	SI03123176	ATGGGACACTTTAGACCATTA	SI03154340
900097-1	D3	2958	*GTF2A2*	CAGAGAGGTGACAGAACTTAT	SI00431928	CAGAAATACTACTTTGGGAAA	SI00431935	CACCTTTATTATACAGCATGA	SI00431942	TCCAGTAGAAAGAAACTTGTA	SI00431949
900097-1	D4	4437	*MSH3*	TCGAGTCGAAAGGATGGATAA	SI00037828	AGCCCGAGAGCTCAATATTTA	SI00037835	CAGCAAGGAGTTATGGATTAA	SI02628591	CGCCGCTAGAATTACAATACA	SI03084564
900097-1	D5	4256	*MGP*	CCCAGATTGATAAGTAATGAA	SI00645407	CAGCAGAGATGGAGAGCTAAA	SI00645414	ATGTGATTGTTCCATAATAAA	SI00645421	TAGCAGCATTACTGAAATACA	SI00645428
900097-1	D6	9099	*USP2*	CAGATTGTGGTTACTGTTCTA	SI03246698	CAGGAGAATGGCACACTTTCA	SI03247034	CCGAGTGACACTGAGACCTAA	SI00758198	CCGCGCTTTGTTGGCTATAAT	SI00758212
900097-1	D7	86	*ACTL6A*	AAAGCTTTAACTGGCTCTATA	SI02779987	CAACAGTGGAACGGAGGTTTA	SI02779994	TACGAGGTCTTGGCACAATTA	SI00062405	TGGCAGGAGGAAACACACTAA	SI04433940
900097-1	D8	3549	*IHH*	CTGGGTGTATTACGAGTCAAA	SI00446859	CCGCCTGAACTCGCTGGCTAT	SI00446866	CGGCTTTGACTGGGTGTATTA	SI00446873	CCCGGCCATTCTGAGGTATGA	SI04382714
900097-1	D9	1053	*CEBPE*	TAGACCATGGATAAATGGCAA	SI00342909	CTCCGATCTCTTTGCCGTGAA	SI00342916	AGGACCCAGCATAATGATTAT	SI00342923	CGGGACCTACTACGAGTGTGA	SI00342930
900097-1	D10	1557	*CYP2C19*	CAGAAACGTTTCGATTATAAA	SI00015162	AACCCATAACAAATTACTTAA	SI00015176	AAGGATGTCAGCAAATCCTTA	SI00015183	TCCCACTATCATTGATTATTT	SI03114076
900097-1	D11	53616	*ADAM22*	CCCAGTCATTAGCCCATAATA	SI00060823	TACGTGAGTTTGATGAAATACA	SI00060837	TTAGACCTCGGTCTAATTCAA	SI03021298	CTCGATGTCGTGCTAAATCAT	SI03091228
900097-1	E2	11217	*AKAP2*	AAGAACAATACTGCATTAGAA	SI00293818	AAGGATGATGACCATGGGATT	SI00293832	CAGGCTTGGAATCAGCTACAA	SI03176096	CTGGTGCAGAATGCCATTCAA	SI03213819
900097-1	E3	10594	*PRPF8*	ACGGGCATGTATCGATACAAA	SI00088543	ATGGCTTGTCATCCTGAATAA	SI00088550	CAACGTCGTCATCAACTATAA	SI00088557	CTCATCGTGGACCACAACATA	SI03089135
900097-1	E4	5618	*PRLR*	CCGGGACTCCTGAGAACAATA	SI00019047	AACCCATAAGTTTAACTGTAT	SI00019061	ACGCTCCTGTATGAAATTCGA	SI00019068	CTCCGCTAAACCCTTGGATTA	SI03090052
900097-1	E5	54820	*NDE1*	ACGCAGCTGCAACAAATTGAA	SI00655851	CGCGCAGACCAAAGCCATTAA	SI00655858	AGCCGAGAATATGAAGCTGAA	SI00655865	AAGACCAATTGCAGAAATACA	SI00655872
900097-1	E6	26271	*FBXO5*	CATGTTCATTCCGGACTTAAA	SI00097839	GAGAATTTCGGTGACAGTCTA	SI00097853	AAGCAAGCGCTTGCATAACAA	SI00097860	GTGGATTTGATTATTGTACGA	SI03106999
900097-1	E7	53353	*LRP1B*	CCCTACTATACTCGTTAGCAA	SI02777782	TAGATGATTCATGTAAGTTAA	SI00122290	AACGTTTATACTGGACAGATA	SI00122297	CACTGGCGGTGTGATAGCCAA	SI04439813
900097-1	E8	1325	*CORT*	TAAGAGCAAATTCGCTCTAAA	SI00025102	CAGCTGGAGATTGGGCTTAAA	SI00025116	AAAGACCTGAAGCTTTAATTA	SI03026394	TCCAGGCGTTAGACATGTATA	SI03113761
900097-1	E9	10732	*TCFL5*	CTCGGCTAGTCTGACTCGAAA	SI00090132	CTCATTCGACATCCATCTGAA	SI00090153	ATCCATGAATGTCCTCTTAGA	SI03047408	TACTGTCACAGTAGACGCTTA	SI03110310
900097-1	E10	6585	*SLIT1*	CCGGATCTGAACCCATGTCAA	SI00726103	CACGGCAGAACTGCGATTGAA	SI00726110	ACGGAGGATAGACCTGAGCAA	SI00726117	CACCGAGCGCCTGGAACTCAA	SI03058258
900097-1	E11	1298	*COL9A2*	CAGGATATTGGAGTTACTCTA	SI00029162	AAGCCCGGGATTGATGGTTTA	SI00029169	AACGGCAAGGATGGAGATCGA	SI03029901	CTGGCGCAGATTAGAGGTCCA	SI03098130
900097-1	F2	4324	*MMP15*	CACCAAATACTGGAAATTCGA	SI03648946	CTGGCTCTTTCGAGAAGCGAA	SI00037688	CCGGAGCGCTGAGCCAGACAA	SI00037695	TACATTTATTTCTGTAAATAA	SI00037702
900097-1	F3	81029	*WNT5B*	CTGGTGGTCATTAGCTTTGAA	SI00763483	CAGGGTCTTGTCCAGAATGTA	SI00763490	CAGGATGTAGGGACTTGGAAA	SI00763497	CACAAAGGTCTATATTATATA	SI00763504
900097-1	F4	917	*CD3G*	CTCGAGGGATGTATCAGTGTA	SI00001358	CTGGCTATCATTCTTCTTCAA	SI00001365	CAGCCCTAAATCTAGACTCAA	SI02622893	ATGATCGGCTTCCTAACTGAA	SI03049592
900097-1	F5	1476	*CSTB*	CAAGAAGTTCCCTGTGTTTAA	SI00355936	TCCAATCTCTCCCTCATGAAA	SI00355950	CAAGCATGATGAGCTGACCTA	SI03157252	CAAGCCCTTGACCTTATCTAA	SI04337375
900097-1	F6	9543	*PUNC*	CACCAAGGAGATCATCGGCTA	SI00696031	CCACGTCAGGCTCAAGAATAA	SI00696038	AGCCTACAGTTTCTACATCAA	SI00696045	TGGGTCCTTGATGATCCGTCA	SI04347483
900097-1	F7	2132	*EXT2*	GAGGTTCTGGCTAACGTTAAA	SI00008827	CCGGTGGTCTGGGAAGCGTA	SI00008834	CCCATTCTATCGAGTCCTCAA	SI00008841	CAGGTCAAACATGGAGAGTCA	SI00008848
900097-1	F8	3720	*JARID2*	TCGGATTTATTTCAAGGCGAA	SI00449624	CCCGTGGTCGATTTATATATA	SI00449631	CAGCAAGTGACTGACCTCAAA	SI00449638	AGGGTGAAGTTTGGAGAACAA	SI03147550
900097-1	F9	10893	*MMP24*	CACCTACAGCATTCACAACTA	SI00091077	CAGGGTCTCTTCGGAGACCCA	SI00091084	AACGATCGAGTGGATGAAGAA	SI00091098	TAGGCGGAGAAACAAGCGCTA	SI03111857
900097-1	F10	23481	*PES1*	CACCTTGAAGCTGGAGGATAA	SI00682339	CTCGACCACATCATCAAGGAA	SI00682346	CCGGCTCACTGTGGAGTTCAT	SI00682360	TAGAGCGTTTAAAGGACAATA	SI03226965
900097-1	F11	4194	*MDM4*	AGCCCTCTCTATGATATGCTA	SI00037142	AAAGAGGTCATGCACTATTTA	SI00037149	GACCTAAAGATGCGTATATAA	SI00037163	GACCACGAGACGGGAACATTA	SI03101476
900097-1	G2	6125	*RPL5*	AAACTTATGAACAGCAACTAA	SI00706615	TTCGTGTGACAAACAGAGATA	SI00706622	ACTGATTATTATGCTCGGAAA	SI03142433	TCAGATATAGATAATAAACTT	SI03229856
900097-1	G3	5714	*PSMD8*	TCCAACTGACATGTTCATTAA	SI00694967	AAGGGCGAGTGGAACCGTAAA	SI00694974	CTGCAGGGTTTCGCCCAATAA	SI00694981	ATGCTACTACTTTGATTACAA	SI00694988
900097-1	G4	6135	*RPL11*	ATGGCGCAGGATCAAGGTGAA	SI00705586	GCGGGAGTATGAGTTAAGAAA	SI00705593	CGGGAACTTCGCATCCGCAAA	SI03196683	CTGGTTCCAGCAGAAGTATGA	SI03214127
900097-1	G5	330	*BIRC3*	AATTGGGAACCGAAGGATAAT	SI00299439	AAGACACTTCAAGATACACAG	SI02654449	TTCAAGATACACAGTTTCTAA	SI02654456	CAAGAACATGATGTTATTAAA	SI02661918
900097-1	G6	7157	*TP53*	AAGGAAATTTGCGTGTGGAGT	SI02655170	CGGCATGAACCGGAGGCCCAT	1024849	TTGGTGAACCTTAGTACCTAA	SI04384079	CAGAGTGCATTGTGAGGGTTA	SI00011655
900097-1	G7	5224	*PGAM2*	CCAGGCGATCATGGAGCTGAA	SI00682983	CTGCTACACGTCAGTGCTGAA	SI00682990	CAAGGCCATCAAGGATGCCAA	SI00682997	TCGATGCAGAGCTGAGTGAAA	SI00683004
900097-1	G8	81930	*KIF18A*	ATCCGTCTACAGTAACCTTAA	SI00140224	CAGGTGGAACTAATCTGGTTA	SI00140238	CAGGAGGACTTGGACTCTACA	SI00140245	CTCGAAGTGTAAATTACCCGA	SI03090941
900097-1	G9	3491	*CYR61*	AGGGCACACCTAGACAAACAA	SI02626421	TCCCTTCTACAGGCTGTTCAA	SI02626428	AACCCTTTACAAGGCCAGAAA	SI03028655	CAAGAACGTCATGATGATCCA	SI03053477
900097-1	G10	1891	*ECH1*	ACCCAGTTTCCCAGTTTATAA	SI00375921	CAGCCGGGTGTTCCCAGACAA	SI00375928	CCGTGACATCATCACTCGATA	SI00375935	CCCGTGATTGCTGCCGTCCAT	SI03186330
900097-1	G11	27175	*TUBG2*	AAGCTTGGTTGTCAGTATAAA	SI00116067	CCGGGAGATCATCACCCTGCA	SI00116074	TTCAGCTCTAATAAAGTAATA	SI00116081	CAGGACGAGATGAGCGACGTA	SI00116088
900097-2	B2	55558	*PLXNA3*	CTGCCTATTTATTGAATCGAA	SI02778055	CGCGGGAGACTCAGAGTTCTA	SI00118510	ATGCCTCTTGTCGGAAGCATA	SI02642661	ACGAGCTGTATTTCTATGTCA	SI02642668
900097-2	B3	6217	*RPS16*	CTGGTGGCCTATTACCAGAAA	SI00707329	ATGATTGAGCCGCGCACGCTA	SI03151960	CCCGCGCTCGCTACCAGAAAT	SI03184993	ACGCGGCAATGGTCTCATCAA	SI04367167
900097-2	B4	9782	*MATR3*	ACCAGCGTTAGTATTTGGCAA	SI03137659	ATGGTTGACCATTGTTTGAAA	SI03155131	CAAGAGCTTGGACGTGTGATA	SI03156937	CACGCCTTGCTAGTTTAATGA	SI03163867
900097-2	B5	5973	*RENBP*	CTCCGTCATTGCATTCGGAAA	SI00701183	CACGTGATTGACAAGTTCCTA	SI00701197	GCGGGTGTATGATGACCTCAA	SI00701204	ATGGATGTATTGTCGCCTGTA	SI03153801
900097-2	B6	5199	*CFP*	GAGCCTATCAACCCAGATAAA	SI00682787	CCGACGGAACATGAAGTCCAT	SI00682794	ACGAACCTAAGGAGACACGAA	SI00682801	AAGGATCATGGTCAGAGTGGA	SI00682808
900097-2	B7	8467	*SMARCA5*	CTCAATTGTTATGTCATTTAA	SI00726775	AACCAGTAGTTCTTTAATTTA	SI00726782	CTGGATTCAATAGTCATTCAA	SI00726789	CAGAGGAGATGTAATACCTTA	SI00726796
900097-2	B8	102	*ADAM10*	ATGGTAAGAAGAAGTCCTTAA	SI00022029	ATGGTGTTGCTGAGAGTGTTA	SI00022043	TTGGTGGGCAGTATTACTTAT	SI03025582	GAGAGATATATTAAAGACCGA	SI03102680
900097-2	B9	3375	*IAPP*	ATGCAGGGTATTGCGAAACAA	SI00009107	TTAGAGGACAATGTAACTCTA	SI00009121	TTGGTGCCATTCTCTCATCTA	SI03025547	CAACGTGGGATCCAATACATA	SI03053218
900097-2	B10	578	*BAK1*	AAGCGAAGTCTTTGCCTTCTC	SI00299376	CAGGCTGCAGAGGCACCTCAA	SI02654512	CTCAAGAGTACAGAAGCTTTA	SI02654519	CTGGACTTAAGCGAAGTCTTT	SI02654526
900097-2	B11	1005	*CDH7*	CTCGAAGGTCTTATCAATATA	SI00341852	ACGGATAGAAGCTGCAAATAA	SI00341866	CACCAAGAGACTGGATCGTGA	SI03160444	CTCCTTATCGTCACTATGAGA	SI03203732
900097-2	C2	3550	*IK*	AATGACATTGTCATTAGCAAG	SI00300531	AACCCGTAACAAGAAGCTTAA	SI00084336	AACGGGAGCGTGATCGGGAAA	SI03030013	CAGCGAGTATATGAACAACAA	SI00084350
900097-2	C3	3817	*KLK2*	CTGAGGTTCCCTAGAGTTCAA	SI00078743	CAGCATATAATTCTCCCTGCA	SI00078750	TACTTGAATAATTGACCTGAA	SI00078757	GACGAGTATGGAGTACTTGAA	SI00078764
900097-2	C4	2539	*G6PD*	CTGCGTTATCCTCACCTTCAA	SI00008855	ATCGGGTGACCTGGCCAAGAA	SI00008862	CCCTATATTTATGGCAGCCGA	SI03079251	CCGGGACGTGATGCAGAACCA	SI03082450
900097-2	C5	6168	*RPL37A*	ATGGGTTAATTTATGTAACAA	SI00706489	AAAGTCGGGATCGTCGGTAAA	SI03122413	CATGGCCAAACGTACCAAGAA	SI04277721	AGGAGTTGAAAGACCAGTAGA	SI04309788
900097-2	C6	10077	*TSPAN32*	TCAAATATACATCACATCAAA	SI00683739	CAGCAATGGTGCATTGAGCAA	SI00683746	CACCATGGTGACTCTTACCTA	SI00683753	CAGCTTGGACCGCAAGGGCAA	SI00683760
900097-2	C7	11093	*ADAMTS13*	TGGCGAGAGCTTCATCATGAA	SI00292047	CCAAGAGTAAACACTGCCTAA	SI00292054	TGGGTGGAGCCAGACCATCAA	SI00292061	CACCAGGAGGCGGCAGTGCAA	SI00292068
900097-2	C8	10174	*SORBS3*	ACCACTAGCCTGGCTCAAATA	SI03137463	ACCGGGCGATGTACCAGTACA	SI03139402	CGGAACGTTCCCTGGAAATTA	SI03195248	TACGTGCAGGTGTCTCGTGAA	SI03225971
900097-2	C9	4780	*NFE2L2*	AAGGATTATTATGACTGTTAA	SI03246614	CCCATTGATGTTTCTGATCTA	SI03246950	ATGCCCTCACCTGCTACTTTA	SI04320904	CCGGCATTTCACTAAACACAA	SI00657930
900097-2	C10	57109	*REXO4*	GAGGCTGTACGTCATGGTGAA	SI03218642	TTGAGCATTATCTAAATTAA	SI00764043	CACGCTCTGCATAATGACCTA	SI00764050	AAGGTACTATTTCTTGATCAT	SI00764064
900097-2	C11	8412	*BCAR3*	AAGGTATCAGTTATATGATAT	SI00053095	GCCCAACGAGTTTGAGTCAAA	SI00053102	CCGAGCGGCCACTCTGAGTAA	SI03080196	CCGGAACTCTGGCGTCAACTA	SI03081603
900097-2	D2	51056	*LAP3*	AAGCTTAATTTGCCCATTAAT	SI02777488	AAAGCTTAATTTGCCCATTAA	SI00112490	ATGGAGGAGGAGCTGCAACTATA	SI00112497	AGCAGGCGTGATGACCAACAA	SI03042739
900097-2	D3	11010	*GLIPR1*	CAGCACTAGCCCAAATTGCAA	SI00427980	TCCGATCAGAGGTGAAACCAA	SI00427987	CAGGAGGGAGATTACCCAACTT	SI00427994	CTGCGTTCGAATCCATAACAA	SI03208940
900097-2	D4	10163	*WASF2*	TAGGATTAGATCATTAGCTCA	SI02664312	TGGTTTGGTAATGATCTGCAA	SI02664319	CTCAGTTGTATTGCTACTCCA	SI00093611	CCGGCTTGAGGAGAGAGTGTA	SI00093618
900097-2	D5	6911	*TBX6*	CCCACAGATCACACAACTGAA	SI00740887	CAGACGGAACTACAACATGTA	SI00740894	GCCGAGGAGTTAATTATGTAA	SI00740901	AGCCGAGGAGTTAATTATGTA	SI00740908
900097-2	D6	9141	*PDCD5*	CAGAACAAGGTTTAATAGAAA	SI00681191	CAAGTCTAGGACAGAAGTTAA	SI00681198	ATGACGATTATTGAACTACAA	SI00681205	TGGCAAGATATGGACAACTAA	SI00681212

900097-2	D7	6161	*RPL32*	CAGGTAATCCACCACGGTTAA	SI00706251	AACCTAGTCATAAATATTTAT	SI00706265	AAAGAACAGGATGATGTACAA	SI00706272	CAGATTCTTACGGGATGCCTA	SI03169299
900097-2	D8	5430	*POLR2A*	CTCGGGAAGAACAAAGCTAAA	SI00688975	CACCACAGTCTCCAACCTATA	SI00688982	CCGACTTAAGGAGCTCATCAA	SI00688989	CAGGACATTCAGAACACTATT	SI00688996
900097-2	D9	5071	*PARK2*	CACTGTAAATTTAATAGATAA	SI00677845	CACGTTTAGAAATGATTTCAA	SI00677852	ATGGATTGGATTTCAGTTCAA	SI02661267	ATCGCATTAATCGAAATACCA	SI03150301
900097-2	D10	3084	*NRG1*	TCGGCTCAGGTTCCAAACTA	SI03116974	TGGCTGATTCTGGAGAGTATA	SI03120530	CTAGTTGGCTCTGAGATACTA	SI03088071	TATCACCATCGTGGAATCAAA	SI03112487
900097-2	D11	7314	*UBB*	CAGGATCCTGGTATCCGCTAA	SI00754243	CACCTGGTCCTGCGTCTGAGA	SI03059280	TAAAGGTTTCGTTGCATGGTA	SI03107328	TCCGCCAGCCCGGAGCATTTA	SI03115105
900097-2	E2	3166	*HMX1*	ATGAATCTACTTATTTGCGTA	SI00439656	CTCGCGGGTGCTGGTGGAGAA	SI00439663	CTCCTCCTTCCTCATCGAGAA	SI00439670	TGCCGGTGCTCTACCACGAAA	SI04342387
900097-2	E3	2954	*GSTZ1*	CGCGCTGAAATTTGGCGTGAA	SI00153678	ACGGTGCCCATCAATCTCATA	SI00153685	CCCTACCATCAGCTCCATCAA	SI03079181	TCGAATTGCTCTGGCCTTGAA	SI03115987
900097-2	E4	4326	*MMP17*	AAGGACAATAACGTAGAGGAA	SI02781086	CTGGGTGTTCAAGGACAATAA	SI00113897	CACAAGATCGTCTTCTTTAAA	SI00113904	ATGACAGGACTTATTTCTTTA	SI00113911
900097-2	E5	83416	*FCRL5*	AAGGTCGGCACTTGACACCAA	SI03134425	CCCACCTATCACAATGTACCA	SI03182795	CACTACAGATATGCAACTAAA	SI02658047	CAGTGAGATGGTGACACTGAA	SI02658054
900097-2	E6	8633	*UNC5C*	CGGCCTCGTCTTGCAATCCAA	SI00055174	TTGAATTTAGCTATCAACCTA	SI00055181	CCCATGTACATGCCCACTTTA	SI00055188	CGCCATGGAGCAAGTGGTCTA	SI03084382
900097-2	E7	8050	*PDHX*	CTGGTCAAAGATGACATTAAA	SI00681555	CTAGATGTAAATCAAAGTATA	SI00681562	TCGGAATATATTTAAATAAAT	SI00681569	AAGAGATTAACTGAATCTAAA	SI00681576
900097-2	E8	6871	*TADA2L*	AGCGATCATACTTATGAAATA	SI00738479	CCAGGTTAAAGGAGAGACAAA	SI00738486	ACCTTAGAAAGTTTCAATTAA	SI00738493	CAAAGCGATCATACTTATGAA	SI00738500
900097-2	E9	83540	*NUF2*	CAGGAGCTACAACAATCACTA	SI00140630	CACAGTAATTGAGGATTGCAA	SI00140637	AAGCATGCCGTGAAACGTATA	SI00140644	AAGATACGGTCCAGAAGCTTA	SI03032141
900097-2	E10	1786	*DNMT1*	AACGGTGCTCATGCTTACAAC	SI00300062	CCCAATGAGACTGACATCAAA	SI02663409	CGGGAAGTGAATGGACGTCTA	SI02663416	CCCATCGGTTTCCGCGCGAAA	SI02626169
900097-2	E11	14	*AAMP*	GAGGAAGAGATACTAGTTAAA	SI00289051	CTGGATGTGGAAAGTCCCGAA	SI00289065	CTGGACTTTGCCCTCAGCAAA	SI00289072	CCGCATGGAGTCCGAATCGGA	SI03188815
900097-2	F2	5202	*PFDN2*	CTGGTGGAGCGAACTGTCAAA	SI00682850	CAGCGAGGCCTGGCATCCAAA	SI00682857	AAGGAGGTAGATGAAACTCGT	SI00682864	CAACAAGGAGCAGATACAGAA	SI03155971
900097-2	F3	1117	*CHI3L2*	CAAGGTTATCATCAAGGACAA	SI00345849	AACCAAGAATCCCAAACTGAA	SI00345856	ATGATTGATAACAGCTATCAA	SI00345863	AGGGCCAAGCTCCTACTACAA	SI04231626
900097-2	F4	8661	*EIF3A*	GAGGATCTAGATAATATTCAA	SI03019107	CAGGAACATGAACAAATCAAA	SI00377671	TTGGCTTGAAGTAGAATTTAA	SI00377678	TACGAGGAACAGAGAATTAAA	SI02656752
900097-2	F5	5914	*RARA*	CTCCACCAAGTGCATCATTAA	SI00019369	CCACATCTTCATCACCAGCAA	SI00019376	CACTGAGATCTACTGGATAAA	SI03062318	CAGGAAATGTTGGAGAACTCA	SI03068821
900097-2	F6	54971	*BANP*	CACTGTAATTATGCATTTCTA	SI00310268	CTGGATGATGTCGGAACACGA	SI00310275	TGCTATGGTGTTTATAACAAA	SI00310282	CCCGACAAACACCACGAGAAT	SI03184167
900097-2	F7	50640	*PNPLA8*	CTAGATGAAAGTCGAAATGAA	SI00448056	AAGGGTTGAAATACATAGAAA	SI00448070	CACAAGTTCTATAACCACAAA	SI00448077	AAGGTCTGTTTAGAAGATCAA	SI02657487
900097-2	F8	10581	*IFITM2*	ACCATGAACCACATTGTGCAA	SI00446012	CAGCGATAGATCAGGAGGCAT	SI00446033	TCCCACGTACTCTATCTTCCA	SI03231263	CTGGTGCTGCTGTGACTTCAA	SI04429705
900097-2	F9	3685	*ITGAV*	TAGCATGATGTTACAGGAATA	SI00034342	CACATACATGATACCATCCAA	SI00034349	CAGGCAATAGAGATTATGCCA	SI03070165	GAGTGCAATCTTGTACGTAAA	SI03104374
900097-2	F10	8662	*EIF3B*	CGGGAAGATTGAACTCATCAA	SI00377825	TTCGGTGATTGTAGTGGACAA	SI00377832	CAGGTTGAACATGGAAATAAA	SI00377839	CCGCACTTCCATATTCTGGAA	SI00377846
900097-2	F11	10578	*GNLY*	CAGGGTGACCTGTTGACCAAA	SI02652839	CAGAAATTTCATGAGGAGGTA	SI02652846	CCAGGGTGACCTGTTGACCAA	SI02652853	ACTGAAGAAGATGGTGGATAA	SI02652860
900097-2	G2	8993	*PGLYRP1*	GAGGTCCAACTATGTGCTCAA	SI00072261	CAGCTTCATGGGCAACTACAT	SI02634352	CTGGTGCGACGTGGGCTACAA	SI02634359	TACAACTTCCTGATTGGAGAA	SI03107853
900097-2	G3	64231	*MS4A6A*	AAGGGCTGATGACTTCAGAAA	SI03036054	CACATCCTGTATAGCTGTAAA	SI03056585	CACGCAGAAATCAAAGTTATT	SI00133364	ACCGTTGATGGGACTGAGAAA	SI02645181
900097-2	G4	10881	*ACTL7A*	CTGCTCGGAGATGTTCTTCAA	SI00291431	CACAAGAACCTACTGAATCAA	SI00291438	CAAGTGTGACATCGCCCTCAA	SI00291445	CAGCTGGGCCTCCACACCCAA	SI00291452
900097-2	G5	5494	*PPM1A*	AAGCGTGATTTCAAACCATAA	SI02659258	TTCCATGAGTATTGCAGGTAA	SI02659265	TCCGCTGGAGAAAGAACGAAT	SI03115203	ACCTAGAGGATCAAGACATAA	SI03040625
900097-2	G6	6167	*RPL37*	TCCGTGAAGGAACAACACCTA	SI04264176	AAGGTGGCTAGCTAAGTGAAA	SI04271127	AAGGAACAACACCTAAACCCA	SI03131856	CTGGGCTAGCTTCATATCAGA	SI03213140
900097-2	G7	3596	*IL13*	CAGCATGGTATGGAGCATCAA	SI00033831	TTCATTGACTATCAAACTGAA	SI00033838	AACAAGTTGTTTCATTGACTA	SI00033845	CAGCTCTCAGCCAACGAGTAA	SI00033852
900097-2	G8	6597	*SMARCA4*	CCGCGCTACAACCAGATGAAA	SI00047579	TCACTGGATGTCAAACAGTAA	SI00047586	CCCGTGGACTTCAAGAAGATA	SI02630208	CTGGGCGTACGAGTTTGACAA	SI03098998
900097-2	G9	10579	*TACC2*	CTGTATTTCCTTTCTAAATAA	SI00738395	TGGATTTAATATGAACATGTA	SI00738402	CACCTTTGTAAACGAGACCAA	SI00738416	GACGCTGGAGCAGAAGATTAA	SI03216570
900097-2	G10	10370	*CITED2*	TGGAATGTCGTCAGTGGCAAA	SI00084252	CAGATTTACGAGTGAAAGGAA	SI00084266	CTCCCTTATGTAGTTGAAATA	SI00084273	CAGAAGCTCAACAACCAGTAT	SI03063102
900097-2	G11	6830	*SUPT6H*	TAGGAGCATCTTTGAAATGTA	SI00736575	CAGCACAATTCAGAAGATCAA	SI00736582	CAGGACAATTTCCCTGAACTA	SI00736589	TCAGTGTATGCTAGGCAACAA	SI03230171
900097-3	B2	80326	*WNT10A*	CTGAGCTGAAGTAATTCCATA	SI00763350	CATGGGCAATAAACAAGTAAA	SI00763357	ATCCACGAATGCCAACACCAA	SI00763364	CACGCTCATCCGGCCGCACAA	SI04312742
900097-3	B3	54700	*RRN3*	TAGAAAGACATTTACATATAA	SI00708330	TCCGGGTTTGGTGGAAAGTAA	SI00708337	AAGGACATAGTGGAAGATGAA	SI00708344	CGCGACCTGATAAACATCTTT	SI03194296
900097-3	B4	6867	*TACC1*	AAGGCTCAGATCTTAATTAAA	SI00738367	CAGAAGAACCAAGAAATTGAA	SI00738381	CAGGAGATATGAGAACCTGAA	SI00738388	CTCGGCAGTCCTCGGAACAAA	SI03204635
900097-3	B5	4001	*LMNB1*	AACGCGCTTGGTAGAGGTGGA	1022061	CAGCTTTCTAATCTACAGAAA	SI02656248	TCAGTTATAAATATACCTCAA	SI02656255	CAGGGAGAGGAGGTTGCTCAA	SI02656262
900097-3	B6	3073	*HEXA*	CCCGAACAACTTTCAATTCCA	SI00011039	AGGGCTGGGAATGAAACCTAT	SI03046029	CAAGGCGTTGGTATATGGAAA	SI03054562	CCCTGGTACCTGAACCGTATA	SI03079622
900097-3	B7	7316	*UBC*	GAGGTTGATCTTTGCCGGAAA	SI00754271	GAGGTTGATCTTTGCTGGGAA	SI00754278	AACGTCAAAGCAAAGATCCAA	SI00754285	ATCGCTGTGATCGTCACTTGA	SI03048423
900097-3	B8	5092	*PCBD1*	ATGGTTTAACGTGTACAACAA	SI00006139	CTGGCCAGCTTCATCGAACAA	SI00006146	ACCACCATCCTGAATGGTTTA	SI00006153	AGGCCGTGATGCCATCTTCAA	SI00006160
900097-3	B9	6651	*SON*	ATGATGTTGATTTATCTTTAA	SI00729246	AAAGATATTCATCTTGATTTA	SI00729260	CAGCGCTGGAATCCTATAATA	SI03171861	TAGGTCTTTCGTGGTCAGTAA	SI03229009
900097-3	B10	9132	*KCNQ4*	CGGGCATCTCTGAGACTCAAA	SI00067186	CCGCTCCATCAGGATTCTCAA	SI00067193	GACGATTACATTGACAACCAT	SI00067200	CTGGTACTACTATGACAGTAT	SI00067207
900097-3	B11	64783	*RBM15*	AAGGTGATAGTTGGGCATATA	SI00699475	TTCGGTGATGTAAGTGTGAAA	SI00699482	ACCCGCAACAATGAAGGGAAA	SI00699489	CCGGACGCTCTTCTTGGGCAA	SI00699496
900097-3	C2	5888	*RAD51*	AAGGGAATTAGTGAAGCCAAA	SI02663682	CAGGATAAAGCTTCCGGGAAA	SI00045010	CACTTCTAAATTAATGGTAAA	SI02629837	CACGGTTAGAGCAGTGTGGCA	SI03061338
900097-3	C3	7123	*CLEC3B*	CAAGAAGATTGTAAATGCCAA	SI00748559	CTGCCAGTTCGGGATCGTGTA	SI00748566	CAACGGCAAGTGGTTCGACAA	SI00748573	CTCGGAGAACGACGCCCTGTA	SI00748580
900097-3	C4	2524	*FUT2*	CAGCGGCTAGCGAAGATTCAA	SI00010892	CACGGGCGGAGACACCATCTA	SI00010899	CACCATCTACCTGGCCAATTA	SI00010906	CAGTGCTAGCCTCAACATCAA	SI00010913
900097-3	C5	9092	*SART1*	CTCAACCTTCATATTAAATAA	SI00710787	CCCGTGTACAGTGACCCAGAA	SI00710794	AAGGAGAAGGACGGCTACAAA	SI00710801	CAGCATCGAGGAGACTAACAA	SI00710808
900097-3	C6	5456	*POU3F4*	ACAGTTCGCCAAACAATTCAA	SI00006748	TGGTTCTGTAATCGAAGACAA	SI03021214	CAGAAACTTCTCCAAAGTGAT	SI03062675	CCCGACCAGCATTGACAAGAT	SI03077410
900097-3	C7	6883	*TAF12*	CTCCAATTTCTCATCCATAAA	SI00738598	CAGGGAATGGACAGCAATGTA	SI00738605	CCAGAATAAAGAAGTTATTAA	SI00738612	CAGGTATTGACCAAGAAGAAA	SI03177167
900097-3	C8	11096	*ADAMTS5*	CAGTGGTGACCTTGCAATATA	SI00093940	CCCGTTAACTTCATAGCAAAT	SI00093947	AACCATTAGATGTCCGTTATA	SI00093954	AAGCACTTGATTGTACCCGTA	SI00093961
900097-3	C9	57292	*KIR2DL5A*	CAGGTCTATTTGGGAAACCTT	SI00126910	AGGGAGGACTCTGATGATCAA	SI02694510	CAGCGTCATGGAACATTCCA	SI02717477	CACCATGTCGCTCATGGTCAT	SI04346776
900097-3	C10	4622	*MYH4*	CTGGATCATCTTGAAACTCTA	SI00652743	CTGGGTTTCACTGCTGATGAA	SI00652750	CGGCAATGAGTTCGTAACCAA	SI00652757	CAACTTAATGAGAAACTCAAA	SI00652764
900097-3	C11	10213	*PSMD14*	CAGGCATTAATTCATGGACTA	SI00081396	TTGGATACTGTCGTATTTAAA	SI00081410	ACAGACATTATTACTCCATTA	SI00081417	CTGGGTCACTTAAACAAGCCA	SI03099152
900097-3	D2	84159	*ARID5B*	CTGGGAGGATATGAAACAATA	SI00303177	AAGGATAAACTCTTAGAGAAA	SI00303184	CCCGCCGTCAGTGGAAACATA	SI00303198	CACCTAATTTATATACAGGTA	SI03162362
900097-3	D3	4849	*CNOT3*	CTCGGACACTGTGGAATTCTA	SI00349601	ACGGCTGAGGGACCAAATCAA	SI00349608	AAGAAGGAGATTAAGAAGCTA	SI00349615	GACACTCTTCTTCATCTTCTA	SI00349622
900097-3	D4	5865	*RAB3B*	CAGCACTAGACTAACATAACA	SI00044814	CGGGTGCAGAATCACTTTACA	SI00044821	CCGGACCATCACAACAGCCTA	SI00044828	CAGAGAGCTCAGACTCTCTAA	SI00044835
900097-3	D5	7517	*XRCC3*	CAGAATTATTGCTGCAATTAA	SI00077112	GAGACACTTAAGGGAAATTAA	SI00077119	AAGCCAAACTGAAATCGGTAA	SI00077126	CCGCTGTGAATTTGACAGCCA	SI00077133
900097-3	D6	51529	*ANAPC11*	CCGCCAGGAATGGAAGTTCAA	SI02777691	ATGAATAAACATCTCCGTGAA	SI00116270	AAGGAGCAGGATCAGACGGTA	SI00116277	CAGGCCCATTTGAGATCTTTG	SI02642304
900097-3	D7	84365	*MKI67IP*	ATGGTTGAAGCTGGAAGTTTA	SI00646023	CAAACAATAACCAGCAATTAA	SI00646037	ACTAGGTACAATGGAATACAA	SI03142356	TGGATAGAAAGTTGACCACTA	SI03237668
900097-3	D8	10771	*ZMYND11*	CCGGATGAAGTCGGACCACAA	SI00090412	ACAGAGCGTGTTGTCCGAGAA	SI03037706	ACCGAAGTTATGAAGAGTTCA	SI03039694	CCACCAGTAATGAGCAGCTAA	SI03074470
900097-3	D9	5877	*RABIF*	CTTCTCTCGCCGACAGCTTTT	SI00301595	TAGAAGGAAGTTTATTATATA	SI02651075	CAGCTCCATCTCCAAAGATAA	SI02651089	CAGTTTGATCTGAAAGAAATA	SI02651096
900097-3	D10	54361	*WNT4*	CAGGGAAGGCCGATAATTTAA	SI00139160	CAGACCTAAGGTGGAGTAACA	SI00139167	CTCATGTTATTTATTGCCGAA	SI00139174	CAAGCGGAACCTGGAAGTCAT	SI03054219
900097-3	D11	1203	*CLN5*	CACGTTTAATATGTAAATGTA	SI00348425	TAGGATTGCTGTAAAGATGTA	SI00348432	ATGGTTCATATCAGTTATGTA	SI00348439	CAGAAGTACATTAACTGGCAA	SI00348446
900097-3	E2	145282	*MIPOL1*	CAGGAGCTTCATCATGTGAAA	SI00645799	AAGAATCATAATGCTAACAAA	SI00645806	CACAGTTATCTTGAACAAGAA	SI00645820	CAGGAATTACTGAACAGAATA	SI03173716
900097-3	E3	648	*BMI1*	ATGGGTCATCAGCAACTTCTT	SI03051874	CAAGACCAGACCACTACTGAA	SI03053610	TTGGATCGGAAAGTAAACAAA	SI00073311	CAGAGTTCGACCTACTTGTAA	SI00073325
900097-3	E4	6191	*RPS4X*	CAGATCTTTGCTAGTAATTAA	SI00707875	CGGGAGAGAATTTCCGTCTGA	SI00707896	CTGGAGGTGCTAACCTAGGAA	SI03210851	AAGGGTCGCTTTGCTGTACAT	SI04136062
900097-3	E5	412	*STS*	TAGATTAATGACGTATACATA	SI00007707	ACCCATCACGACCCACCTTTA	SI00007721	CAGCATCAAGGCCGAACATCA	SI00007728	CGGAAGTAATGGGATCTATAA	SI03085628
900097-3	E6	55743	*CHFR*	AACCAGAGGTTTGACATGGAA	SI02778125	AACAGTGATTAACAAGCTGAA	SI00121107	TCCCGTGGAGCGGATCTGTAA	SI00121121	TCACCACGCCATGAAATTCAA	SI00121128
900097-3	E7	8945	*BTRC*	CAGGATGAGCAACAACAGTAA	SI02632322	CACGTTGATTCACCATTGTGA	SI03061807	CTGGTTTCAGGTGGAACTTAA	SI00058072	CTGGAGGCAGATGACATCTAA	SI00058079
900097-3	E8	10524	*HTATIP*	CACCACATTGCCTGTCCTCTA	SI02780897	CGGCGGAACCAGGACAACGAA	SI00088053	CACCCGGATGAAGAACATTGA	SI00162981	CTGATCGAGTTCAGCTATGAA	SI02637383
900097-3	E9	4594	*MUT*	CTGGGCTCACTTAATAGAGAA	SI00005565	AAGAGAGATACTATGGACTTA	SI00005572	AGGGAATACCTAAACTTCGAA	SI00005579	CACGTGGACCATATCCTACCA	SI03061639
900097-3	E10	1184	*CLCN5*	ATCCTTGTTCCTGTTACAATA	SI00001659	TAAGTGGACTCTGGTTATCAA	SI00001666	CACCGAGAGATTACCAATAAA	SI00001673	CAGGTTCGTTAGCTGGTTTGA	SI03072482
900097-3	E11	64147	*KIF9*	CAGGACTTGGTTATGAGACA	SI00133280	AACAGACAGACTGGTCGTTTA	SI00133287	CACGCTCTGAAGGACTCGTTA	SI02649850	CCCGGACCTTATCAGAGGAAA	SI02649857
900097-3	F2	6227	*RPS21*	TGGAATATTTGTCATAAATAA	SI00707483	CGGCGAGTTCGTGGACCTGTA	SI00707490	CAAGGTCACAGGCAGGTTTAA	SI00707497	GCGGAAATGCTCCGCTAGCAA	SI00707504
900097-3	F3	2821	*GPI*	CCGCGTCTGGTATGTCTCCAA	SI02664011	GAGTTTGGAATTGACCCTCAA	SI02664018	ACGGAAGGGTCTGCATCACAA	SI00003738	GAGCTTGATGGCAGTGCTCAA	SI03103268
900097-3	F4	6924	*TCEB3*	TAGCATCTTTGTAAACTATAA	SI00741279	TACAATCATGTATTAATTGAA	SI00741286	CACGAGCATGTTGGAAGCTTT	SI00741293	CAGAATGAATTCCAAGATGCA	SI00741300
900097-3	F5	1728	*NQO1*	CCAGCAGACGCCCGAATTCAA	SI00018137	TTGGGATGAGACACCACTGTA	SI03025218	CAGATATTGTGGCTGAACAAA	SI03064621	CCGAGTCTGTTCTGGCTTATA	SI03080287
900097-3	F6	6525	*SMTN*	CAGCACCATGATGCAAACCAA	SI00727531	CACATTCACCATCGAGATCAA	SI00727538	CACGGACTTTGAAGAGCGGAA	SI00727552	TCCGTCTGTCGGGTCCCGAAA	SI03232803
900097-3	F7	1871	*E2F3*	TTGCGTTACTTTAAGTACTAA	SI00030408	TTGGGAGTAGGCAAACTACTA	SI00030422	TACGAAGTCCAGATAGTCCAA	SI00030429	CAGCGATCTCTTCGATGCTTA	SI03066966
900097-3	F8	50863	*HNT*	CAGCAACCAATCAGATATATA	SI00439971	AAGAGAAGTATGTTTGTTAAA	SI00439978	ACCATACATTTCAGAAGCCAA	SI00439985	CCCGGCTCCGAAACTTACAAA	SI03185707
900097-3	F9	6890	*TAP1*	AGGGCGAAGCCCAGAAGTTTA	SI00012411	CAGCCGATACCTTCACTCGAA	SI00012418	TACCGCCTTCGTTGTCAGTTA	SI00012425	CGGGATCTATAACAACACCAT	SI00012432
900097-3	F10	2953	*GSTT2*	CAAGGAGTTCTTGCAGATCAA	SI00431844	AAGGATTCATTCTGTTACTTA	SI00431851	AAGCTCGGCCATCCTGATTTA	SI00431858	AGAGACTAAATCTGCAATATA	SI00431865
900097-3	F11	5333	*PLCD1*	GCCGGTGCTGTCGCTACTCAA	SI00085911	ACGGAGTTTGCGTTTGAGGTA	SI00085918	CCGCTTCTTGGTGGAAGATTA	SI00085925	AAGGACCAGCGCAATACACTA	SI03034430
900097-3	G2	10868	*USP20*	CCGGATGATGAAACAGGGATA	SI00090965	CAGGCTTGTTGAAACGACCAA	SI00090972	ACCGTCGTACGTGCTCAAGAA	SI00090979	TCGAGTGACACGGATGAGAAA	SI00090986
900097-3	G3	26269	*FBXO8*	ACAGCTGTTAACGAATGATAA	SI00097895	GTGCATTAGATTTGCCTGAAA	SI00097909	CCGTTAAGAGTCGGAGCCTAA	SI00097916	CAGGACCTTGCGAATGATGAA	SI03069283
900097-3	G4	6232	*RPS27*	ACGGTCTTTAGCCATGCACAA	SI00707623	AGAGGAGAAGAGGAAACACAA	SI00707630	TCAGGAGGAAGCAGCACTAAA	SI00707637	CTCCTTCATCCCTCTCCAGAA	SI00707644
900097-3	G5	6223	*RPS19*	CCCGACTTGTGCGCCCGGGAA	SI03077522	CTCTCTATCCTCTCTCTATTA	SI03092460	TACCGTCAAGCTGGCCAAGCA	SI03109078	TCCAGCCACGAACGACGCAAA	SI03113649
900097-3	G6	8302	*KLRC4*	TAGCTTCTTAAGTGCAGTTAT	SI00102004	ATGTTTCAACACTTTACTAAA	SI00102011	ATGGAAACCCATGCTTCTATA	SI03153052	TAGGAAATTAGTACCAGACTT	SI03228050
900097-3	G7	23521	*RPL13A*	CAGCATGAGCTTGCTGTTGTA	SI00705635	CAGGGTATTTCTAGAAGCAGA	SI00705642	AACCAGTTACTATGAGTGAAA	SI00705649	TGCAGCTGTTAGAATGTGCAA	SI00705656
900097-3	G8	9924	*PAN2*	CAGATACAACCTGAACATCAA	SI00758807	CTGGAGGACTTTGACTTCAAA	SI00758814	CACCATCATGAGACAGACAAA	SI00758821	TTCAATGACTTTCTTATTGAA	SI00758828

900097-3	G9	7067	*THRA*	CCCGATGACATTGGCCAGTCA	SI00049798	CAGCGAGTTTACCAAGATCAT	SI00049805	TCCCACCTATTCCTGCAAATA	SI03114041	TCGCCGCACAATCCAGAAGAA	SI03116470
900097-3	G10	8239	*USP9X*	CCGCCAGATAGCACAACGATA	SI00066584	CAGCTAGTATTTAGCCCAAAT	SI00066598	CCGCCTGCAGTGGAAAGTGTA	SI00066605	GACGATGTATTCTCAATCGTA	SI03101973
900097-3	G11	8846	*ALKBH1*	CTGGATGACCAGAATAGCGAA	SI00294595	CACAGTGGTGACATCATGATA	SI00294602	CTGGGTGAAACAGTGCCTTAA	SI00294609	CTGGGTTTCCTCTCAGAGCAA	SI00294616
900097-4	B2	10885	*WDR3*	CCGGGATGTTATCGGCTTCAA	SI00091868	AAGAGTTGGCGTCATCTTAAA	SI00091875	CCAGCGGGTGACTAATATAAA	SI00091889	TCCGCTGATAGGAATGTGAAA	SI02638055
900097-4	B3	9324	*HMGN3*	GAGGGCAAAGATGGATCCAAA	SI00061579	CTGCTGACTATTTGTGGTTTA	SI00061586	AACCTGAACCCAAACCAAGAA	SI03029054	CAGAGGTGCTAAAGGGAAGAA	SI03064229
900097-4	B4	8643	*PTCH2*	CCGCACCTGGCTGCACTATTA	SI00055230	CTGGCGATGATGACAGTGGAA	SI00055237	CAGGTACTTCTCCCTGTACGA	SI03071796	CCCGGATATCCAGTGGACCAA	SI03078369
900097-4	B5	29920	*PYCR2*	AAGAGTGTTATTTAATGCTAA	SI00696591	CAGGATTAGACTGGGTTTATA	SI00696598	CGGGCTTTCTCTGAATTGGTA	SI00696605	CAGCACAAGGAGACGGTGAA	SI00696612
900097-4	B6	5742	*PTGS1*	CATTGCCATGGAGTTCAACCA	SI00301511	AATACCGCAACCGCATTGCCA	SI00301518	AATTGCCATGGAGTTCAACCA	SI02655016	CTGGTTCTGGGAGTTTGTCAA	SI03100132
900097-4	B7	10531	*PITRM1*	TAGGTTCTTCACGTACGGTAA	SI00108647	CCGGTGAATTACGTGGGTGAA	SI00108654	CAGCGTTGAAAGTTTCCGATA	SI00108661	TCCGATATTGAACCCACCATA	SI00108668
900097-4	B8	9499	*MYOT*	CAGCAAATATTTAGCACTTAA	SI00752899	CAGGTTATAAATTAGTATCAA	SI00752906	AAGAGTTACTTTACTGATAAA	SI00752913	CAGGAACAAATCATTATAGTA	SI00752920
900097-4	B9	3442	*IFNA5*	CAGGAGGACTTTGATGATAAT	SI00033474	ATCCTTCTCTTTATCAGCAAA	SI00033481	ATGCAGGAGGTTGGAGTGGAA	SI00033488	TTGGGATGAGACACTTCTAGA	SI03025225
900097-4	B10	672	*BRCA1*	CAGCAGTTTATTACTCACTAA	SI02654575	CAGGAAATGGCTGAACTAGAA	SI02664361	ACCATACAGCTTCATAAATAA	SI02664368	AACCTATCGGAAGAAGGCAAG	SI00299495
900097-4	B11	3978	*LIG1*	CCGGACGTTTGAGAAGATCGA	SI02663542	CCCGTCATTTCTTTCAATAAA	SI02757482	CAGGCTCTGACCCTGAAGATA	SI00005061	CCTGTTTGTACCGGAAGCAAA	SI00005068
900097-4	C2	10979	*FERMT2*	AAGGATGGCTTGATTCCTCAA	SI00686777	ACCGTAGAGTTTGCAGATGAA	SI00686784	TCCGTCGTGCTTGATTTGTTT	SI04331768	CTCCACGGCCATAACAATATT	SI04376029
900097-4	C3	1840	*DTX1*	GACCAAGAAGAAGCACCTTAA	SI00374577	GTGGAGTCAGCCAAGATTTA	SI00374584	CCGGGAAGAAGTTCACCGCAA	SI00374598	CTGCTACCTCATCTACTTCAA	SI03095743
900097-4	C4	183	*AGT*	CAGAGTCTACCCAACAGCTTA	SI00000350	ACCGACCAGCTTGTTTGTGAA	SI00000357	CTCCGTGTAGTGTCTGTAATA	SI00000371	CAGGCTGTACAGGGCCTGCTA	SI03070921
900097-4	C5	3265	*HRAS*	CCGGAAGCAGGTGGTCATTGA	SI02654806	CACAGATGGGATCACAGTAAA	SI02662030	AGGAGCGATGACGGAATATAA	SI02662576	CTGAGGAGCGATGACGGAATA	SI04435368
900097-4	C6	26190	*FBXW2*	CACGCAGTGCACAATCATTTA	SI00097706	CAGAGTGTATGCACTTTACTA	SI00097713	ACGGAATGGGTCACCAAGGTA	SI00097720	CCAGTGGGACTTTGCCAGTTA	SI00097727
900097-4	C7	7480	*WNT10B*	ACGAATGCGAATCCACAACAA	SI00051800	CAGGATCGGCTGAGGGCCAAA	SI00051807	AAACCTGAAGCGGAAATGCAA	SI00051814	CAGGCACTGTTTGGAGCCATA	SI00051821
900097-4	C8	3646	*EIF3E*	AAGCTGGCCTCTGAAATCTTA	SI02661981	CCCAAAGGTCGCGATAATATT	SI02662499	CAGGGATGGTAGGATGCTCTT	SI04228644	AAGGAATTATTACAAGGTAAA	SI00377755
900097-4	C9	51514	*DTL*	CCGAGTCTACTGGGTATAACA	SI02777684	CTGGGATACCAGGTGCAACAA	SI03098718	TGGCGCTTGAATAGAGGCTTA	SI03120334	CACCATAGCCTTAACTGAATA	SI00116221
900097-4	C10	898	*CCNE1*	AAGGCAAACGTGACCGTTTTT	SI00299691	CAGGGTATCAGTGGTGCGACA	SI00024024	ACCGGGTTTACCCAAACTCAA	SI02625693	AAGGCCCTTAAGTGGCGTTTA	SI03035319
900097-4	C11	9412	*MED21*	CAGTTTATTATTGCCAAATAA	SI00736631	CAACAATTACAGAAACATTAA	SI00736638	CTGGAGGATGTTGTTTATCGA	SI00736645	CACTGGAAAGGTGGAATTGAA	SI00736652
900097-4	D2	6756	*SSX1*	ATCCTTATAATTGATGAGCAA	SI00733887	AACGGTGAAACTTGAGCGTCA	SI00733894	CTGCTTTGTCTCTGAAGTGAG	SI00733901	AAACGATGGGAAACAACTGCA	SI00733908
900097-4	D3	59277	*NTN4*	CAGCATGACATTTAGCAGTAA	SI00662235	AAGGCATTCTGTGGAATGAAA	SI00662242	CCGAGGAAAGCGAACATTATA	SI00662249	CTCAGGTAAATGCGAATGTAA	SI00662256
900097-4	D4	9266	*PSCD2*	CTGGTTTATCCTCACAGACAA	SI00061292	CACGCTGTTGGTAATCTTATT	SI00061299	ATGGAGGACGGCGTCTATGAA	SI03050894	CAGTAAGACCTTGCAACGGAA	SI03072629
900097-4	D5	10565	*ARFGEF1*	CAGGATTTATTTAGAATTGTT	SI00300825	TCCGGTATTACTGAAATGAAA	SI00300832	AGGGATAGATTTATTTAATAA	SI00300839	CAAGATTGTGATGATACTGAA	SI00300853
900097-4	D6	2057	*EPOR*	CCAGTGCAGACTCAAGACTTA	SI02780351	CAGATGATCAGGGATCCAATA	SI02780400	CCGCCGGGCTCTGAAGCAGAA	SI04435060	CACCGAGTGTGTGCTGAGCAA	SI00002408
900097-4	D7	2107	*ETF1*	CAGCATGATATCATTGATCAT	SI00381332	TCGGTGATATATATTGCATTA	SI00381339	AGGGAGTATGTCTTAAATGTA	SI00381346	TTGGAACTGCATCTAACATTA	SI03244416
900097-4	D8	9509	*ADAMTS2*	CAGCTGCACCCTGAACCATGA	SI03068310	CTGGGCCTACCTCCAGCAGAA	SI03098935	GAGGAGGAGTTCTTCATCGAA	SI03103555	GCGGATCATCCTCCTGAGCTA	SI03105368
900097-4	D9	3749	*KCNC4*	CACGCTGGACTTCGTCAAGAA	SI00070868	ACCCATGTACTGCAAGTCTGA	SI03039148	AGAGGGTATGATCGAGAGGAA	SI03042354	CCGGCGGGAGGTAGAGACAGA	SI03082233
900097-4	D10	6134	*RPL10*	TGGCCTTATGTCAGTTGTCTA	SI00083678	CAGAAACAGGTTGACAACTCA	SI00083685	CAGCAAAGCCTTGCAATCCCA	SI02636473	ACCAAGTTCAATGCTGATGAA	SI04380957
900097-4	D11	7514	*XPO1*	CCCATTGTAAAGCGACTTCAA	SI00051856	TACATGTTACTCCCTAATCAA	SI00051863	TTCTCAGAATATGAATACGAA	SI03023342	ATGGTTAGTCGAATGGCTAAA	SI03052210
900097-4	E2	1719	*DHFR*	AACCTCCACAAGGAGCTCATT	SI00299992	TACGGAGAAACTGAACTGAGA	SI00015729	TTGAGGTACGGAGAAACTGAA	SI00015736	AAGGTAACTAACGGAAGGAAA	SI00015750
900097-4	E3	4599	*MX1*	TCCGTTAGCCGTGGTGATTTA	SI02781093	TAAGATCAAGACACTCATCAA	SI00038185	TAGAGCTTACTTTGCAAAGAA	SI00038192	AGGAGACTCACCAGAGAATAA	SI04435956
900097-4	E4	7321	*UBE2D1*	TCCACTGGCAAGCCACTATTA	SI00302085	CAGAAAGAATTGAGTGATCTA	SI02651201	ATGGTGTAGTCATTAGCAAA	SI02651208	TAGCTATATTATTATAAGCTA	SI02651222
900097-4	E5	26091	*HERC4*	GAGGGAATTATTGGATCCTAA	SI00111531	TAGAAATACTACATAGGGTAA	SI00111545	CAGGTTGCTTGTGGTTACTAT	SI03072538	TAGGACAGCTAGGTCATGAAA	SI03111528
900097-4	E6	2736	*GLI2*	TAGGGAGCATTTGGGTTTGAA	SI00074844	CCCGGACCACAGCATGCTCTA	SI03078215	CTCGCTAGTGGCCTACATCAA	SI03091445	TACCCGGGCTACAGTCCGCAA	SI03108847
900097-4	E7	8288	*EPX*	CCCGGAATTTGGCACAGCTTA	SI00010668	AACCGTCAGGATGCCATGTTA	SI00010675	CTCGAATTATATGTGACAATA	SI00010689	ACCGGCTGTTTCGGCAAGTGA	SI03040247
900097-4	E8	4928	*NUP98*	CTGGAGTTAGCACTAACATAA	SI00115332	CAGTGTATTACTGCTATGAAA	SI02635115	AACCCTATTGCCAAACCTATT	SI03028578	CTCACTAAGGTTGGTTACTAT	SI03088491
900097-4	E9	9351	*SLC9A3R2*	CGCGCTCTAAATAATTGCAAT	SI00068376	TCGAGCCATGCGAGTCAACAA	SI00068383	CCAGGTGGTGCAAAGGATCAA	SI03075562	CGCGCCACAGATGGACTGGAA	SI03084977
900097-4	E10	162466	*PHOSPHO1*	TACAACTATAAAGGAGGTGAA	SI00161168	CGCCAACATGTGCAAGCACAA	SI00161175	CCGTCCCTATCTATTCAGTTA	SI00161182	TCGGGTCCGGACAGCCAGTAA	SI00161189
900097-4	E11	5934	*RBL2*	ACCCTATATTAGGAAACTTTA	SI02664473	TGCGGAGATGCTTTACTATAA	SI02664480	GTGCAAGGTATTGCCAATGAA	SI00079541	CTGATGAGTTACAGCCTGTTA	SI00079548
900097-4	F2	112950	*MED8*	GCCGAACAAGCAGACCTTTAA	SI00019775	AAGCAGCTTGAGGCATCATTA	SI03032883	CAGATCCAGAGCTTGAATAAA	SI03064691	CAGGCAGGTCAACCAGGGAAA	SI00019768
900097-4	F3	10694	*CCT8*	AAACGTTGGATTAGATATTGA	SI03026212	AAGGTGATGTAACATCTGTCA	SI03036460	TAGAGTGGATCAGATCATCAT	SI03110681	TCGTACCTCCATAATGAGTAA	SI03117261
900097-4	F4	2734	*GLG1*	CCGGATTGAGATGTGGAGTTA	SI00097993	CCGCTTGGAGCCAGATCTATA	SI00098000	GTGGTGGAGTGTAGAGATATA	SI00098007	CACGCCTACCGCACTGAGGAA	SI03060218
900097-4	F5	84925	*DIRC2*	ACAGATTATCACATTAATTTA	SI00364133	CACAGACTTGAAGGAGTTTAA	SI00364140	CTGATTACTTGTAATTATGAA	SI00364147	CTGGTCTAAGATGCATACCTA	SI00364154
900097-4	F6	931	*MS4A1*	TTCAATCATGGACATACTTAA	SI00131572	TTGGGCTAACTGAAACATCTT	SI03025337	CAGGAATCCTCACCAATAGAA	SI03069059	CCCATCTACCCAATACTGTTA	SI03077123
900097-4	F7	6513	*SLC2A1*	CAGGCTTGAAATCGCATTATT	SI00089257	CAGACACTAAGTTATAGTATA	SI00089271	CAGCTGGATGAGACTTCCAAA	SI03068436	CTCCACGAGCATCTTCGAGAA	SI03089401
900097-4	F8	7262	*PHLDA2*	AAGTACGTGTACTTCACCATC	SI00301182	CGCGCTCGGCACGACATGAAA	SI02225636	CTGGTTCTTTGTAGTCACATA	SI02225643	GCGCCTTAAGTTATTGGACTA	SI00050561
900097-4	F9	25803	*SPDEF*	CGGGAATGGATAATAAAGATA	SI00731311	CAAGGAGAAGGGCATCTTCAA	SI00731318	CCGCTTCATTAGGTGGCTCAA	SI00731325	CAAGAAGGGCATCATCCGGAA	SI00731332
900097-4	F10	10291	*SF3A1*	CAGGATAAGACGGAATGGAAA	SI00082194	CGCAAGGATTATGATCCCAAA	SI00082201	CAGCATGTAGGTAGCGTCCTA	SI00082208	CTCATTCAGGAGCGCTATGAA	SI03089233
900097-4	F11	55450	*CAMK2N1*	AGCAAGCGGGTTGTTATTGAA	SI02224873	CGGGTTGTTATTGAAGATGAT	SI02224880	AAGGCACCTCCTGGTGTCTAA	SI03035235	CAAGCTATCTGCCCAAGTTAA	SI04440037
900097-4	G2	8170	*SLC14A2*	AAGGGCATCAATCATAACAAA	SI00720111	CTCGGAGAAGTTAGACTACTA	SI00720118	CAGGAGTGTATTTCACATCGA	SI00720125	AAGCCTCATCCTTAAAGAGAA	SI00720132
900097-4	G3	775	*CACNA1C*	CTGGTTTGGTTCGGTTATCTA	SI00337176	ACCGAGGTAAACCCAGCTGAA	SI03039862	GTGGGTAGCATTGTTGATATA	SI03107167	TCCAGGGATGTTAGTCTGTAT	SI03113768
900097-4	G4	1659	*DHX8*	CCCAGAGAAGTGGGAGATCAA	SI03019373	CCGGGAGAGGAATAAAGTGAA	SI00363909	CTGGAGAAGATCTAAACCCAA	SI00363916	CAGCAGGTGGTCTATATCCAT	SI04241797
900097-4	G5	8228	*PNPLA4*	AAGGATTTGAGAAATAGGCAA	SI00688303	ACCAAGTAGCCTAGTAAATAA	SI00688310	AAACATGTAGTTTAATCTTAA	SI00688317	CCCAGTTTAGGAGATTATCAA	SI00688324
900097-4	G6	9138	*ARHGEF1*	CAGCAGCTCTGAGAACGGCAA	SI00302680	CAAGAAGAAATTGGTCCACGA	SI00302694	CACCGATCACAAAGCCTTCTA	SI03161900	TAGGGCTGACCTCATCTCCGA	SI03228771
900097-4	G7	200081	*TXLNA*	CCGGACCTATGTGGCAAGGAA	SI00159467	CGCGAGGAGCATATCGACAAA	SI00159474	AGCCGCCAACTGGAAGACATA	SI03043376	CAGCGAGGTATTCACCACATT	SI03066903
900097-4	G8	8664	*EIF3D*	TCGGAACATGTTGCAGTTCAA	SI00377797	CAGTTTGGTGGTGGAAGTCAA	SI00377811	CAGGAGCCACATACCAAGATA	SI00377818	GCCCTAGAATACTACGACAAA	SI03219741
900097-4	G9	8668	*EIF3I*	CAGATTAAGTATAACCGCGAA	SI00377713	AAGAAATAAATTGGTTTGGTA	SI00377720	AAGGACCCTATCGTCAATGTA	SI00377734	TCCGAGCCAGATTGACAACAA	SI04162368
900097-4	G10	51327	*ERAF*	CTGGATGAACTTCTACATCAA	SI00380793	AAGGCAGATGGCTCTTCTTAA	SI00380800	CAGGACTTCCTGAAGTCTCA	SI00380807	CCGCAGGATTGAAGGAGTTCA	SI00380814
900097-4	G11	1374	*CPT1A*	CAGCAAGCACATCGTCGTGTA	SI00029407	CTGGATGGGTATGGTCAAGAT	SI00029414	AACGATGTACGACCAATCGA	SI00029421	AGCGTTCTTCGTGACGTTAGA	SI03044202
900097-5	B2	29929	*ALG6*	CCGCCTATGTTTGGTGATTAT	SI00101598	CCGGTTGATCGTGGATTATTT	SI00101605	ACAGCTTATCATAGTCTCCTA	SI00101612	AGGCCTTATTCTTATAGACTA	SI00101619
900097-5	B3	4839	*NOL1*	CAAGAAGGATCTGGCCATTTA	SI00659974	CAGGAAATTCTGAAACAGCCA	SI00659981	CAGATCAATGTGGATGAGGAA	SI00659988	AAGCGTTGCTGCCCATTGAAA	SI03131121
900097-5	B4	825	*CAPN3*	ACCATGCGGTACGCAGACAAA	SI03038798	CCACCTCTGGAACAAGATTAA	SI03074554	CCGAGCCGCCTCGGTTCTGAA	SI03080147	TAGCATGATTGCGCTCATGGA	SI03111003
900097-5	B5	32	*ACACB*	CCCGCTGTGCATAGAAATGTA	SI00021798	TACCGTGGAATACCTCATTAA	SI00021805	AAGGATCGATCCAGCTTACAA	SI00021812	AAGGGTTGCGTGAAAGACGTA	SI00021819
900097-5	B6	8532	*CPZ*	TACGCCAAAGTCATCAAGAAA	SI00054229	CCGGATCTCAGTCAAAGGCAT	SI00054236	CGCGCAGAACCTGGATCTGAA	SI00054243	CCCGGAGACAAAGGCAATCAT	SI00054250
900097-5	B7	29028	*ATAD2*	CTGGTACTTAGGCACCATAAA	SI00103131	AAGGCATTTATAAAGATCGAA	SI00103138	AAGAATAATTAGCAGCGTTAA	SI00103145	CCGGATAAAGAAGAACGGACA	SI02640232
900097-5	B8	22938	*SNW1*	AACGTCTGGCTGCTGATGGAA	SI00301812	CCCGATGAAGAAGCTATTAAA	SI02655478	TCGGATGGTAGAAATGCAGAA	SI00098609	CACAGGTCTCTCCAAAGTGAA	SI00098616
900097-5	B9	3851	*KRT4*	AAGGTGCAGTTCTTAGAGCAA	SI00464828	CGAGGTGAGAATGCCCTTAAA	SI00464835	TCCATCTACATCAGAACCAAA	SI00464842	CAGTCTTAATGACGAGATCAA	SI00464849
900097-5	B10	10376	*TUBA1B*	TCCATCATATCTCAAAGTAAA	SI00084308	AAGGAGAGGAATACTAATTAT	SI00084329	CCCGCCCTAGTGCGTTACTTA	SI02636627	CAGCTTAACTGACAGACGTTA	SI02636634
900097-5	B11	30837	*SOCS7*	CCGAAAGTTCTACTACTATGA	SI00729218	CCACACTAGAATGGAGCACTA	SI03074400	GAGAGCCATTATGCACTCCAA	SI03102687	TCCCGATTCAGCAATGTCAAA	SI03114356
900097-5	C2	6193	*RPS5*	CTCGAACTCCTATGCCATTAA	SI00707966	CCGGGAGGACTCCACACGCAT	SI00707980	CGCGTGGTCTACGCCGAGTGA	SI03195031	TTCCCAGCTGCTGCCCAATAA	SI03241469
900097-5	C3	8436	*SDPR*	CAGAGGATAATTAAGATACTA	SI03246789	TACGTAGTAAAGTCAGGACTA	SI04366915	TAAGCTAAACATATAACTATA	SI00713328	CCGGGACAACTCACAGGTGAA	SI02656738
900097-5	C4	9994	*CASP8AP2*	CAGCTGATGTGCGGAAGTCAA	SI00097272	AACGAAGTTTCGAGATTCTTA	SI00097279	CAGTCTGATCTCAATAAGGAA	SI02638937	CACATACGTAGATCTAACGAA	SI03056501
900097-5	C5	10135	*PBEF1*	TACAAGGTTACTCACTATAAA	SI00080836	ATGGGTTGCAGTACATTCTTA	SI00080850	TTGTTCAGTCCTGGTATCCAA	SI03025897	CAGAGTGTTACTGGCTTACAA	SI03064432
900097-5	C6	81488	*GRINL1A*	GACCCGATACTTGATACTTCA	SI03101553	TCCAGAAGATACCGCTCTAAA	SI03113579	ACCAGGGACTTGGACGTCCTA	SI03038644	ACCCTGTTAGTTTAGACTGTA	SI03039603
900097-5	C7	6945	*MLX*	CTGGATCGAGGAGCACTGTAA	SI03097535	CAGATTTCTGCTCATGATCTA	SI00741510	CAAGGATGTCACCGCCCTAAA	SI00741517	CAGGTCAAGTTCAACGTGTTT	SI00741524
900097-5	C8	288	*ANK3*	CGCATCCTTAGCTTTACGTAA	SI02780204	CAGGATTATCACGAAAGATTT	SI00128611	CTAGTCGATTATTTAAGCATA	SI02625385	AAGGATCTTAACCTCAATTAA	SI02625399
900097-5	C9	6187	*RPS2*	ATCAGAGATCATTGATTTCTT	SI03148796	CAAGTTGGGCCGCTTGGTCAA	SI03158197	CCTGCCTATTAAGGAATCAGA	SI03192525	GAAGGAGACTGTATTCACCAA	SI03215429
900097-5	C10	3921	*RPSA*	TCGACATGAGTTGTACTTCTA	SI00467964	TACCTGGGATTGCATATCAAA	SI00467971	TTGCATATCAAAGCATAATAA	SI00467978	AGGCTCTTAAGCAGCATGGAA	SI03045371
900097-5	C11	10244	*RABEPK*	AAAGAGGATTCAGCTGACAAA	SI00081732	CAGCATTGGACCTTGCTTAAA	SI00081753	AAGCAACATGGTACACCTTGA	SI02636116	CTGGACGATTGGACCATTCCA	SI03096758
900097-5	D2	4650	*MYO9B*	CAGCATGAAGGACGTCCTCAA	SI00653709	ACCGCGATTTGCATAACCAAA	SI00653716	TACCGCGATTTGCATAACCAA	SI03224844	CCAGCCGGTTTGGGAAATTCA	SI04211942
900097-5	D3	5054	*SERPINE1*	TTGGAGGACCTTTAGGTCAAA	SI00012628	TTCACGAGTCTTTCAGACCAA	SI03021599	ACCGACATGTTCAGACAGTTT	SI03039715	CACGGTCAAGCAAGTGGACTT	SI03061198
900097-5	D4	9752	*PCDHA9*	CGGGATCTTCTGTTTCAACAA	SI02640141	TTCTCTTACACTAGAATCCTA	SI03023363	CAGTAGATTTCTGGGCTCCTA	SI03072734	CTGAGTATTTGAAATGTTATA	SI00141162
900097-5	D5	10403	*NDC80*	CCGAGACCACTTAATGACAAA	SI02653224	TCCCTGGGTCGTGTCAGGAAA	SI02653567	TCCAAAGGTTATGACTTTGAA	SI00084525	ATGAATTGCAGCAGACTATTA	SI00084532
900097-5	D6	63929	*XPNPEP3*	CACTAGCTACCTACCAAACAA	SI02778559	TCGGTGATTGTGGGCGGCCAA	SI02778566	TGGGATGGTAATCACAATTGA	SI00132524	CTGGTGGTAATCGGTCAAACA	SI00132531
900097-5	D7	11015	*KDELR3*	TTGGCCTTTCCTTCCTTGAAA	SI03025029	CACCAGGTACCTGGACCTGTT	SI03057397	CTGTGCCTATGTTACAGTGTA	SI03100580	CCGCCAGAAAGTTTCCTAGAA	SI02638181
900097-5	D8	10856	*RUVBL2*	CCGGAGATCCGTGATGTAACA	SI00709247	CACGCAGTACATGAAGGAGTA	SI00709254	AACCGTTACAGCCACAACCAA	SI00709261	CCAGGACGCCTTCCTCTTCAA	SI00709268
900097-5	D9	56126	*PCDHB10*	AAGGAATTAATTACTATTATA	SI00679315	ATGCCTTATAATTGAAATAAA	SI00679322	TTGGATTTAATATTCAGTAAA	SI00679329	CAAATTTAAGTTATTATGCAA	SI00679336
900097-5	D10	1687	*DFNA5*	GCGGTCCTATTTGATGATGAA	SI00363013	TAGAAAGAGTCTGACCCTTTA	SI00363020	AACATACTAATTCTATGTCTA	SI00363027	CTGATAAGTTACAGCTTCTAA	SI00363034

900097-5	D11	9960	*USP3*	AGCGCTCTAAGAATCAAGAAA	SI00089432	CTGGATCGGATAAACTTTAAT	SI00089439	CAGAAGTAAGCGCTCTAAGAA	SI00089446	CAGGGCGGTTTCAACGGTGTT	SI03071432
900097-5	E2	10712	*C1orf2*	CCCAATTGCCTGCTCCATTAA	SI00323120	TTCAGGGACCTTTATACCAAA	SI00323134	CACACGCTGTCTGTGACTAAA	SI04435228	AAGAAGGAATCGGATATAACA	SI00003276
900097-5	E3	6203	*RPS9*	ACGGCGTCTGTTCGAAGGCAA	SI00708071	CCCGCGGAGACCCTTCGAGAA	SI00708078	CCCGGGCCGCGTGAAGAGGAA	SI00708092	CGGAGCTGGGTTTGTCGCAAA	SI04359782
900097-5	E4	3309	*HSPA5*	TAGGGTGTGTGTTCACCTTCA	SI02780554	TGGGATAAGGAAACACTTCTA	SI02781016	CAAGCCCAATACAGCCATTAA	SI00075922	AACTGTTACAATCAAGGTCTA	SI00075929
900097-5	E5	10905	*MAN1A2*	TCACTTTAATTTCATAATAAA	SI00627375	CTGGCTGAATTTGGTACACTA	SI00627382	AAGACTTAATTAACTAATATA	SI00627389	AAGAATATTTATAGAATTCCA	SI00627396
900097-5	E6	2074	*ERCC6*	ATGGATGGTACCACTACAATA	SI00002485	CAGAGCGTTTAGAAAGTGAAA	SI03064075	CAGGACTCGTGGTTCAAATTA	SI03069402	CAGGATGACATTAGCAGGTAT	SI03069934
900097-5	E7	7311	*UBA52*	AAACTGCCGCAAGAAGAAGTG	SI02780764	CTCCGCCAGCTTGCCCAGAAA	SI00050939	CCCAATGGTGTAGAACGTTGA	SI03076255	CTCCTATGATAATCACTCTTA	SI03090465
900097-5	E8	2335	*FN1*	CCGGTTGTTATGACAATGGAA	SI02663997	CCCGGTTGTTATGACAATGGA	SI02664004	TACCGAGTGGGTGACACTTAT	SI03108945	AACCGGGAACCGAATATACAA	SI00031682
900097-5	E9	2877	*GPX2*	AACCCTCTGGTTGGTGATTCA	SI02627709	CCGCACCTTCCCAACCATCAA	SI02627716	CCGGGACTTCACCCAGCTCAA	SI02627723	CAGAATGATGGCACCTTCCTA	SI03063340
900097-5	E10	5519	*PPP2R1B*	CAGGAAATAACTACTAAGCAA	SI02653364	CAGAAGTTAGGTCAAGATGAA	SI02653763	CTGACGTTCGTTTGAATATCA	SI02659433	ATCGACGAGCTCCGCAATGAA	SI03047996
900097-5	E11	80824	*DUSP16*	AACCCAGTTGTTACTCTCTTA	SI02759421	AAGGTTGTAGTTTACGATCAA	SI00374829	CCGGCCATTTGTGGAATACAA	SI00374836	CAGGACAAAGTGTTAATTACA	SI00374843
900097-5	F2	6372	*CXCL6*	ACGCGTTACGCTGAGAGTAAA	SI00046669	TACGCTTCTCCCTGAAGTTTA	SI00046676	CAAGTTTGTCTGGACCCGGAA	SI00046683	TACCGTTATCTGTGCAGAATA	SI03109099
900097-5	F3	221150	*C13orf3*	CAGCTGGAAAGTATTATCTAA	SI00317940	AGGGAATATATTCAAAGTGTA	SI00317947	AAAGACAAACATGAACATTAA	SI00317954	AAGAGAGTATTTCCAGAAGTA	SI04327995
900097-5	F4	10742	*RAI2*	CCGGAGCATAAAGTTAAAGAA	SI00697963	TAGGATGAATATTTCACAATA	SI00697970	AAGCTGAGAATTAGGCTTAAA	SI00697977	CACCGAGGCCTGGAACATCAA	SI00697984
900097-5	F5	7980	*TFPI2*	ATGGTTGTATCTGAAGAATAA	SI00743519	CAGAAGAGGATCATAACTGAA	SI00743526	CAGCATGAGGAAACAAATCAT	SI00743533	TTGCAATGCATAAGATATAAA	SI00743540
900097-5	F6	10426	*TUBGCP3*	CACGACTCGCATGGACTTTAA	SI00087465	CCACTACCTAAGAGTATTTAA	SI00087486	AAAGAGCTTATTCGACAACGA	SI03026464	CTCGATTATCATGTTGACGGA	SI03091235
900097-5	F7	11274	*USP18*	TAAGCGCTTCCTGGAAGTGAA	SI00118034	AAGATGGAGTGCTAATGGAAA	SI00118041	TTCGCTTTCCATTCAGTGGAA	SI03022915	CTGGATCTACGGAGTCTTCTA	SI03097570
900097-5	F8	3902	*LAG3*	CAGCAGCCCAGTCCAAATAAA	SI00467796	CAACGTCTCCATCATGTATAA	SI00467803	CCGGTCTTTCCTCACTGCCAA	SI00467810	CCGCAGGCTCAGAGCAAGATA	SI00467817
900097-5	F9	8303	*SNN*	CAGGGCAGAGGTGAACTTGAA	SI00728203	AGGGATGTCATCCTTCTTCAA	SI00728210	CTGCATAGACTCACTTGTTAA	SI00728217	CTGGAGGGTCATAGGCTTGTA	SI00728224
900097-5	F10	9112	*MTA1*	GCGCATCTTGTTGGACATATT	SI02757699	CAGCATCATTGAGTACTACTA	SI00067130	TTGGACATATTGGAAGAAATA	SI00067151	CAGGGTCGGAGACTACGTCTA	SI02633736
900097-5	F11	79154	*MGC4172*	CCCAGTGGATTTCATGGTGAT	SI02778804	TGGGCACATCATTAACATCAA	SI00136052	CGCCTTCAAACTCCACGACAA	SI00136066	CAGATGTGACCTATCAAATGA	SI00136073
900097-5	G2	55506	*H2AFY2*	CTCAAAGAAGATATAGGTAAA	SI00433076	CAGCTTGAAGAGACCATCAAA	SI00433083	AAGAAGGAACTTCAAATTCCA	SI00433090	CACAGATAGAACCCAAAGAAA	SI00433097
900097-5	G3	1312	*COMT*	CTGGAACGAGTTCATCCTGCA	SI00014868	CTGGCTAGCTATATTATCTTA	SI00014875	CCAGCTGAAGAAGAAGTATGA	SI03075191	CGGCCTGGAGAAGGCCATCTA	SI03086251
900097-5	G4	10346	*TRIM22*	ACCGCTAATGCCTTAATGAAA	SI00750708	AAGAGTGTATTCCGAGTACCA	SI03032057	CCGCATAAACGAGGTGGTCAA	SI03080644	CTCCAGATATAGACCTCAATA	SI03089485
900097-5	G5	55294	*FBXW7*	CCCTAAAGAGTTGGCACTCTA	SI00146636	AAACATATGATGCAAGTGATA	SI03026023	CTCATTGATAGTTGTGAACCA	SI03089240	CTGGAGGCGAGGAGAACTCAA	SI03097241
900097-5	G6	2197	*FAU*	AAGCCACTTAGTTCAGTCAAA	SI00031122	CCGCCGTTCAGTCGCCAATAT	SI02627499	GGCCGCATGCTTGGAGGTAAA	SI02627513	AAGTGAGAGGTCAGACTCCTA	SI03036908
900097-5	G7	5713	*PSMD7*	AAGAATAGTTGGCTGGTACCA	SI00301483	CCGAATCGGCAAGGTTGGAAA	SI00043596	TTCCGTATTGGTCATCATTGA	SI00043610	CACGTGACCAGTGAAATTGGA	SI03061541
900097-5	G8	29106	*SCG3*	AACACTGATTACTATCATGAA	SI02639749	CAGACATTGAGTTTACTTATA	SI02639756	TTCCCAAATTTCTATGCGCTA	SI03022138	CAGAGCCGTGTTTGACAAGAT	SI03064026
900097-5	G9	11331	*PHB2*	AACCCAGGAATTCTCAATAAA	SI02780918	CAGCCTCATCAAGGGTAAGAA	SI03066504	CTGGATGATGTGGCCATCACA	SI03097633	CAGAATCGTATCTATCTCACA	SI00096124
900097-5	G10	4809	*NHP2L1*	CAGCTACTCTCTATTGTTATA	SI00658539	CAGGGTGGTGGCACCGTTGAA	SI00658546	AACAGAGATGGTGGAGTCCAA	SI00658560	CAAGTGTCGTTTACTAATGTA	SI03158127
900097-5	G11	226	*ALDOA*	CAGGAGGAGTATGTCAAGCGA	SI03069675	CAGGCCATGCTTGCACTCAGA	SI03070340	CAGGGACAAATGGCGAGACTA	SI03071005	TGCCAAATAAACAGCTATTTA	SI03118045
