# Supplementary material for: An RNA Interference Lethality Screen of the Human Druggable Genome to Identify Molecular Vulnerabilities in Epithelial Ovarian Cancer
Source: PLoS One. 2012 Oct 9;7(10):e47086. doi: 10.1371/journal.pone.0047086 (PMC3467214; doi:10.1371/journal.pone.0047086)
Supplement: Table S3 — List of the eight epithelial ovarian cancer (EOC) tumorigenic and the three human immortalized ovarian surface epithelial (HIO) non-tumorigenic cell lines used in this study. The EOC cell lines have been selected to represent epithelial serous histotype, which is the major subtype of ovarian cancer. The transfection conditions were optimized for cell seeding density per well, dilution of the lipid-based transfection reagent, and the final siRNA concentration for each of the cell lines used in the study. The following ranges for each parameter were evaluated during optimization: cell densities (6.5×10^3^–1×10^4^ per well); lipid dilution (1∶250–1∶1000); siRNA concentration (50 nM–100 nM). (DOC) [file pone.0047086.s009.doc]

**Supplementary Table S3**: **Cell line information**. List of the eight epithelial ovarian cancer (EOC) tumorigenic and the three human immortalized ovarian surface epithelial (HIO) non-tumorigenic cell lines used in this study. The EOC cell lines have been selected to represent epithelial serous histotype which is the major subtype of ovarian cancer. The transfection conditions were optimized for cell seeding density per well, dilution of the lipid-based transfection reagent, and the final siRNA concentration for each of the cell lines used in the study. The following ranges for each parameter were evaluated during optimization: cell densities (6.5*10^3^ - 1*10^4^ per well); lipid dilution (1:250 - 1:1000); siRNA concentration (50 nM -100 nM).

| **Cell line** | **Subtype** | **Reference** | **Optimized conditions for siRNA transfection** | | |
| --- | --- | --- | --- | --- | --- |
|  |  |  | **Cell seeding density *10^3^/well** | **Final DharmaFECT-1 dilution** | **Final siRNA concentration (nM)** |
| A1847 | Serous carcinoma |  | 6.5 | 1:800 | 50 |
| SKOV 3 | Serous carcinoma |  | 6.5 | 1:500 | 50 |
| OVCAR 5 | Serous carcinoma |  | 7 | 1:250 | 50 |
| OVCAR 8 | Serous carcinoma |  | 8.0 | 1:250 | 50 |
| UPN275 | Mucinous | (11) | 7 | 1:1000 | 50 |
| A2780 | Serous carcinoma |  | 6.5 | 1:1000 | 50 |
| CP70 | Cisplatin-resistant derivative of A2780 |  | 7 | 1:1000 | 50 |
| C30 | Cisplatin-resistant derivative of A2780 |  | 7 | 1:1000 | 50 |
| HIO 80 | Non-tumorigenic human immortalized ovarian surface epithelial cells |  | 7 | 1:1000 | 50 |
| HIO 117 | Non-tumorigenic human immortalized ovarian surface epithelial cells |  | 7 | 1:1000 | 50 |
| HIO 120 | Non-tumorigenic human immortalized ovarian surface epithelial cells |  | 8 | 1:1000 | 50 |

**References for Table S1**

1. Fu M, Maresh EL, Soslow RA, Alavi M, Mah V, Zhou Q, et al. Epithelial membrane protein-2 is a novel therapeutic target in ovarian cancer. Clin Cancer Res. 2010 Aug 1;16(15):3954-63.

2. Alessandra Eva KCR, Philip R. Anderson, Alagarsamy Srinivasan, Steven R. Tronick, E. Premkumar reddy, Nelson W. Ellmore, Angela T. galen, James A. Lautenberger, Takis S. Papas, Eric H. Westin, flossie Wong-Staal, Robert C. Gallo & Stuart A. Aaronson Cellular genes analogous to retroviral *onc* genes are transcribed in human tumor cells. Nature. 1982;295(4).

3. Berger S, Siegert A, Denkert C, Kobel M, Hauptmann S. Interleukin-10 in serous ovarian carcinoma cell lines. Cancer Immunol Immunother. 2001 Aug;50(6):328-33.

4. Shoemaker RH, Monks A, Alley MC, Scudiero DA, Fine DL, McLemore TL, et al. Development of human tumor cell line panels for use in disease-oriented drug screening. Prog Clin Biol Res. 1988;276:265-86.

5. Pitteri SJ, JeBailey L, Faca VM, Thorpe JD, Silva MA, Ireton RC, et al. Integrated proteomic analysis of human cancer cells and plasma from tumor bearing mice for ovarian cancer biomarker discovery. PLoS One. 2009;4(11):e7916.

6. Egan K, Crowley D, Smyth P, O'Toole S, Spillane C, Martin C, et al. Platelet adhesion and degranulation induce pro-survival and pro-angiogenic signalling in ovarian cancer cells. PLoS One. 2011;6(10):e26125.

7. Godwin AK, Meister A, O'Dwyer PJ, Huang CS, Hamilton TC, Anderson ME. High resistance to cisplatin in human ovarian cancer cell lines is associated with marked increase of glutathione synthesis. Proc Natl Acad Sci U S A. 1992 Apr 1;89(7):3070-4.

8. Roland IH, Yang WL, Yang DH, Daly MB, Ozols RF, Hamilton TC, et al. Loss of surface and cyst epithelial basement membranes and preneoplastic morphologic changes in prophylactic oophorectomies. Cancer. 2003 Dec 15;98(12):2607-23.

9. Auersperg N, Maines-Bandiera S, Booth JH, Lynch HT, Godwin AK, Hamilton TC. Expression of two mucin antigens in cultured human ovarian surface epithelium: influence of a family history of ovarian cancer. Am J Obstet Gynecol. 1995 Aug;173(2):558-65.

10. Capo-Chichi CD, Smith ER, Yang DH, Roland IH, Vanderveer L, Cohen C, et al. Dynamic alterations of the extracellular environment of ovarian surface epithelial cells in premalignant transformation, tumorigenicity, and metastasis. Cancer. 2002 Oct 15;95(8):1802-15.

11. Personal communications, A.K. Godwin
