# Supplementary material for: An RNA Interference Lethality Screen of the Human Druggable Genome to Identify Molecular Vulnerabilities in Epithelial Ovarian Cancer
Source: PLoS One. 2012 Oct 9;7(10):e47086. doi: 10.1371/journal.pone.0047086 (PMC3467214; doi:10.1371/journal.pone.0047086)
Supplement: Supplementary Information S1 — (DOC) [file pone.0047086.s012.doc]

**Supplementary Information**

**Statistical analysis for siRNA HTS**: For every screening experiment, the transfection efficiency was calculated using the positive control (PLK1 siRNA, 4 wells/plate) and negative control (either no siRNA (mock transfection, 8 wells/plate) or GL2 siRNA (4 wells/plate)) treated wells included on each screen plate and reported as a Z’-factor as described previously. In the primary screen, 42% of the plates had an average Z’ factor of 0.5 or greater; in the secondary screens, 100% of the plates had an average Z’-factor of 0.5 or greater. Data from plates where the Z’-factor fell below 0.5 were not excluded.

For the primary screen using the siRNA library targeting the druggable genome, two statistical methods were employed to analyze the siRNA screening data and identify hits, negative control normalization and B-score normalization. In the first method, negative control normalization, the raw fluorescence intensity values from the CTB readings collected following HTS were normalized to the median fluorescence intensity values from the internal reference controls on each plate to yield a normalized viability score (defined as the (fluorescence intensity_sample_)/(median fluorescence intensity_reference_)) corresponding to each gene. Mock transfected wells (8 wells/plate) were used as the internal reference controls for data analysis of the primary screen. An average normalized viability score from the two technical replicates was calculated for each gene. Genes were then ranked based on their viability score. All genes yielding a viability score ≤ 0.85 were selected as hits (n=222). In order to account for potential row or column effects associated with the primary screen, we also employed a second normalization method, the B-score method for each plate , a non-control based normalization method used to correct for the row and column artifacts. The B-score method yielded an average B-score for the technical replicates and a false discovery rate (FDR) associated with each gene. Genes which did not yield a viability score of 0.85 or less from the negative control normalization method were then ranked based on their B-score and FDR. Those genes with a positive B-score and a FDR of ≤ 0.25 were selected as additional hits (n=78). Therefore, a total of 300 genes (222 from the negative control normalization and 78 from the B-score normalization) were selected as hits from the primary screen and taken forward for the secondary round of HTS. All calculations were automated using the cellHTS and cellHTS2 packages within the Bioconductor open source software package ([http://bioconductor.org](http://bioconductor.org/)).

In the secondary screens, the negative control normalization method described above was used to analyze the siRNA screening data and identify hits. However, the median fluorescence intensity values from the CTB readings for the GL2 siRNA transfected wells (4 wells/plate) were used as the internal reference controls for data analysis of the secondary screens. An average normalized viability score from the two technical replicates was calculated for each gene. Genes were then ranked based on their viability score. LIMMA methodology (Linear Models for Microarray Data) was applied to the normalized viabilities. Hits were identified based on statistical significance as well as biological significance. Statistical significance was determined by a p-value adjusted false discovery rate (FDR) using the Benjamini-Hochberg method to account for multiple testing. Hits showing an FDR of less than 5% were considered statistically significant. Biological significance was measured by a change of at least 15% in viability relative to GL2 controls. The fluorescence intensity values from the CTB readings following the deconvolution screens were analyzed as described for the secondary screens.

**qRT-PCR**: Quantitative RT-PCR (qRT-PCR) was performed using TaqMan assays (Applied Biosystems). To quantify the mRNA levels of a specific gene following siRNA treatment, each siRNA-treated sample (either siRNA against the gene of interest or siRNA against GL2) was analyzed in triplicate by qRT-PCR for the gene of interest and in triplicate for the internal control reference gene, *PPIA* (cyclophilin A). The cycle threshold (Ct) values were determined from the amplification curves for each sample and then normalized to the level of *PPIA* in that sample to calculate a ΔCt for each siRNA treated sample as follows: ΔCt_gene of interest or GL2 siRNA treated_ = Ct_gene of interest or GL2 siRNA treated_ - Ct_PPIA_. The amount of mRNA remaining following siRNA treatment was calculated as follows: 2^-ΔΔCt^, where ΔΔCt = ΔCt_gene of interest siRNA treated_ - ΔCt_GL2 siRNA treated_. The percent of gene knockdown following siRNA treatment was calculated as follows: (1-2^-ΔΔCt^)*100.

**References**

1. Zhang, J.H., T.D. Chung, and K.R. Oldenburg, *A Simple Statistical Parameter for Use in Evaluation and Validation of High Throughput Screening Assays.* J Biomol Screen, 1999. **4**(2): p. 67-73.

2. Birmingham, A., et al., *Statistical methods for analysis of high-throughput RNA interference screens.* Nat Methods, 2009. **6**(8): p. 569-75.

3. Ngo, V.N., et al., *A loss-of-function RNA interference screen for molecular targets in cancer.* Nature, 2006. **441**(7089): p. 106-10.

4. Brideau, C., et al., *Improved statistical methods for hit selection in high-throughput screening.* J Biomol Screen, 2003. **8**(6): p. 634-47.

5. Boutros, M., L.P. Bras, and W. Huber, *Analysis of cell-based RNAi screens.* Genome Biol, 2006. **7**(7): p. R66.

6. Gentleman, R.C., et al., *Bioconductor: open software development for computational biology and bioinformatics.* Genome Biol, 2004. **5**(10): p. R80.

7. Smyth, G.K., *Linear models and empirical bayes methods for assessing differential expression in microarray experiments.* Stat Appl Genet Mol Biol, 2004. **3**: p. Article3.

8. Benjamini, Y.H., Yosef, *Controlling the false discovery rate: a practical and powerful approach to multiple testing.* J. Roy. Statist. Soc, 1995. **Ser. B 57**(no. 1): p. 289–300.
